# Regeneration‐Optimized Chemisorbents: Evaluating Hybrid Coordination Networks for Atmospheric Water Harvesting

**DOI:** 10.1002/anie.6095254

**Published:** 2026-09-21

**Authors:** Xiang‐Jing Kong, Asif Raza, Lilia Croitor, Andrey Bezrukov, Xia Li, Angela Shipman, William Morris, Carlos J. Solanilla‐Salinas, Adam Hogan, Brian Space, Volodymyr Bon, Stefan Kaskel, Soumya Mukherjee, Alan C. Eaby, Tao He, Michael J. Zaworotko

**Affiliations:** ^1^ Bernal Institute and Department of Chemical Sciences University of Limerick Limerick Ireland; ^2^ Department of Chemical Engineering College of Materials Science and Engineering Beijing University of Technology Beijing China; ^3^ Department of Chemistry North Carolina State University Raleigh North Carolina USA; ^4^ Department of Inorganic Chemistry Technische Universitat Dresden Dresden Germany

**Keywords:** atmospheric water harvesting, crystal engineering, desiccant, hybrid coordination network, regeneration‐optimized sorbents

## Abstract

Atmospheric water harvesting (AWH) can be enabled by solid desiccants that exhibit S‐shaped adsorption isotherms with one or more steps at low relative humidity (RH, <30%) and mild regeneration temperatures (<60°C). There remains a dearth of such regeneration‐optimized sorbents (ROSs), which thus far are limited exclusively to physisorbents mainly because chemisorbents typically require elevated temperatures for water recovery. Herein, the first comprehensive water sorption study of the **SIFSIX‐3‐Ni** family of hybrid coordination networks (HCNs) and a new, related family of HCNs, reveals a shared cooperative chemisorption mechanism with markedly different RH thresholds and regeneration requirements. Whereas the **SIFSIX‐3‐Ni** family operates outside the RH and regeneration limits of ROSs, four **M′FSIX‐25‐M** analogues (M = Co^2+^, Ni^2+^; M' = Si^4+^, Ti^4+^, **25** = 1,4‐bis(imidazol‐1‐yl)naphthalene) exhibit both low‐RH chemisorption and regeneration from 34‐44°C. The lead regeneration optimized chemisorbent, **ROC‐1** (**TIFSIX‐25‐Ni**), has a projected water‐harvesting productivity of ca. 3.5 ${\mathrm{kg}}_{H_{2}O}$ L_sorbent_^−1^ d^−1^. Density functional theory calculations provide insight into the energetics and mechanism of hydration/dehydration in these first‐generation ROCs. This work establishes ROCs as a new class of desiccants that offers reversible chemisorption within the low‐RH and mild‐regeneration conditions relevant to AWH in arid environments.

## Graphical abstract

Atmospheric water harvesting (AWH) under arid conditions requires desiccants that load water below 30% RH and regenerate below 60°C, a performance regime traditionally limited to physisorbents. Here, we show that the inherent hydrolytic instability of **M′FSIX‐25‐M** coordination networks enable regeneration‐optimized chemisorbents (ROCs) with projected water harvesting productivities of 3.5 ${\mathrm{kg}}_{H_{2}O}$ L_sorbent_^−1^ d^−1^, demonstrating the potential utility of chemisorbents for AWH.

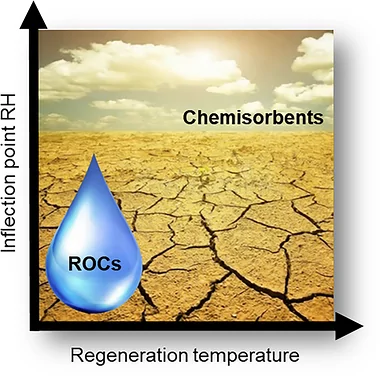

## 1 Introduction

Porous coordination polymers [1] (PCPs), also known as metal–organic frameworks [2] (MOFs), porous coordination networks (PCNs) [3] or metal–organic materials [4, 5] (MOMs), represent a versatile class of porous materials that have garnered considerable attention for their potential utility as sorbents to reduce the energy footprint of gas separation, gas storage, wastewater treatment, and atmospheric water harvesting (AWH) technologies [6, 7, 8, 9, 10, 11, 12, 13, 14, 15]. Hybrid coordination networks (HCNs) [6], CPs sustained by both inorganic and organic linkers, have exceptional compositional modularity as the metal node (M), N‐donor linker (L), and bridging anion (A = M'O_x_F_6‐x_) can each be varied independently, enabling systematic control over pore size and chemistry [16]. The prototypical HCN, **SIFSIX‐1‐Zn** [17] (SIFSIX = SiF_6_^2−^, 1 = 4,4'‐bipyridine, see Text S3 for details on nomenclature), is comprised of square lattice (**sql**) CNs pillared by SIFSIX anions to form a primitive cubic, **pcu**, topology HCN. A **pcu** topology analog, the hybrid ultra‐microporous material (HUM) [18] **SIFSIX‐3‐Zn** (**3** = pyrazine, Scheme 1), was subsequently found to exhibit benchmark CO_2_ capture properties over N_2_ and CH_4_ [6], providing motivation to systematically study structure–function relationships in such materials by exploiting the node‐and‐linker approach [19]. Several variants have subsequently emerged as benchmark sorbents for gas separation [6, 20, 21, 22].

**SCHEME 1:**
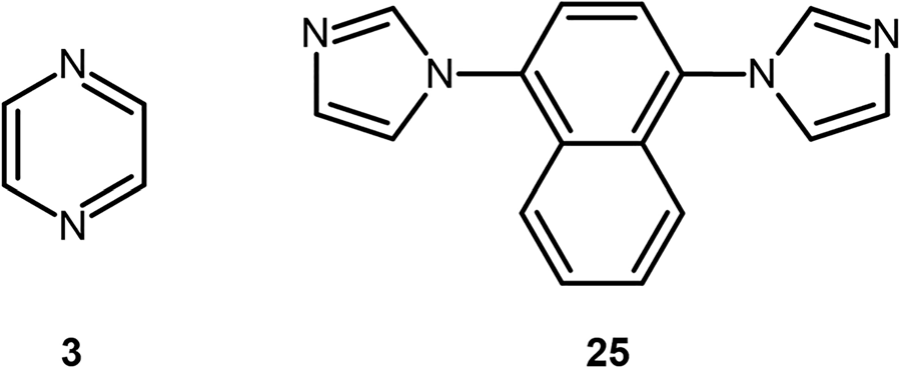
Pyrazine, **3**, and 1,4‐bis(imidazol‐1‐yl)naphthalene), **25**.

That humidity can influence the sorption performance of HCNs often leads to negative impacts, including reduced uptake capacities because of co‐adsorption [23] and hydrolytic instability [24]. Understanding and controlling hydrolytic stability of HCNs is therefore an important consideration prior to deployment in real‐world applications where water vapor is ubiquitous. To date, the response of HCNs to humidity has resulted in one of five outcomes (Figure 1, (i)–(v)). For (i) and (ii), water is physisorbed and the host framework is either rigid or undergoes a reversible structural transformation, respectively. **SIFSIX‐2‐Cu‐i** [24] (2 = 1,2‐bis(4‐pyridyl)acetylene, i = interpenetrated) exemplifies (i), whereas there are just a few examples of (ii): **NbOFFIVE‐1‐Ni** [25] (NbOFFIVE = NbOF_5_^2^^−^, 1 = pyrazine); **SIFSIX‐23‐Cu** [26] (23 = 1,4‐bis(1‐imidazolyl)benzene); **dia‐SNIFFOH‐1‐Cu** [27] (SNIFFOH = SnF_5_OH^2−^, **1** = 2,2′‐bipyrimidine). (iii)–(v) are possible outcomes of hydrolytic instability through chemisorption, whereby a water molecule coordinates as an aqua ligand to either M′ (iii) or M (iv, v). Coordination to M′ has thus far been observed only in trivalent metal pentafluorometallate pillars such as **AlFFIVE‐1‐Ni** and **FeFFIVE‐1‐Ni** (1 = pyrazine, AlFFIVE = AlF_5_^2−^, FeFFIVE = FeF_5_^2−^) [28], where coordination of an aqua ligand transforms the metal moiety from trigonal bipyramidal to octahedral geometry. This process is reversible with heating under reduced pressure (105°C, 3 mmHg) and does not change topology. Coordination at M proceeds through displacement of inorganic pillars by aqua ligands to form **sql** topology CNs, either irreversibly (iv) or reversibly (v). For example, **pcu‐SIFSIX‐1‐Cu**, which is isostructural to **SIFSIX‐1‐Zn**, irreversibly transforms to the catenated dihydrate sql‐c*‐SIFSIX‐1‐Cu at 40%–50% relative humidity (RH), and **pcu‐SIFSIX‐14‐Cu‐i** (14 = 1,2‐bis(4‐pyridy)diazene) similarly transforms to sql‐c*‐SIFSIX‐14‐Cu at 50%–60% RH [24]. Scenario (v) involves humidity‐induced switching between two (or more) structural states, with the hydrated phase(s) stabilized by hydrogen‐bonding networks. A notable example of (v) is the HUM **pcu‐SIFSIX‐3‐Ni** [29], which converts to non‐interpenetrated **sql‐SIFSIX‐3‐Ni** [30] at 50% RH. Unlike **SIFSIX‐1‐Cu** and **SIFSIX‐14‐Cu**, this transformation can be reversed upon heating to 160°C [24, 30]. Because sorbents exhibiting mechanisms (iii)–(v) can also sorb lattice water molecules, they can be described as combined chemi‐physisorption processes.

**FIGURE 1:**
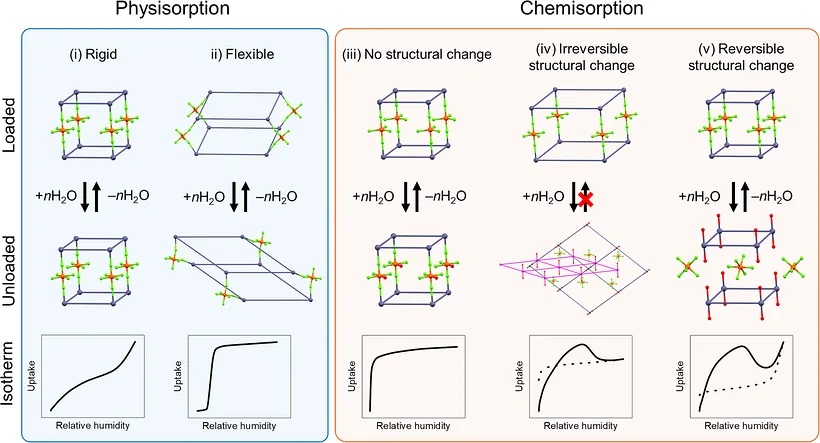
Five structural responses of hybrid coordination networks (HCNs) upon exposure to humidity, (i)–(v). Representative isotherm profiles (loading, solid lines; unloading, dotted lines) are presented underneath.

For consideration in AWH applications under arid conditions, desiccants should be regeneration optimized, that is adsorb water (load) at <30% RH and regenerate (unload) via mild heating (<60°C) [31]. To date, the top performing AWH desiccants are microporous physisorbents that load water through pore filling [32]. Accordingly, crystal engineering [33, 34] efforts to design AWH sorbents have largely focused on physisorbents since chemisorption typically requires high regeneration temperatures, sometimes >200°C [28]. Consequently, chemisorption has generally been considered incompatible with the combination of low‐RH water capture and low‐temperature regeneration required for AWH, making chemisorbents nonobvious candidates for regeneration‐optimized sorbents [35] (ROSs) for AWH. Indeed, we are unaware that any chemisorbent has yet been shown to meet the criteria to serve as a ROS for AWH. Herein, we address this matter by introducing **M'FSIX‐25‐M** (M = Co^2+^, Ni^2+^, Cu^2+^; M' = Si^4+^, Ti^4+^; **25** = 1,4‐bis(imidazol‐1‐yl)naphthalene, Scheme 1), a new family of HCNs, and comparing their water vapor sorption properties with **SIFSIX‐3‐Ni** and its analogs. Whereas both families were found to undergo reversible water uptake through cooperative chemisorption, only the former qualifies as a ROS. This makes **M'FSIX‐25‐M** the first regeneration optimized chemisorbents (ROCs).

## 2 Results and Discussion

Single crystals of four new **sql** topology networks with general formula [M(**25**)_2_(H_2_O)_2_](M'F_6_)(H_2_O)_n_, **sql‐M'FSIX‐25‐M** (M = Co^2+^, Ni^2+^; M' = Si^4+^, Ti^4+^) were synthesized by solvent layering (see ESI). A review of crystal structures containing this ligand deposited in the Cambridge Structural Database (CSD, V6.01, update Feb 2026, Figure S1) identified 12 structures (Table S1), none of which are HCNs. **25** is therefore a new ligand in the context of pillared HCNs (Figure S2). Single‐crystal X‐ray diffraction (SCXRD) analysis revealed that each variant had crystallized in the monoclinic space group *C*2/*c* (Tables S2–S4). The frameworks are comprised from **sql** nets that are stacked such that the intrinsic voids align to form 1D channels along [001] (Figures 2a and S4–S7; Table S5). The octahedral coordination sphere contains four equatorial **25** ligands and two axial aqua ligands that hydrogen bond to neighboring uncoordinated (extra‐framework) M'F_6_^2−^ anions. Synthesis with M = Cu^2+^ yielded 3D **pcu** topology HCNs, **pcu‐SIFSIX‐25‐Cu** and **pcu‐TIFSIX‐25‐Cu**, where coordinated anions pillar adjacent **sql** layers by coordinating at the axial positions of Cu cations (Figures S8 and S9). Interestingly, mild heating (60°C, dry N_2_) of single crystals of **sql‐TIFSIX‐25‐Ni**, **sql‐SIFSIX‐25‐Ni**, **sql‐TIFSIX‐25‐Co** and **sql‐SIFSIX‐25‐Co** led to displacement of the aqua ligands with M'F_6_^2−^ anions coordinated in a *cis*‐configuration [26] to yield **pcu** nets isostructural to **pcu‐SIFSIX‐25‐Cu** and **pcu‐TIFSIX‐25‐Cu** (Figures 2a and S10–S13; Tables S2–S4). Unexpectedly, this single‐crystal‐to‐single‐crystal **sql**‐to‐**pcu** transformation was found to be reversible for **TIFSIX‐25‐Ni**, **SIFSIX‐25‐Ni,** and **TIFSIX‐25‐Co** when left at ambient conditions (40% RH, 22°C). This reversible coordination of M'F_6_^2−^ anions is analogous to that reported for **SIFSIX‐3‐Ni** [29], where the SIFSIX pillar in **pcu‐SIFSIX‐3‐Ni** is displaced by aqua ligands to form **sql‐SIFSIX‐3‐Ni** [30], [Ni(**3**)_2_(H_2_O)_2_](SiF_6_)(H_2_O)_2_ (Figure 2b). As such, the well‐studied **M'FSIX‐3‐M** family provides a suitable benchmark for comparison to the new **M'FSIX‐25‐M** compounds. While a handful of studies have addressed hydrolytic stability of HCNs [20, 23, 24, 27, 30, 36, 37], to our knowledge none have systematically examined how variation of M and M' influences the reversibility of this process and the conditions under which it occurs. We therefore synthesized analogs of **M'FSIX‐3‐M** (M = Co^2+^, Ni^2+^, Zn^2+^; M' = Si^4+^, Ti^4+^, and Ge^4+^) to systematically probe how composition influences hydrolytic stability and regeneration conditions.

**FIGURE 2:**
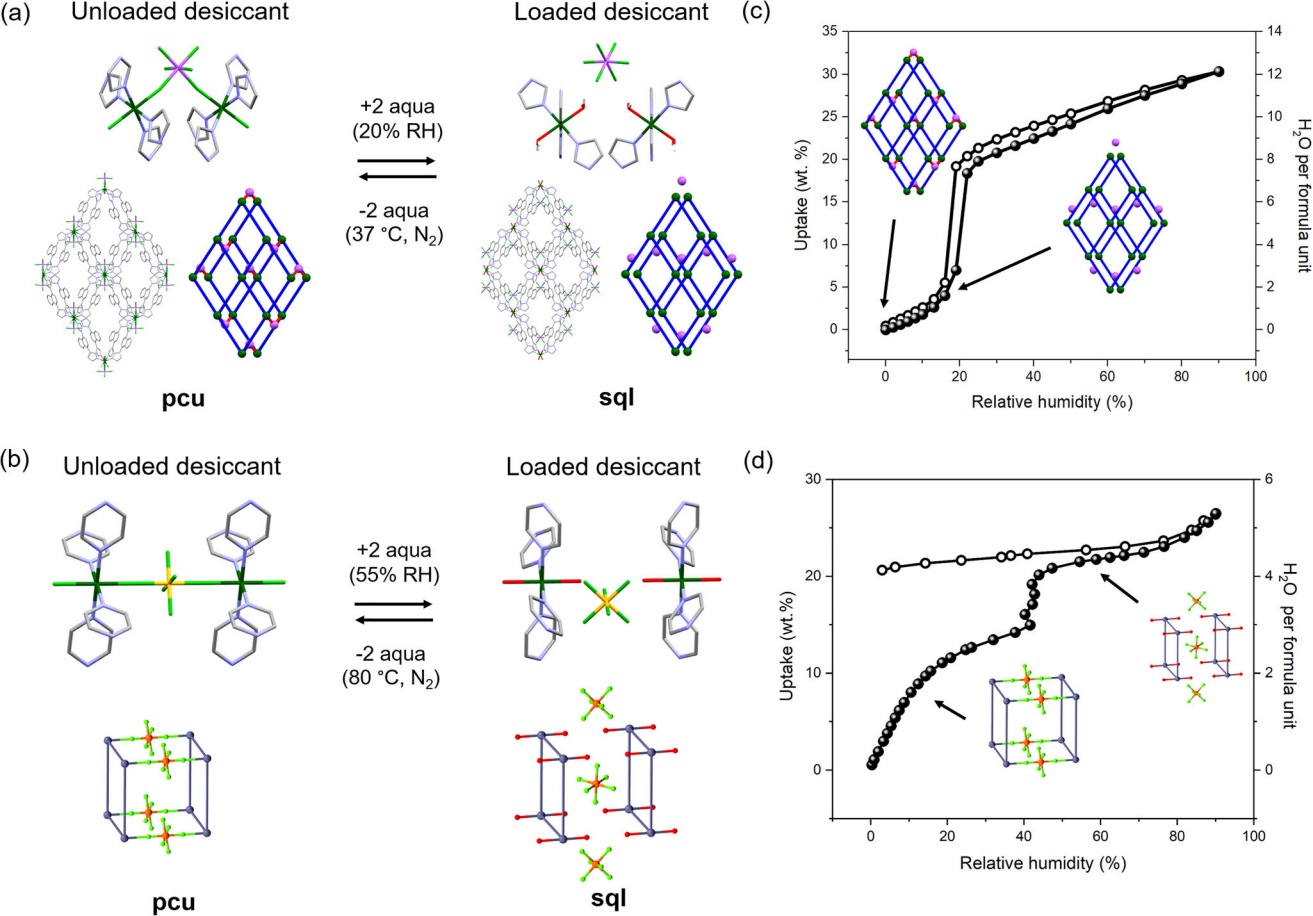
Reversible cooperative ligand insertion mechanism of water in (a) **TIFSIX‐25‐Ni** and (b) **SIFSIX‐3‐Ni**. Water vapor adsorption (closed circles) and desorption (open circles) isotherms for (c) **TIFSIX‐25‐Ni** and (d) **SIFSIX‐3‐Ni**. Atom colors: carbon, gray; nitrogen, blue; oxygen, red; fluorine, green; silicon, yellow; titanium, purple; nickel, dark green; **SIFSIX‐3‐Ni pcu**‐to‐**sql** colors (bottom left): oxygen, red; fluorine, green; nickel, gray; silicon, orange.

Bulk microcrystalline powders of **M'FSIX‐3‐M** and **M'FSIX‐25‐M** were synthesized as water loaded **sql** phases (see ESI) with phase purity established by powder x‐ray diffraction (PXRD, Figures S14–S17). Temperature‐induced desorption of **sql‐M'FSIX‐3‐M** and **sql‐M'FSIX‐25‐M** was studied by thermogravimetric analysis (TGA, ramp rate 1°C min^−1^ under dry N_2_ gas, Figures S18–S31). **sql‐SIFSIX‐3‐Zn** underwent two weight loss steps upon heating with onset temperatures, *T*_on_, of *T*_on_ = 30°C and 81°C, respectively (Figure S20). The former is probably due to surface water whereas the latter is consistent with concurrent loss of lattice water and two aqua ligands per formula unit (H_2_O/FU, Table S6). Variable‐temperature PXRD (VTPXRD) analysis revealed that the second step was concomitant with structural transformation to **pcu‐SIFSIX‐3‐Zn** [38] (Figure S32). The other **sql‐M'FSIX‐3‐M** variants also underwent **sql**‐to‐**pcu** conversions during heating with PXRD diffractograms (Figures S33 and S42) consistent with those calculated from reported crystal structures [20, 29, 39, 40, 41] and that determined from SCXRD analysis of **SIFSIX‐3‐Co** (Table S4). All exhibited a single weight‐loss step with a narrow range of *T*_on_ values: **TIFSIX‐3‐Ni** (78°C); **TIFSIX‐3‐Co** (78°C); **SIFSIX‐3‐Ni** (80°C); **SIFSIX‐3‐Co** (82°C); **GEFSIX‐3‐Ni** (85°C); **GEFSIX‐3‐Co** (95°C). The regeneration temperatures of **sql‐M'FSIX‐25‐M** were determined to be markedly lower, with *T*_on_ values as follows: **TIFSIX‐25‐Co** (34°C); **TIFSIX‐25‐Ni** (37°C); **SIFSIX‐25‐Cu** (40°C); **TIFSIX‐25‐Cu** (42°C); **SIFSIX‐25‐Co** (43°C); **SIFSIX‐25‐Ni** (44°C). VTPXRD experiments verified that heating bulk **sql‐TIFSIX‐25‐Ni** to 60°C induced transformation to **pcu‐TIFSIX‐25‐Ni**, as evidenced by characteristic peaks at 7.2° and 8.0° 2*θ* shifting to 6.9° and 8.5°, respectively (Figure S39). These shifts coincide with the calculated PXRD patterns of the corresponding SCXRD‐derived structures. Comparable peak shifts were also observed in the diffractograms of **SIFSIX‐25‐Ni**, **TIFSIX‐25‐Co,** and **SIFSIX‐25‐Co** (Figures S40–S42). **pcu‐SIFSIX‐25‐Cu** and **pcu‐TIFSIX‐25‐Cu** did not undergo PXRD changes during unloading (Figures S43 and S44). CO_2_ sorption analysis (−78°C) of **pcu‐M'FSIX‐25‐M** revealed that they are microporous (Figures S49–S60) with Brunauer–Emmett–Teller (BET) surface areas as follows: **pcu‐TIFSIX‐25‐Co** (234.2 ± 25 m^2^ g^−1^); **pcu‐SIFSIX‐25‐Ni** (541.9 ± 0.1 m^2^ g^−1^); **pcu‐TIFSIX‐25‐Ni** (575.9 ± 0.5 m^2^ g^−1^); **pcu‐SIFSIX‐25‐Cu** (516.8 ± 5.5 m^2^ g^−1^); **pcu‐TIFSIX‐25‐Cu** (559.3 ± 14.8 m^2^ g^−1^). We observed that **pcu‐SIFSIX‐25‐Co** was sensitive to activation conditions; one batch remained stable and yielded a BET surface area of 151.4 m^2^ g^−1^, whereas a second batch partially degraded during activation (Figures S60 and S63). The microporosity of **pcu‐M'FSIX‐3‐M** was confirmed by CO_2_ sorption data at 0°C and 25°C [42], yielding BET surface areas (Figures S67–S87) consistent with previously reported values (Table S7): **pcu‐SIFSIX‐3‐Ni** (267 m^2^ g^−1^), **pcu‐TIFSIX‐3‐Ni** (227 m^2^ g^−1^), **pcu‐SIFSIX‐3‐Zn** (208 m^2^ g^−1^), **pcu‐TIFSIX‐3‐Co** (230 m^2^ g^−1^), **pcu‐GEFSIX‐3‐Ni** (217m^2^ g^−1^), **pcu‐SIFSIX‐3‐Co** (221 m^2^ g^−1^) and **pcu‐GEFSIX‐3‐Co** (187 m^2^ g^−1^).

To establish the reversibility of the **pcu**‐to‐**sql** transformations, dynamic vapor sorption (DVS) data were collected at 27°C (see Figure 2 and S88–S13). **M'FSIX‐25‐M** samples were activated with mild heating under dry air. The DVS profile of **TIFSIX‐25‐Ni** revealed an S‐shaped isotherm with an inflection point at just below 20% RH, reaching a maximum uptake of 30.3 ± 1.3 wt. % at 90% RH (Figure 2c and S95). We attribute the gradual uptake of *ca*. 2.5 ± 0.3 wt.% by 13% RH to physisorption of H_2_O by **pcu‐TIFSIX‐25‐Ni** before concomitant conversion and water loading by **sql‐TIFSIX‐25‐Ni**. Analysis of the result of refinement of lattice water molecules in **sql‐TIFSIX‐25‐Ni** suggested 4–5 disordered lattice water molecules per FU from a crystal that had been exposed to ambient humidity (Table S4). The isotherm suggests further water loading up to 90% RH. When combined with the two aqua ligands, a weight gain of 15%–17.5% would be expected for the inflection and this is indeed the case. The desorption profile of **TIFSIX‐25‐Ni** has negligible hysteresis. A similar loading profile was observed for **SIFSIX‐25‐Ni** with a step at *ca*. 12% RH, maximum uptake of 35.6 ± 1.4 wt. % and modest hysteresis in the desorption trace (Figure S88 and S97).

**TIFSIX‐25‐Co** has a more gradual inflection point between 12–30% RH, reaching maximum uptake of 28.0 ± 0.1 wt.% along with little hysteresis in the desorption trace (Figures S89 and S96). **SIFSIX‐25‐Co** exhibits gradual uptake until 50% RH, with a slight step at 50% RH and a maximum uptake of 10 wt. % (Figure S90); however, this sorption profile could not be reproduced in subsequent measurements. **SIFSIX‐25‐Cu** and **TIFSIX‐25‐Cu** exhibit moderately hydrophilic **TIFSIX‐25‐Ni** Type I [43] loading profiles with slight inflections consistent with loading into a rigid HCN (Figures S91 and S92).

The water sorption profiles of **M'FSIX‐3‐M** (Figures S101–S135) were measured volumetrically following in situ activation under dynamic vacuum. **SIFSIX‐3‐Ni** exhibits initial uptake consistent with a Type I [43] profile, indicative of physisorption, up to a loading of 3 H_2_O/FU (14.9 wt.%, Figure 2d). A sharp inflection point was observed at 42% RH, leading to additional uptake of 1 H_2_O/FU (5.1 wt.%), followed by gradual surface loading at higher RH. A pronounced hysteresis loop was observed during the desorption RH branch, with sorbed water largely retained upon lowering of RH. Similar isotherm profiles were observed for **SIFSIX‐3‐Zn**, **GEFSIX‐3‐Ni**, **GEFSIX‐3‐Co**, **SIFSIX‐3‐Co**, and **TIFSIX‐3‐Ni**, each exhibiting uptake to 3 H_2_O/FU prior to the inflection point, followed by the uptake of an additional 1 H_2_O/FU and retention of 4 H_2_O/FU during desorption. In contrast, **TIFSIX‐3‐Co** underwent loading to 2 H_2_O/FU before the inflection point, 1 H_2_O/FU after the inflection point, with 3 H_2_O/FU retained during desorption. Variable‐humidity PXRD (VHPXRD) measurements (Figures S136–S142) were conducted to gain insight into the phase‐transformation behavior of **M'FSIX‐3‐M** during loading (adsorption). The diffractograms match **pcu‐M'FSIX‐3‐M** prior to the inflection point and **sql‐M'FSIX‐3‐M** after the inflection point, indicating that the inflection indeed corresponds to a **pcu**‐to‐**sql** phase transformation. The sharpness of the inflection point (Table S8) correlates with the extent of phase coexistence observed by VHPXRD, with relatively sharp steps for **SIFSIX‐3‐Ni** and **GEFSIX‐3‐Ni** and broader steps for **SIFSIX‐3‐Zn**, **GEFSIX‐3‐Co**, **TIFSIX‐3‐Ni**, and **SIFSIX‐3‐Co** consistent with coexistence of the **pcu** and **sql** phases over a wider RH range. **TIFSIX‐3‐Co** retained its structure throughout the entire loading range. These data are consistent with the reported crystal structures of the **pcu** and **sql** phases, with the **pcu** phases containing a maximum of three lattice H_2_O/FU and the **sql** phases containing 4 H_2_O/FU, (two aqua and two lattice). This explains the additional uptake observed after the inflection point. The lower pretransformation loading of 2 H_2_O/FU for **TIFSIX‐3‐Co** is reminiscent of the sorption mechanism reported for **NbOFFIVE‐1‐Ni**, in which pyrazine ring alignment precedes the adsorption step, but incoming water does not coordinate to the M atoms, leading to a maximum uptake of 3 H_2_O/FU [25]. It should be noted that attempts to reproduce the reported vacuum‐based gravimetric isotherm of **SIFSIX‐3‐Ni** matched the literature profile only up to the inflection point (Figure S116) [24]. At higher RH, the sample degraded, likely because of the harsher dynamic vapor‐flow and reduced‐pressure conditions used during measurement [44]. Similar behavior was observed under ambient‐pressure gravimetric conditions (Text S12.2). To assess intrinsic stability, **sql‐M′FSIX‐3‐M** samples were exposed to 95% RH at 1 atm for 6 days (Figure S150). Under these static conditions, the Co analogues deliquesced, whereas **SIFSIX‐3‐Ni**, **TIFSIX‐3‐Ni**, **SIFSIX‐3‐Zn,** and **GEFSIX‐3‐Ni** retained crystallinity, indicating stability to prolonged high RH exposure. The total unloading enthalpies ∆*h* provide a reasonable representation of the energetic requirements of desiccant loading/unloading during operation [45]. The values of ∆*h* were determined using differential scanning calorimetry (DSC, ramp rate 1°C min^−1^, Figures S151–S161; Table S9) analysis and were found to be reproducible (in kJ mol^−1^): **TIFSIX‐25‐Ni** (49 ± 1.3), **TIFSIX‐25‐Co** (42 ± 1.0), **SIFSIX‐25‐Ni** (38 ± 0.8), **SIFSIX‐25‐Co** (23 ± 1.1), **SIFSIX‐3‐Zn** (40 ± 0.1), **TIFSIX‐3‐Ni** (26 ± 1.1), **GEFSIX‐3‐Ni** (42 ± 2.1), **SIFSIX‐3‐Ni** (43 ± 1.0), **SIFSIX‐3‐Co** (51 ± 0.5), **TIFSIX‐3‐Co** (52 ± 1.0) and **GEFSIX‐3‐Co** (44 ± 0.2). These values are much lower than those reported for conventional desiccants such as zeolites 13X (69) and MgY (72) [46], desiccant HUMs **AlFFIVE‐1‐Ni** (63) and **FeFFIVE‐1‐Ni** (65) [28], and are within the range of AWH physisorbents (absolute values ranging from 36 to 75 kJ mol^−1^) [7, 47].

That **TIFSIX‐25‐Ni, TIFSIX‐25‐Co**, and **SIFSIX‐25‐Ni** exhibit S‐shaped isotherms with inflection points <30% RH and unload at <60°C suggests AWH potential and we designate these materials as **ROC‐1**, **ROC‐2,** and **ROC‐3**, respectively. **ROC‐1, ROC‐2,** and **ROC‐3** were benchmarked against several well‐studied ROSs [35], [Cu_2_(glutarate)_2_(bipy)] [48] (**ROS‐037**), [Zn(1,2,4‐triazolate)F]_n_ (**ROS‐039**) [49], [Al(OH)(1H‐pyrazole‐3,5‐dicarboxylate)]_n_ (**MOF‐303**) [45], [Al(OH)(fumarate)]_n_ (**Al‐OH‐fumarate**) [50, 51], [Al(OH)(benzene‐1,3‐dicarboxylate)]_n_ (**CAU‐10‐H**) [52] and **Syloid AL‐1** [53], an industrial desiccant. Powdered samples of comparable mass were used for all analyses (*ca*. 10 mg dry mass for RH‐swing experiments and *ca*. 5 mg dry mass for temperature‐swing experiments, each sieved to particle size <100 µm). We first conducted six consecutive RH‐swing DVS experiments at 27°C (Figure 3a and S162–S164) with RH‐swing cycles 1–3 conducted at 0–30% RH and cycles 4–6 at 0–60% RH. These conditions provide a useful basis for comparison with reported kinetic data. **ROC‐1** and **ROC‐2** retained their sorption performance during cycling, whereas **ROC‐3** exhibited progressive degradation and was not investigated further (Figure S163). Kinetics experiments revealed that **ROC‐1** and **ROC‐2** exhibit relatively short sorption times, reaching full loading within 32 and 62 min, and complete unloading within 40 and 60 min, respectively. Under practical operating conditions, unloading is typically driven by heating at constant or reduced pressure. Therefore, temperature‐swing (T‐swing, constant partial pressure) and temperature‐humidity‐swing (T‐RH‐swing) experiments can be used to provide information about the productivity of desiccants for AWH and dehumidification processes. Indeed, T‐RH‐swing (Figures S166–S169) and T‐swing DVS experiments (Figures 3b and S170) revealed rapid desorption, with **ROC‐1** and **ROC‐2** fully unloading within 7 and 10 min, respectively. **ROC‐1** exhibited adsorption kinetics comparable to those reported for the benchmark **dia‐SNIFFOH‐1‐Cu** [27] under T‐swing conditions, reaching an uptake of 12.9 wt.% within 16 min (Figure 3c). The deliverable capacity of **ROC‐2** is lower under T‐swing conditions owing to its residual water uptake at 5.4% RH (constant H_2_O pressure of 10.7 mbar, Figure S170), whereas **ROC‐1** retained a working capacity of 20 wt.% across the operating RH window. Importantly, both sorbents fully regenerated within 5 min, demonstrating that the cooperative chemisorption mechanism does not impede rapid water release at the macroscopic level. As heat transfer is an important consideration in the design of multicyclic water‐harvesting devices [54], the heat capacities (*C*_p_) and thermal conductivities (*k*) of **ROC‐1** and **ROC‐2** (dry) were also determined by modulated DSC at 30°C (see ESI). The resulting values were *C*_p_(**ROC‐1**) = 0.87 ± 0.15 J g^−1^°C^−1^; *C*_p_(**ROC‐2**) = 0.81 ± 0.08 J g^−1^°C^−1^; *k*(**ROC‐1**) = 0.25 ± 0.02 W m^−1^°C^−1^; and *k*(**ROC‐2**) = 0.38 ± 0.04 W m^−1^°C^−1^. Compared with other porous sorbents, **ROC‐1** and **ROC‐2** exhibit relatively low heat capacities and moderate thermal conductivities [55], which can facilitate rapid heating and cooling during T‐swing operation.

**FIGURE 3:**
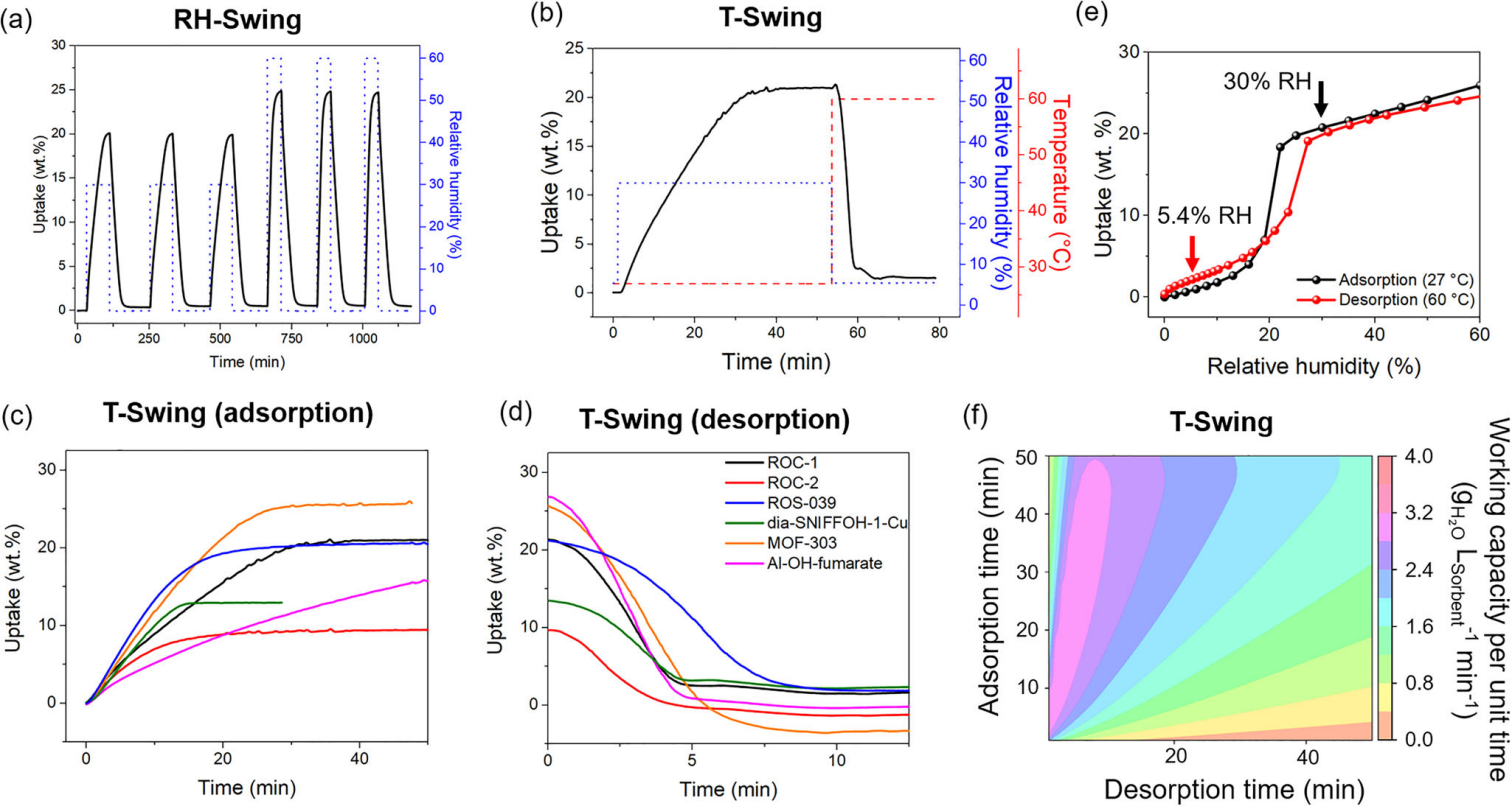
Water vapor (a) RH‐swing cycles (loading [30% or 60% RH] at 27°C) and (b) T‐swing (loading [30% RH, 27°C]—unloading [5.4% RH, 60°C], ca 5 mg) kinetics measurements on **ROC‐1**. T‐swing (c) adsorption and (d) desorption kinetic plots measured for **ROC‐1** and benchmark sorbents (ca 5 mg). (e) DVS isotherms of **ROC‐1** measured at 27 and 60°C, with arrows indicating position of the steps with respect to AWH operating conditions. (f) Water vapor sorption productivity “heatmap” modeled using diffusion to sorbent bed surface limitation model (*k* experimental, 10 mg sample) predicting sorption productivity for [30% RH, 27°C]—[5.4% RH, 60°C] temperature swing cycling. Performance is calculated per crystal volume of the desiccant (desolvated structure).

While full loading and unloading cycles provide the largest working capacity per ccycle, this is not optimal for AWH productivity (working capacity per unit time). Indeed, Bezrukov et al. demonstrated [35] that AWH productivity can be enhanced by non‐equilibrium cycling, that is, cycling between different partially loaded states to leverage the most rapid regions of desiccant loading/unloading. Using sorption data isotherm and kinetics data, productivity can be projected from “heatmaps” that indicate the optimal conditions for maximum productivity under a given set of conditions (see Text S20). These tests are designed to mimic processes such as desiccant wheels in HVAC systems and are therefore appropriate for the following reasons: such systems tend to operate under non‐equilibrium conditions with rapid loading–unloading cycles of ca. 3 min per cycle [56]; solid desiccants are typically formulated as fully‐exposed fine particles on surfaces, meaning that the intrinsic properties of particles translate well to a scaled‐up device [35, 57]; given these typical formulations, volumetric productivity is more relevant than gravimetric productivity [27, 35]. As **ROC‐1** exhibits a well‐defined working capacity across the T‐swing operating window (Figure 3e), its projected maximum water‐harvesting productivity was calculated to be 3.5 ${\mathrm{kg}}_{H_{2}O}$ L_sorbent_^−1^ d^−1^ (Figure 3f and S179–S191). While this productivity is lower than the top performing ROSs, **dia‐SNIFFOH‐1‐Cu** (9.5) [27], **ROS‐040** (8.5), **MIL‐160** (8.1), **ROS‐039** (7.8), **ROS‐037** (6.5), **MOF‐303** (6.5), and **CAU‐10‐H** (5.4), it outperforms the industrial desiccant **Syloid** (3.2) [35].

To assess cycling stability, **ROC‐1** and **ROC‐2** were subjected to 100 consecutive RH‐swing (0%–60% RH) and T‐swing (5.4%–30% RH) sorption cycles (Figures S191–S199). Under RH‐swing conditions, **ROC‐1** and **ROC‐2** exhibited capacity losses of 3.5 and 5.8 wt.%, respectively, while absolute losses of 12.9 and 8.5 wt.% were observed under T‐swing conditions. Both materials retained crystallinity after cycling, albeit with diminished diffraction intensity (Figure S193). Fitting and extrapolation of the RH‐swing cycling data to 500 cycles indicated that the capacities approach plateaus of 21.6 and 4.5 wt.% for **ROC‐1** and **ROC‐2**, respectively, corresponding to reductions of 5.8 and 9.6 wt.% relative to their initial capacities (Figures S196 and S197). SEM (Figure S202) and particle size distribution (Figure S203) analyses of pristine **ROC‐1** and **ROC‐2** and samples recovered after water sorption revealed that both materials retained their morphology following sorption. SEM images indicated reduced particle agglomeration after water sorption, resulting in more discrete crystallites. Mean particle sizes were observed to be 47.4 ± 2.0 µm before and 24.0 ± 1.8 µm after sorption for **ROC‐1**, and 25.8 ± 1.2 µm before and 26.4 ± 2.0 µm after sorption for **ROC‐2**. Overall, water sorption did not affect the particle morphology11.

A cooperative ligand insertion mechanism appears to drive the low‐temperature regeneration of the HCNs. To gain further insight into this mechanism, we conducted computational analyses of the unloading of the **M'FSIX‐25‐M** family, **SIFSIX‐3‐Ni** and **HKUST‐1** [58], a rigid MOF with unsaturated metal sites. The unloading process was separated into two steps with the energy *E* calculated for each phase (Figure 4; Tables S14 and S15): the initial loaded phase (α), initial loaded phase with water removed (α_1_), and that after structural transformation (β). The differences in energy ∆*E* were determined for select pairs: $\Delta E_{\alpha ↔\alpha_{I}}$ is the energy required for breaking the aqua‐ligand bond (endothermic), $\Delta E_{\alpha_{I}↔\beta }$ is the energy of the structural transformation (exothermic) and Δ*E*_α↔β_ is the total energy of unloading. **HKUST‐1** had the lowest $\Delta E_{\alpha ↔\alpha_{I}}$(53.7 kJ mol^−1^), with **SIFSIX‐25‐Co** (115.7), **ROC‐2** (127.4), **ROC‐3** (152.6), **SIFSIX‐3‐Ni** (154.8), and **ROC‐1** (158.1) all exceeding it. However, these relatively high values are offset by the energy released during the structural transformation yielding net unloading energies of **ROC‐2** (38.8), **SIFSIX‐25‐Co** (42.6), **HKUST‐1** (53.7), **ROC‐1** (54.0), **ROC‐3** (58.1), and **SIFSIX‐3‐Ni** (76.8). These values indicate that the unexpectedly facile removal of coordinated aqua ligands occurs due to energy compensation provided by the **sql**‐to‐**pcu** transformation (Figure 4). In the cases of **SIFSIX‐25‐Cu** and **TIFSIX‐25‐Cu**, which did not form **sql** phases under the conditions studied, hypothetical removal of coordinated water is associated with energies Δ*E*_α↔β_ = 40.7 and 46.5 kJ mol^−1^, respectively. We hypothesize that crystalline **sql** formation is unattainable for **M'FSIX‐25‐Cu** because of the low charge density of Cu^2+^ relative to Co^2+^ and Ni^2+^, as well as its propensity toward weakened axial bonds (i.e., aqua ligand bonds) due to the Jahn–Teller effect (Text S23). The low energy penalty of these phase transformations can be attributed to the relatively subtle structural changes between the **sql** and **pcu** phases: the **sql** sheets being preserved during unloading and being non‐interpenetrated. Moreover, the sheets undergo only minor shifts along [001] (0.07 and 1.0 Å for **TIFSIX‐3‐Ni** and **ROC‐1**, respectively), the transformation primarily involving local rearrangement at the metal node together with a modest displacement of the pillars. The relatively high calculated unloading energy determined for **SIFSIX‐3‐Ni** can be attributed to the diffusion pathway of water having less space and unfavorable steric interactionsin the denser **TIFSIX‐3‐Ni** structure than in **M'FSIX‐25‐M**s (Figure S211). The simulations also suggest that unloading of each coordinated water molecule occurs simultaneously and not in a step‐wise fashion, as evidenced by the higher energy penalty that occurs with asymmetric unloading (See Supporting Information).

**FIGURE 4:**
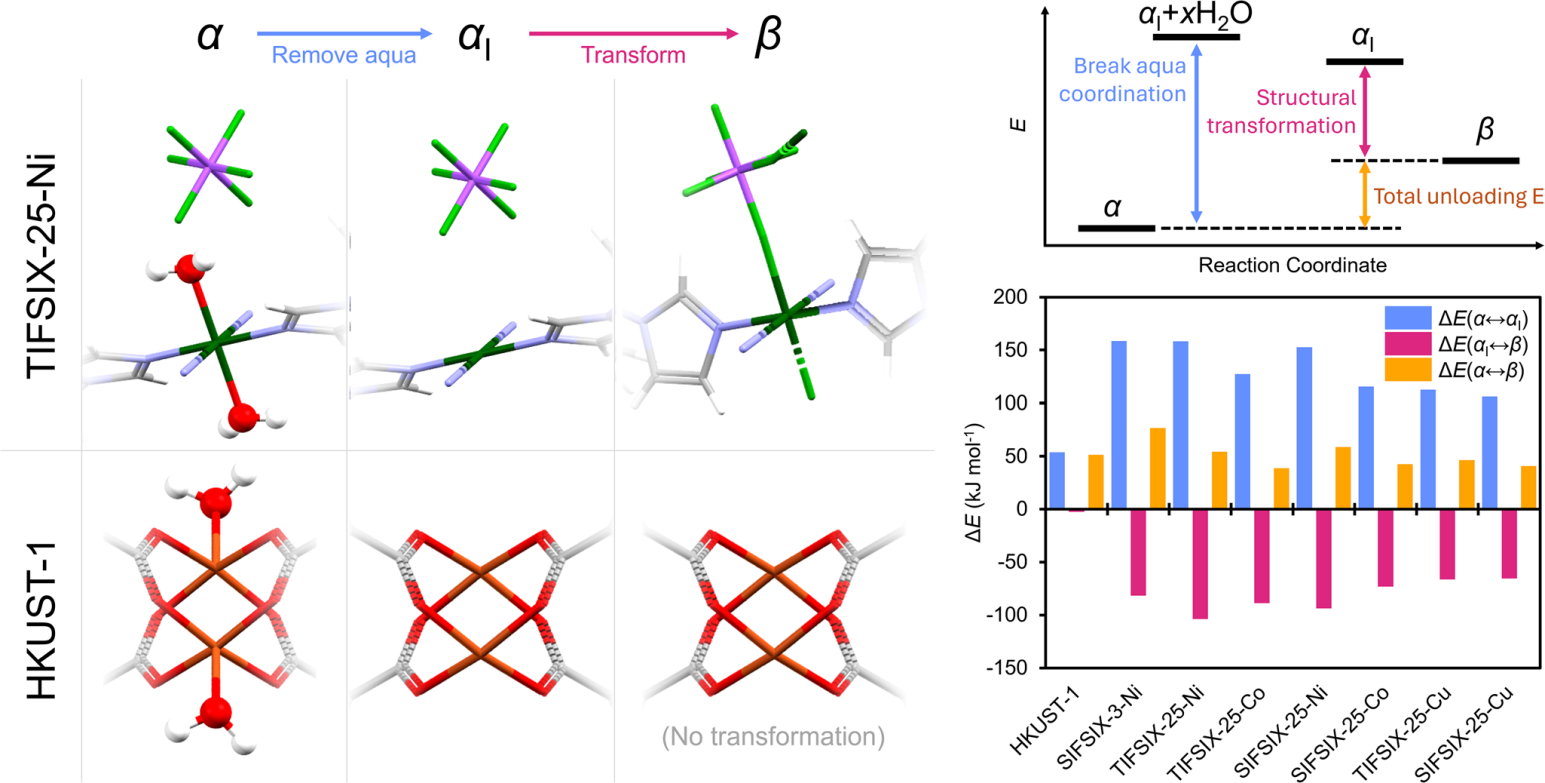
Computational analysis of the structural transformation pathway in **M'FSIX‐25‐M**, **SIFSIX‐3‐Ni** and **HKUST‐1**. (Left) Representative metal coordination environments in the *α* (loaded), *α*_I_ (intermediate, unloaded), and *β* (unloaded) phases, shown for **TIFSIX‐25‐Ni** (top) and **HKUST‐1** (bottom); **HKUST‐1** exhibits no *α*_I_ → *β* transformation upon unloading. (Top right) Schematic energy profile illustrating the two sequential steps: aqua ligand removal (Δ*E*(*α*↔*α*_I_), blue) and structural transformation (Δ*E*(*α*_I_↔*β*), pink), with the net unloading energy Δ*E*(*α*↔*β*) indicated in orange. (Bottom right) Single‐point energy differences for all investigated MOFs, showing that guest‐induced structural stabilization offsets the energetic cost of aqua ligand removal, enabling low regeneration temperatures.

**ROC‐1**, **ROC‐2**, and **ROC‐3** are, to our knowledge, the first chemisorbents to fall within the “sweet spot” for AWH (Figure 5, powder blue box) [32]. These materials can therefore serve as proof‐of‐concept sorbents for reversible chemisorption under AWH‐relevant conditions. Several trends emerged from analyzing the regeneration temperatures and conversion RHs. Compared with the M  =  Ni analogs in the **M'FSIX‐3‐M** family, the M  =  Co variants underwent **sql**‐to‐**pcu** phase transformation at higher regeneration temperatures and the reverse **pcu**‐to‐**sql** transformation at lower RH values (Figure S212).

**FIGURE 5:**
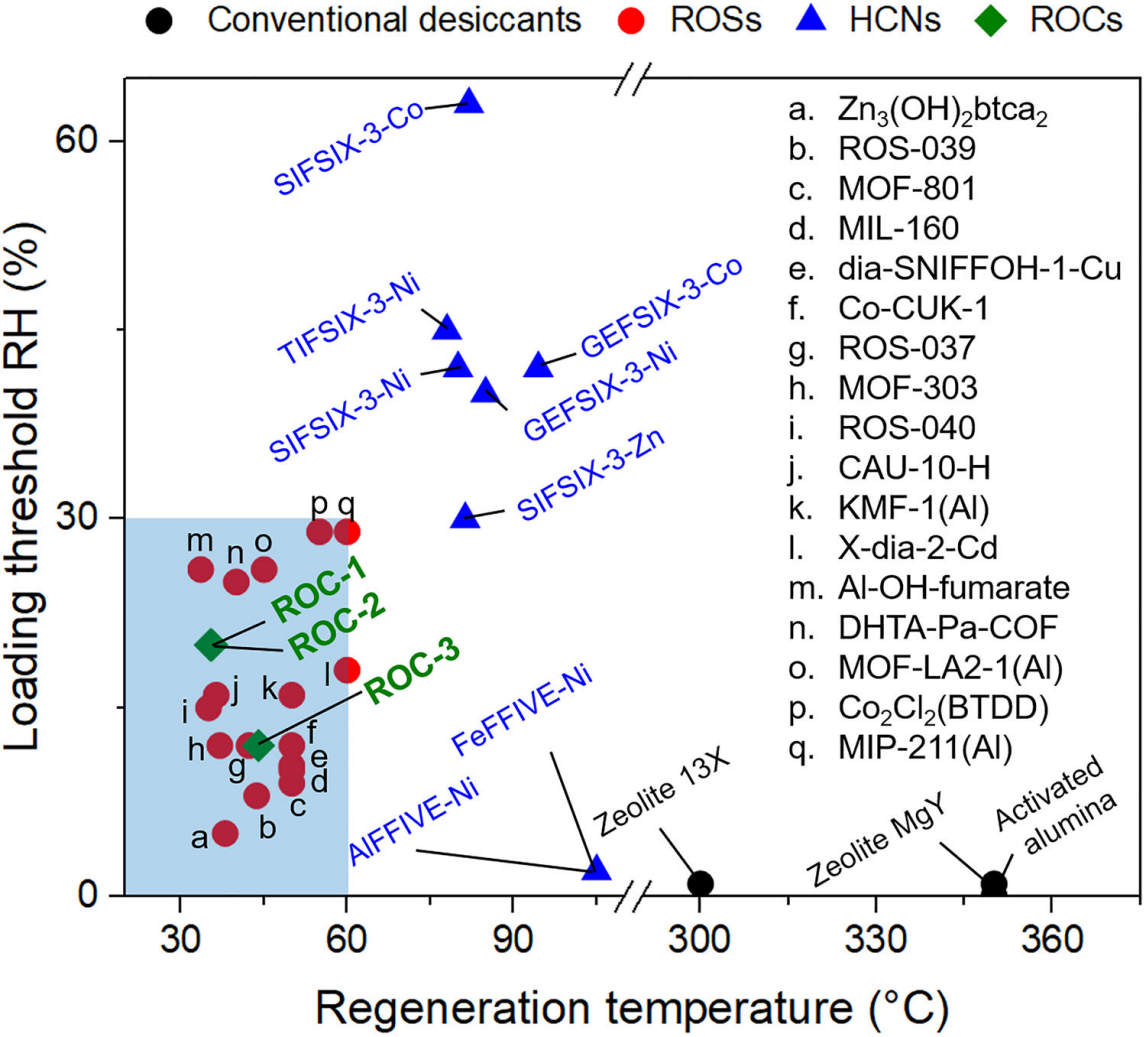
Scatter plot of reported loading threshold RH (sharp uptake or inflection point), determined from DVS analysis (room temperature), as a function of regeneration temperature [59] of conventional desiccants, ROSs, HCNs, and ROCs. The powder blue shaded region indicates the “sweet spot” of operating conditions relevant to AWH.

The order of regeneration temperatures by pillar was observed to be TIFSIX < SIFSIX < GEFSIX with no clear trend observed in the RH required for the reverse transformation. **M'FSIX‐25‐M** exhibited similar trends with respect to regeneration temperature, **TIFSIX‐25‐Ni** < **SIFSIX‐25‐Ni**, and **pcu**‐to‐**sql** transformation RH, **SIFSIX‐25‐Ni** < **TIFSIX‐25‐Ni**.

## 3 Conclusion

Chemisorption‐based desiccants have been largely overlooked as AWH desiccants due to their generally high regeneration temperatures. We have found, by fine‐tuning the coordination environment and pore structure of HCNs, that a new class of ROSs, namely ROCs, are capable of loading and unloading water vapor through a cooperative chemisorption mechanism within the sweet spot of conditions for AWH, low RH loading and low‐temperature regeneration. **ROC‐1** is therefore able to exhibit a water harvesting productivity of 3.5 ${\mathrm{kg}}_{H_{2}O}$ L_sorbent_^−1^ d^−1^. Overall, this work introduces a new class of AWH desiccants by counterintuitively revealing that hydrolytic instability can be turned into a good thing by enabling reversible cooperative chemisorption of water molecules as aqua ligands. Nevertheless, these prototypal ROCs need to be improved upon with respect to their recyclability and productivity in order to be considered for practical utility. Given the potentially broad scope of materials with higher water content in their water‐loaded phases, we are optimistic that the performance of **ROC‐1** can be improved upon.

## Author Contributions

**Xiang‐Jing Kong**: writing – original draft, investigation, formal analysis, writing – review and editing, methodology. **Asif Raza**: formal analysis, data curation, investigation, writing – original draft, writing – review and editing, methodology, validation. **Lilia Croitor**: formal analysis, data curation. **Andrey Bezrukov**: data curation, software, formal analysis, writing – review and editing. **Xia Li**: formal analysis, data curation. **Angela Shipman**: writing – review and editing, formal analysis, data curation, investigation, visualization. **William Morris**: investigation, formal analysis. **Carlos J. Solanilla‐salinas**: investigation, formal analysis, data curation. **Adam Hogan**: formal analysis, software, data curation, investigation. **Brian Space**: supervision, investigation, writing – review and editing, resources. **Volodymyr Bon**: investigation, formal analysis, data curation. **Stefan Kaskel**: investigation, supervision, resources. **Soumya Mukherjee**: writing – review and editing, supervision, resources, funding acquisition. **Alan C. Eaby**: writing – review and editing, supervision, formal analysis, project administration, investigation, data curation, conceptualization, writing – original draft. **Tao He**: writing – original draft, writing – review and editing, formal analysis, supervision, conceptualization, project administration, data curation, investigation, resources. **Michael J. Zaworotko**: conceptualization, funding acquisition, project administration, supervision, writing – review and editing, resources.

## Conflicts of Interest

The authors declare no conflicts of interest.

## Supporting information



CCDC 2535708, 2535739–2535749 contain the supplementary crystallographic data for this paper. These data can be obtained free of charge via www.ccdc.cam.ac.uk/data_request/cif, or by emailing data_request@ccdc.cam.ac.uk, or by contacting The Cambridge Crystallographic Data Centre, 12 Union Road, Cambridge CB2 1EZ, UK; fax: +44 1223 336033.**Supporting File**: anie74915‐sup‐0001‐SuppMat.pdf.

## Data Availability

The data that support the findings of this study are available from the corresponding author upon reasonable request.
