# Supplementary material for: Regeneration‐Optimized Chemisorbents: Evaluating Hybrid Coordination Networks for Atmospheric Water Harvesting
Source: Angew Chem Int Ed Engl. 2026 Sep 21;65(45):e6095254. doi: 10.1002/anie.6095254 (PMC13725610; doi:10.1002/anie.6095254)
Supplement: Supplementary file 1 [file ANIE-65-e6095254-s001.pdf]

Regeneration-Optimized Chemisorbents: Evaluating Hybrid Coordination Networks for Atmospheric Water Harvesting

Xiang-Jing Kong,+, [a], [b] Asif Raza, +,[a] Lilia Croitor,[a] Andrey Bezrukov,[a] Xia Li,[a] Angela Shipman,[c] William Morris,[c] Carlos J. Solanilla-Salinas,[c] Adam Hogan,[c] Brian Space,[c] Volodymyr Bon,[d] Stefan Kaskel,[d] Soumya Mukherjee,[a] Alan C. Eaby,*,[a] Tao He,*,[a], [b] Michael J. Zaworotko*,[a]

[a] Dr Xiang-Jing Kong, Dr Asif Raza, Dr Lilia Croitor, Dr Andrey Bezrukov, Prof. Soumya Mukherjee, Dr Alan C. Eaby, Prof. Tao He, Prof. Michael J. Zaworotko
Bernal Institute and Department of Chemical Sciences
University of Limerick
Limerick V94 T9PX, Ireland
E-mail: alan.eaby@ul.ie; xtal@ul.ie

[b] Dr Xiang-Jing Kong, Prof. Tao He
Department of Chemical Engineering, College of Materials Science and Engineering
Beijing University of Technology
Beijing 100124, China
Email: hetao@bjut.edu.cn

[c] Dr Angela Shipman, Mr William Morris, Dr Carlos J. Solanilla-Salinas, Dr Adam Hogan, Prof. Brian Space
Department of Chemistry
North Carolina State University
Raleigh, NC 27607, USA.

[d] Dr Volodymyr Bon, Prof. Stefan Kaskel
Department of Inorganic Chemistry
Technische Universitat Dresden

Table of contents

1 Materials and Methods 4
1.1 Materials 4
1.2 Single Crystal X-ray diffraction 4
1.3 Crystallographic Software 4
1.4 Powder X-ray diffraction 5
1.5 Variable-Temperature Powder X-ray diffraction 5
1.6 Variable Humidity Powder X-ray Diffraction (VH-PXRD) 5
1.7 Thermal analysis 7
1.8 Gas sorption 7
1.9 Particle size and morphology analysis 7
1.10 Dynamic Vapor Sorption 8

1.10.1 Water Vapor Sorption Isotherms ... 8
1.10.2 Water Vapor Sorption Kinetic Analysis ... 10
1.10.3 Water Vapor Sorption Hydrolytic Stability (cycling) ... 10
2 CSD survey ... 11
3 Nomenclature ... 12
4 Ligand Synthesis ... 14
5 Material Synthesis ... 15
5.1 Synthesis of **M'FSIX-3-M** compounds ... 15
5.1.1 Synthesis of $\{[Ni(pyrazine)_2(SiF_6)]\}_n$ (**pcu-SIFSIX-3-Ni**) ... 15
5.1.2 Synthesis of $\{[Ni(pyrazine)_2(GeF_6)]\}_n$ (**pcu-GEFSIX-3-Ni**). ... 15
5.1.3 Synthesis of $\{[Co(pyrazine)_2(SiF_6)]\}_n$ (**SIFSIX-3-Co**) ... 15
5.1.4 Synthesis of $\{[Ni(pyrazine)_2(TiF_6)]\}_n$ (**pcu-TIFSIX-3-Ni**). ... 15
5.1.5 Synthesis of $\{[Co(pyrazine)_2(TiF_6)]\}_n$ (**pcu-TIFSIX-3-Co**). ... 16
5.1.6 Synthesis of $\{[Zn(pyrazine)_2(SiF_6)]\}_n$ (**pcu-SIFSIX-3-Zn**). ... 16
5.1.7 Synthesis of $\{[Co(pyrazine)_2(GeF_6)]\}_n$ (**pcu-GEFSIX-3-Co**). ... 16
5.2 Synthesis of **M'FSIX-25-M** compounds ... 16
5.2.1 Synthesis of **SIFSIX-25-Co**. ... 16
5.2.2 Synthesis of **SIFSIX-25-Ni**. ... 17
5.2.3 Synthesis of **SIFSIX-25-Cu**. ... 17
5.2.4 Synthesis of **TIFSIX-25-Co**. ... 17
5.2.5 Synthesis of **TIFSIX-25-Ni**. ... 17
5.2.6 Synthesis of **TIFSIX-25-Cu**. ... 17
5.2.7 Bulk synthesis of **M'FSIX-25-M** compounds ... 18
6 Crystallographic tables and analysis ... 19
6.1 Crystal structures of **sql-M'FSIX-25-M** ... 22
6.2 Crystal structures of **pcu-M'FSIX-25-M** ... 24
7 Powder X-ray diffraction ... 29
8 Thermogravimetric analysis ... 33
9 Variable-temperature powder X-ray diffraction ... 44
10 Comparison of loaded and unloaded PXRDs ... 53
11 Gas sorption ... 56
11.1 M'FSIX-25-M and Post-Adsorption PXRD ... 56
11.2 M'FSIX-3-M ... 65
12 Water vapor sorption ... 77
12.1 **M'FSIX-25-M** and Post-Adsorption PXRD ... 77
12.2 **M'FSIX-3-M** ... 84
12.2.1 Volumetric vacuum water sorption ... 84
12.2.2 Gravimetric vacuum water sorption ... 85

12.2.3 Gravimetric ambient-pressure water sorption 87
13 Variable-humidity powder X-ray diffraction 106
14 Powder X-ray diffraction after DVS measurement 113
15 Estimation of Adsorption Enthalpy using Differential Scanning Calorimetry 121
16 Humidity-swing kinetics 128
17 Temperature-humidity-swing kinetics 130
18 Temperature-swing kinetics 132
19 Estimation of the thermal conductivities of **ROC-1** and **ROC-2** by modulated DSC analysis 136
19.1 Theory 136
19.2 Experimental details 137
19.3 Calibration and thermal conductivity determination 138
20 Water harvesting productivity heatmaps 141
20.1 Sorption kinetics analysis 142
21 Humidity-swing cycling 153
22 Particle size and morphology 159
23 Computational modelling 161
23.1 Unit Cell Preparation 161
23.2 Periodic Density Functional Theory (DFT) Optimizations 161
23.3 Pore Analyzer for Measuring PLD 161
23.4 Design of Computational Procedures 162
23.4.1 Phase Transition Energy Calculations 162
23.4.2 TGA and Pore Limiting Diameter for Kinetics 165
23.5 Validation from Experiment 167
23.6 Results: Thermodynamic Handles 167
23.7 Results: Kinetic Handles 167
23.8 Discussion of Computational Results 171
23.8.1 The Transformation Mechanism is Concerted 171
23.8.2 Theoretical **sql-M’FSIX-25-Cu** Structures 172
23.8.3 Energetic Trade and Kinetically-Favorable **25** Linker Allow **TIFSIX-25-Ni** to Reversibly Transform at Low Temperatures 172
24 References 178

1 Materials and Methods

1.1 Materials

Starting materials, reagents and solvents were purchased from commercial sources (Sigma-Aldrich, FluoroChem, Fisher Scientific, Lennox, and TCI Europe N.V.) and used without further purification.

1.2 Single Crystal X-ray diffraction

Single crystal X-ray diffraction data of all the crystals were collected on a Bruker Quest diffractometer equipped with a IμS microfocus X-ray source (Cu Kα, λ = 1.54178 Å; Mo Kα, (λ = 0.71073 Å) and CMOS detector. APEX4 was used for collecting, indexing, integrating and scaling the data. Open-flow nitrogen attachment with Oxford Cryosystem was used for low temperature measurements. Absorption correction was performed by multi-scan method.[1] Space groups were determined using XPREP as implemented in APEX4.[2] The datasets were corrected by empirical absorption correction using spherical harmonics, implemented in the SCALE3 ABSPACK scaling algorithm.[3] All the structures were solved using direct methods and refined by full-matrix least-squares on F^2 with anisotropic displacement using the SHELXTL software package.[4] Non-hydrogen atoms on frameworks were refined with anisotropic displacement parameters during the final cycles. The hydrogen atoms on frameworks were positioned geometrically or added according to theoretical models, and assigned isotropic displacement parameters to ride on their respective parent atoms. Some N, C, and O atoms in the structure of MOFs were disordered and treated by occupancy refinements. The volume fractions of disordered solvents in pores could not be modeled in terms of atomic sites, but were treated by using the MASK routine in the Olex2 software package.[5] Crystallographic data for all the HCNs reported in this paper are summarized in Tables S1-S3. Crystal structures are deposited to the Cambridge Crystallographic Data Centre (CCDC 2535708, 2535739-2535749).

1.3 Crystallographic Software

The Cambridge Structural Database[6] (CSD, v.6.01, February 2026) was accessed using ConQuest.[7] Pore volumes and geometries (pore limiting diameters and maximum pore diameters) were calculated using the Pore Analyser (default settings) feature in Mercury[8] (2025.3.3, Build 472890). Crystal structure models and void volumes were visualized using Mercury[8] unless otherwise specified.

1.4 Powder X-ray diffraction

Diffractograms were recorded using a PANalytical Empyrean™ diffractometer equipped with a PIXcel3D detector operating in scanning line detector mode with an active length of 4 utilizing 255 channels. The diffractometer is outfitted with an Empyrean Cu LFF (long fine-focus) HR (9430 033 7310x) tube operated at 40 kV and 40 mA and CuKα radiation (λα = 1.540598 Å) was used for diffraction experiments. Continuous scanning mode with the goniometer in the theta-theta orientation was used to collect the data. Incident beam optics included the Fixed Divergences slit with anti-scatter slit PreFIX module, with a 1/8° divergence slit and a 1/4° anti-scatter slit, as well as a 10 mm fixed incident beam mask and a Soller slit (0.04 rad). Divergent beam optics included a P7.5 anti-scatter slit, a Soller slit (0.04 rad) and a Ni-β filter. In a typical experiment, 25 mg of sample was dried, ground into a fine powder and was loaded on a zero background silicon disks. The data was collected from 3°−40° (2θ) with a step-size of 0.0131303° and a scan time of 30 seconds per step. Crude data were analyzed using the X'Pert HighScore Plus™ software V 4.1 (PANalytical, The Netherlands).

1.5 Variable-Temperature Powder X-ray diffraction

Diffractograms at different temperature were recorded using a PANalytical X'Pert Pro-MPD diffractometer equipped with a PIXcel3D detector operating in scanning line detector mode with an active length of 4 utilizing 255 channels. Anton Paar TTK 450 stage coupled with the Anton Paar TCU 110 Temperature Control Unit was used to record the variable temperature diffractograms. The diffractometer is outfitted with an Empyrean Cu LFF (long fine-focus) HR (9430 033 7300x) tube operated at 40 S5 kV and 40 mA and CuKα radiation (λα = 1.54056 Å) was used for diffraction experiments. Continuous scanning mode with the goniometer in the theta-theta orientation was used to collect the data. Incident beam optics included the Fixed Divergences slit, with a 1/2° divergence slit and a Soller slit (0.04 rad). Divergent beam optics included a P7.5 anti-scatter slit, a Soller slit (0.04 rad), and a Ni-β filter. In a typical experiment, 20 mg of sample was dried and loaded on a zero background sample holder made for Anton Paar TTK 450 chamber. The data were collected from 4°−40° (2θ) with a step-size of 0.0167113° and a scan time of 50 seconds per step. Crude data were analyzed using the X'Pert HighScore Plus™ software V 4.1 (PANalytical, The Netherlands).

1.6 Variable Humidity Powder X-ray Diffraction (VH-PXRD)

Diffractograms at different relative humidity (RH) for **M'FSIX-3-M** compounds, were collected using customized Empyrean (Malvern Panalytical GmbH) powder X-ray diffractometer (ω–2θ

goniometer, Alpha1 system), equipped with ARS DE-102 closed-cycle helium cryostat (cryostat was not used in this experiment). Customized X-ray transparent adsorption cell is based on 1.33″ CF-flange, one part of which is mounted on the cryostat and another is brazed to a Kapton dome. The cell was connected to the low-pressure port of the BELSORPmax (Mictrotrac MRB) volumetric adsorption instrument using 1/8″ stainless steel capillary. Water vapor was dosed to the system using a flask attached to the manifold of BELSORPmax instrument. The temperature of the flask was maintained at 300 K by water bath connected to a chiller. The diffraction experiments were conducted in a transmission geometry (ω–2θ scan mode) in the range of 2θ = 5 - 40°. Parallel beam optic involving hybrid 2xGe(220) monochromator, primary divergence slits (1/4°) and motorized anti-scatter slits (1/4°), 4 mm mask, Solar slits (0.04°) on the incident and diffracted beam optics. Pixcel-3D detector in 1D scanning mode (255 active channels) was used for counting the signal. PXRD patterns were in situ recorded after equilibration (0.1% of pressure drop within 30 min; monitored via Belmaster software) at selected points of humidity which were calculated via vapor pressure. Antoine equation was used to calculate the vapor pressure.

$$\log_{10} P_{sat} = A - \frac{B}{C + T}$$

Where P is the vapor pressure, T is temperature, *A*, *B* and *C* are component-specific constants. In our experiment we used (A = 8.07131, B = 1730.63 and C = 233.426).

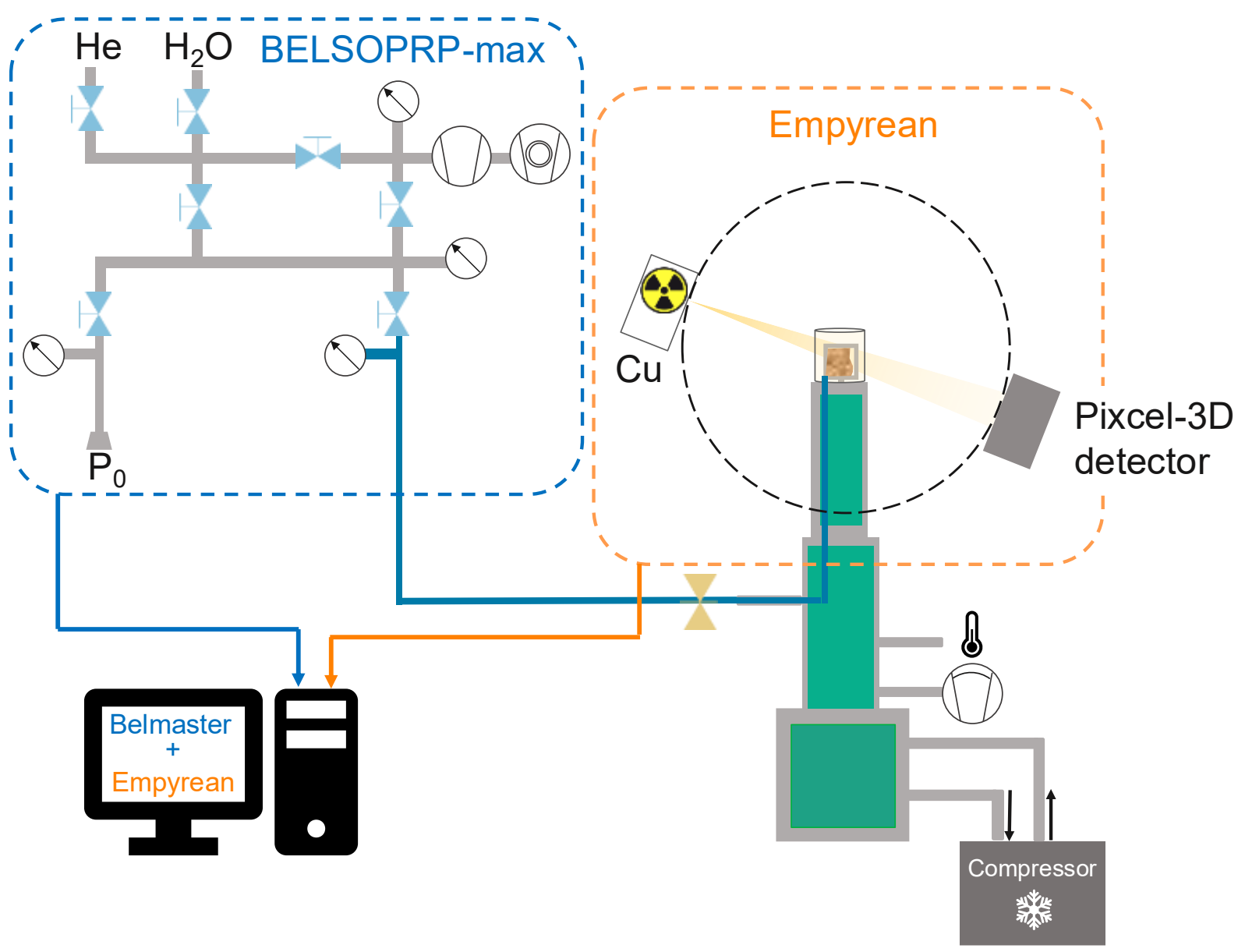

Scheme S1 Schematic of the in-situ variable humidity setup used in this study. The BELSORP-max instrument (in blue box) was used to dose water vapor to the sample, and pressure-drop was monitored on Belmaster software screen. Once equilibration was achieved Empyrean PXRD instrument (in orange box) was used to collect the diffractogram.

1.7 Thermal analysis

Thermogravimetric Analysis (TGA): Thermograms were recorded under nitrogen using TGA instrument TA Q50 V20.13 Build 39. Aluminum pans and a flow rate of 60 cm^3 min^{-1} for the nitrogen gas were used for the experiments. Initial data was collected in the High Resolution Dynamic mode with a sensitivity of 1.0, a resolution of 4.0 and a Temperature ramp rates of 1 and 10 °C min^{-1} were used The data was evaluated using the T.A. Universal Analysis suite for Windows XP/Vista Version 4.5A.

Differential scanning calorimetry (DSC): Enthalpies associated with unloading i.e.; loss of aqua ligands and lattice water were determined using temperature-ramp DSC analysis. Thermograms were recorded on a Q2000 TA Instruments system with a Refrigerated Cooling System (RCS90). During experiments, the sample compartment was purged using nitrogen gas (50 cm^3 min^{-1}).

1.8 Gas sorption

For gas sorption experiments, ultrahigh-purity gases were used as received from BOC Gases Ireland: research-grade He (99.999%), CO_2 (99.995%), and N_2 (99.998%). Adsorption experiments (up to 1 bar) for 77 K N_2 were performed on Micromeritics 3Flex surface area and pore size analyser 3500. Before sorption measurements, activation of all HCNs was achieved by degassing the air-dried samples filtered from methanol on a SmartVacPrep™ using dynamic vacuum and heating for 6 h (each sample heated from RT to 373 K with a ramp rate of 10 °C). Brunauer-Emmett-Teller (BET) surface areas were determined from the N_2 adsorption isotherms at 77 K respectively, using the Micromeritics Microactive software. About 50 mg of activated samples were used for the measurements. The low temperature of 77 K was controlled by a 4 L Dewar filled with liquid N_2.

1.9 Particle size and morphology analysis

Scanning electron microscopy measurements were carried out for the as synthesized and post water adsorption samples of **TIFSIX-25-Co** and **TIFSIX-25-Ni** to study morphology particle size uniformity. The images were captured on a Hitachi SU-70 instrument, using 5-10 kV acceleration voltage and a working distance of 15 mm. Before the measurement, the samples were dispersed on carbon tape attached to SEM stubs,and were gold-coated for 50 seconds to enhance surface conductivity.

Furthermore, bulk particle size distributions were determined using a Morphologi G3 automated imaging system (Malvern Panalytical). Measurements were performed the as synthesized and post water adsorption samples of **TIFSIX-25-Co** and **TIFSIX-25-Ni**. Prior to analysis, powders

were gently dispersed onto glass slides using a controlled dry powder disperser to minimise agglomeration. Images of individual particles were captured under bright-field illumination, and size distributions were generated based on equivalent circular diameter (ECD). Gaussian fitting of the distributions was carried out using the Origin software package. Reported size ranges may include both primary crystallites and aggregates.

1.10 Dynamic Vapor Sorption

1.10.1 Water Vapor Sorption Isotherms

Water vapor sorption measurements were carried out on **M'FSIX-25-M** desiccants using a Adventure dynamic vapor sorption (DVS) instrument manufactured by Surface Measurement Systems. The instrument gravimetrically measures water vapor uptake using air as a carrier gas. Digital mass flow controllers regulate flows of dry and saturated gases. The saturated flow is created by passing dry air through a water bubbler. Relative humidity is generated by precisely mixing dry and saturated gas flows in desired, calibrated flow ratios which produce expected relative humidity. Pure water was used to generate water vapor for these measurements and temperature was maintained at 300 K or at 333 K by enclosing the system in a temperature-controlled incubator. The mass of the sample was determined by a high resolution microbalance Ultrabalance Low Mass with a precision of 0.01 µg. The microbalance has a symmetric configuration with both the sample pan and reference pan being exposed to the same gas and being kept at the same temperature, allowing negation of buoyancy and drag effects. The instrument is equipped with two such balances, allowing measurement of two samples in parallel. Prior to the measurement, each sample was activated in-situ in dry air at 100 °C for 60 minutes using the built-in preheater and consequently cooled to sorption temperature in 90 minutes. Isotherm measurements were performed on approximately 10 mg of sample powder. 400 sccm min^{-1} total flow was used for the measurements at 300 K and 50 sccm min^{-1} total flow was used for measurements at 333 K. We found the lower flow was necessary to achieve a stable relative humidity reading at higher temperatures, especially at higher humidity. The flow is split between two samples, thus the flow experienced by the sample was half of the total flow. For each isotherm point, $dm/dt < 0.05$ % min^{-1} for a minimum of 10 minutes was used as criteria of reaching equilibrium. Kinetic measurements were performed with a 400 sccm min^{-1} flow, which is split between two samples. For adsorption and desorption kinetic curves, $dm/dt < 0.05$ % min^{-1} was used as criteria of reaching equilibrium.

Water vapor sorption analysis was carried out on the **M'FSIX-3-M** family using three different techniques methods: the Adventure DVS, detailed above, Vacuum DVS gravimetric and Belsorp Max volumetric sorption analyzers.

Vacuum DVS: Water vapor sorption measurements were carried out on **M'FSIX-3-M** desiccants using a Surface Measurement Systems DVS Vacuum at 298 K (Scheme S2). Samples were activated under high vacuum ($2x10^{-6}$ Torr) in-situ and stepped increases in relative humidity were controlled by equilibrated weight changes of the sample (dM/dT = 0.006 %/min) from 0 to 90% relative humidity. Vacuum pressure transducers were used with ability to measure from $1x10^{-6}$ to 760 Torr with a resolution of 0.01%. Approximately 10 mg of sample was used for each experiment. The mass of the sample was determined by comparison to an empty reference pan and recorded by a high-resolution microbalance with a precision of 0.1 µg.

Belsorp Max: Samples were activated *ex-situ* under dynamic vacuum for 24 h using a **BELSORP VacPrep**. Water vapor adsorption isotherms were collected at 298 K using a **BELSORP-max** (Microtrac BEL, Japan). Deionized water was used as the adsorptive and was degassed by three freeze–pump–thaw cycles using liquid nitrogen prior to measurement. The sample temperature was controlled using a water bath connected to a recirculating chiller. Equilibrium at each pressure step was defined as a pressure tolerance within 0.1% of P/P_0 over 450 s.

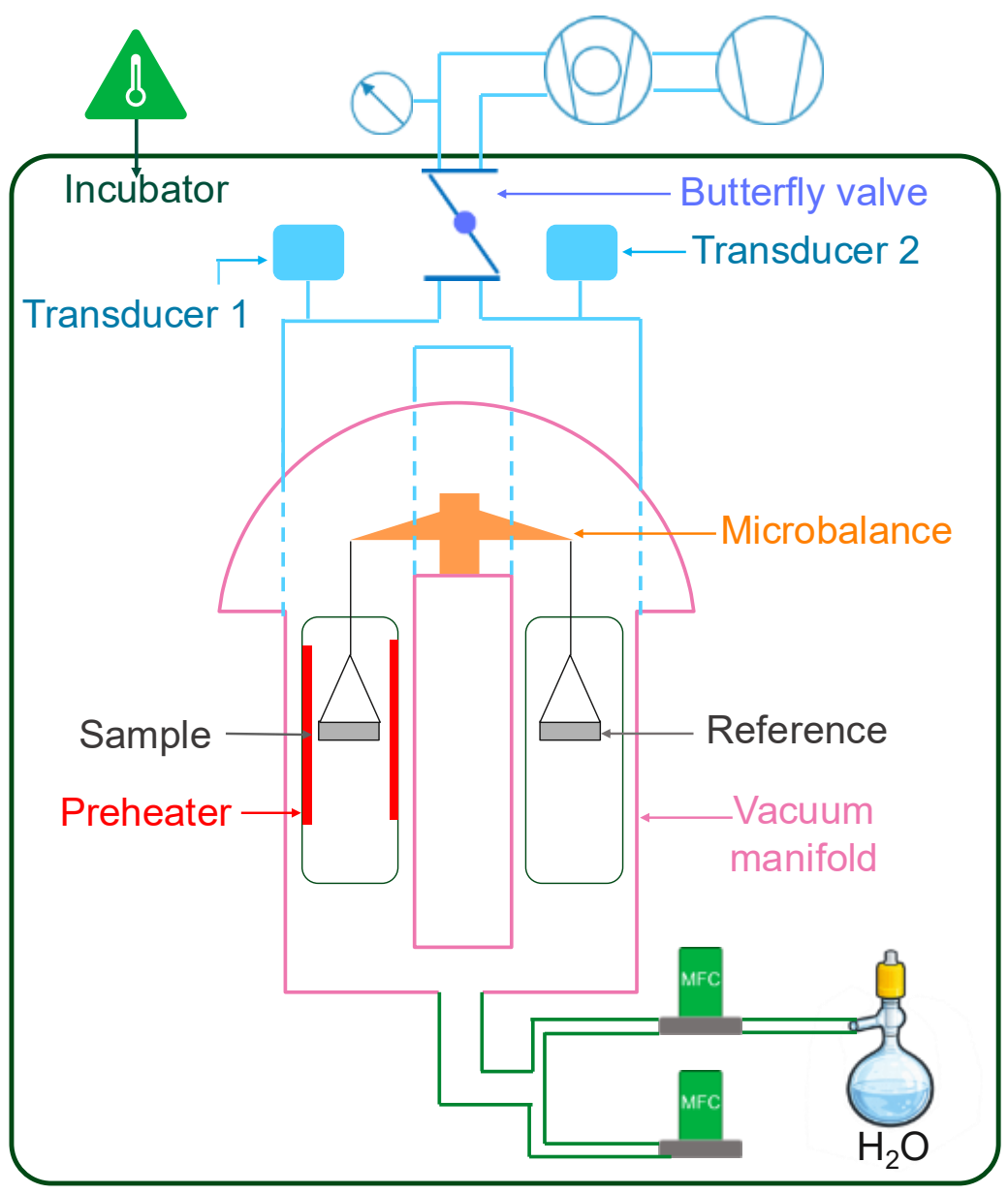

Scheme S2: Schematic of the vacuum dynamic vapor sorption instrument used in this study. The manifold is brought to vacuum and relative humidity is increased through controlled inflow of water from a reservoir. The mass increase of the sample is calibrated with a reference pan. The entire system is kept within an incubator to maintain a constant temperature.

1.10.2 Water Vapor Sorption Kinetic Analysis

Humidity-swing, temperature-swing and temperature-humidity-swing experiments were carried out on samples with particle size <100 μm with dm/dt ≤ 0.05 %·min^{-1} used as the equilibrium criteria. Dry mass of T-swing measurements were taken from equilibrium mass at 5.4% RH.

1.10.3 Water Vapor Sorption Hydrolytic Stability (cycling)

Water vapor sorption cycling was performed at 25 °C on a DVS Intrinsic gravimetric instrument (Surface Measurement Systems) using dry air as a carrier gas. The mass of the sample was determined by comparison to an empty reference pan and recorded by a high-resolution microbalance with a precision of 0.1 μg. Prior to the measurement, each sample was placed in an aluminum pan, activated in-situ in dry air at temperature *T* (*T* was chosen depending on the sample and ranged between 40-100 °C) and for activation duration *t* (*t* was chosen depending on the sample and ranged between 60-120 minutes) using the built-in preheater, and consequently cooled to sorption temperature in 90 minutes. 400 sccm·min^{-1} flow (split between two samples) was used for the measurements. For each isotherm point, dm/dt ≤ 0.05 %·min^{-1} was used as criteria of reaching equilibrium.

Experimental RH-Swing cycling data were extrapolated to 500 cycles to provide an indication of longer-term performance. The uptake per cycle for **TIFSIX-25-Ni** and **TIFSIX-25-Co** was fitted using two models: single- and double-exponential decay functions (ExpDec1 and ExpDec2, respectively). ExpDec2 provided the best fit for both materials. Extrapolation to 500 cycles gives projected uptakes of 21.6 and 4.5 wt.% for **TIFSIX-25-Ni** and **TIFSIX-25-Co**, respectively, corresponding to absolute decreases of 5.8 and 9.6 wt.% from their initial uptakes, or approximately 21% and 71% total performance loss, respectively.

Experimental T-Swing cycling data were extrapolated to 500 cycles to provide an indication of longer-term performance.The uptake per cycle for **ROC-1** and **ROC-2** was fitted using two models: single- and double-exponential decay functions (ExpDec1 and ExpDec2, respectively). ExpDec2 provided the best fit for both materials. Extrapolation to 500 cycles gives projected uptakes of 1.0 and 2.45 wt.% for **ROC-1** and **ROC-2**, respectively, corresponding to absolute decreases of 12.9 and 8.5 wt.% from their initial uptakes, or approximately 50% and 79% total performance loss, respectively.

2 CSD survey

The Cambridge Structural database (CSD version 6.01, February 2026) was searched for ligand **25**. The search afforded 12 hits (Table S1), none of which were **M'FSIX** (M= Si, Ti, Ge etc) pillared materials.

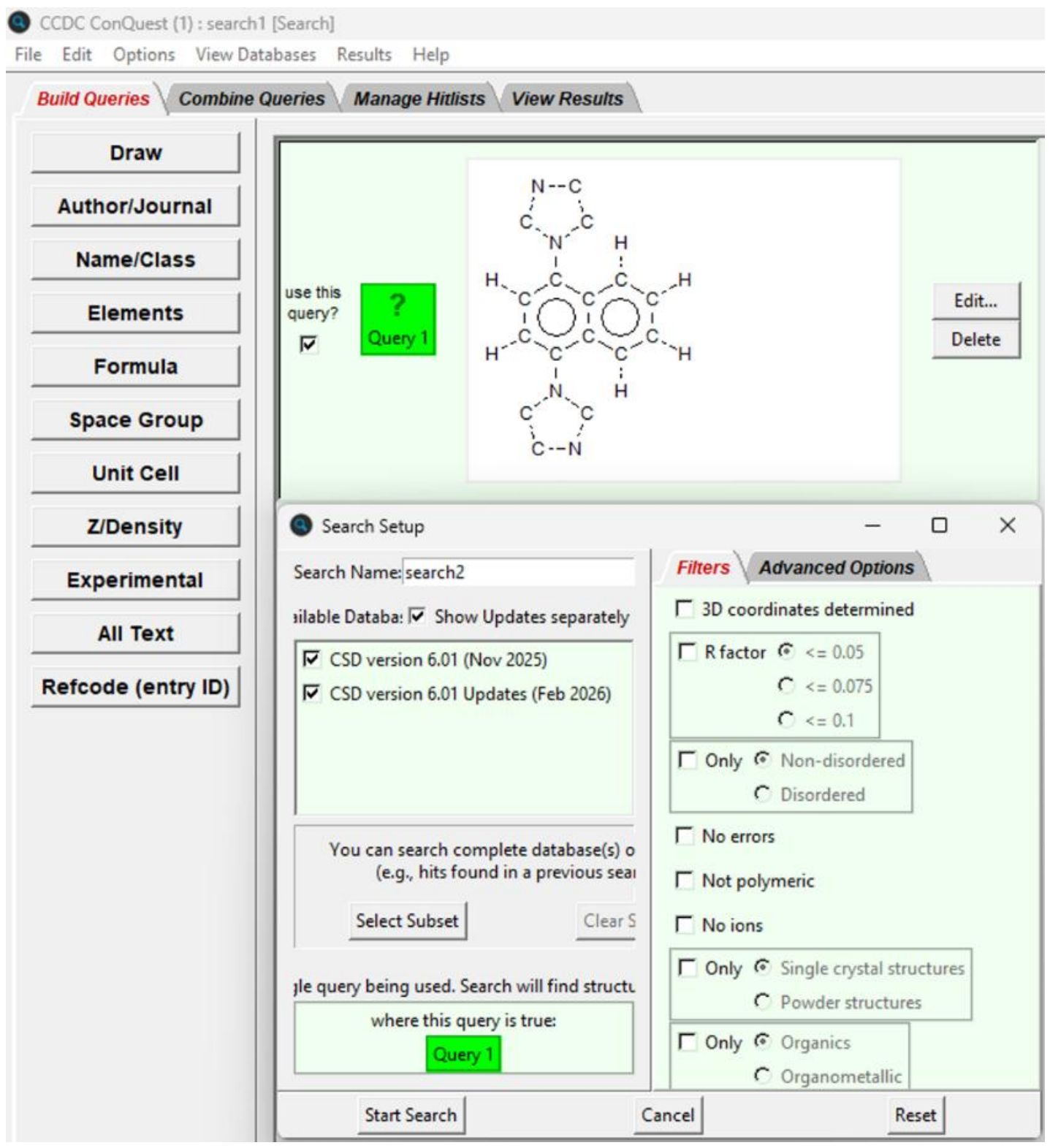

Figure S1: ConQuest queries used to search the CSD database for the **25** ligand.

Table S1: Complete list of CSD entries used in this study

Refcode	Dimension	Metal	Reference
COBXUQ	3D	Zn	[9]
COBYAX	3D	Zn	[9]
KONBOI	3D	Cd	[10]
KONCEZ	3D	Cd	[10]
KONCID	3D	Cd	[10]
NIMGUP	3D	Cd	[11]
NIMHAW	3D	Cd	[11]
NOHRIP	3D	Cu	[12]
NOHRIP01	3D	Cu	[12]
NOHROV	3D	Cu	[12]
NOHROV01	3D	Cu	[12]
WAVLOX	3D	Cu	[13]

3 Nomenclature

The nomenclature used for HCNs follows [topology]-A-L-M-[interpenetration], with classifiers in square parentheses included where necessary, was first introduced by us in 2012[14] for MO_4^{2-} (M = Cr, Mo) anions and 2013[15] for SiF_6^{2-} anions, and has been adopted by several research groups. Here the anion A is further distinguished by its chemical structure as A = M'FSIX for hexafluorometallate anions, M'OFFIVE for oxopentafluorometallate anions, M'FFIVE for pentafluorometallate anions and M'OOFOUR for tetraoxometallate anions. To date, 24 L-type ligands have been reported within this naming system (Figure S2). The ligand reported herein represents the 25th, hence the designation **M'FSIX-25-M**.

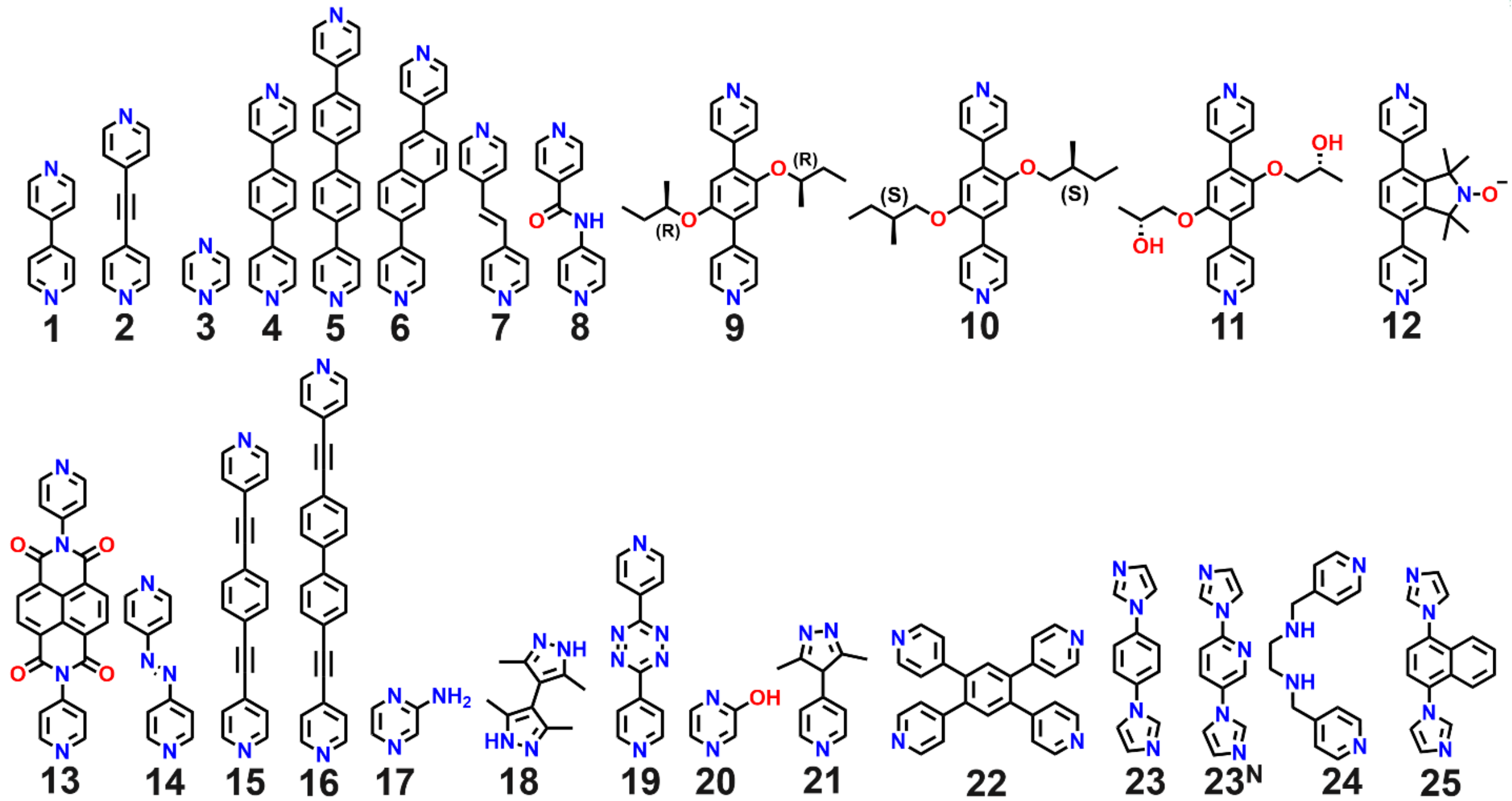

Figure S2: Representative N-donor ligands previously reported for the construction of hybrid ultramicroporous materials (M'FSIX pillared materials). Ligands **1–22** were summarized in the review by Li and coworkers,[16] whereas ligands **23**[17] and **24**[18] were reported separately. Ligand **25** was designed for present study.

4 Ligand Synthesis

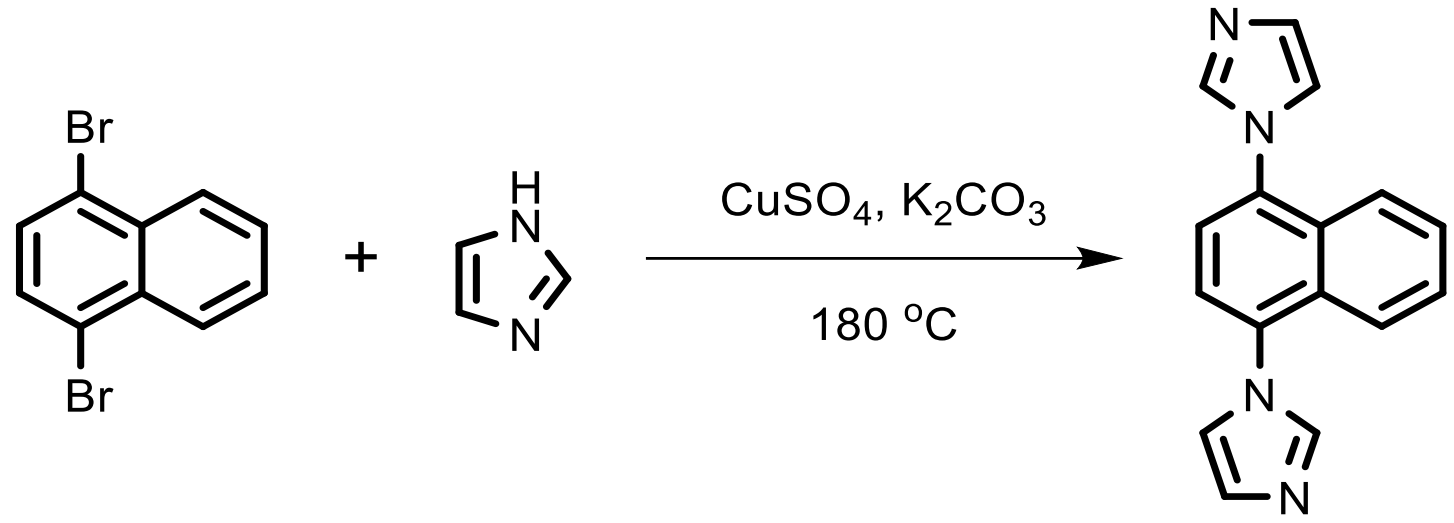

Scheme S3 The synthesis of imidazole ligand (**25**).

A mixture of 1,4-dibromonaphthalene (5.0 g, 17.5 mmol), imidazole (6.0 g, 87.4 mmol), $CuSO_4$ (280 mg, 1.7 mmol), and K_2CO_3 (4.8 g, 35.0 mmol) was stirred at 180 °C for 24 h under nitrogen atmosphere. After cooling down to room temperature, the solid mixture was dispersed in water (200 mL), filtered and washed with water (50 mL × 2). Then the crude product was dissolved in MeOH (100 mL). The mixture was filtered and the filtrate was concentrated to give 1,4-di(1*H*-imidazol-1-yl)naphthalene (**25**) as a light yellow solid (3.5 g, yield: 76.9%).

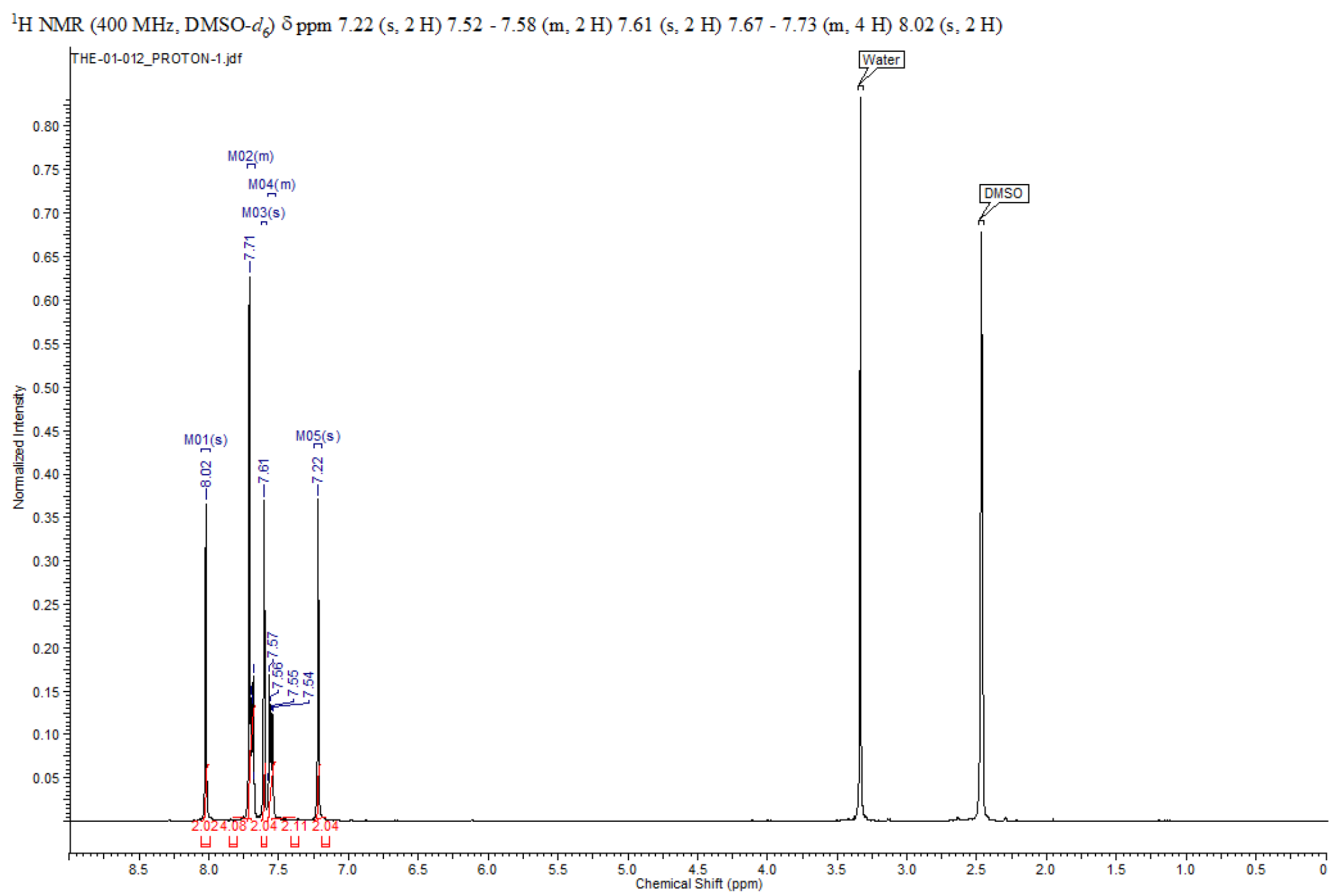

Figure S3 ^{1}H NMR of **25**.

5 Material Synthesis

5.1 Synthesis of **M'FSIX-3-M** compounds

5.1.1 Synthesis of {[Ni(pyrazine)$_2$(SiF$_6$)]}$_n$ (**pcu-SIFSIX-3-Ni**).

Nickel hexafluorosilicate hexahydrate ([Ni(H$_2$O)$_6$]SiF$_6$) (463 mg, 1.5 mmol) and pyrazine (240 mg, 3.0 mmol) were each dissolved in 1.5 mL of deionized water and mixed under ambient conditions. The resulting mixture was stirred for 72 h at room temperature to yield a polycrystalline 2D **sql** precursor, {[Ni(pyrazine)$_2$(H$_2$O)$_2$](SiF$_6$).2H$_2$O}$_n$ (**sql-SIFSIX-3-Ni**). This precursor was air dried and heated at 80 °C for 24 h using dynamic vacuum to obtain the desired **pcu-SIFSIX-3-Ni**.

5.1.2 Synthesis of {[Ni(pyrazine)$_2$(GeF$_6$)]}$_n$ (**pcu-GEFSIX-3-Ni**).

pcu-GEFSIX-3-Ni was synthesized in the same manner as **pcu-SIFSIX-3-Ni**, instead of ([Ni(H$_2$O)$_6$]SiF$_6$) salt, nickel hexafluorogermanate hexahydrate ([Ni(H$_2$O)$_6$]GeF$_6$) (530 mg, 1.5 mmol) salt was used.

5.1.3 Synthesis of {[Co(pyrazine)$_2$(SiF$_6$)]}$_n$ (**SIFSIX-3-Co**).

Single crystal of **SIFSIX-3-Co** was obtained by layering method. In a 10 mL tube, a methanolic solution (2 mL) of pyrazine (80 mg, 1 mmol) on an aqueous solution (2 mL) of cobalt hexafluorosilicate hexahydrate ([Co(H$_2$O)$_6$]SiF$_6$) (154 mg, 0.5 mmol). Crystals suitable for single-crystal X-ray diffraction (SCXRD) were obtained after 6 months. Bulk powder of **pcu-SIFSIX-3-Co** was obtained by slurry method. ([Co(H$_2$O)$_6$]SiF$_6$) (927 mg, 3.0 mmol) and pyrazine (480 mg, 6.0 mmol) were each dissolved in 2.5 mL of deionized water and mixed under ambient conditions. The resulting mixture was stirred for 24 h at room temperature to yield a polycrystalline 2D **sql** precursor, {[Co(pyrazine)$_2$(H$_2$O)$_2$](SiF$_6$).xH$_2$O}$_n$ (**sql-SIFSIX-3-Co**). This precursor was washed with methanol and air dried. Further heating at 80 °C for 24 h using dynamic vacuum to obtain the desired **pcu-SIFSIX-3-Co**.

5.1.4 Synthesis of {[Ni(pyrazine)$_2$(TiF$_6$)]}$_n$ (**pcu-TIFSIX-3-Ni**).

Following a reported procedure,[19] Nickel hexafluorotitanate hexahydrate ([Ni(H$_2$O)$_6$]TiF$_6$) (500 mg, 2.27 mmol) and an excess of pyrazine (2500 mg, 31.2 mmol) were each dissolved in 1.5 mL of deionized water and mixed under ambient conditions. The resulting mixture was stirred for 48 h at room temperature to yield a polycrystalline 2D **sql** precursor, {[Ni(pyrazine)$_2$(H$_2$O)$_2$](TiF$_6$).xH$_2$O}$_n$ (**sql-TIFSIX-3-Ni**). This precursor was air dried and heated at 140 °C for 24 h using dynamic vacuum to obtain the desired **pcu-TIFSIX-3-Ni**.

5.1.5 Synthesis of {[Co(pyrazine)$_2$(TiF$_6$)]}$_n$ (**pcu-TIFSIX-3-Co**).

Cobalt hexafluorotitanate hexahydrate ([Co(H$_2$O)$_6$]TiF$_6$) (328 mg, 1 mmol) and an excess of pyrazine (640 mg, 8 mmol) were each dissolved in 1 mL of deionized water and mixed under ambient conditions. The resulting mixture was stirred for 48 h at room temperature to yield a polycrystalline 2D **sql** precursor, {[Ni(pyrazine)$_2$(H$_2$O)$_2$](TiF$_6$).xH$_2$O}$_n$ (**sql-TIFSIX-3-Co**). This precursor was washed with methanol and air dried. Further heating at 100 °C for 24 h using dynamic vacuum to obtain the desired **pcu-TIFSIX-3-Co**.

5.1.6 Synthesis of {[Zn(pyrazine)$_2$(SiF$_6$)]}$_n$ (**pcu-SIFSIX-3-Zn**).

Zinc hexafluorosilicate hexahydrate ([Zn(H$_2$O)$_6$]SiF$_6$) (1800 mg, 6 mmol) and an excess of pyrazine (1040 mg, 13 mmol) were placed into a 35 mL teflon jar with two 10 mm teflon steel core grinding balls. The mixture was then ground for 60 minutes at 25 Hz in a Retsch MM400 laboratory ball mill to form polycrystalline 2D **sql** precursor, {[Zn(pyrazine)$_2$(H$_2$O)$_2$](SiF$_6$).xH$_2$O}$_n$ (**sql-SIFSIX-3-Zn**). This precursor was heated at 60 °C for 6 h using dynamic vacuum to obtain the desired **pcu-SIFSIX-3-Zn**.

5.1.7 Synthesis of {[Co(pyrazine)$_2$(GeF$_6$)]}$_n$ (**pcu-GEFSIX-3-Co**).

Cobalt hexafluorogermanate hexahydrate ([Co(H$_2$O)$_6$]GeF$_6$) (1060 mg, 3 mmol) and an excess of pyrazine (520 mg, 6.5 mmol) were placed into a 35 mL teflon jar with two 10 mm teflon steel core grinding balls. The mixture was then ground for 20 minutes at 25 Hz in a Retsch MM400 laboratory ball mill to form polycrystalline 2D **sql** precursor, {[Co(pyrazine)$_2$(H$_2$O)$_2$](GeF$_6$).xH$_2$O}$_n$ (**sql-GEFSIX-3-Co**). This precursor was heated at 140 °C for 18 h using dynamic vacuum to obtain the desired **pcu-GEFSIX-3-Co**.

5.2 Synthesis of **M'FSIX-25-M** compounds

Based on the imidazole ligand **25**, six HCNs were synthesized.

5.2.1 Synthesis of **SIFSIX-25-Co**.

A solution of CoSiF$_6$ (0.15 mg, 0.002 mmol) in 0.5 mL of H$_2$O was added to a NMR tube. MeOH: H$_2$O (v:v = 1:1, 0.5 mL) was carefully layered over this to act as a buffer layer. Finally, a solution of **25** (0.15 mg, 0.002 mmol) in 0.5 mL MeOH was layered over the buffer layer, and the tube was left undisturbed for 4 days. pink block crystals of [Co(25)$_2$SiF$_6$] (as-synthesised SIFSIX-25-Co) were obtained.

5.2.2 Synthesis of **SIFSIX-25-Ni**.

A solution of $NiSiF_6.6H_2O$ (0.15 mg, 0.002 mmol) in 0.5 mL of H_2O was added to a NMR tube. MeOH: H_2O (v:v = 1:1, 0.5 mL) was carefully layered over this to act as a buffer layer. Finally, a solution of **25** (0.15 mg, 0.002 mmol) in 0.5 mL MeOH was layered over the buffer layer, and the tube was left undisturbed for 4 days. pink block crystals of [Ni(**25**)$_2$SiF$_6$] (as-synthesised SIFSIX-25-Ni) were obtained.

5.2.3 Synthesis of **SIFSIX-25-Cu**.

A solution of $CuSiF_6.4H_2O$ (0.15 mg, 0.002 mmol) in 0.5 mL of H_2O was added to a NMR tube. MeOH: H_2O (v:v = 1:1, 0.5 mL) was carefully layered over this to act as a buffer layer. Finally, a solution of **25** (0.15 mg, 0.002 mmol) in 0.5 mL MeOH was layered over the buffer layer, and the tube was left undisturbed for 4 days. pink block crystals of [Cu(**25**)$_2$SiF$_6$] (as-synthesised SIFSIX-25-Cu) were obtained.

5.2.4 Synthesis of **TIFSIX-25-Co**.

A solution of $CoTiF_6.6H_2O$ (0.15 mg, 0.002 mmol) in 0.5 mL of H_2O was added to a NMR tube. MeOH: H_2O (v:v = 1:1, 0.5 mL) was carefully layered over this to act as a buffer layer. Finally, a solution of **25** (0.15 mg, 0.002 mmol) in 0.5 mL MeOH was layered over the buffer layer, and the tube was left undisturbed for 2 months. Pink block crystals of [Co(**25**)$_2$TiF$_6$] (as-synthesised TIFSIX-25-Co) were obtained.

5.2.5 Synthesis of **TIFSIX-25-Ni**.

A solution of $NiTiF_6.6H_2O$ (0.15 mg, 0.002 mmol) in 0.5 mL of H_2O was added to a NMR tube. MeOH: H_2O (v:v = 1:1, 0.5 mL) was carefully layered over this to act as a buffer layer. Finally, a solution of **25** (0.15 mg, 0.002 mmol) in 0.5 mL MeOH was layered over the buffer layer, and the tube was left undisturbed for 2 months. Light green block crystals of [Ni(**25**)$_2$TiF$_6$] (as-synthesised TIFSIX-25-Ni) were obtained.

5.2.6 Synthesis of **TIFSIX-25-Cu**.

A solution of $CuTiF_6.4H_2O$ (0.15 mg, 0.002 mmol) in 0.5 mL of H_2O was added to a NMR tube. MeOH: H_2O (v:v = 1:1, 0.5 mL) was carefully layered over this to act as a buffer layer. Finally, a solution of **25** (0.15 mg, 0.002 mmol) in 0.5 mL MeOH was layered over the buffer layer, and the tube was left undisturbed for 4 days. Purple block crystals of [Cu(**25**)$_2$TiF$_6$] (as-synthesised TIFSIX-25-Cu) were obtained.

5.2.7 Bulk synthesis of **M'FSIX-25-M** compounds

Bulk synthesis of TIFSIX-25-Ni and SIFSIX-25-Ni: A solution of 25 (100 mg, 0.38 mmol) in acetonitrile (15 mL) was prepared by sonication and added slowly to an aqueous solution (10 mL) of $NiM'F_6 \cdot 6H_2O$ (M′ = Si, Ti; 50 mg, ~0.16 mmol). The resulting clear solution was stirred at 200 rpm for 18 h at room temperature, affording fine microcrystalline powders, which were collected by filtration, washed with water and acetonitrile mixture, and dried under ambient conditions.

Bulk synthesis of TIFSIX-25-Co and SIFSIX-25-Co: An aqueous solution (10 mL) of $CoM'F_6 \cdot 6H_2O$ (M′ = Si, Ti; 50 mg, ~0.16 mmol) was added dropwise to a solution of **25** (100 mg, 0.38 mmol) in acetonitrile (15 mL), prepared by sonication. The resulting clear solution was stirred at 200 rpm for 18 h at room temperature, affording fine microcrystalline powders.

Bulk synthesis of TIFSIX-25-Cu and SIFSIX-25-Cu: A solution of **25** (100 mg, 0.38 mmol) in methanol (10 mL) was mixed with an aqueous solution (10 mL) of $CuM'F_6 \cdot 4H_2O$ (M′ = Si, Ti; 50 mg, ~0.16 mmol). Immediate formation of a fine microcrystalline precipitate was observed. The suspension was stirred at 200 rpm for 18 h at room temperature, and the product was collected by filtration, washed with water and methanol, and dried under ambient conditions.

6 Crystallographic tables and analysis

Table S2 Crystallographic data and structure refinements for **M'FSIX-25-M** compounds.

Name	**sql-SIFSIX-25-Co**	**pcu-SIFSIX-25-Co**	**sql-TIFSIX-25-Co**	**pcu-TIFSIX-25-Co**
Formula	$C_{32}H_{28}CoF_6N_8O_2Si$	$C_{32}H_{24}CoF_6N_8Si$	$C_{32}H_{28}CoF_6N_8O_2Ti$	$C_{32}H_{24}CoF_6N_8Ti$
M.W.	757.64	721.61	777.45	741.42
Crystal system	Monoclinic	Monoclinic	Monoclinic	Monoclinic
Space group	*C2/c*	*C2/c*	*C2/c*	*C2/c*
a/ Å	16.351(3)	17.328(4)	16.958(2)	16.7521(12)
b/ Å	22.505(3)	21.167(5)	22.246(2)	21.7804(14)
c/ Å	13.033(4)	11.987(3)	13.535(3)	12.2634(16)
α/ °	90	90	90	90
β/ °	122.640(4)	115.389(13)	122.535(2)	120.2310(10)
γ/ °	90	90	90	90
V/ Å^3	4038.5(14)	3972.1(17)	4305.0(11)	3866.0(6)
Void/ %	29.9	33.9	33.3	30.4
D_C /g cm^{-3}	1.246	1.207	1.200	1.274
μ /mm^{-1}	0.519	4.193	0.631	0.965
T/ K	101	150	150	100
Reflections collected	24323	14128	29154	26532
Independent reflections	4637 R_{int} = 0.0801	3701 R_{int} = 0.1280	4916 R_{int} = 0.0779	4457 R_{int} = 0.0462
Goodness-of-fit on F^2	1.026	1.017	1.039	1.054
$R_1{}^a$, $wR_2{}^b$ [$I > 2\sigma(I)$]	R_1 = 0.0726 wR_2 = 0.1968	R_1 = 0.0860 wR_2 = 0.2254	R_1 = 0.0858 wR_2 = 0.2400	R_1 = 0.0487 wR_2 = 0.1346
$R_1{}^a$, $wR_2{}^b$ (all data)	R_1 = 0.1093 wR_2 = 0.2180	R_1 = 0.1237 wR_2 = 0.2526	R_1 = 0.1287 wR_2 = 0.2713	R_1 = 0.0648 wR_2 = 0.1436
Largest diff. peak and hole (e.Å^{-3})	0.66/–0.49	0.83 / –0.47	1.37 / –0.86	0.63/–1.07

^{a} $R_1=\Sigma||F_o|-|F_c|| / \Sigma|F_o|$

^{b} $wR_2=\{\Sigma[w(F_o^2-F_c^2)^2]/[w(F_o^2)^2]\}^{1/2}$, $[F_o>4\sigma(F_o)]$

Table S3 Crystallographic data and structure refinements for **M'FSIX-25-M** compounds.

Name	**sql-SIFSIX-25-Ni**	**pcu-SIFSIX-25-Ni**	**sql-TIFSIX-25-Ni**	**pcu-TIFSIX-25-Ni**
Formula	$C_{32}H_{28}F_6N_8NiO_2Si$	$C_{32}H_{24}F_6N_8NiSi$	$C_{32}H_{28}F_6N_8NiO_2Ti$	$C_{32}H_{24}F_6N_8NiTi$
M.W.	757.42	721.39	777.23	741.20
Crystal system	Monoclinic	Monoclinic	Monoclinic	Monoclinic
Space group	*C2/c*	*C2/c*	*C2/c*	*C2/c*
a/ Å	16.6450(8)	17.404(3)	16.8652(10)	17.3404(15)
b/ Å	22.5284(11)	21.021(3)	22.1503(12)	21.2835(17)
c/ Å	13.5743(11)	11.9766(17)	13.3594(8)	12.2710(9)
α/ °	90	90	90	90
β/ °	124.431(2)	115.838(7)	122.416(2)	118.155(5)
γ/ °	90	90	90	90
V/ Å^3	4198.4(5)	3943.7(10)	4213.0(4)	3992.9(6)
Void/ %	32.2	33.5	31.8	33.3
D_C /g cm^{-3}	1.198	1.215	1.225	1.233
μ /mm^{-1}	1.487	1.521	2.709	2.797
T/ K	150	150	150	150
Reflections collected	21952	13749	23866	23535
Independent reflections	4148 R_{int} = 0.0534	3640 R_{int} = 0.0749	4158 R_{int} = 0.0512	3943 R_{int} = 0.0716
Goodness-of-fit on F^2	1.115	1.043	1.081	1.114
R_1[a], wR_2[b] [$I > 2\sigma(I)$]	R_1 = 0.0873 wR_2 = 0.2655	R_1 = 0.0875 wR_2 = 0.2341	R_1 = 0.0477 wR_2 = 0.1243	R_1 = 0.0853 wR_2 = 0.2450
R_1[a], wR_2[b] (all data)	R_1 = 0.0920 wR_2 = 0.2697	R_1 = 0.1056 wR_2 = 0.2558	R_1 = 0.0513 wR_2 = 0.1277	R_1 = 0.0987 wR_2 = 0.2603
Largest diff. peak and hole (e.Å^{-3})	0.80/–1.11	1.26/–1.08	0.58/–0.56	1.10/–1.11

[a] $R_1=\Sigma||F_o|-|F_c|| / \Sigma|F_o|$

[b] $wR_2=\{\Sigma[w(F_o^2-F_c^2)^2]/[w(F_o^2)^2]\}^{1/2}$, $[F_o>4\sigma(F_o)]$

Table S4 Crystallographic data and structure refinements for **M'FSIX-25-M** compounds and **SIFSIX-3-Co**.

Name	**sql-TIFSIX-25-Ni (loaded)**	**pcu-SIFSIX-25-Cu**	**pcu-TIFSIX-25-Cu**	**SIFSIX-3-Co**
Formula	$C_{32}H_{28}F_6N_8NiO_{9.68}Ti$	$C_{32}H_{24}CuF_6N_8Si$	$C_{32}H_{24}CuF_6N_8Ti$	$C_8H_{22}CoF_6N_4O_3Si$
M.W.	900.11	726.22	746.03	423.31
Crystal system	Monoclinic	Monoclinic	Monoclinic	Tetragonal
Space group	*C2/c*	*C2/c*	*C2/c*	*P4/mmm*
a/ Å	16.758(3)	17.3594(8)	17.707(2)	7.1017(2)
b/ Å	22.255(4)	20.8978(9)	21.368(2)	7.1017(2)
c/ Å	13.397(3)	12.1765(5)	12.7601(14)	7.5476(4)
α/ °	90	90	90	90
β/ °	123.25(3)	116.703(2)	120.759(4)	90
γ/ °	90	90	90	90
V/ $Å^3$	4178.3(19)	3946.2(3)	4148.7(8)	380.66(3)
Void/ %	NA	34.0	35.8	24.0
D_C /g cm^{-3}	1.431	1.222	1.194	1.847
μ /mm^{-1}	0.727	1.592	0.761	1.290
T/ K	293(2)	153	104	150
Reflections collected	24408	22706	52304	5053
Independent reflections	3680 R_{int} = 0.0452	3848 R_{int} = 0.0477	4876 R_{int} = 0.1211	229 R_{int} = 0.0513
Goodness-of-fit on F^2	1.062	1.085	1.055	1.013
R_1[a], wR_2[b] [$I > 2\sigma(I)$]	R_1 = 0.0621 wR_2 = 0.1942	R_1 = 0.0384 wR_2 = 0.1048	R_1 = 0.0987 wR_2 = 0.2518	R_1 = 0.0249, wR_2 = 0.0855
R_1[a], wR_2[b] (all data)	R_1 = 0.0668 wR_2 = 0.2003	R_1 = 0.0390 wR_2 = 0.1063	R_1 = 0.1278 wR_2 = 0.2678	*R1* = 0.0266, *wR2* = 0.0869
Largest diff. peak and hole (e.$Å^{-3}$)	0.786/-1.116	0.53/–0.72	0.58/–1.12	0.291 and -0.311

[a] $R_1=\Sigma||F_o|-|F_c|| / \Sigma|F_o|$

[b] $wR_2=\{\Sigma[w(F_o^2-F_c^2)^2]/[w(F_o^2)^2]\}^{1/2}$, $[F_o>4\sigma(F_o)]$

Table S5 Pore limiting diameter d_{lim}, maximum pore diameter d_{max} and surface area (SA) determined using the pore analyzer feature in Mercury[8] for **M'FSIX-25-M** family.

	**sql**			**pcu**		
	d_{lim} (Å)	d_{max} (Å)	SA (m^2 g^{-1})	d_{lim} (Å)	d_{max} (Å)	SA (m^2 g^{-1})
**SIFSIX-25-Co**	3.7	4.9	235	4.0	4.7	335
**SIFSIX-25-Ni**	3.7	5.4	335	4.1	4.6	353
**SIFSIX-25-Cu**	NA	NA	NA	4.1	4.7	405
**TIFSIX-25-Co**	4.2	5.2	430	4.0	4.7	222
**TIFSIX-25-Ni**	4.1	5.1	353	4.1	4.8	382
**TIFSIX-25-Cu**	NA	NA	NA	4.3	5.5	477

6.1 Crystal structures of **sql-M'FSIX-25-M**

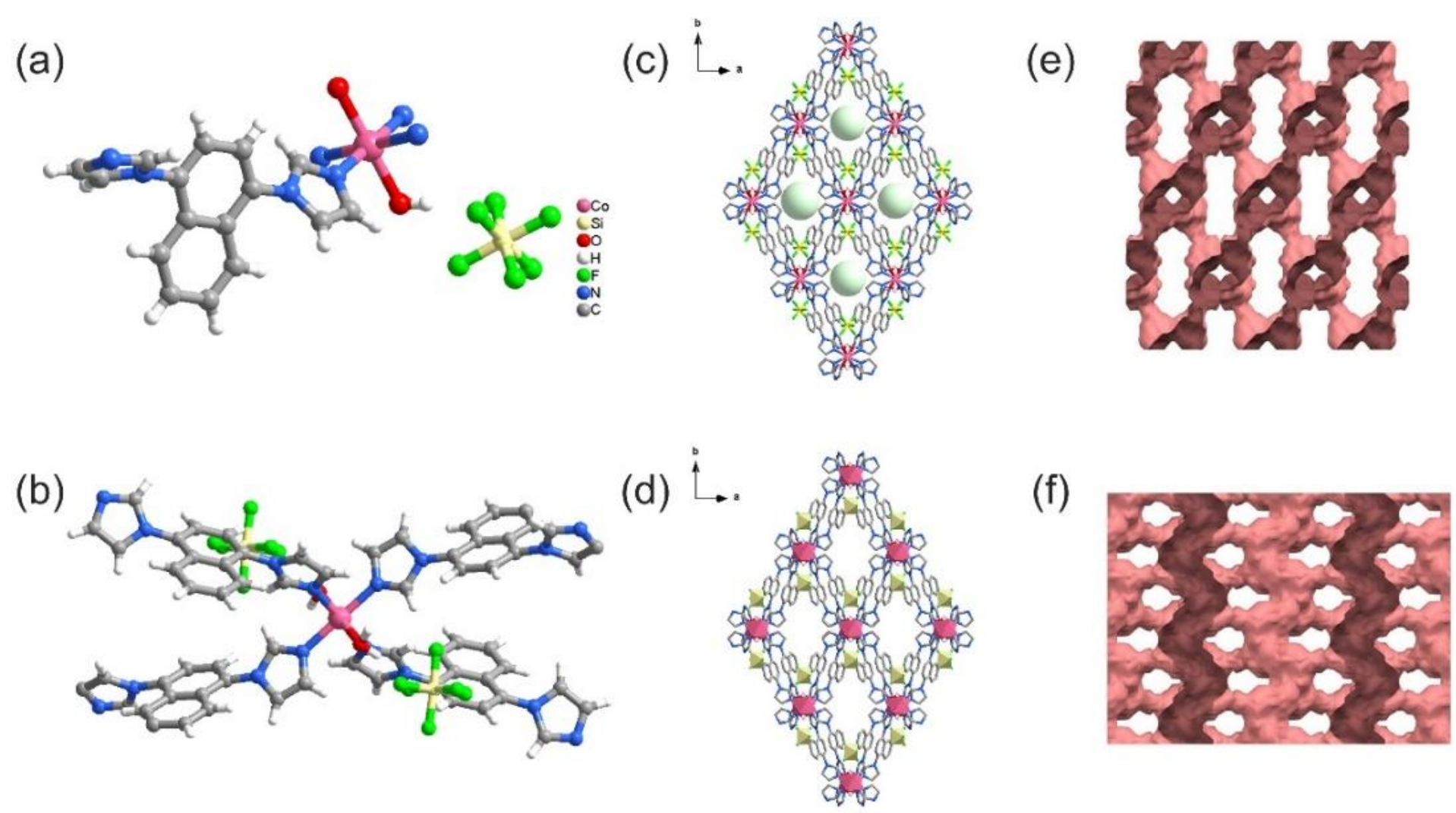

Figure S4 Crystals structure of **sql-SIFSIX-25-Co** generated in Mercury: (a,b) Ball-and-stick representation, (c,d) framework structure viewed along [001] and solvent-accessible surface areas viewed along (e) [001] and (f) [100].

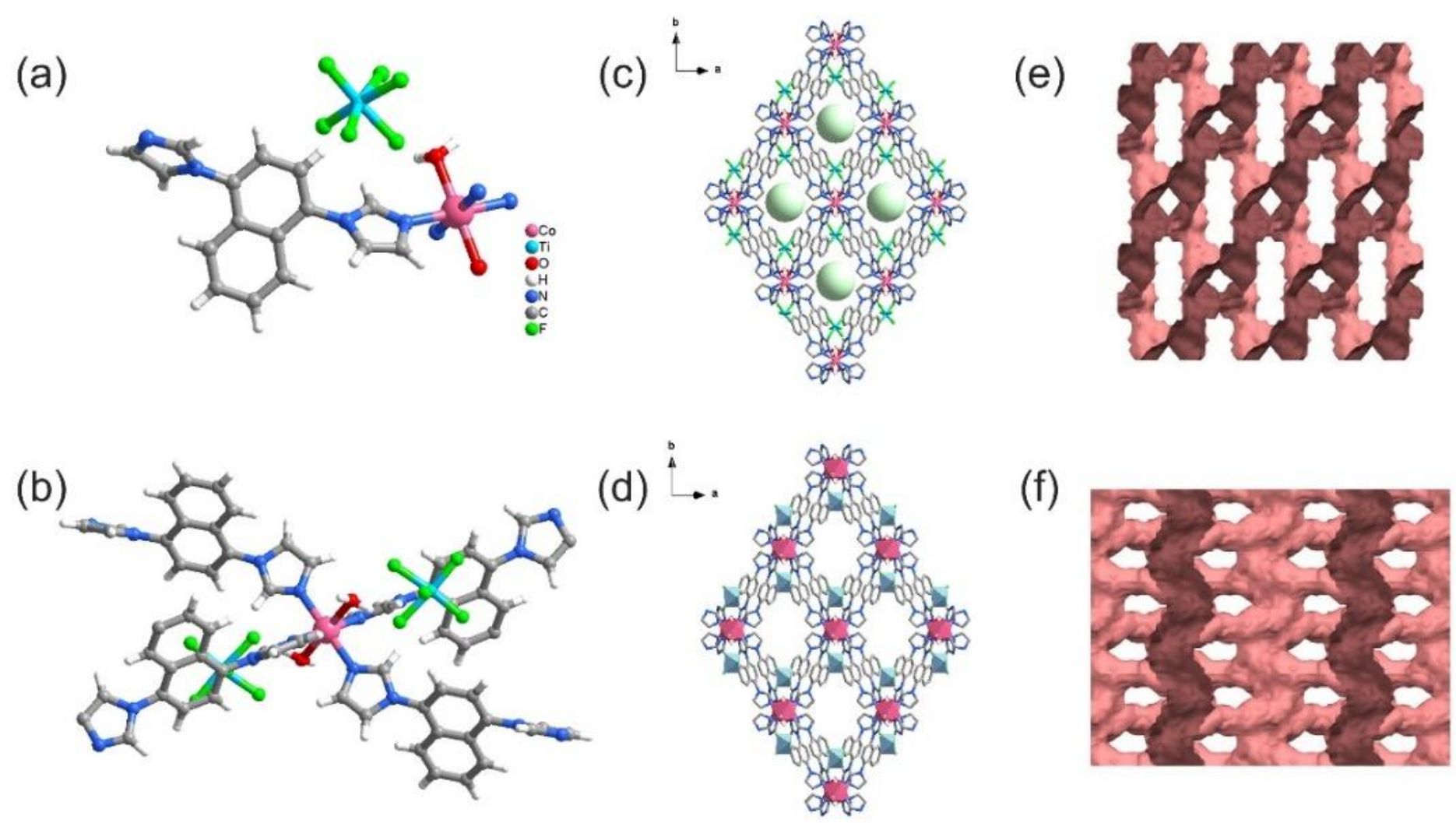

Figure S5 Crystals structure of **sql-TIFSIX-25-Co** generated in Mercury: (a,b) Ball-and-stick representation, (c,d) framework structure viewed along [001] and solvent-accessible surface areas viewed along (e) [001] and (f) [100].

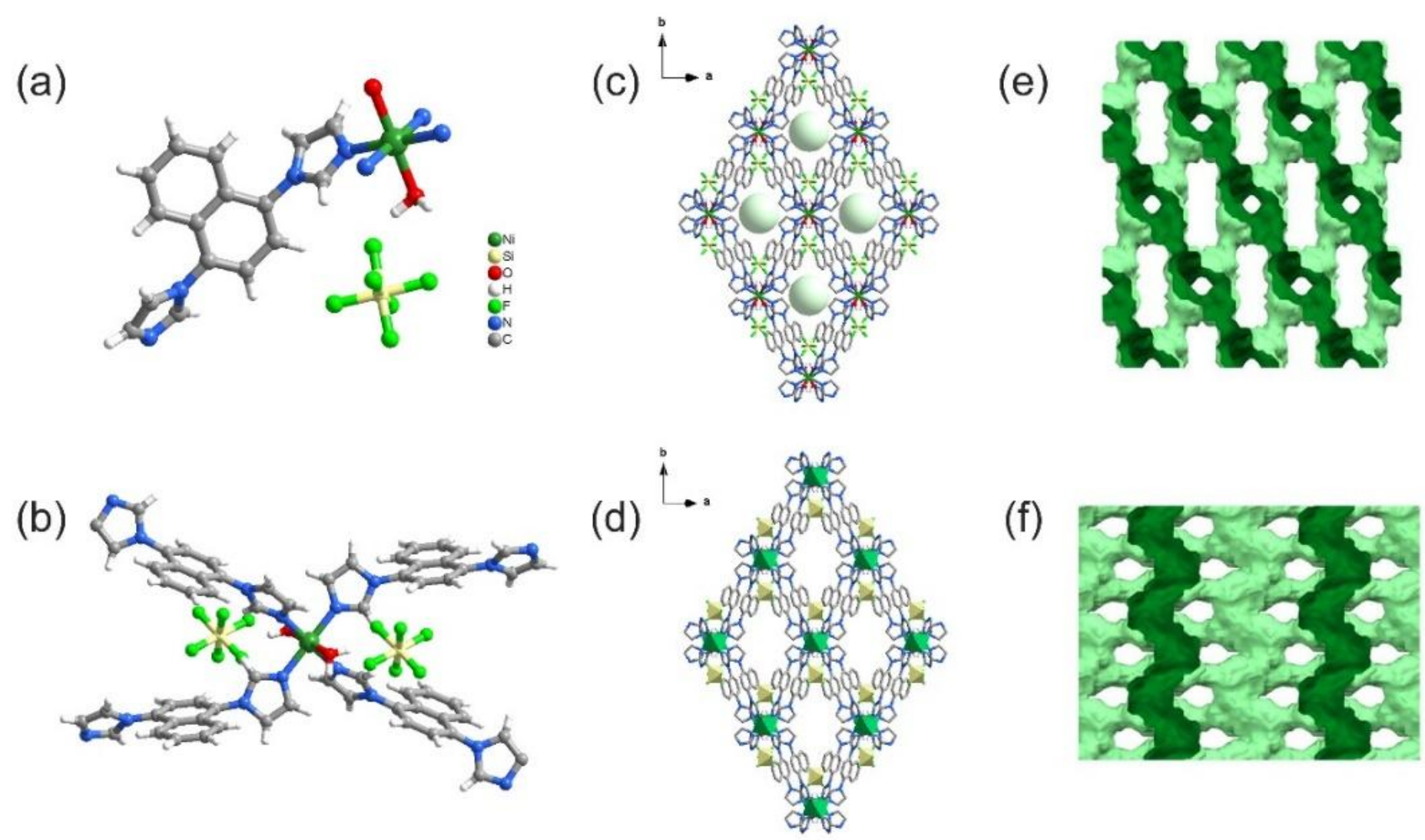

Figure S6 Crystals structure of **sql-SIFSIX-25-Ni** generated in Mercury: (a,b) Ball-and-stick representation, (c,d) framework structure viewed along [001] and solvent-accessible surface areas viewed along (e) [001] and (f) [100].

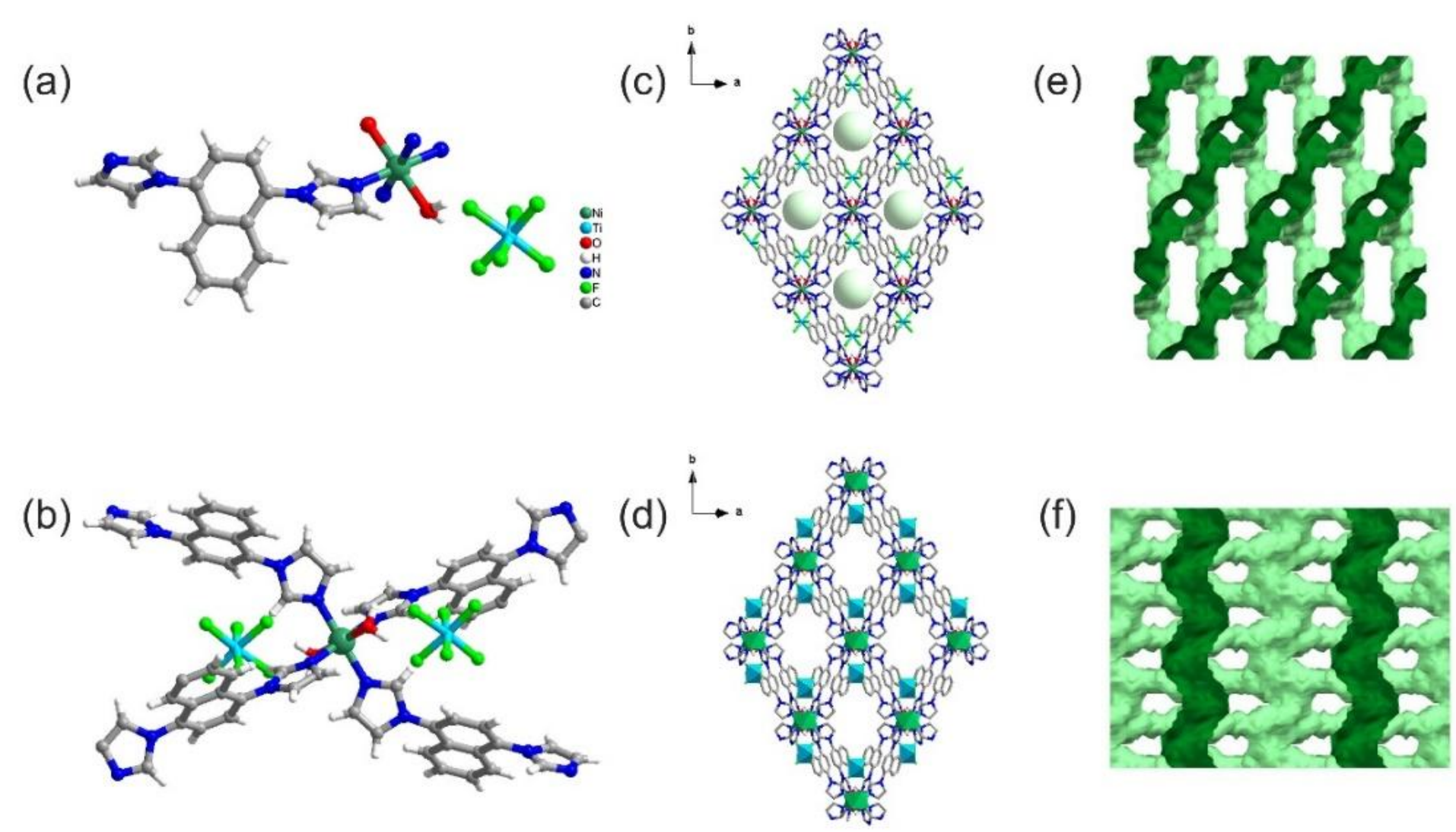

Figure S7 Crystals structure of **sql-TIFSIX-25-Ni** generated in Mercury: (a,b) Ball-and-stick representation, (c,d) framework structure viewed along [001] and solvent-accessible surface areas viewed along (e) [001] and (f) [100].

6.2 Crystal structures of **pcu-M'FSIX-25-M**

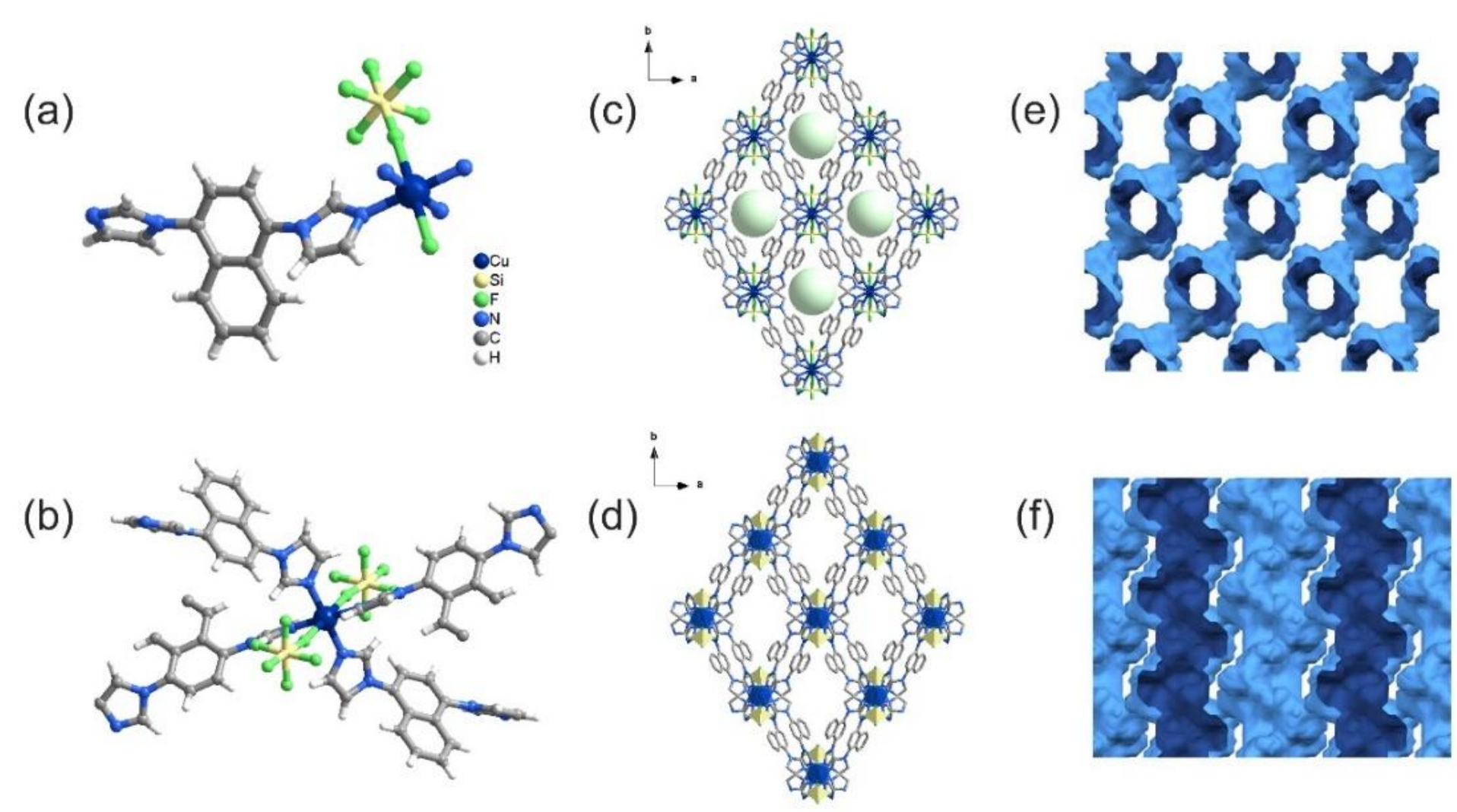

Figure S8 Crystals structure of **pcu-SIFSIX-25-Cu** generated in Mercury: (a,b) Ball-and-stick representation, (c,d) framework structure viewed along [001] and solvent-accessible surface areas viewed along (e) [001] and (f) [100].

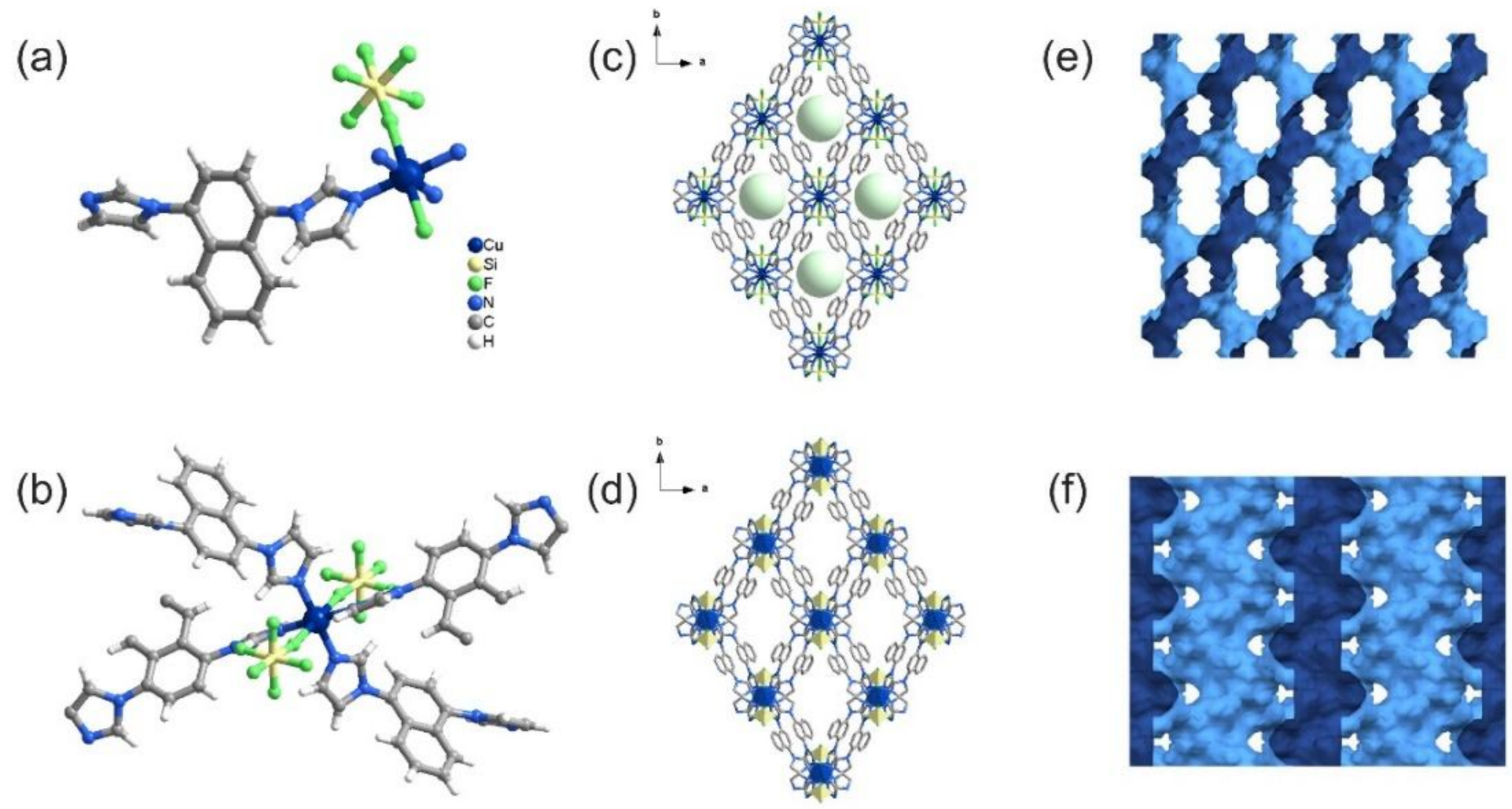

Figure S9 Crystals structure of **pcu-TIFSIX-25-Cu** generated in Mercury: (a,b) Ball-and-stick representation, (c,d) framework structure viewed along [001] and solvent-accessible surface areas viewed along (e) [001] and (f) [100].

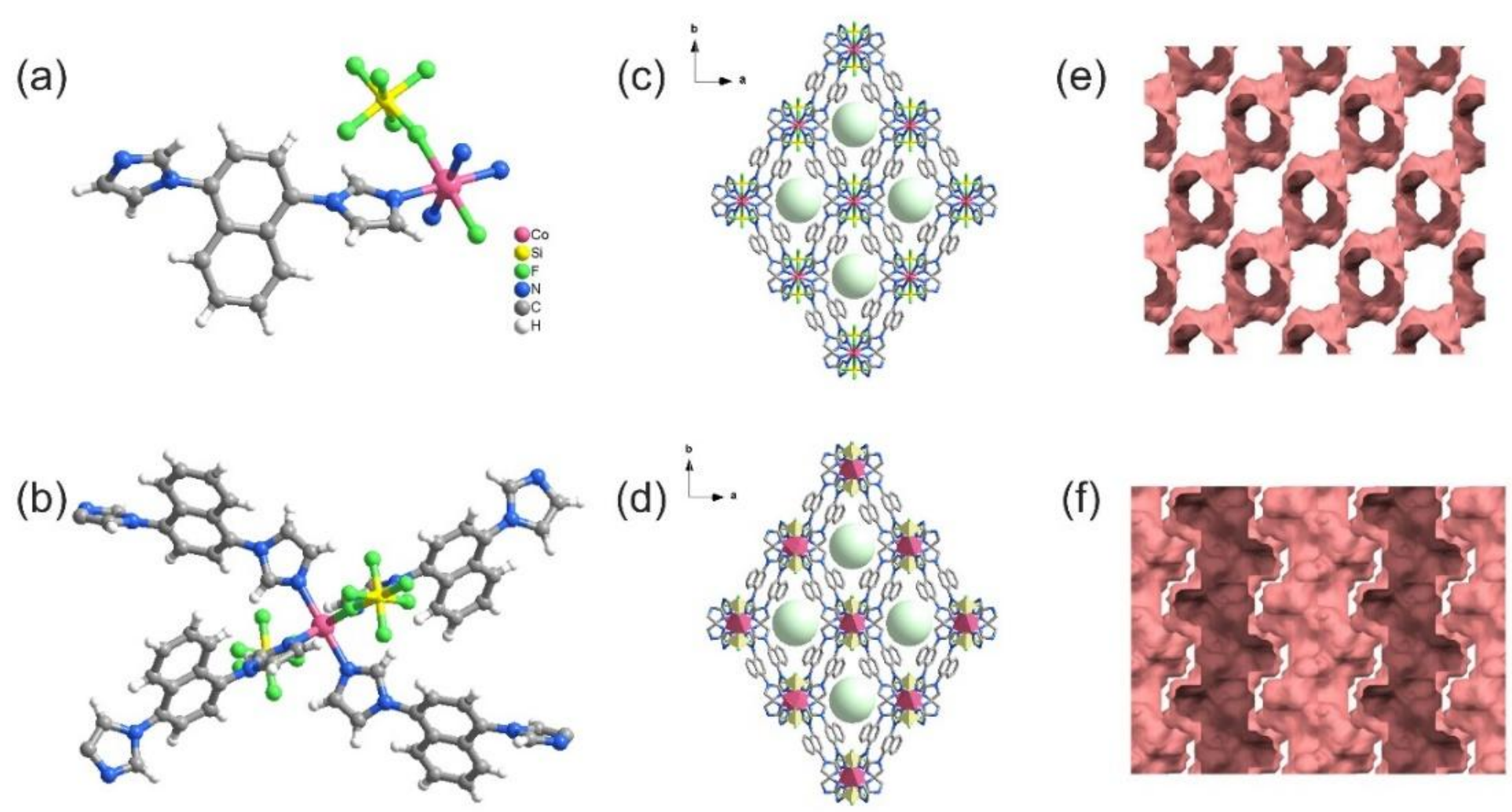

Figure S10 Crystals structure of **pcu-SIFSIX-25-Co** generated in Mercury: (a,b) Ball-and-stick representation, (c,d) framework structure viewed along [001] and solvent-accessible surface areas viewed along (e) [001] and (f) [100].

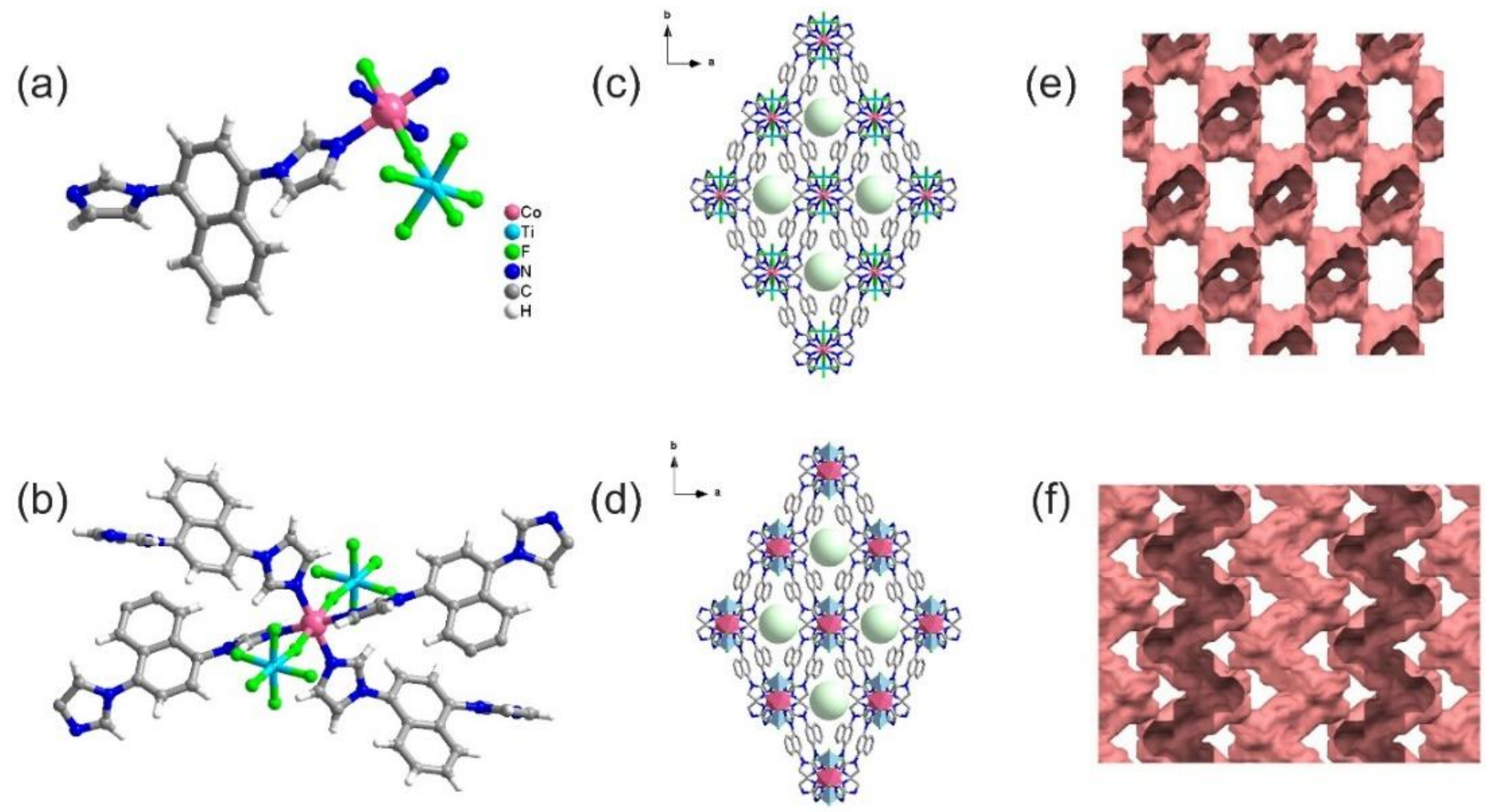

Figure S11 Crystals structure of **pcu-TIFSIX-25-Co** generated in Mercury: (a,b) Ball-and-stick representation, (c,d) framework structure viewed along [001] and solvent-accessible surface areas viewed along (e) [001] and (f) [100].

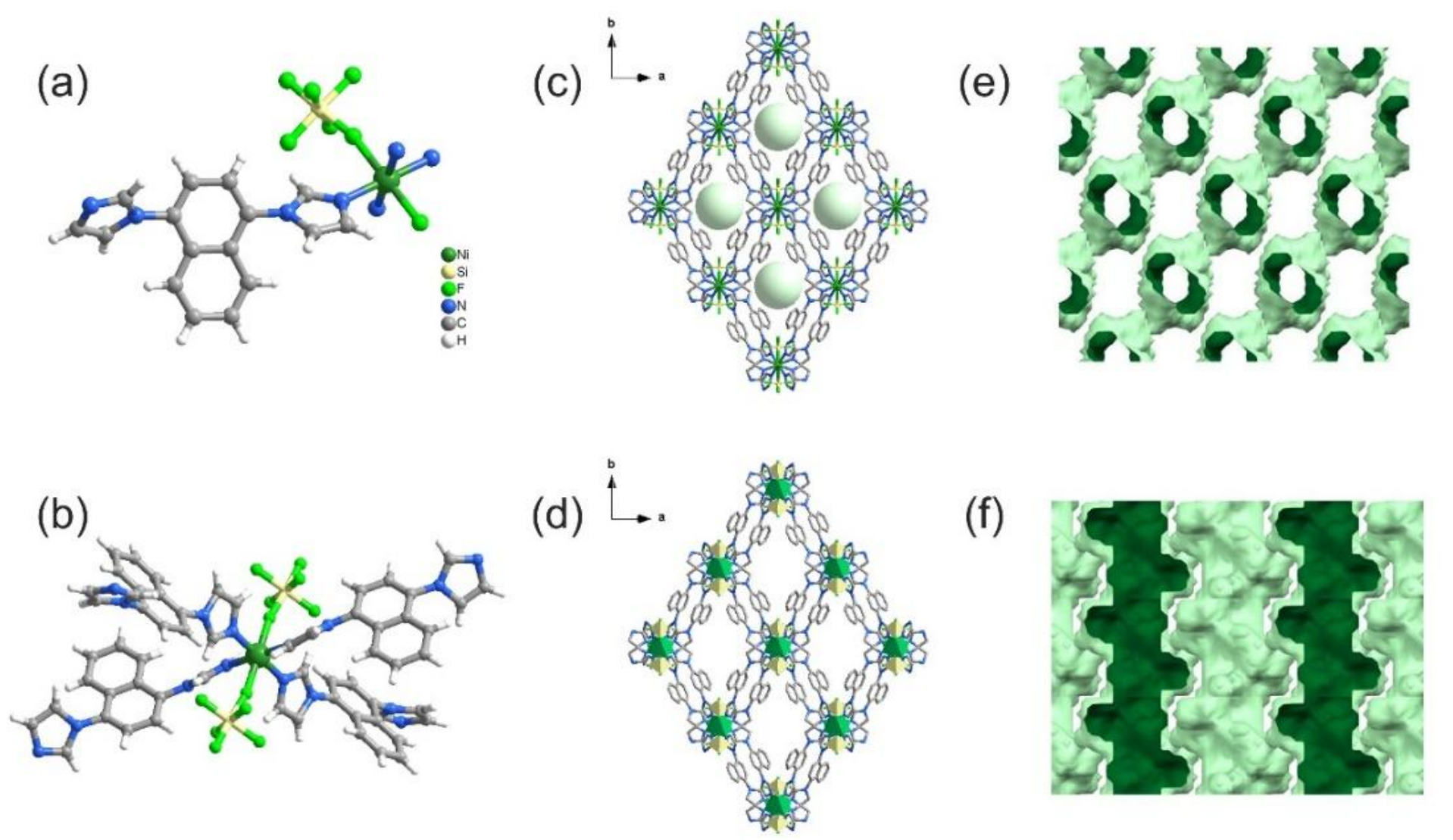

Figure S12 Crystals structure of **pcu-SIFSIX-25-Ni** generated in Mercury: (a,b) Ball-and-stick representation, (c,d) framework structure viewed along [001] and solvent-accessible surface areas viewed along (e) [001] and (f) [100].

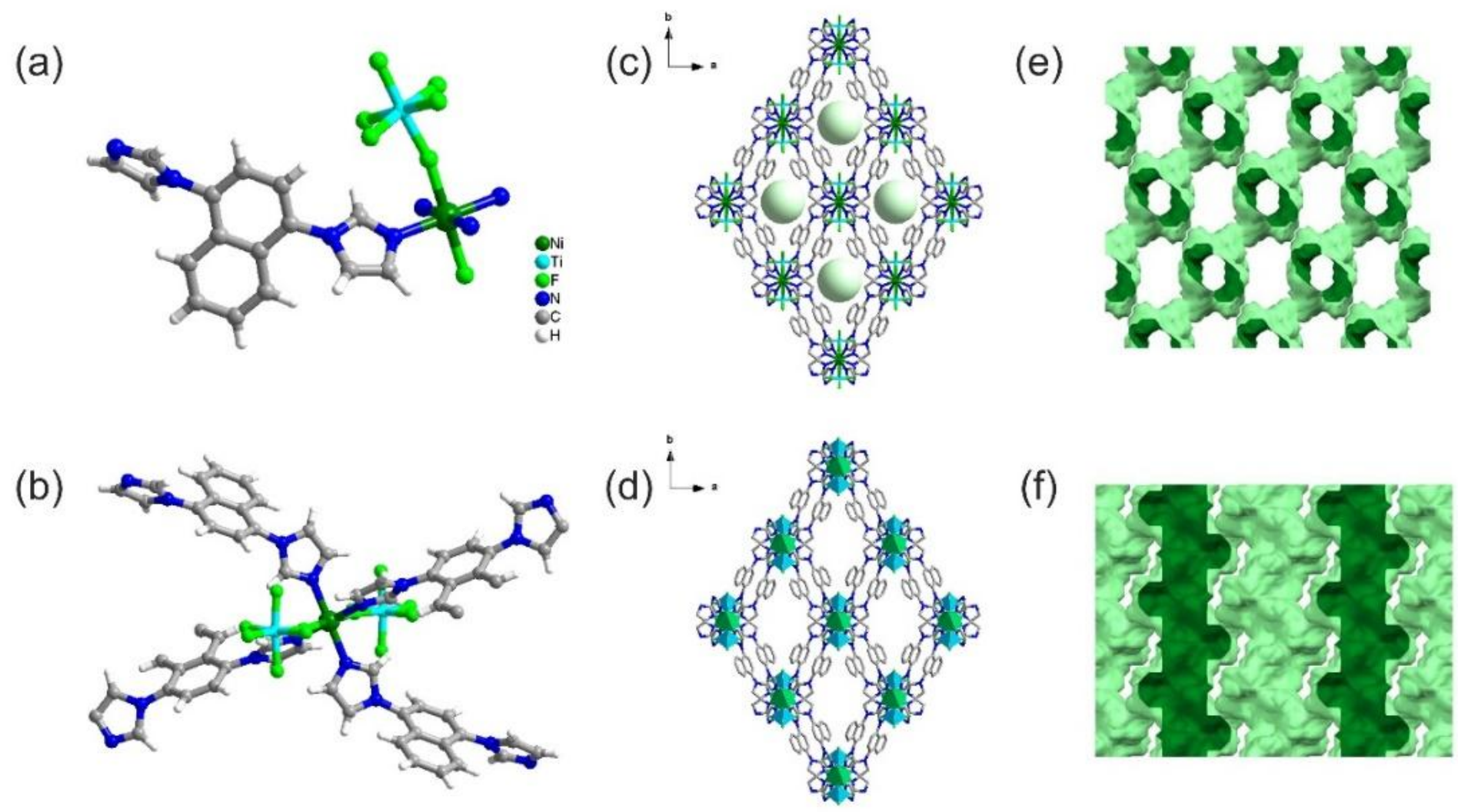

Figure S13 Crystals structure of **pcu-TIFSIX-25-Ni** generated in Mercury: (a,b) Ball-and-stick representation, (c,d) framework structure viewed along [001] and solvent-accessible surface areas viewed along (e) [001] and (f) [100].

Table S6 Calculated and measured H_2O molecules desorbed from **sql-M'FSIX-3-M** compounds from thermal analysis.

Anhydrous Formula unit	Formula weight (g mol^{-1})	Weight loss in TGA (%)	Equivalent weight per H_2O molecule (%)	Calculated number of H_2O molecules desorbed	Calculated number of coordinated H_2O molecules desorbed	Calculated number of lattice H_2O molecules desorbed	Enthalpy of dehydration (kJ mol^{-1}) From DSC	Temperature range integrated for enthalpy calculation (°C)
$[Ni(C_4N_2H_4)_2 \cdot SiF_6]$	360.94	19	5	4	2$	2$	42	40-100
$[Ni(C_4N_2H_4)_2 \cdot TiF_6]$	380.73	16	4.7	3.4	2	1.4	26	27-139
$[Ni(C_4N_2H_4)_2 \cdot GeF_6]$	405.50	18	4.4	4.0	2	2	35	40-120
$[Co(C_4N_2H_4)_2 \cdot SiF_6]$	361.18	17	4.9	3.4	2	~2	51	44-115
$[Zn(C_4N_2H_4)_2 \cdot SiF_6]$	367.64	17	4.8	3.5	2	1.5	38	40-107
$[Co(C_4N_2H_4)_2 \cdot GeF_6]$	405.74	18	4.4	4.0	2	2	56	39-130
$[Co(C_4N_2H_4)_2 \cdot TiF_6]$	380.97	17	4.7	3.6	2	1.6	53	46-136

$values as per CSD (refcode: POCREI).

7 Powder X-ray diffraction

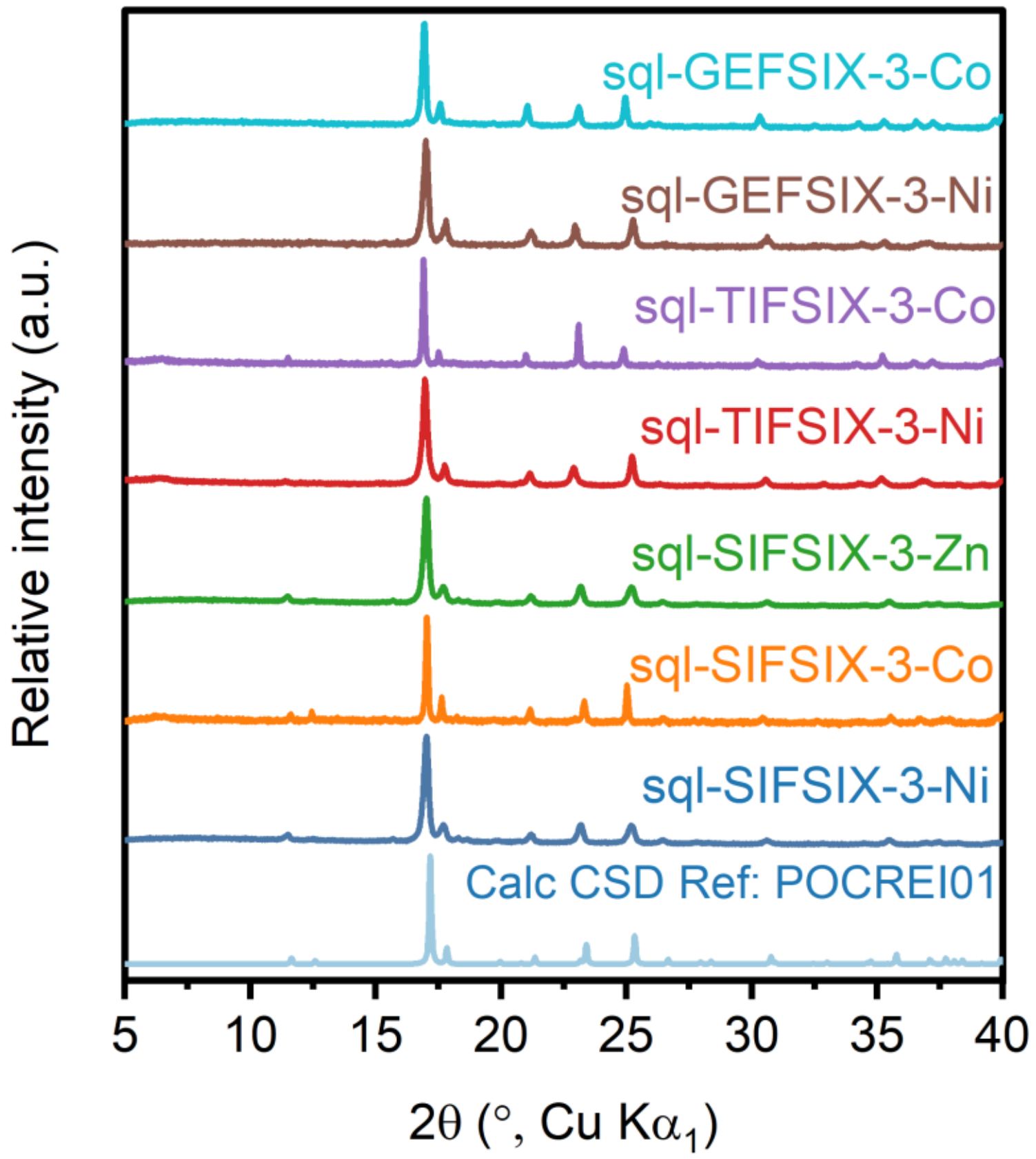

Figure S14 PXRD of as synthesized **sql-M'FSIX-3-M** (bottom to top): **SIFSIX-3-Ni**, **SIFSIX-3-Co, SIFSIX-3-Zn, TIFSIX-3-Ni, TIFSIX-3-Co, GEFSIX-3-Ni, GEFSIX-3-Co.** Experimental patterns collected at room temperature are shown alongside calculated pattern of **sql-SIFSIX-Ni** (bottom) generated from published data archived in CSD (Refcode **POCREI01**).

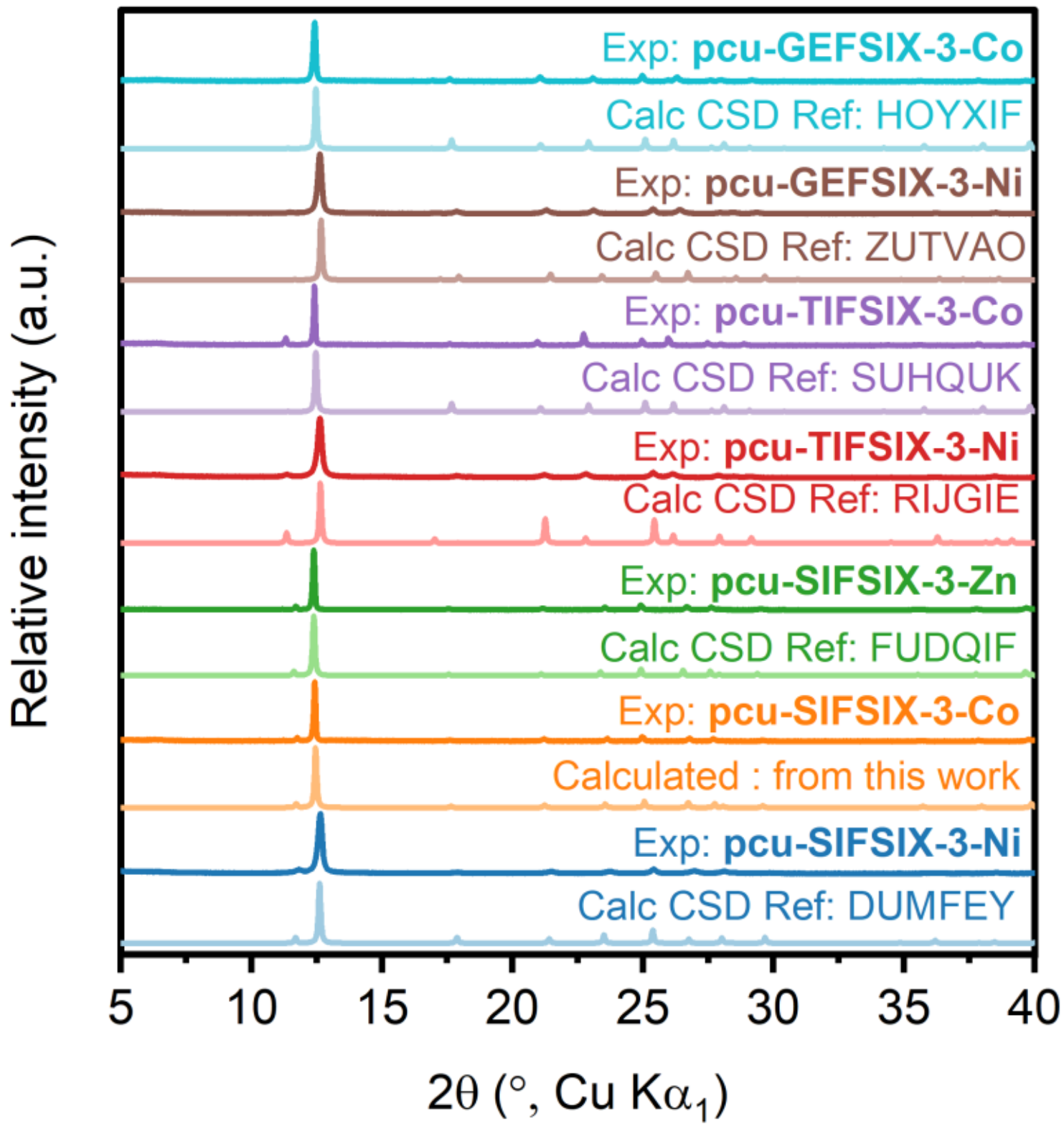

Figure S15 PXRD of **pcu-M'FSIX-3-M** HCNs (bottom to top): **SIFSIX-3-Ni**, **SIFSIX-3-Co, SIFSIX-3-Zn, TIFSIX-3-Ni, TIFSIX-3-Co, GEFSIX-3-Ni, GEFSIX-3-Co.** Experimental patterns collected at room temperature are shown alongside calculated patterns generated from published data archived in CSD (reference codes indicated).[6]

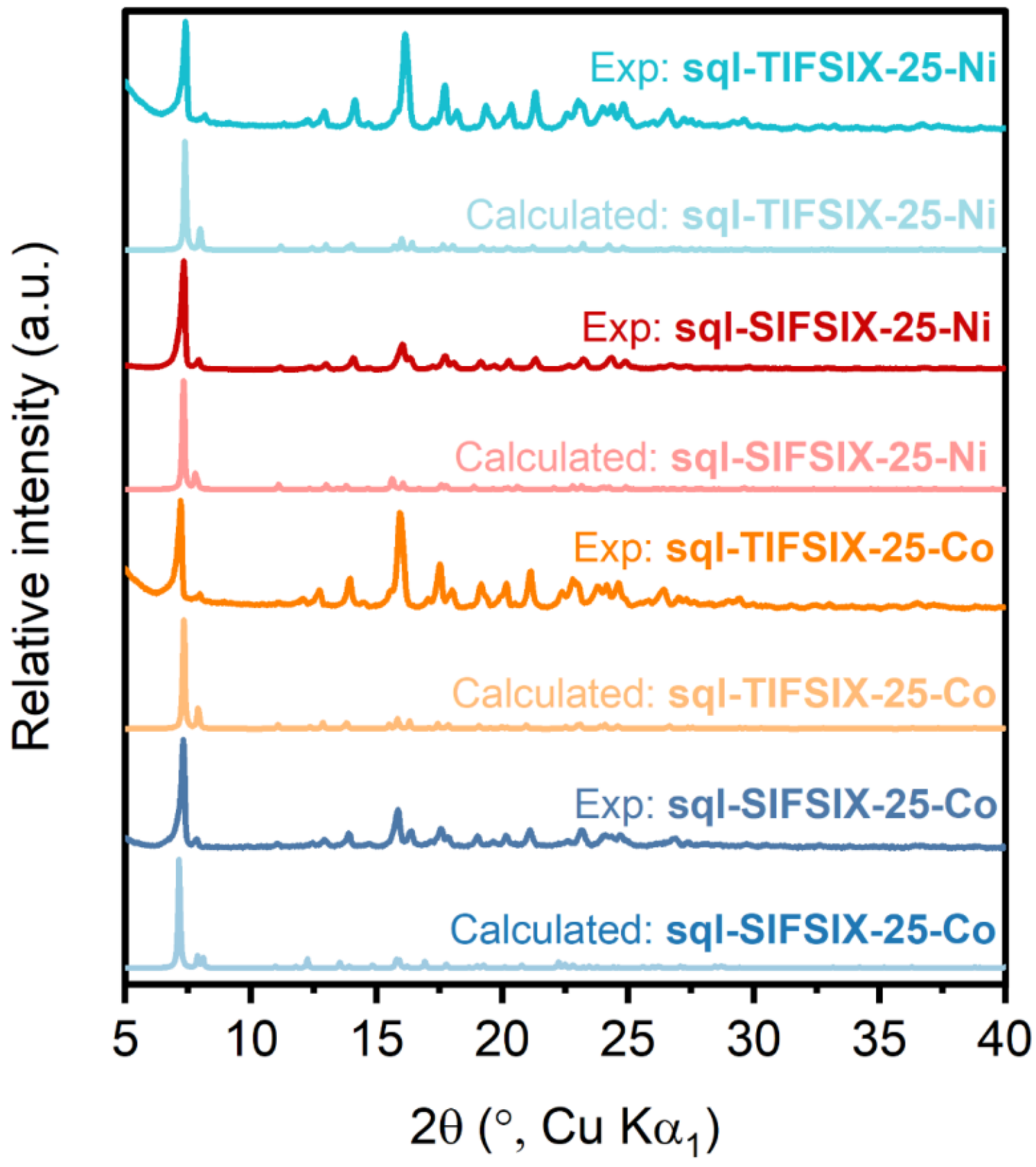

Figure S16 PXRD of as synthesized **sql-M'FSIX-25-M** (bottom to top): **sql-SIFSIX-25-Co**, **sql-TIFSIX-25-Co, sql-SIFSIX-25-Ni, sql-TIFSIX-25-Ni.** Experimental patterns collected at room temperature are shown alongside calculated patterns.

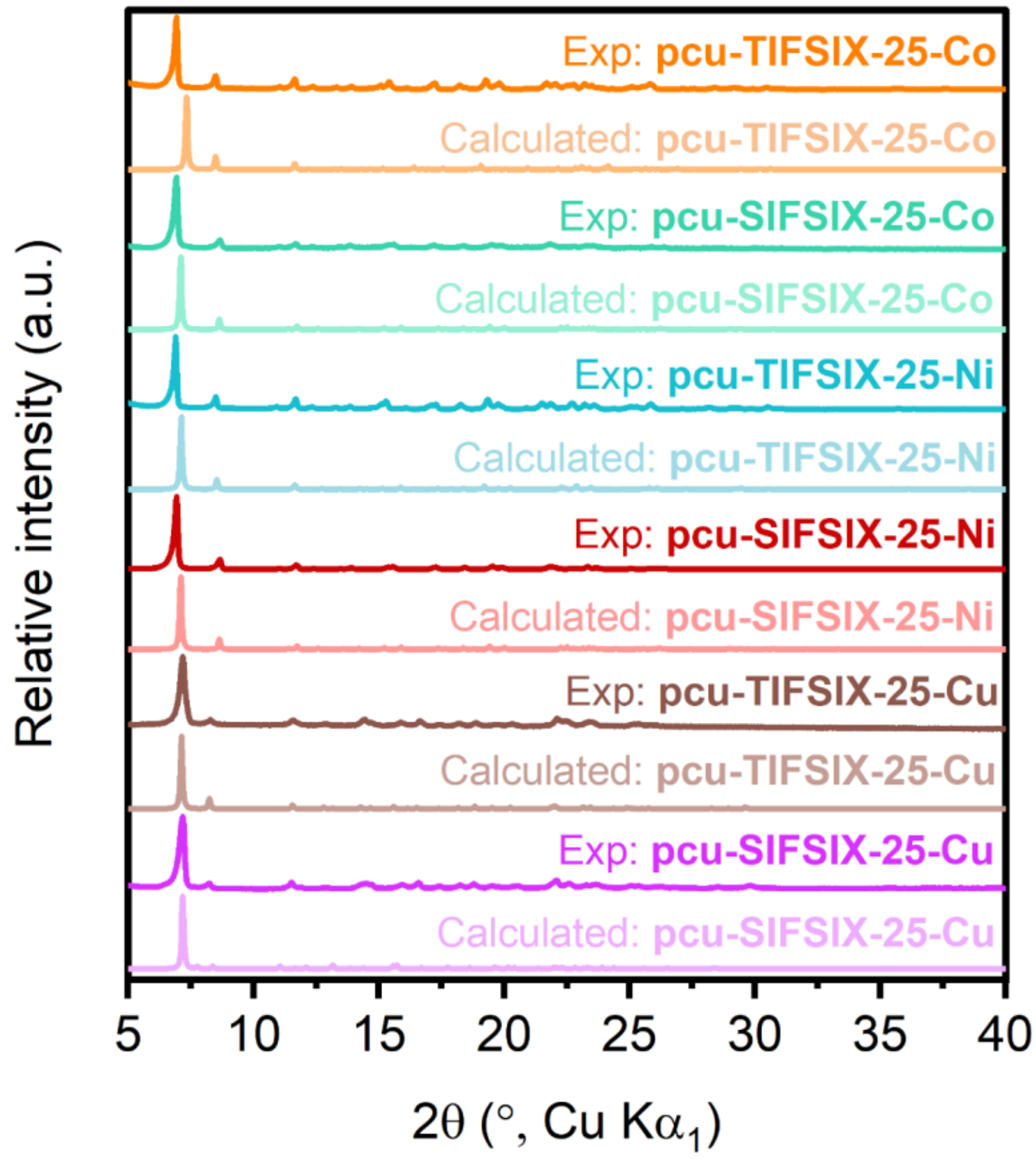

Figure S17 PXRD of **pcu-M'FSIX-25-M** HCNs (bottom to top): **pcu-SIFSIX-25-Cu**, **pcu-TIFSIX-25-Cu**, **pcu-SIFSIX-25-Ni**, **pcu-TIFSIX-25-Ni**, **pcu-SIFSIX-25-Co**, and **pcu-TIFSIX-25-Co**.

8 Thermogravimetric analysis

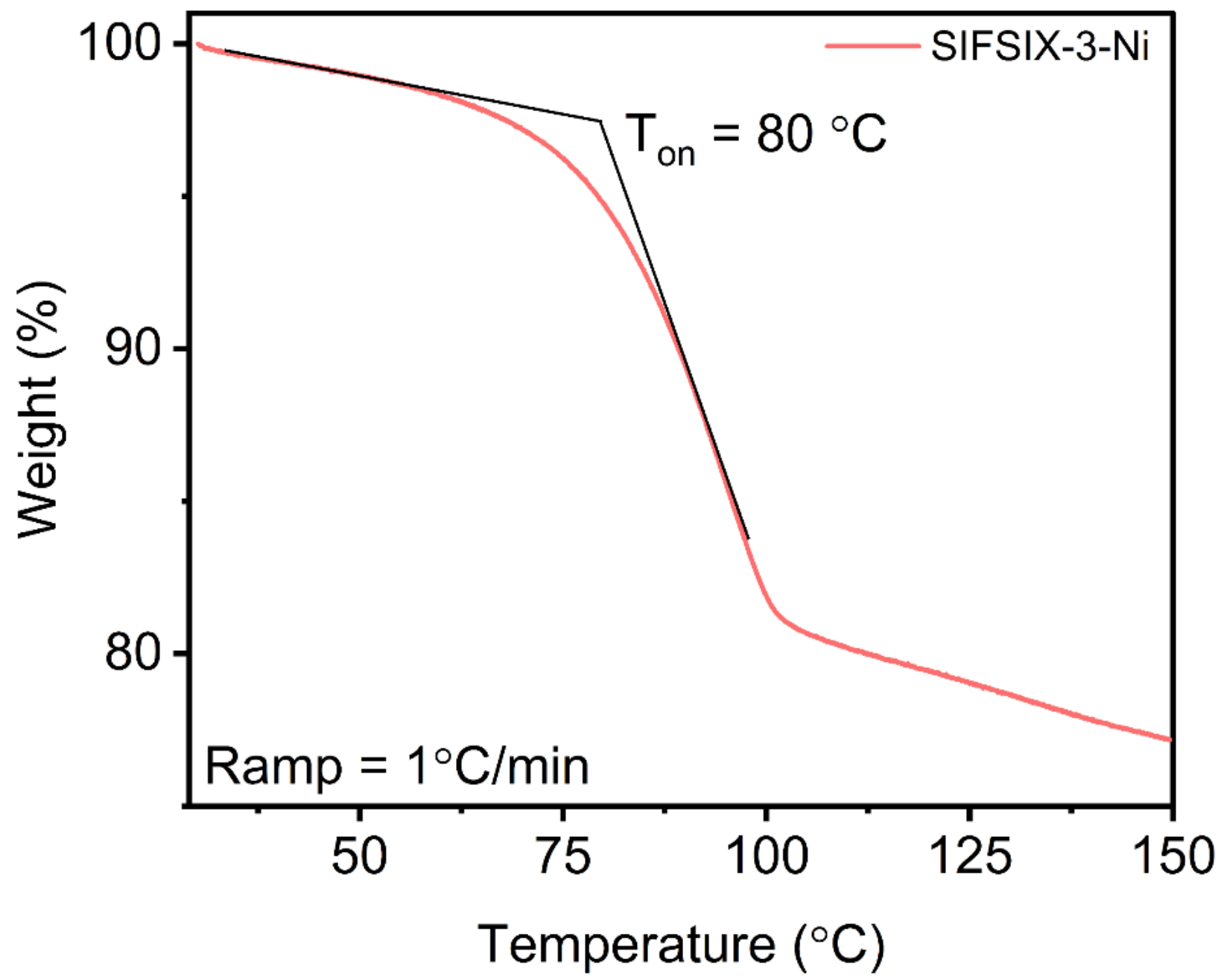

Figure S18 TGA profile of the as-synthesized **sql-SIFSIX-3-Ni** collected under a N_2 flow of 60 cm^3 min^{-1} at a heating rate of 1 °C min^{-1}. The sample exhibits an initial gradual mass loss followed by a pronounced weight decrease with an onset temperature (T_{on}) of ~80 °C, corresponding to the removal of lattice and coordinated water molecules from the framework.

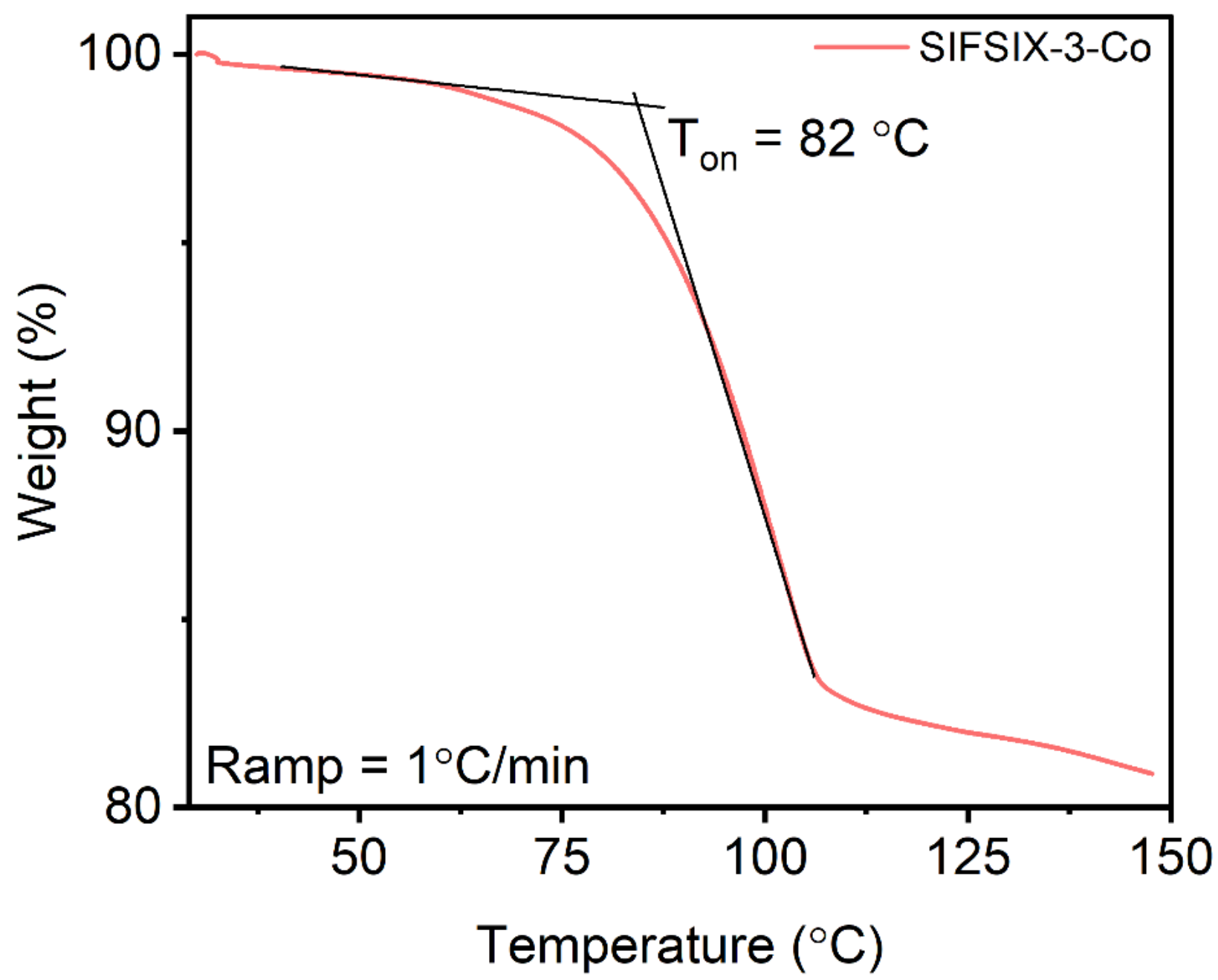

Figure S19 TGA profile of the as-synthesized **sql-SIFSIX-3-Co** collected under a N_2 flow of 60 cm^3 min^{-1} at a heating rate of 1 °C min^{-1}. The sample exhibits an initial gradual mass loss followed by a pronounced weight decrease with an onset temperature (T_{on}) of ~82 °C, corresponding to the removal of lattice and coordinated water molecules from the framework.

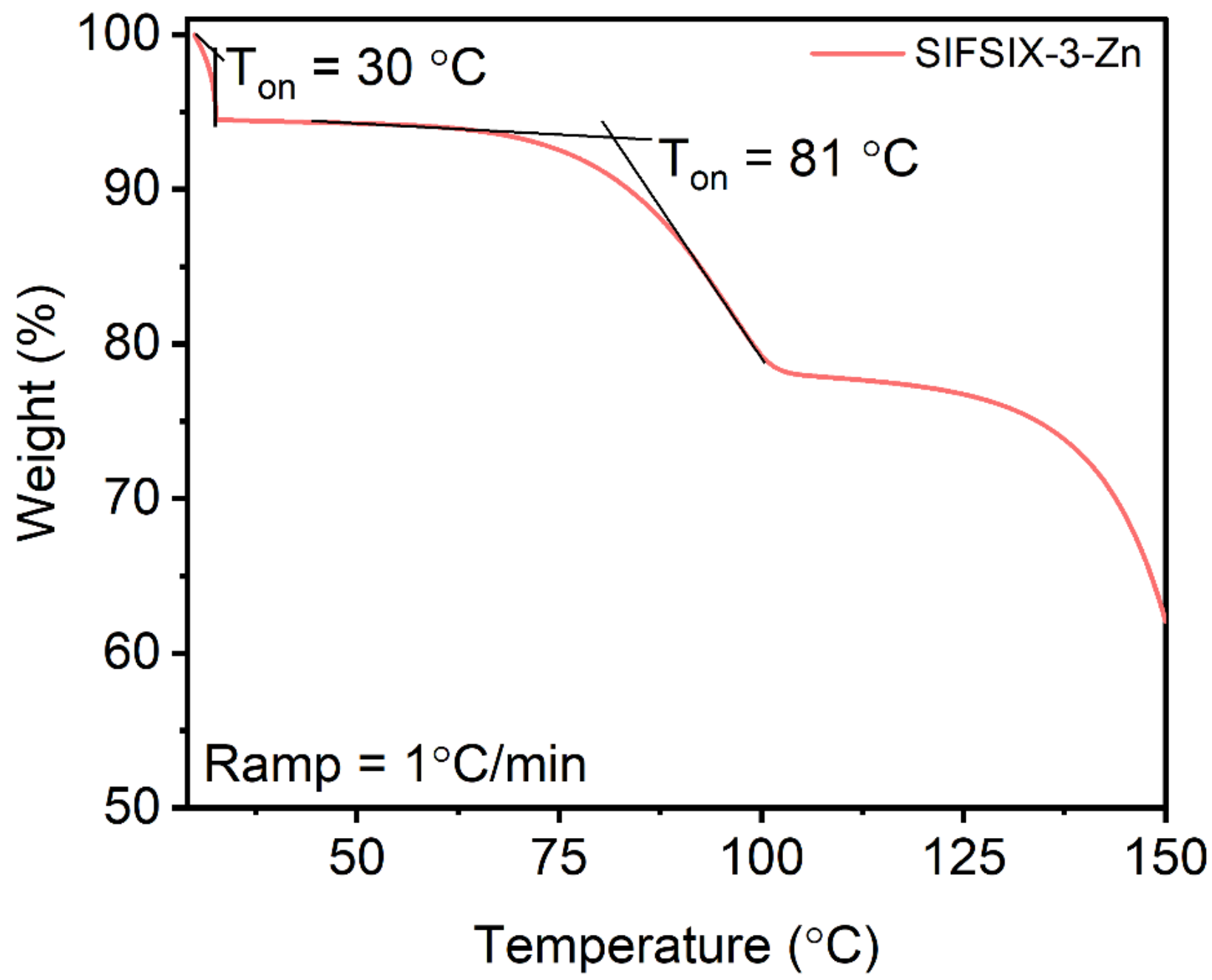

Figure S20 TGA profile of the as-synthesized **sql-SIFSIX-3-Zn** collected under a N_2 flow of 60 cm^3 min^{-1} at a heating rate of 1 °C min^{-1}. An initial weight loss at low temperature corresponds to the release of one lattice water molecule. This is followed by a major mass loss with an onset temperature (T_{on}) of ~81 °C, attributed to the removal of coordinated solvent molecules and framework desolvation.

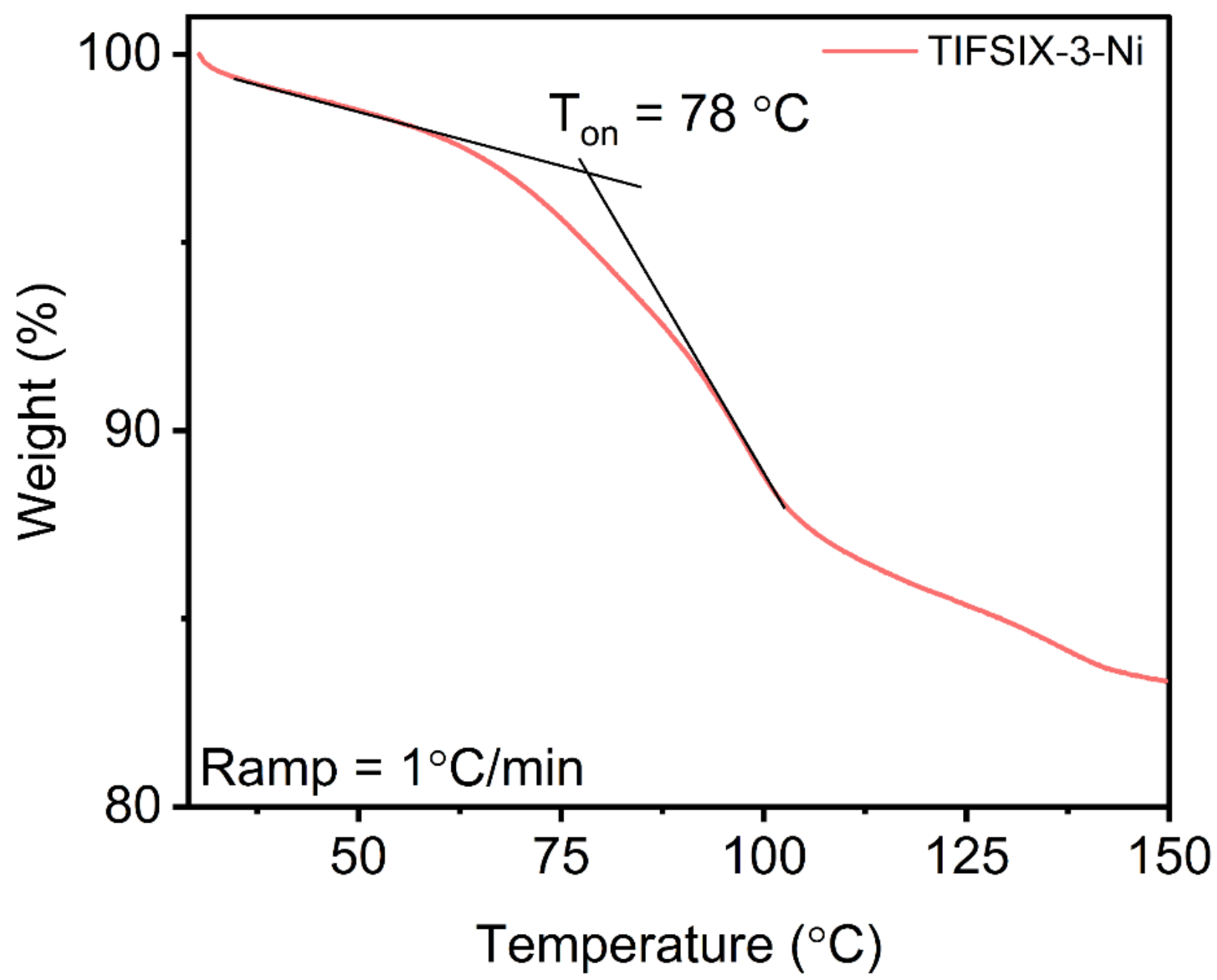

Figure S21 TGA profile of the as-synthesized **sql-TIFSIX-3-Ni** collected under a N_2 flow of 60 cm^3 min^{-1} at a heating rate of 1 °C min^{-1}. The sample exhibits an initial gradual mass loss followed by a pronounced weight decrease with an onset temperature (T_{on}) of ~78 °C, corresponding to the removal of lattice and coordinated water molecules from the framework.

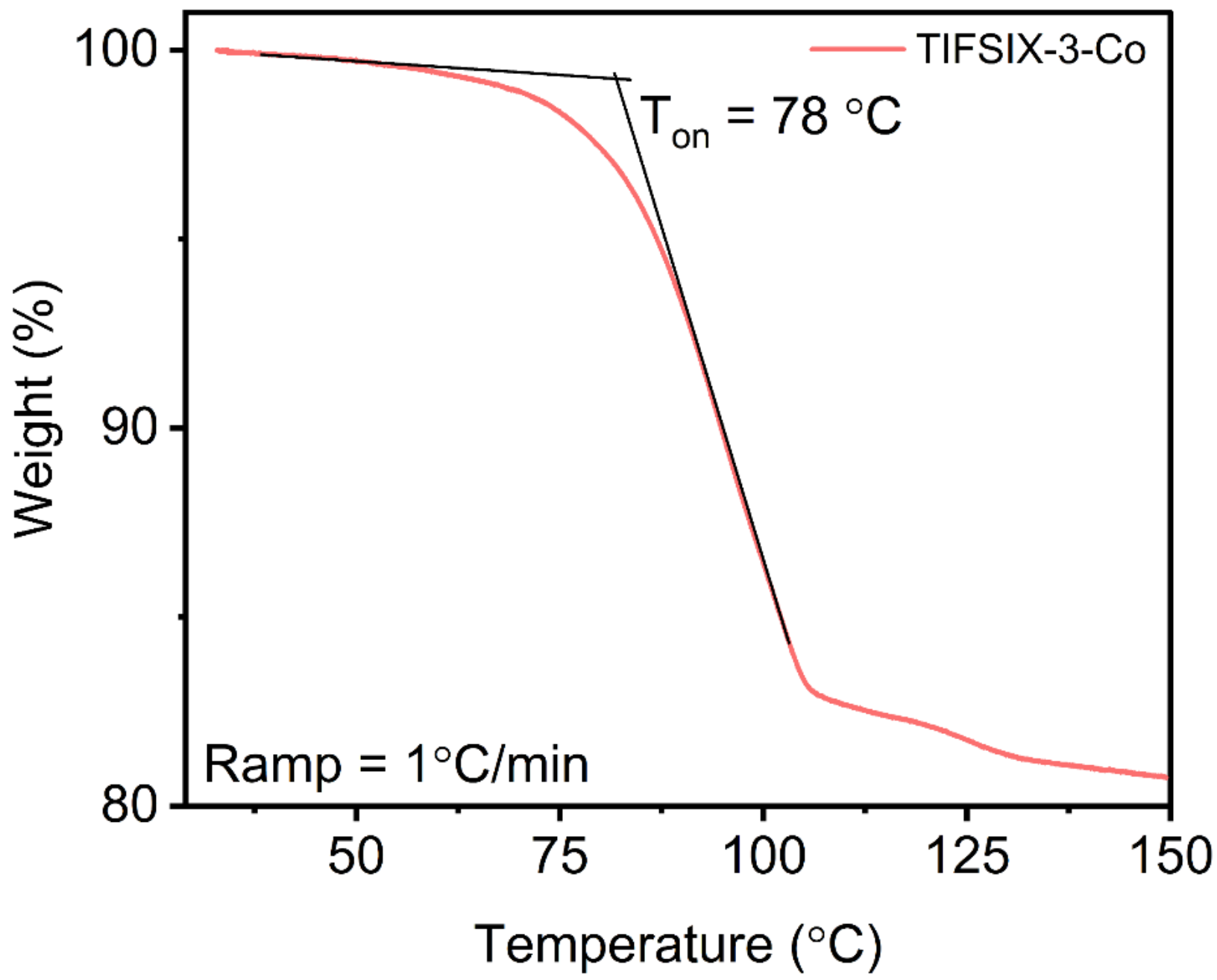

Figure S22 TGA profile of the as-synthesized **sql-TIFSIX-3-Co** collected under a N_2 flow of 60 cm^3 min^{-1} at a heating rate of 1 °C min^{-1}. The sample exhibits an initial gradual mass loss followed by a pronounced weight decrease with an onset temperature (T_{on}) of ~78 °C, corresponding to the removal of lattice and coordinated water molecules from the framework.

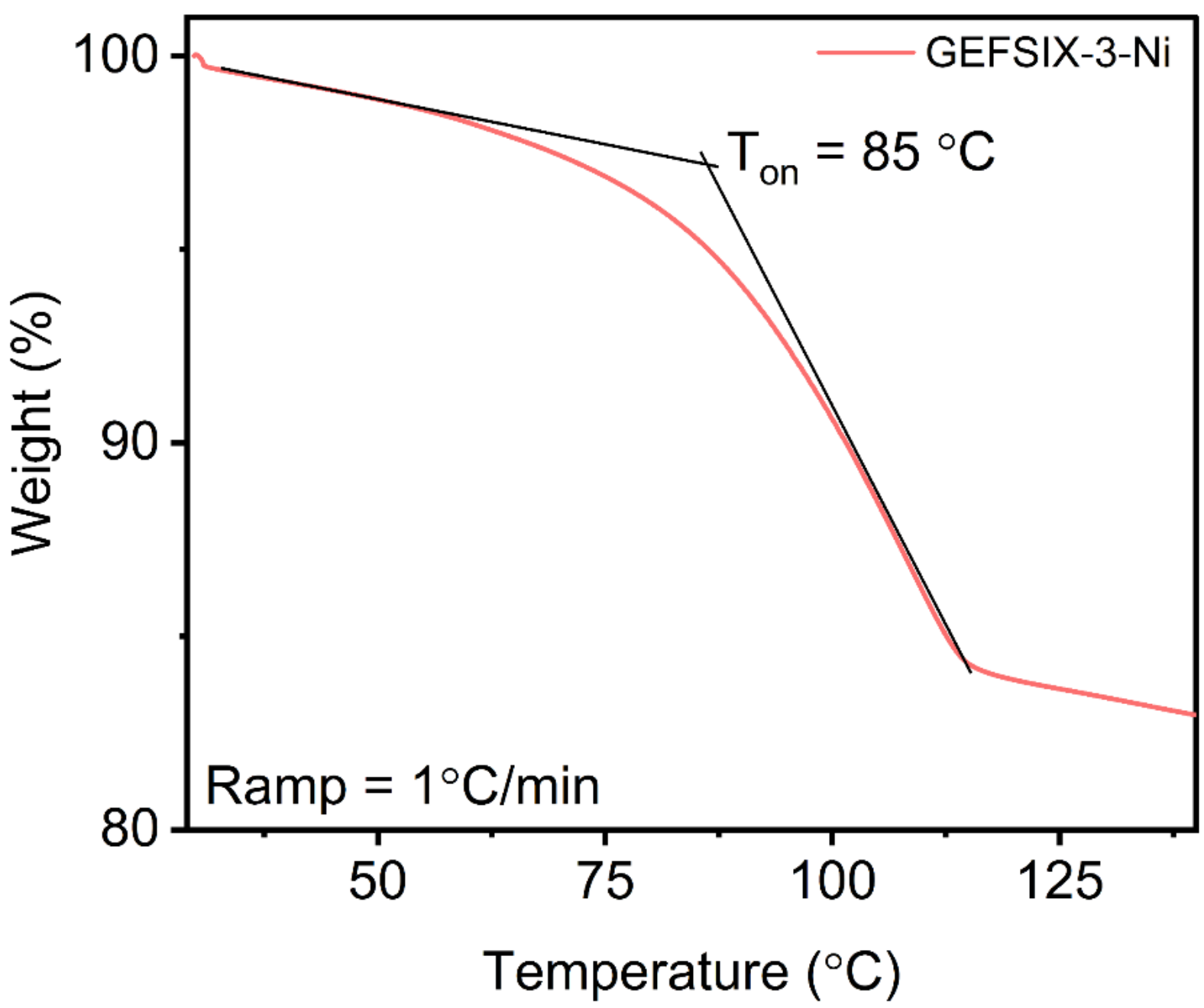

Figure S23 TGA profile of the as-synthesized **sql-GEFSIX-3-Ni** collected under a N_2 flow of 60 $cm^3\ min^{-1}$ at a heating rate of 1 °C min^{-1}. The sample exhibits an initial gradual mass loss followed by a pronounced weight decrease with an onset temperature (T_{on}) of ~85 °C, corresponding to the removal of lattice and coordinated water molecules from the framework.

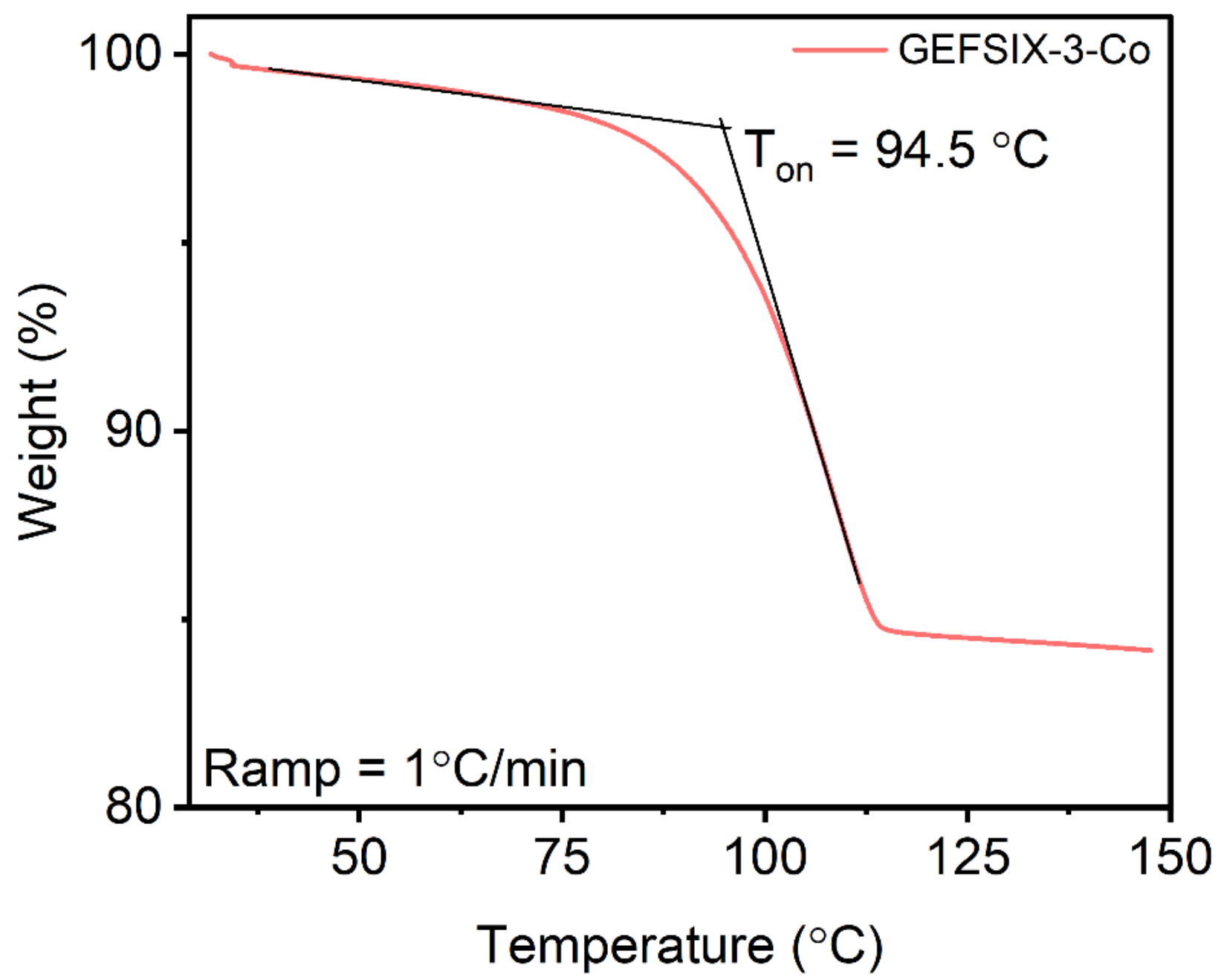

Figure S24 TGA profile of the as-synthesized **sql-GEFSIX-3-Co** collected under a N_2 flow of 60 cm^3 min^{-1} at a heating rate of 1 °C min^{-1}. The sample exhibits an initial gradual mass loss followed by a pronounced weight decrease with an onset temperature (T_{on}) of ~94 °C, corresponding to the removal of lattice and coordinated water molecules from the framework.

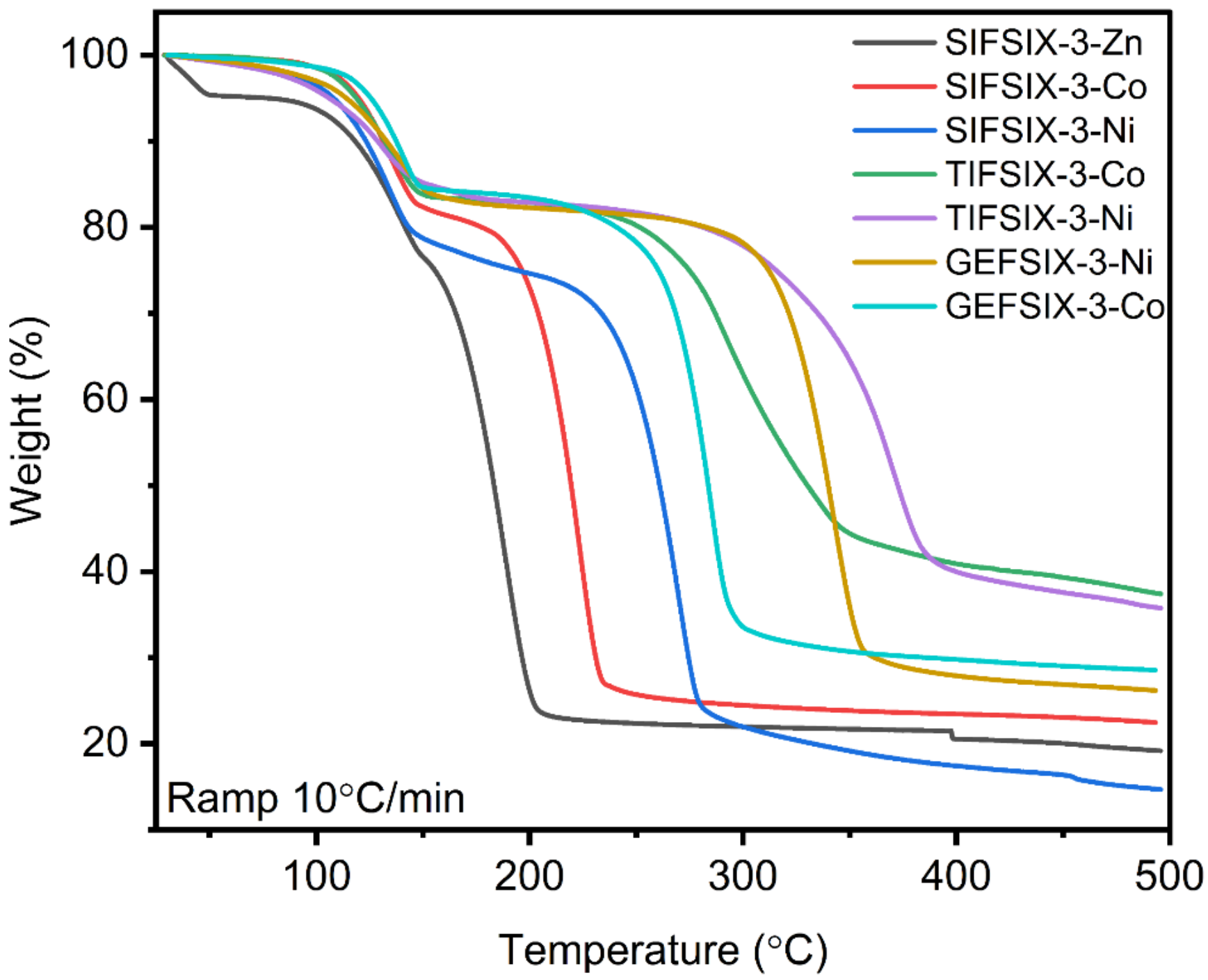

Figure S25 Comparative TGA profiles of as-synthesized **sql-M'FSIX-3-M** recorded under N_2 atmosphere at a heating rate of 10 °C min^{-1}.

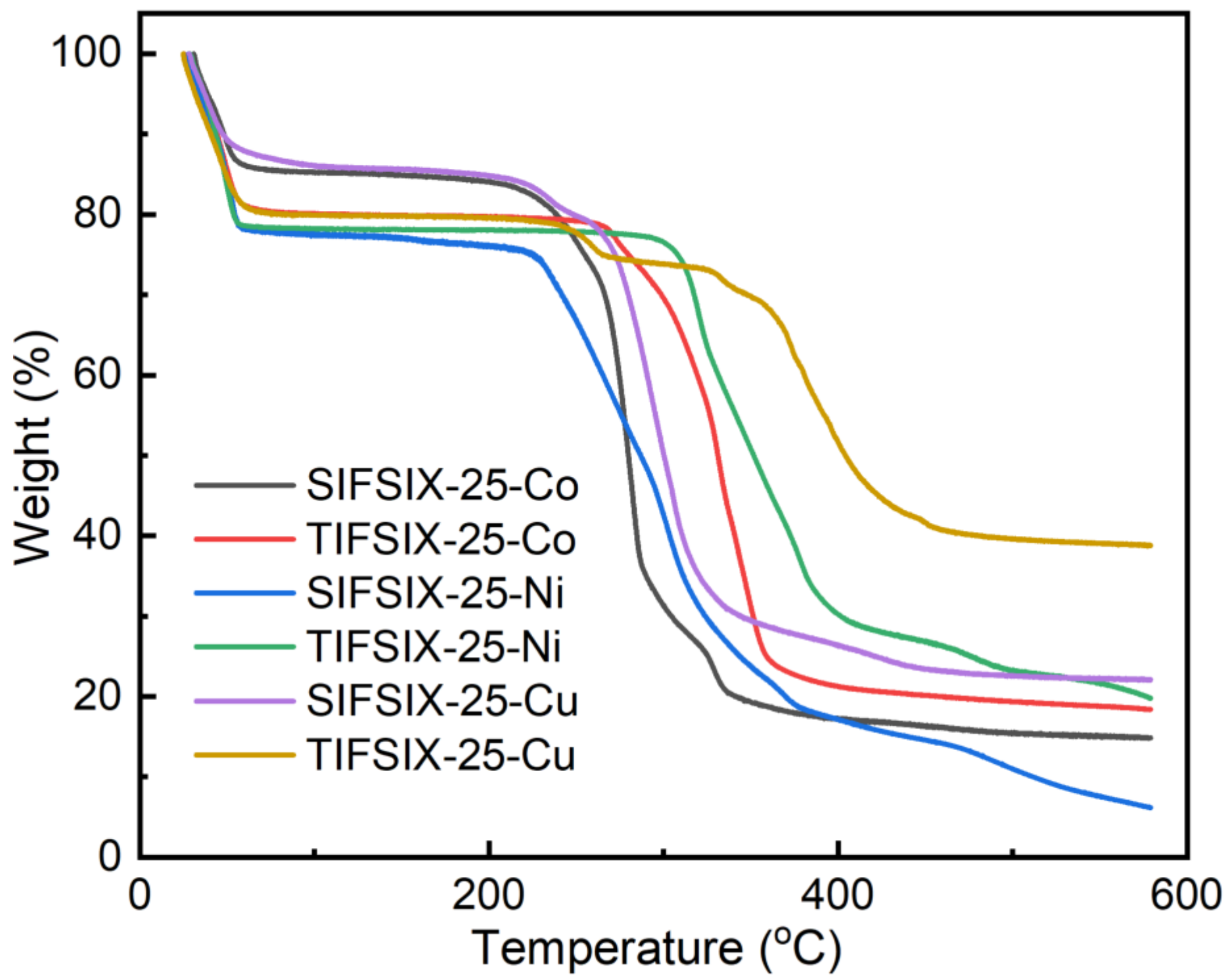

Figure S26 Comparative TGA profiles of as-synthesized **M'FSIX-25-M** compounds recorded at a heating rate of 10 °C min^{-1}.

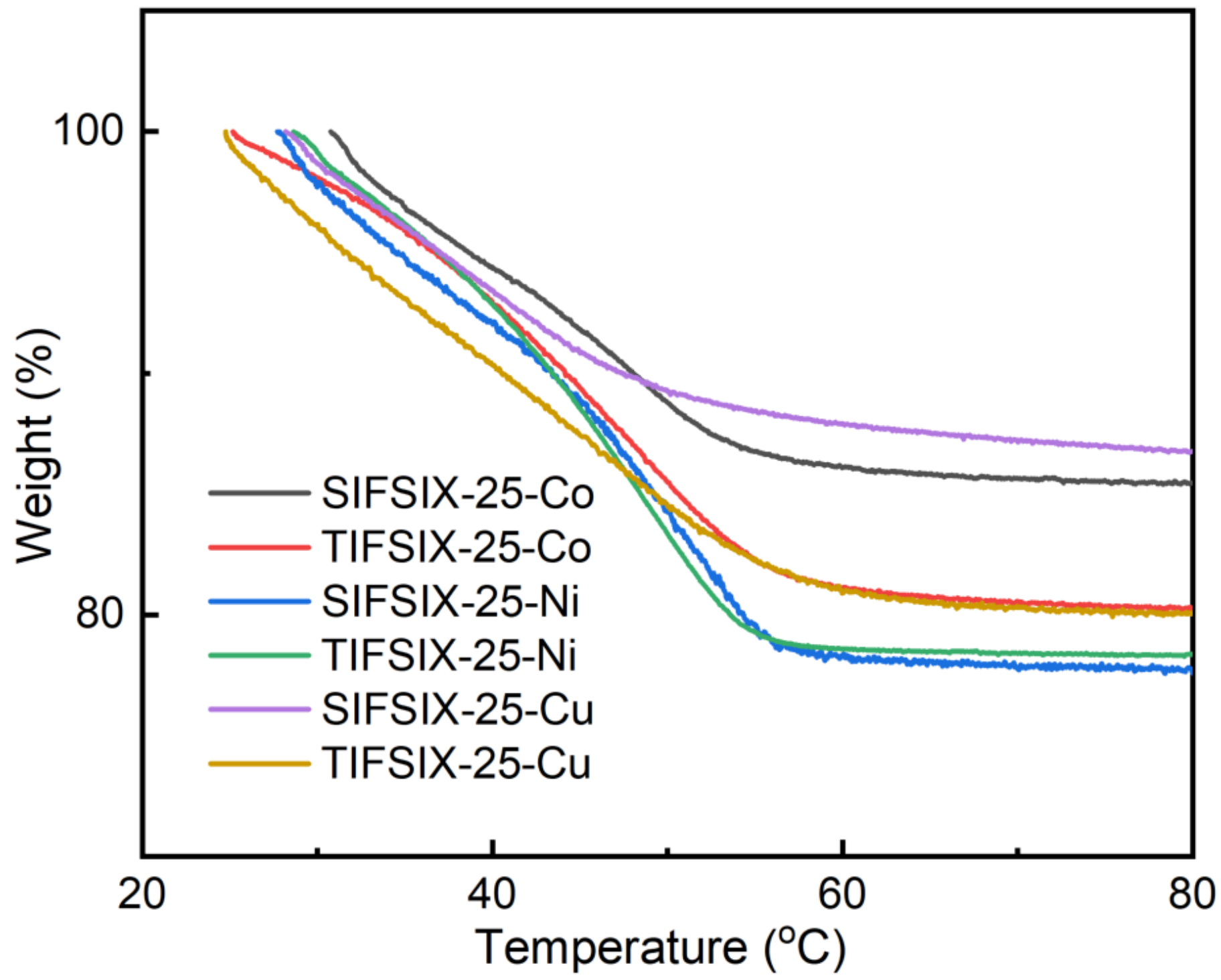

Figure S27 Comparative TGA profiles of as-synthesized **M'FSIX-25-M** compounds recorded at a heating rate of 10 °C min^{-1}.

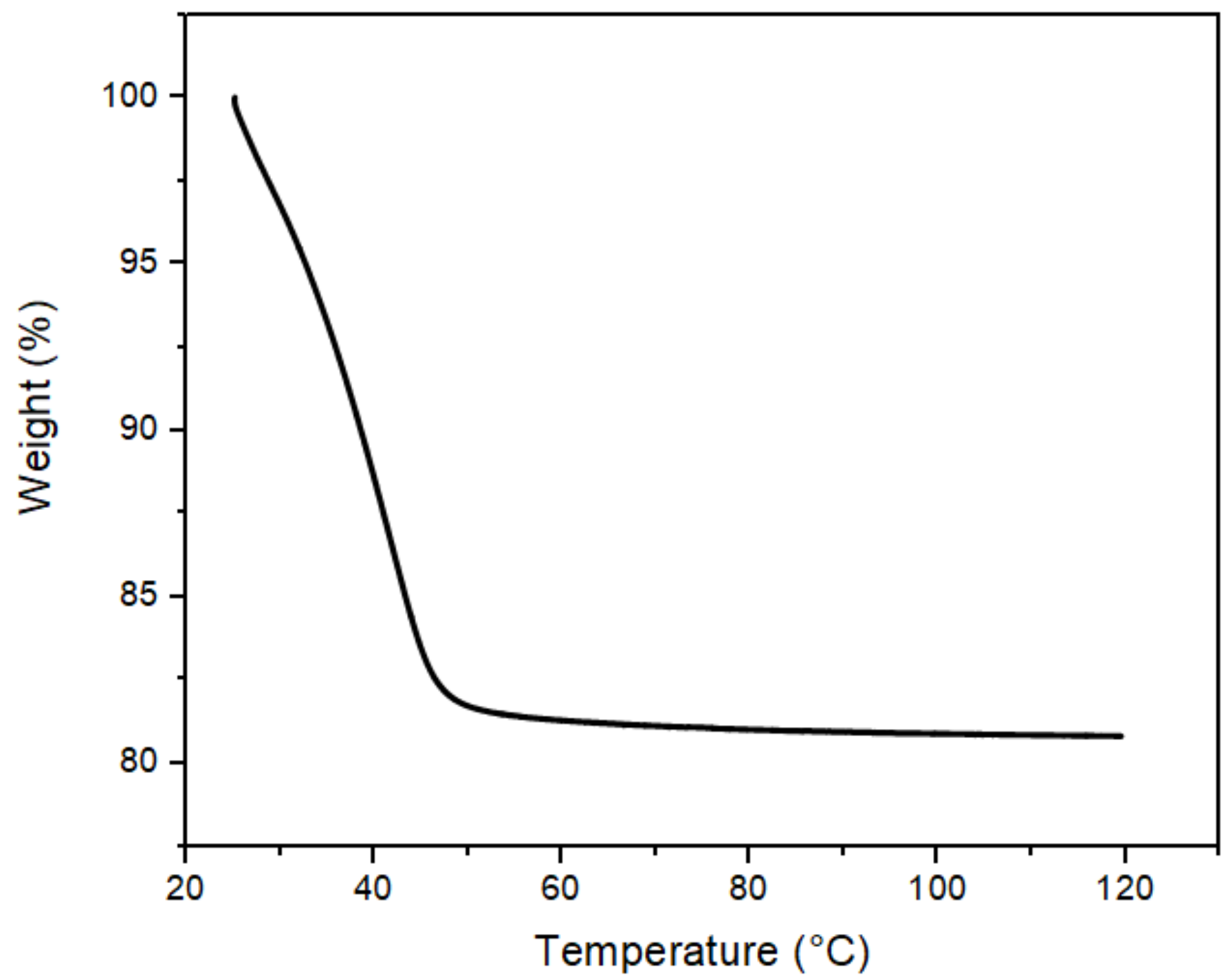

Figure S28 TGA thermogram of **TIFSIX-25-Ni** during a temperature-ramp sequence carried out at 1 °C min^{-1} from room temperature.

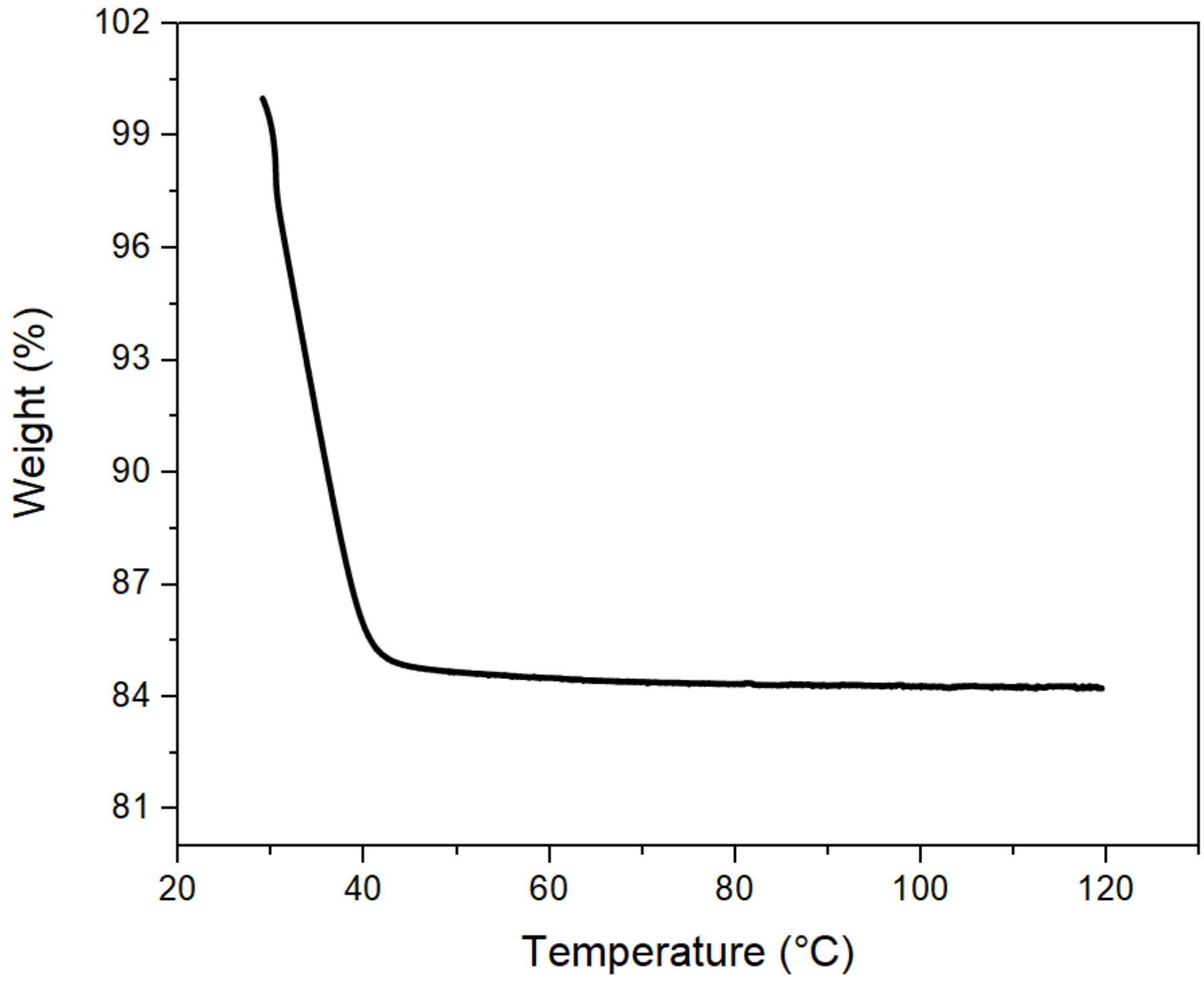

Figure S29 TGA thermogram of **TIFSIX-25-Co** during a temperature-ramp sequence carried out at 1 °C min^{-1} from room temperature.

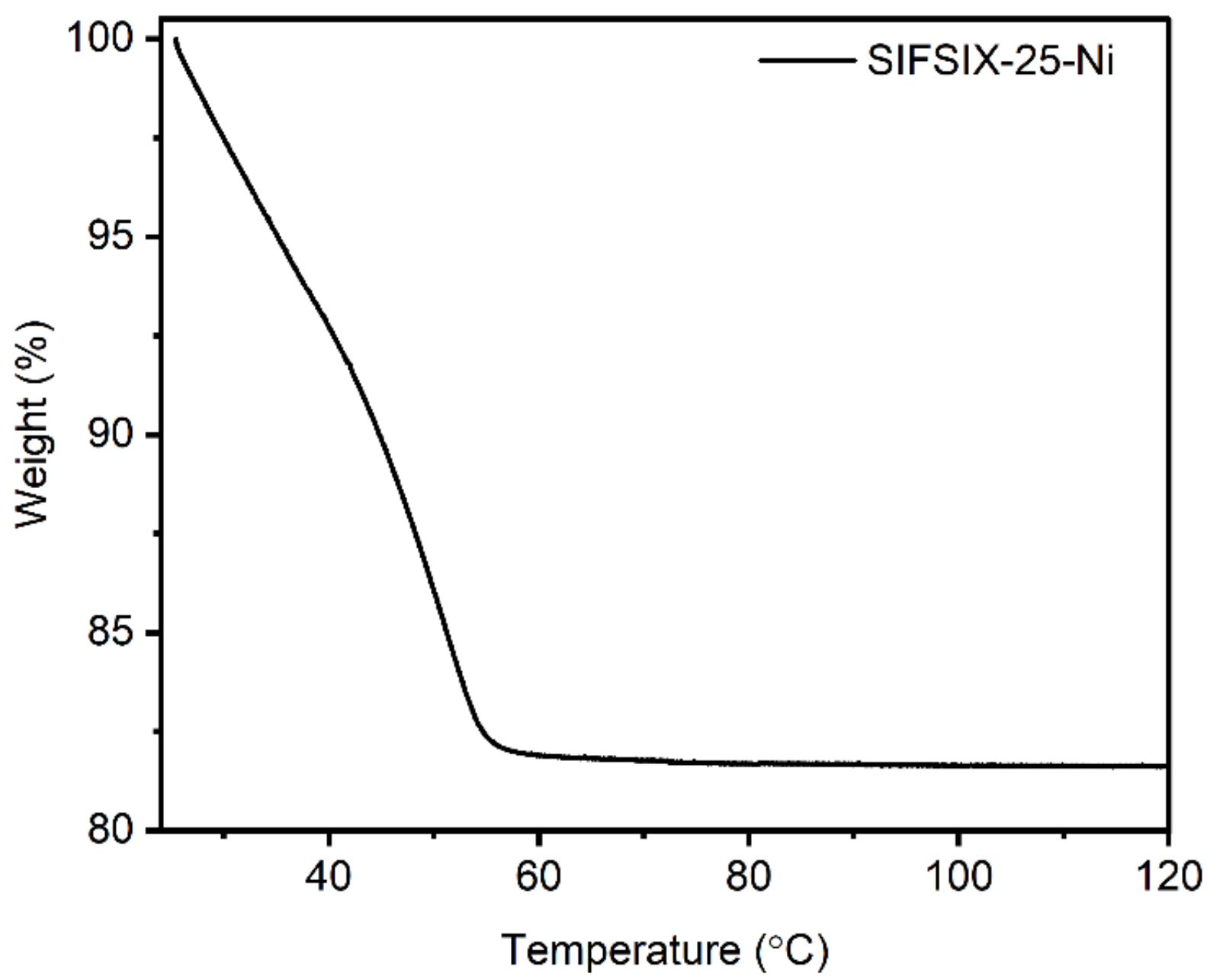

Figure S230 TGA thermogram of **SIFSIX-25-Ni** during a temperature-ramp sequence carried out at 1 °C min^{-1} from room temperature.

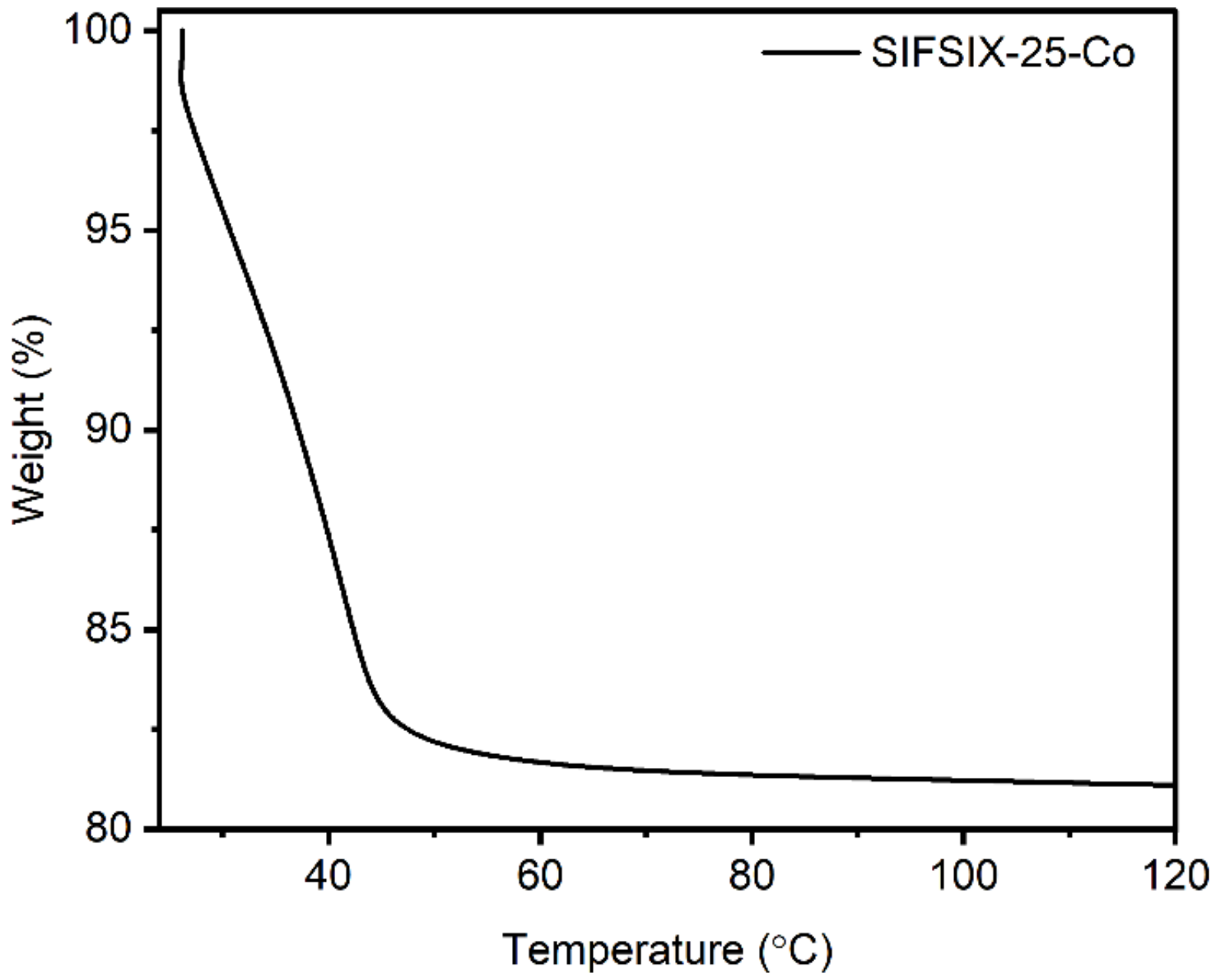

Figure S31 TGA thermogram of **SIFSIX-25-Co** during a temperature-ramp sequence carried out at 1 °C min^{-1} from room temperature.

9 Variable-temperature powder X-ray diffraction

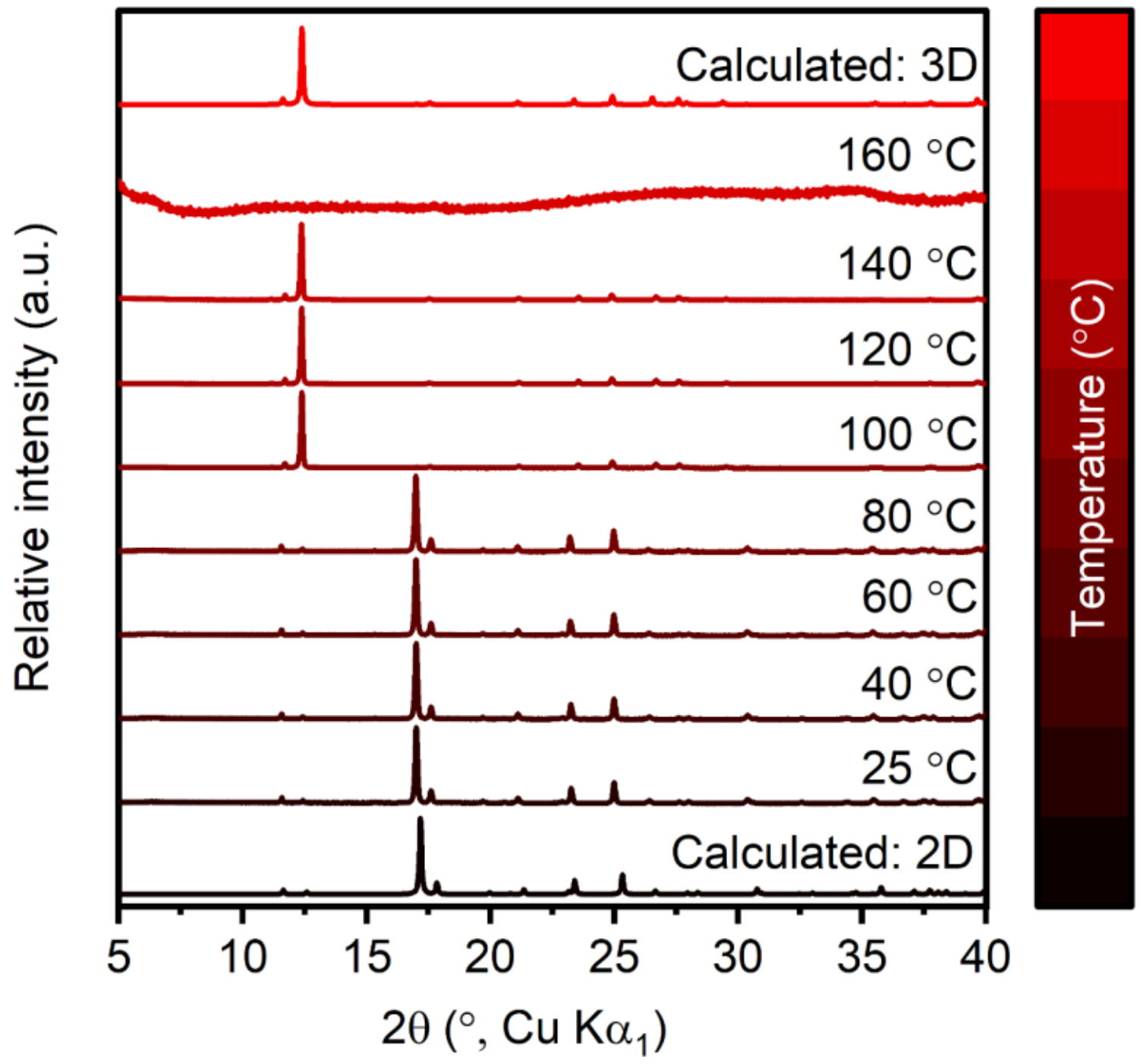

Figure S32 Variable-temperature PXRD patterns collected under a N_2 flow of 20 cm^3 min^{-1} showing the structural transformation from the as-synthesized 2D **sql** phase (**sql-SIFSIX-3-Zn**) to the 3D phase (**pcu-SIFSIX-3-Zn)** upon heating (25–120 °C). Experimental patterns are compared with simulated PXRD patterns calculated from crystal structures corresponding to the **sql-SIFSIX-3-Ni** (CSD refcode **POCREI01**) and the **pcu-SIFSIX-3-Zn** phase (CSD refcode **FUDQIF**). Loss of crystallinity observed at 160 °C indicates framework degradation, consistent with the TGA data. Patterns are vertically offset for clarity.

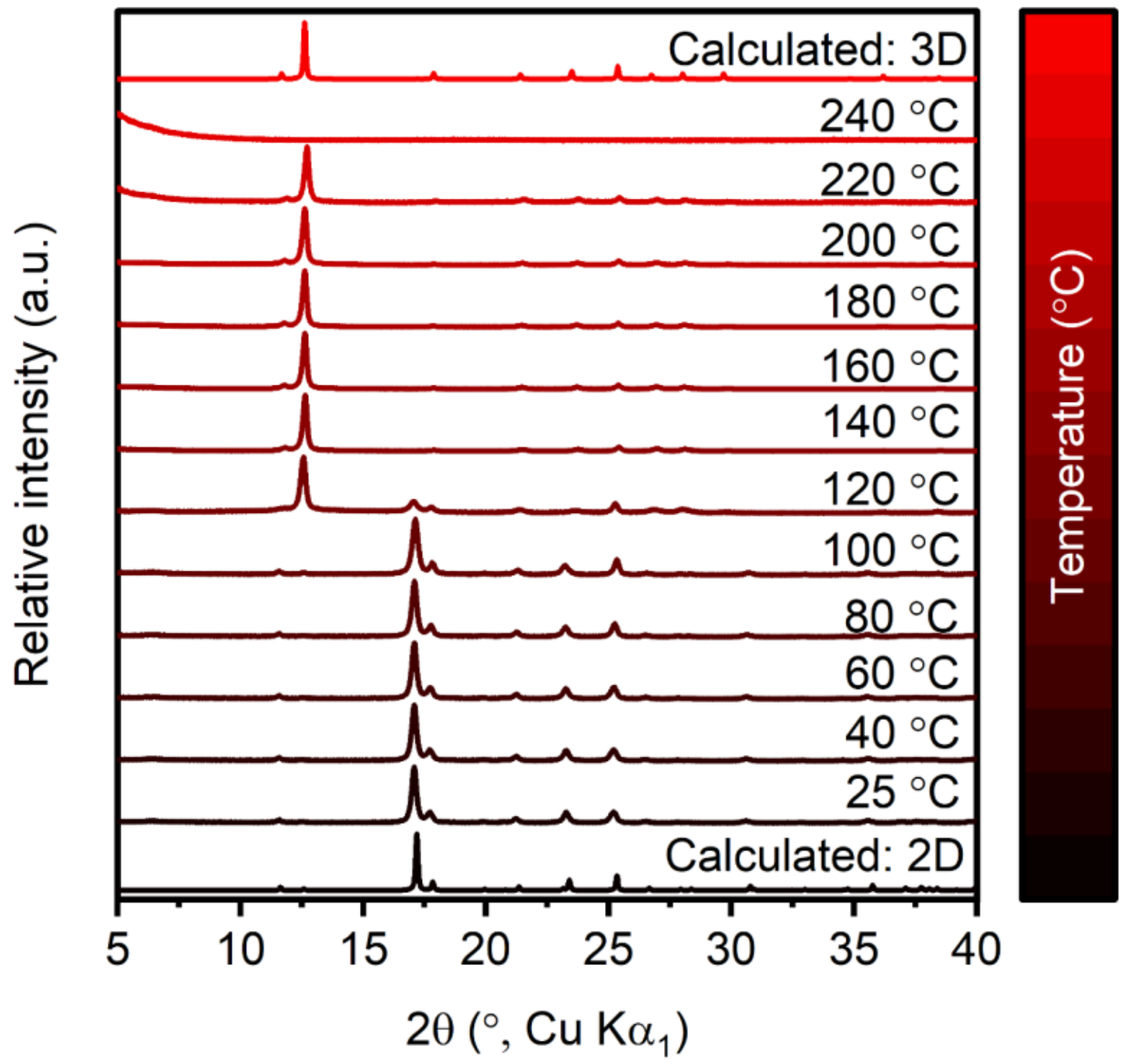

Figure S33 Variable-temperature PXRD patterns collected under a N_2 flow of 20 cm^3 min^{-1} showing the structural transformation from the as-synthesized 2D **sql** phase (**sql-SIFSIX-3-Ni**) to the 3D phase (**pcu-SIFSIX-3-Ni)** upon heating (25–120 °C). Experimental patterns are compared with simulated PXRD patterns calculated from crystal structures corresponding to the **sql-SIFSIX-3-Ni** (CSD refcode **POCREI01**) and the **pcu-SIFSIX-3-Ni** phase (CSD refcode **DUMFEY**). Loss of crystallinity observed at 240 °C indicates framework degradation, consistent with the TGA data. Patterns are vertically offset for clarity.

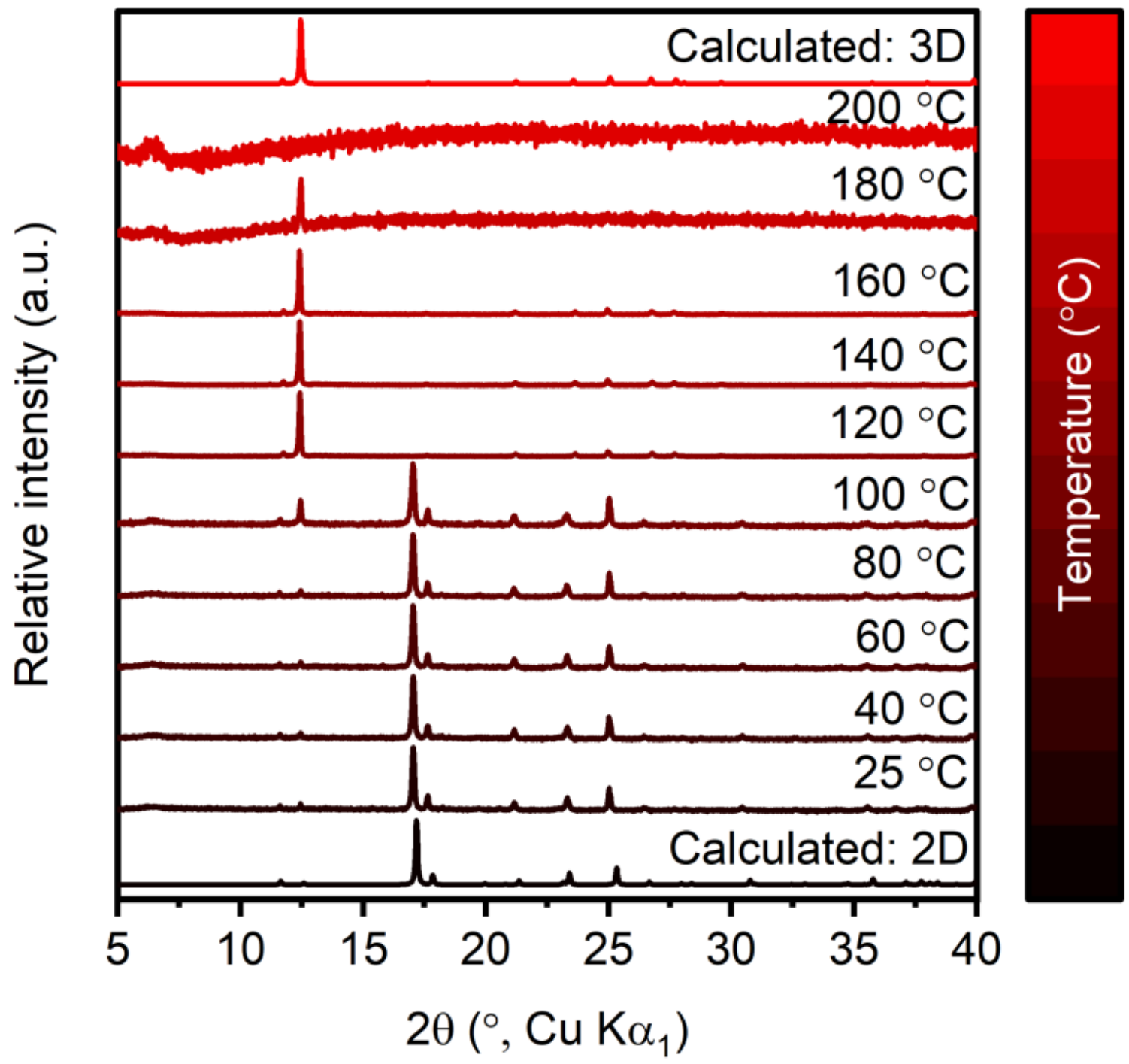

Figure S34 Variable-temperature PXRD patterns collected under a N_2 flow of 20 cm^3 min^{-1} showing the structural transformation from the as-synthesized 2D **sql** phase (**sql-SIFSIX-3-Co**) to the 3D phase (**pcu-SIFSIX-3-Co)** upon heating (25–120 °C). Experimental patterns are compared with simulated PXRD patterns calculated from crystal structures corresponding to the **sql-SIFSIX-3-Ni** (CSD refcode **POCREI01**) and the **pcu-SIFSIX-3-Co** phase (from SCXRD data of this work). Loss of crystallinity observed at 200 °C indicates framework degradation, consistent with the TGA data. Patterns are vertically offset for clarity.

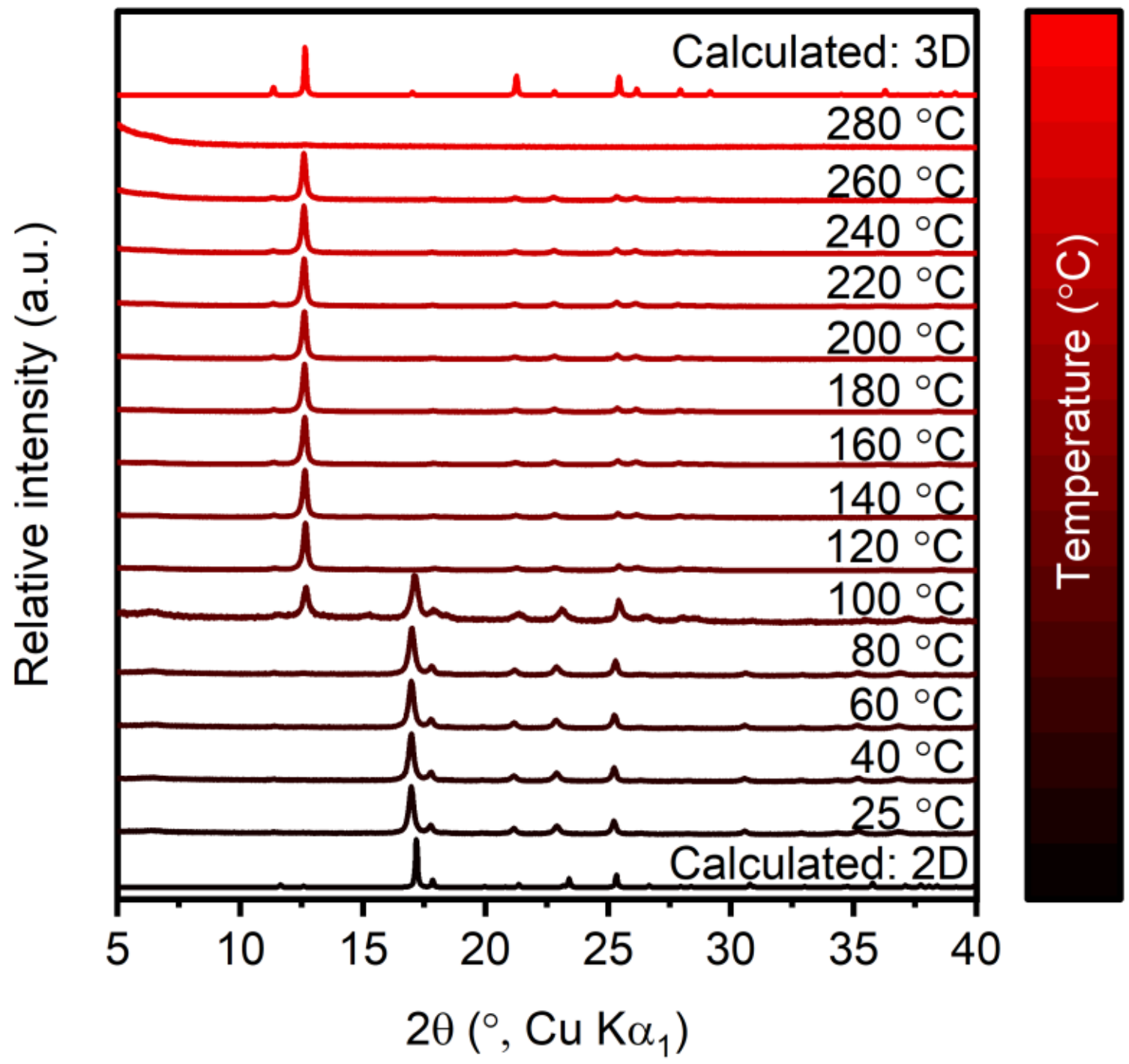

Figure S35 Variable-temperature PXRD patterns collected under a N_2 flow of 20 cm^3 min^{-1} showing the structural transformation from the as-synthesized 2D **sql** phase (**sql-TIFSIX-3-Ni**) to the 3D phase (**pcu- TIFSIX-3-Ni)** upon heating (25–140 °C). Experimental patterns are compared with simulated PXRD patterns calculated from crystal structures corresponding to the **sql-SIFSIX-3-Ni** (CSD refcode **POCREI01**) and the **pcu- TIFSIX-3-Ni** phase (CSD refcode **RIJGIE**). Loss of crystallinity observed at 280 °C indicates framework degradation, consistent with the TGA data. Patterns are vertically offset for clarity.

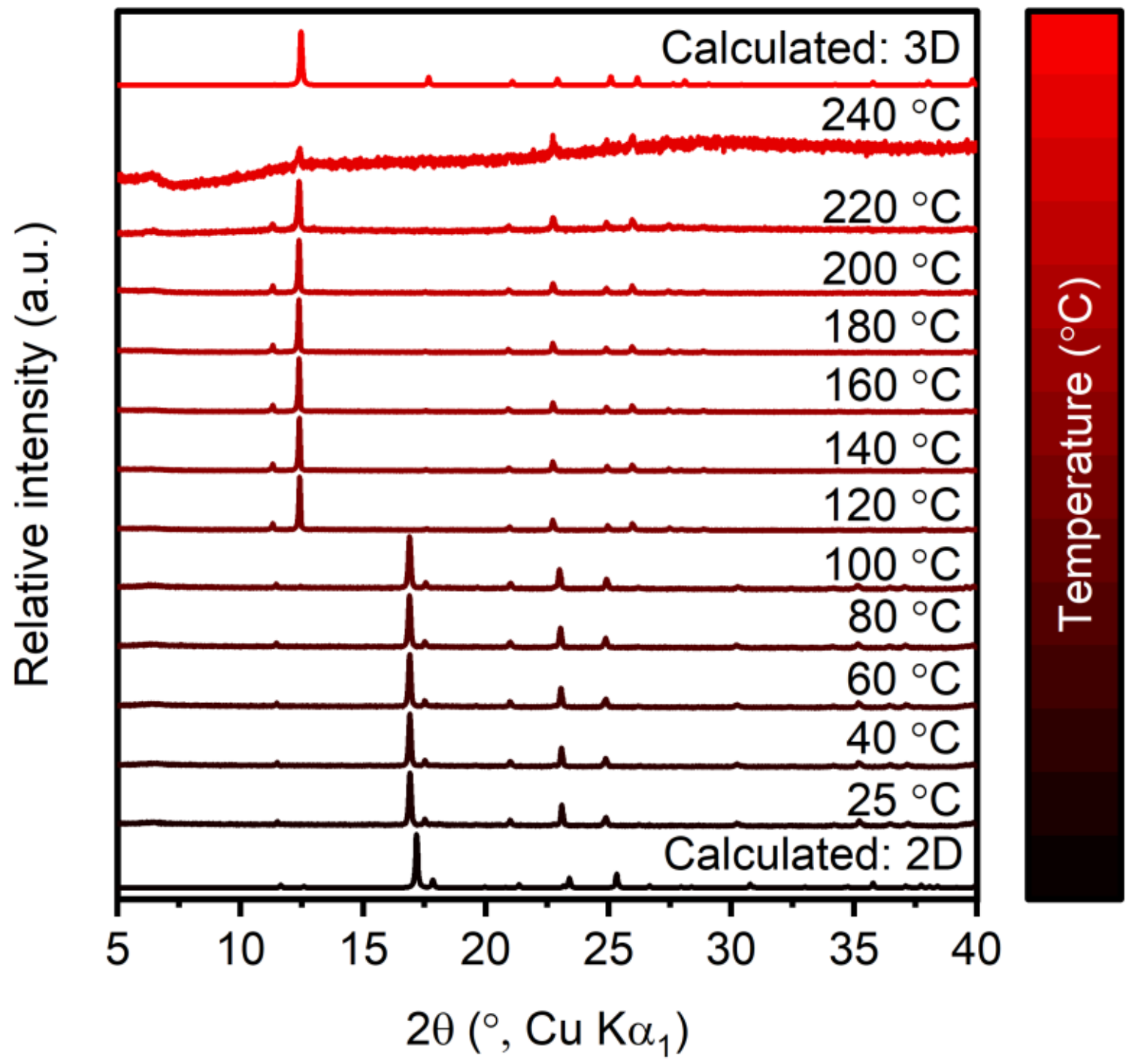

Figure S36 Variable-temperature PXRD patterns collected under a N_2 flow of 20 cm^3 min^{-1} showing the structural transformation from the as-synthesized 2D **sql** phase (**sql-TIFSIX-3-Co**) to the 3D phase (**pcu- TIFSIX-3-Co)** upon heating (25–120 °C). Experimental patterns are compared with simulated PXRD patterns calculated from crystal structures corresponding to the **sql-SIFSIX-3-Ni** (CSD refcode **POCREI01**) and the **pcu-TIFSIX-3-Co** phase (CSD refcode **SUHQUK**). Loss of crystallinity observed at 280 °C indicates framework degradation, consistent with the TGA data. Patterns are vertically offset for clarity.

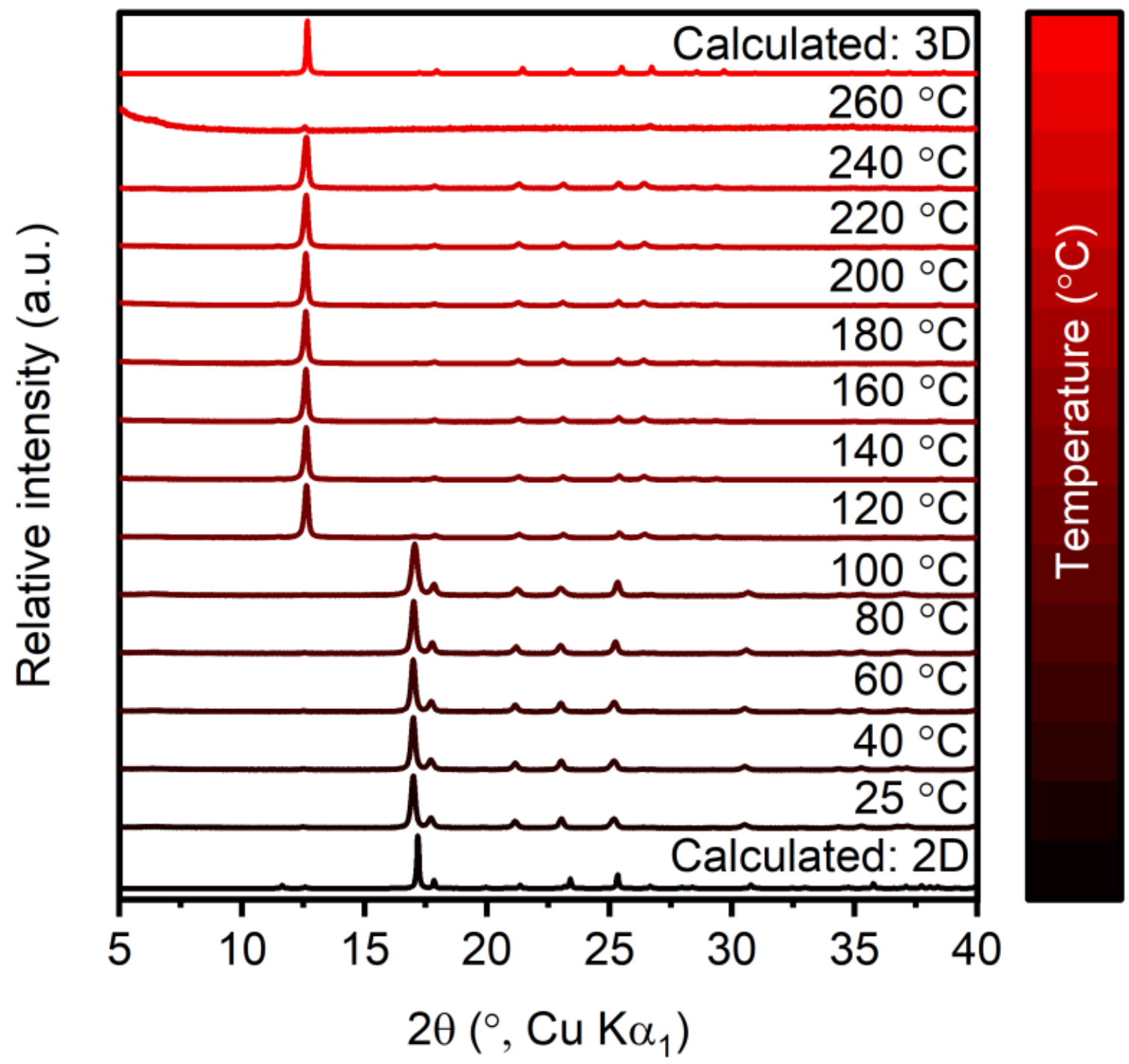

Figure S37 Variable-temperature PXRD patterns collected under a N_2 flow of 20 cm^3 min^{-1} showing the structural transformation from the as-synthesized 2D **sql** phase (**sql-GEFSIX-3-Ni**) to the 3D phase (**pcu- GEFSIX-3-Ni)** upon heating (25–100 °C). Experimental patterns are compared with simulated PXRD patterns calculated from crystal structures corresponding to the **sql-SIFSIX-3-Ni** (CSD refcode **POCREI01**) and the **pcu-GEFSIX-3-Ni** phase (CSD refcode **ZUTVAO**). Loss of crystallinity observed at 260 °C indicates framework degradation, consistent with the TGA data. Patterns are vertically offset for clarity.

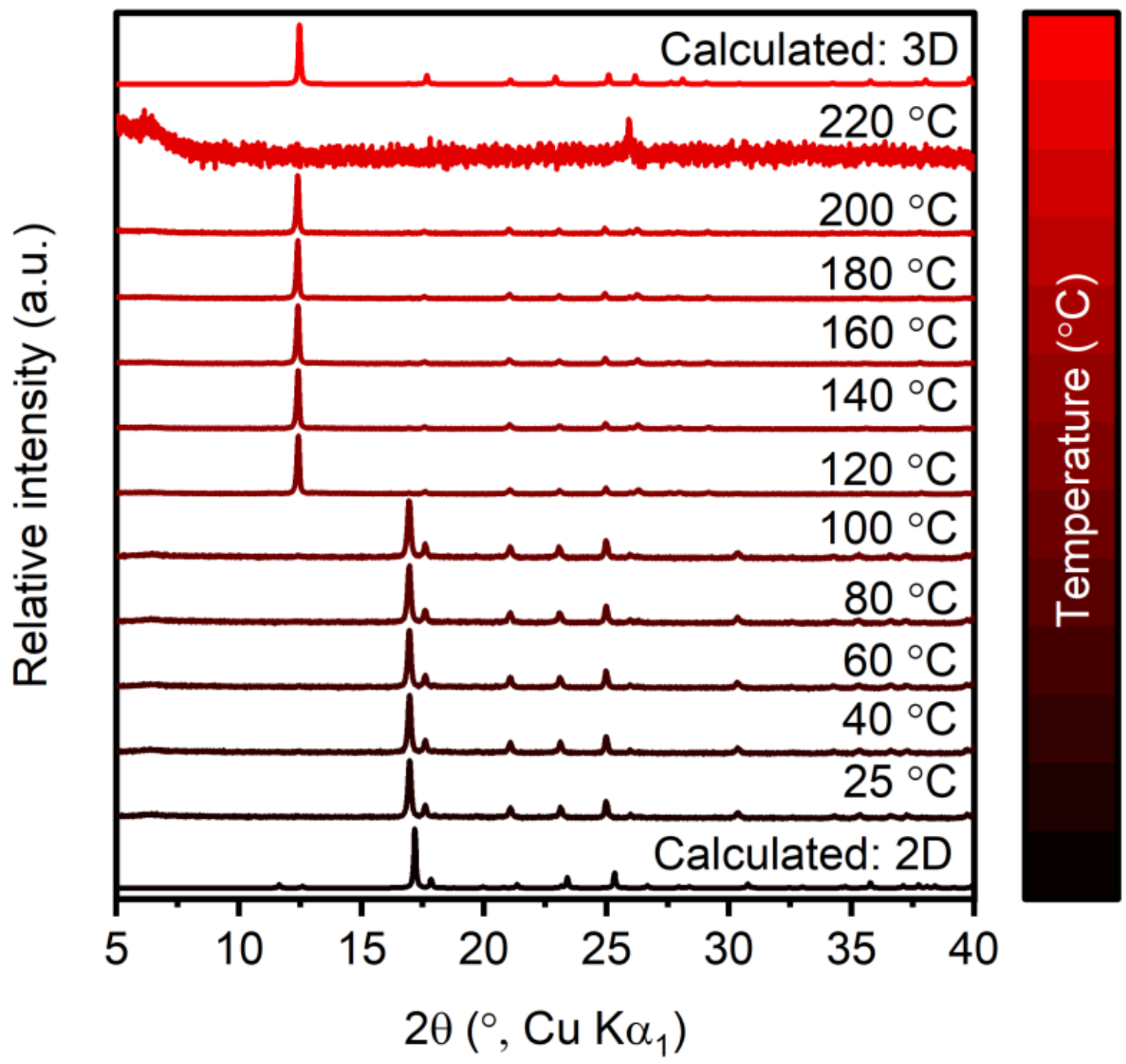

Figure S38 Variable-temperature PXRD patterns collected under a N_2 flow of 20 cm^3 min^{-1} showing the structural transformation from the as-synthesized 2D **sql** phase (**sql-GEFSIX-3-Co**) to the 3D phase (**pcu-GEFSIX-3-Co)** upon heating (25–100 °C). Experimental patterns are compared with simulated PXRD patterns calculated from crystal structures corresponding to the **sql-SIFSIX-3-Ni** (CSD refcode **POCREI01**) and the **pcu-GEFSIX-3-Co** phase (CSD refcode **HOYXIF**). Loss of crystallinity observed at 220 °C indicates framework degradation, consistent with the TGA data. Patterns are vertically offset for clarity.

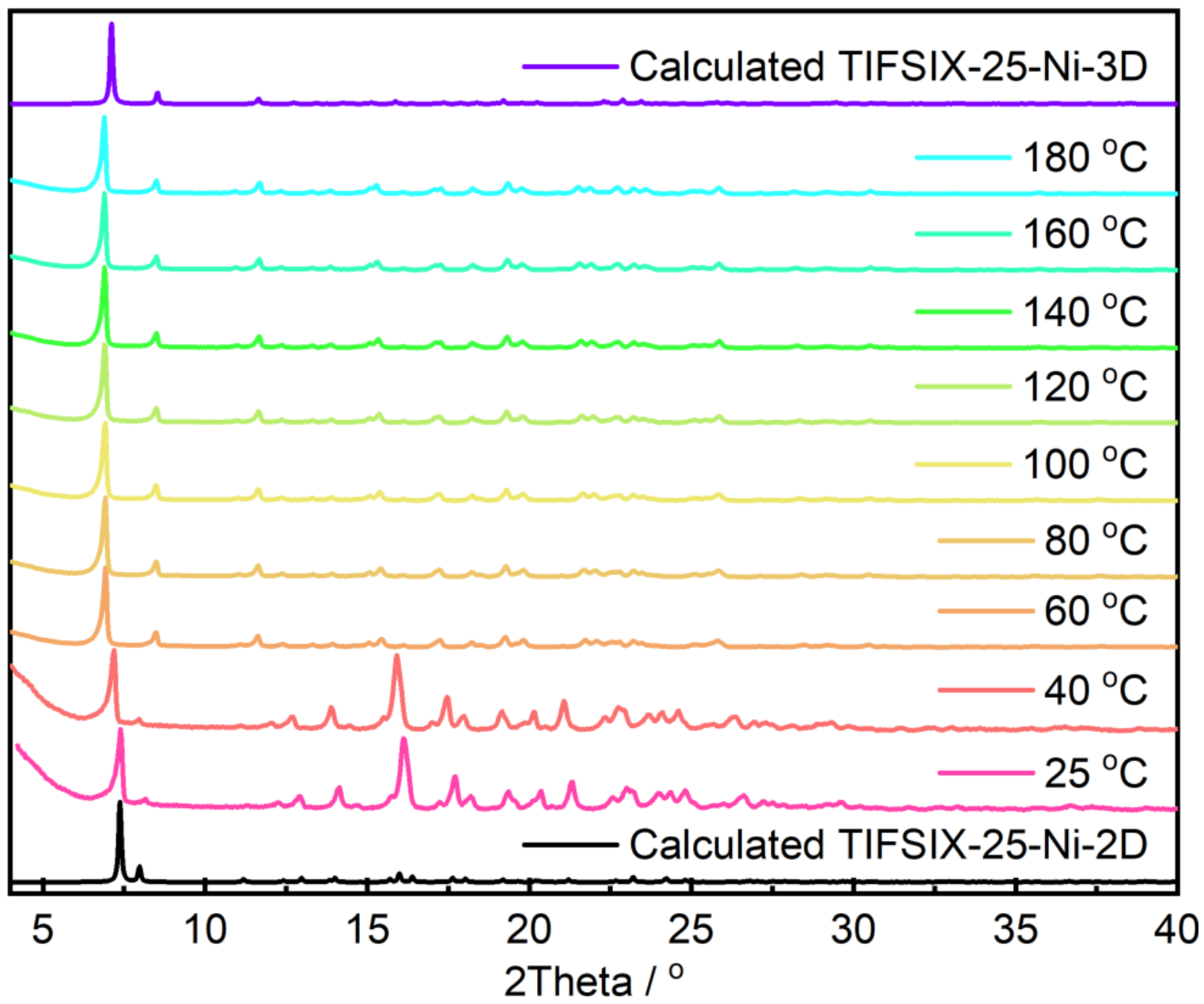

Figure S39 VTPXRD patterns of heating as-synthesized **TIFSIX-25-Ni**. Reference diffractograms were determined from the crystal structures of 2D **sql** (bottom) and 3D **pcu** (top) frameworks.

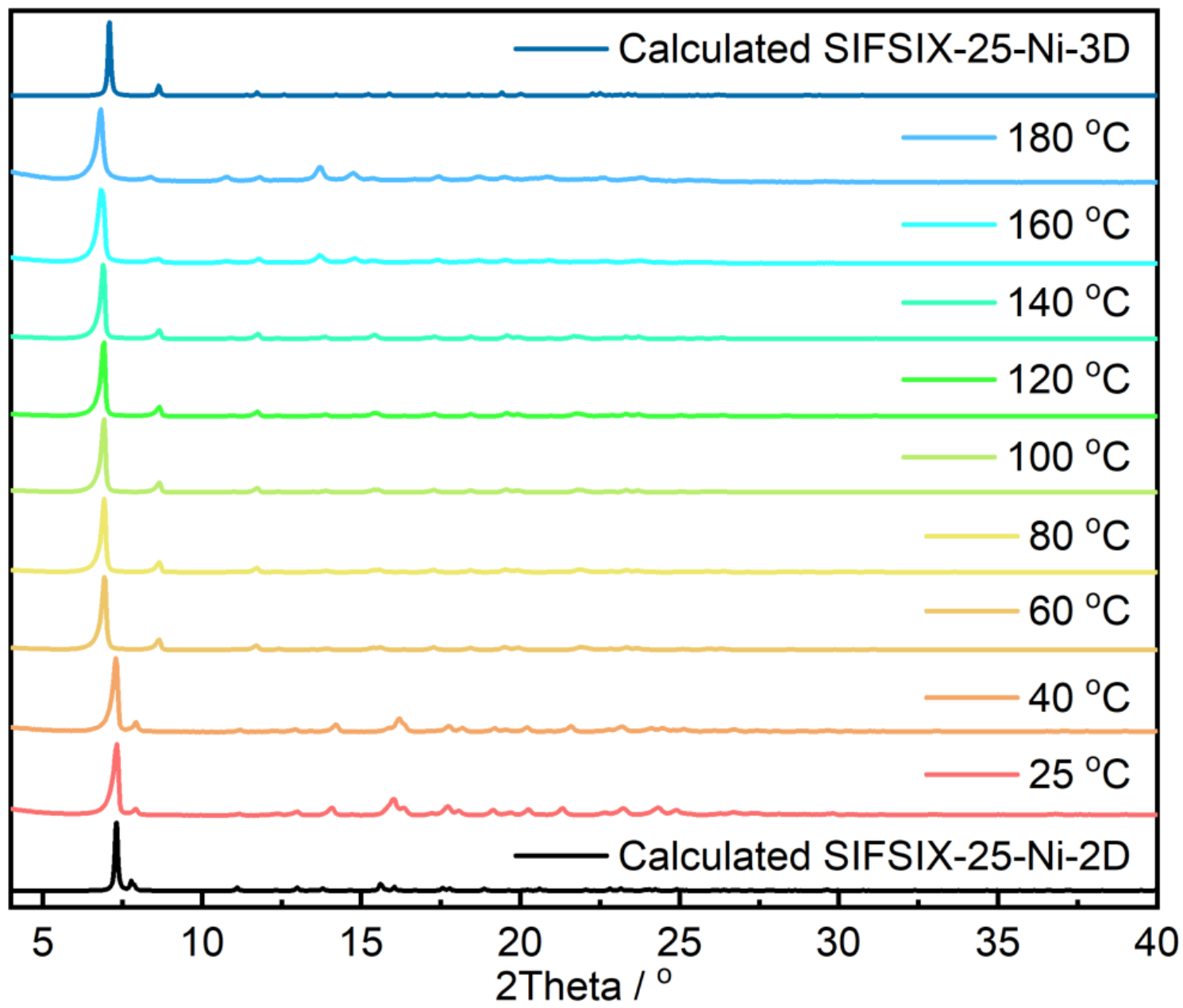

Figure S40 VTPXRD patterns of heating as-synthesized **SIFSIX-25-Ni**. Reference diffractograms were determined from the crystal structures of 2D **sql** (bottom) and 3D **pcu** (top) frameworks.

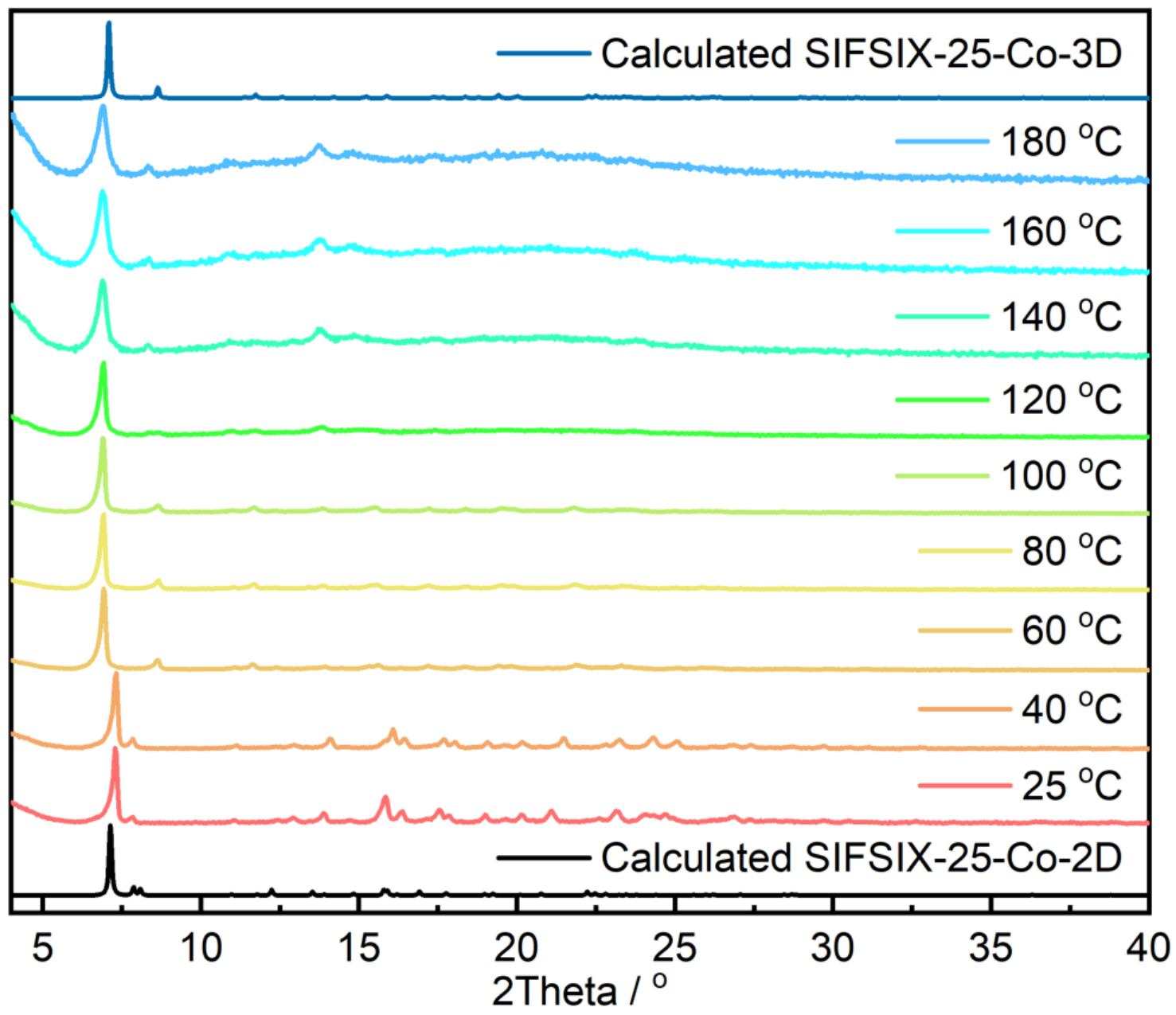

Figure S41 VTPXRD patterns of heating as-synthesized **SIFSIX-25-Co**. Reference diffractograms were determined from the crystal structures of 2D **sql** (bottom) and 3D **pcu** (top) frameworks.

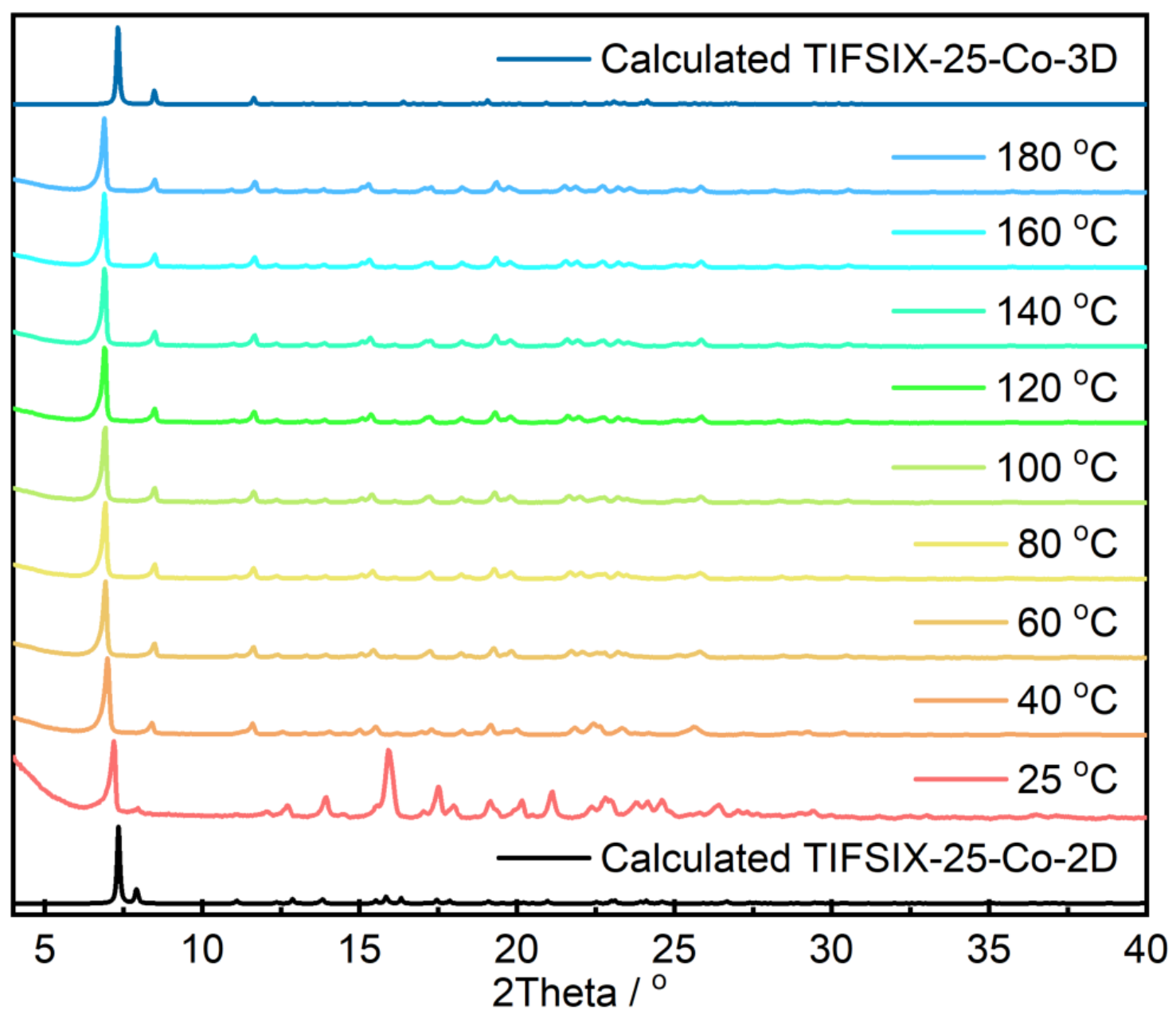

Figure S42 VTPXRD patterns of heating as-synthesized **TIFSIX-25-Co**. Reference diffractograms were determined from the crystal structures of 2D **sql** (bottom) and 3D **pcu** (top) frameworks.

10 Comparison of loaded and unloaded PXRDs

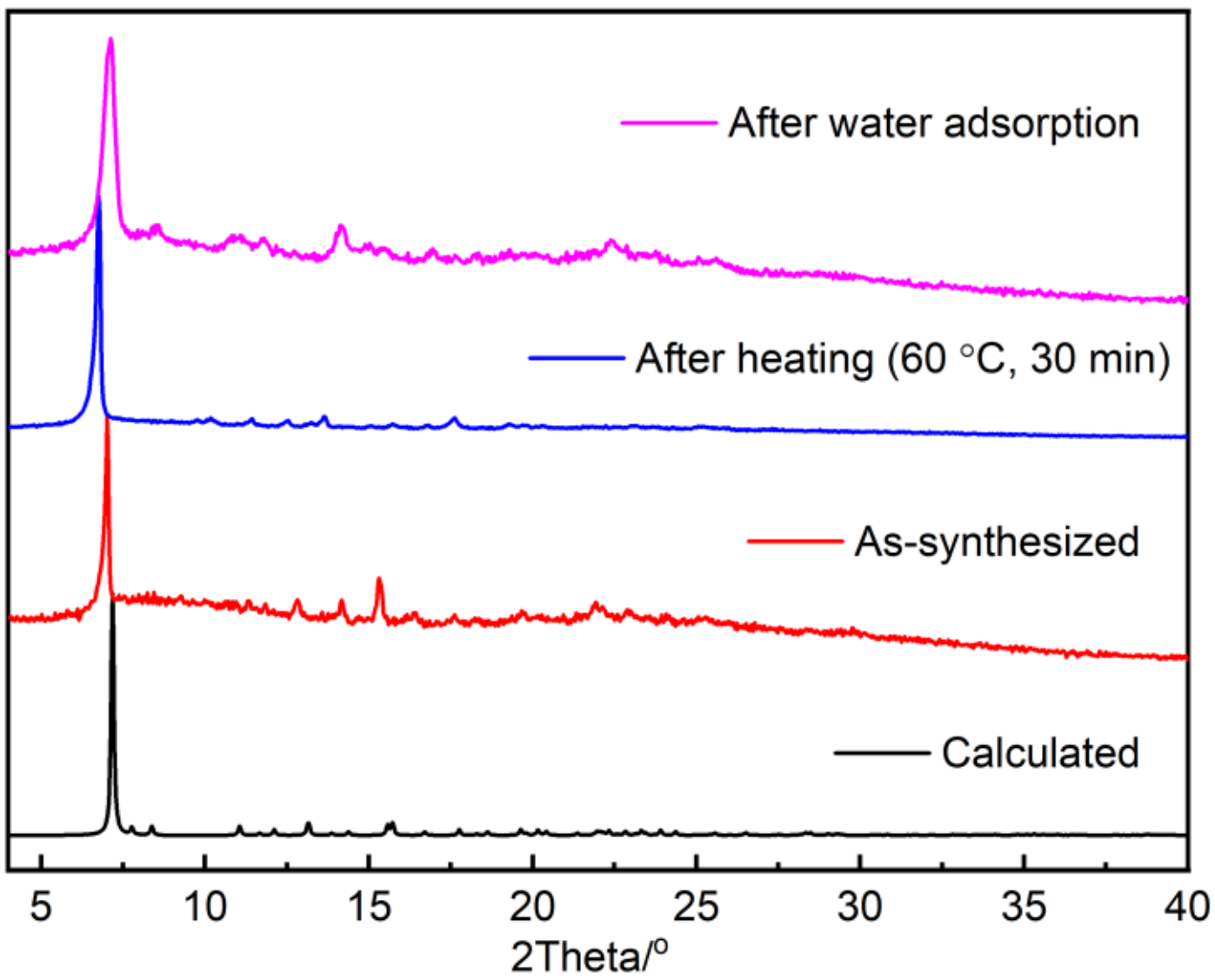

Figure S43 PXRD patterns of **SIFSIX-25-Cu** (bottom to top): calculated from the crystal structure of the **pcu**, as-synthesized, after heating (under N_2 flow) after water sorption.

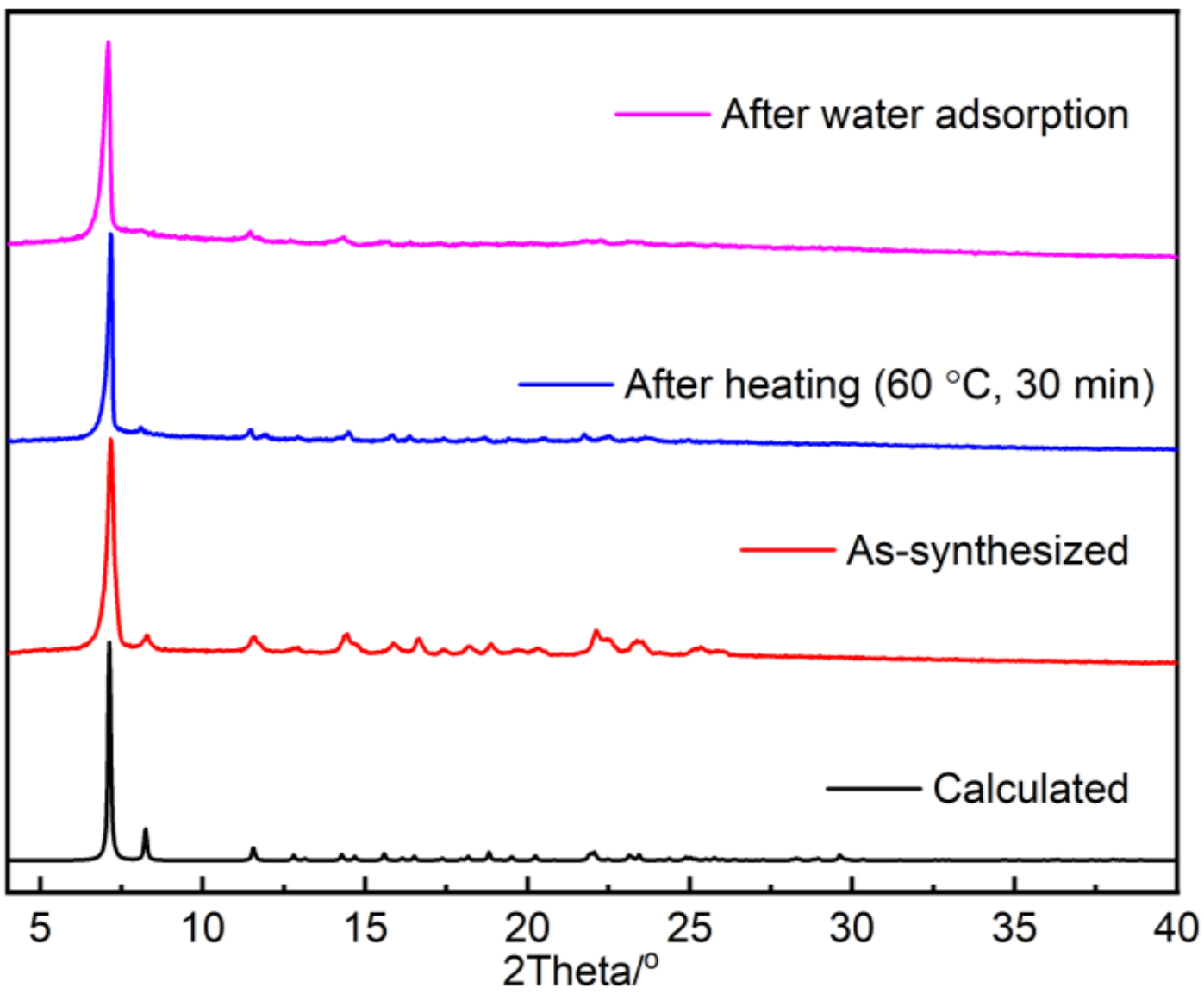

Figure S44 PXRD patterns of **TIFSIX-25-Cu** (bottom to top): calculated from the crystal structure of the **pcu**, as-synthesized, after heating (under N_2 flow) after water sorption.

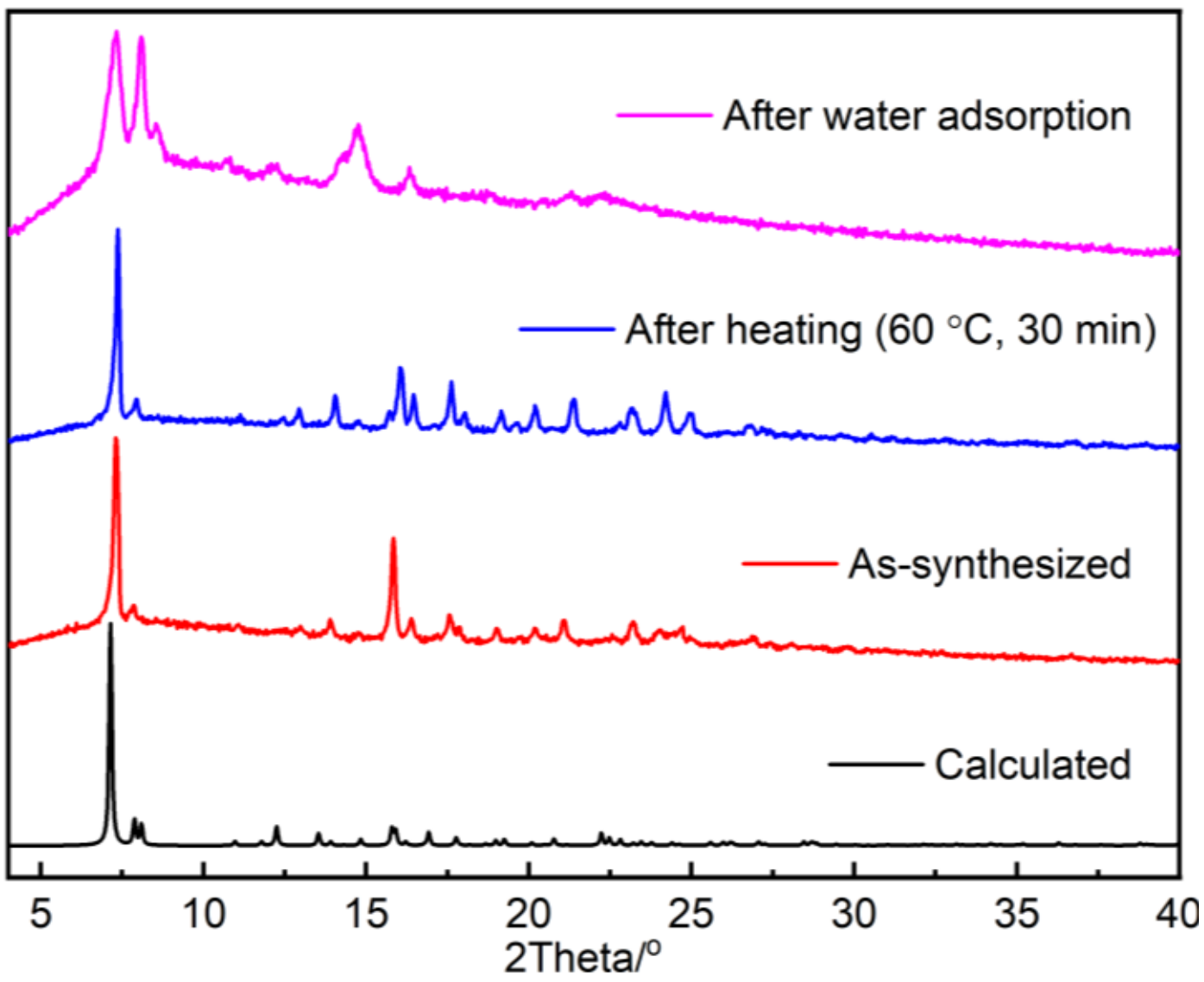

Figure S45 PXRD patterns of **SIFSIX-25-Co** (bottom to top): calculated from the crystal structure of the **sql**, as-synthesized, after heating (under N_2 flow) after water sorption.

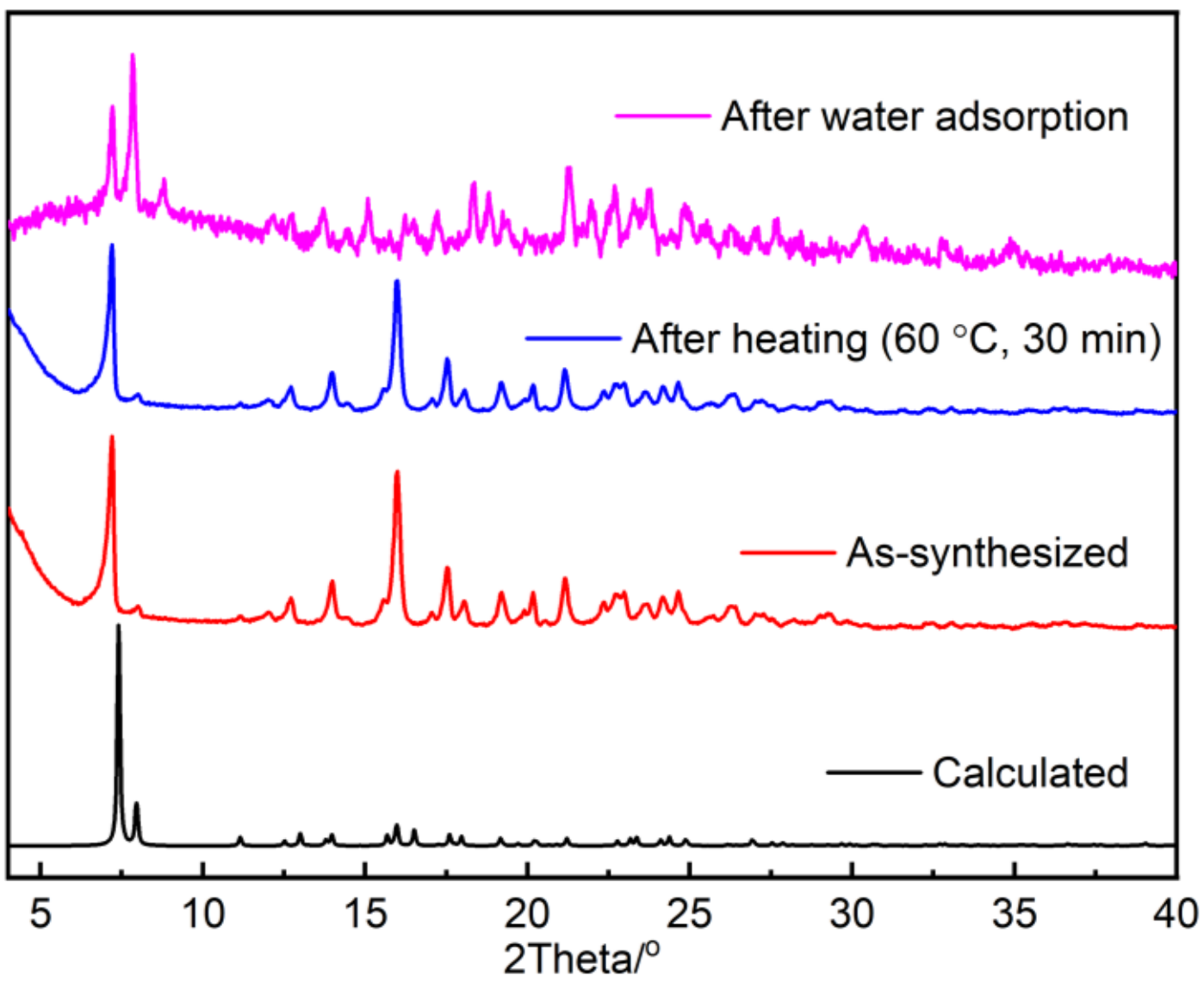

Figure S46 PXRD patterns of **TIFSIX-25-Co** (bottom to top): calculated from the crystal structure of the **sql**, as-synthesized, after heating (under N_2 flow) after water sorption.

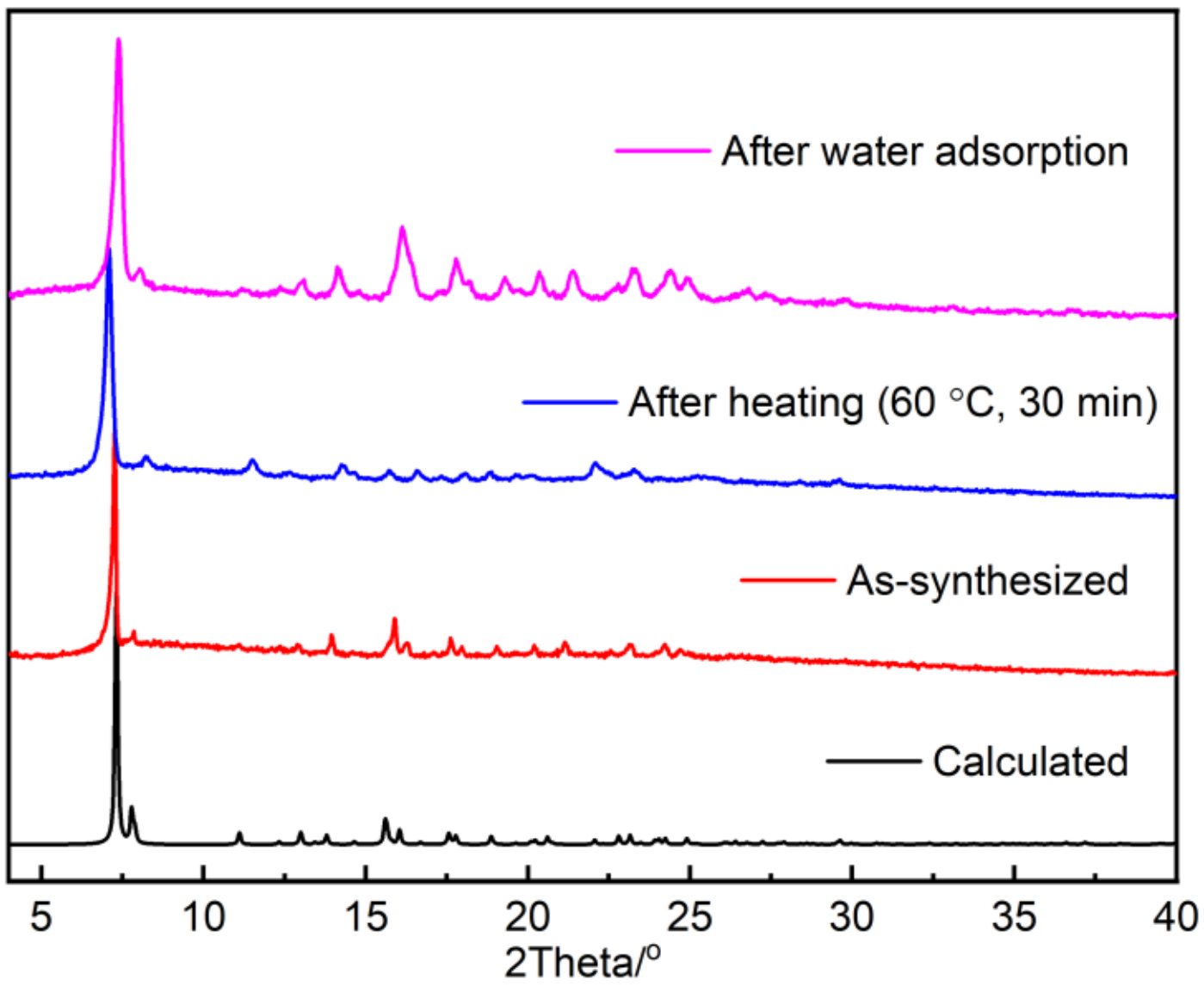

Figure S47 PXRD patterns of **SIFSIX-25-Ni** (bottom to top): calculated from the crystal structure of the **sql**, as-synthesized, after heating (under N_2 flow) after water sorption.

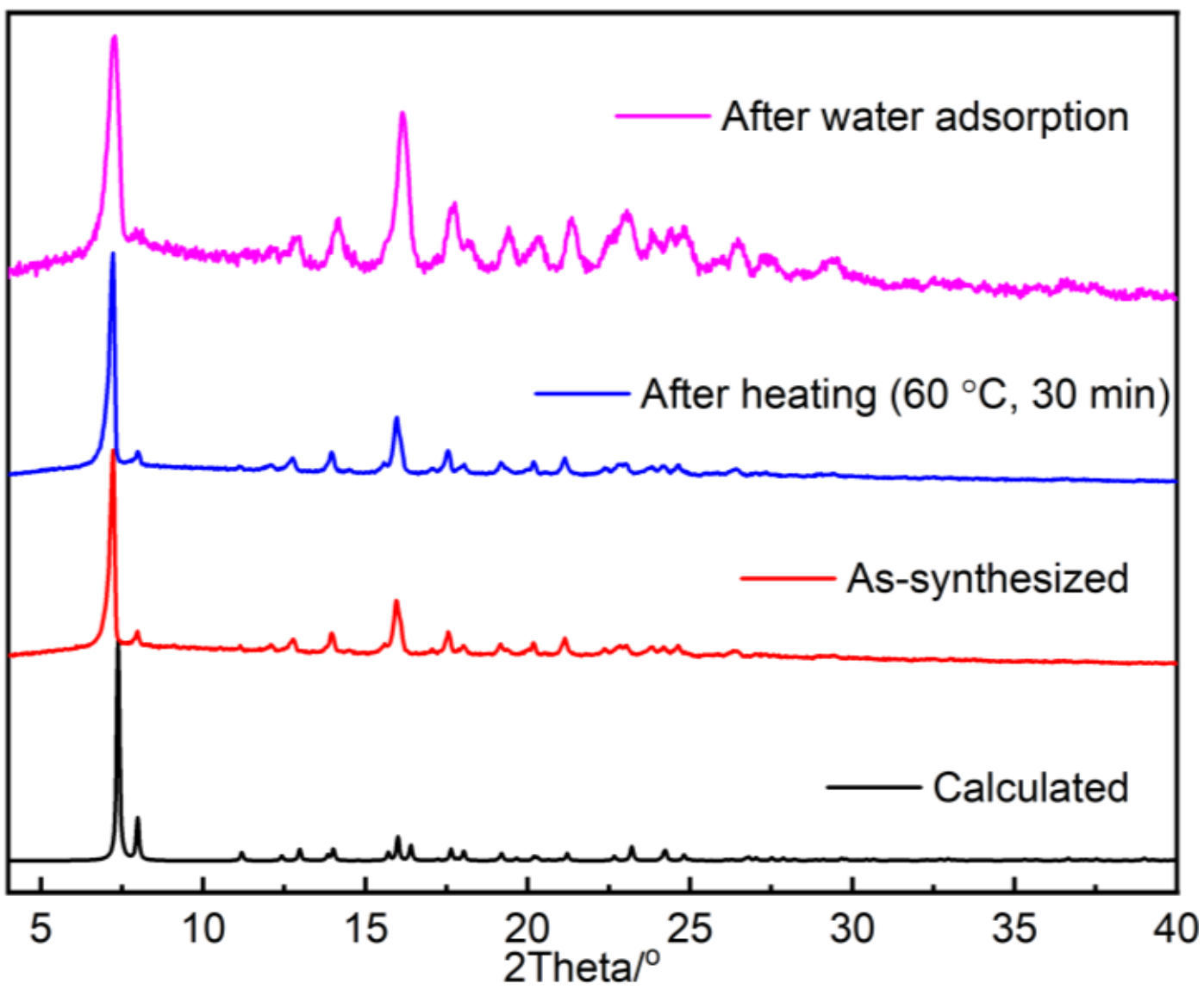

Figure S48 PXRD patterns of **TIFSIX-25-Ni** (bottom to top): calculated from the crystal structure of the **sql**, as-synthesized, after heating (under N_2 flow) after water sorption.

11 Gas sorption

11.1 M'FSIX-25-M and Post-Adsorption PXRD

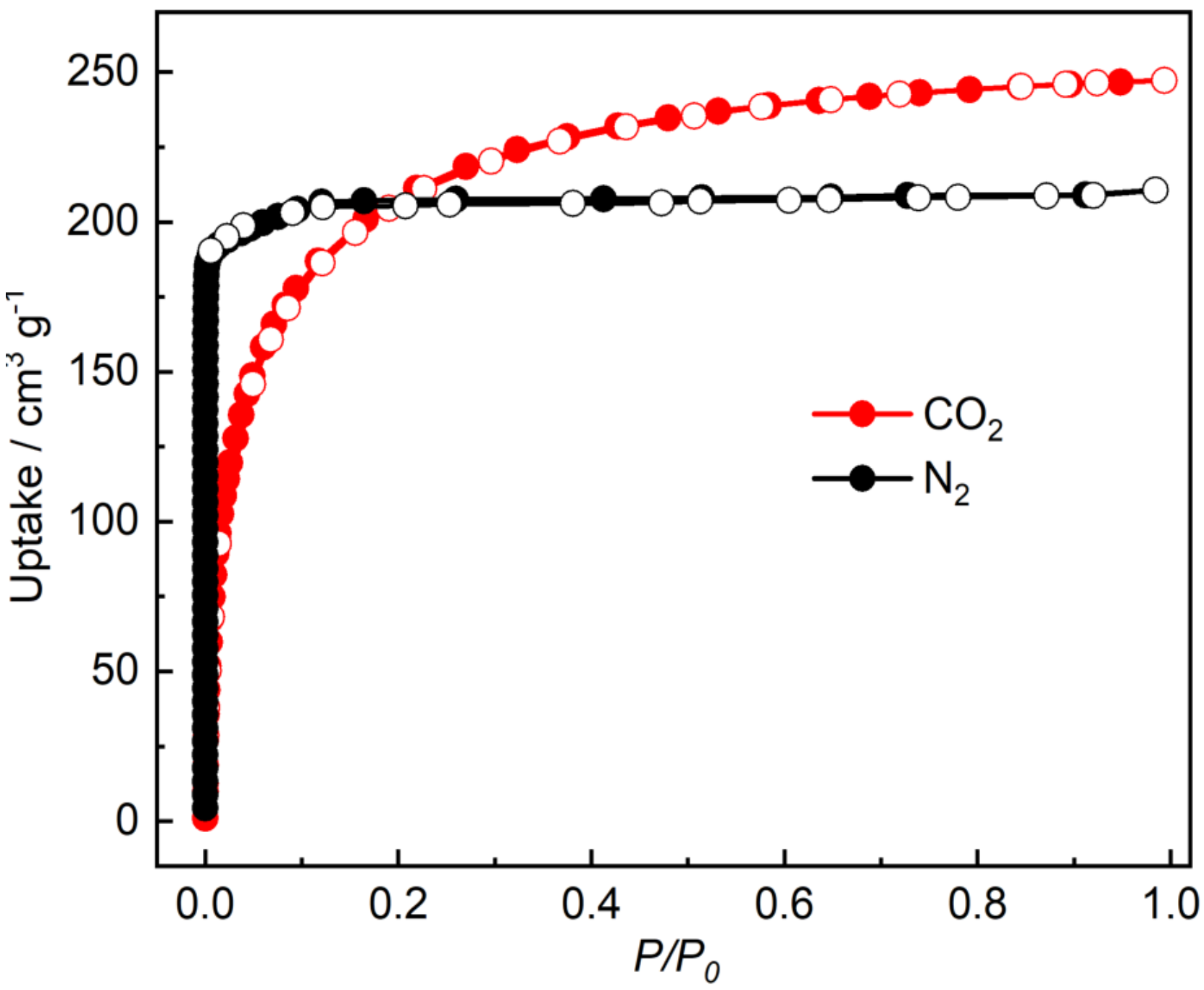

Figure S49 CO_2 and N_2 adsorption isotherms of **TIFSIX-25-Ni** measured at 195 and 77 K, respectively.

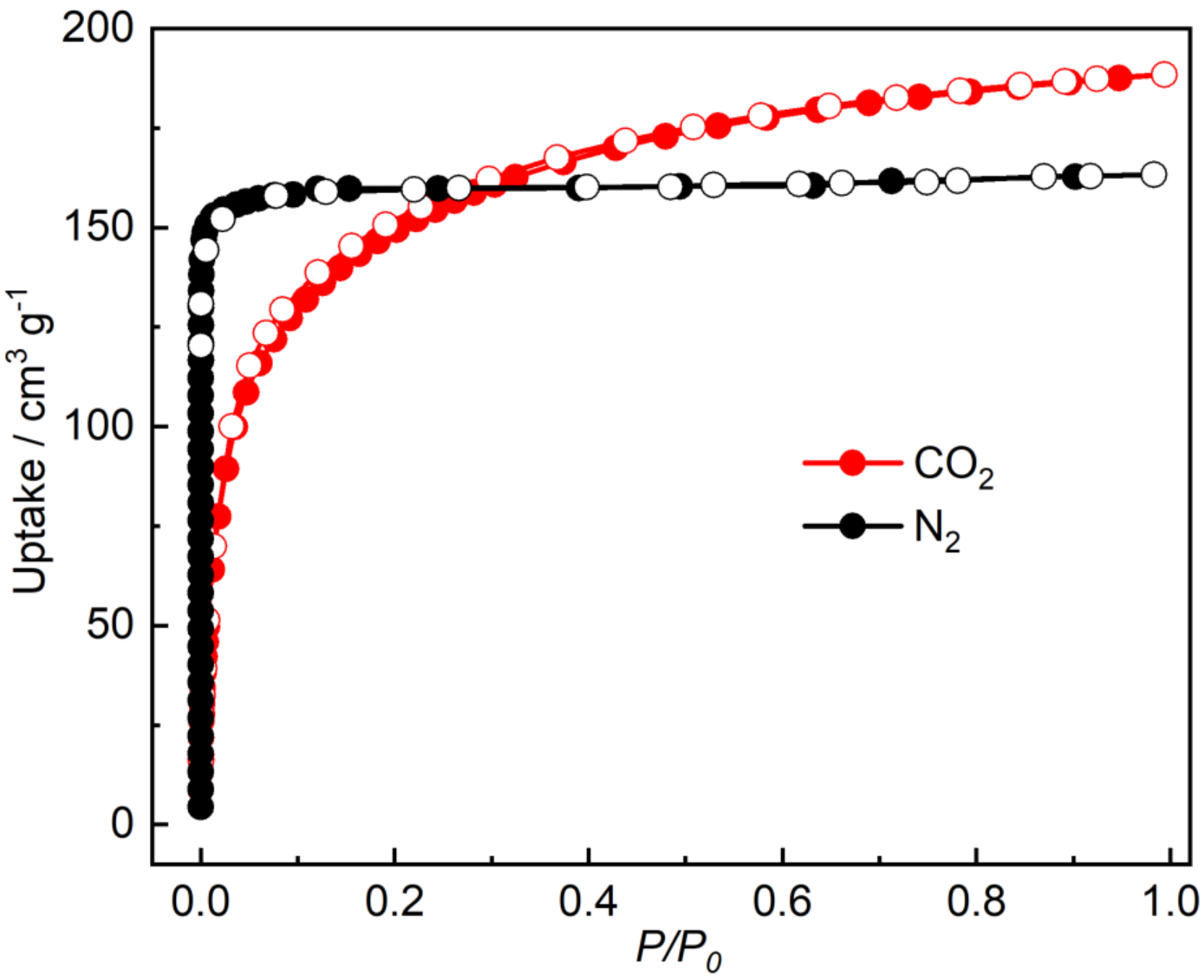

Figure S50 CO_2 and N_2 adsorption isotherms of **SIFSIX-25-Ni** measured at 195 and 77 K, respectively.

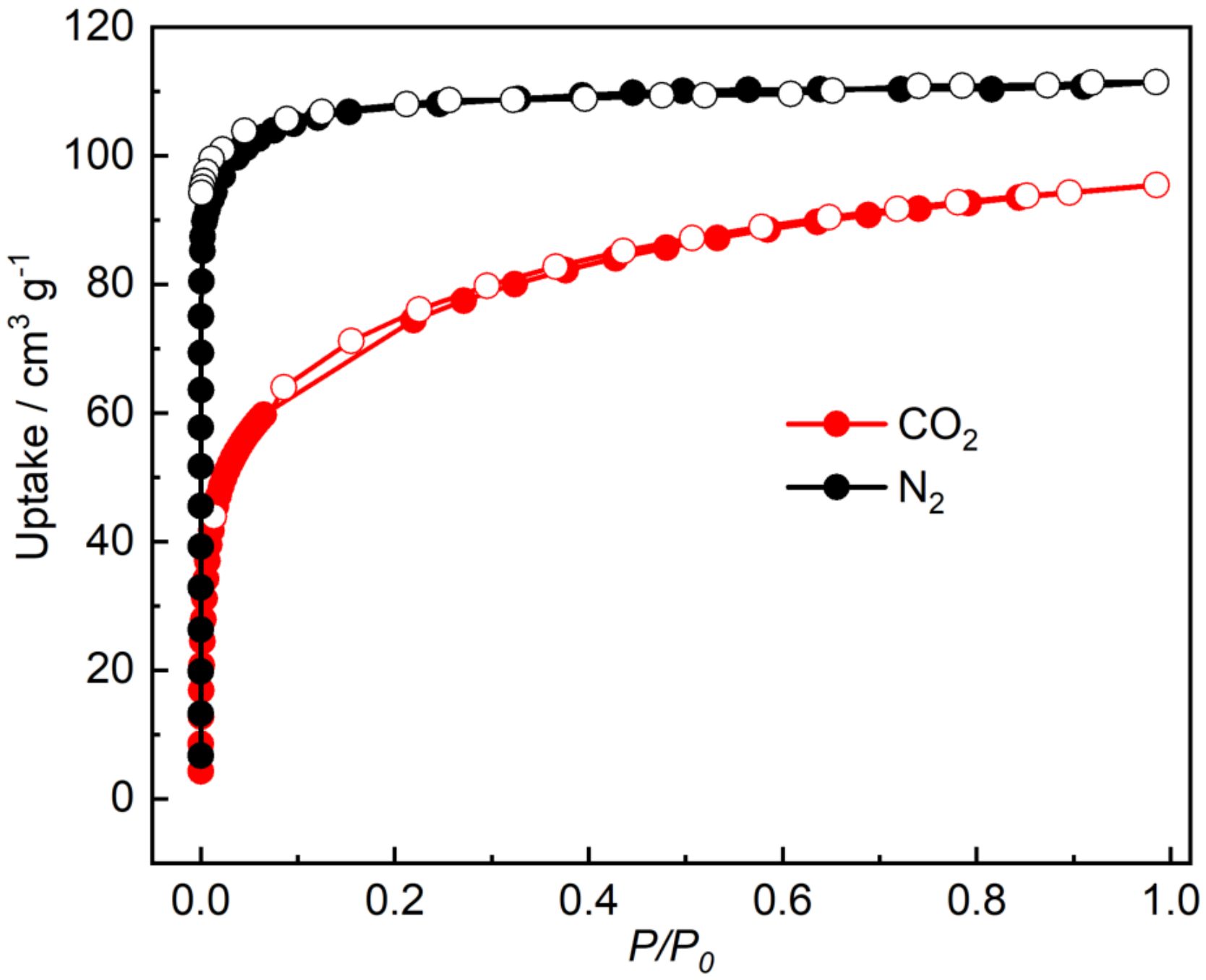

Figure S51 CO_2 and N_2 adsorption isotherms of **TIFSIX-25-Co** measured at 195 and 77 K, respectively.

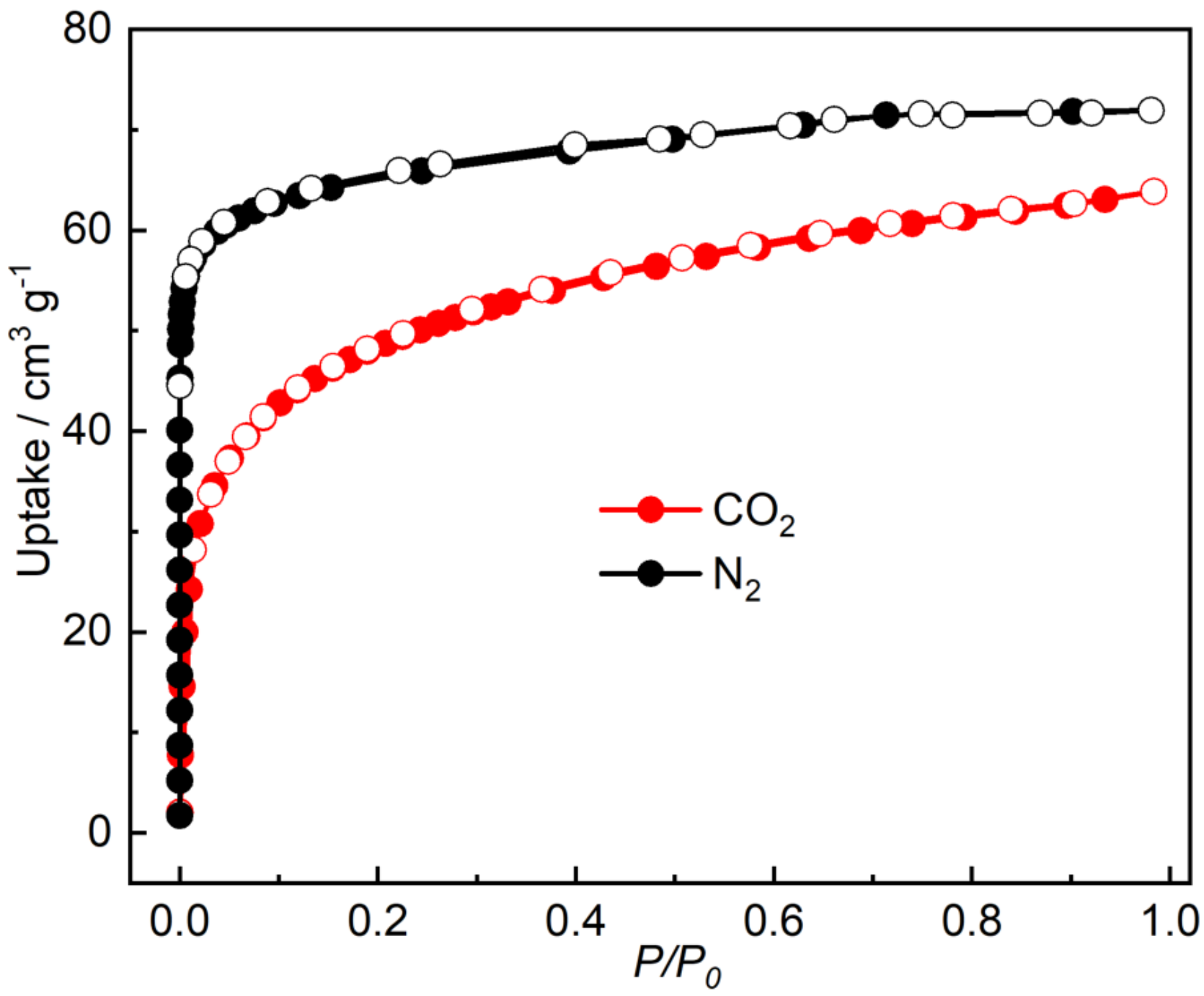

a

Figure S52 CO_2 and N_2 adsorption isotherms of **SIFSIX-25-Co** measured at 195 and 77 K, respectively.

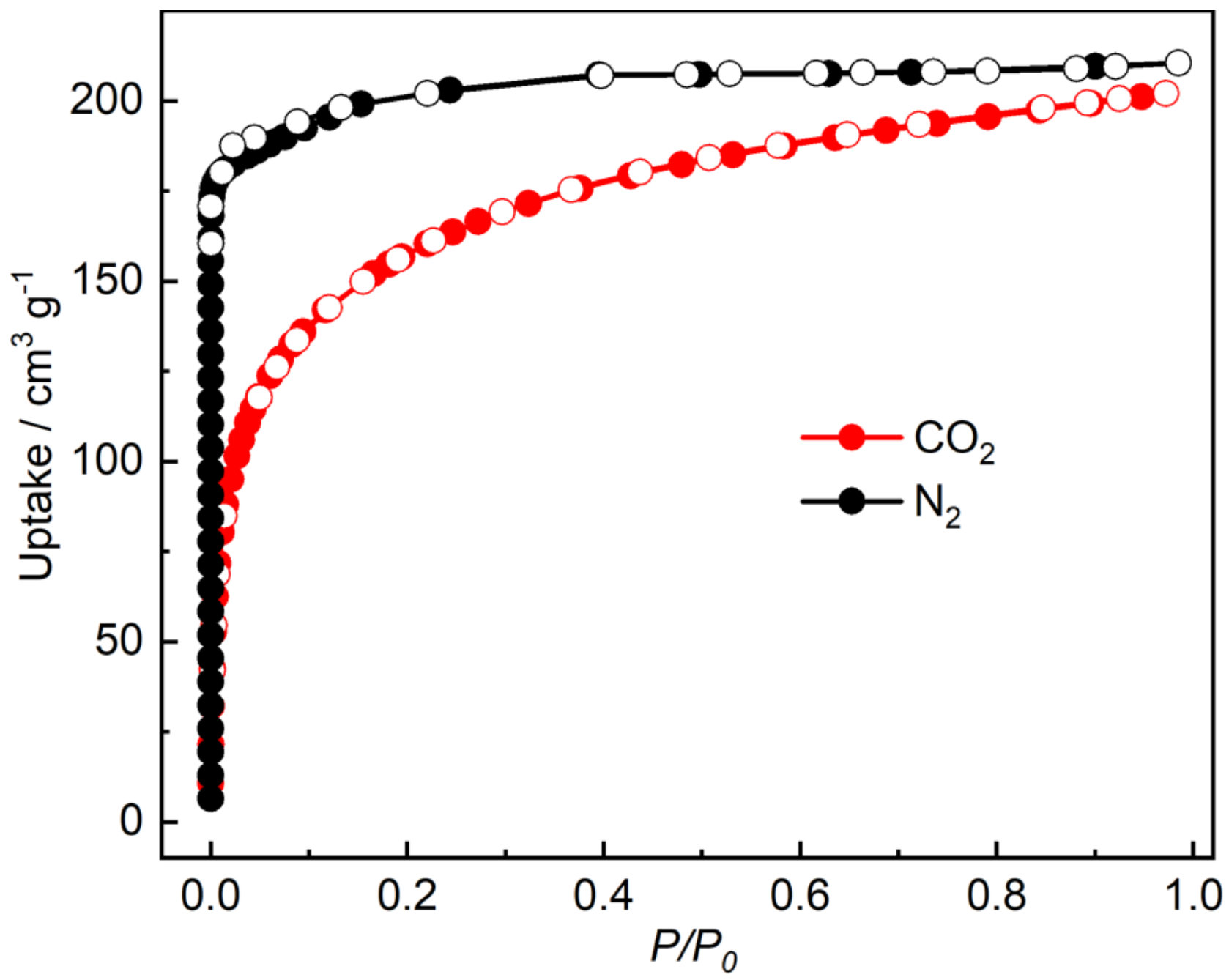

Figure S53 CO_2 and N_2 adsorption isotherms of **TIFSIX-25-Cu** measured at 195 and 77 K, respectively.

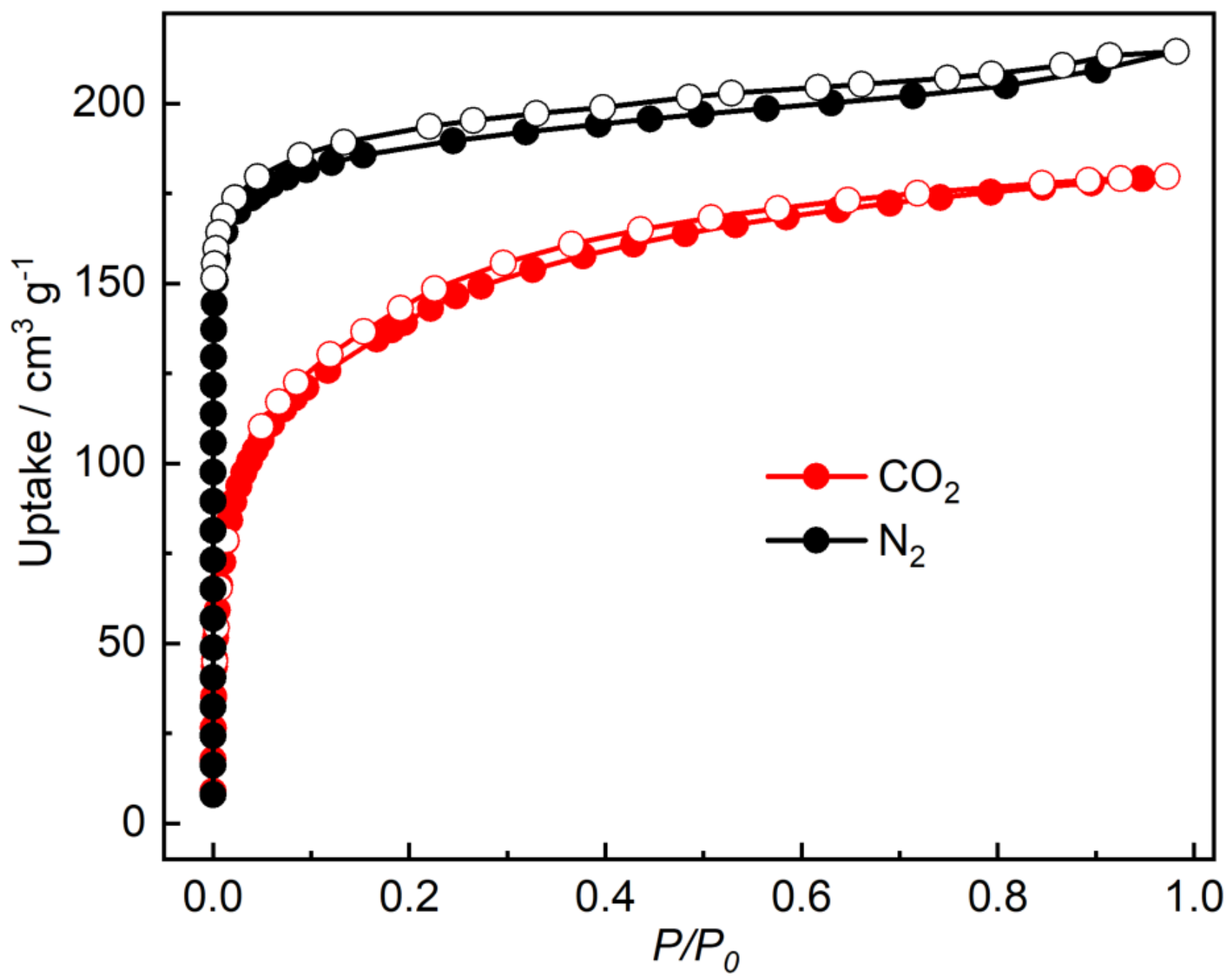

Figure S54 CO_2 and N_2 adsorption isotherms of **SIFSIX-25-Cu** measured at 195 and 77 K, respectively.

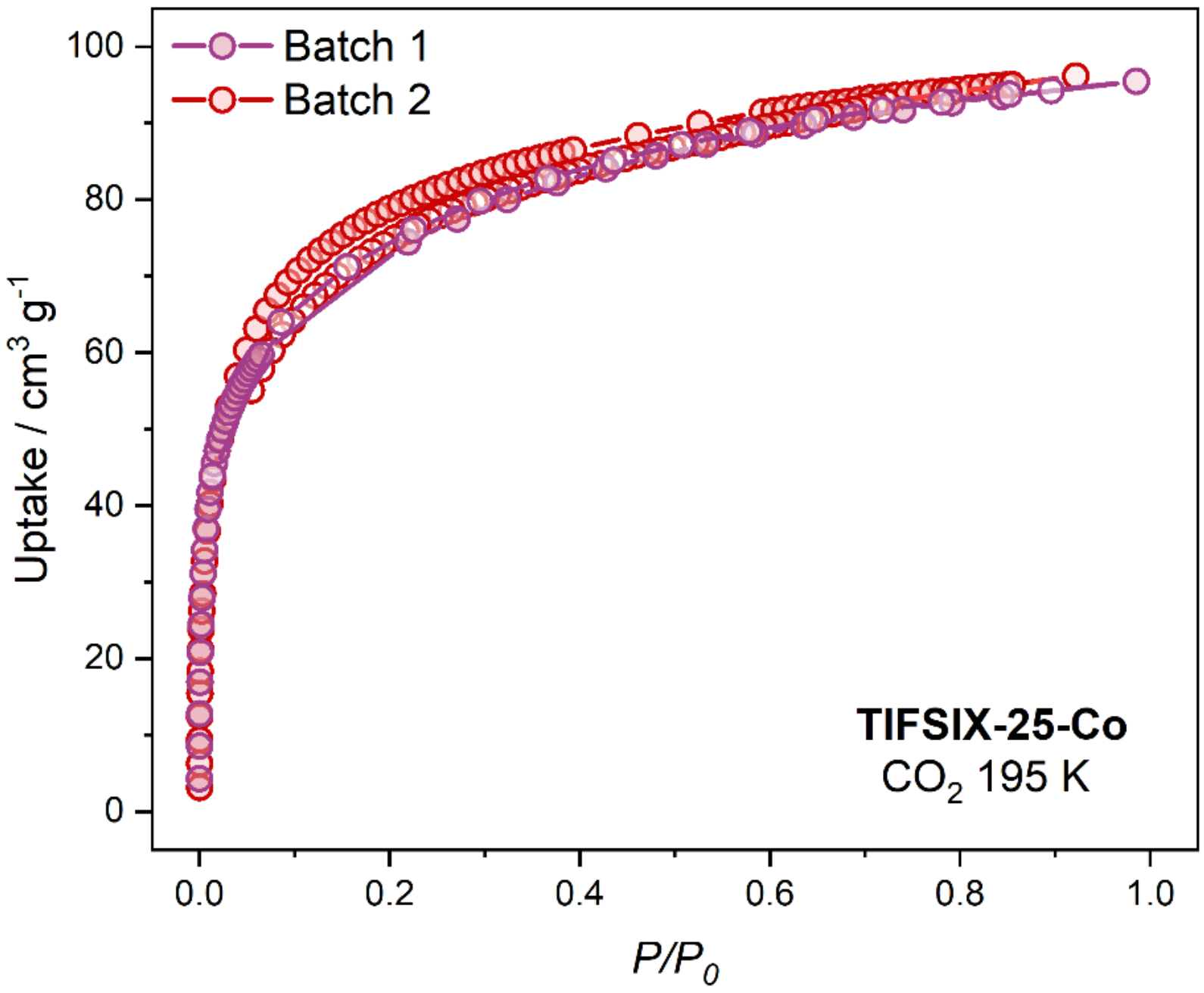

Figure S55 CO_2 adsorption isotherms at 195 K for two independently synthesized batches of **TIFSIX-25-Co**, demonstrating reproducibility in adsorption performance across the entire pressure range.

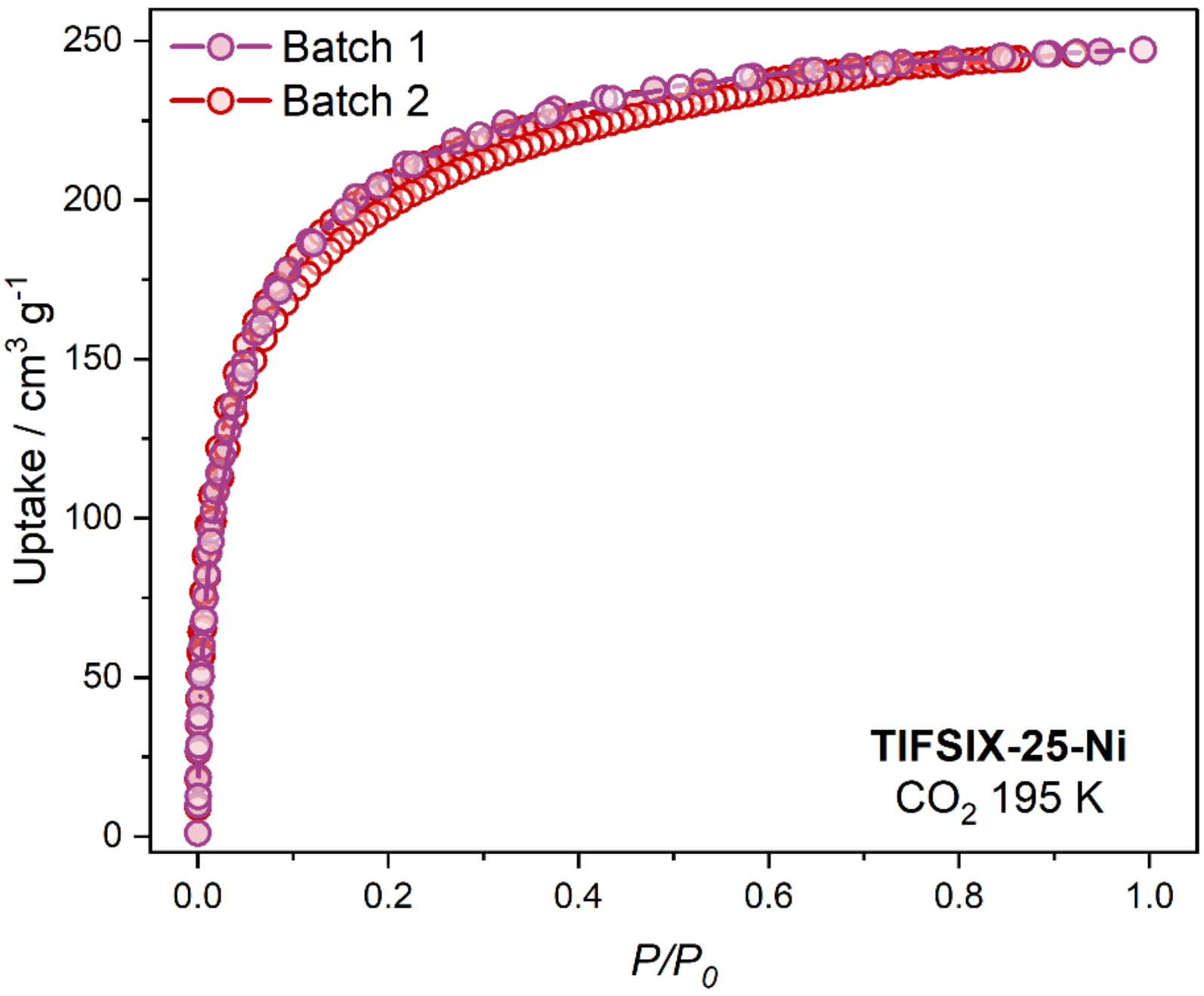

Figure S56 CO_2 adsorption isotherms at 195 K for two independently synthesized batches of **TIFSIX-25-Ni**, demonstrating reproducibility in adsorption performance across the entire pressure range.

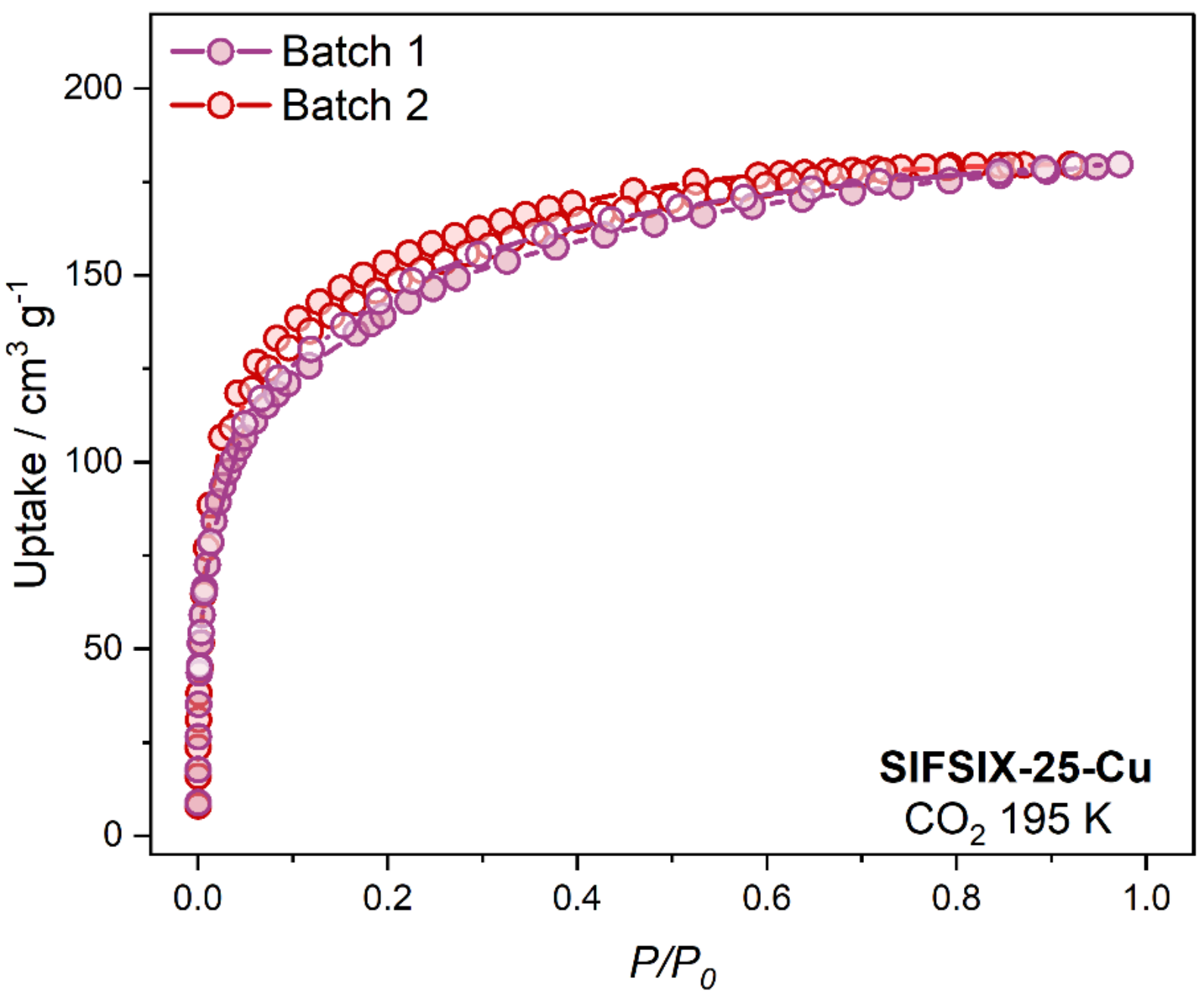

Figure S57 CO_2 adsorption isotherms at 195 K for two independently synthesized batches of **SIFSIX-25-Cu**, demonstrating reproducibility in adsorption performance across the entire pressure range.

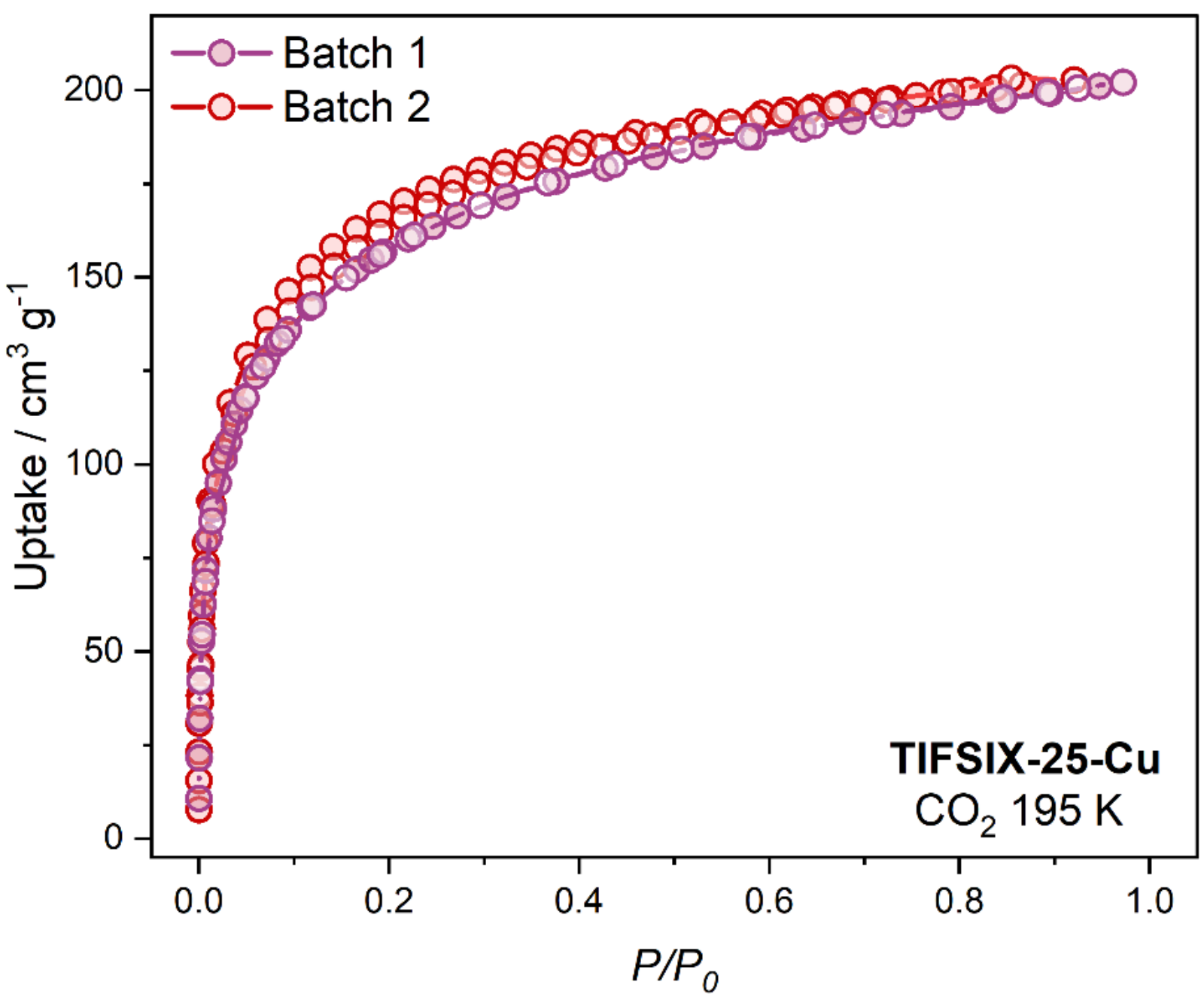

Figure S58 CO_2 adsorption isotherms at 195 K for two independently synthesized batches of **TIFSIX-25-Cu**, demonstrating reproducibility in adsorption performance across the entire pressure range.

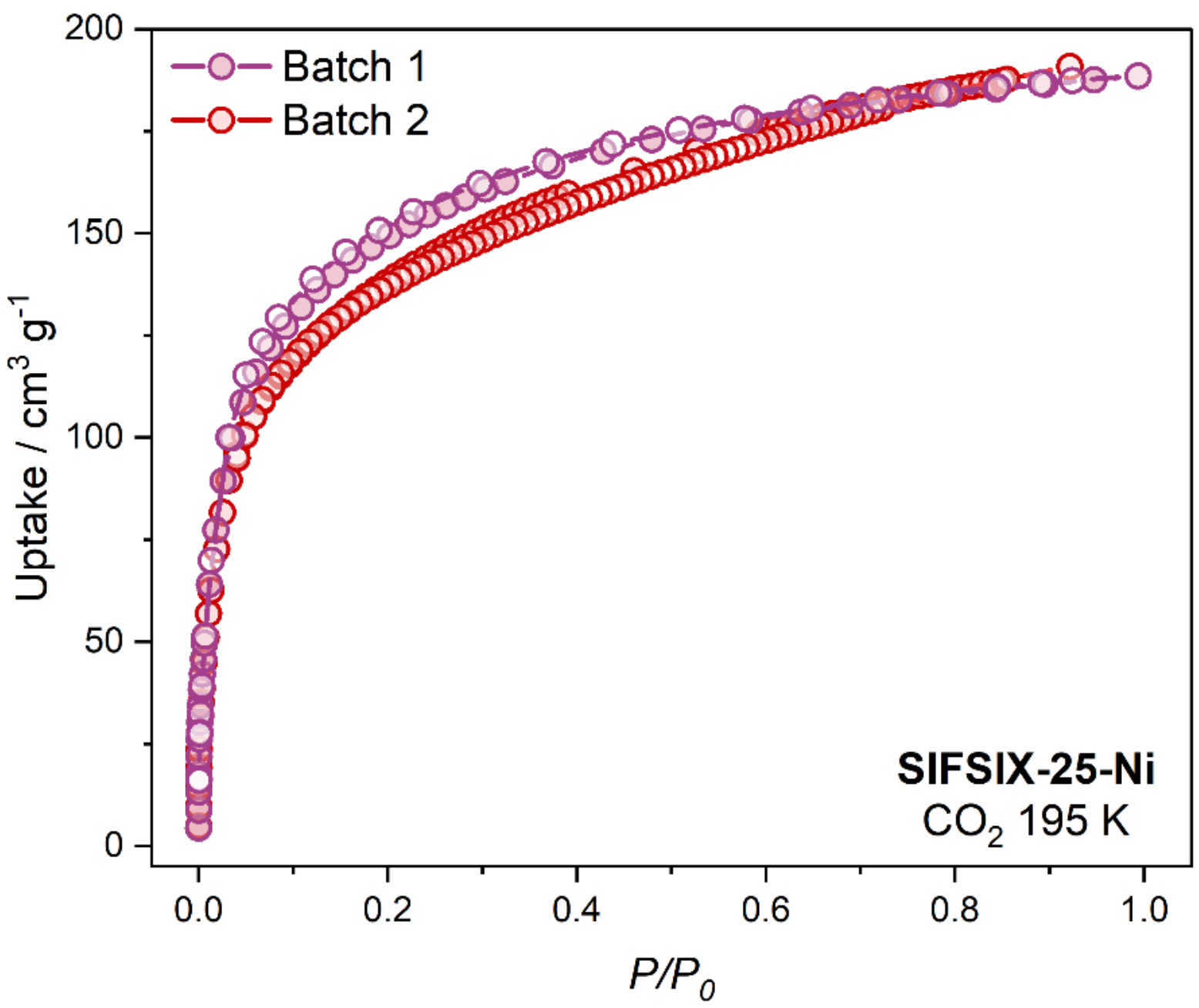

Figure S59 CO_2 adsorption isotherms at 195 K for two independently synthesized batches of **SIFSIX-25-Ni**, demonstrating reproducibility in adsorption performance across the entire pressure range.

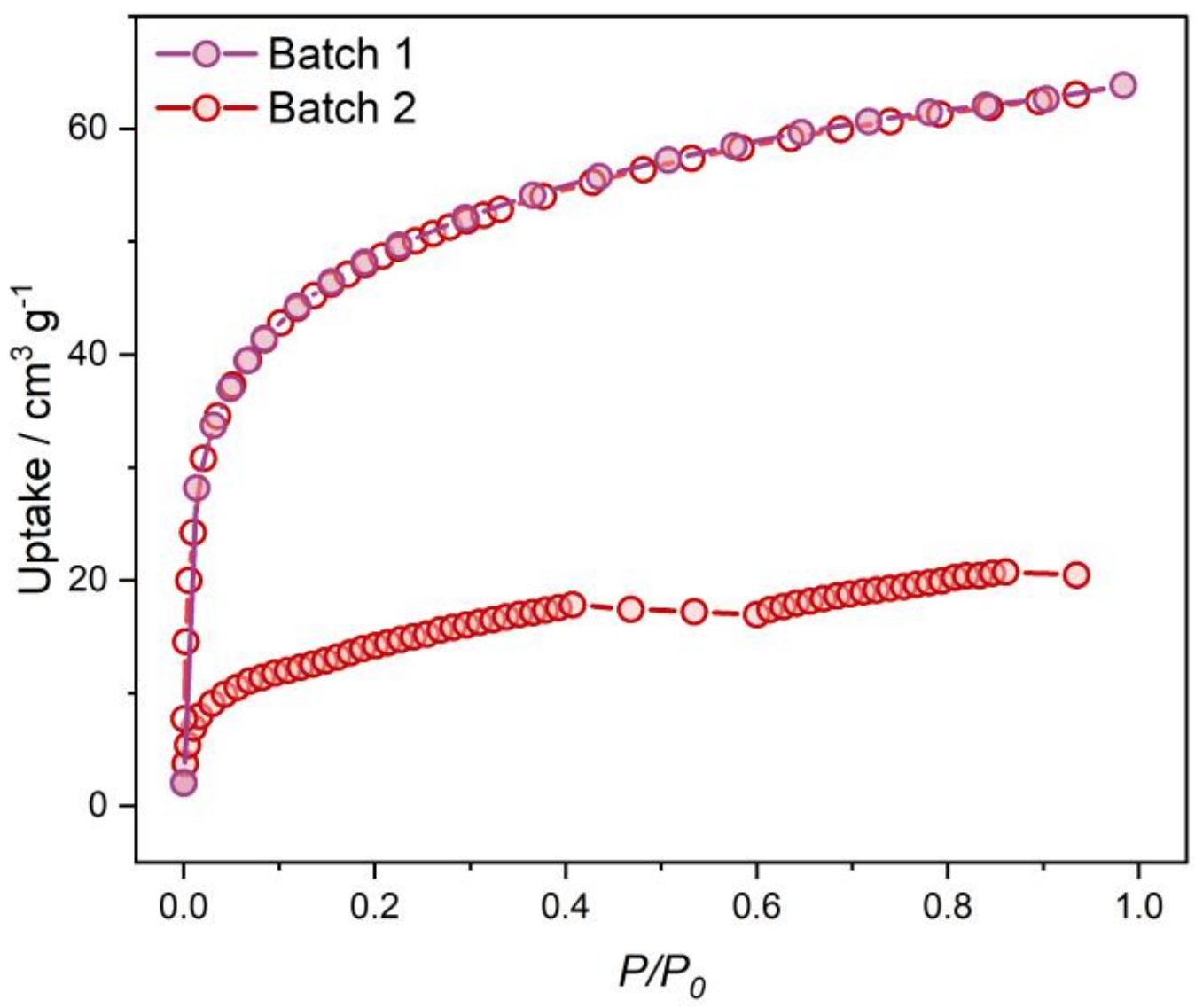

Figure S60 CO_2 adsorption isotherms at 195 K for two independently synthesized batches of **SIFSIX-25-Co**.

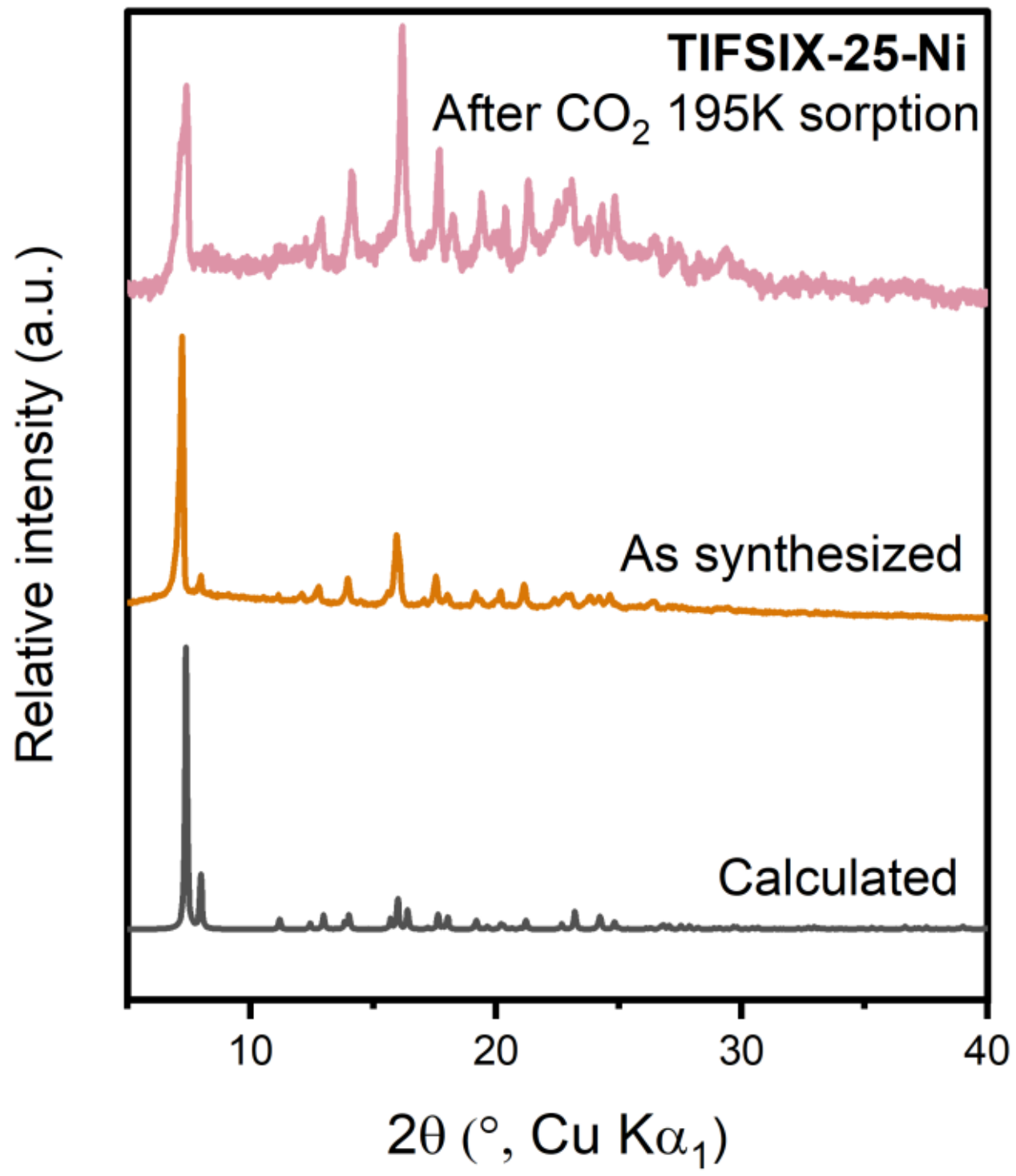

Figure S61 PXRD patterns of calculated, as-synthesized, and after CO_2 (195 K) sorption-treated **TIFSIX-25-Ni**.

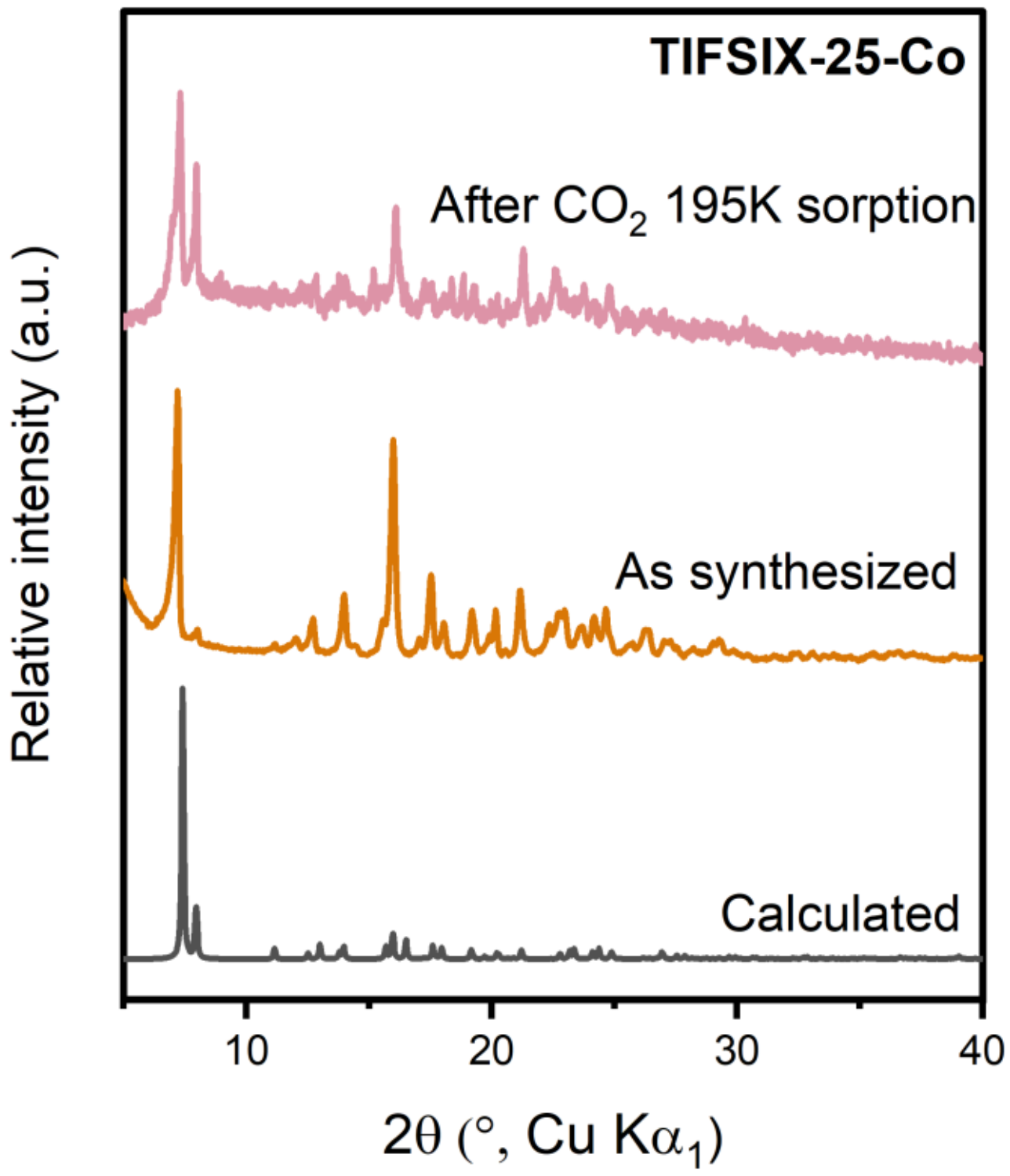

Figure S62 PXRD patterns of calculated, as-synthesized, and after CO_2 (195 K) sorption-treated **TIFSIX-25-Co**.

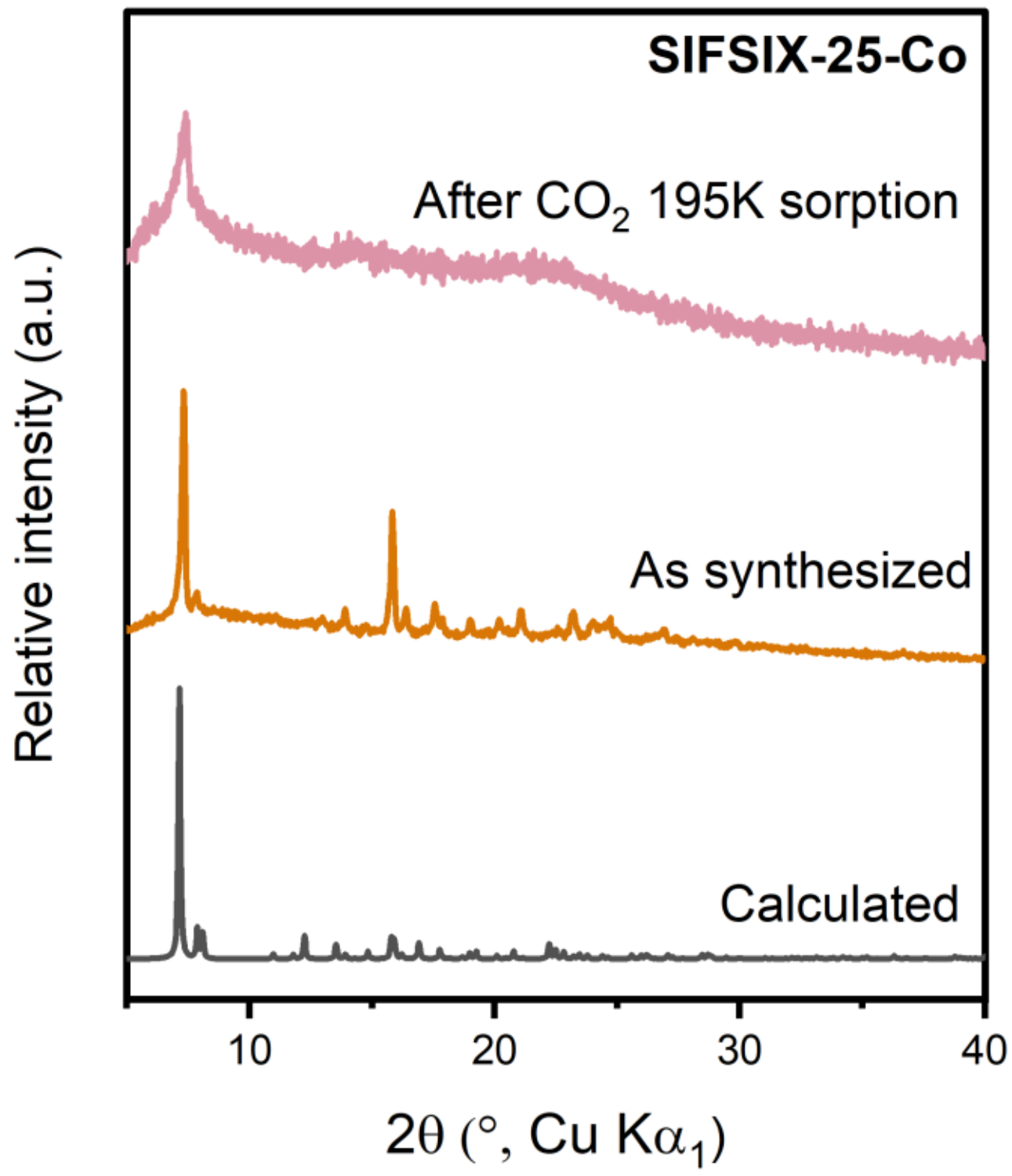

Figure S63 PXRD patterns of calculated, as-synthesized, and after CO_2 (195 K) sorption-treated **SIFSIX-25-Co**.

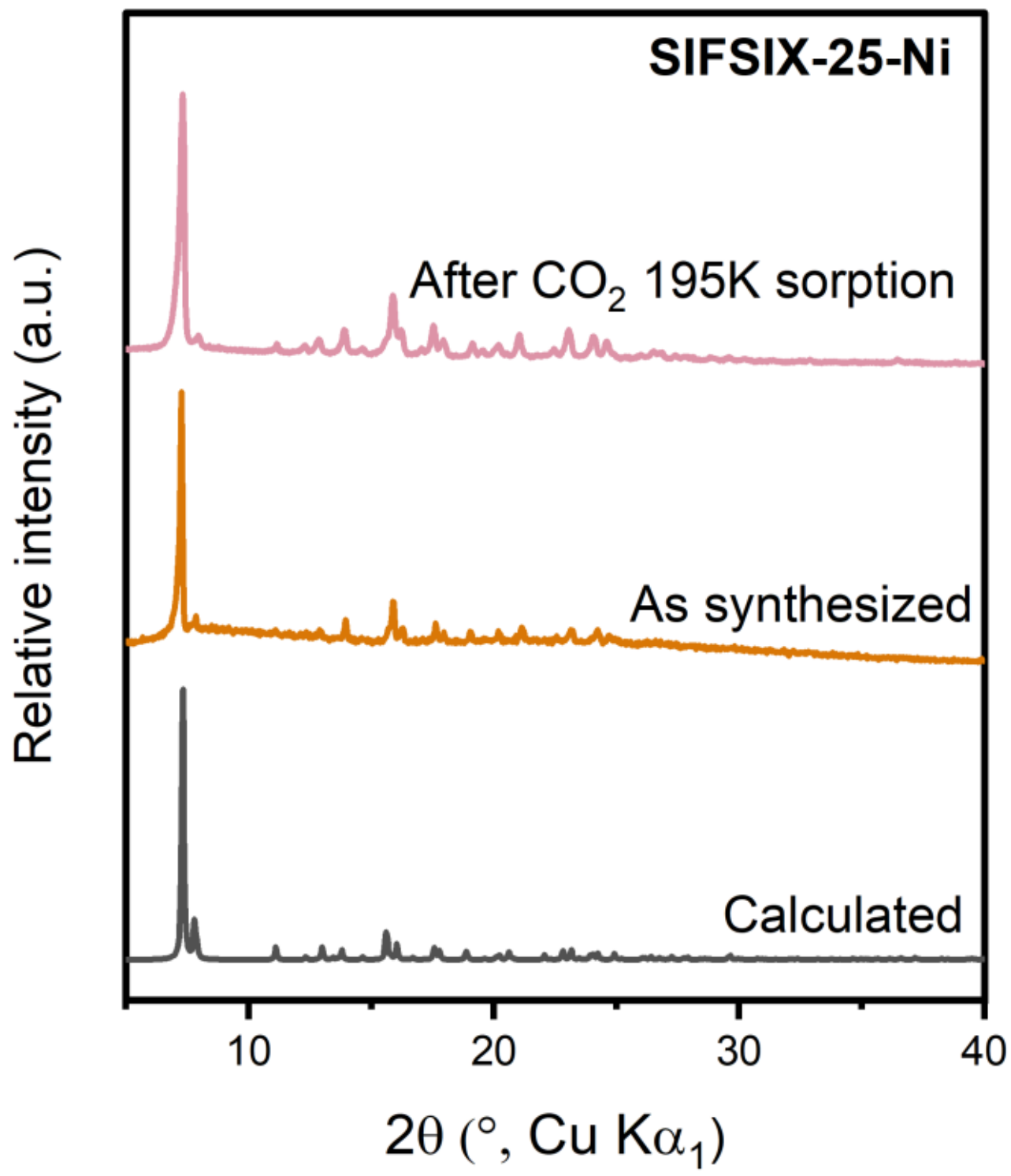

Figure S64 PXRD patterns of calculated, as-synthesized, and after CO_2 (195 K) sorption-treated **SIFSIX-25-Ni**.

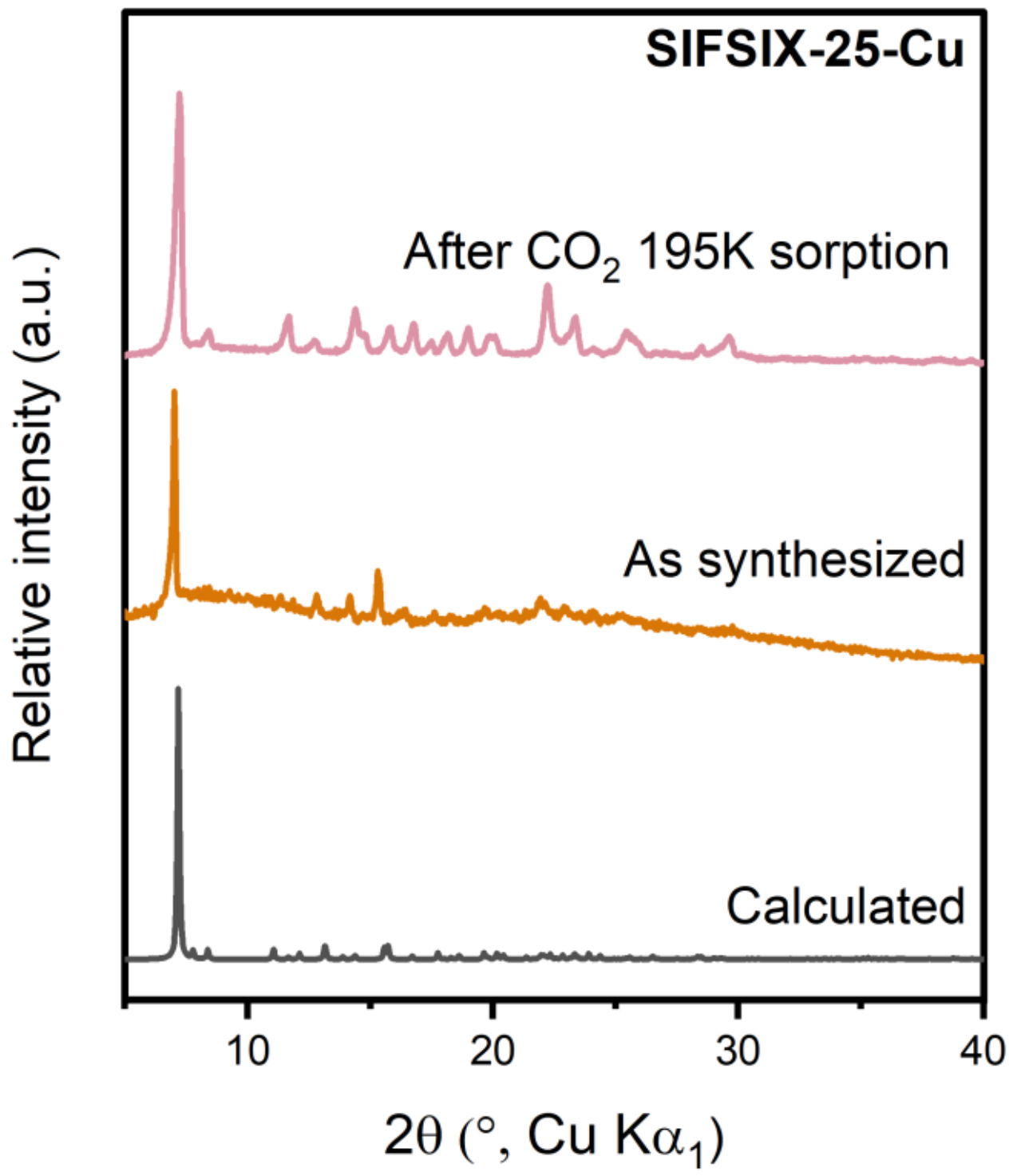

Figure S65 PXRD patterns of calculated, as-synthesized, and after CO_2 (195 K) sorption-treated **SIFSIX-25-Cu**.

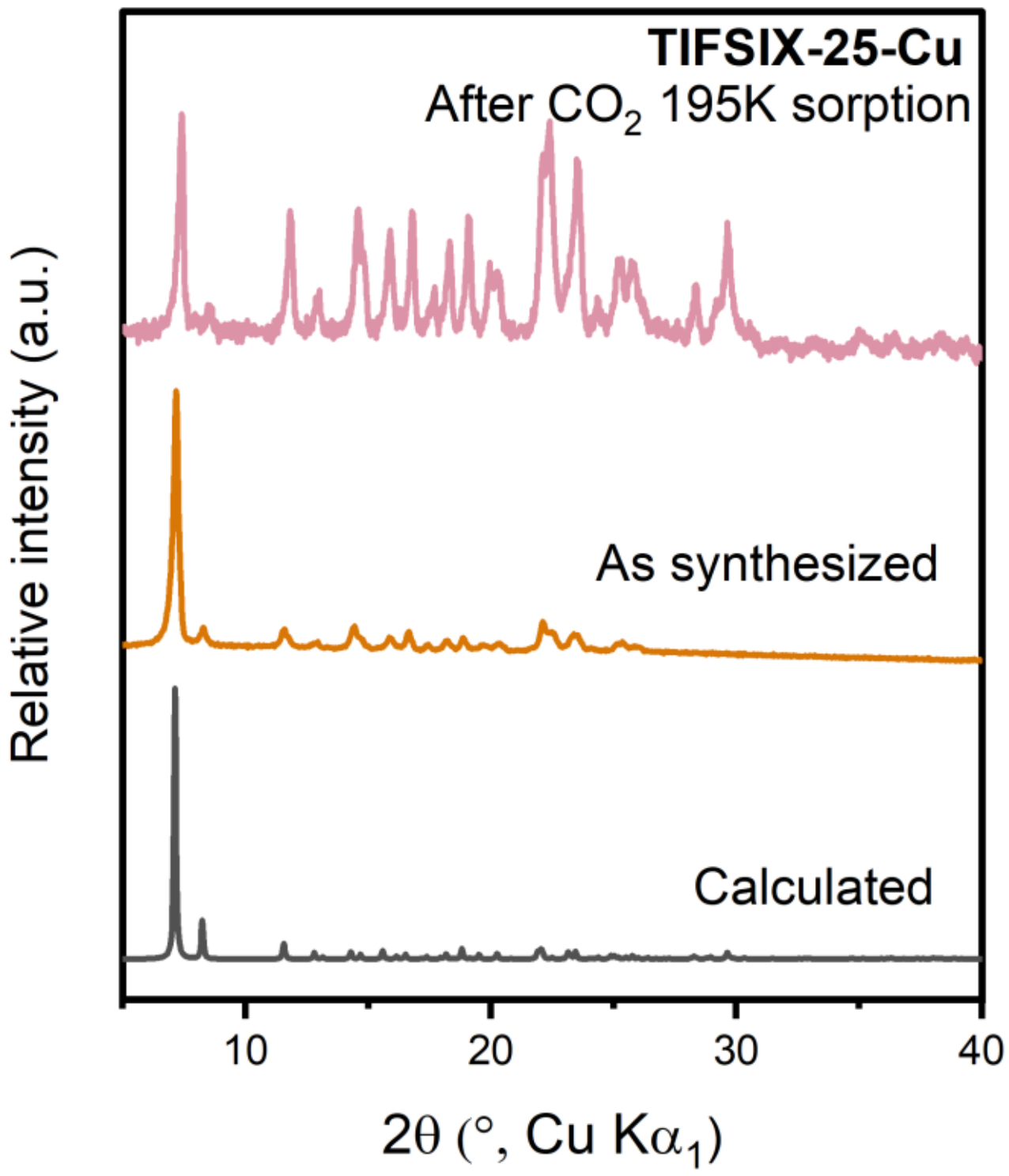

Figure S66 PXRD patterns of calculated, as-synthesized, and after CO_2 (195 K) sorption-treated **TIFSIX-25-Cu**.

11.2 M'FSIX-3-M

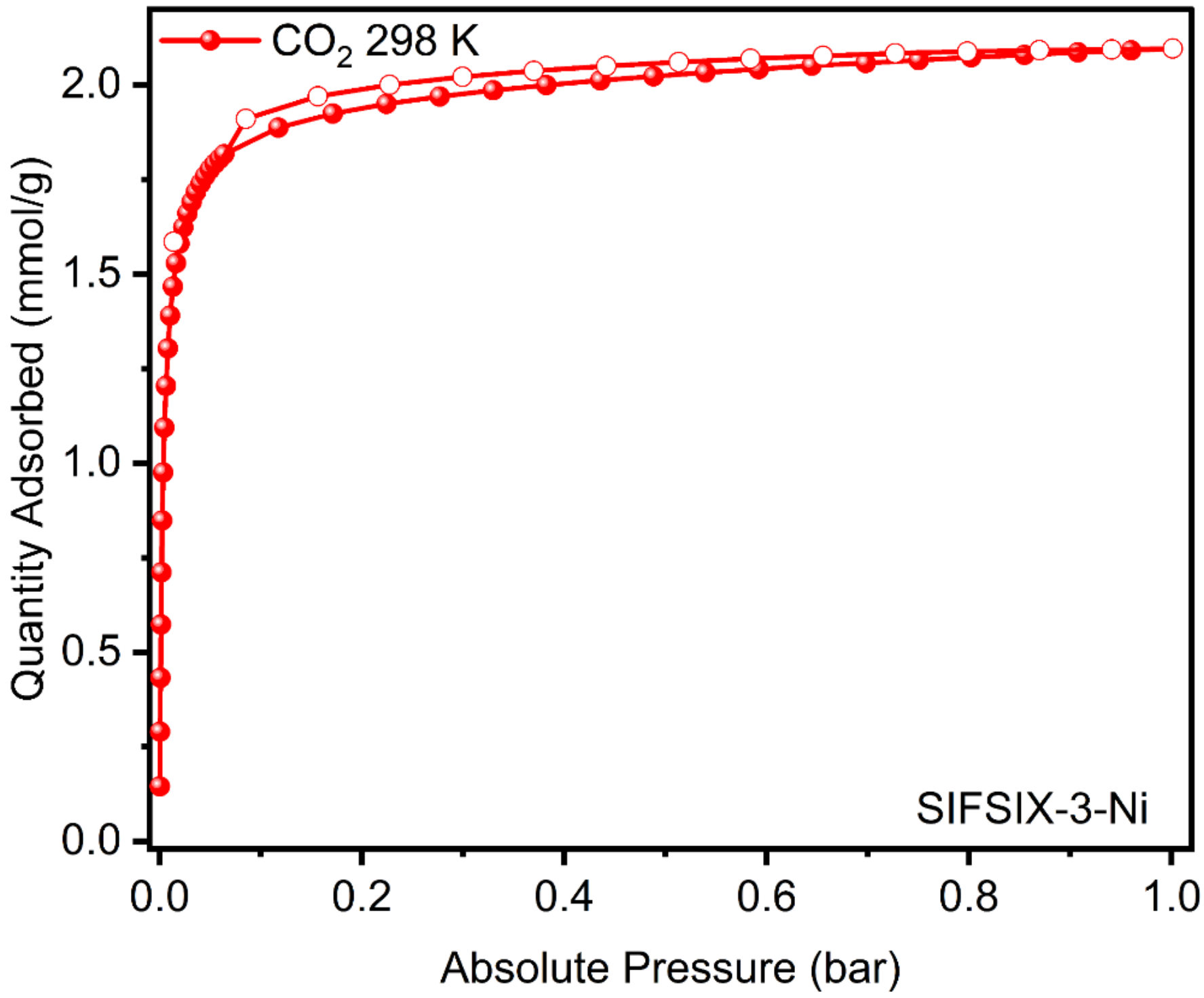

Figure S67 CO_2 adsorption isotherm of **SIFSIX-3-Ni** measured at 298 K.

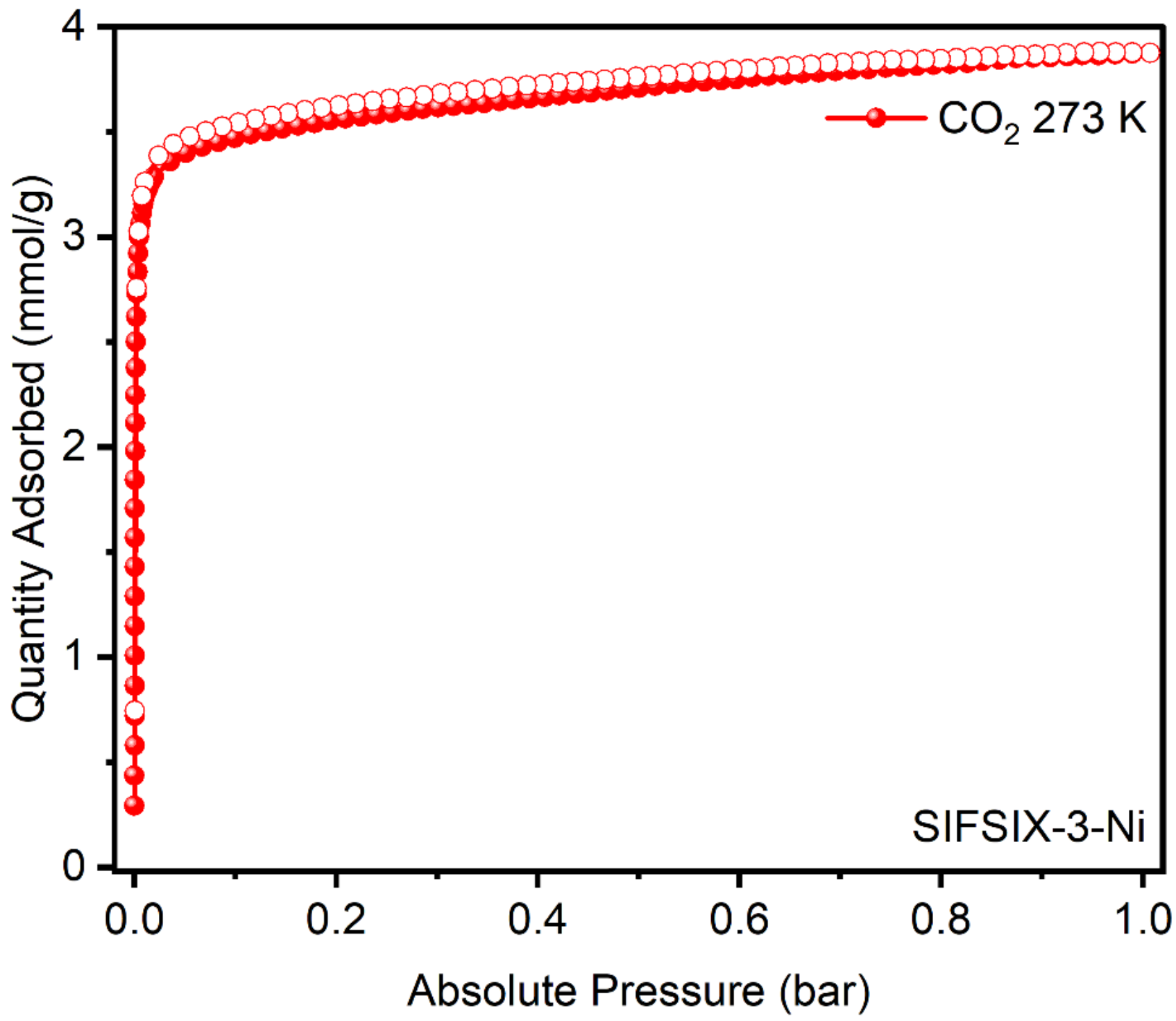

Figure S68 CO_2 adsorption isotherm of **SIFSIX-3-Ni** measured at 273 K.

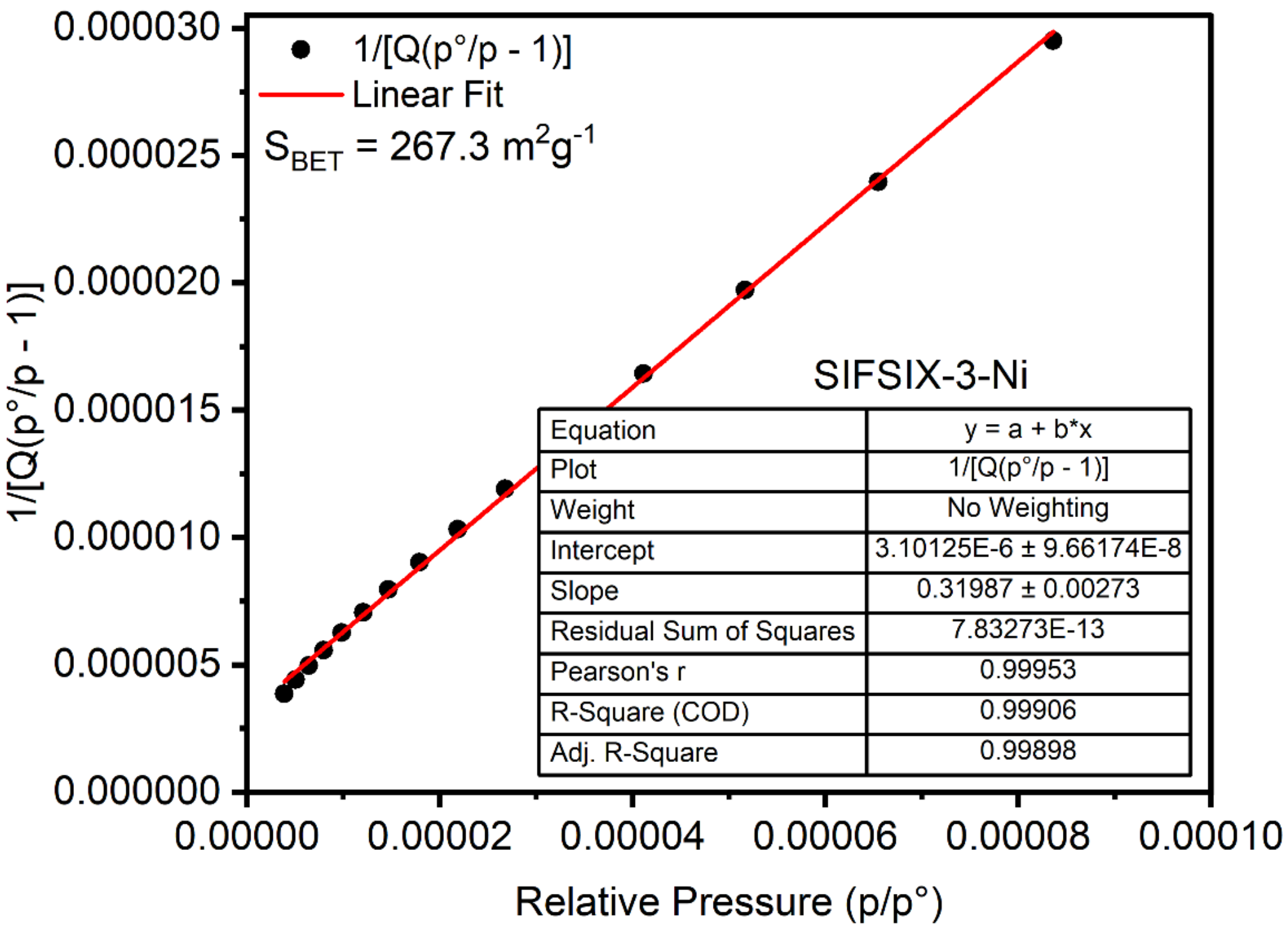

Figure S69 BET plot derived from CO_2 adsorption data for **SIFSIX-3-Ni** (at 273 K) showing the linear fitting region used for surface area determination.

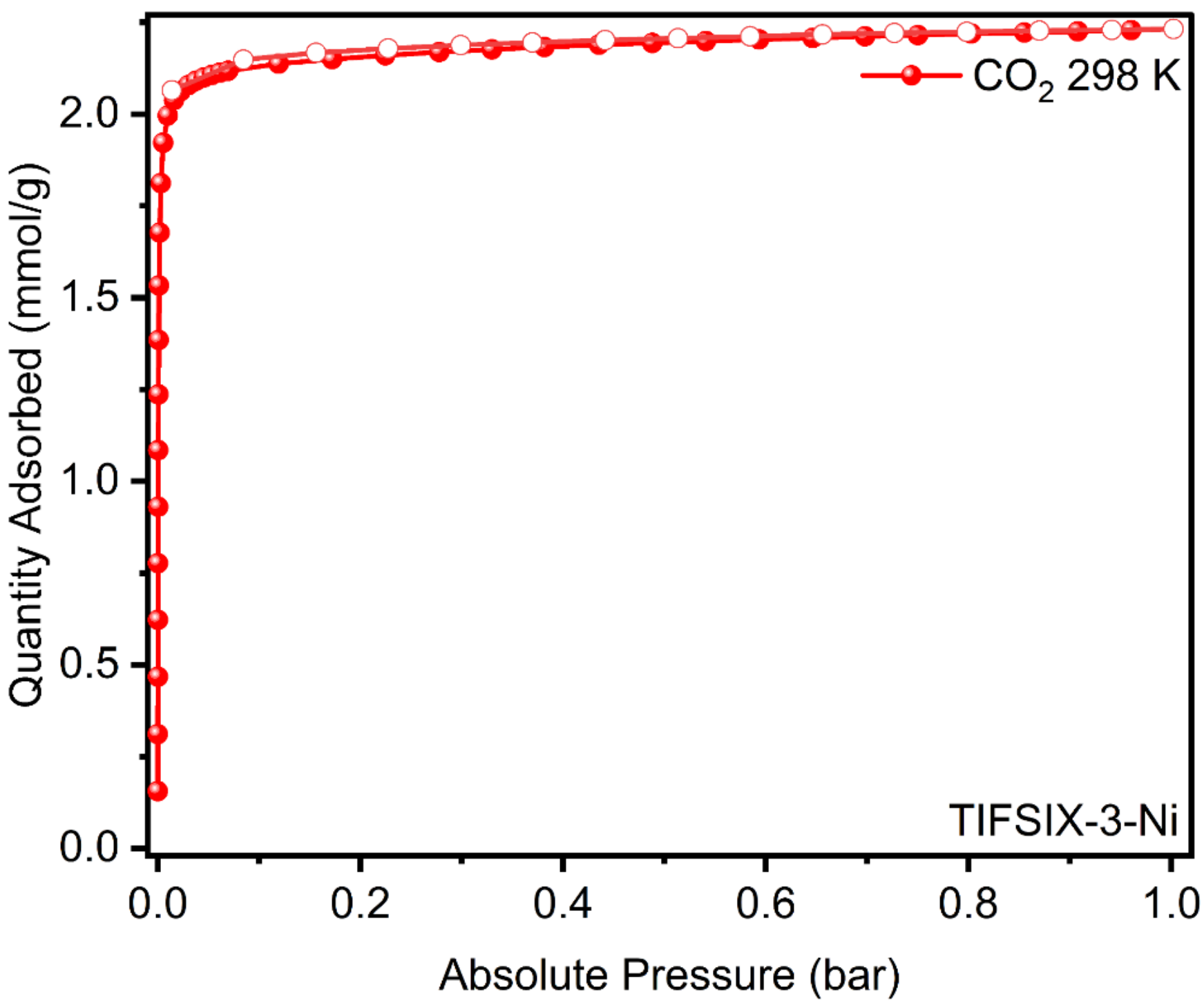

Figure S70 CO_2 adsorption isotherm of **TIFSIX-3-Ni** measured at 298 K.

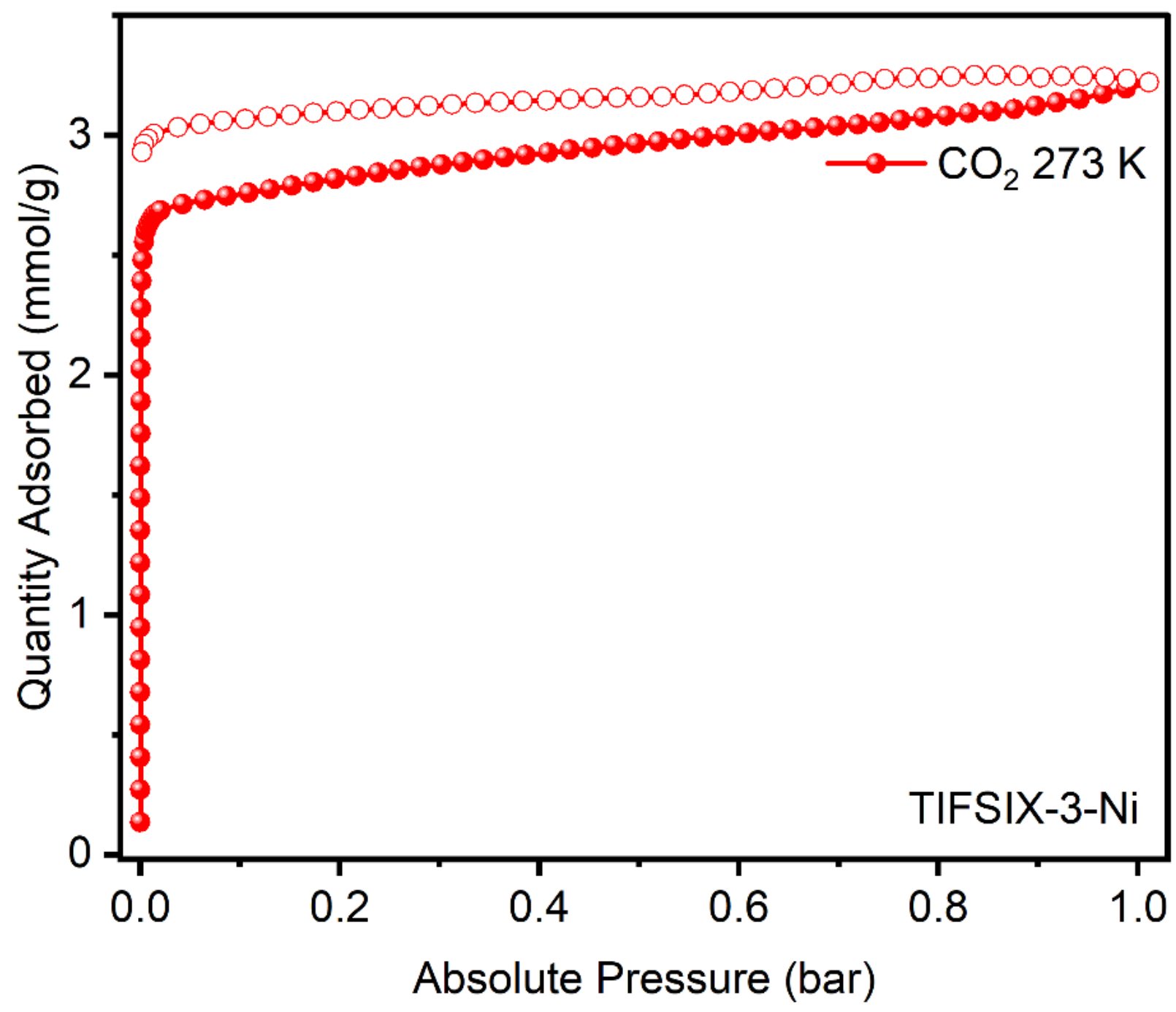

Figure S71 CO_2 adsorption isotherm of **TIFSIX-3-Ni** measured at 273 K.

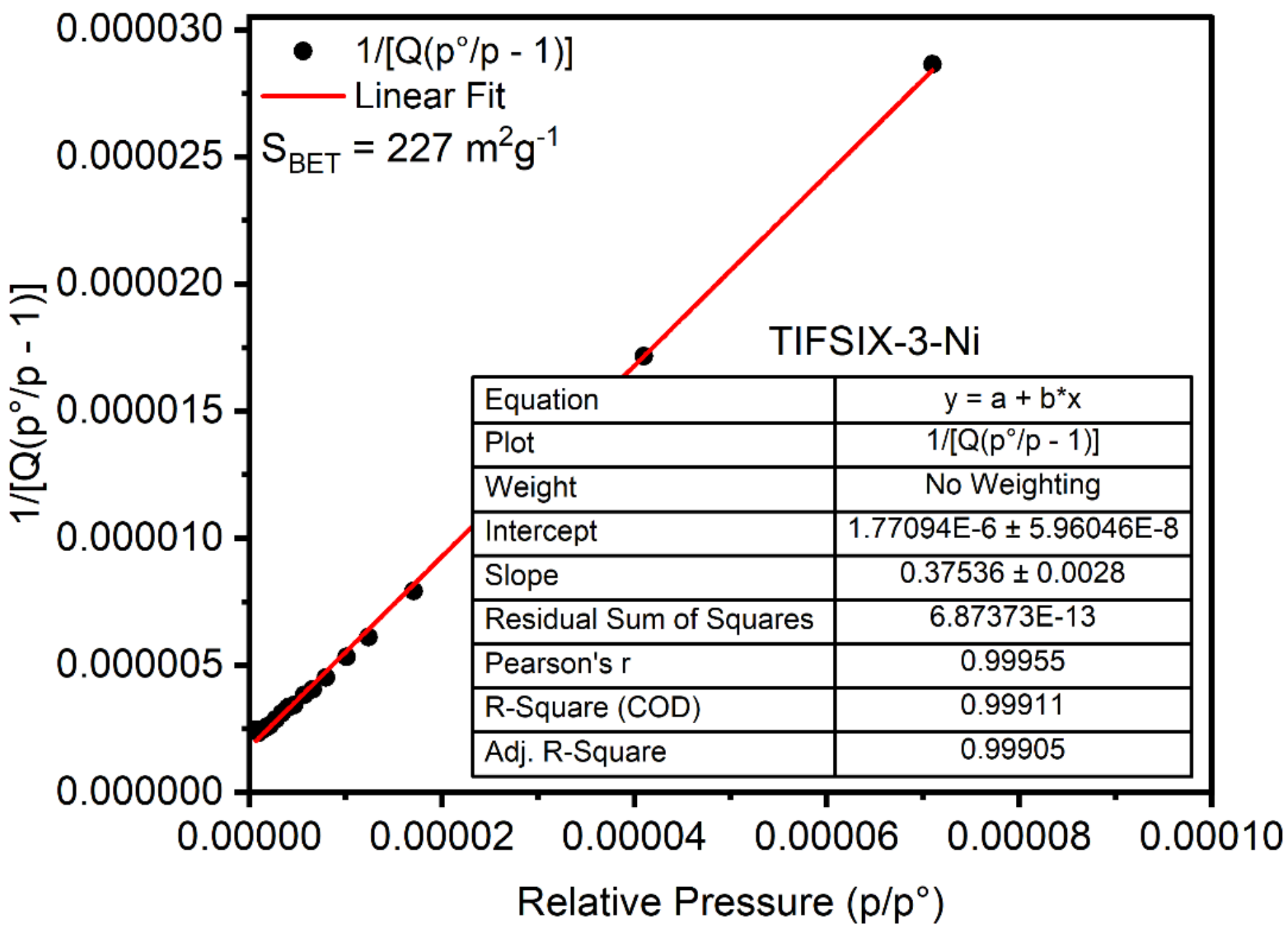

Figure S72 BET plot derived from CO_2 adsorption data for **TIFSIX-3-Ni** (at 273 K) showing the linear fitting region used for surface area determination.

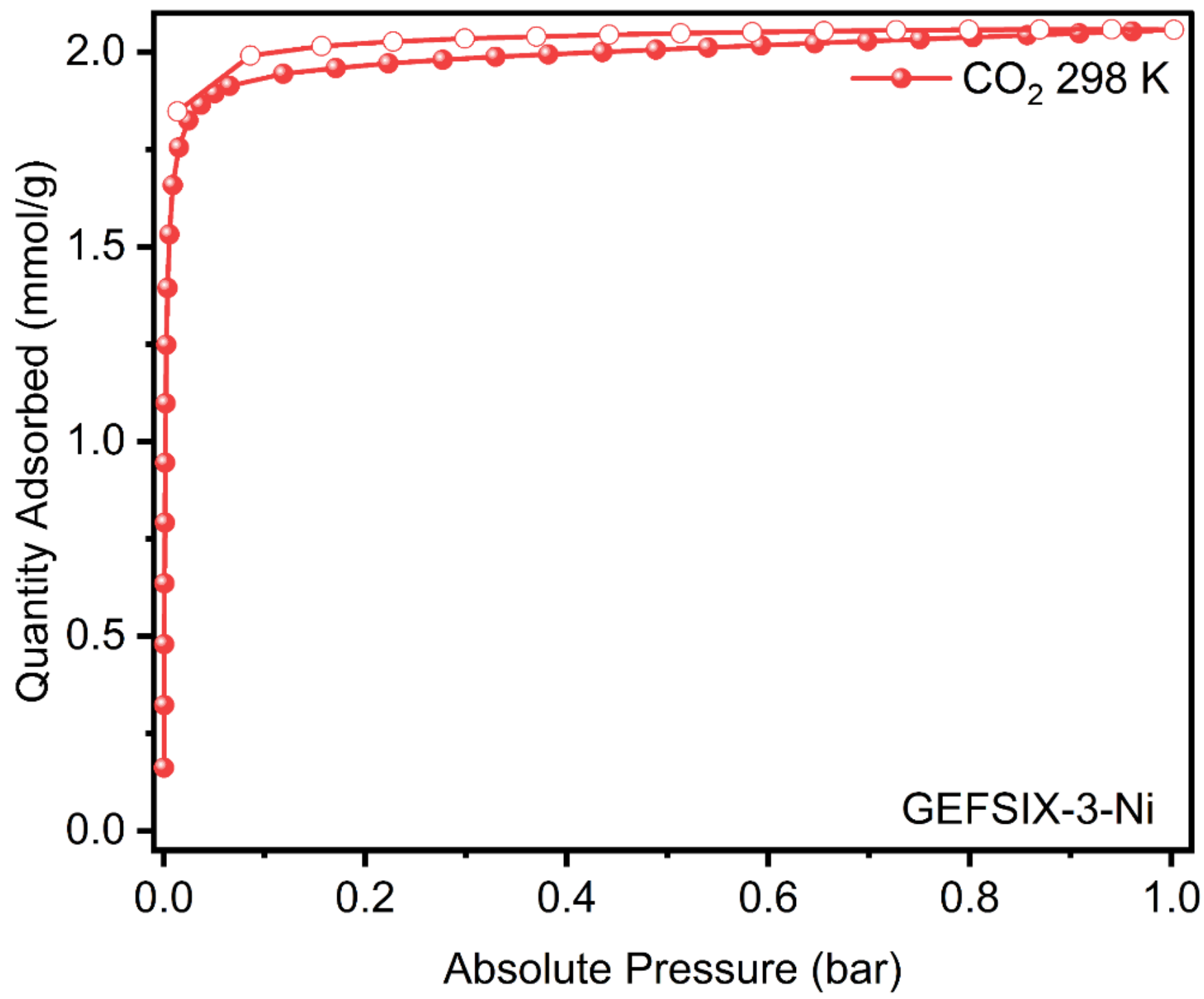

Figure S73 CO_2 adsorption isotherm of **GEFSIX-3-Ni** measured at 298 K.

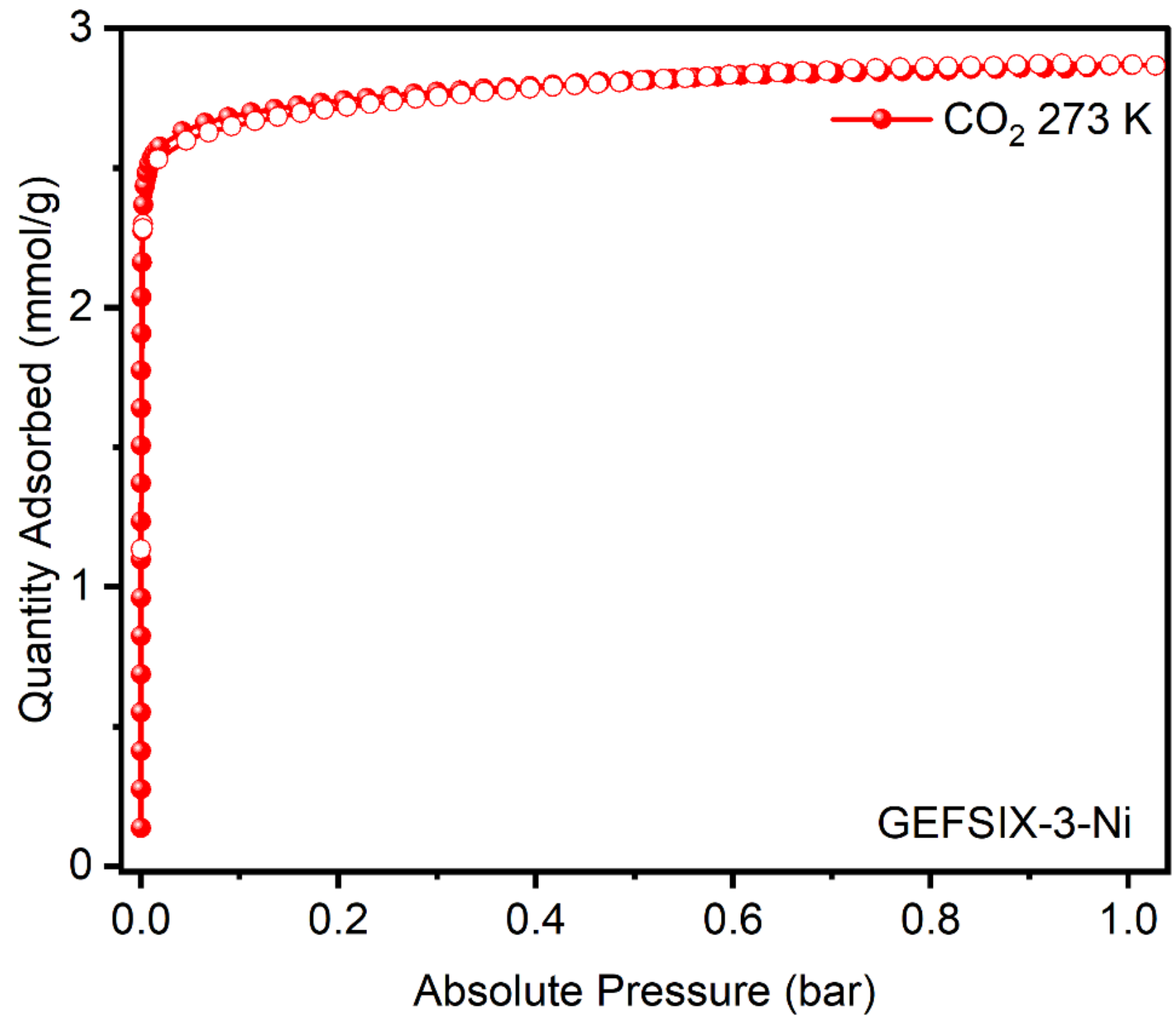

Figure S74 CO_2 adsorption isotherm of **GEFSIX-3-Ni** measured at 273 K.

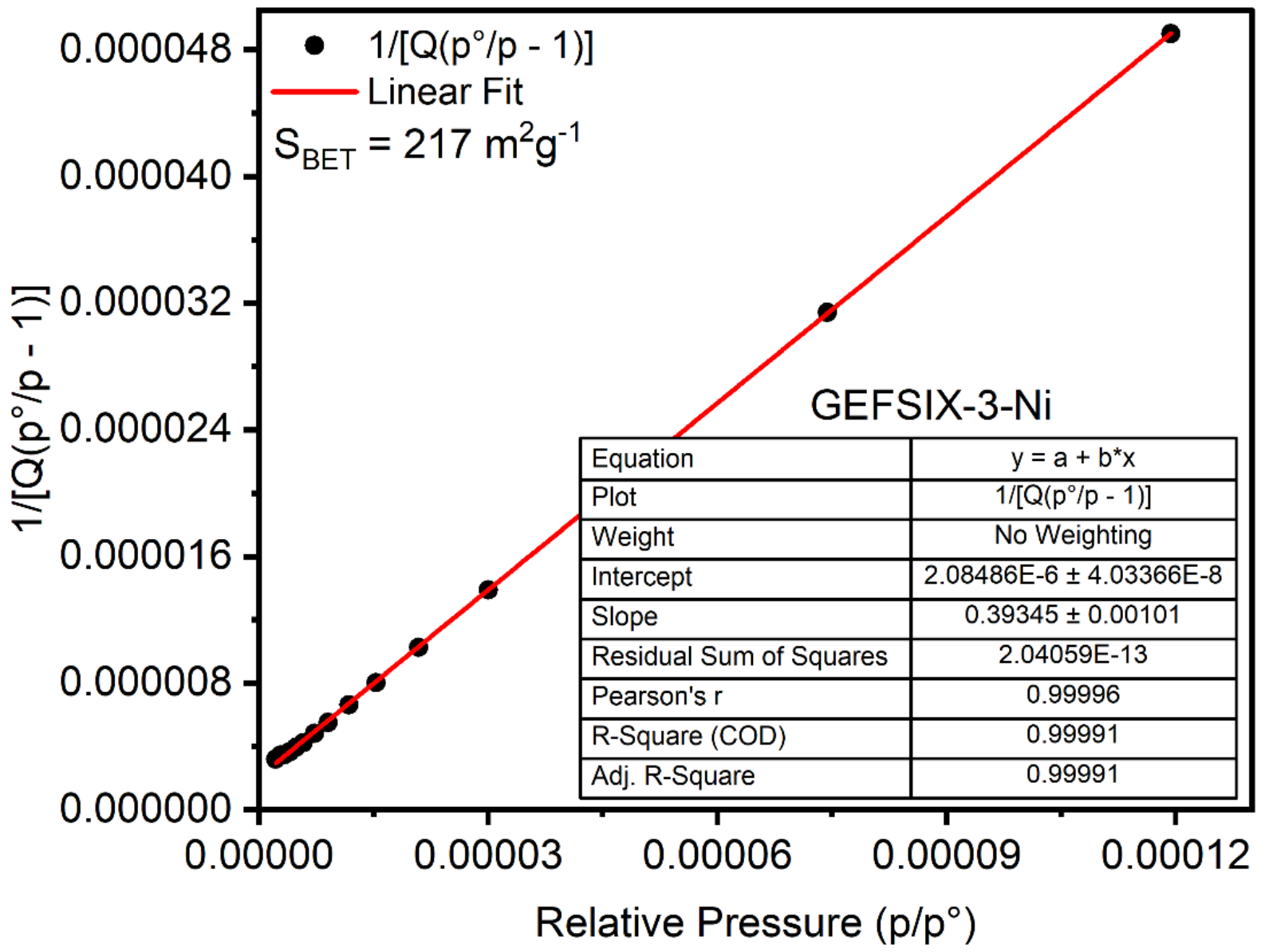

Figure S75 BET plot derived from CO_2 adsorption data for **GEFSIX-3-Ni** (at 273 K) showing the linear fitting region used for surface area determination.

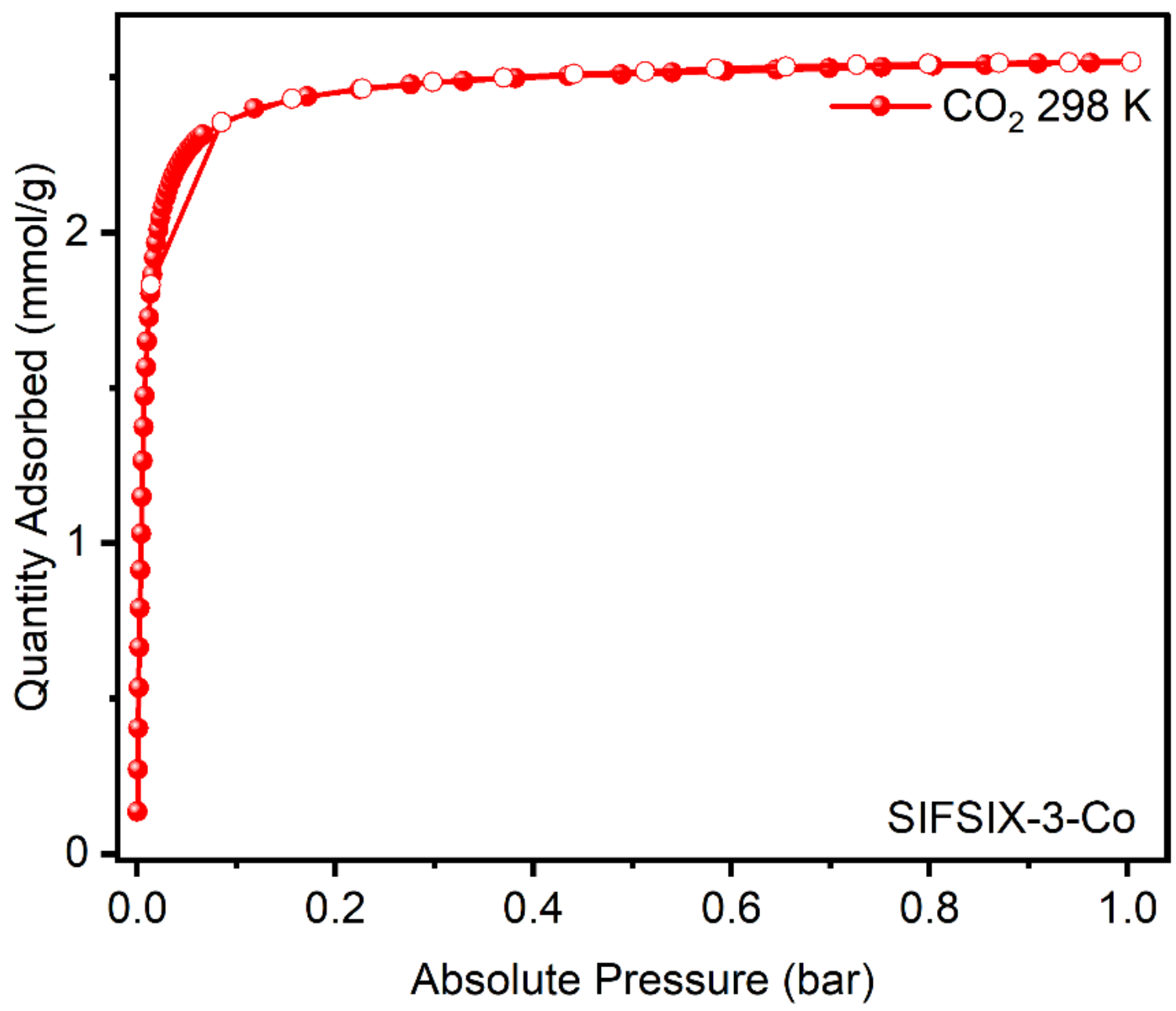

Figure S76 CO_2 adsorption isotherm of **SIFSIX-3-Co** measured at 298 K.

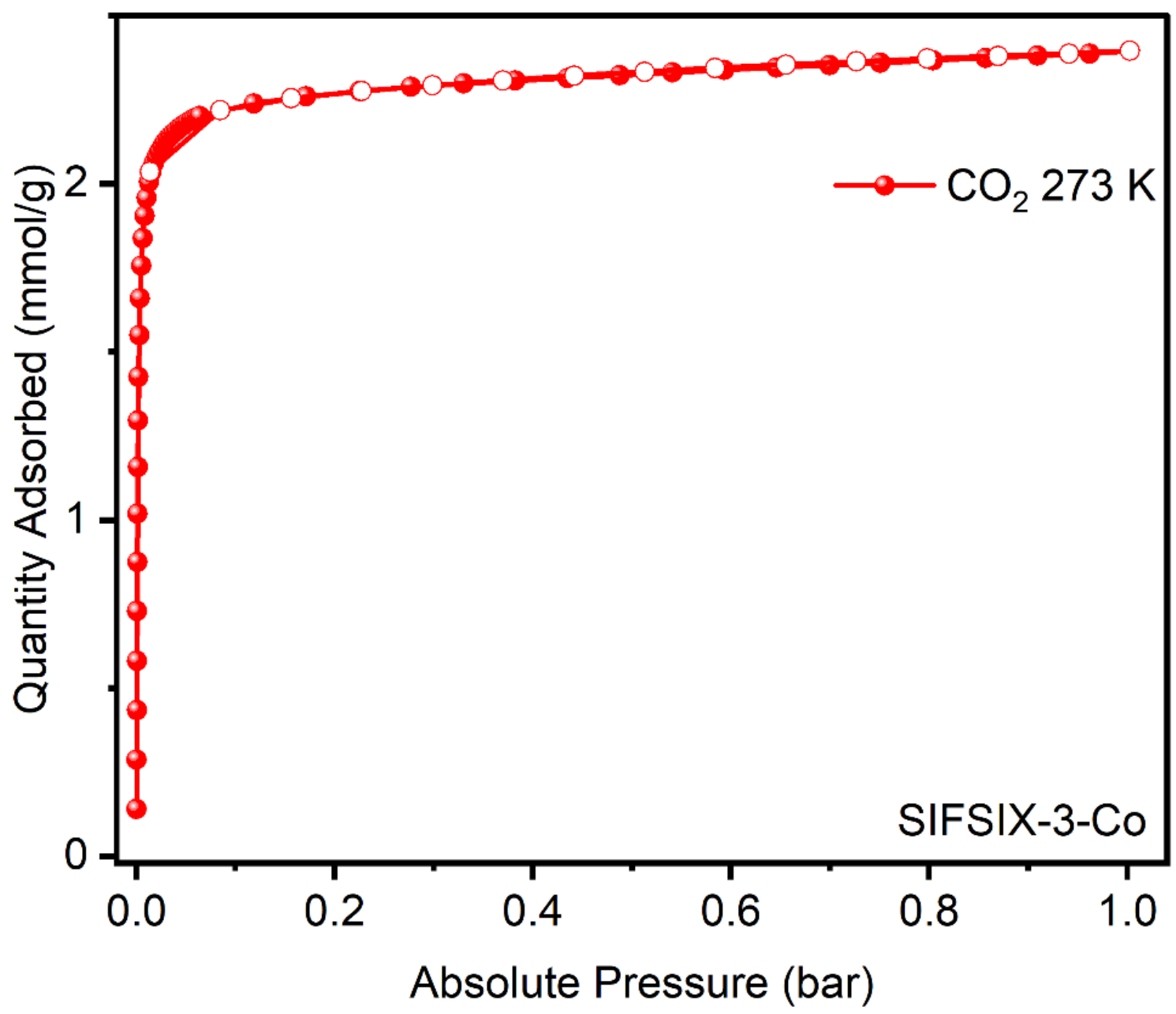

Figure S77 CO_2 adsorption isotherm of **SIFSIX-3-Co** measured at 273 K.

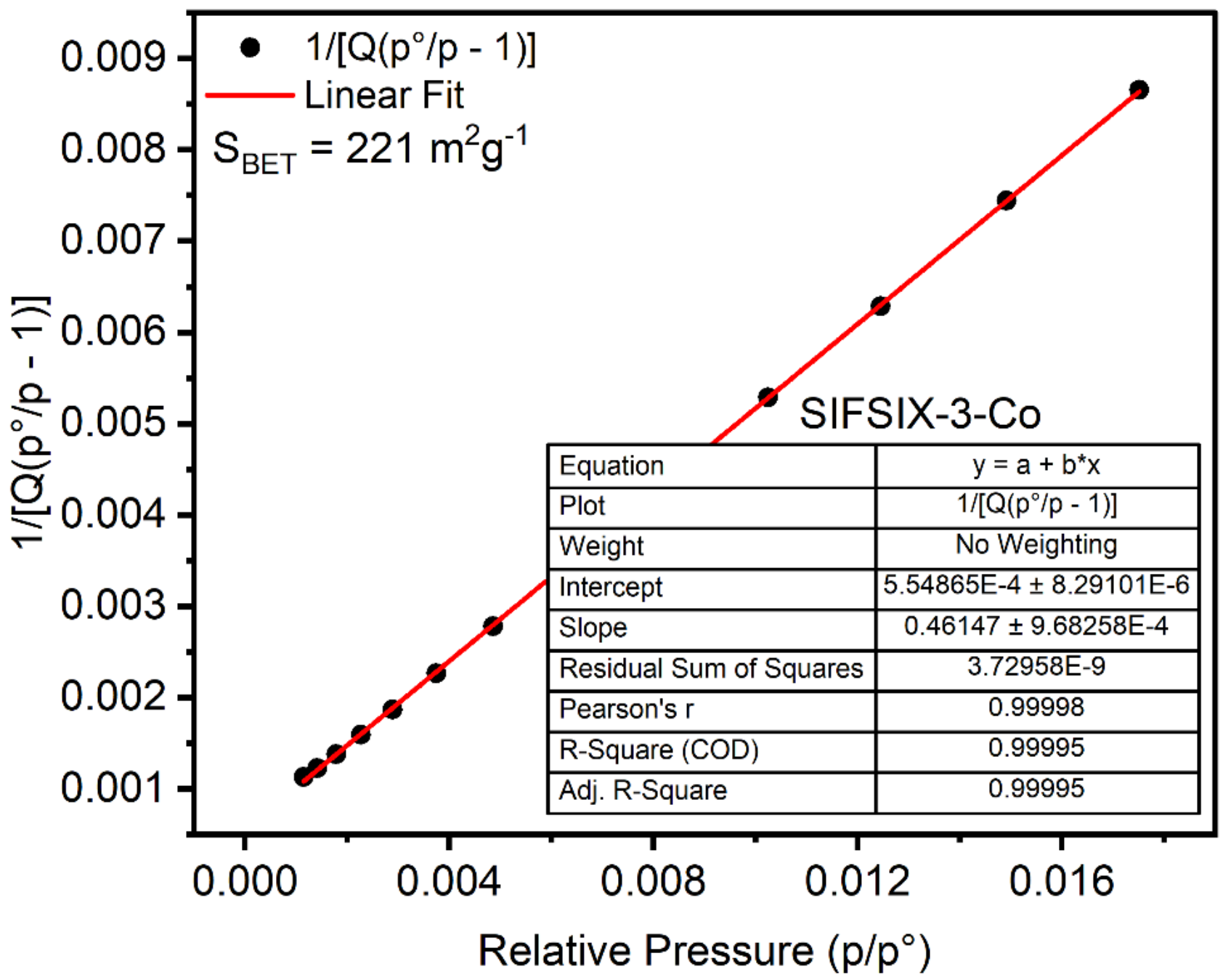

Figure S78 BET plot derived from CO_2 adsorption data for **SIFSIX-3-Co** (at 273 K) showing the linear fitting region used for surface area determination.

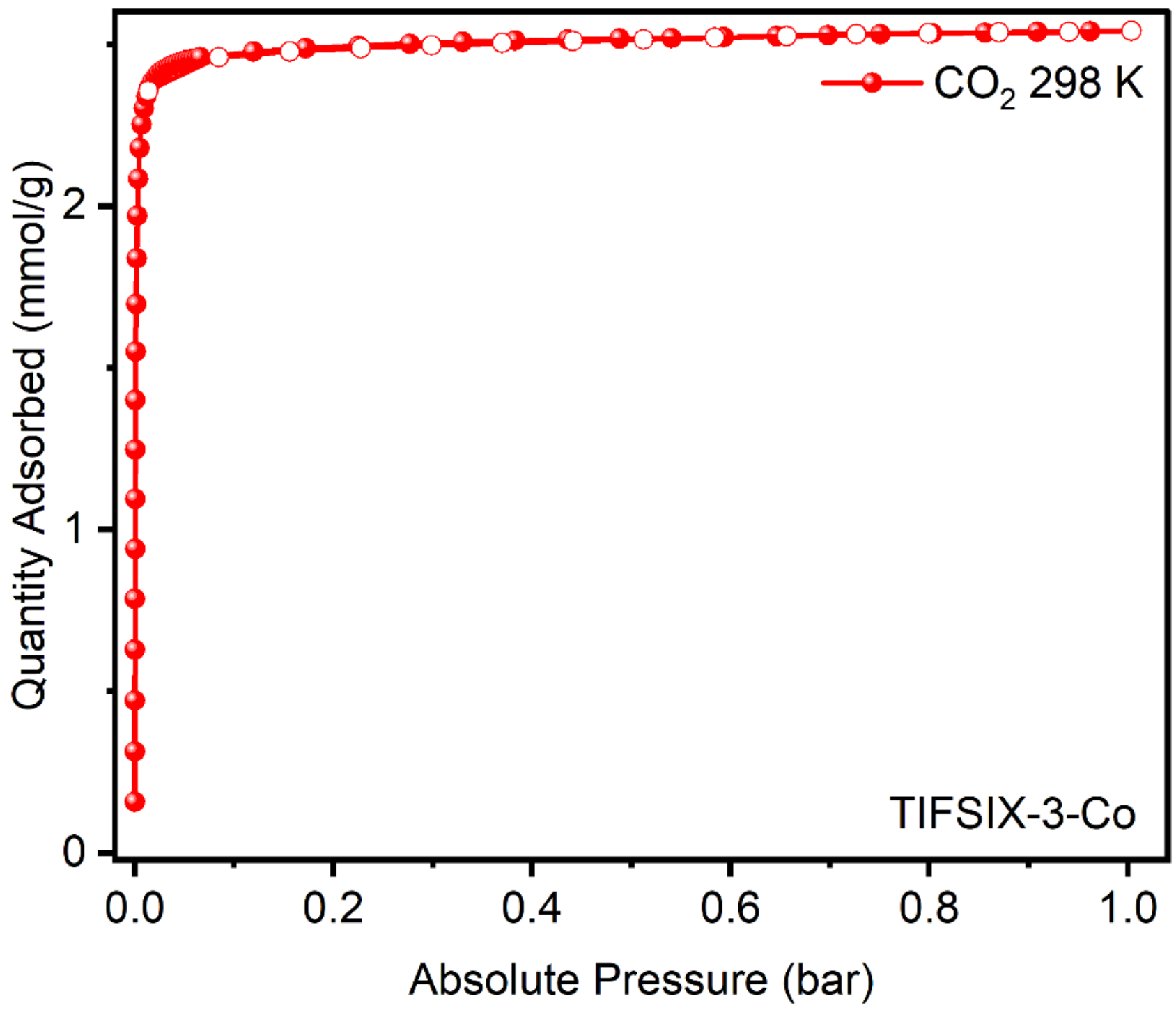

Figure S79 CO_2 adsorption isotherm of **TIFSIX-3-Co** measured at 298 K.

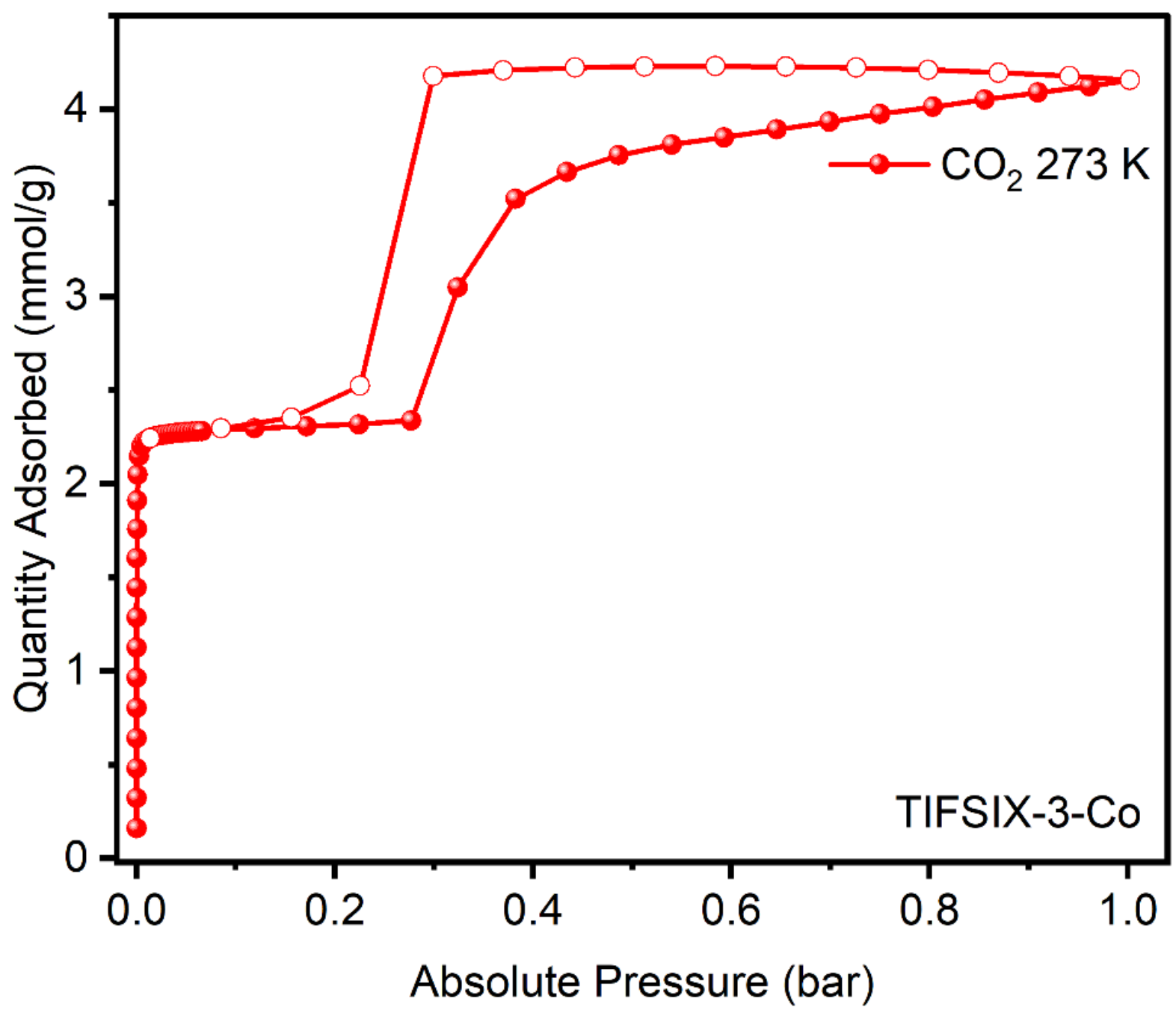

Figure S80 CO_2 adsorption isotherm of **TIFSIX-3-Co** measured at 273 K.

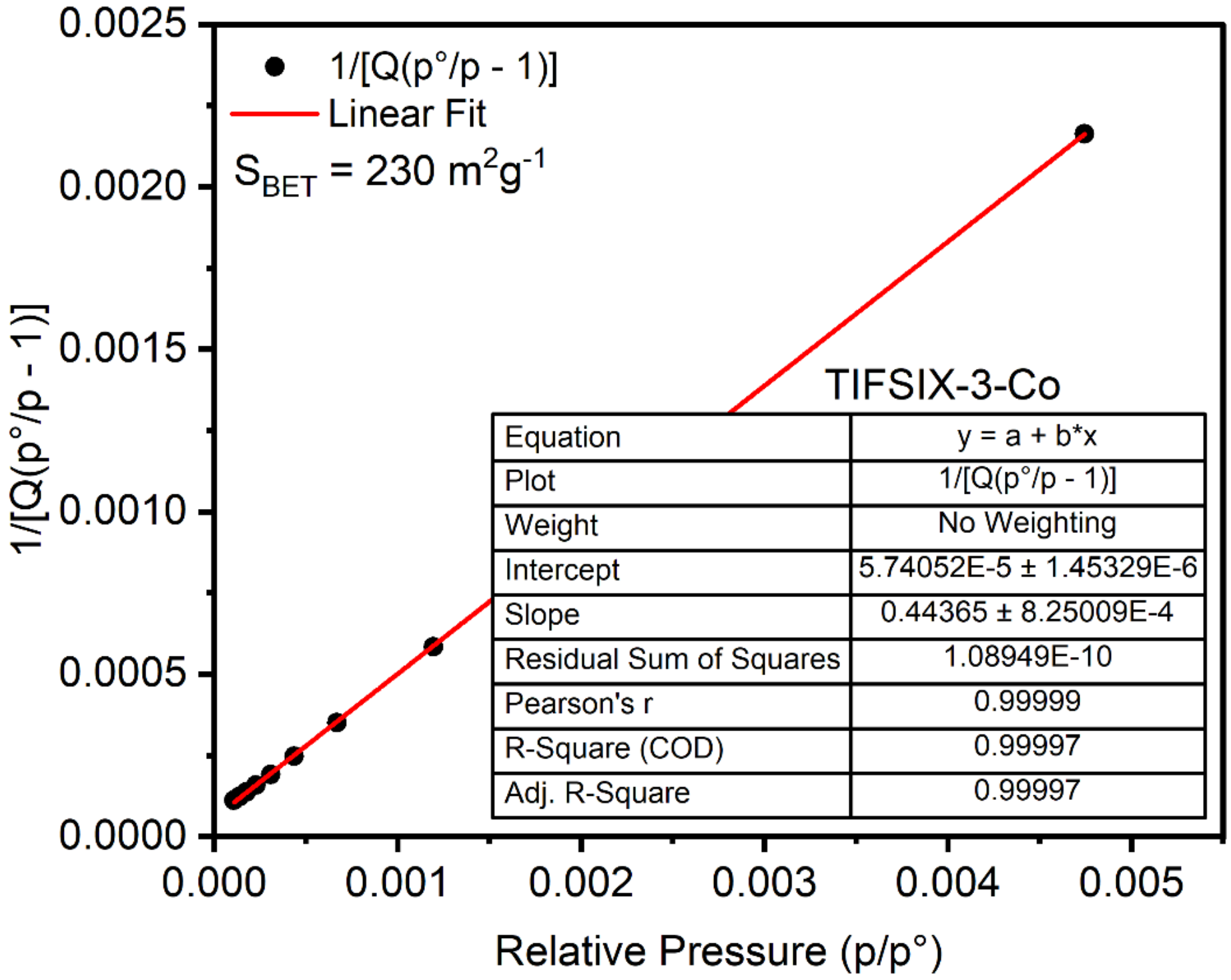

Figure S81 BET plot derived from CO_2 adsorption data for **TIFSIX-3-Co** (at 273 K) showing the linear fitting region used for surface area determination.

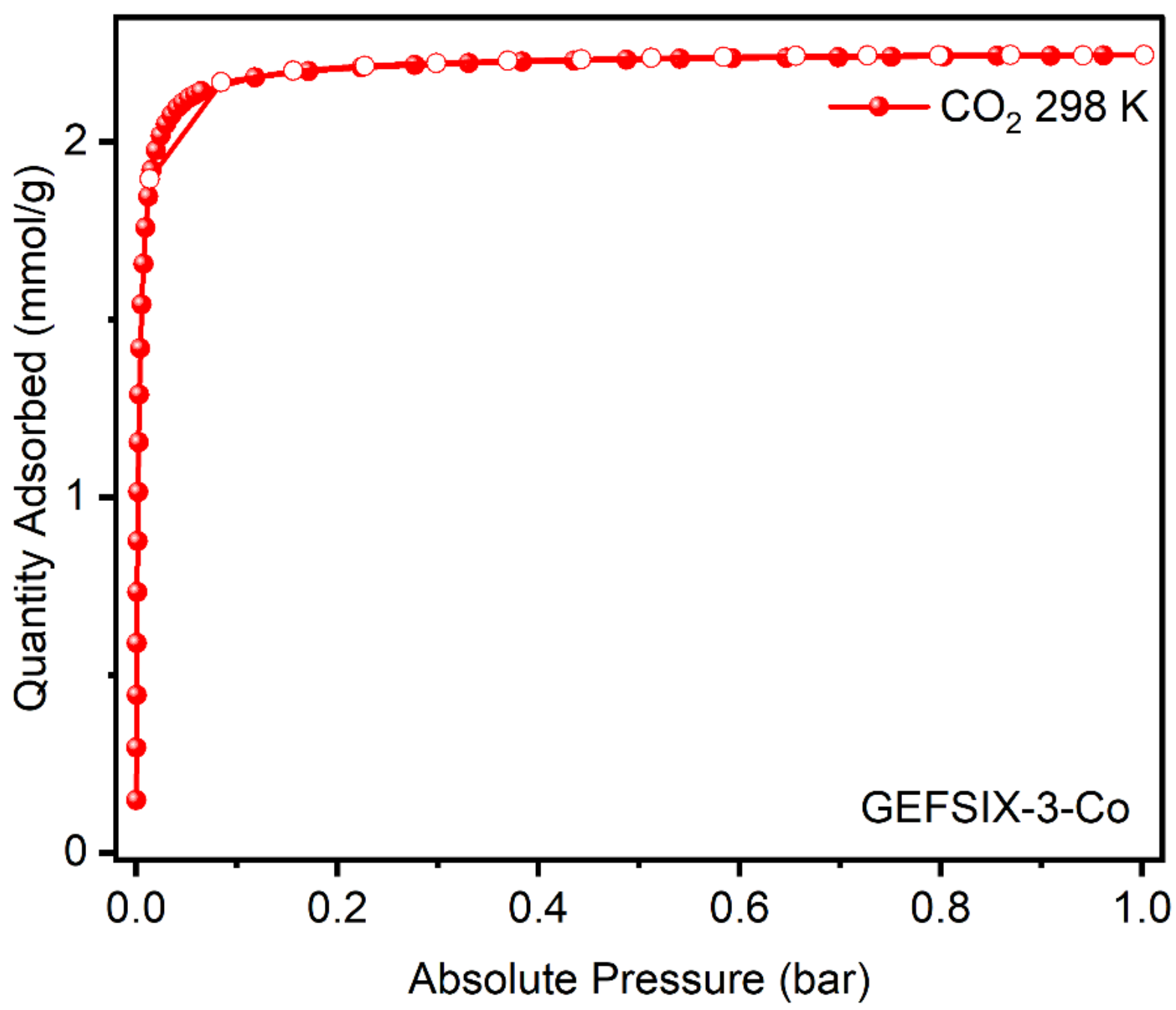

Figure S82 CO_2 adsorption isotherm of **GEFSIX-3-Co** measured at 298 K.

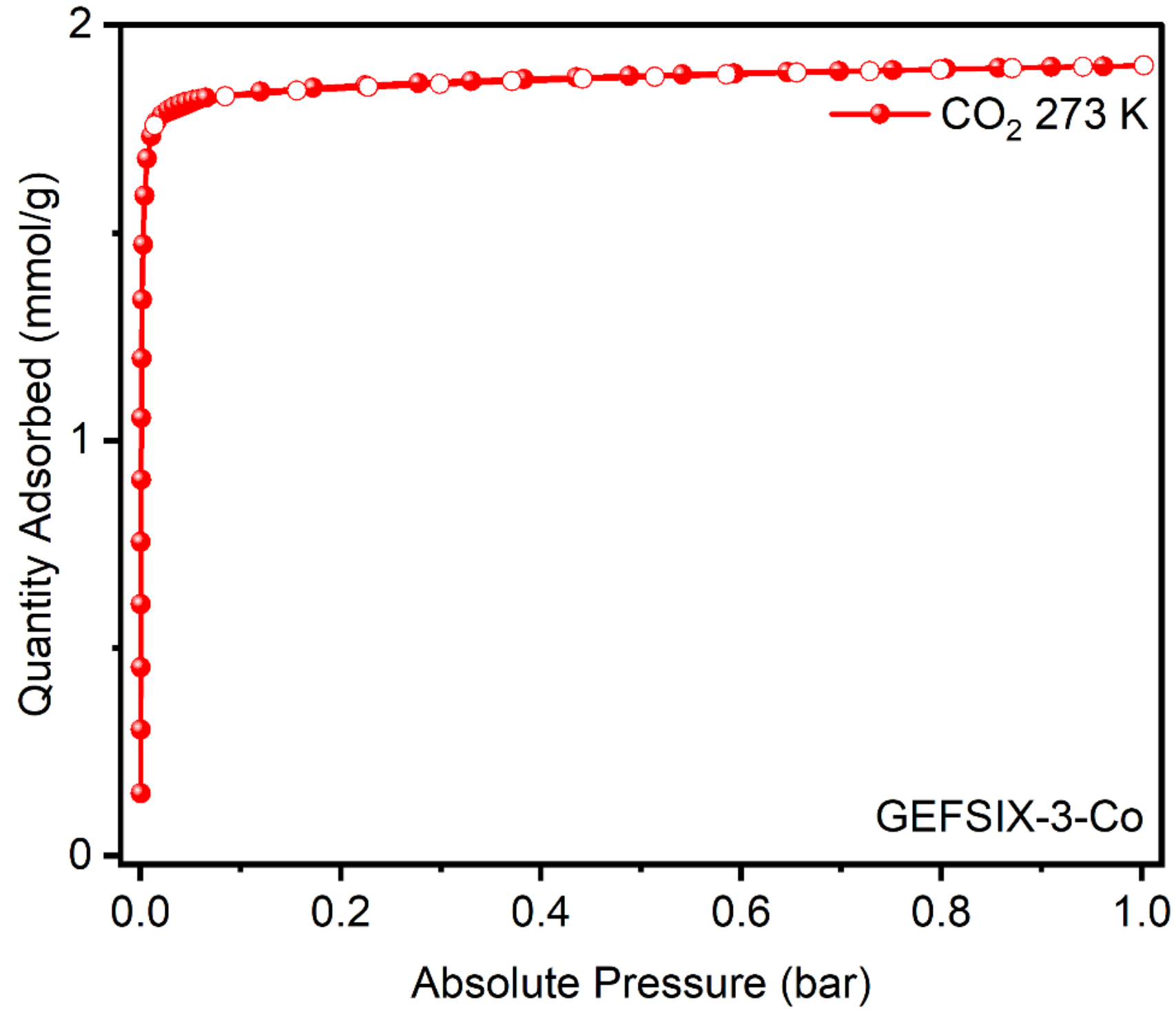

Figure S83 CO_2 adsorption isotherm of **GEFSIX-3-Co** measured at 273 K.

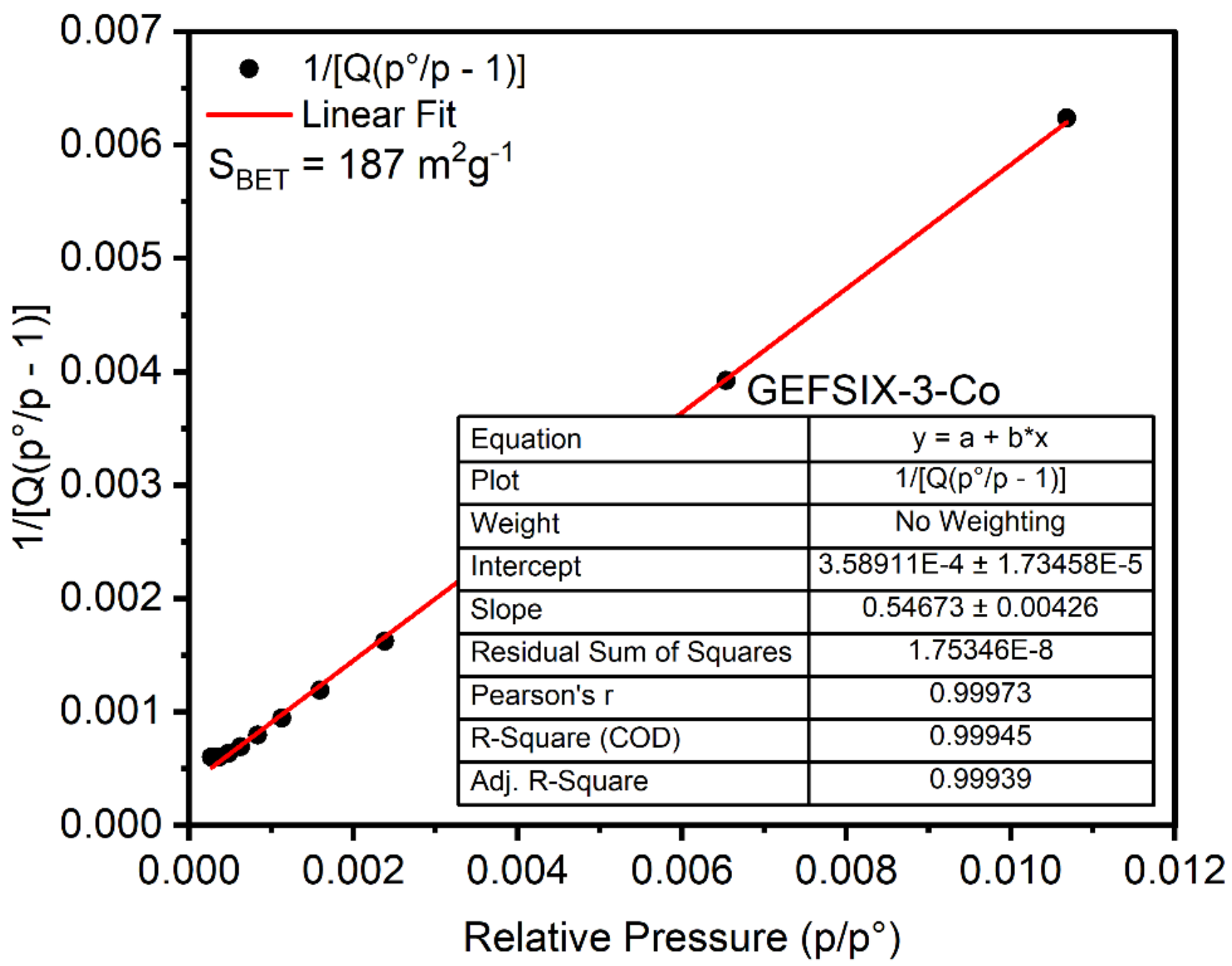

Figure S84 BET plot derived from CO_2 adsorption data for **GEFSIX-3-Co** (at 273 K) showing the linear fitting region used for surface area determination.

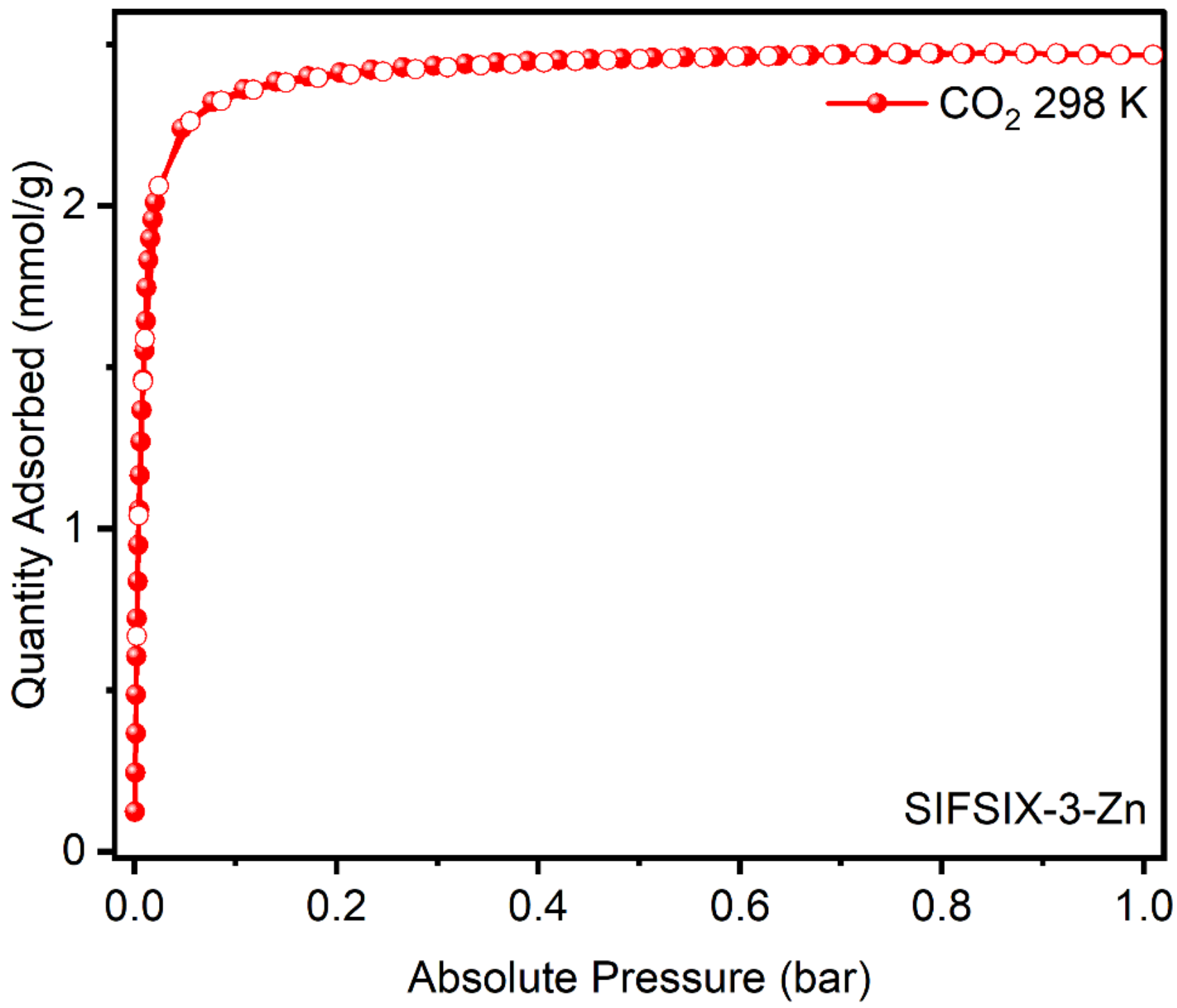

Figure S85 CO_2 adsorption isotherm of **SIFSIX-3-Zn** measured at 298 K.

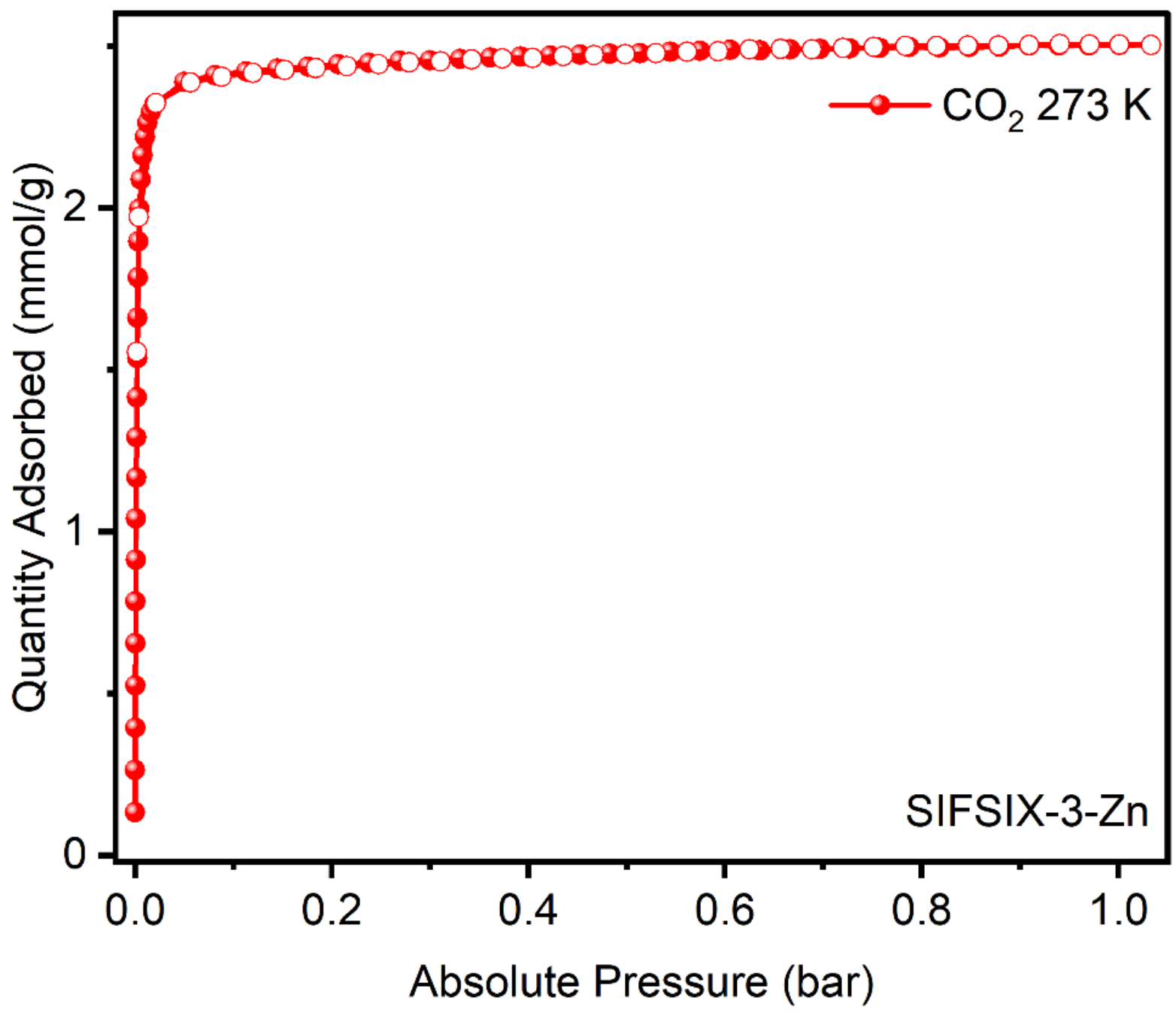

Figure S86 CO_2 adsorption isotherm of **SIFSIX-3-Zn** measured at 273 K.

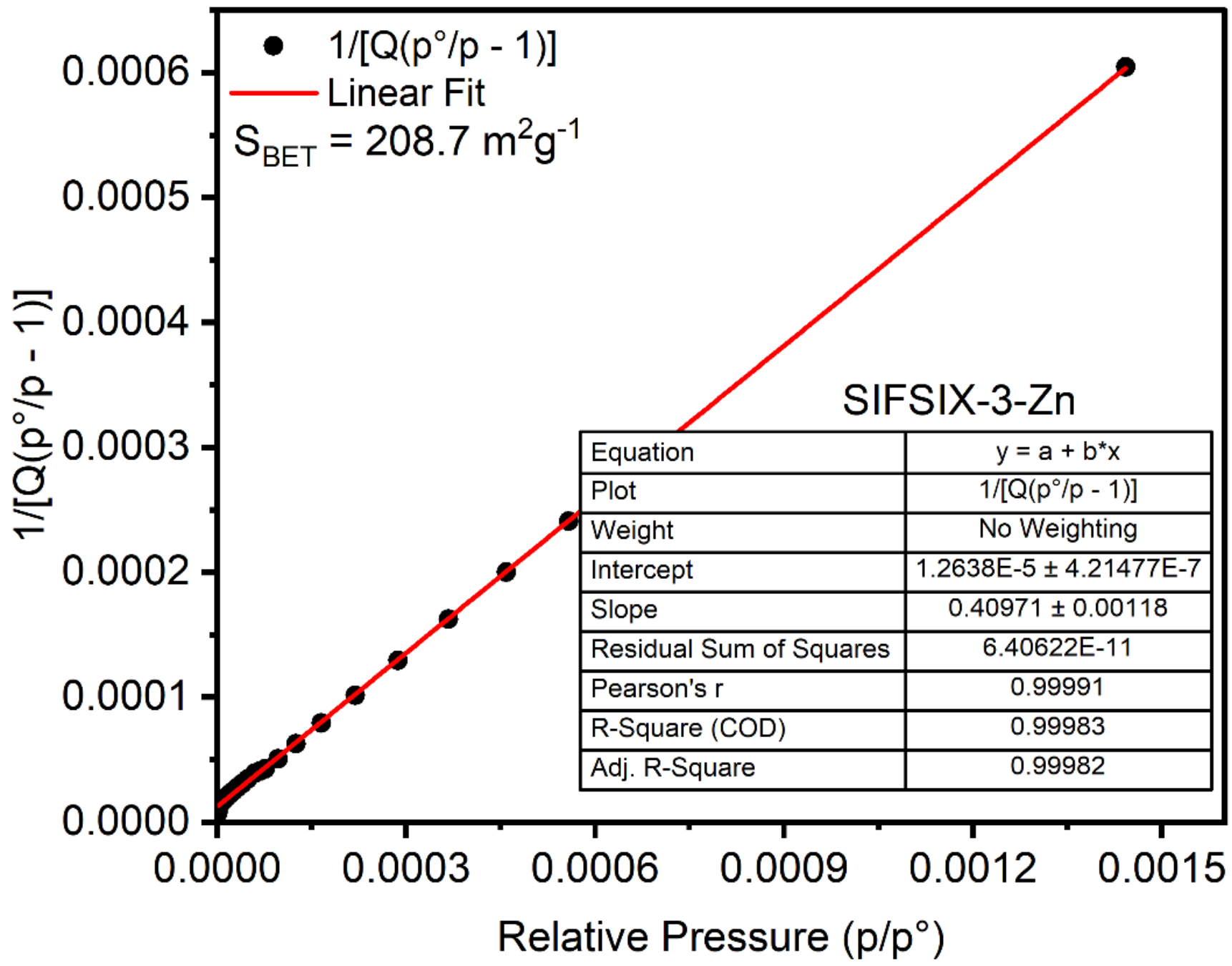

Figure S87 BET plot derived from CO_2 adsorption data for **SIFSIX-3-Zn** (at 273 K) showing the linear fitting region used for surface area determination.

Table S7 Comparison of BET surface areas obtained in this work with literature-reported values for **pcu-M′FSIX-3-M** based on CO_2 sorption analysis.

Compound	Measured BET surface area ($m^2\ g^{-1}$)	Temperature (K)	Reported BET surface area ($m^2\ g^{-1}$)	Temperature (K)	Reference
**SIFSIX-3-Ni**	267.3	273	220	298	[20]
**TIFSIX-3-Ni**	227	273	200	298	[20]
**GEFSIX-3-Ni**	217	273	221	196	[21]
**SIFSIX-3-Co**	221	273	223[a]	77	[22]
**SIFSIX-3-Zn**	208.7	273	250	298	[15]
**GEFSIX-3-Co**	187.0	273	257	196	[20]
**TIFSIX-3-Co**	230	273	260	196	[23]

[a]= from N_2 isotherm

12 Water vapor sorption

12.1 **M'FSIX-25-M** and Post-Adsorption PXRD

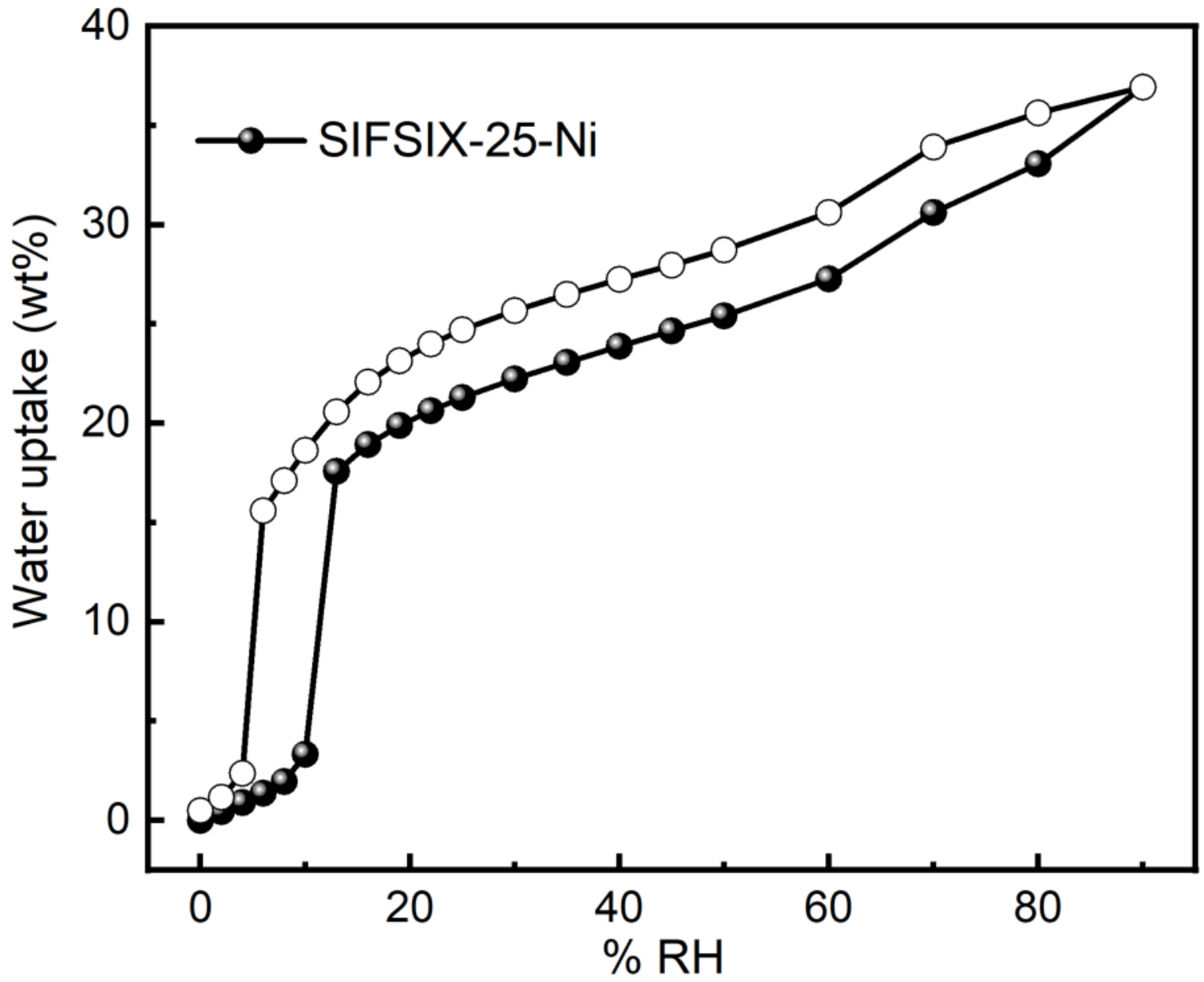

Figure S88 Water vapor adsorption (closed circles) and desorption (open circles) isotherms for **SIFSIX-25-Ni** measured at 25 °C.

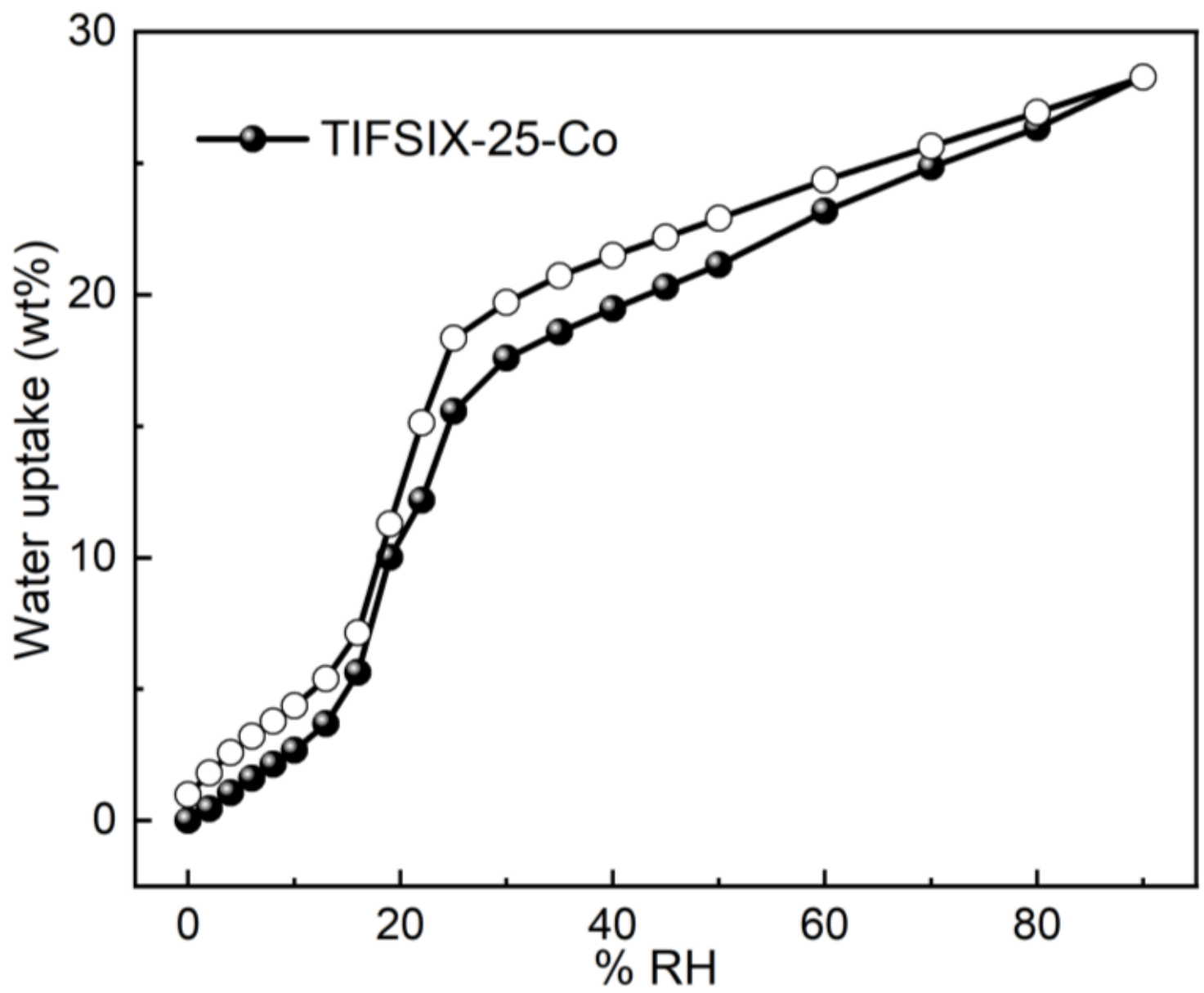

Figure S89 Water vapor adsorption (closed circles) and desorption (open circles) isotherms for **TIFSIX-25-Co** measured at 25 °C.

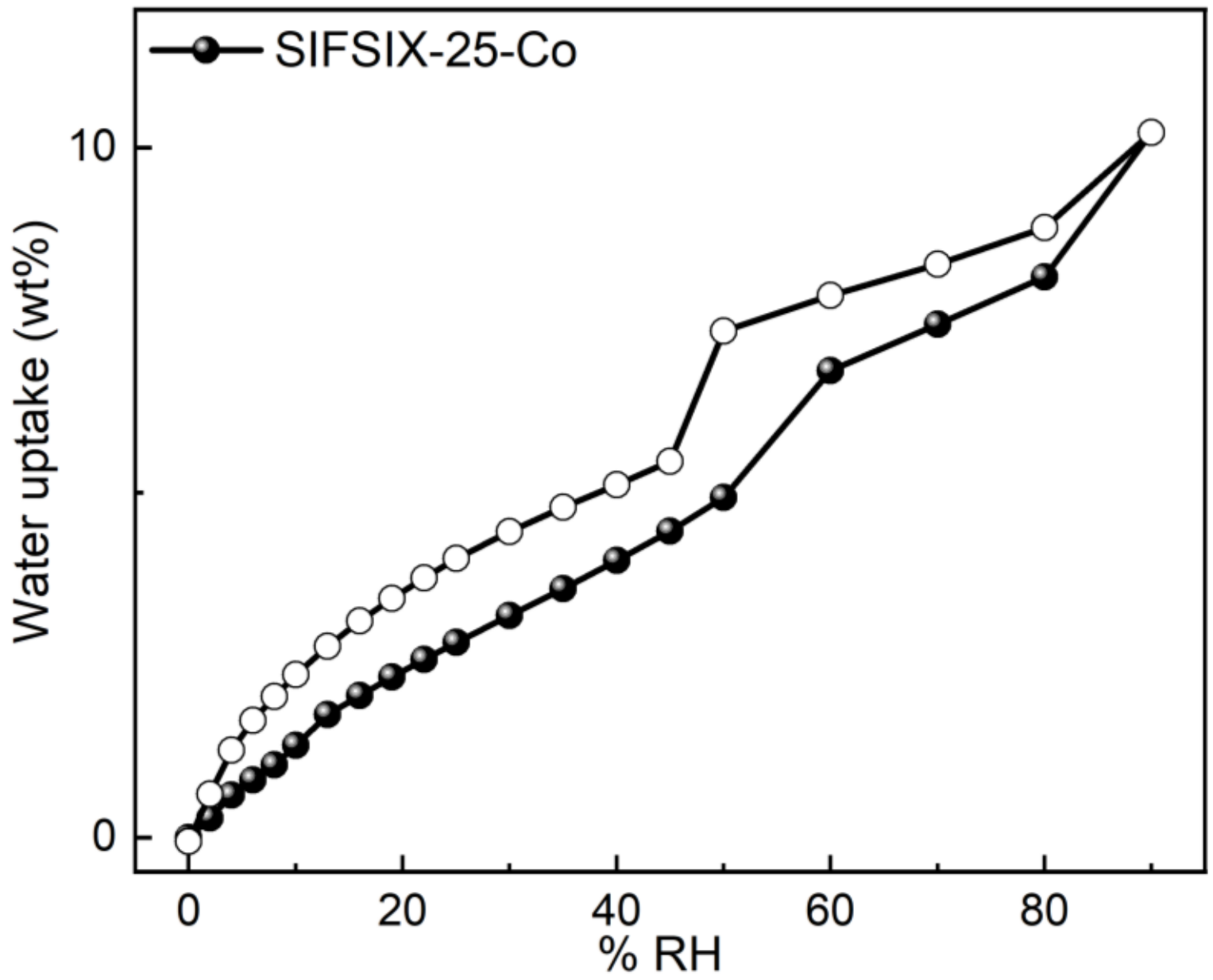

Figure S90 Water vapor adsorption (closed circles) and desorption (open circles) isotherms for **SIFSIX-25-Co** measured at 25 °C.

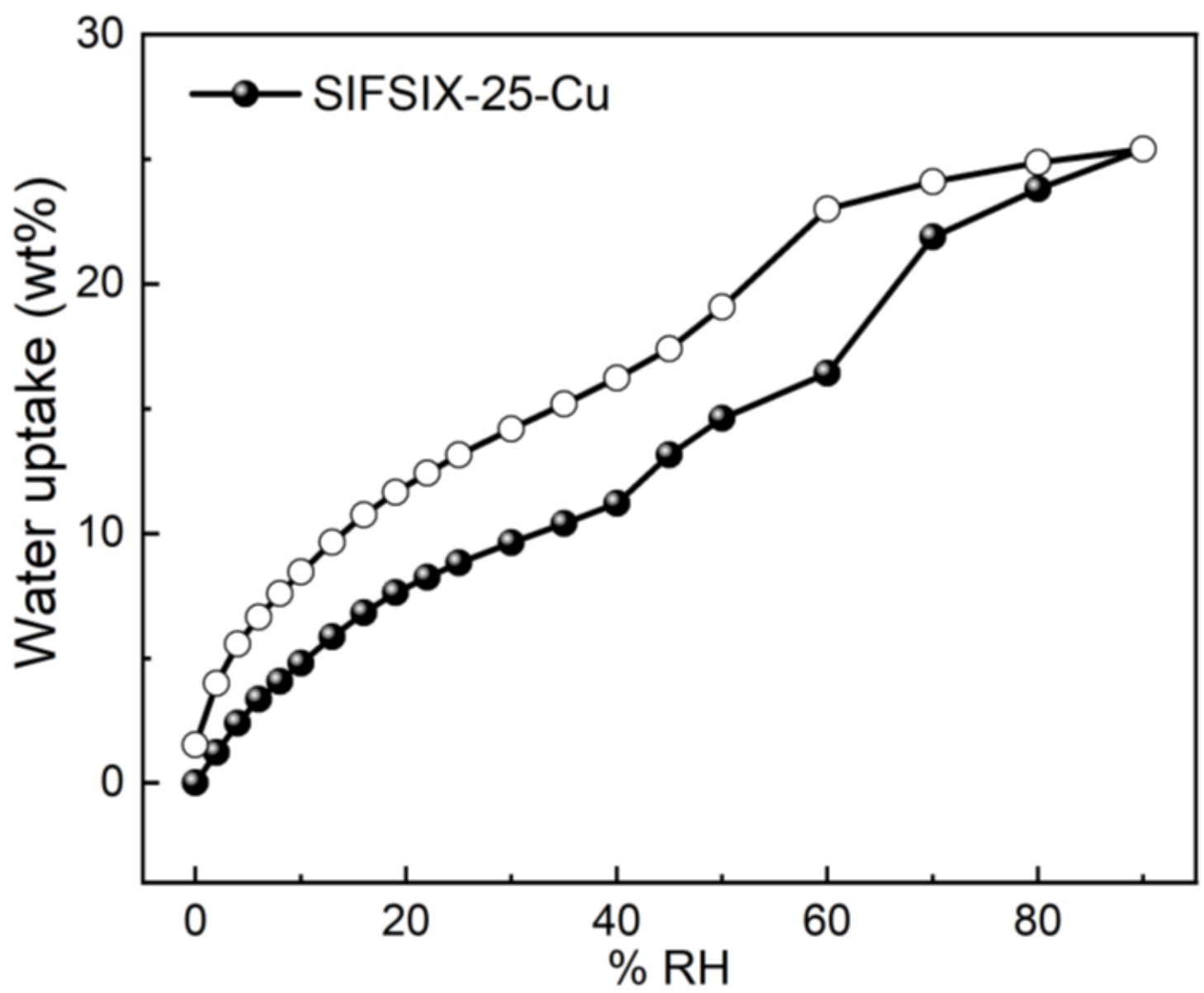

Figure S91 Water vapor adsorption (closed circles) and desorption (open circles) isotherms for **SIFSIX-25-Cu** measured at 25 °C.

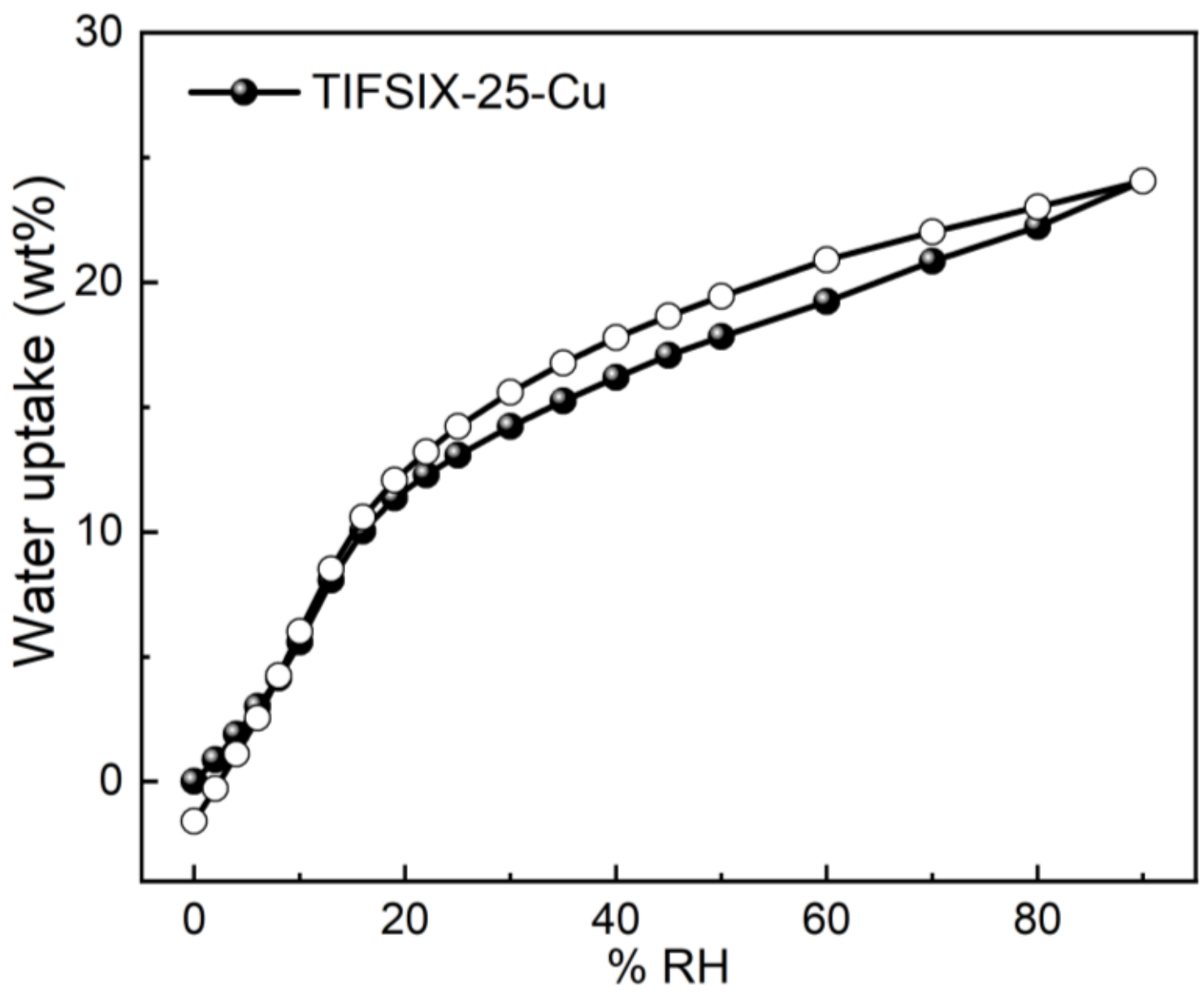

Figure S92 Water vapor adsorption (closed circles) and desorption (open circles) isotherms for **TIFSIX-25-Cu** measured at 25 °C.

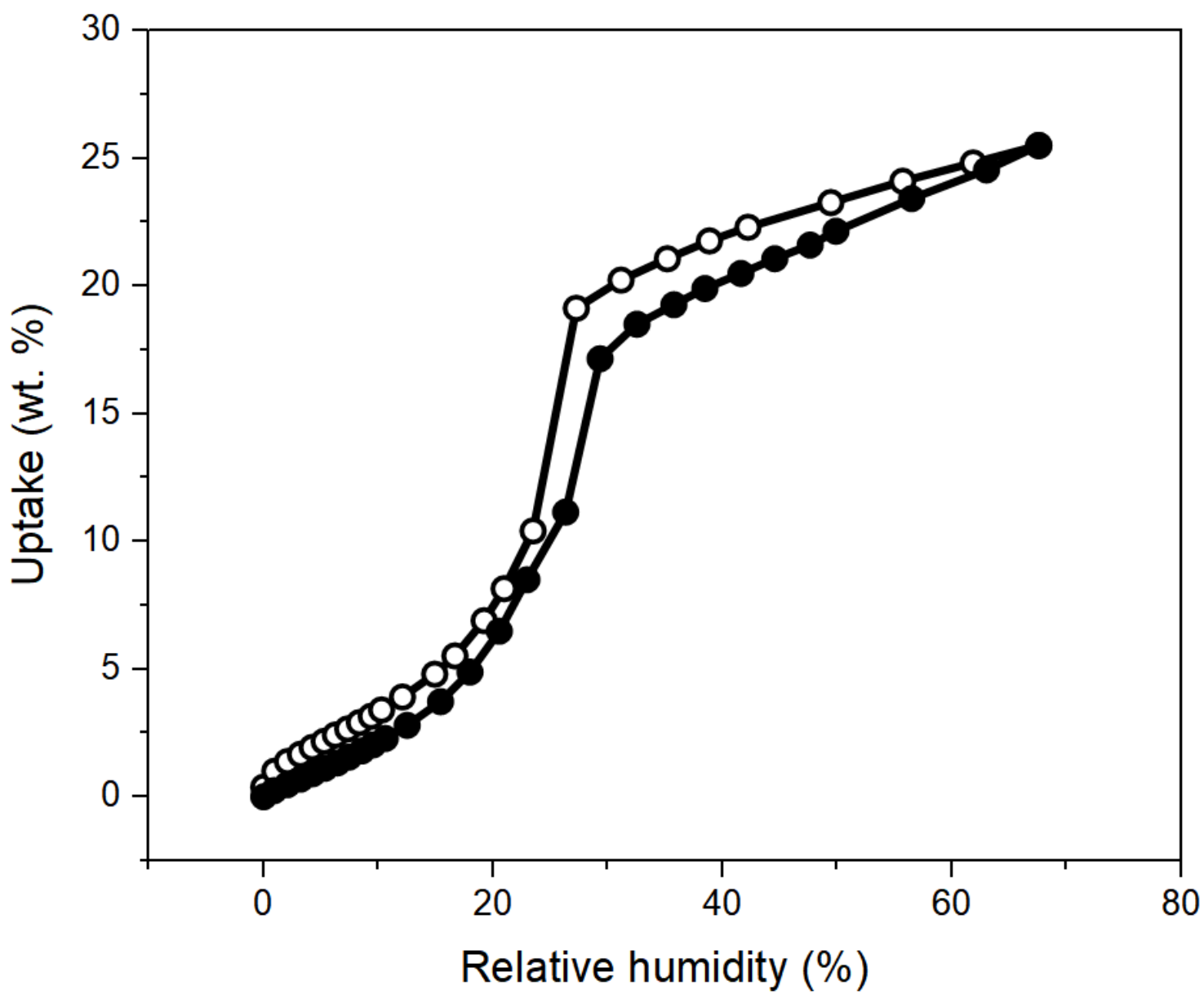

Figure S93 Water vapor adsorption (closed circles) and desorption (open circles) isotherms for **TIFSIX-25-Ni** measured at 60 °C.

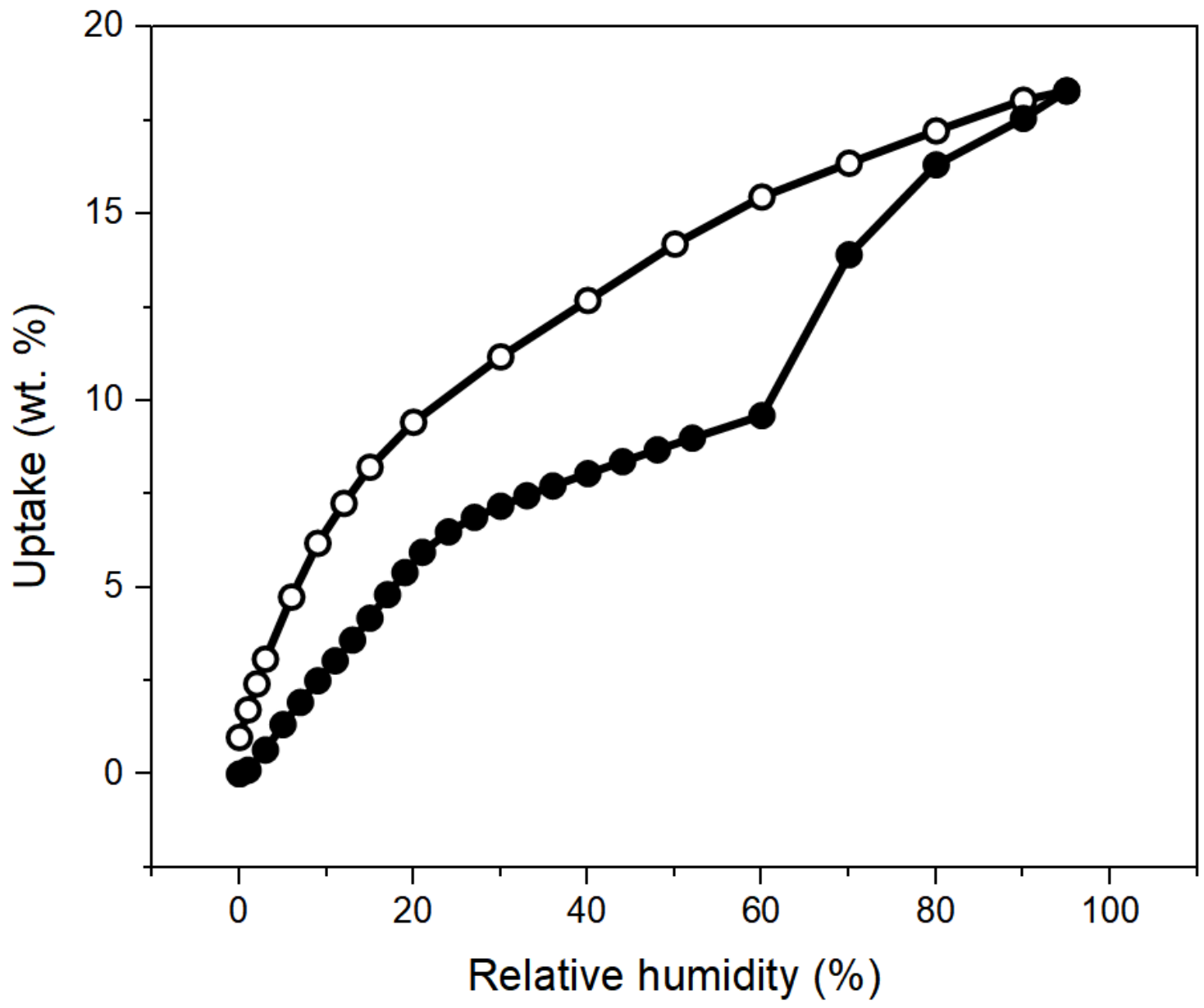

Figure S94 Water vapor adsorption (closed circles) and desorption (open circles) isotherms for **TIFSIX-25-Co** measured at 60 °C.

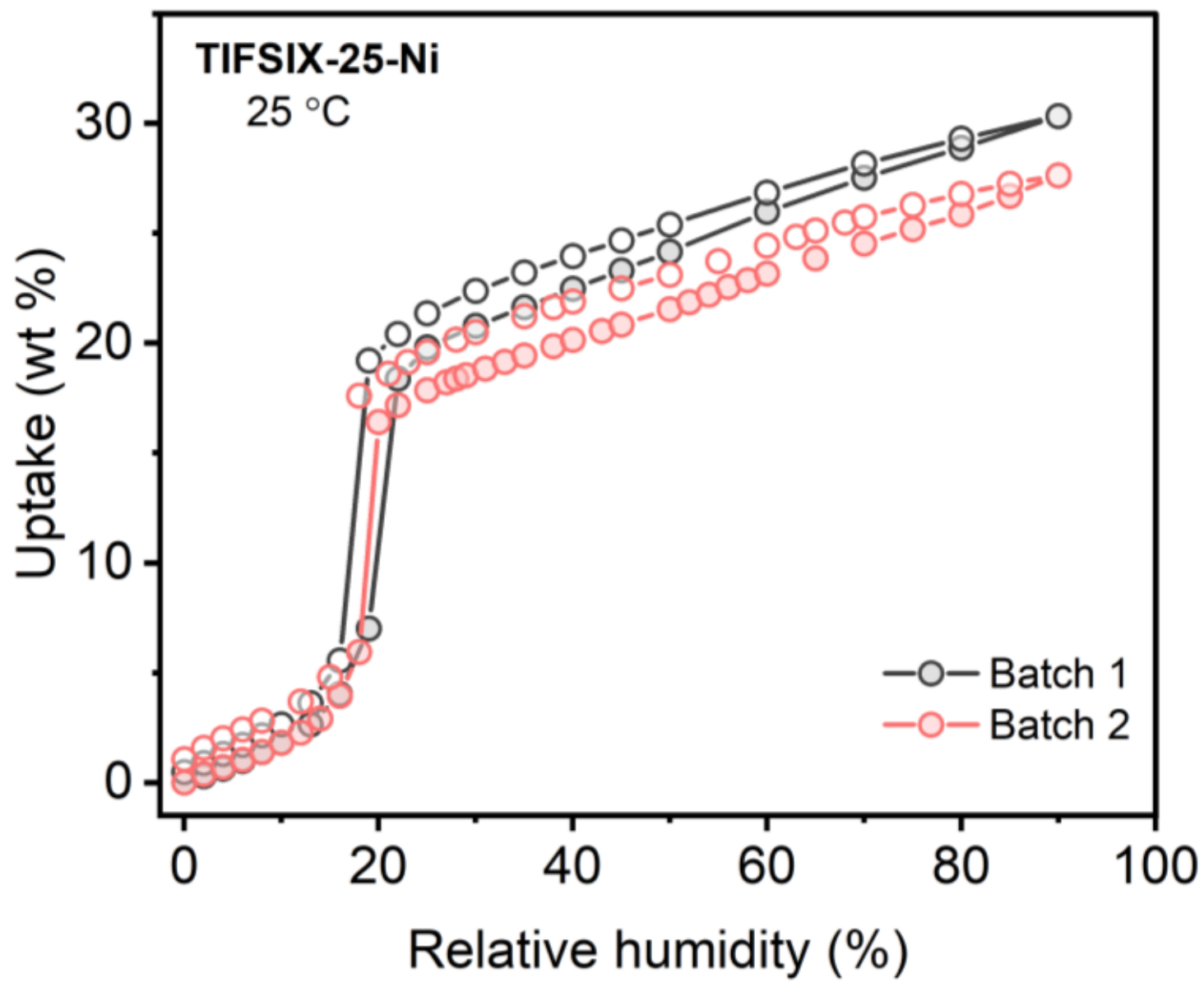

Figure S95 Water vapor adsorption (closed circles) and desorption (open circles) isotherms for two independently synthesized batches of **TIFSIX-25-Ni** collected at 25 °C.

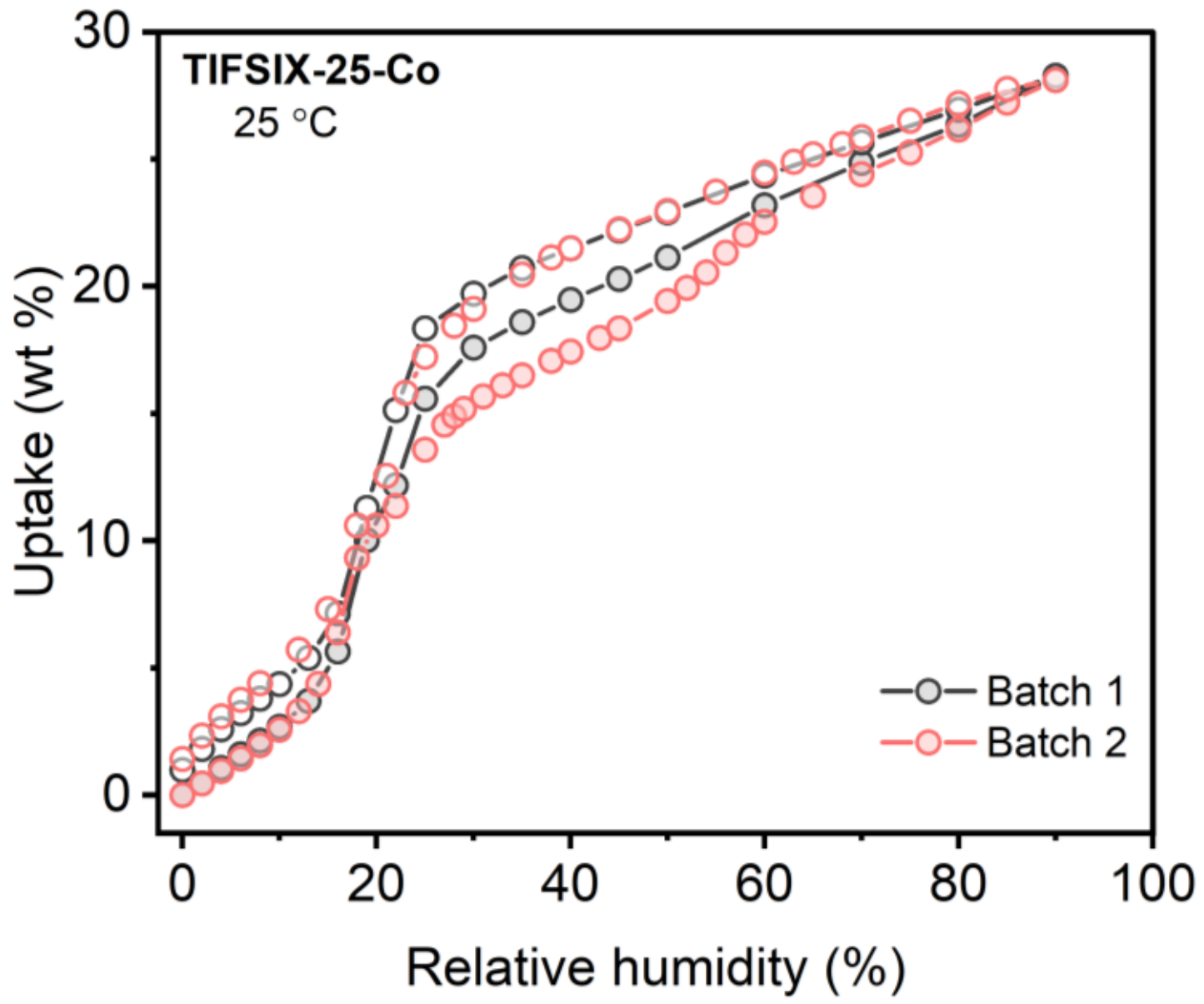

Figure S96 Water vapor adsorption (closed circles) and desorption (open circles) isotherms for two independently synthesized batches of **TIFSIX-25-Co** collected at 25 °C.

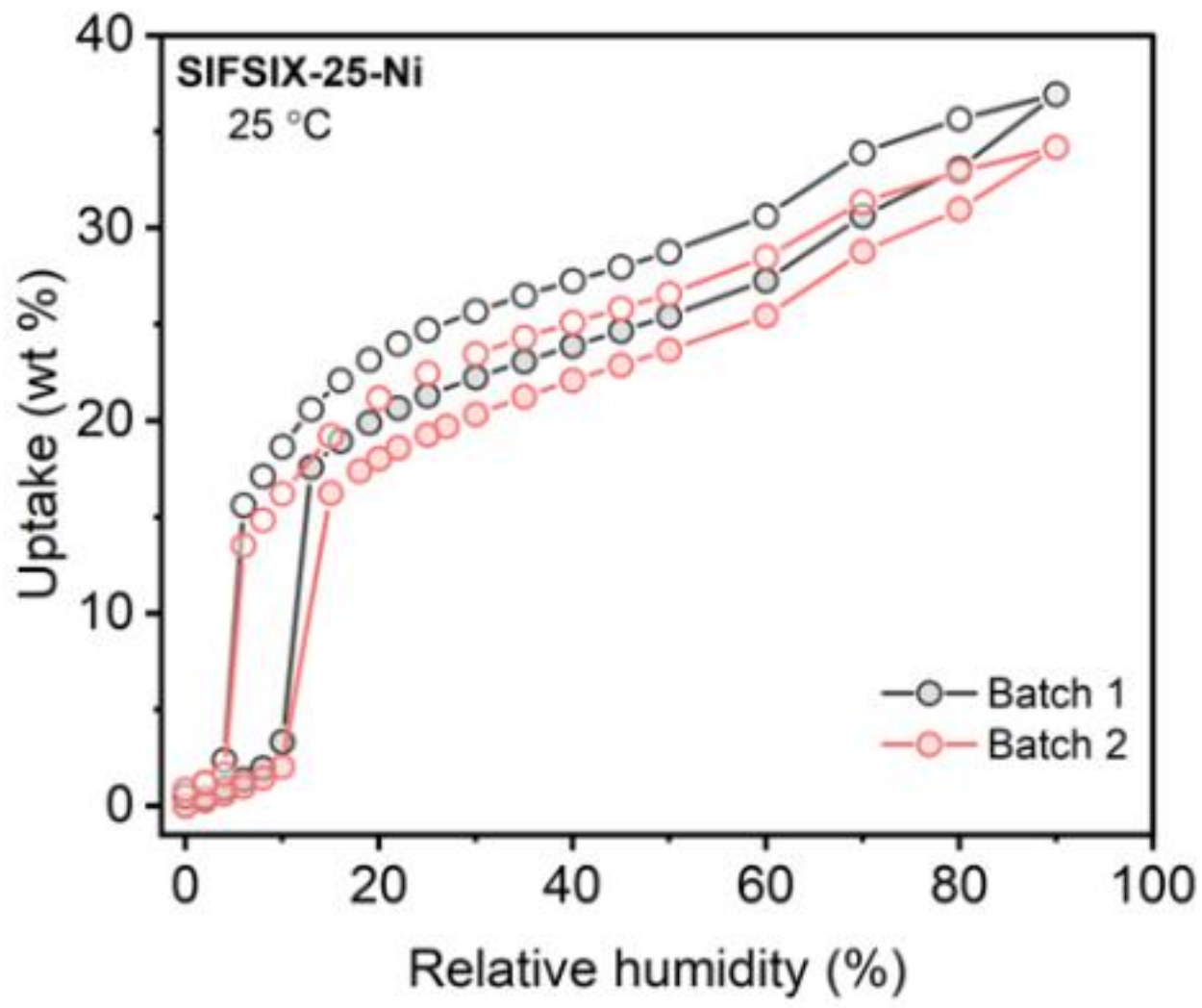

Figure S97 Water vapor adsorption (closed circles) and desorption (open circles) isotherms for two independently synthesized batches of **SIFSIX-25-Ni** collected at 25 °C.

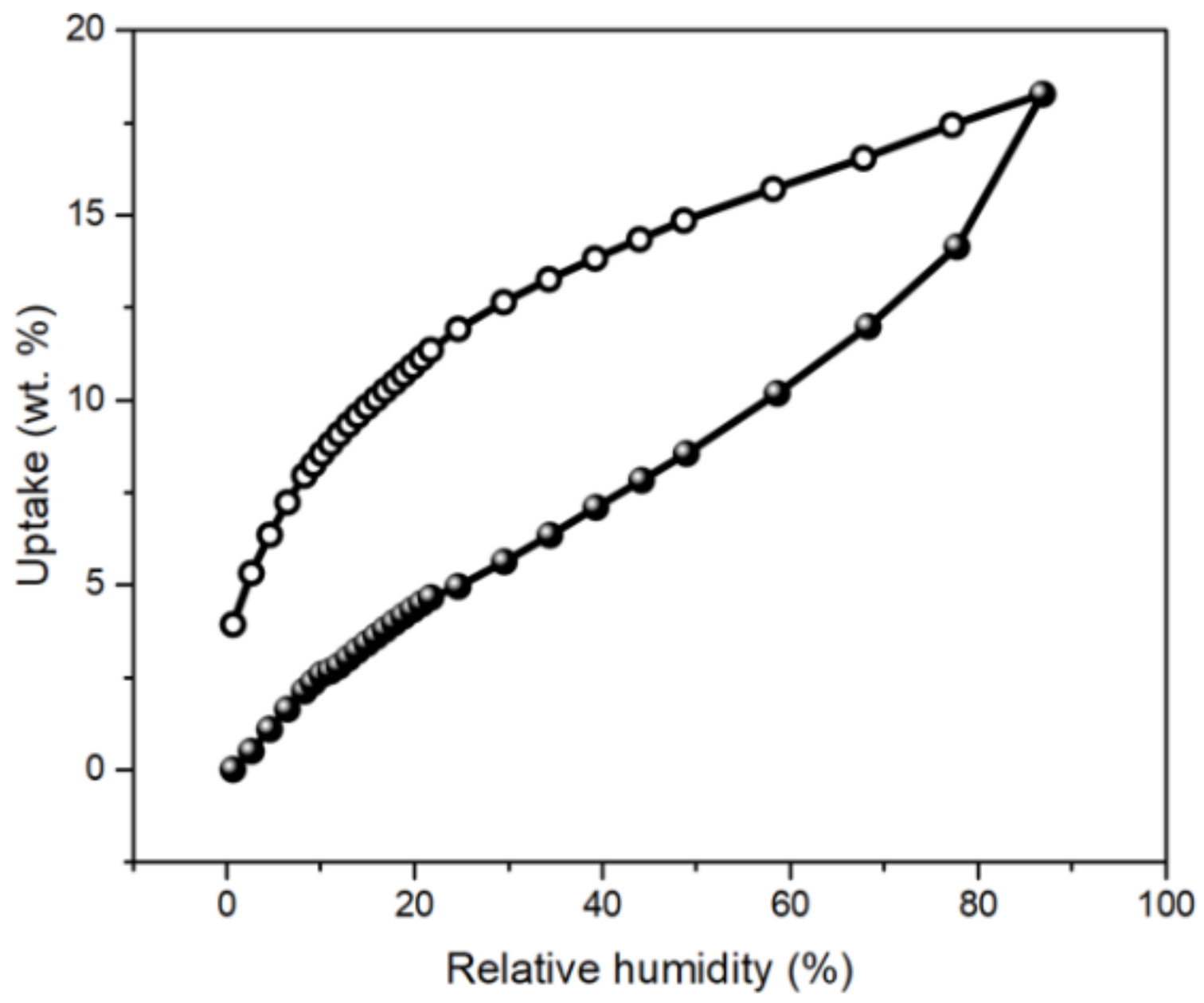

Figure S98 Water vapor adsorption (closed circles) and desorption (open circles) isotherms of **SIFSIX-25-Co** collected at 25 °C during a second repeated measurement.

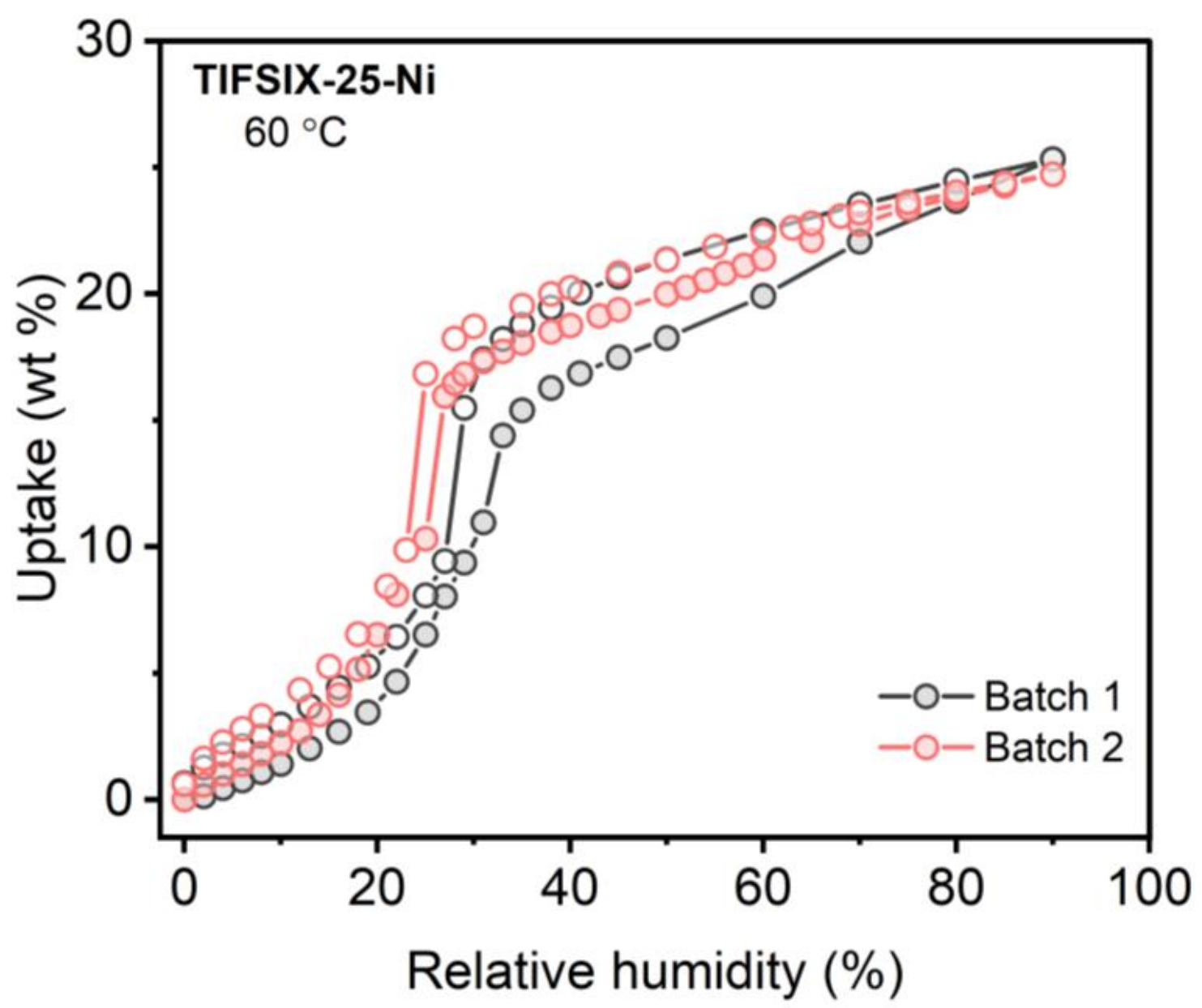

Figure S99 Water vapor adsorption (closed circles) and desorption (open circles) isotherms for two independently synthesized batches of **TIFSIX-25-Ni** collected at 60 °C.

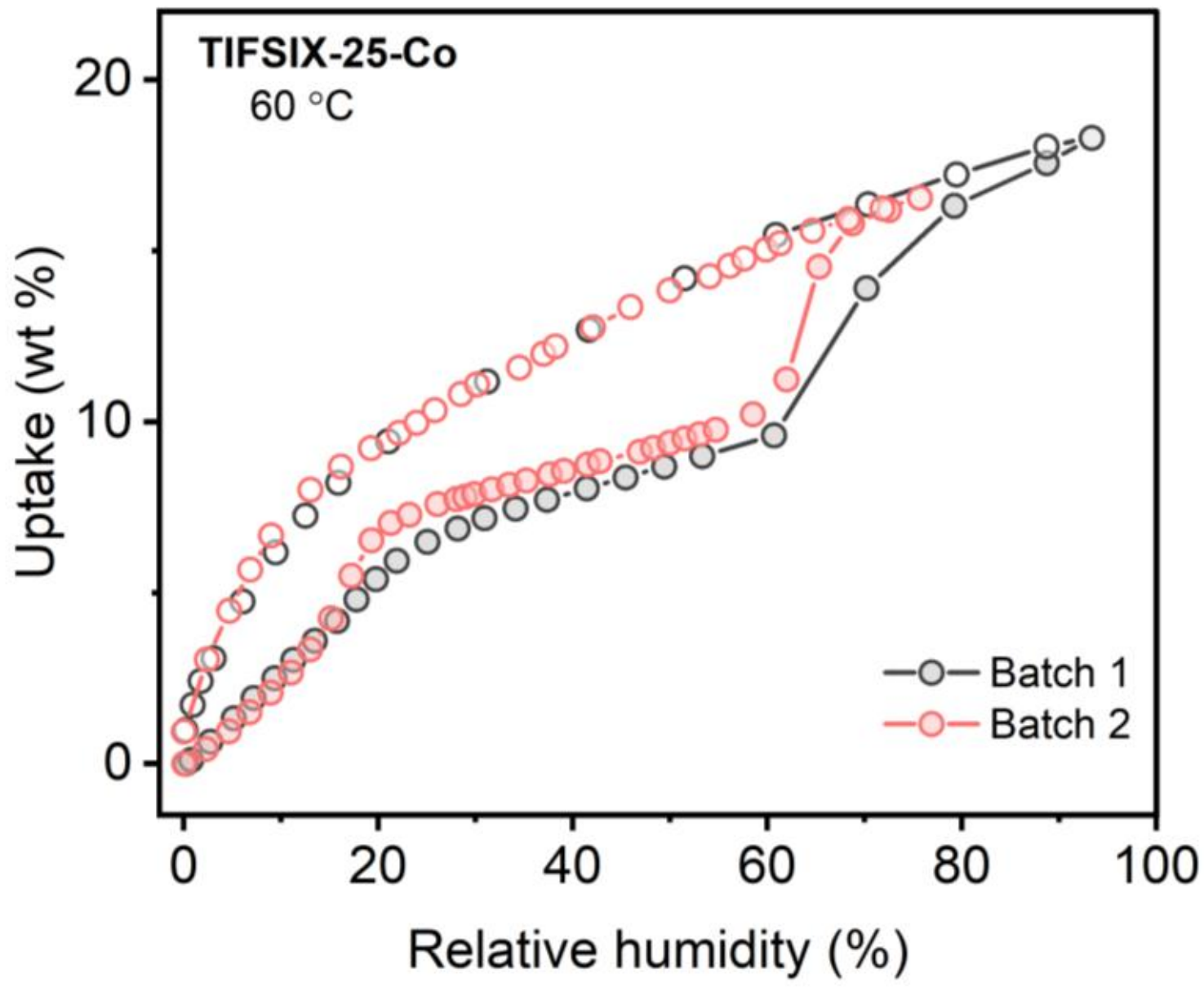

Figure S100 Water vapor adsorption (closed circles) and desorption (open circles) isotherms for two independently synthesized batches of **TIFSIX-25-Co** collected at 60 °C.

12.2 **M'FSIX-3-M**

In the discussion that follows, the inflection region of the sorption isotherms are described using three parameters: the onset RH of the inflection point (RH_i), the RH at the end of the inflection region (RH_f) and α, defined as the RH at the midpoint uptake between RH_i and RH_f.

12.2.1 Volumetric vacuum water sorption

12.2.1.1 SIFSIX-3-Ni

The sample was activated at 80 °C. The initial loading before the step was +2.98 H_2O/FU (14.9 wt.%), with a RH_i of 41.6%, an alpha of 42%, and after the step, a RH_f of 48% and an additional uptake of +1.02 H_2O/FU (5.1 wt.%). The structure of the loaded phase corresponds to that calculated for **sql-SIFSIX-3-Ni**.[24]

12.2.1.2 SIFSIX-3-Co

The sample was activated at 80 °C. The initial loading before the step was +3.19 H_2O/FU (15.9 wt.%), with a RH_i of 46.8%, an alpha of 63%, and after the step, a RH_f of 85% and an uptake of +1.04 H_2O/FU (5.19 wt.%). VH-PXRD showed the 2D **sql** peak intensifying starting from 50% RH. The structure of the loaded phase corresponds to that calculated for **sql-SIFSIX-3-Ni**.[24]

12.2.1.3 SIFSIX-3-Zn

The sample was activated at 60 °C. The initial loading before the step was +2.33 H_2O/FU (11.4 wt.%), with a RH_i of 18.3%, an alpha of 30%, and after the step, a RH_f of 55% and an uptake of +1.65 H_2O/FU (8.1 wt.%). The conversion range was noted between 20-65% RH, consistent with VHPXRD. The structure of the loaded phase corresponds to that calculated for **sql-SIFSIX-3-Ni**.[24]

12.2.1.4 TIFSIX-3-Ni

The sample was activated at 140 °C. The initial loading before the step was +3.0 H_2O/FU (14.56 wt.%), with a RH_i of 36%, an alpha of 45%, and after the step, a RH_f of 55% and a gradual uptake of +1.58 H_2O/FU (7.14 wt.%). Two inflection points were observed (40-60% and 60-80% RH), and VH-PXRD showed the **sql** phase intensifying

from 60% RH. The structure of the loaded phase corresponds to that calculated for **sql-SIFSIX-3-Ni**.[24]

12.2.1.5 TIFSIX-3-Co

The sample was activated at 100 °C. The initial loading before the step was +2.09 H_2O/FU (9.89 wt.%), with a RH_i of 45.0%, an alpha of 75%, and after the step, a RH_f of 90% and a gradual uptake of +1.24 H_2O/FU (5.87 wt.%). The framework stays in the **pcu** phase over the entire RH range.

12.2.1.6 GEFSIX-3-Ni

The sample was activated at 80 °C. The initial loading before the step was +3.04 H_2O/FU (13.50 wt.%), with a RH_i of 37%, an alpha of 40%, and after the step, a RH_f of 50% and a gradual loading of +1.28 H_2O/FU (5.73 wt.%). The structure of the loaded phase corresponds to that calculated for **sql-SIFSIX-3-Ni**.[24]

12.2.1.7 GEFSIX-3-Co

The sample was activated at 140 °C. The initial loading before the step was +2.82 H_2O/FU (12.56 wt.%), with a RH_i of 37.0%, an alpha of 42%, and after the step, a RH_f of 67% and a very gradual uptake of +2.46 H_2O/FU (10.92 wt.%), while desorption data showed +3.80 H_2O/FU (16.90 wt.%) remaining. The structure of the loaded phase corresponds to that calculated for **sql-SIFSIX-3-Ni**.[24]

12.2.2 Gravimetric vacuum water sorption

12.2.2.1 SIFSIX-3-Ni

The sample was activated at 60 °C. The initial loading before the step was +2.98 H_2O/FU (14.9 wt.%), with a RH_i of 44.9%, and after the step, the loading was -2 H_2O/FU (10 wt.%). PXRD pattern after the experiment showed a mixture of **sql** and other phases reported by Elsaidi *et al.*[25]

12.2.2.2 SIFSIX-3-Co

The sample was activated at 40 °C. The initial loading before the step was +2.61 H_2O/FU (13.05 wt.%), with a RH_i of 40%, and after the step, the loading was -4.62

H_2O/FU (-26.16 wt.%). PXRD pattern after the experiment did not match any known phases.

12.2.2.3 SIFSIX-3-Zn

The sample was activated at 40 °C. The initial loading before the step was +1.44 H_2O/FU (7.10 wt.%), with a RH_i of 7.10%, and after the step, the loading was -4.46 H_2O/FU (-21.93 wt.%). PXRD pattern after the experiment corresponds to that of the inorganic starting material.

12.2.2.4 TIFSIX-3-Ni

The sample was activated at 100 °C. The initial loading before the step was +3.60 H_2O/FU (17.02 wt.%), and after the step, the loading was -3.24 H_2O/FU (-15.3 wt.%) with a desorption value of -1.39 H_2O/FU (-6.60 wt.%). PXRD pattern after the experiment did not match any known phases.

12.2.2.5 TIFSIX-3-Co

The sample was activated at 80 °C. The initial loading before the step was +2.69 H_2O/FU (12.73 wt.%), with a RH_i of 25.00%, and after the step, the loading was -3.14 H_2O/FU (-14.89 wt.%) with a desorption value of -2.96 H_2O/FU (-14.01 wt.%). PXRD pattern after the experiment corresponds to that of the inorganic starting material.

12.2.2.6 GEFSIX-3-Ni

The sample was activated at 60 °C. The initial loading before the step was +3.19 H_2O/FU (14.21 wt.%), with a RH_i of 64.38%, and after the step, the loading was -2.41 H_2O/FU (-10.71 wt.%), with a negative desorption trace. PXRD pattern after the experiment did not match any known phases.

12.2.2.7 GEFSIX-3-Co

The sample was activated at 60 °C. The initial loading before the step was +2.49 H_2O/FU (11.09 wt.%), with a RH_i of 35.01%, and after the step, the loading was -4.43 H_2O/FU (19.72 wt.%), with a negative desorption trace. PXRD pattern after the experiment did not match any known phases.

12.2.3 Gravimetric ambient-pressure water sorption

12.2.3.1 SIFSIX-3-Ni

The sample was activated at 80 °C. The initial loading before the inflection was +2.54 H_2O/FU (12.7 wt.%), with a RH_i expected at 85%, and after the step, the loading was +0.78 H_2O/FU (16.6 wt.%), with desorption showing 10 wt.% remaining. PXRD pattern after the experiment showed a mixture of **sql** and other phases reported by Elsaidi *et al.*[25]

12.2.3.2 SIFSIX-3-Co

The sample was activated at 80 °C. The initial loading before the inflection was +2.61 H_2O/FU (13.0 wt.%), with a RH_i of 77.4%, and after the step, the loading was -1.25 H_2O/FU (-6.2 wt.%), with desorption showing 5.2 wt.% remaining. PXRD pattern after the experiment did not match any known phases.

12.2.3.3 SIFSIX-3-Zn

The sample was activated at 80 °C. The initial loading before the inflection was +1.90 H_2O/FU (9.33 wt.%), with a RH_i of 19.86%, and after the step, the loading was -3.12 H_2O/FU (-15.31 wt.%). PXRD pattern after the experiment corresponds to that of the inorganic starting material.

12.2.3.4 TIFSIX-3-Ni

The sample was activated at 100 °C. The initial loading before the small inflection was +2.12 H_2O/FU (10.07 wt.%), with a RH_i of 53.63%, and after the step, the loading was -0.11 H_2O/FU (-0.52 wt.%) with desorption showing +1.34 H_2O/FU (6.35 wt.%) remaining. PXRD pattern after the experiment showed a mixture of **sql** and other phases reported by Elsaidi *et al.*[25]

12.2.3.5 TIFSIX-3-Co

The sample was activated at 100 °C. The initial loading before the inflection was +2.12 H_2O/FU (10.04 wt.%), with a RH_i of 15.01%, and after the step, the loading was -3.84 H_2O/FU (-18.19 wt.%) with an amorphous phase after desorption. PXRD pattern after the experiment indicates that sample loses crystallinity.

12.2.3.6 GEFSIX-3-Ni

The sample was activated at 80 °C. The initial loading before the small inflection was +1.66 H_2O/FU (7.37 wt.%), with a RH_i of 34.48%, and after the step, the total uptake was +2.70 H_2O/FU (12.03 wt.%) with desorption showing +1.47 H_2O/FU (6.54 wt.%) remaining. PXRD pattern after the experiment showed a mixture of **sql**, **pcu** and other phases reported by Elsaidi *et al.*[25]

12.2.3.7 GEFSIX-3-Co

The sample was activated at 80 °C. The initial loading before the inflection was +2.49 H_2O/FU (11.07 wt.%), with a RH_i of 34.48%, and after the step, the total uptake reached 3.65 H_2O/FU, with desorption showing +2.37 H_2O/FU (10.53 wt.%) remaining. PXRD pattern after the experiment showed a mixture of **sql** and other phases reported by Elsaidi *et al.*[25]

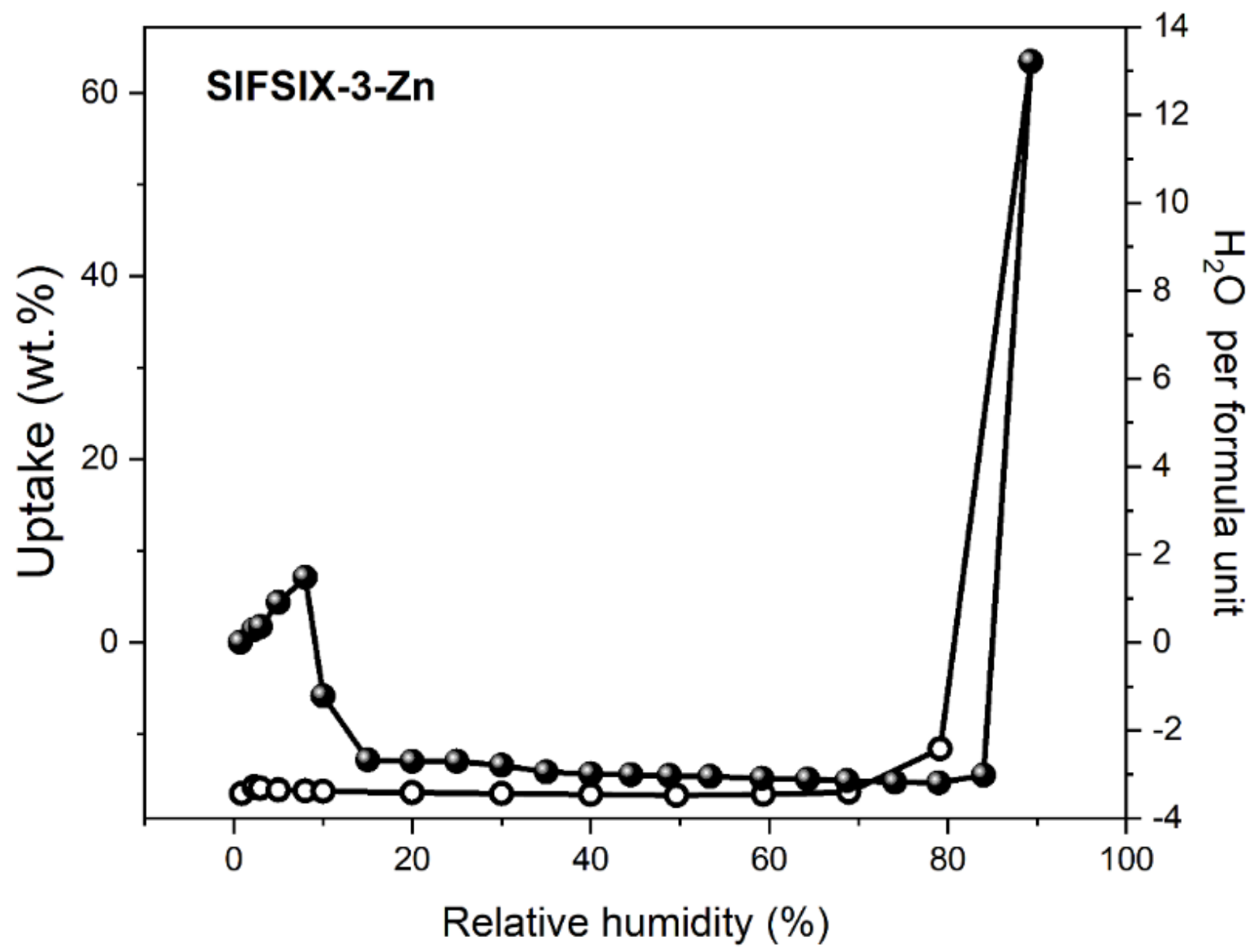

Figure S101 Water vapor adsorption (closed circles) and desorption (open circles) isotherm for **SIFSIX-3-Zn** measured at 25 °C using a DVS Vacuum system.

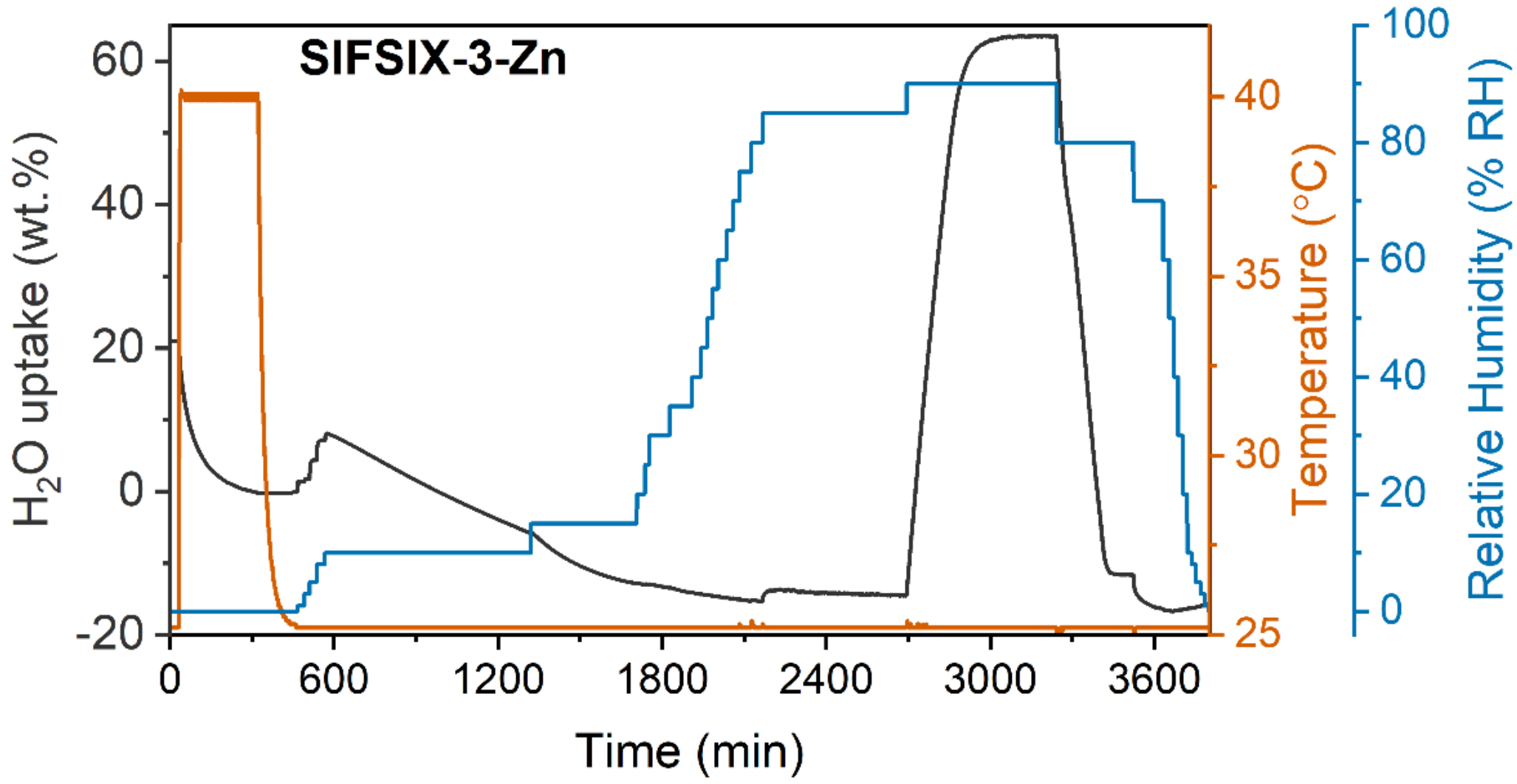

Figure S102 Dynamic water vapor sorption profile of **SIFSIX-3-Zn** displaying the mass change of **SIFSIX-3-Zn** with respect to time and relative humidity measured using a DVS Vacuum system.

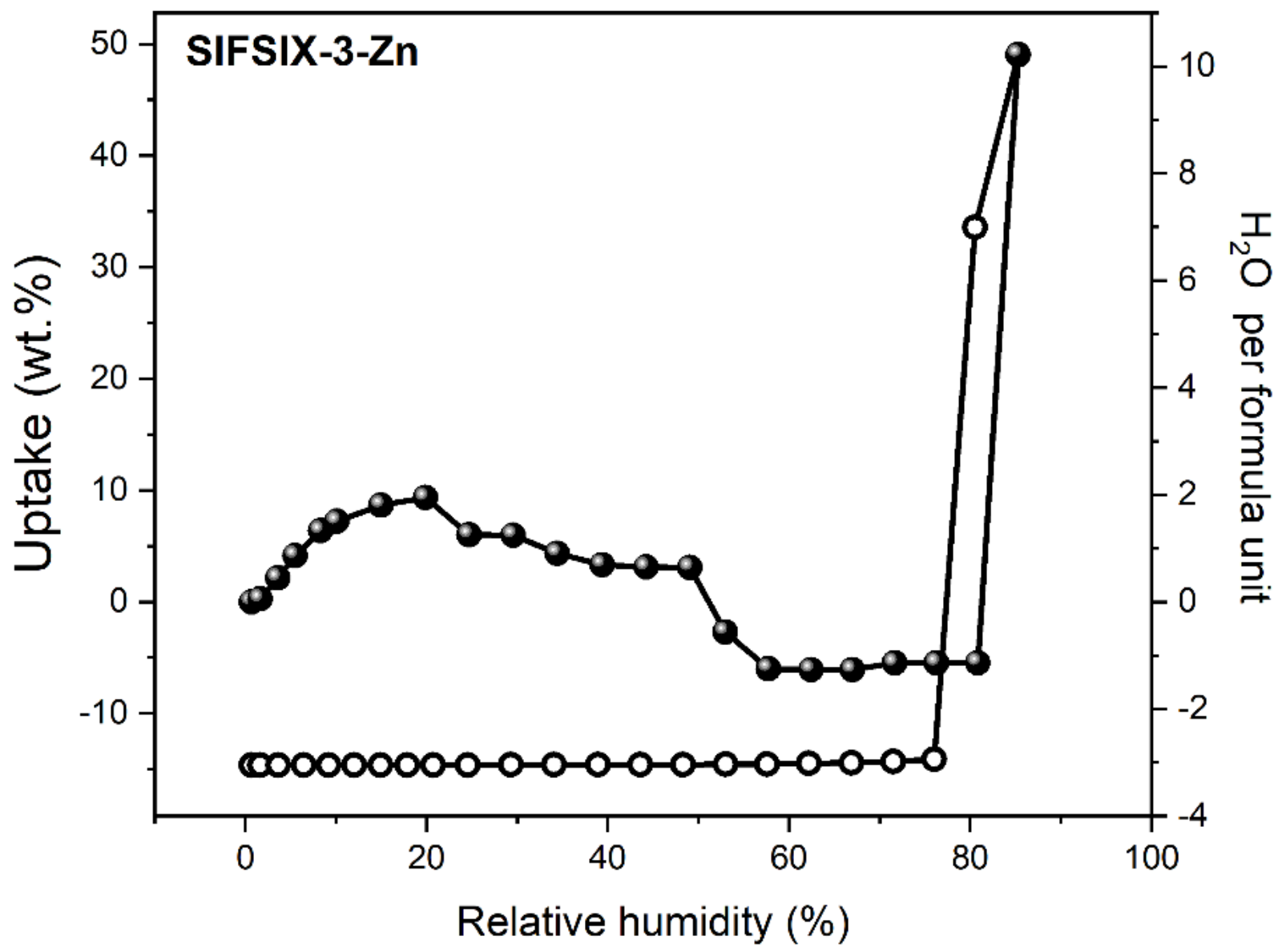

Figure S103 Water vapor adsorption (closed circles) and desorption (open circles) isotherm for **SIFSIX-3-Zn** measured at 25 °C using a DVS Adventure system.

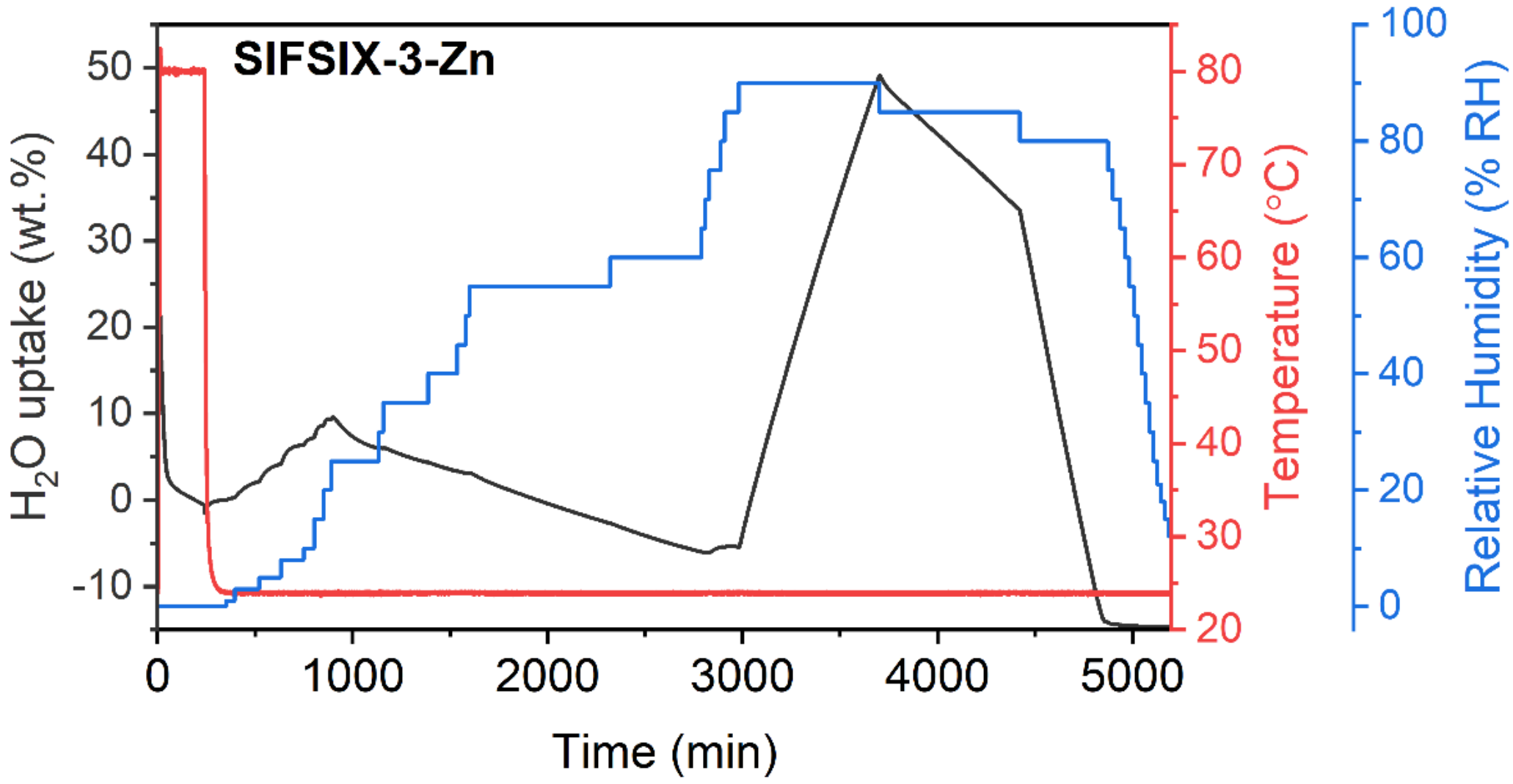

Figure S104 Dynamic water vapor sorption profile of **SIFSIX-3-Zn** displaying the mass change of **SIFSIX-3-Zn** with respect to time and relative humidity measured using a DVS Adventure system.

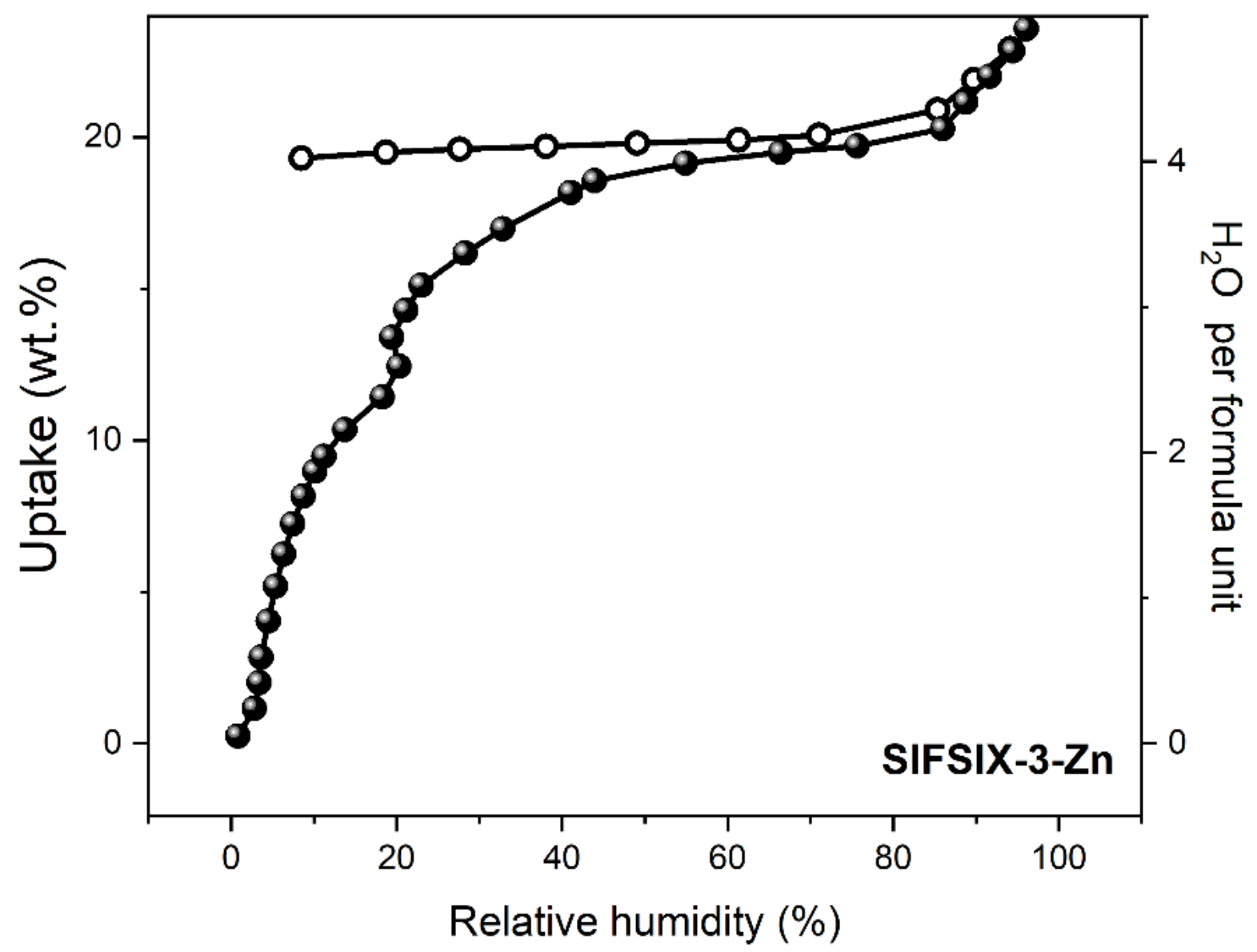

Figure S105 Water vapor adsorption (closed circles) and desorption (open circles) isotherm for **SIFSIX-3-Zn** measured at 25 °C using BELSORP Max volumetric system.

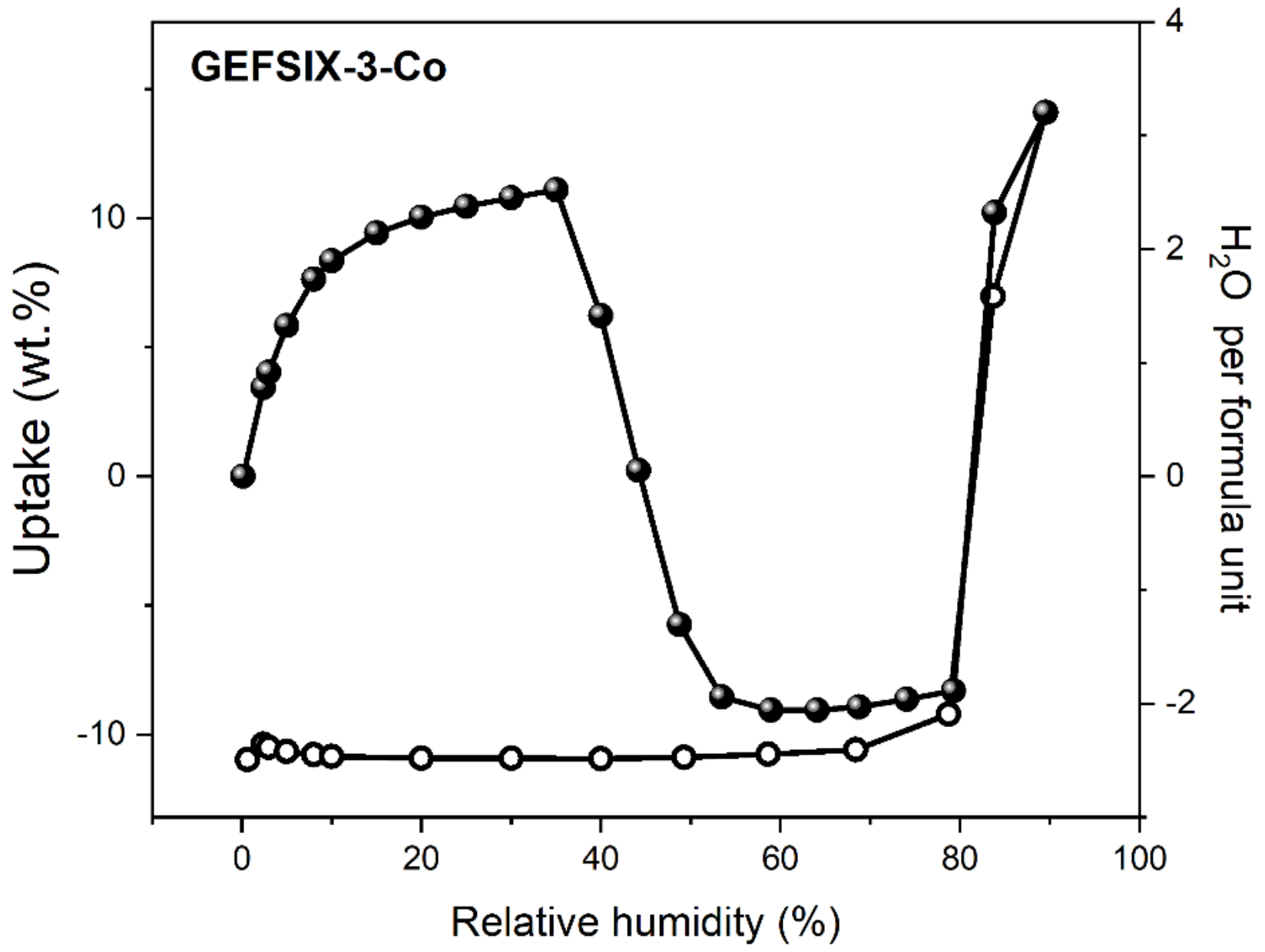

Figure S106 Water vapor adsorption (closed circles) and desorption (open circles) isotherm for **GEFSIX-3-Co** measured at 25 °C using a DVS Vacuum system.

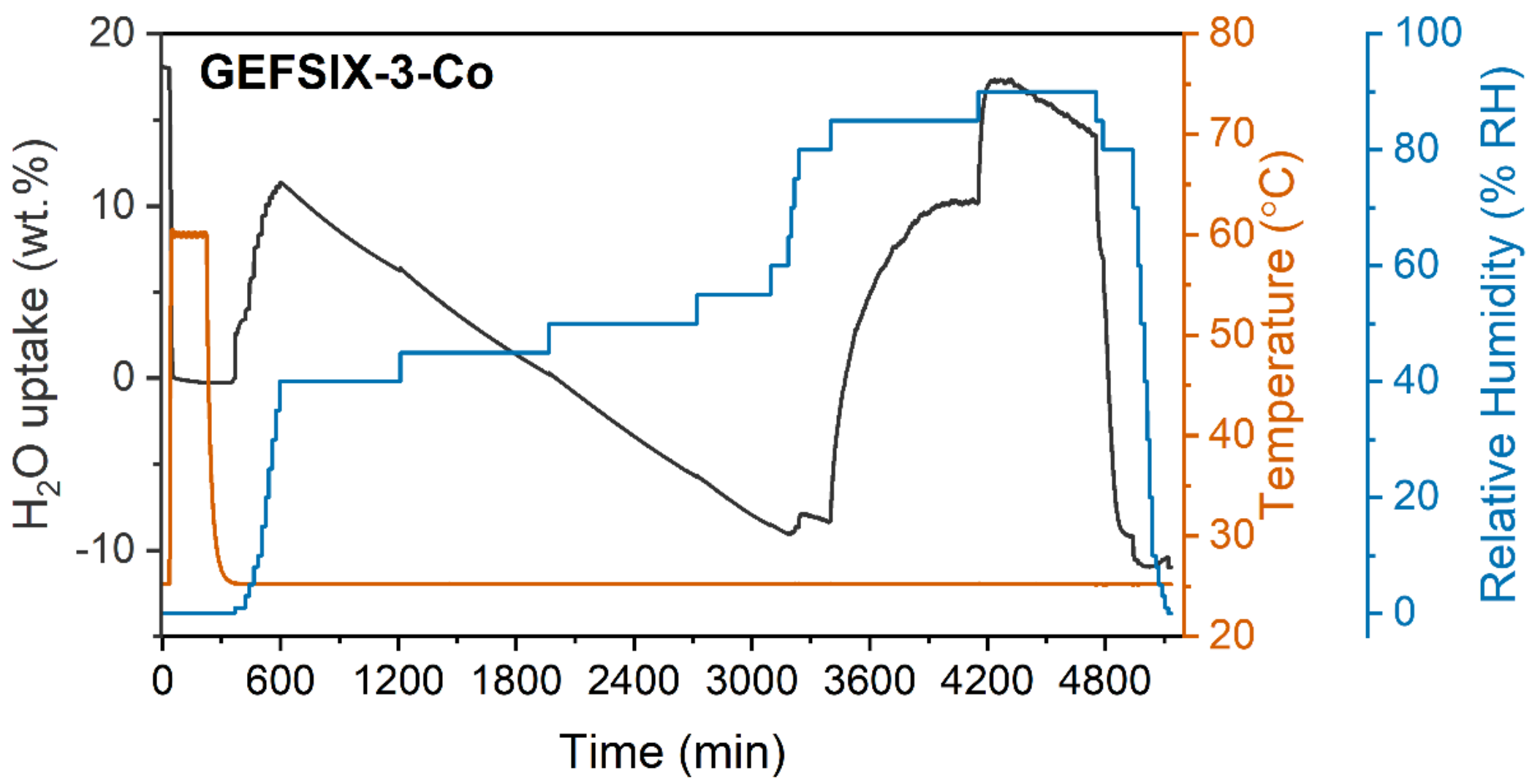

Figure S107 Dynamic water vapor sorption profile of **GEFSIX-3-Co** displaying the mass change of **GEFSIX-3-Co** with respect to time and relative humidity measured using a DVS Vacuum system.

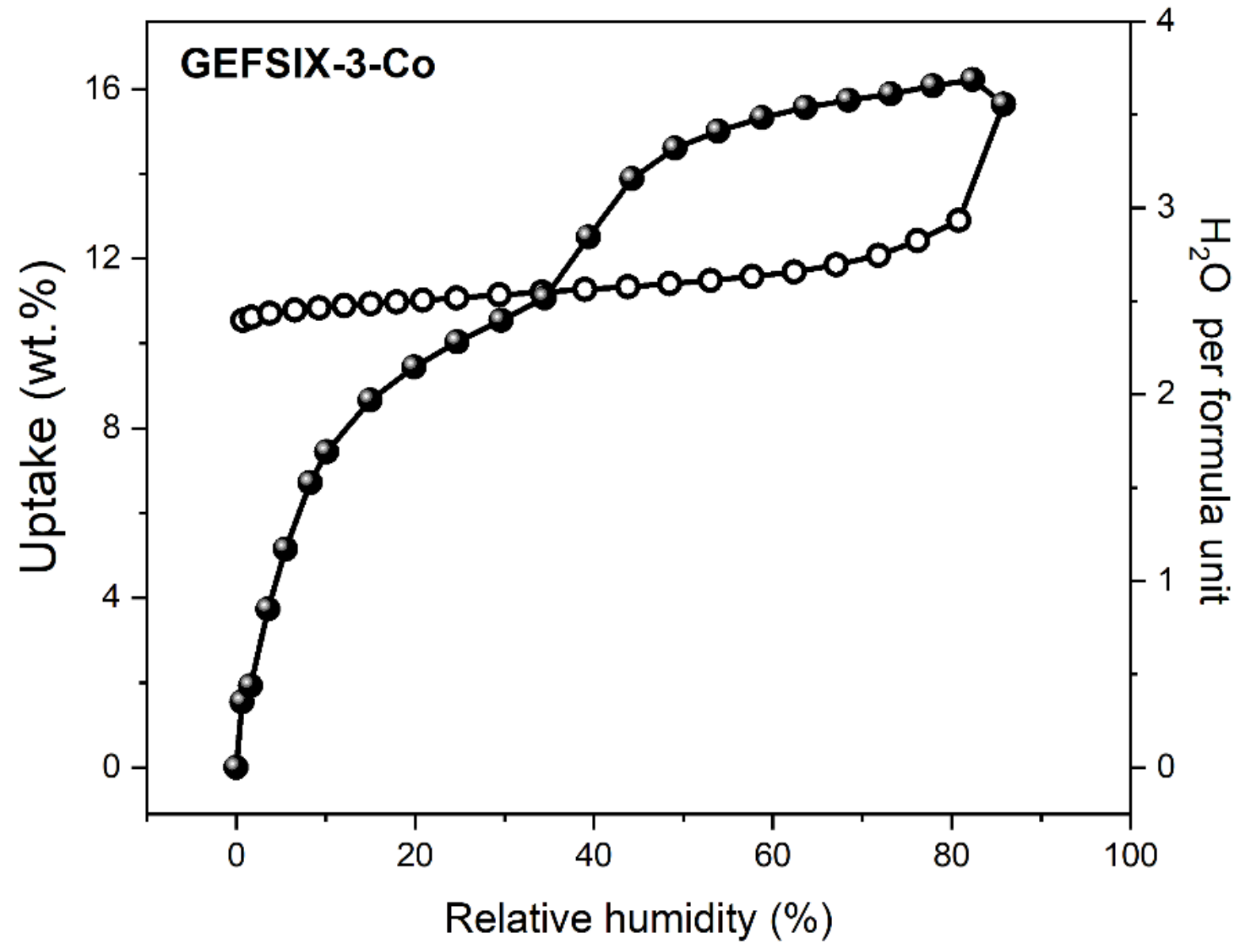

Figure S108 Water vapor adsorption (closed circles) and desorption (open circles) isotherm for **GEFSIX-3-Co** measured at 25 °C using a DVS Adventure system.

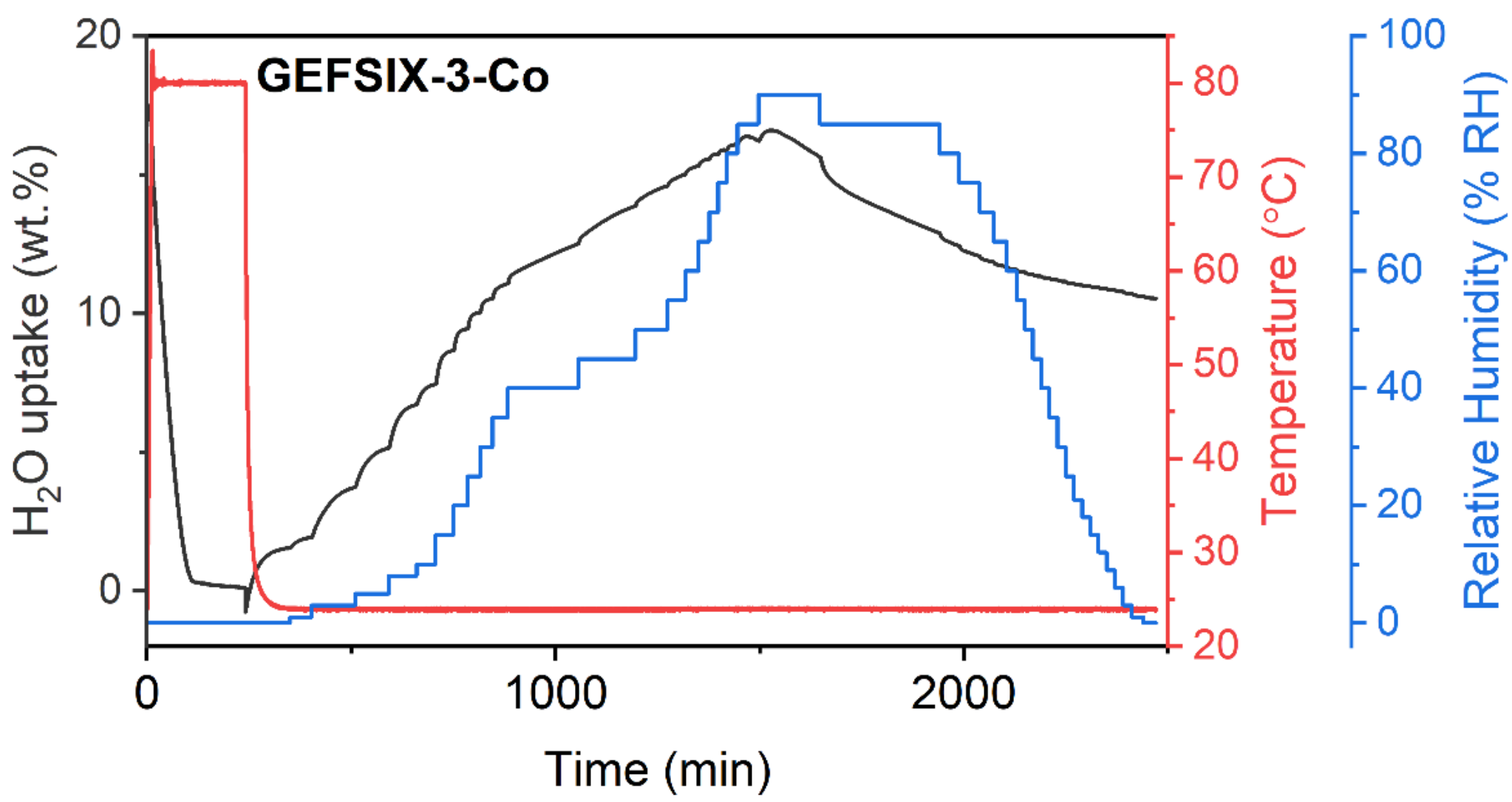

Figure S109 Dynamic water vapor sorption profile of **GEFSIX-3-Co** displaying the mass change of **GEFSIX-3-Co** with respect to time and relative humidity measured using a DVS Adventure system.

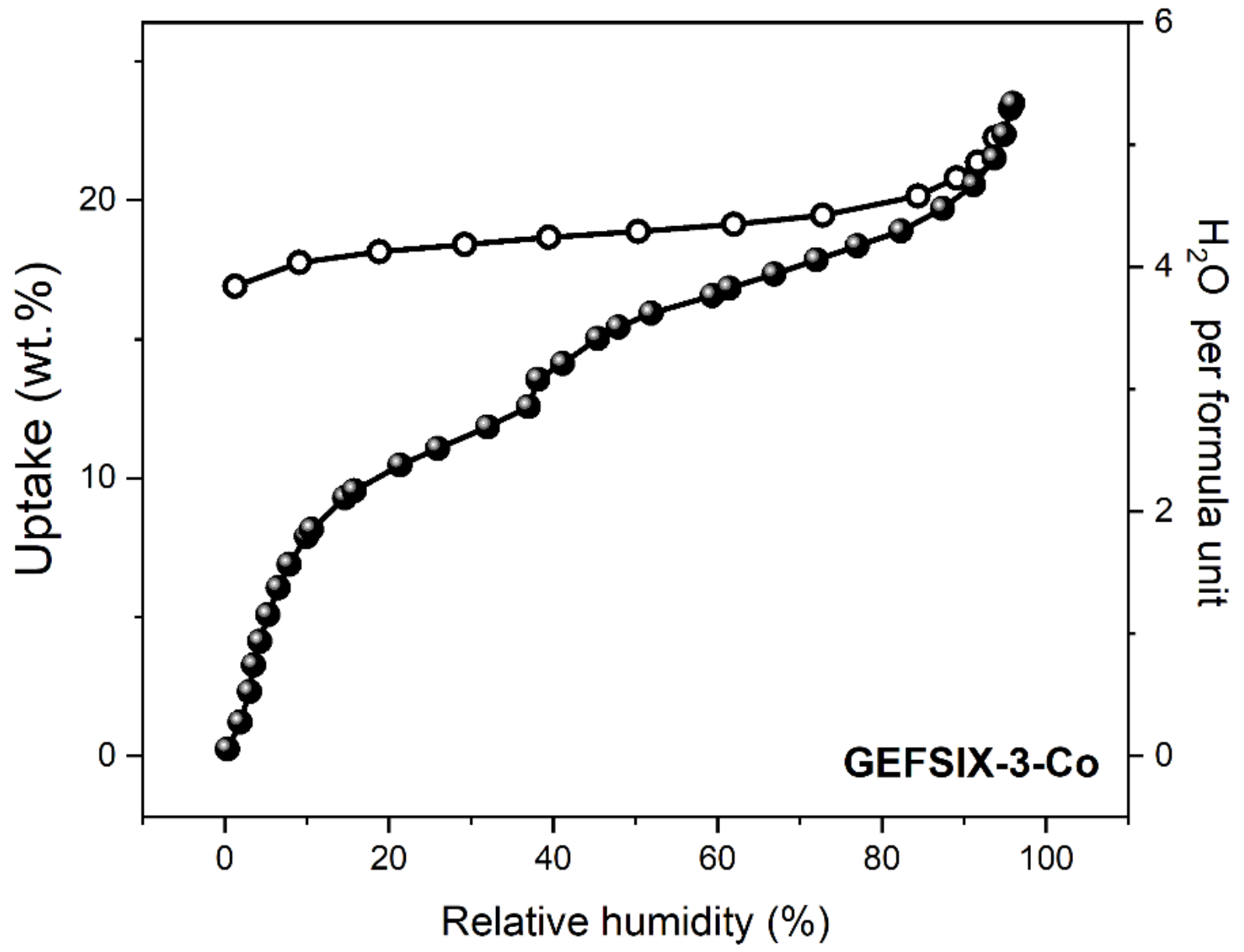

Figure S110 Water vapor adsorption (closed circles) and desorption (open circles) isotherm for **GEFSIX-3-Co** measured at 25 °C using BELSORP Max volumetric system.

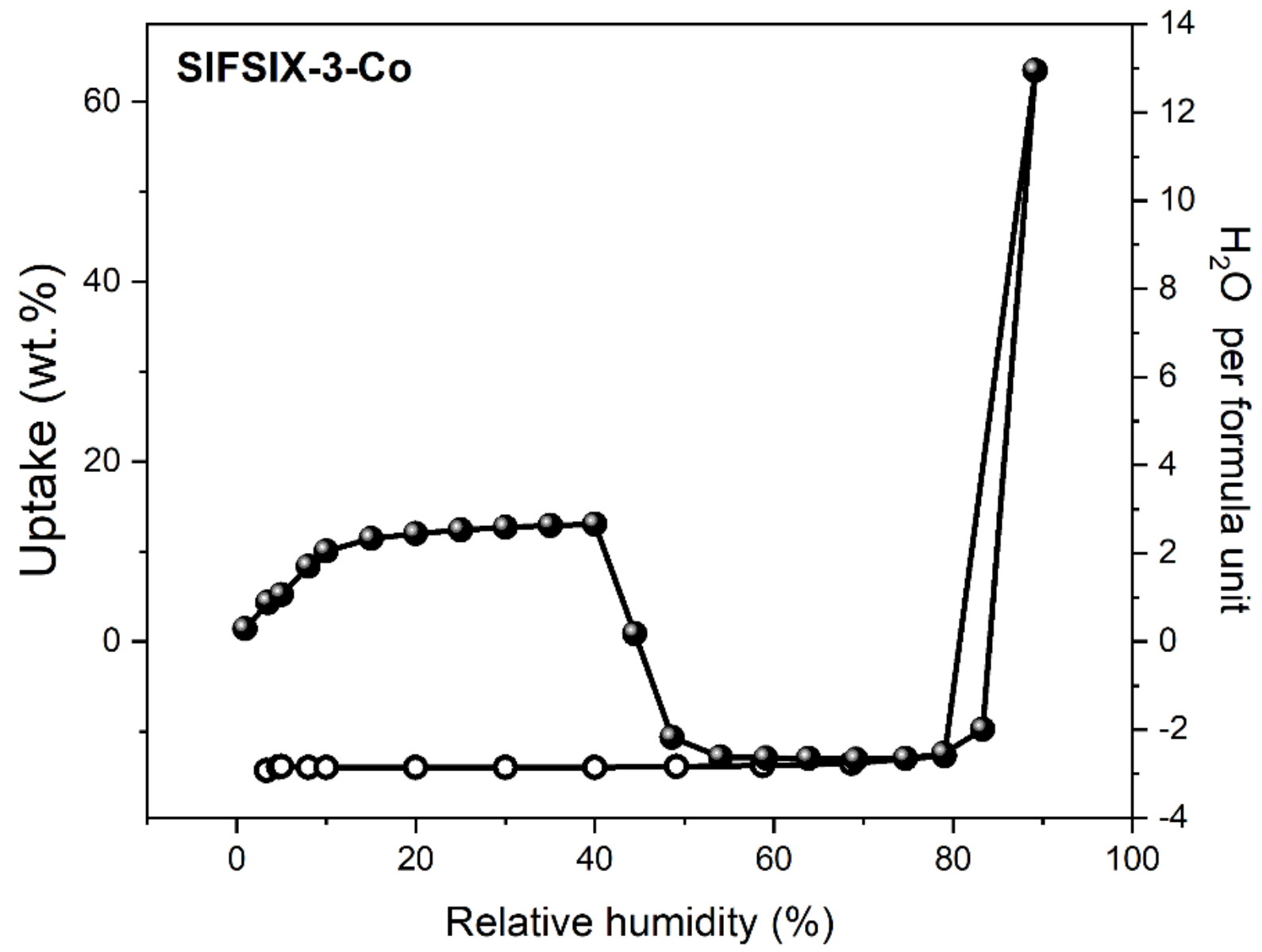

Figure S111 Water vapor adsorption (closed circles) and desorption (open circles) isotherm for **SIFSIX-3-Co** measured at 25 °C using a DVS Vacuum system.

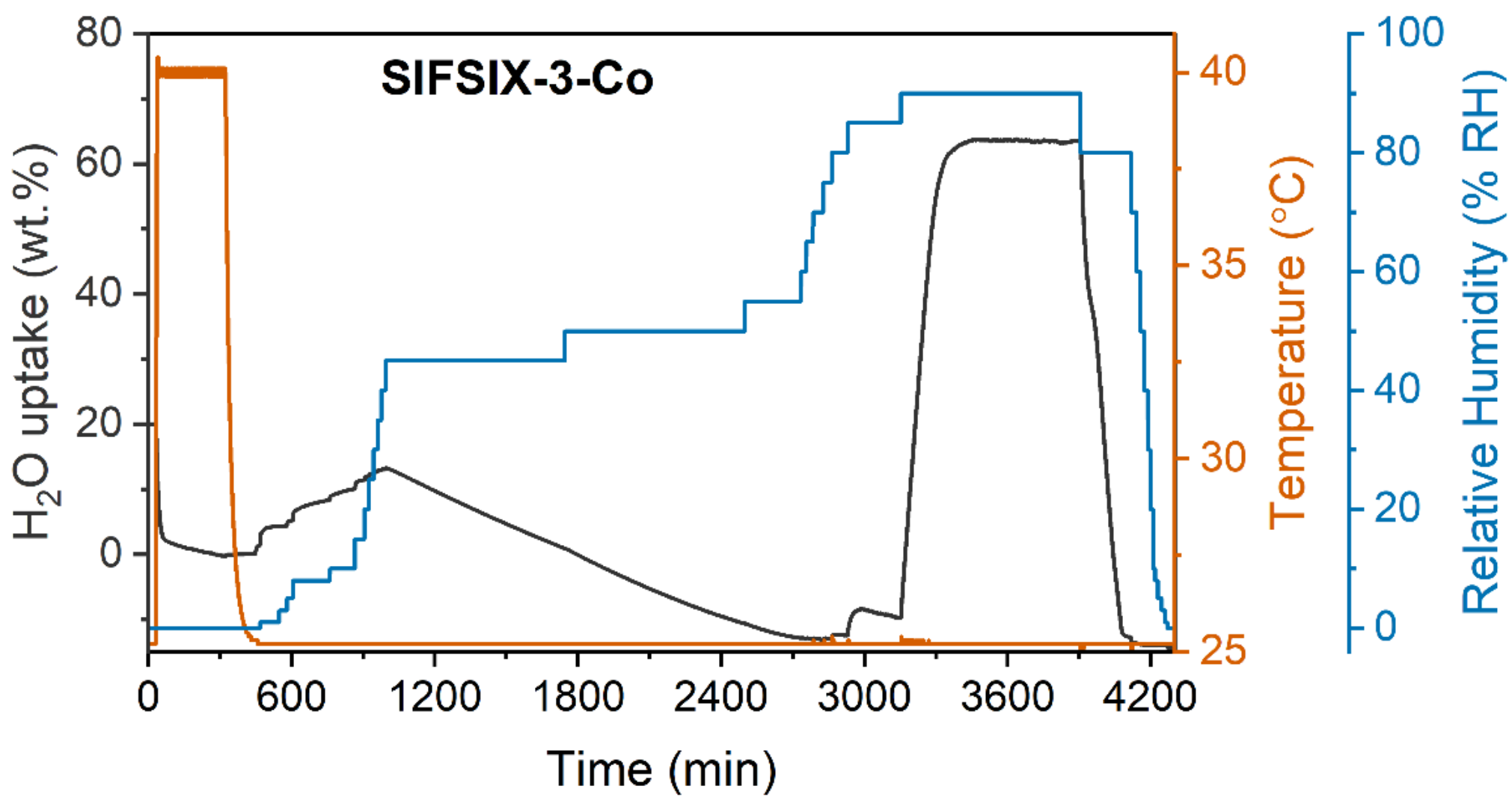

Figure S112 Dynamic water vapor sorption profile of **SIFSIX-3-Co** displaying the mass change of **SIFSIX-3-Co** with respect to time and relative humidity measured using a DVS Vacuum system.

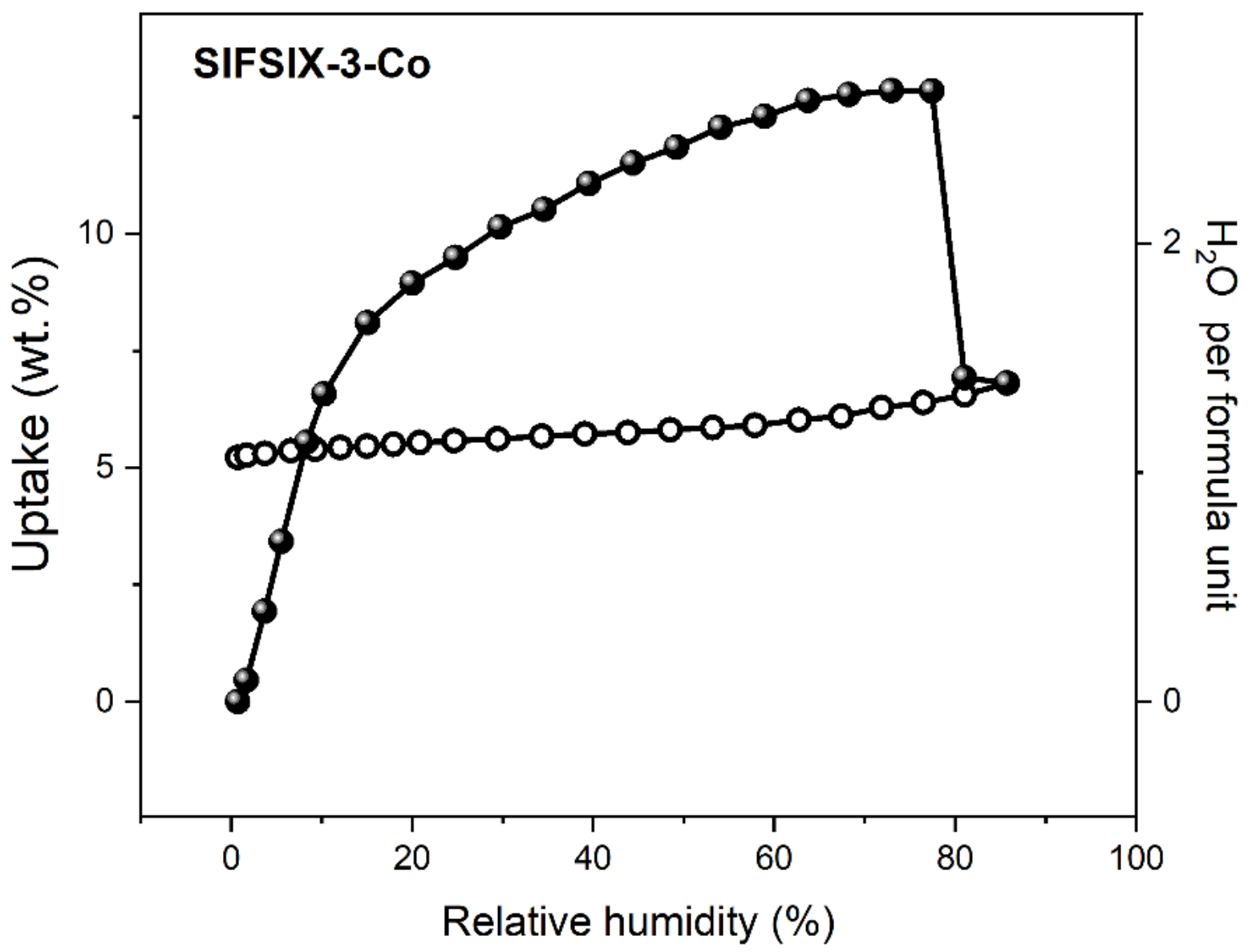

Figure S113 Water vapor adsorption (closed circles) and desorption (open circles) isotherm for **SIFSIX-3-Co** measured at 25 °C using a DVS Adventure system.

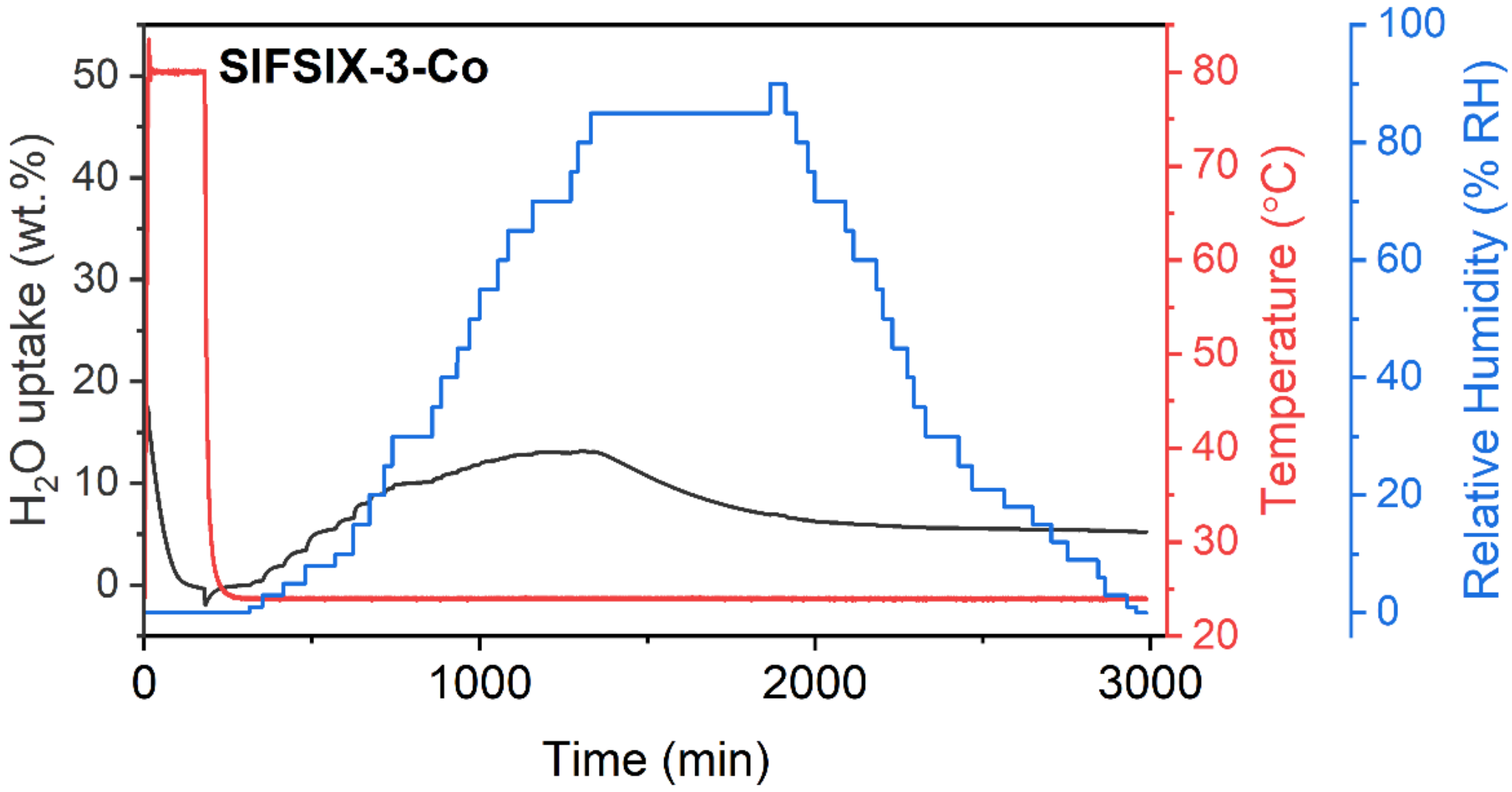

Figure S114 Dynamic water vapor sorption profile of **SIFSIX-3-Co** displaying the mass change of **SIFSIX-3-Co** with respect to time and relative humidity measured using a DVS Adventure system.

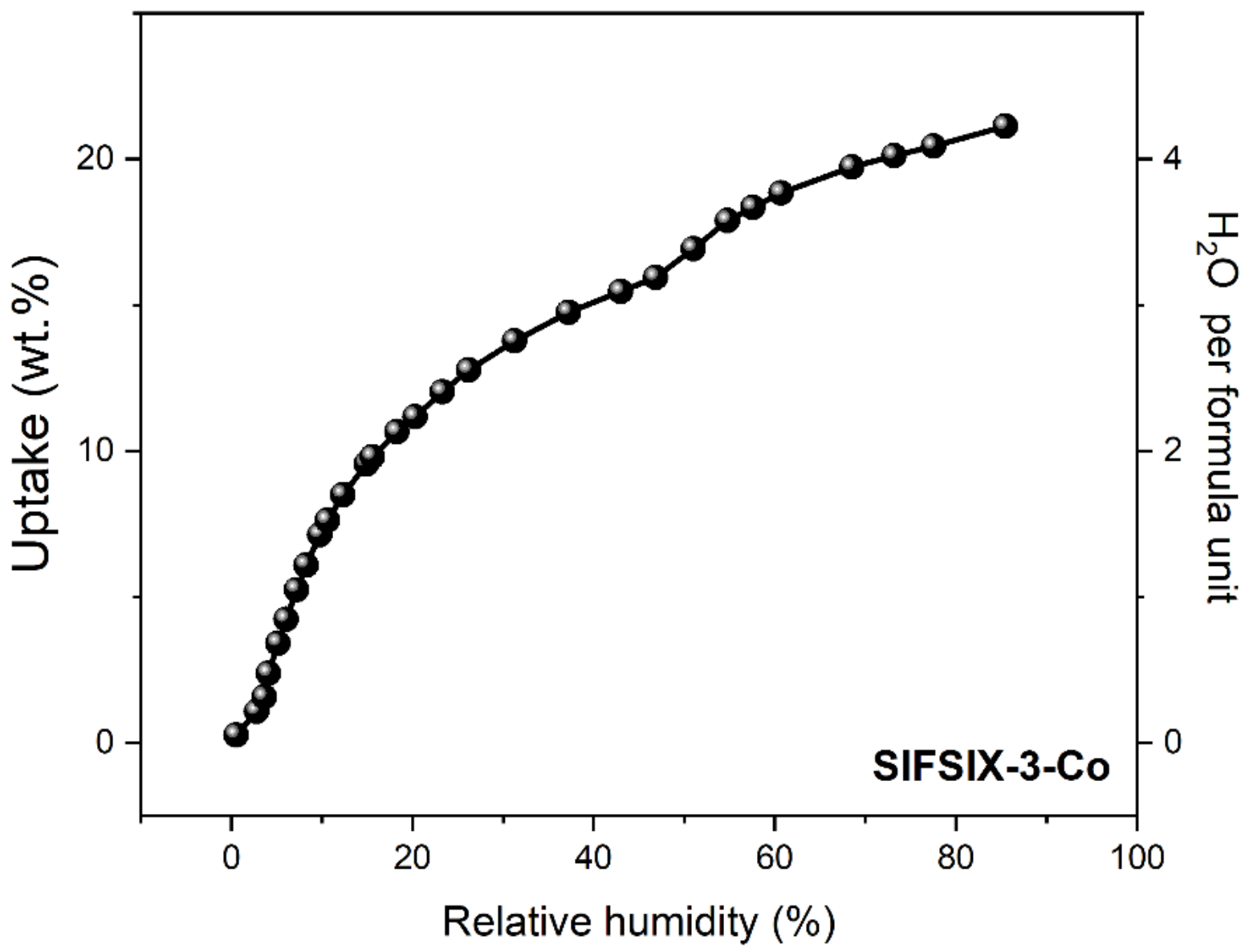

Figure S115 Water vapor adsorption (closed circles) and desorption (open circles) isotherm for **SIFSIX-3-Co** measured at 25 °C using BELSORP Max volumetric system.

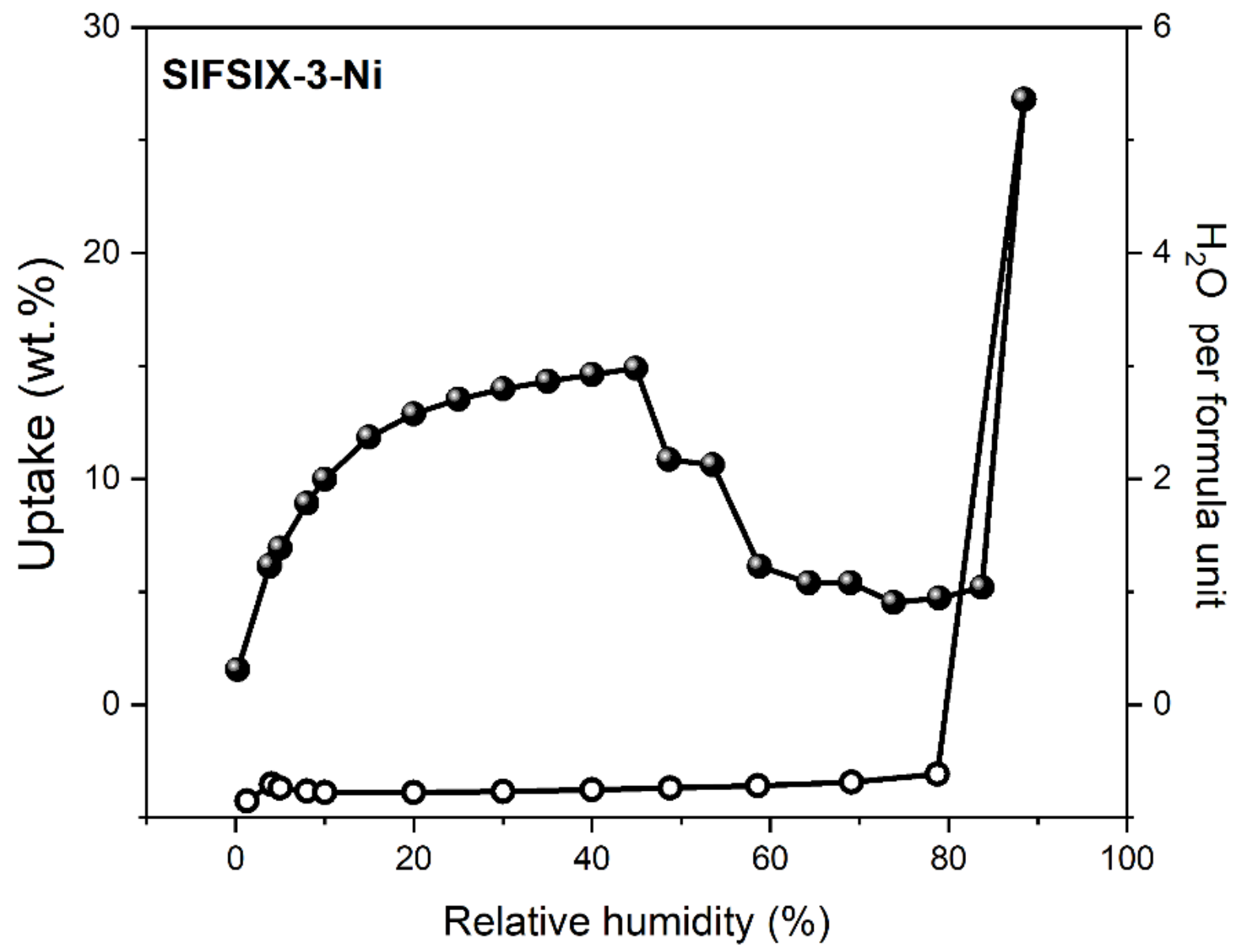

Figure S116 Water vapor adsorption (closed circles) and desorption (open circles) isotherm for **SIFSIX-3-Ni** measured at 25 °C using a DVS Vacuum system.

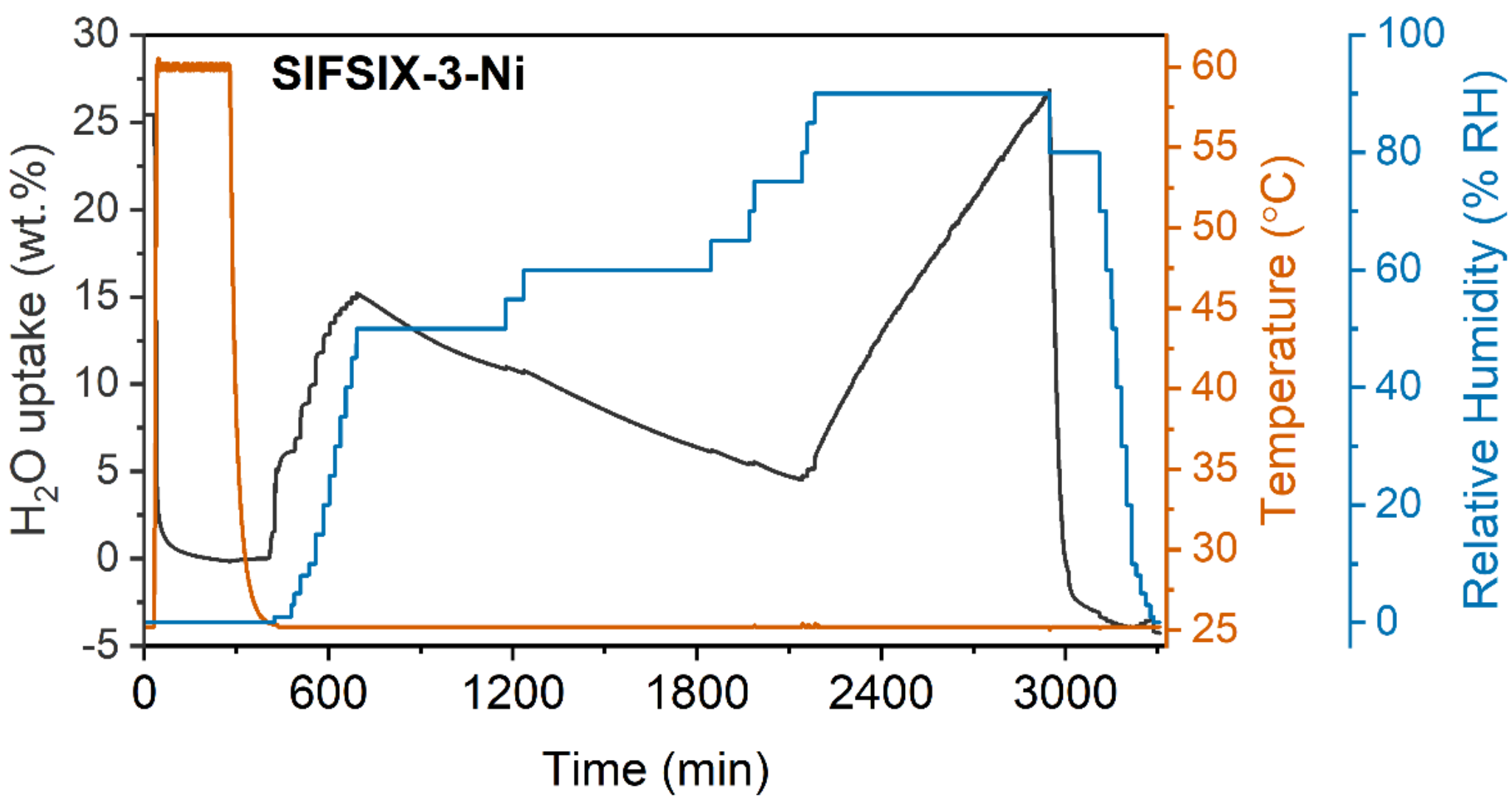

Figure S117 Dynamic water vapor sorption profile of **SIFSIX-3-Ni** displaying the mass change of **SIFSIX-3-Ni** with respect to time and relative humidity measured using a DVS Vacuum system.

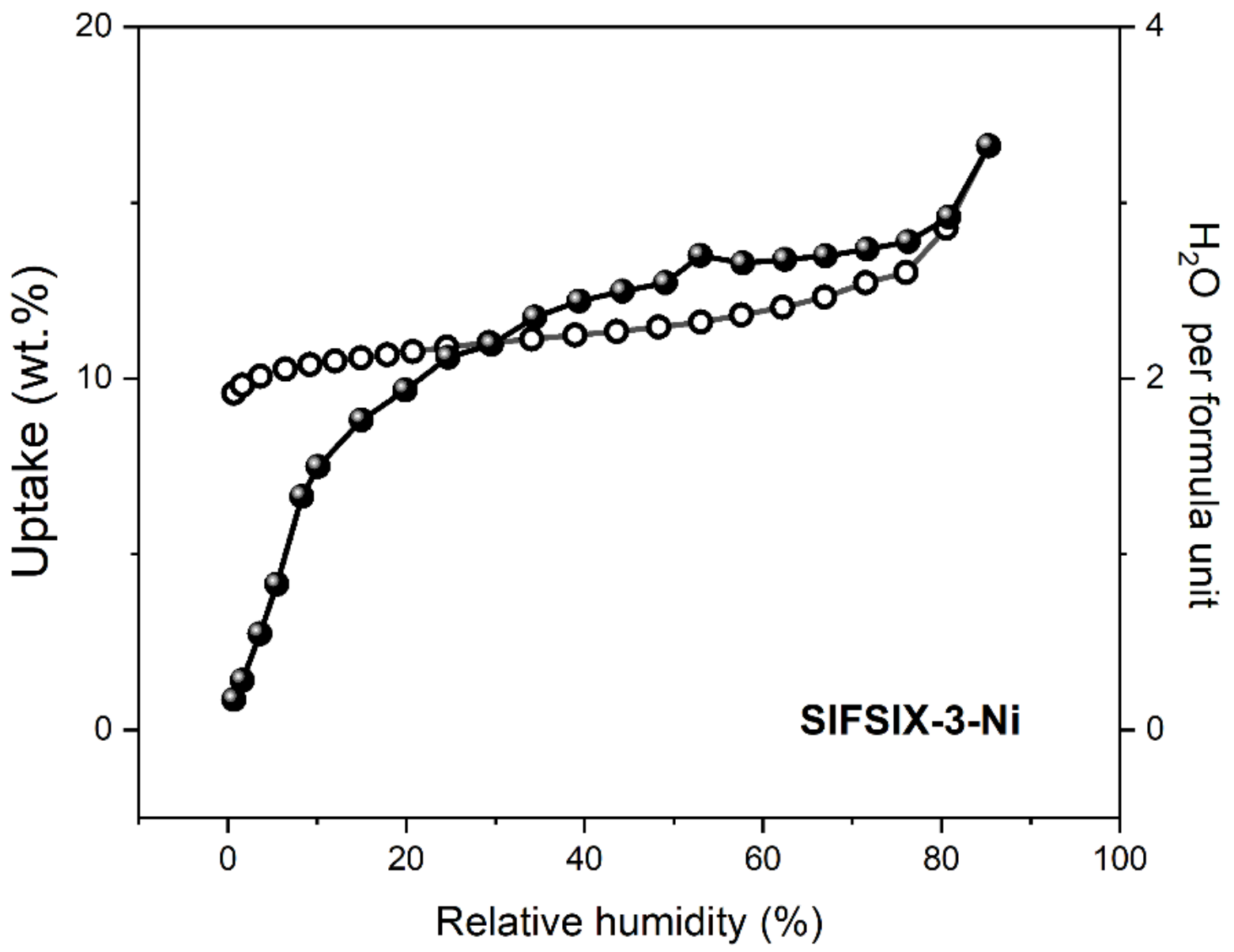

Figure S118 Water vapor adsorption (closed circles) and desorption (open circles) isotherm for **SIFSIX-3-Ni** measured at 25 °C using a DVS Adventure system.

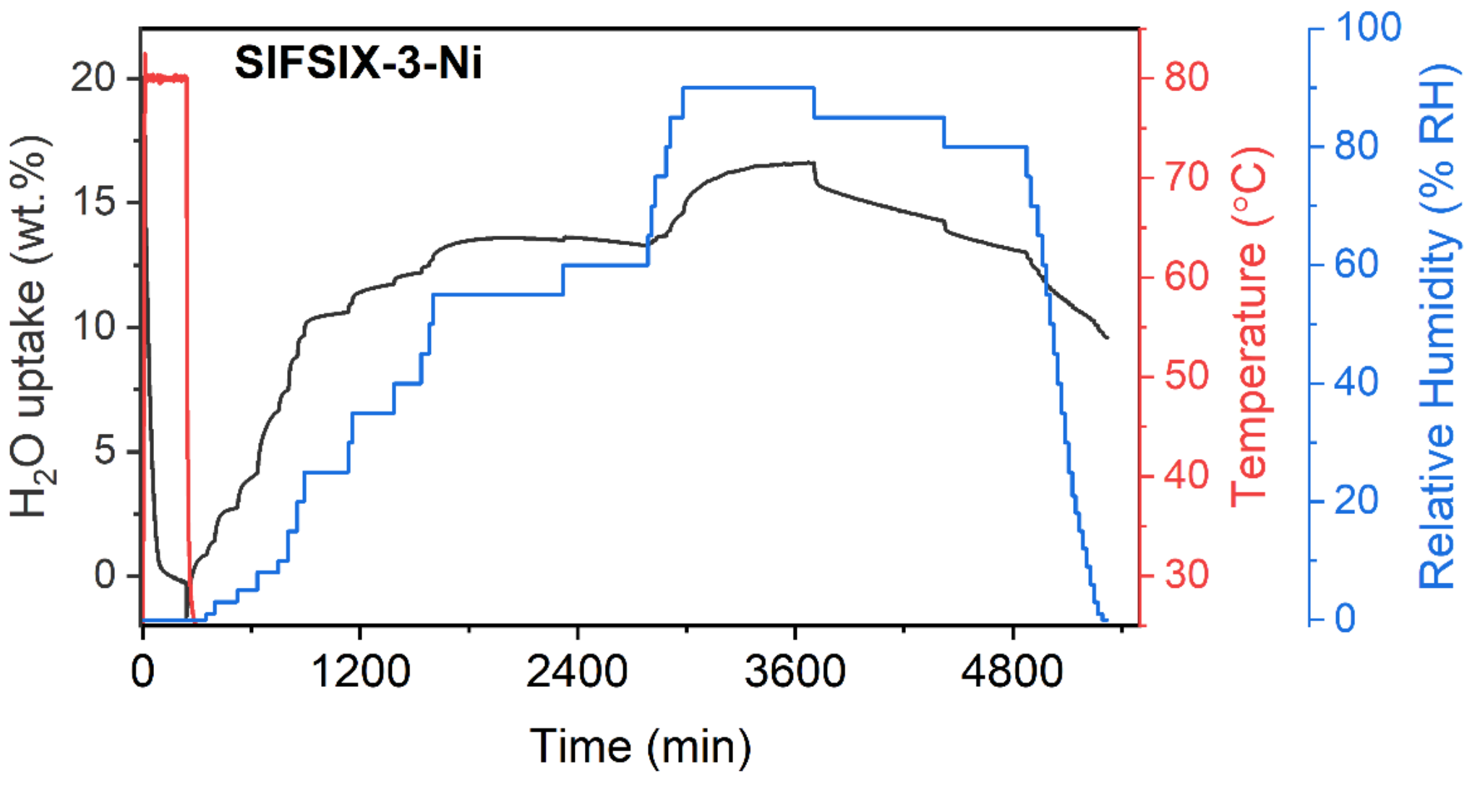

Figure S119 Dynamic water vapor sorption profile of **SIFSIX-3-Ni** displaying the mass change of **SIFSIX-3-Ni** with respect to time and relative humidity measured using a DVS Adventure system.

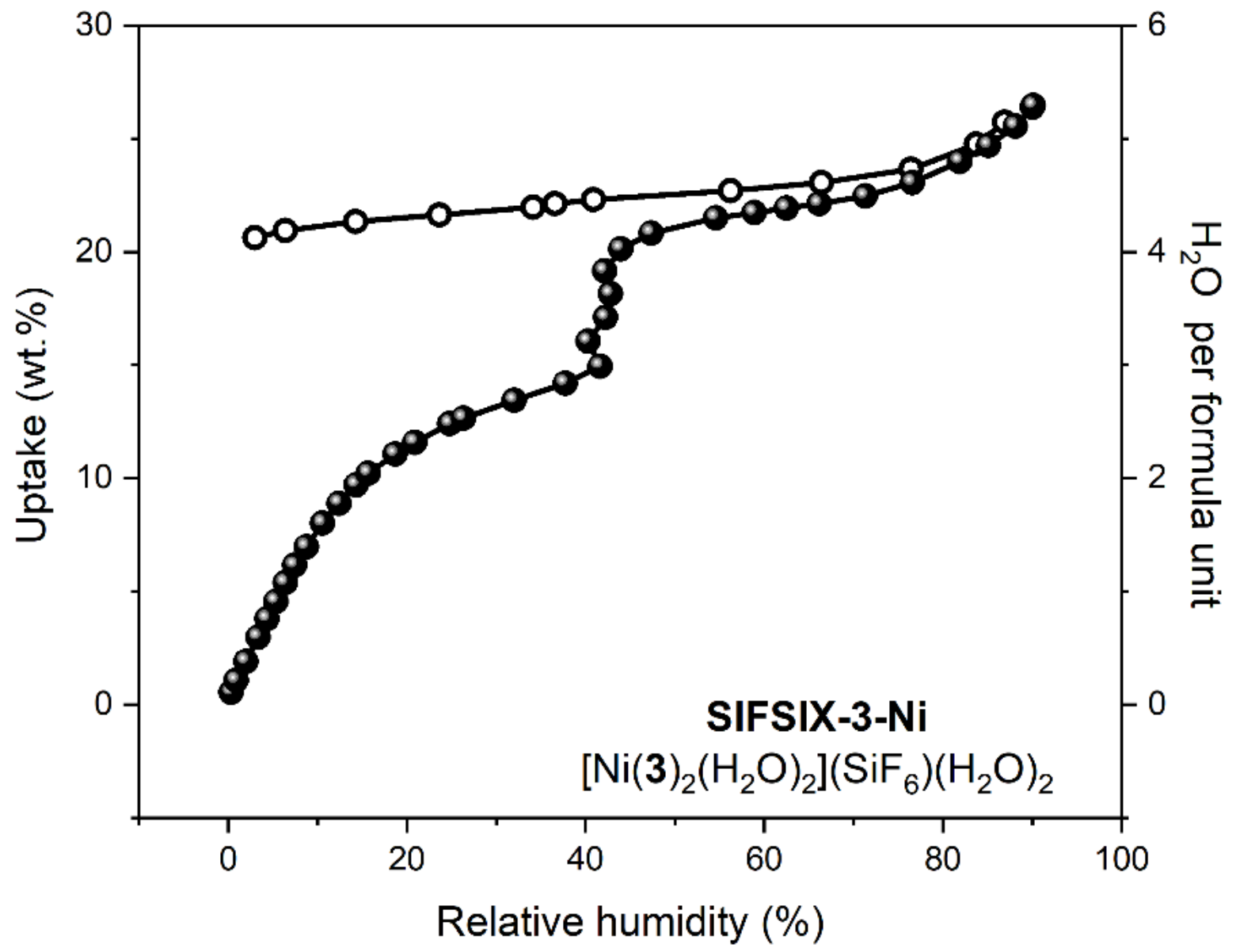

Figure S120 Water vapor adsorption (closed circles) and desorption (open circles) isotherm for **SIFSIX-3-Ni** measured at 25 °C using BELSORP Max volumetric system.

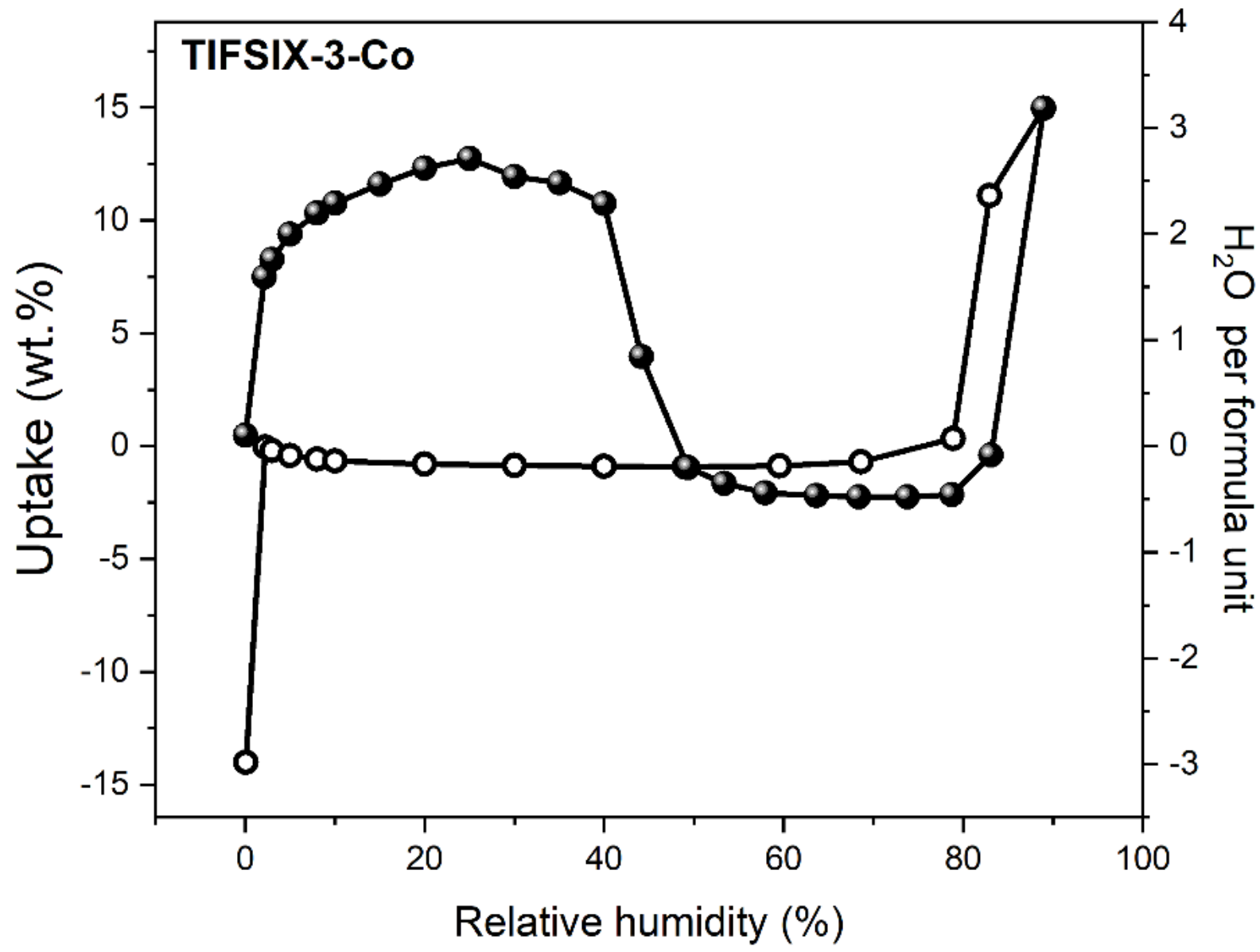

Figure S121 Water vapor adsorption (closed circles) and desorption (open circles) isotherm for **TIFSIX-3-Co** measured at 25 °C using a DVS Vacuum system.

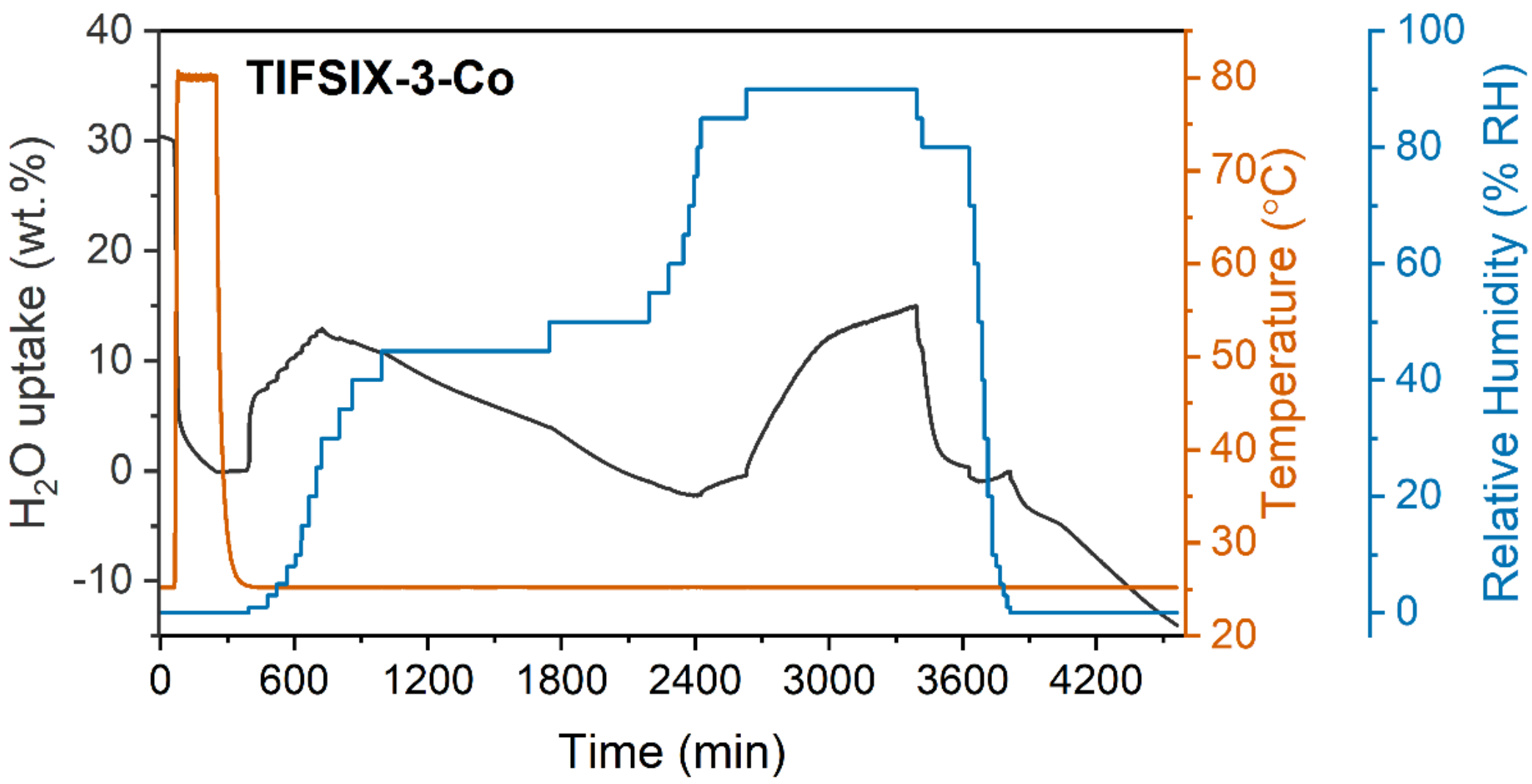

Figure S122 Dynamic water vapor sorption profile of **TFSIX-3-Co** displaying the mass change of **TIFSIX-3-Co** with respect to time and relative humidity measured using a DVS Vacuum system.

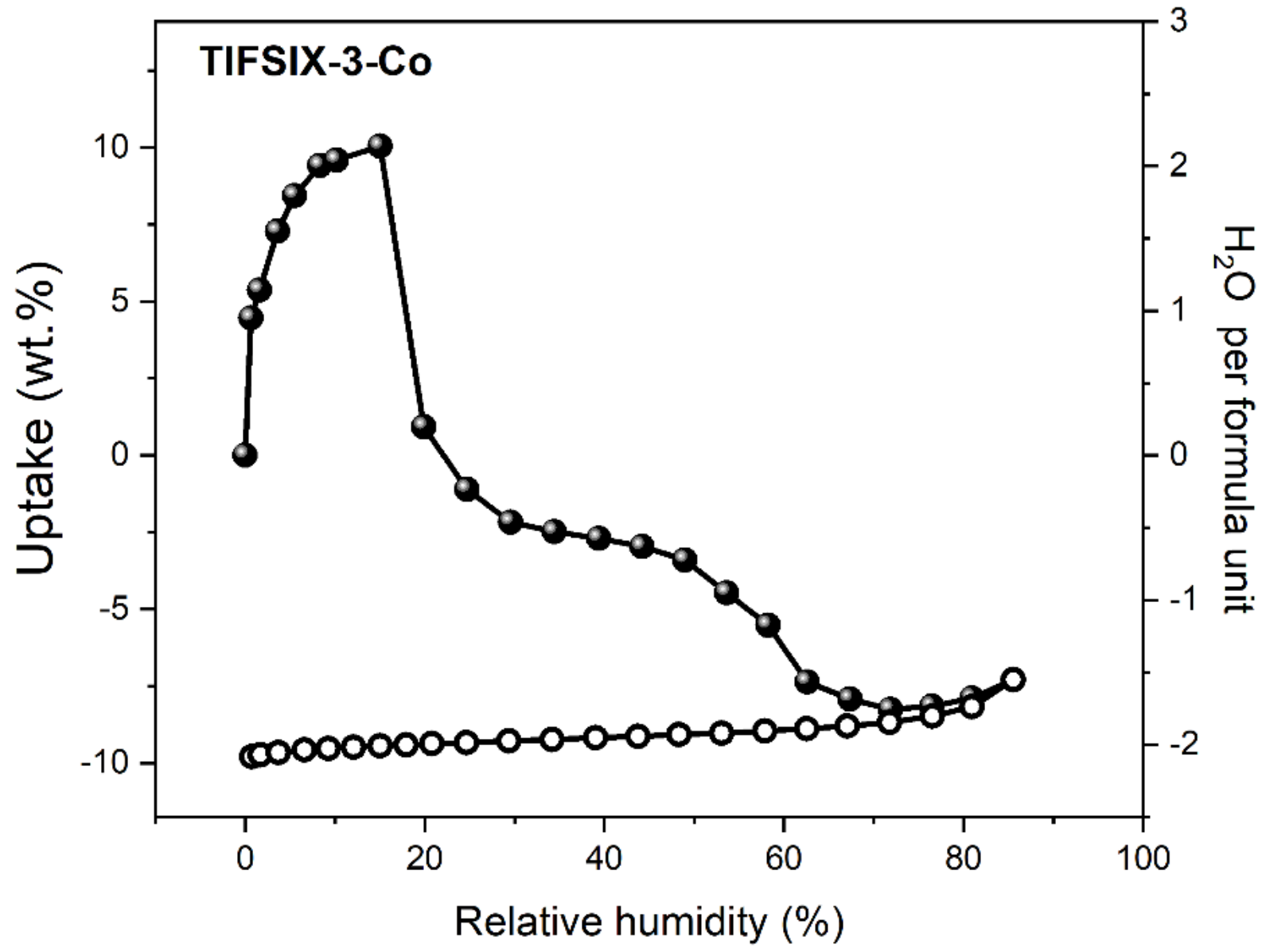

Figure S123 Water vapor adsorption (closed circles) and desorption (open circles) isotherm for **TIFSIX-3-Co** measured at 25 °C using a DVS Adventure system.

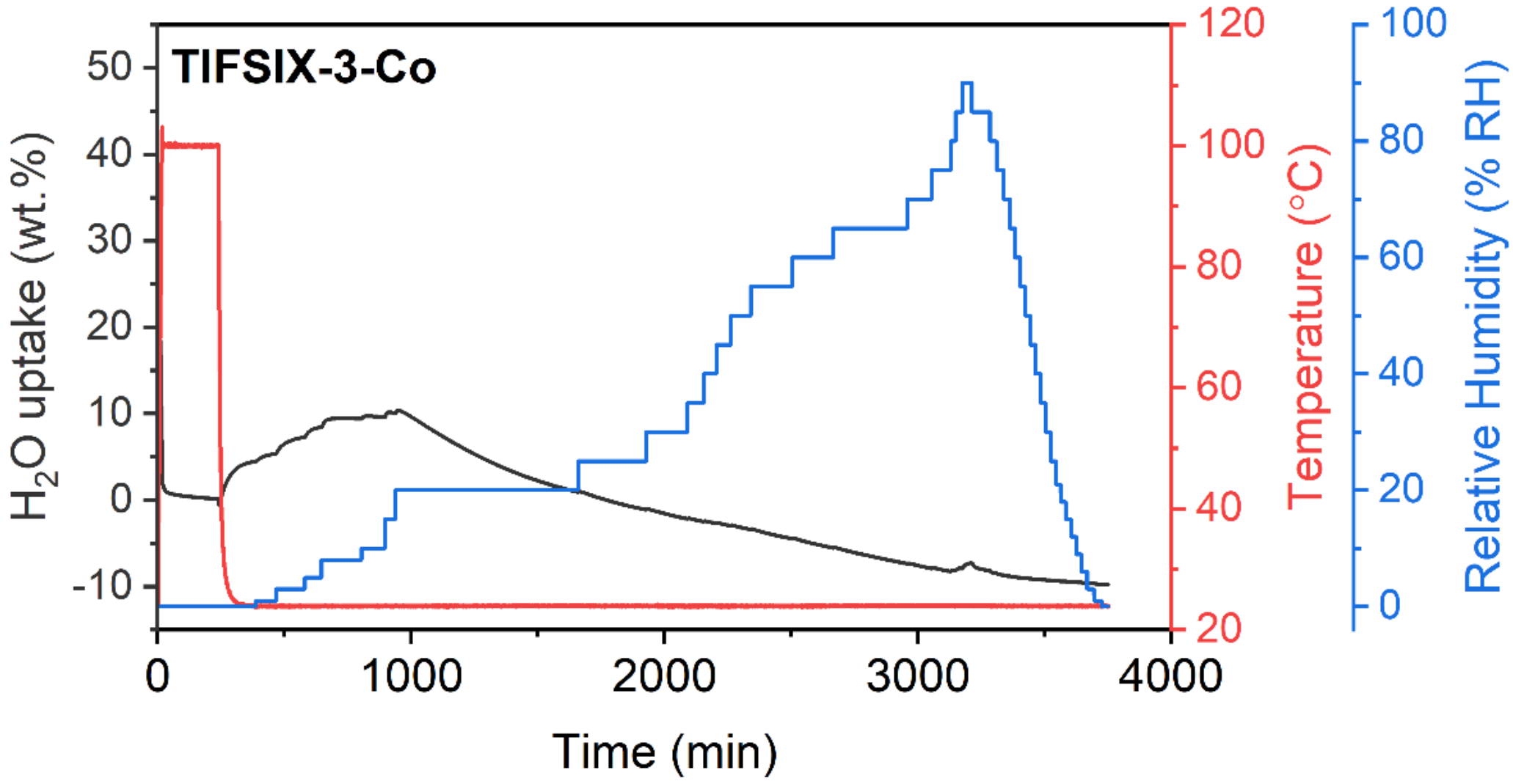

Figure S124 Dynamic water vapor sorption profile of **TIFSIX-3-Co** displaying the mass change of **TIFSIX-3-Co** with respect to time and relative humidity measured using a DVS Adventure system.

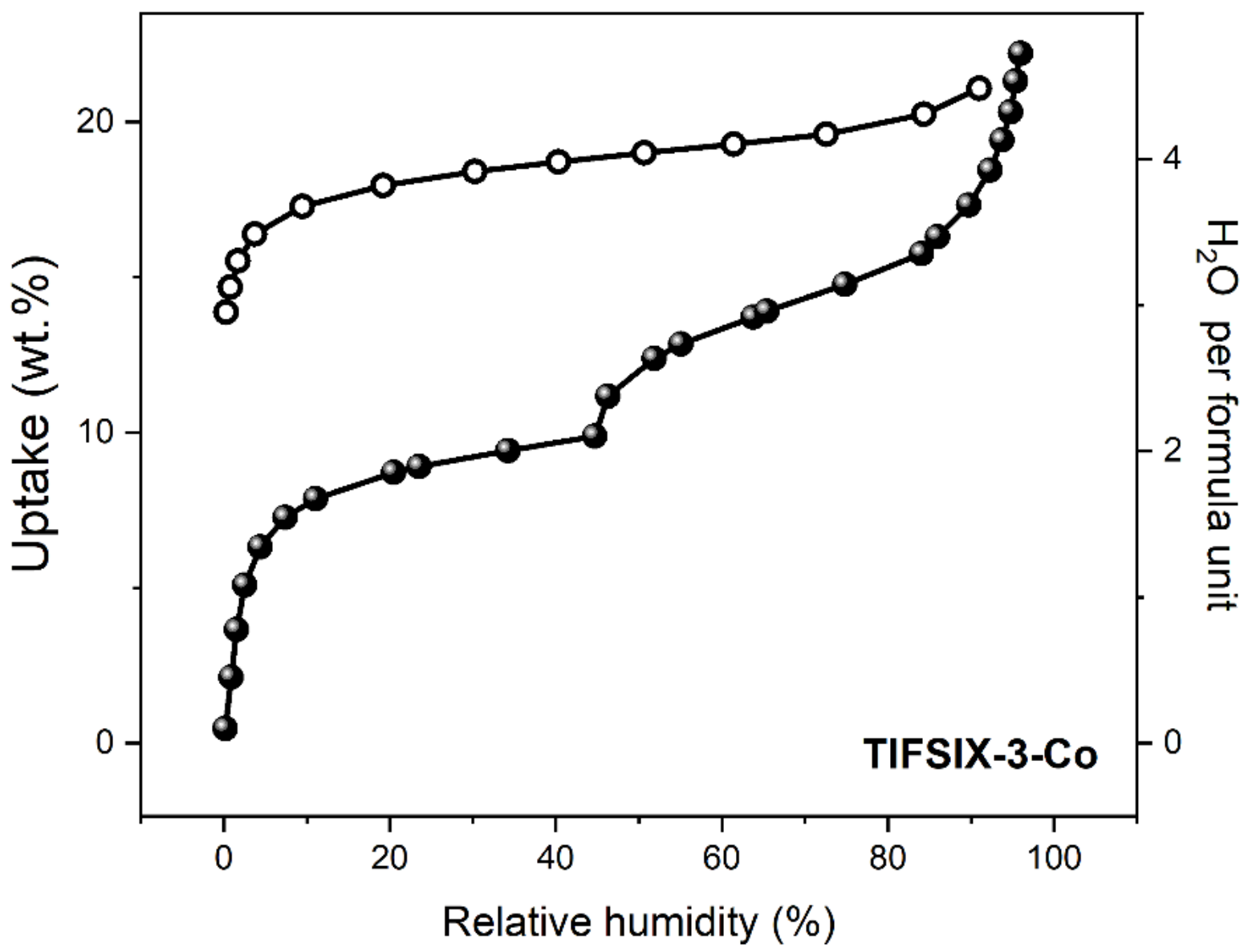

Figure S125 Water vapor adsorption (closed circles) and desorption (open circles) isotherm for **TIFSIX-3-Co** measured at 25 °C using BELSORP Max volumetric system.

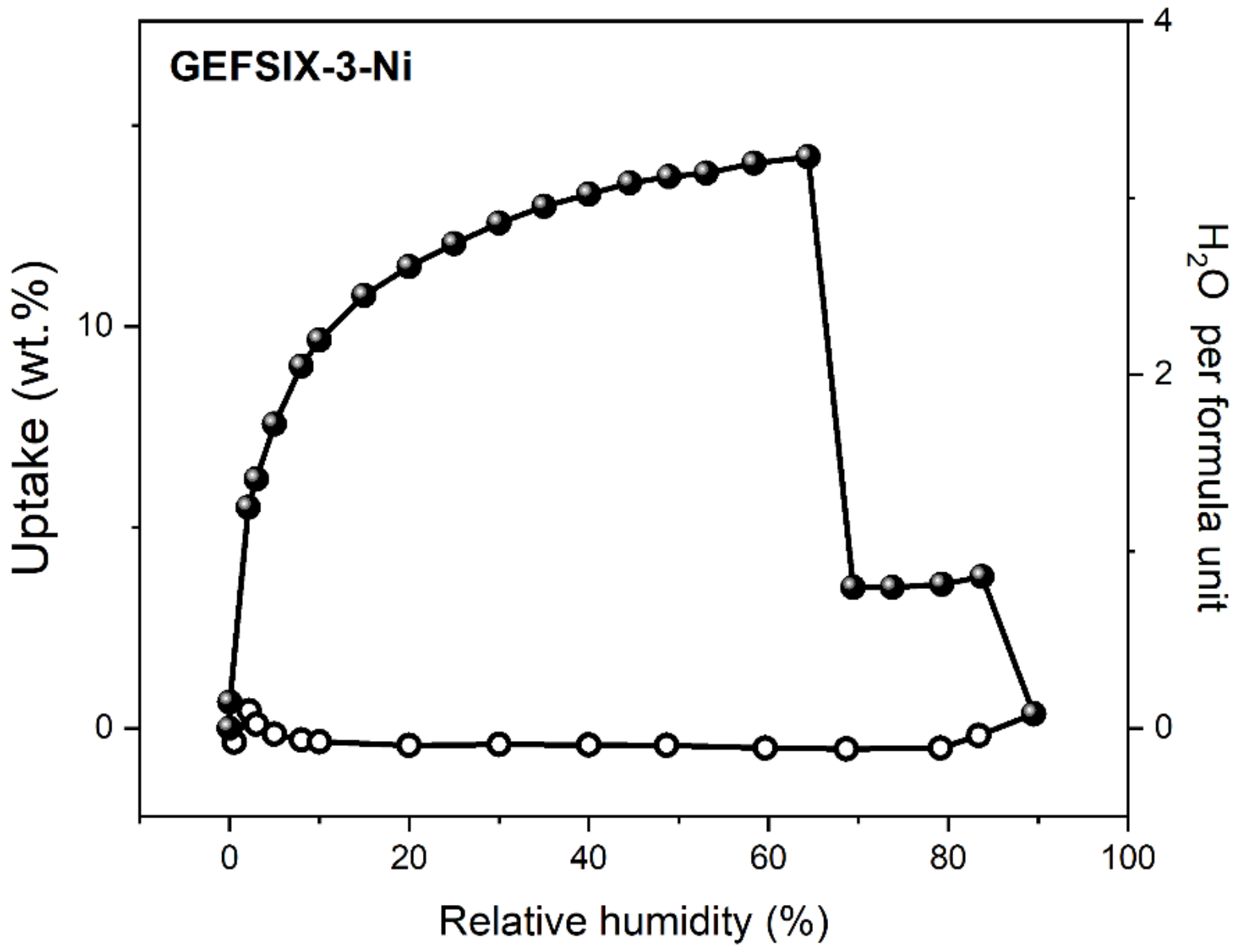

Figure S126 Water vapor adsorption (closed circles) and desorption (open circles) isotherm for **GEFSIX-3-Ni** measured at 25 °C using a DVS Vacuum system.

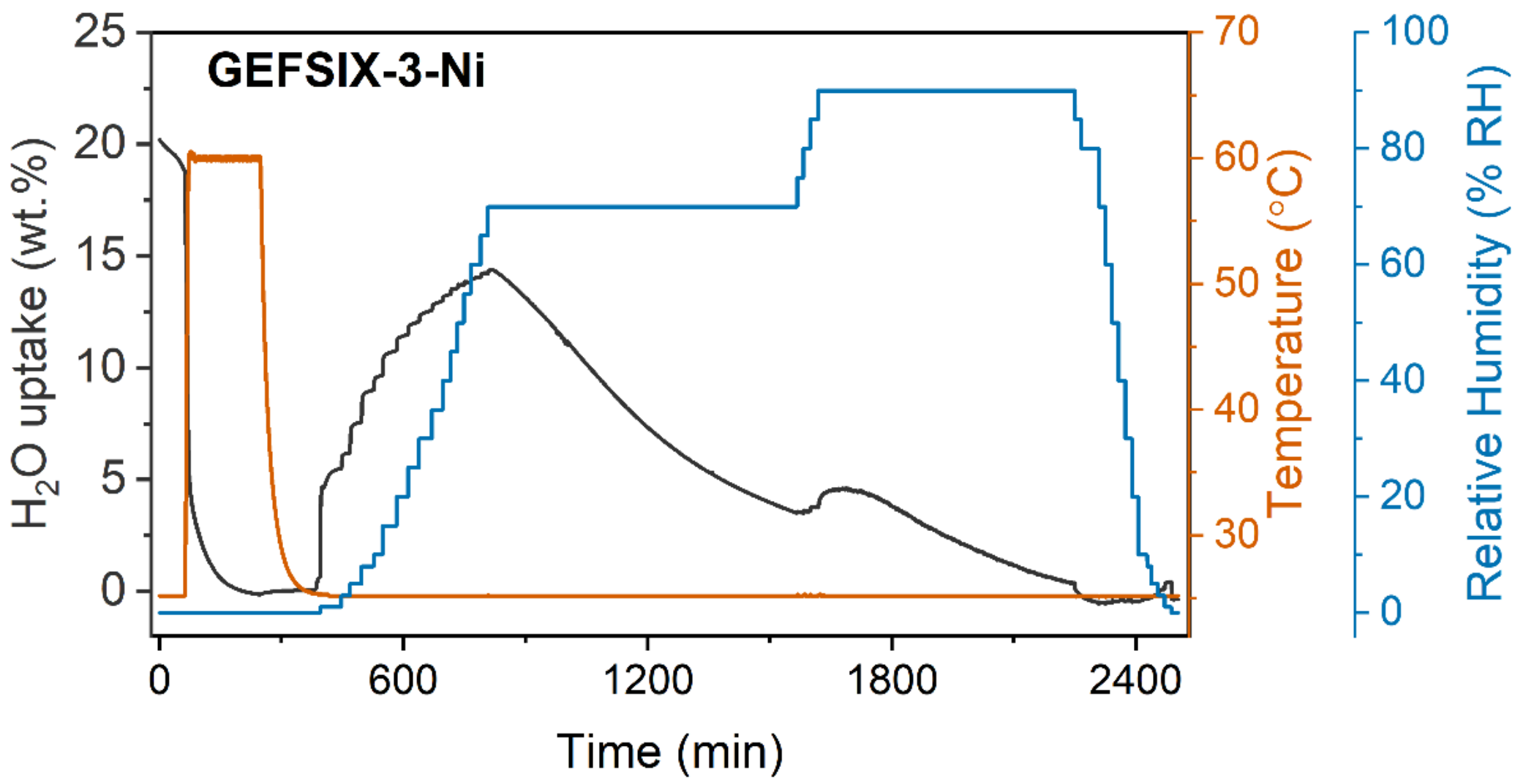

Figure S127 Dynamic water vapor sorption profile of **GEFSIX-3-Ni** displaying the mass change of **GEFSIX-3-Ni** with respect to time and relative humidity measured using a DVS Vacuum system.

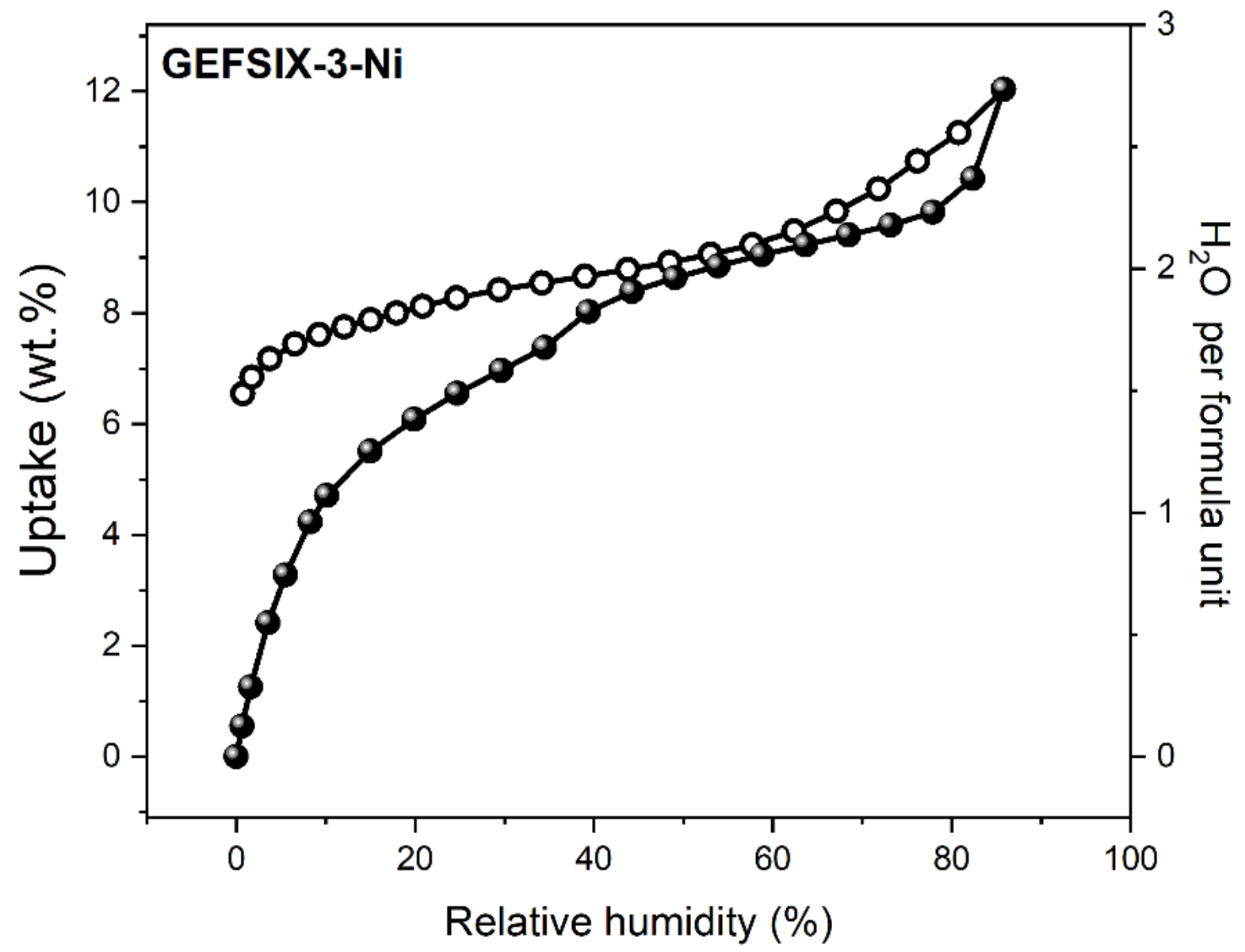

Figure S128 Water vapor adsorption (closed circles) and desorption (open circles) isotherm for **GEFSIX-3-Ni** measured at 25 °C using a DVS Adventure system.

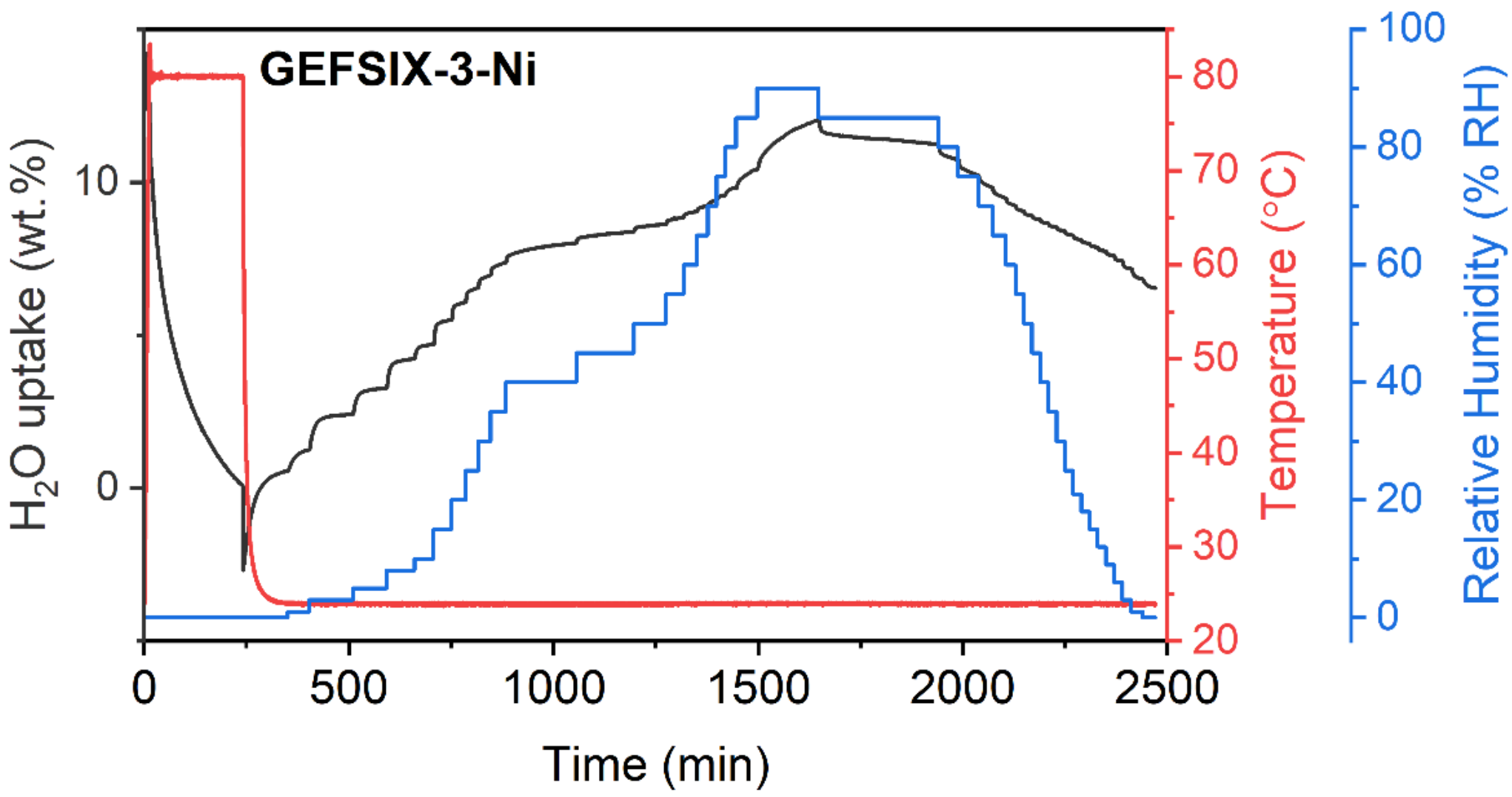

Figure S129 Dynamic water vapor sorption profile of **GEFSIX-3-Ni** displaying the mass change of **GEFSIX-3-Ni** with respect to time and relative humidity measured using a DVS Adventure system.

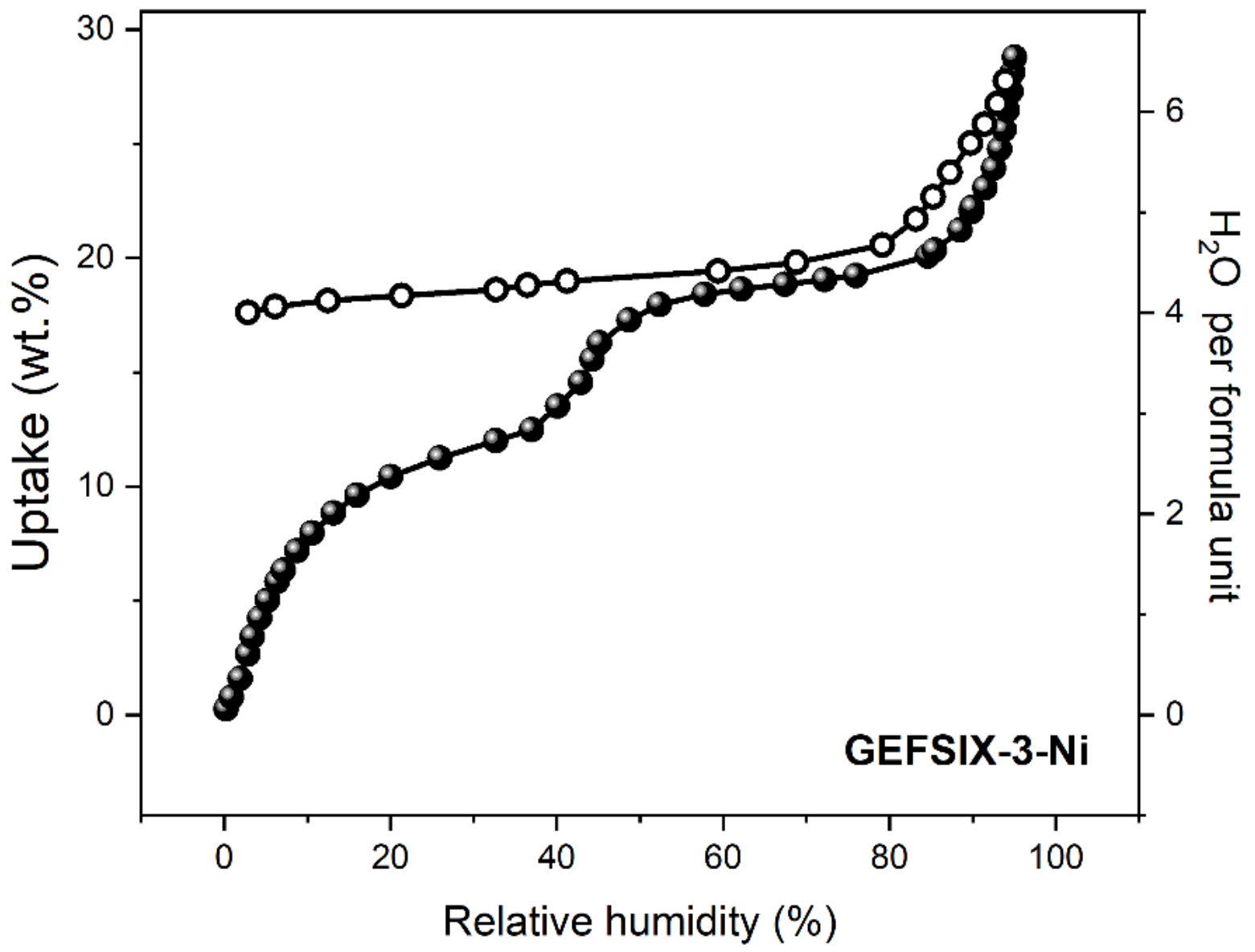

Figure S130 Water vapor adsorption (closed circles) and desorption (open circles) isotherm for **GEFSIX-3-Ni** measured at 25 °C using BELSORP Max volumetric system.

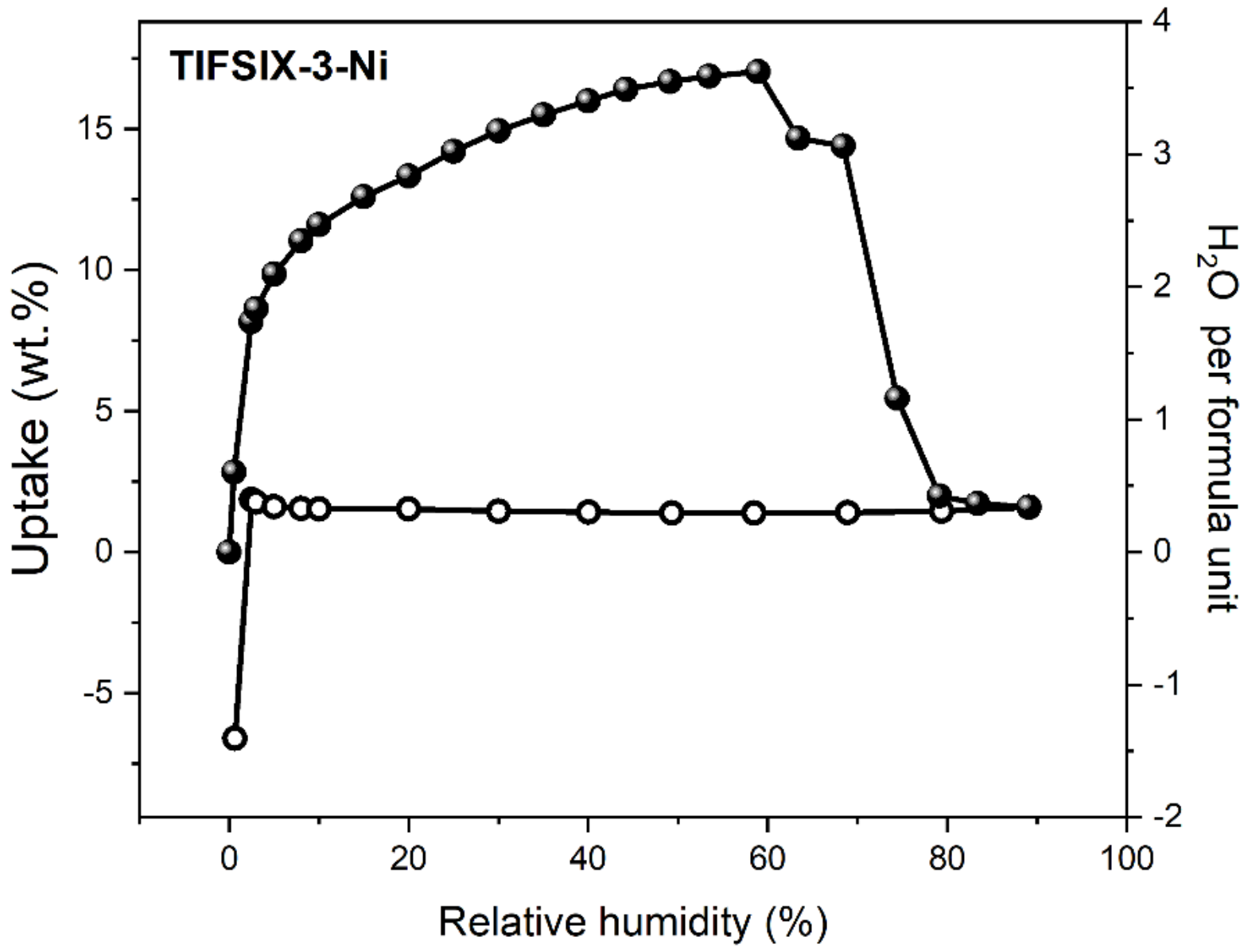

Figure S131 Water vapor adsorption (closed circles) and desorption (open circles) isotherm for **TIFSIX-3-Ni** measured at 25 °C using a DVS Vacuum system.

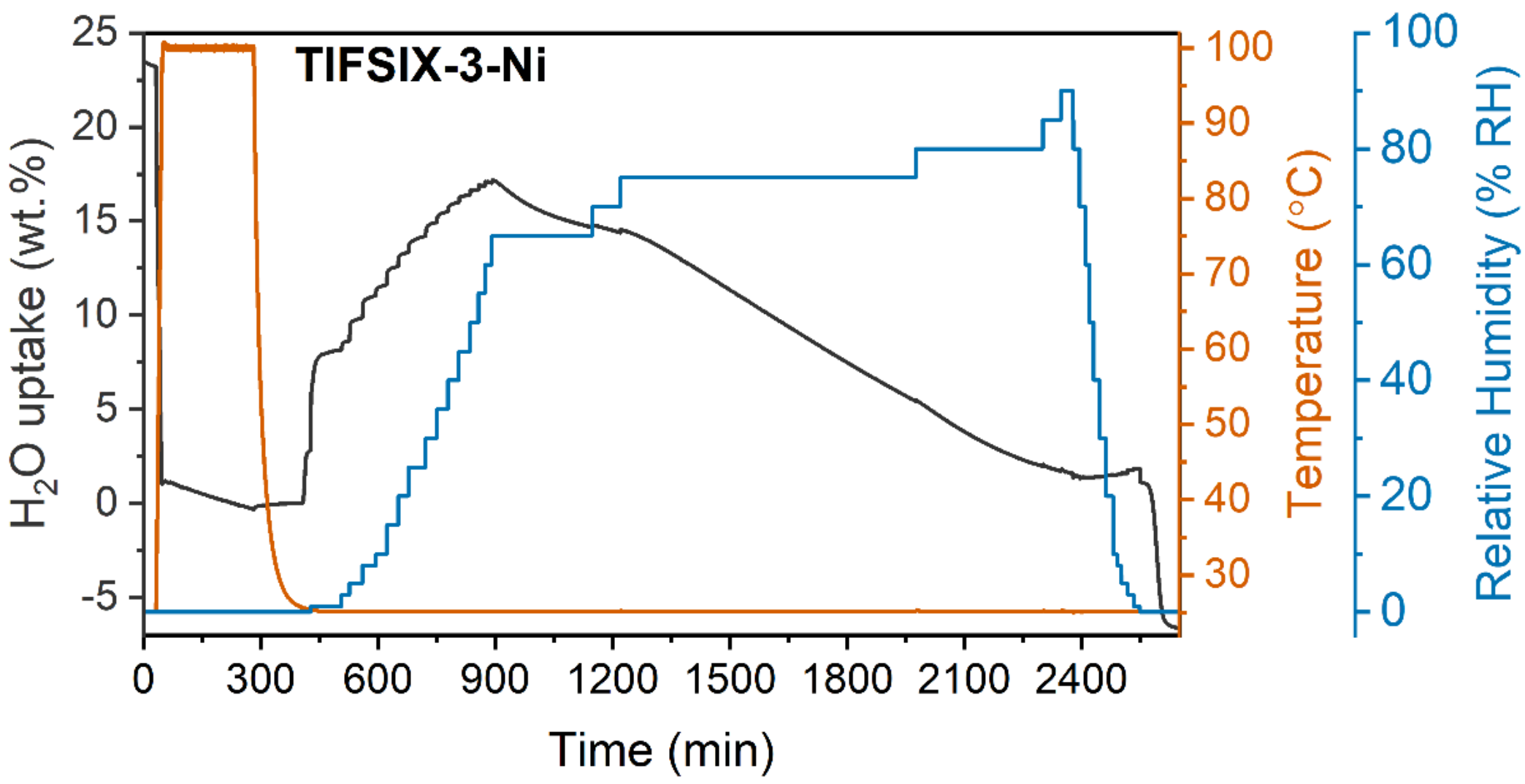

Figure S132 Dynamic water vapor sorption profile of **TIFSIX-3-Ni** displaying the mass change of **TIFSIX-3-Ni** with respect to time and relative humidity measured using a DVS Vacuum system.

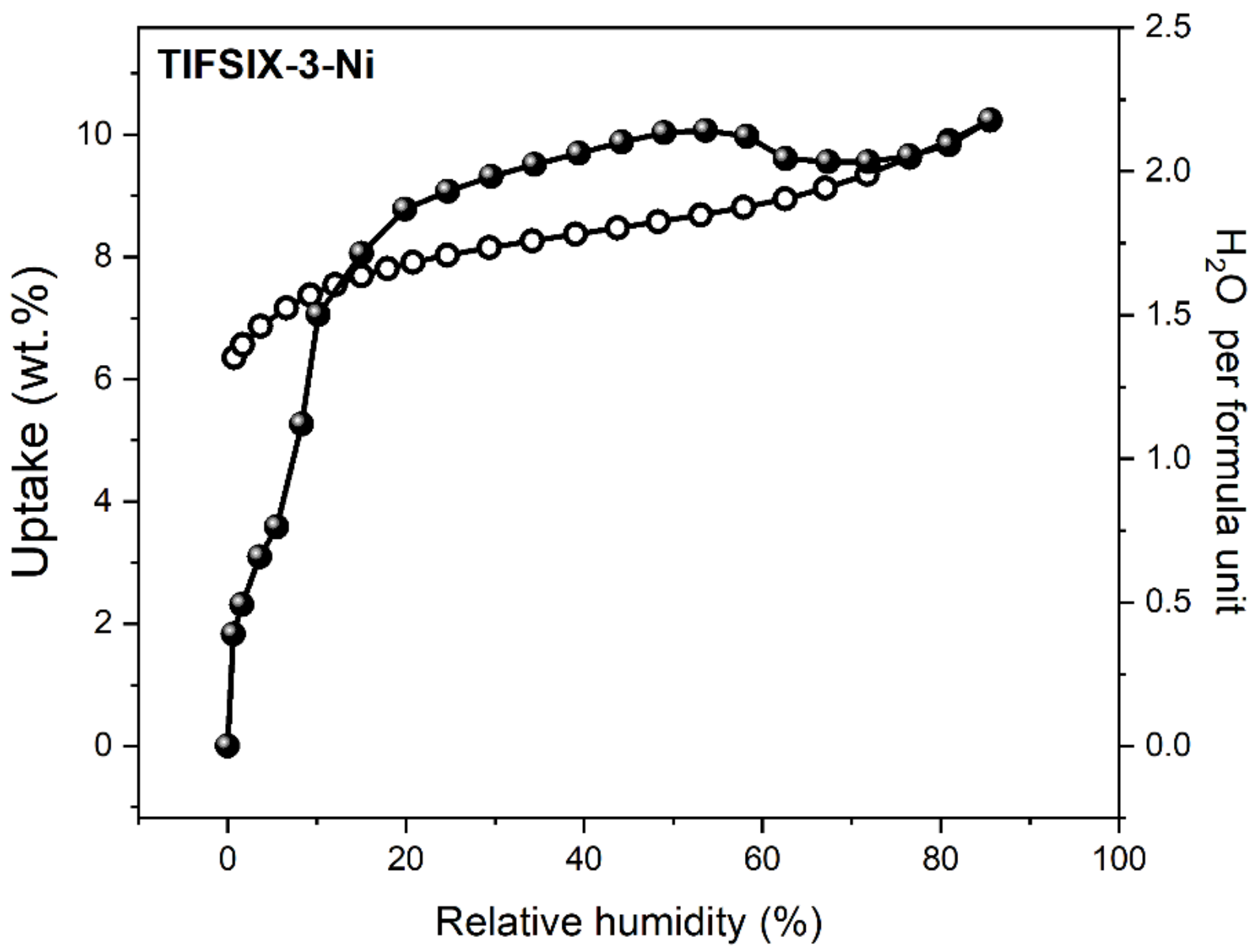

Figure S133 Water vapor adsorption (closed circles) and desorption (open circles) isotherm for **TIFSIX-3-Ni** measured at 25 °C using a DVS Adventure system.

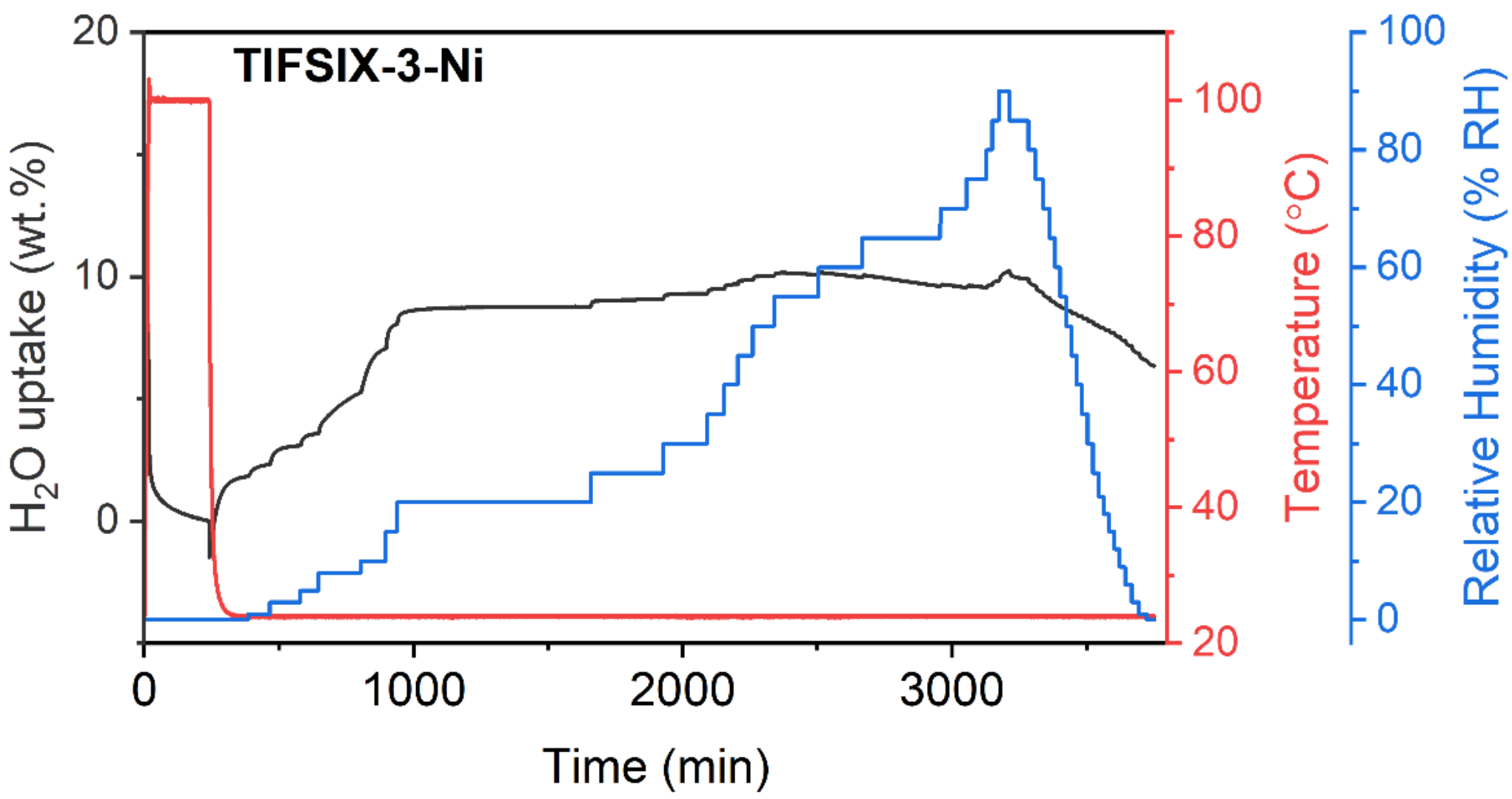

Figure S134 Dynamic water vapor sorption profile of **TIFSIX-3-Ni** displaying the mass change of **TIFSIX-3-Ni** with respect to time and relative humidity measured using a DVS Adventure system.

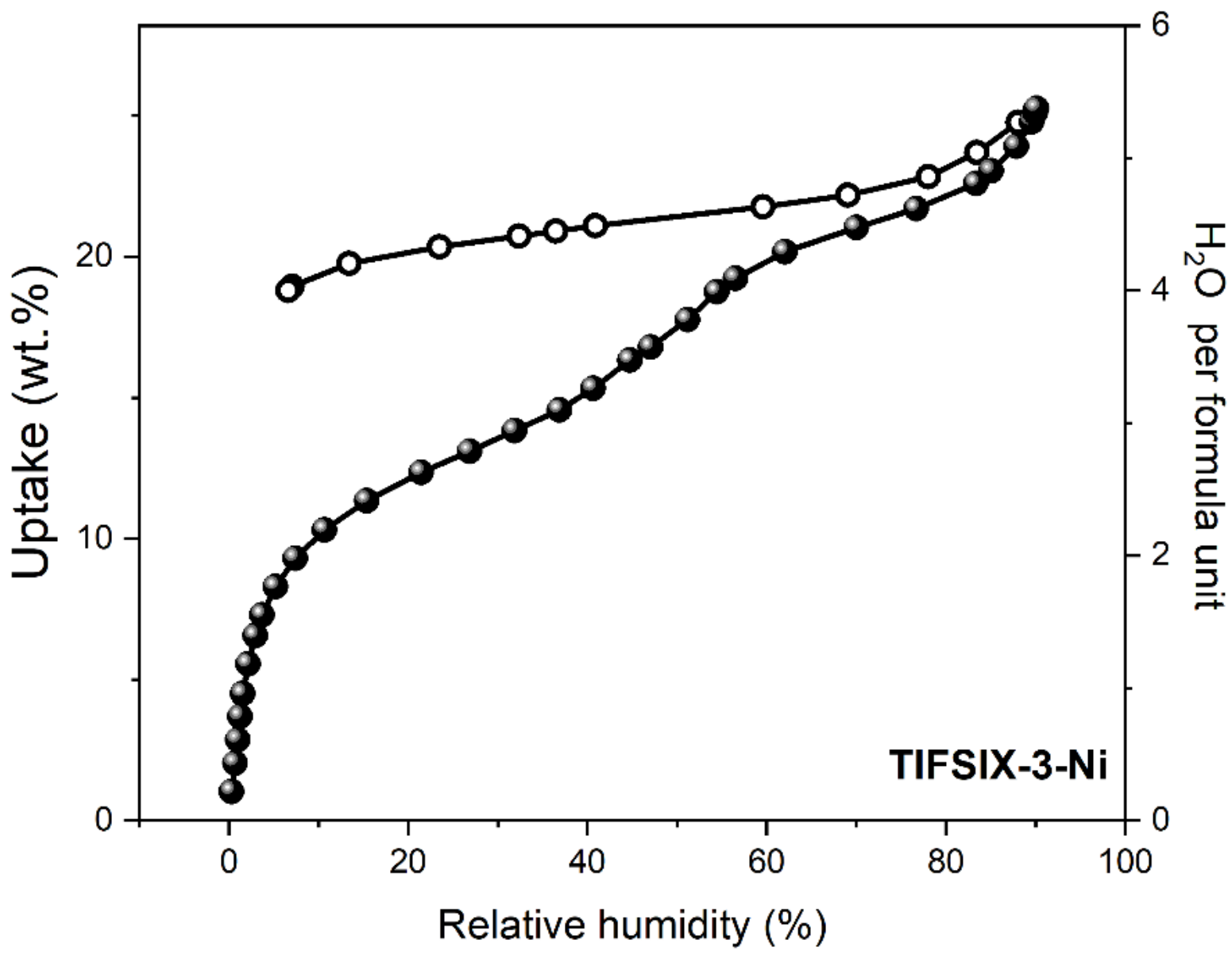

Figure S135 Water vapor adsorption (closed circles) and desorption (open circles) isotherm for **TIFSIX-3-Ni** measured at 25 °C using BELSORP Max volumetric system.

Table S8 Parameters describing the inflection region determined from volumetric vacuum sorption data: onset RH of the inflection point (RH_i), the RH at the end of the inflection region (RH_f) and α, defined as the RH at the midpoint uptake between RH_i and RH_f.

Material	RH_i (%)	RH_f (%)	α (%)
**SIFSIX-3-Ni**	41.6	48	42
**SIFSIX-3-Co**	46.8	85	63
**SIFSIX-3-Zn**	18.3	55	30
**TIFSIX-3-Ni**	36	55	45
**TIFSIX-3-Co**	45.0	90	75
**GEFSIX-3-Ni**	37	50	40
**GEFSIX-3-Co**	37.0	67	42

13 Variable-humidity powder X-ray diffraction

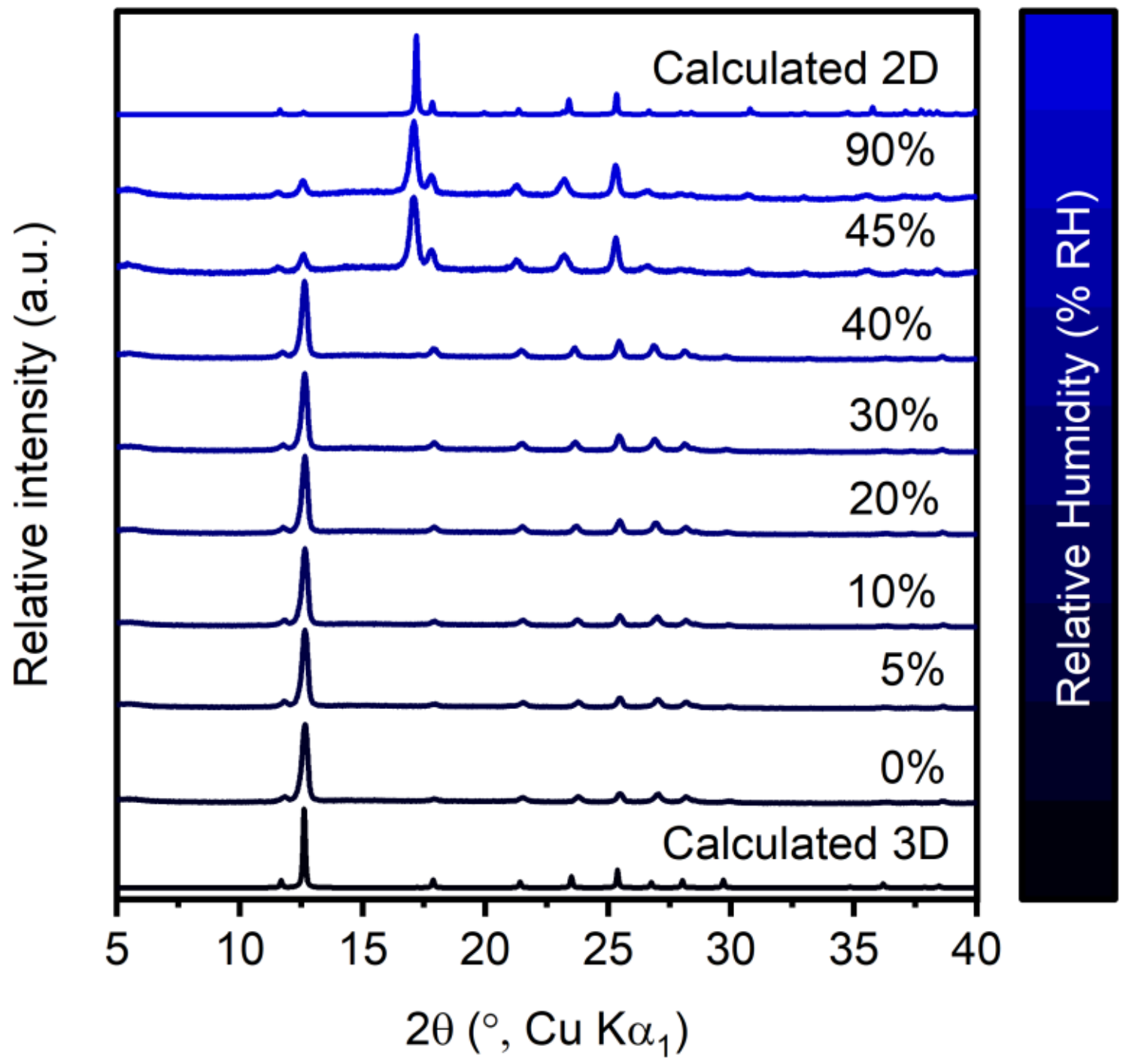

Figure S136 Variable-humidity PXRD patterns showing activated (3D) structure **pcu-SIFSIX-3-Ni** phase (CSD refcode **DUMFEY**) converting to 2D **sql** phase (**sql-SIFSIX-3-Ni**).

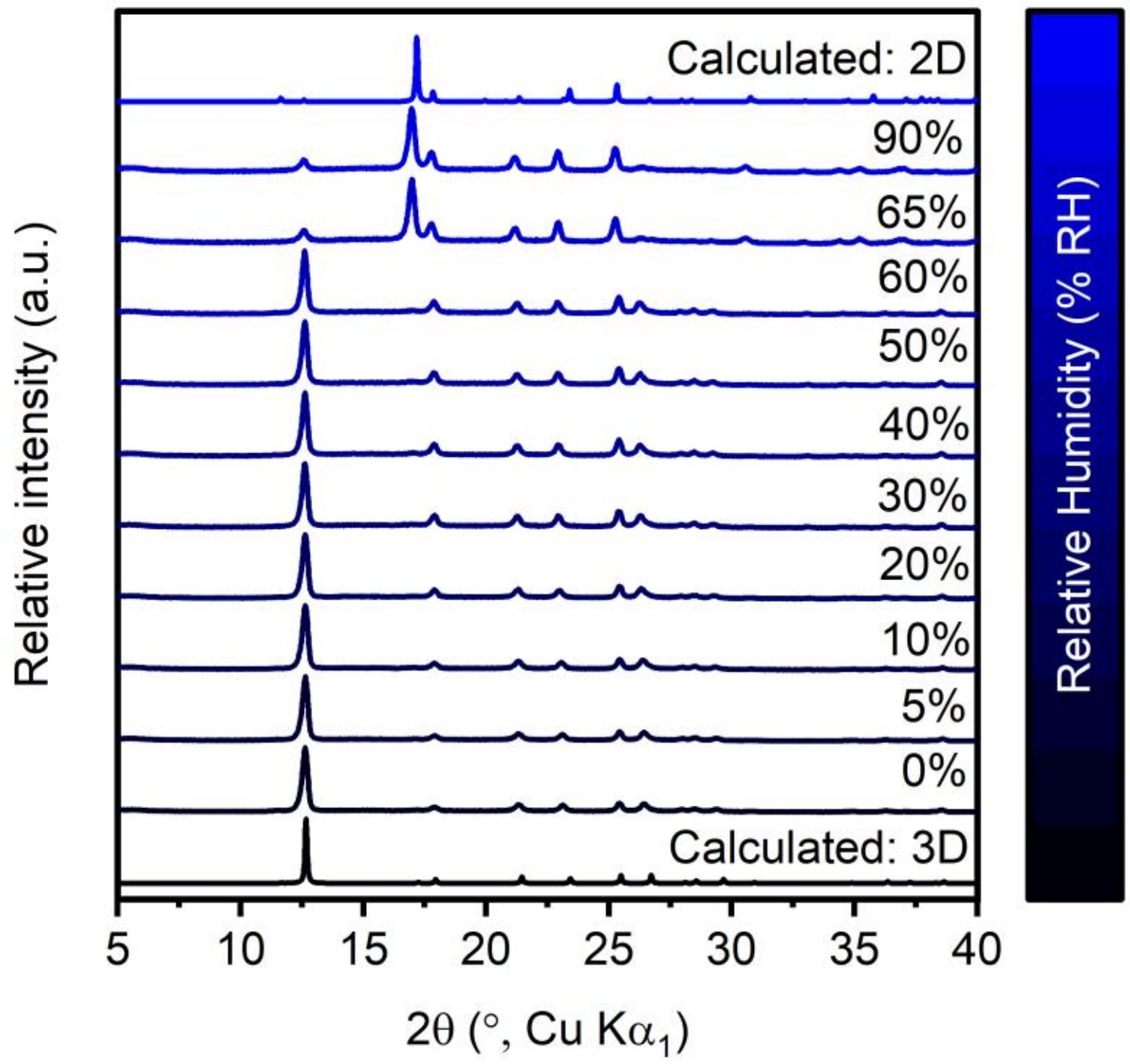

Figure S137 Variable-humidity PXRD patterns showing activated (3D) structure **pcu-GEFSIX-3-Ni** phase (CSD refcode **ZUTVAO**) converting to 2D **sql** phase (**sql-GEFSIX-3-Ni**).

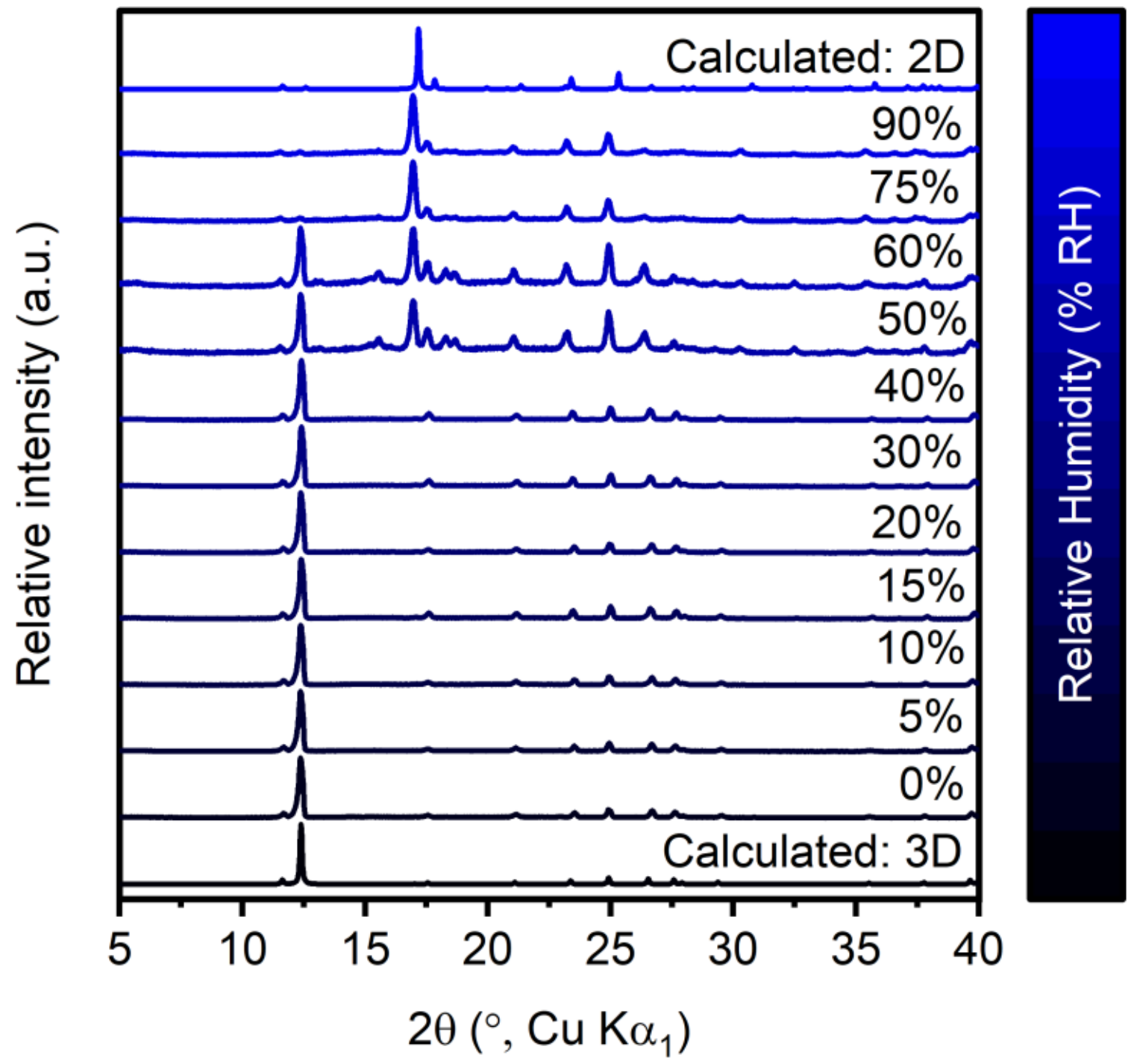

Figure S138 Variable-humidity PXRD patterns showing activated (3D) structure **pcu-SIFSIX-3-Zn** phase (CSD refcode **FUDQIF**) converting to 2D **sql** phase (**sql-SIFSIX-3-Zn**).

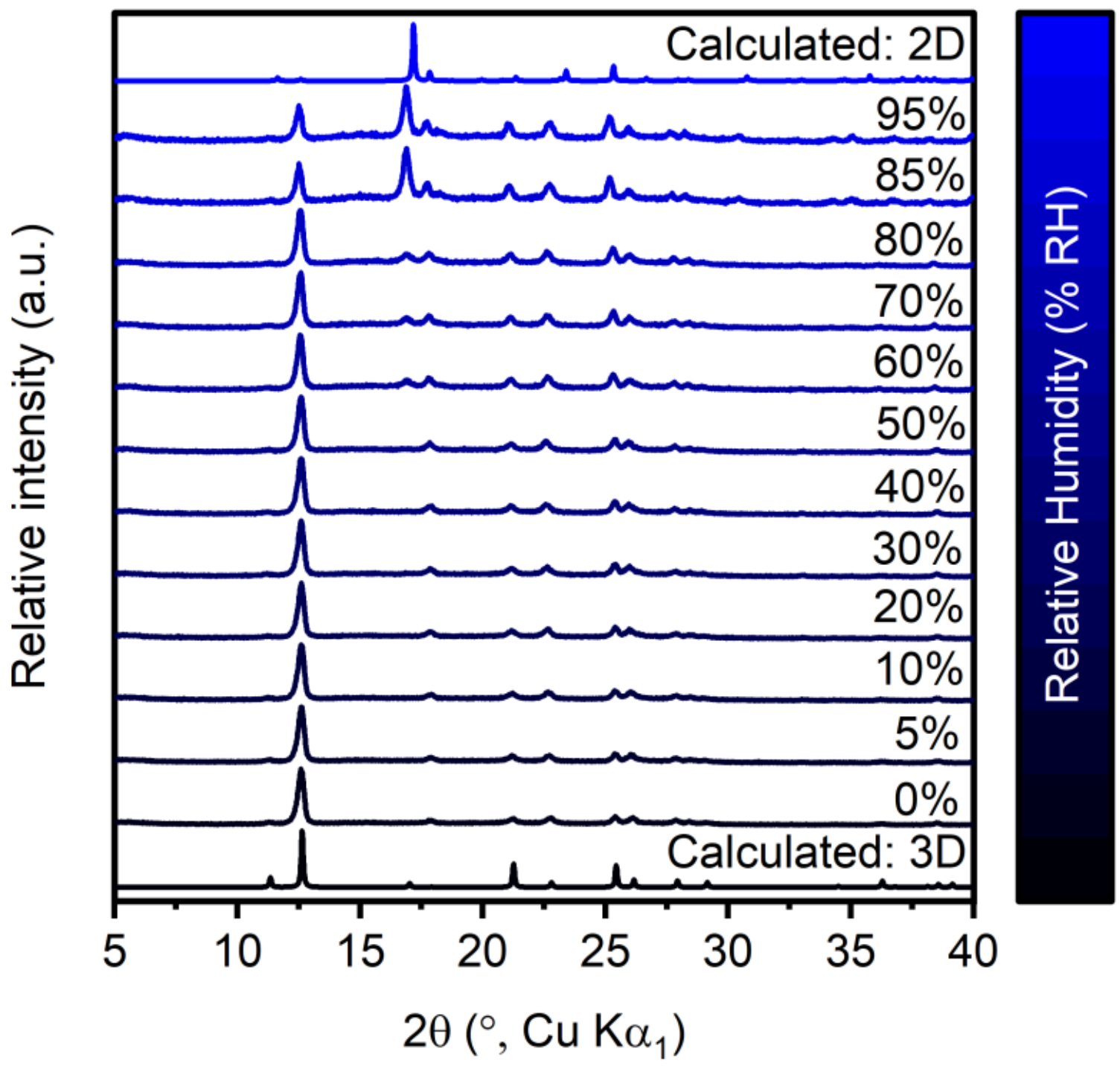

Figure S139 Variable-humidity PXRD patterns showing activated (3D) structure **pcu-TIFSIX-3-Ni** phase (CSD refcode **RIJGIE**) converting to 2D **sql** phase (**sql-TIFSIX-3-Ni**).

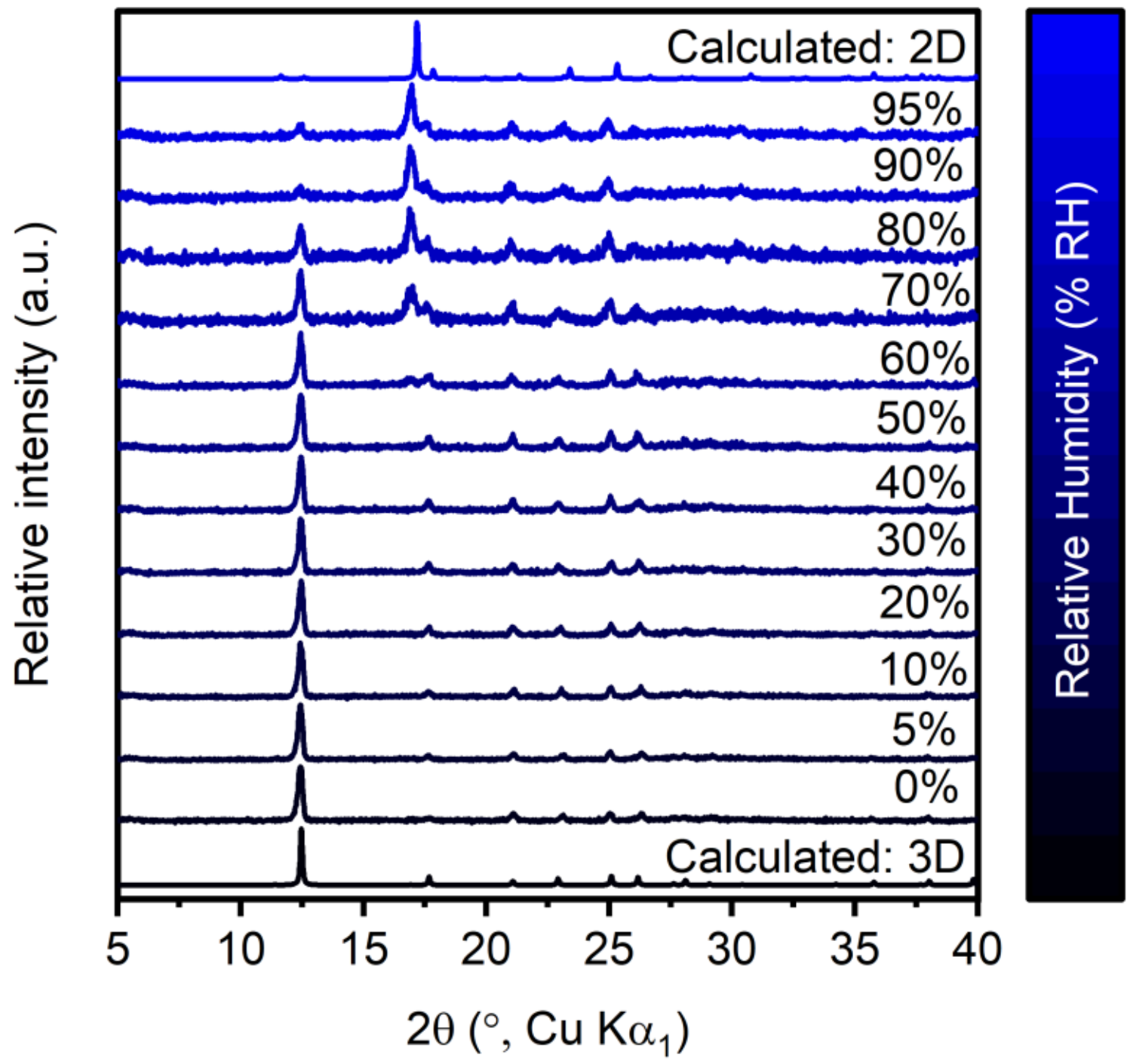

Figure S140 Variable-humidity PXRD patterns showing activated (3D) structure **pcu-GEFSIX-3-Co** phase (CSD refcode **HOYXIF**) converting to 2D **sql** phase (**sql-GEFSIX-3-Co**).

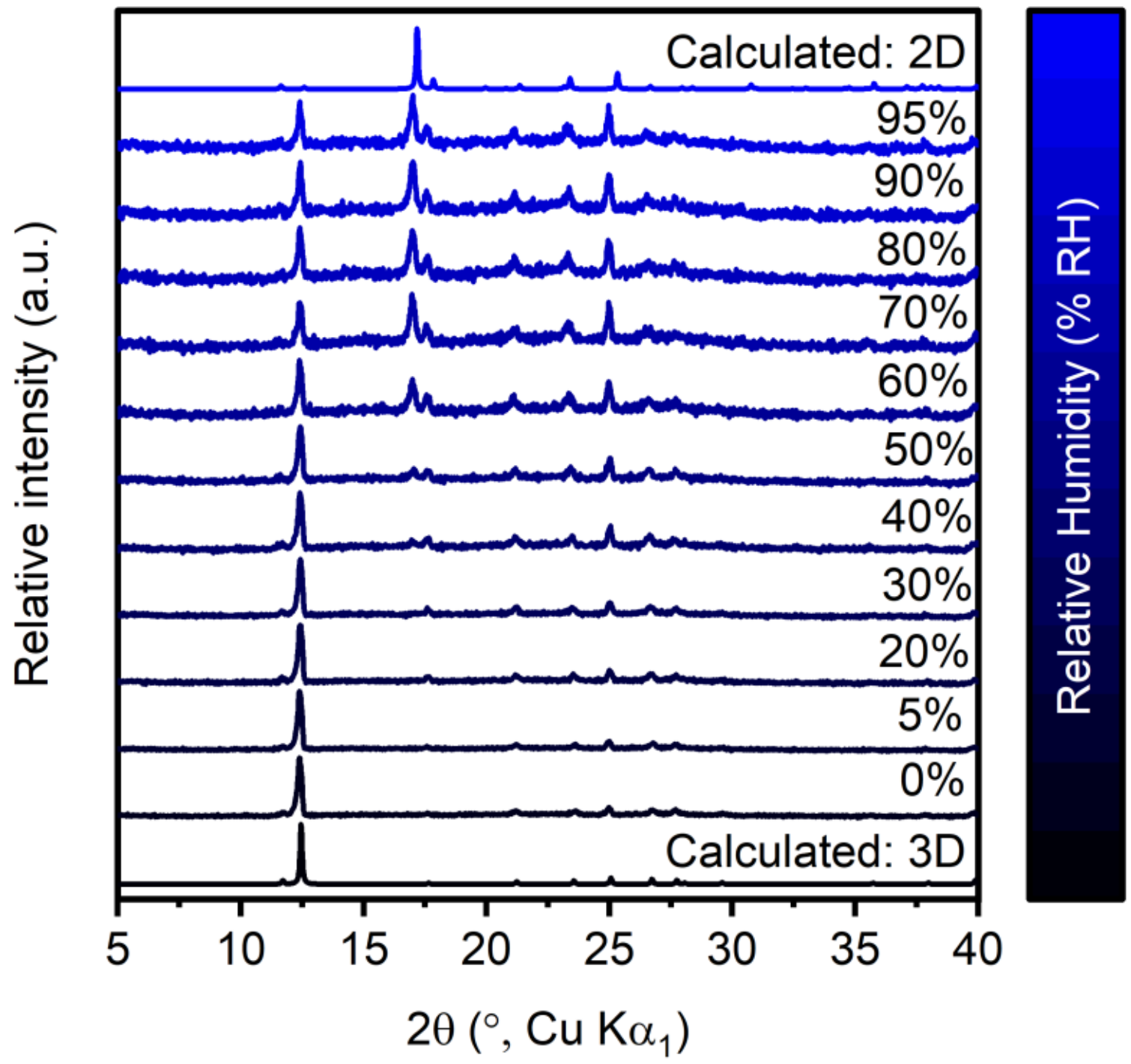

Figure S141 Variable-humidity PXRD patterns showing activated (3D) structure **pcu-SIFSIX-3-Co** phase converting to 2D **sql** phase (**sql-SIFSIX-3-Co**).

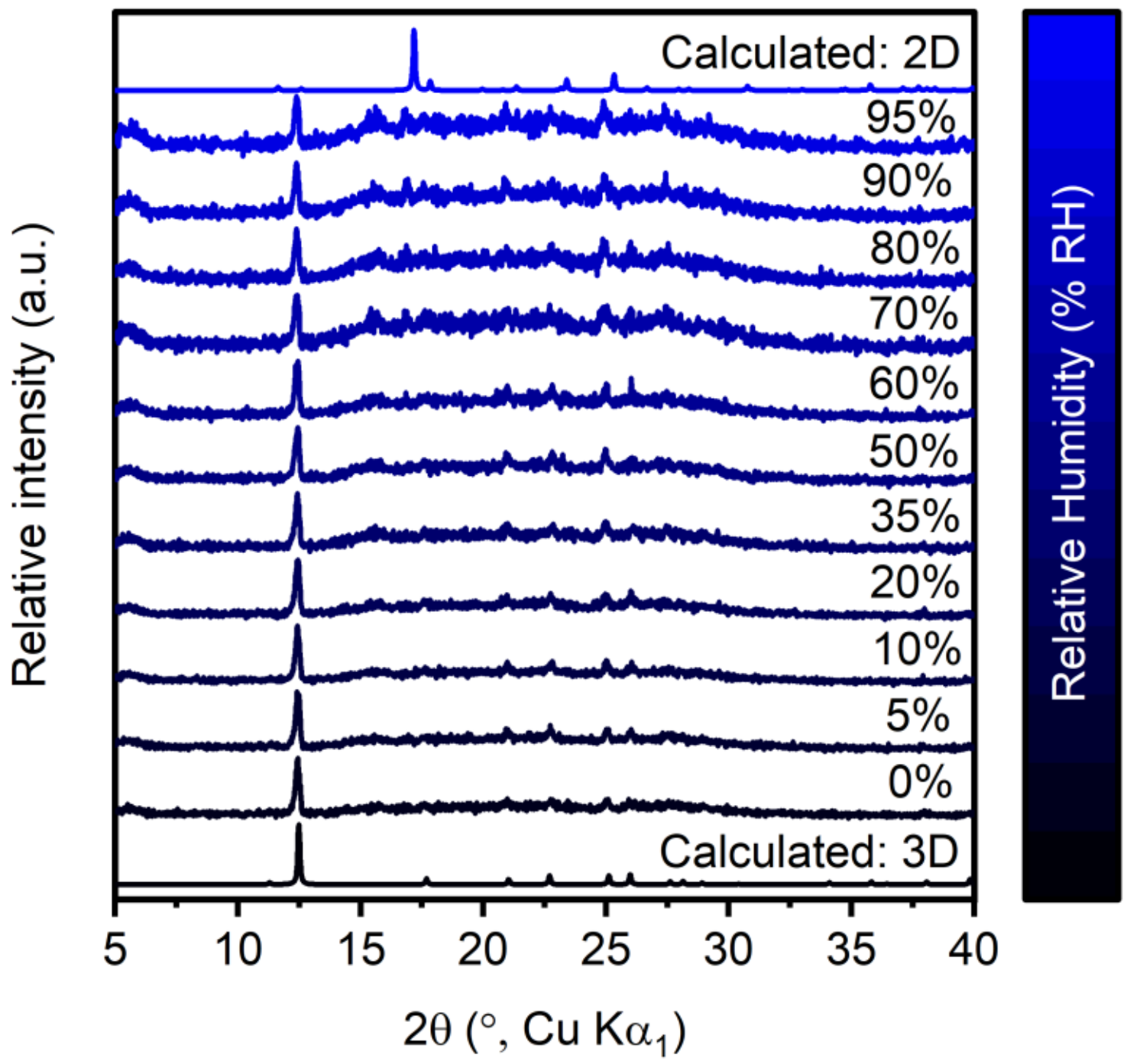

Figure S142 Variable-humidity PXRD patterns showing activated (3D) structure **pcu-TIFSIX-3-Co** phase (CSD refcode **SUHQUK**) converting to 2D **sql** phase (**sql-TIFSIX-3-Co**).

14 Powder X-ray diffraction after DVS measurement

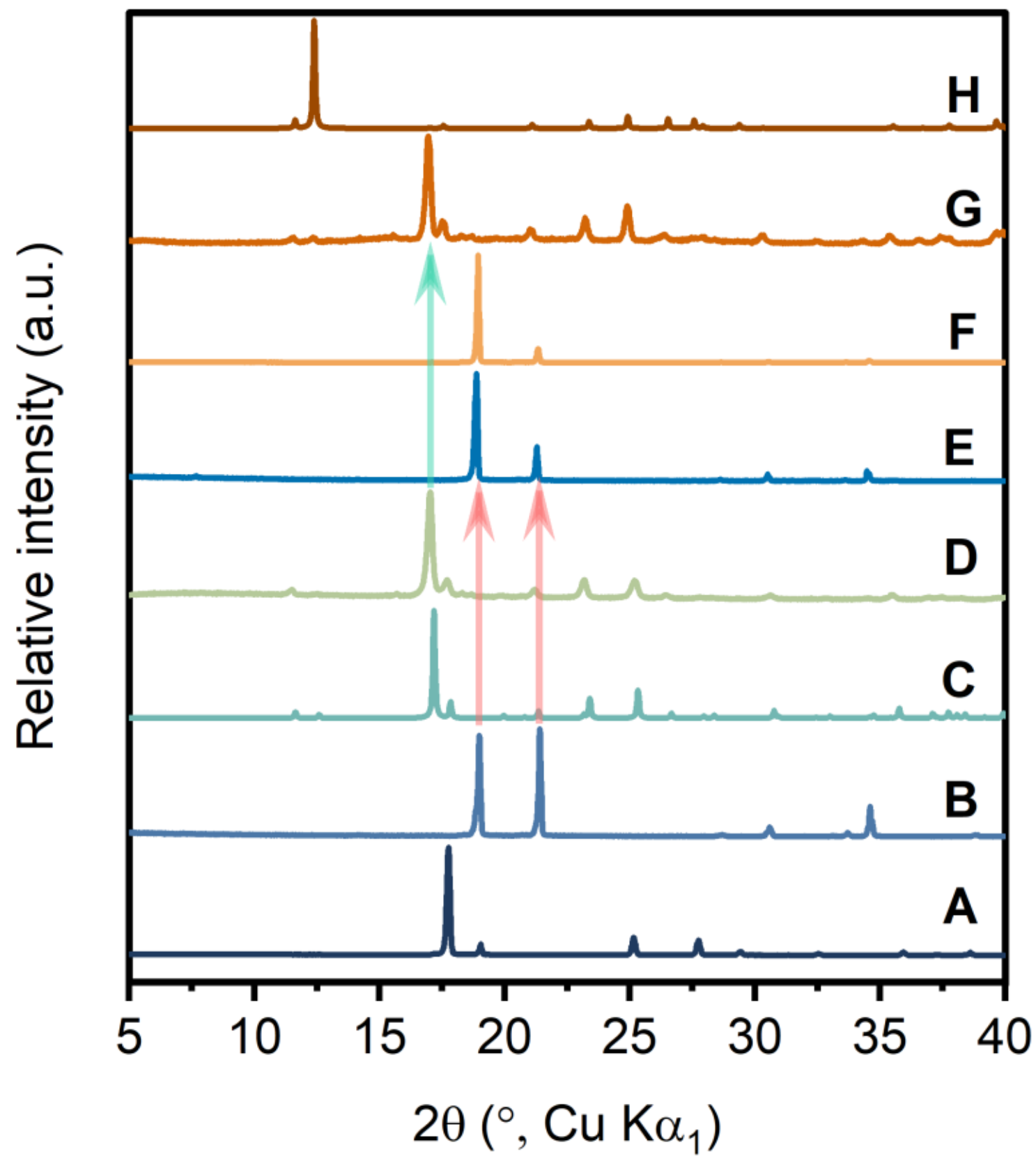

Figure S143 PXRD patterns of **SIFSIX-3-Zn**; all patterns are normalized for clarity. The bottom traces correspond to the starting materials, pyrazine (A, dark blue) and $ZnSiF_6{\cdot}6H_2O$ (B, steel blue). The calculated pattern of the 2D phase (C, teal) is shown for comparison, followed by the as-synthesized **sql-SIFSIX-3-Zn** (D, sage green), confirming formation of the intended 2D precursor. Patterns collected after H_2O sorption using DVS Vacuum (E), DVS Adventure (F), and BELSORP Max (G). The calculated pattern of the 3D phase **pcu-SIFSIX-3-Zn** (H, orange) (CSD refcode **FUDQIF**) is shown for comparison.

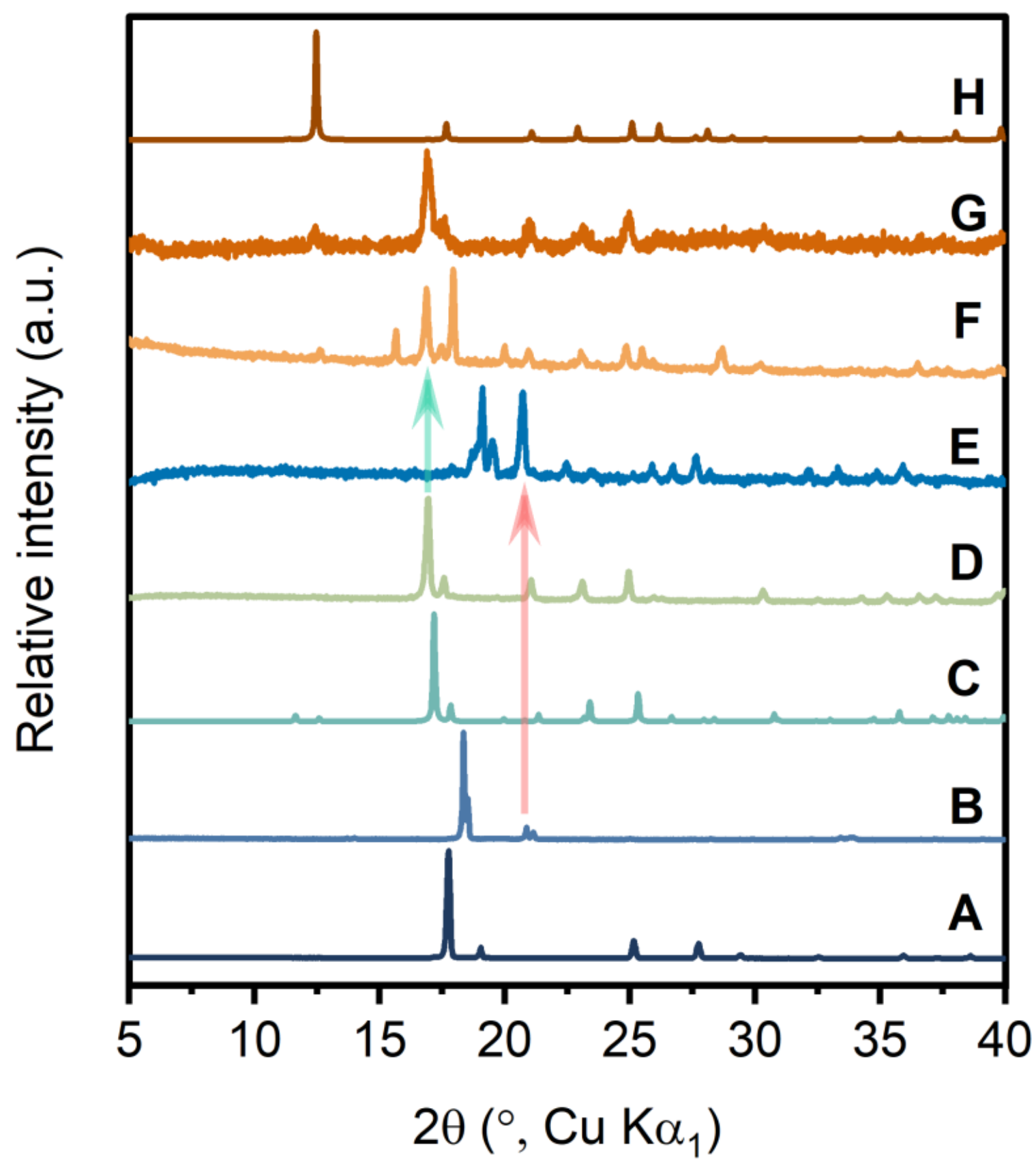

Figure S144 PXRD patterns of **GEFSIX-3-Co**; all patterns are normalized for clarity. The bottom traces correspond to the starting materials, pyrazine (A, dark blue) and $CoGeF_6{\cdot}6H_2O$ (B, steel blue). The calculated pattern of the 2D phase (C, teal) is shown for comparison, followed by the as-synthesized **sql-GEFSIX-3-Co** (D, sage green), confirming formation of the intended 2D precursor. Patterns collected after H_2O sorption using DVS Vacuum (E), DVS Adventure (F), and BELSORP Max (G). The calculated pattern of the 3D phase **pcu-GEFSIX-3-Co** (H, orange) (CSD refcode **HOYXIF**) is shown for comparison.

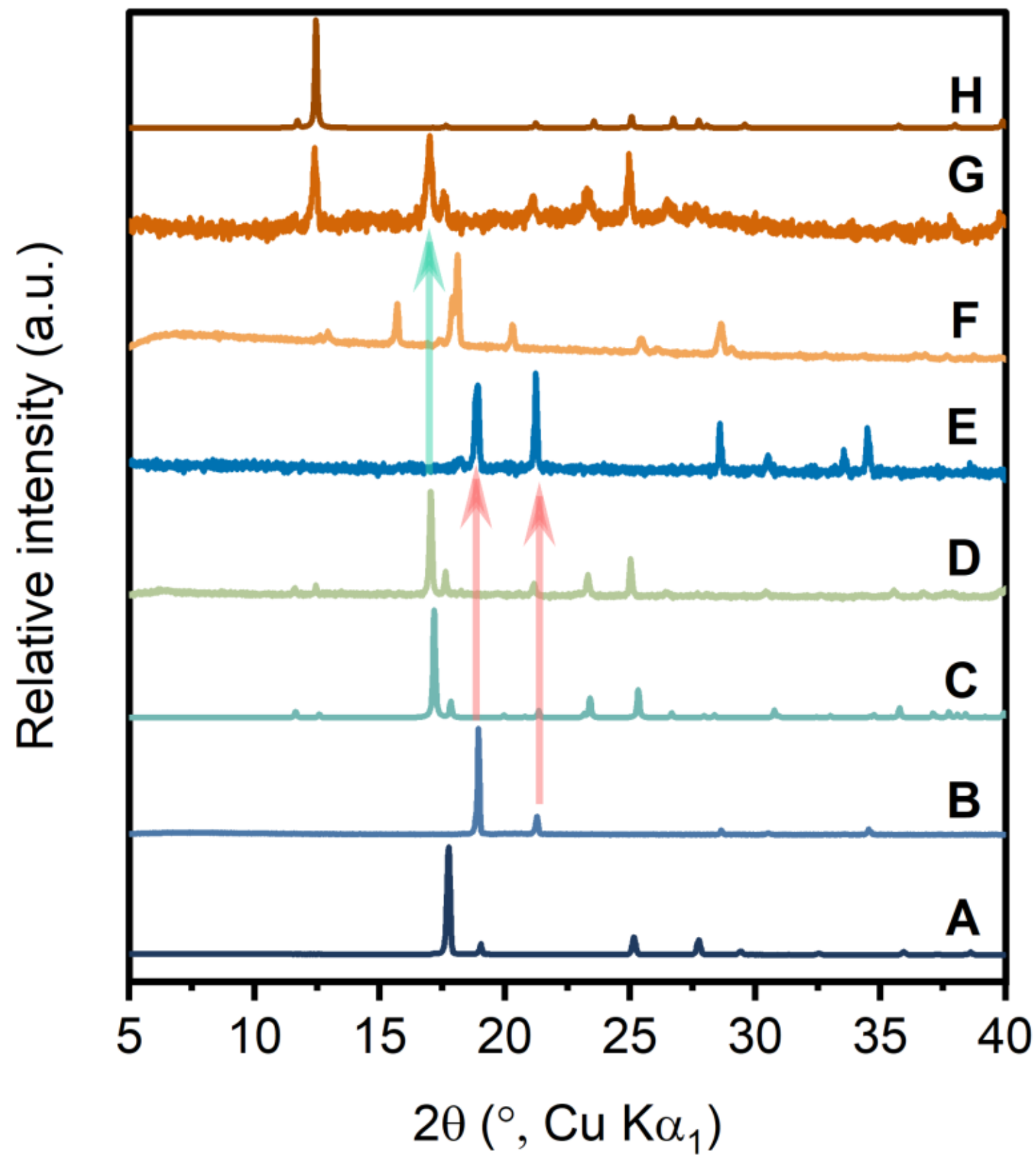

Figure S145 PXRD patterns of **SIFSIX-3-Co**; all patterns are normalized for clarity. The bottom traces correspond to the starting materials, pyrazine (A, dark blue) and $CoSiF_6{\cdot}6H_2O$ (B, steel blue). The calculated pattern of the 2D phase (C, teal) is shown for comparison, followed by the as-synthesized **sql-SIFSIX-3-Co** (D, sage green), confirming formation of the intended 2D precursor. Patterns collected after H_2O sorption using DVS Vacuum (E), DVS Adventure (F), and BELSORP Max (G). The calculated pattern of the 3D phase **pcu-SIFSIX-3-Co** (H, orange) (calculated from this work) is shown for comparison.

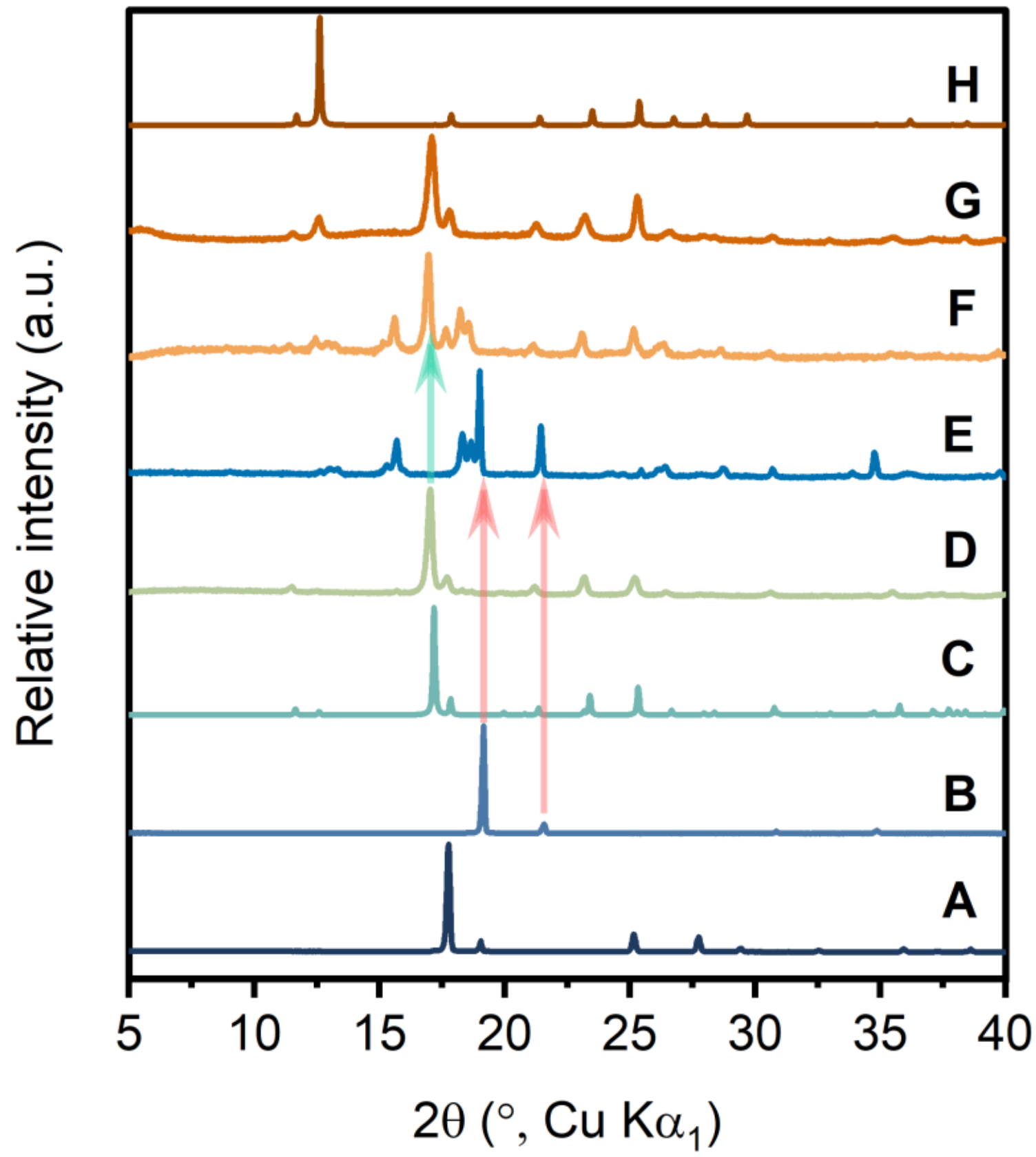

Figure S146 PXRD patterns of **SIFSIX-3-Ni**; all patterns are normalized for clarity. The bottom traces correspond to the starting materials, pyrazine (A, dark blue) and $NiSiF_6{\cdot}6H_2O$ (B, steel blue). The calculated pattern of the 2D phase (C, teal) is shown for comparison, followed by the as-synthesized **sql-SIFSIX-3-Ni** (CSD refcode **POCREI01**) (D, sage green), confirming formation of the intended 2D precursor. Patterns collected after H_2O sorption using DVS Vacuum (E), DVS Adventure (F), and BELSORP Max (G). The calculated pattern of the 3D phase **pcu-SIFSIX-3-Ni** (H, orange) (CSD refcode **DUMFEY**) is shown for comparison.

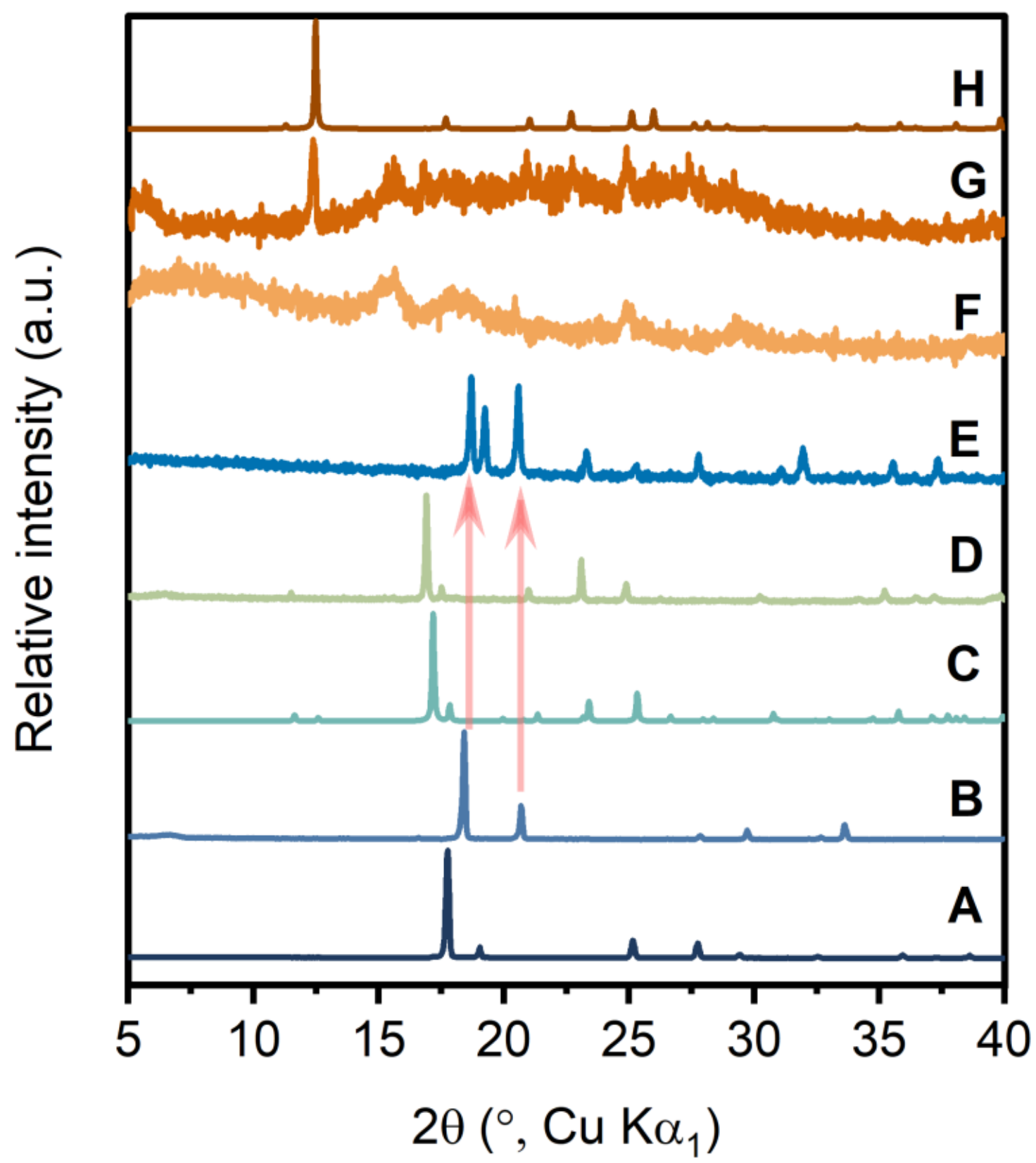

Figure S147 PXRD patterns of **TIFSIX-3-Co**; all patterns are normalized for clarity. The bottom traces correspond to the starting materials, pyrazine (A, dark blue) and $CoTiF_6{\cdot}6H_2O$ (B, steel blue). The calculated pattern of the 2D phase (C, teal) is shown for comparison, followed by the as-synthesized **sql-TIFSIX-3-Co** (D, sage green), confirming formation of the intended 2D precursor. Patterns collected after H_2O sorption using DVS Vacuum (E), DVS Adventure (F), and BELSORP Max (G). The calculated pattern of the 3D phase **pcu-TIFSIX-3-Co** (H, orange) (CSD refcode **SUHQUK**) is shown for comparison.

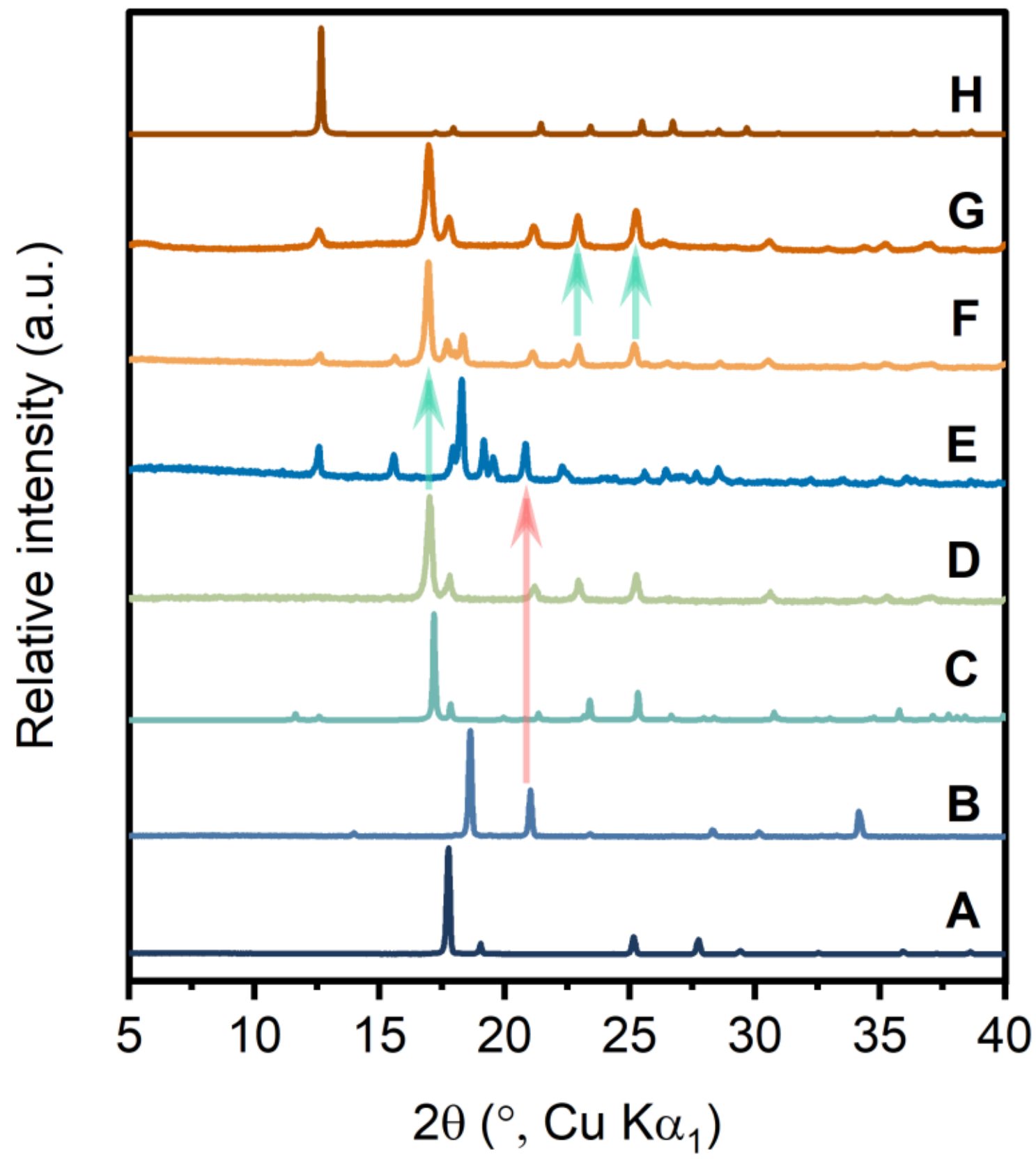

Figure S148 PXRD patterns of **GEFSIX-3-Ni**; all patterns are normalized for clarity. The bottom traces correspond to the starting materials, pyrazine (A, dark blue) and $NiGeF_6{\cdot}6H_2O$ (B, steel blue). The calculated pattern of the 2D phase (C, teal) is shown for comparison, followed by the as-synthesized **sql-GEFSIX-3-Ni** (D, sage green), confirming formation of the intended 2D precursor. Patterns collected after H_2O sorption using DVS Vacuum (E), DVS Adventure (F), and BELSORP Max (G). The calculated pattern of the 3D phase **pcu-GEFSIX-3-Ni** (H, orange) (CSD refcode **ZUTVAO**) is shown for comparison.

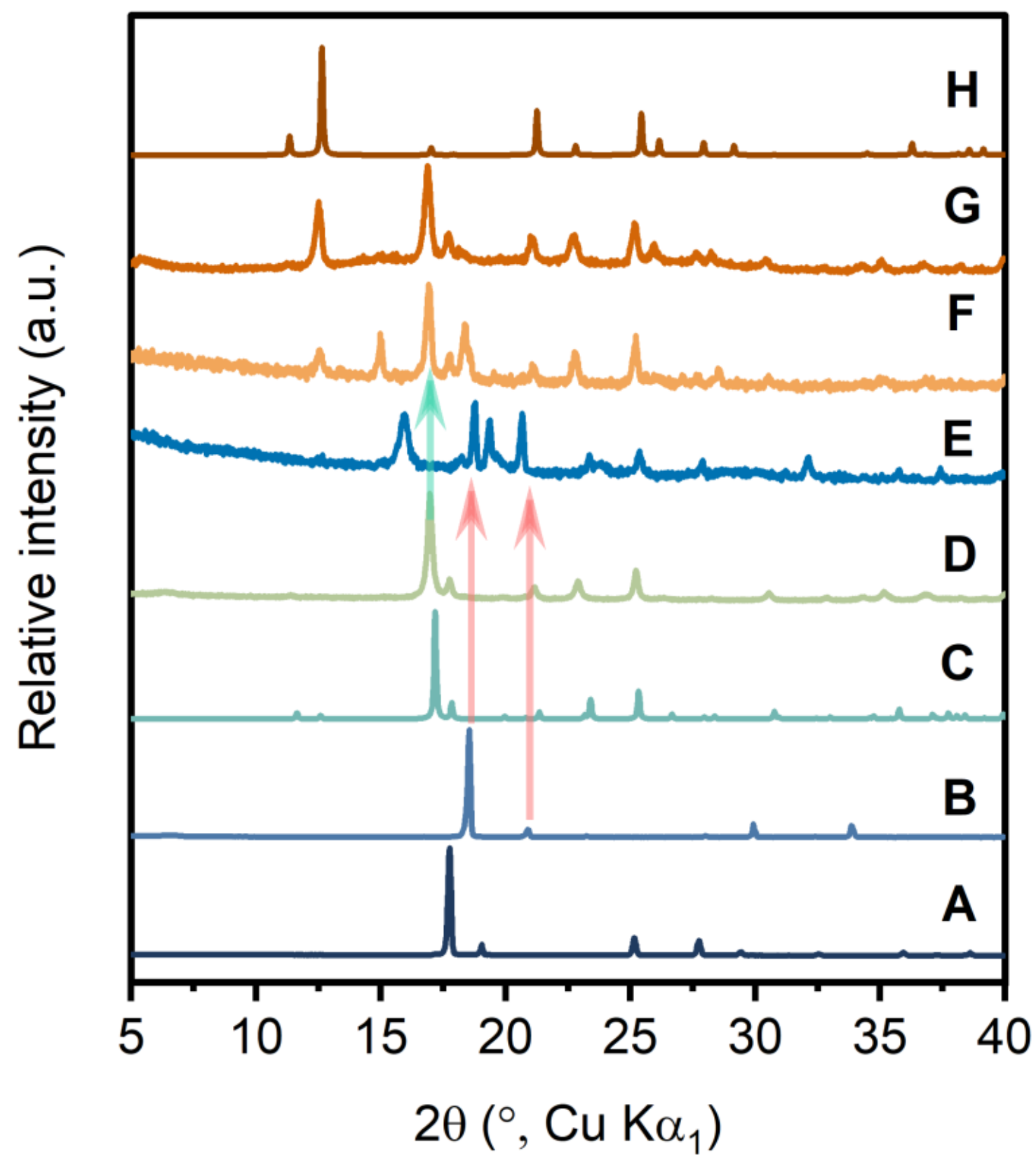

Figure S149 PXRD patterns of **TIFSIX-3-Ni**; all patterns are normalized for clarity. The bottom traces correspond to the starting materials, pyrazine (A, dark blue) and $NiTiF_6{\cdot}6H_2O$ (B, steel blue). The calculated pattern of the 2D phase (C, teal) is shown for comparison, followed by the as-synthesized **sql-TIFSIX-3-Ni** (D, sage green), confirming formation of the intended 2D precursor. Patterns collected after H_2O sorption using DVS Vacuum (E), DVS Adventure (F), and BELSORP Max (G). The calculated pattern of the 3D phase **pcu-TIFSIX-3-Ni** (H, orange) (CSD refcode **RIJGIE**) is shown for comparison.

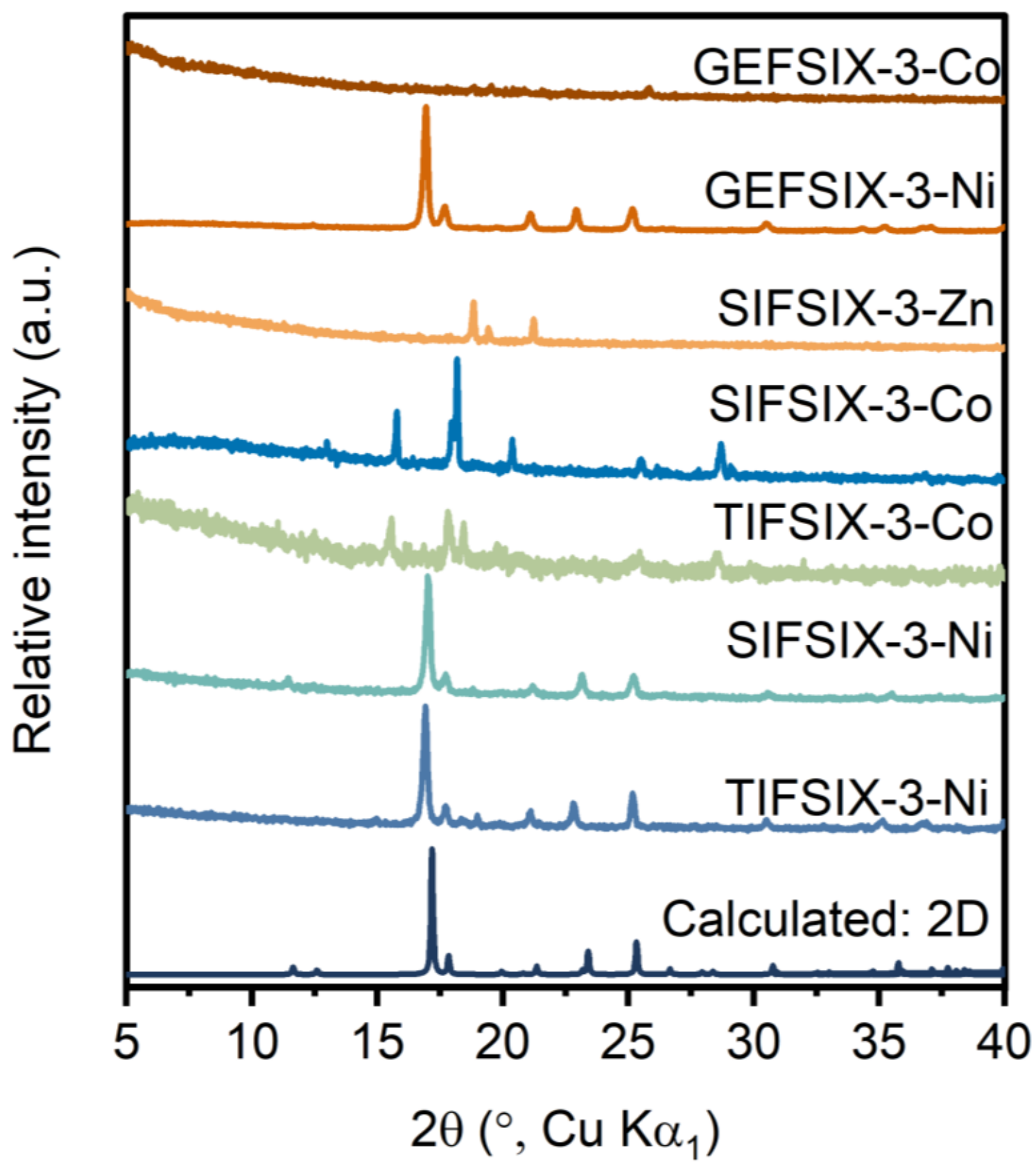

Figure S150 PXRD patterns of **M'FSIX-3-M** compounds after 6 days at 95% RH.

15 Estimation of Adsorption Enthalpy using Differential Scanning Calorimetry

The enthalpies associated with unloading were determined using temperature-ramp DSC analysis. It should be noted that these measurements allow for the approximation of the adsorption enthalpies.[26]

DSC thermograms were recorded using a TA instruments Q2000 analyzer equipped with a Refrigerated Cooling System (RCS90). During experiments, the sample compartment was purged using nitrogen gas (50 cm^3 min^{-1}). Samples of the loaded phases were prepared by keeping them at ambient conditions (50% RH, 22 °C). Each sample underwent two heating and cooling cycles using the following sequence: the sample was cooled from RT to -10 (**M'FSIX-25-M**) or 10 °C (**M'FSIX-3-M**) and kept isothermal for 3 min, followed by heating at a ramp rate of 1 °C min^{-1} and finally cooled to 2 °C at 1 °C min^{-1}. This temperature range was chosen to ensure that sufficient baseline could be achieved before the onset and after the completion of dehydration. In all cases, the sample was placed in an open aluminum pan to allow for the loss of water vapor during heating.

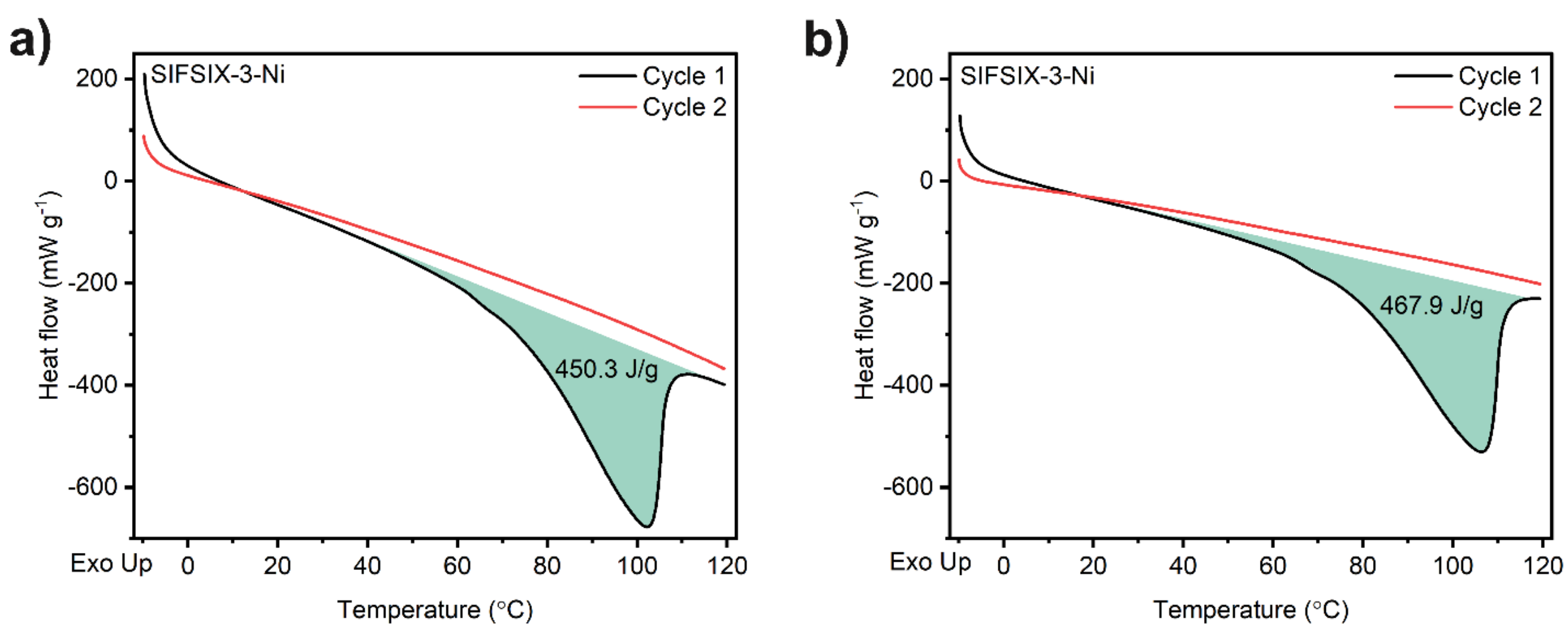

Figure S151 DSC thermograms of the heating segments for **SIFSIX-3-Ni** over two cycles (Cycle 1, black line; Cycle 2, red line): Figure a) and b) are repetition of same sample. The values of Δ*h* was determined by integration of the shaded regions (with respect to time).

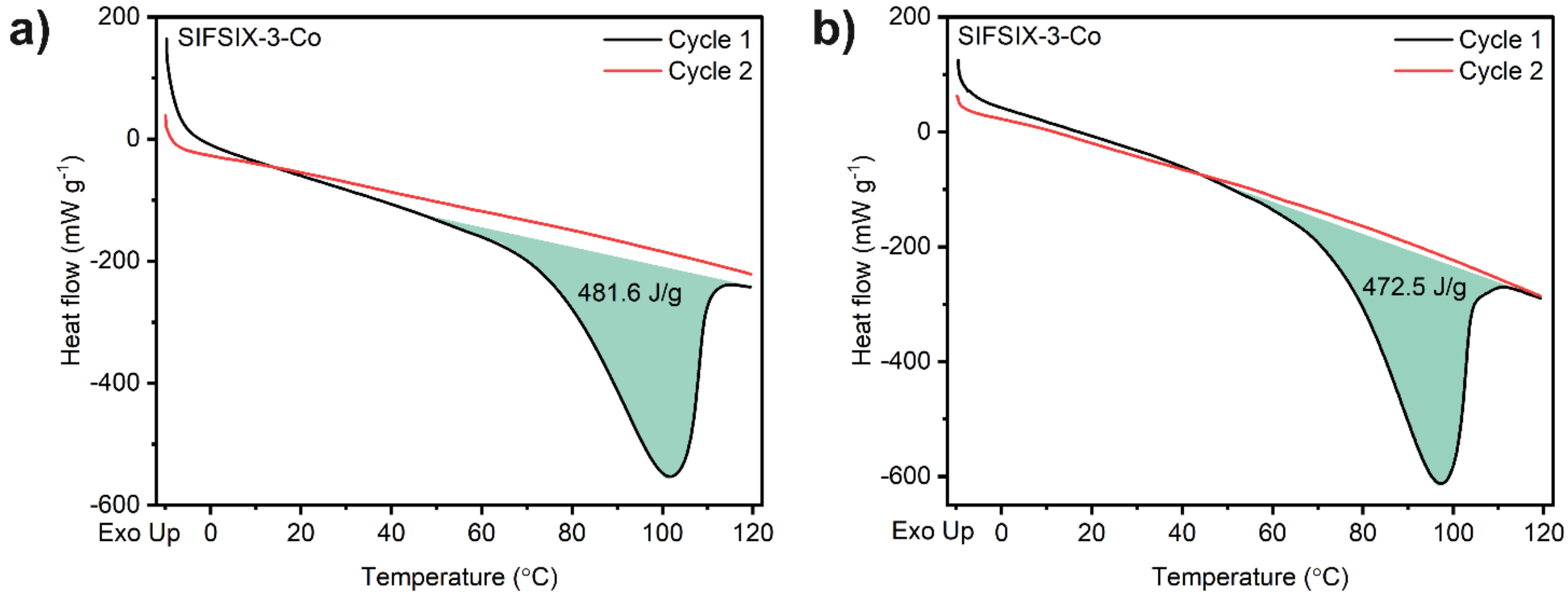

Figure S152 DSC thermograms of the heating segments for **SIFSIX-3-Co** over two cycles (Cycle 1, black line; Cycle 2, red line) : Figure a) and b) are repetition of same sample. The values of Δ*h* was determined by integration of the shaded regions (with respect to time).

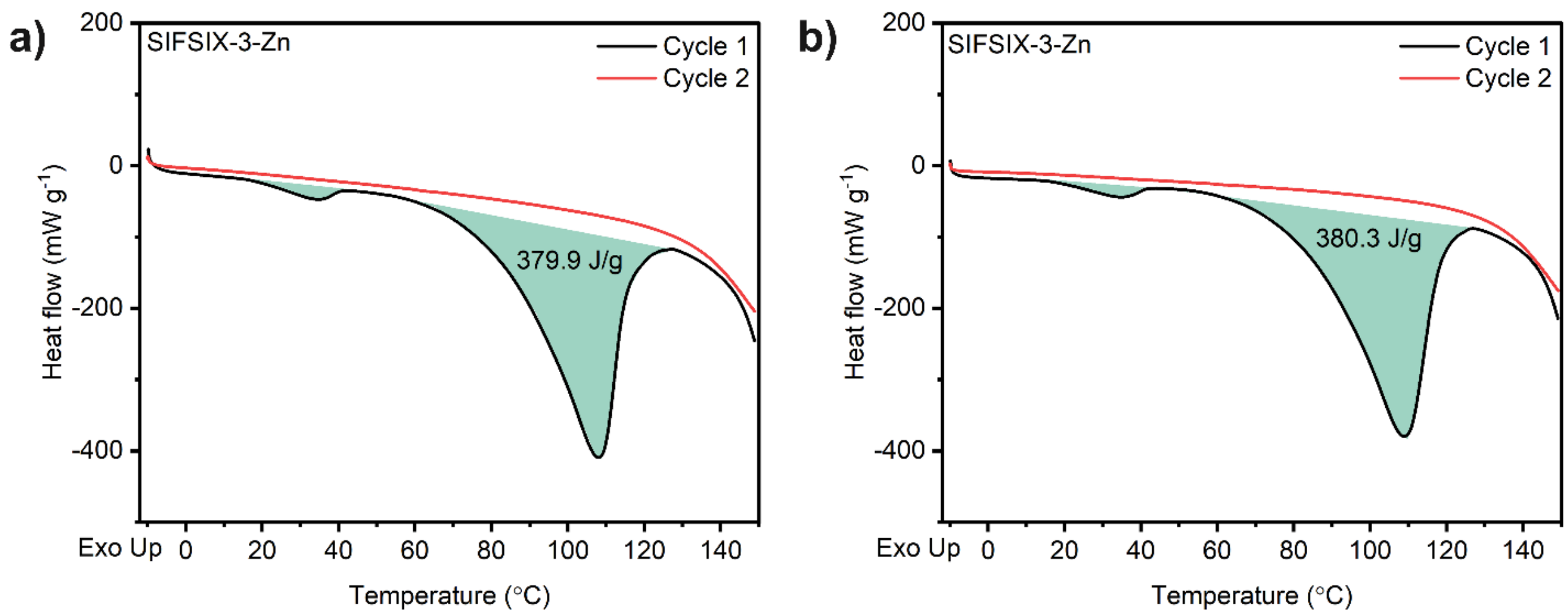

Figure S153 DSC thermograms of the heating segments for **SIFSIX-3-Zn** over two cycles (Cycle 1, black line; Cycle 2, red line). Figure a) and b) are repetition of same sample. The values of Δh was determined by integration of the shaded regions (with respect to time).

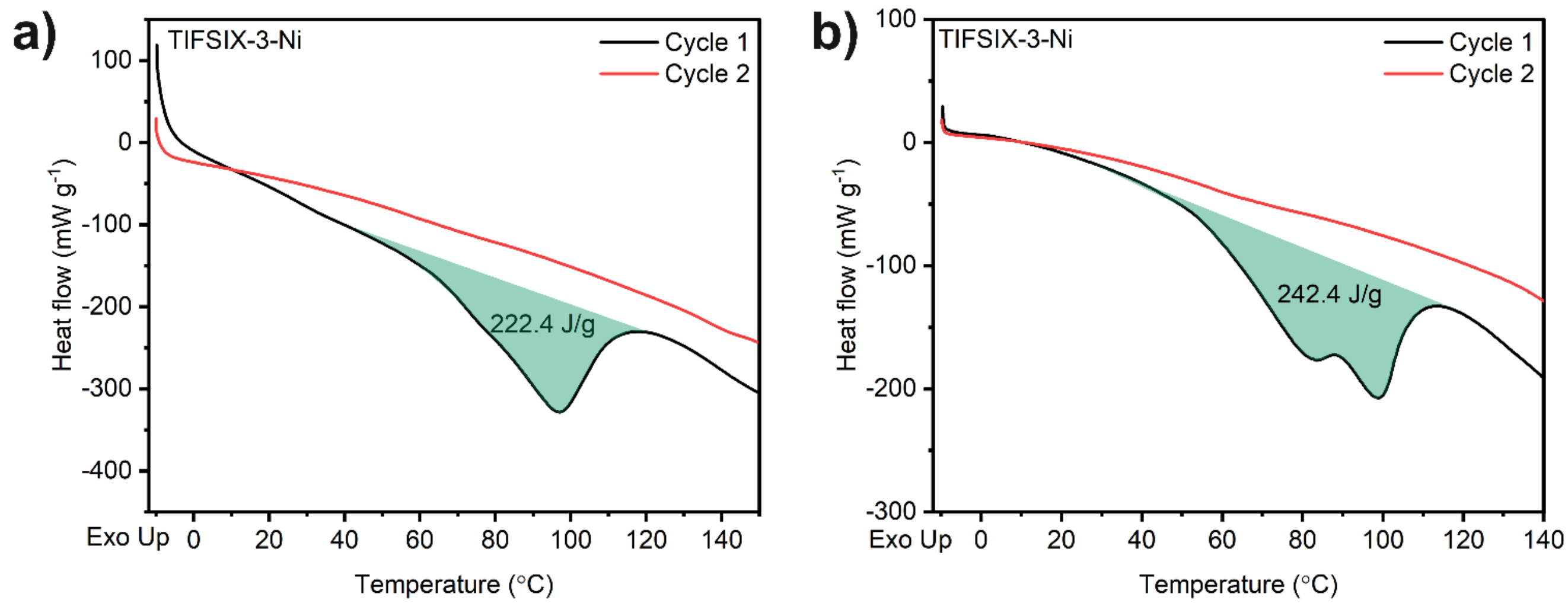

Figure S154 DSC thermograms of the heating segments for **TIFSIX-3-Ni** over two cycles (Cycle 1, black line; Cycle 2, red line): Figure a) and b) are repetition of same sample. The values of Δ*h* was determined by integration of the shaded regions (with respect to time).

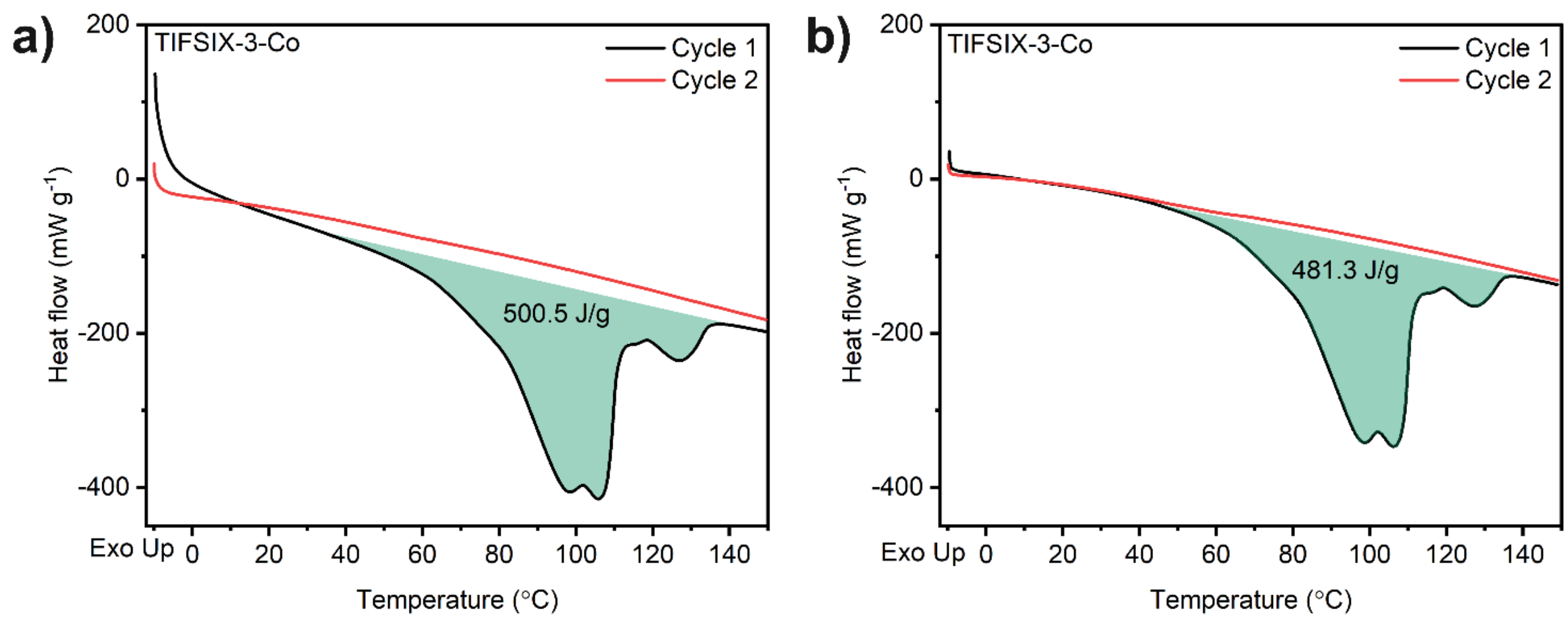

Figure S155 DSC thermograms of the heating segments for **TIFSIX-3-Co** over two cycles (Cycle 1, black line; Cycle 2, red line): Figure a) and b) are repetition of same sample. The values of Δ*h* was determined by integration of the shaded regions (with respect to time).

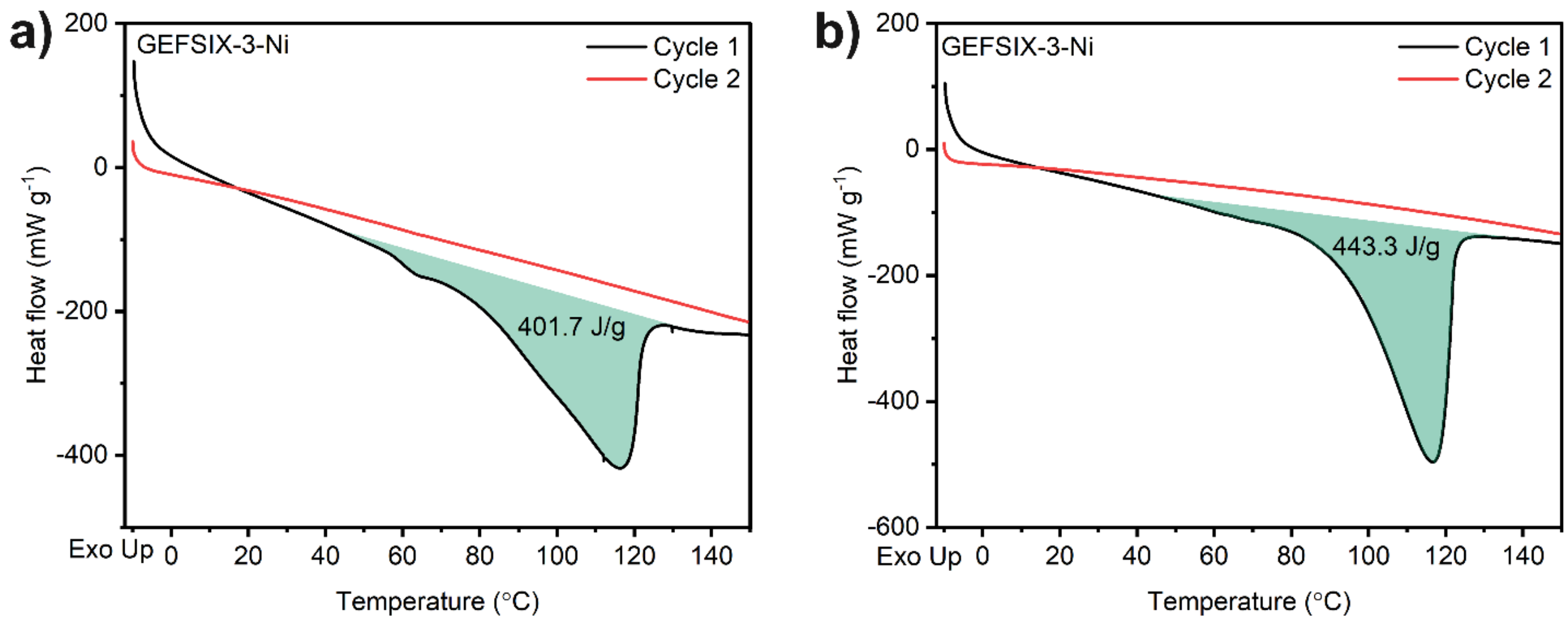

Figure S156 DSC thermograms of the heating segments for **GEFSIX-3-Ni** over two cycles (Cycle 1, black line; Cycle 2, red line): Figure a) and b) are repetition of same sample. The values of Δ*h* was determined by integration of the shaded regions (with respect to time).

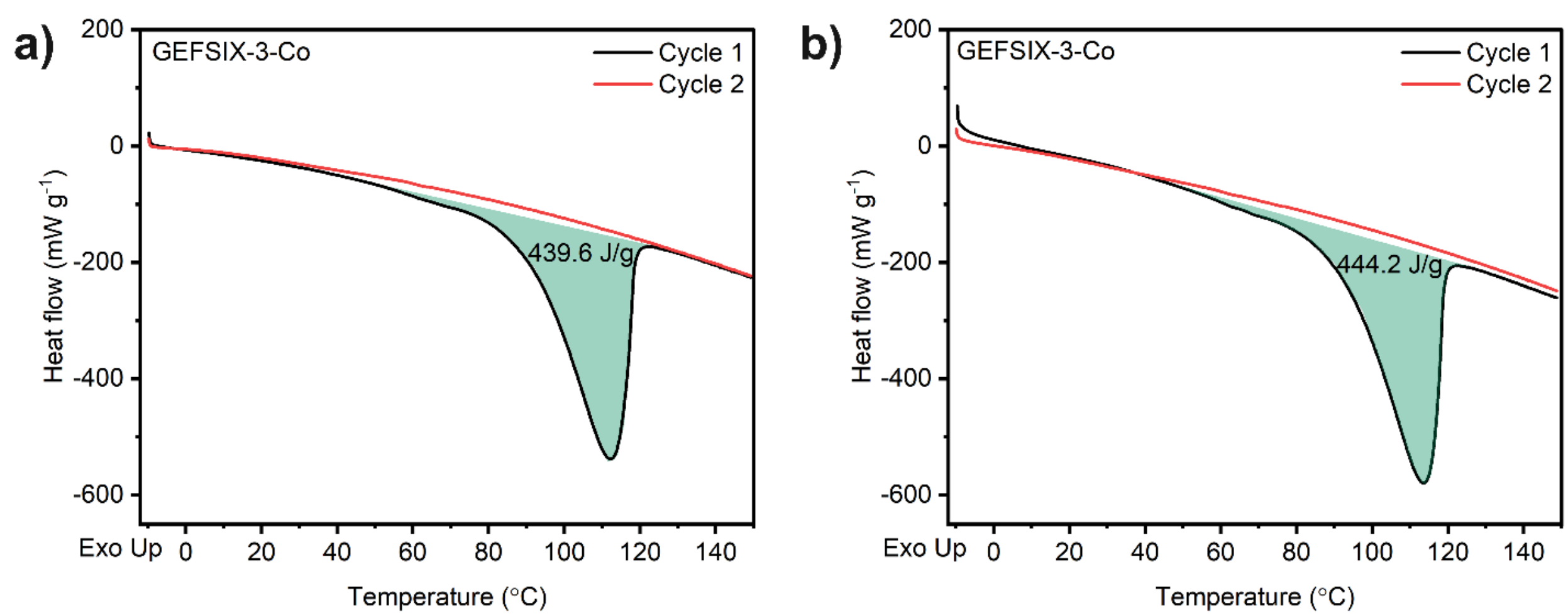

Figure S157 DSC thermograms of the heating segments for **GEFSIX-3-Co** over two cycles (Cycle 1, black line; Cycle 2, red line): Figure a) and b) are repetition of same sample. The values of Δ*h* was determined by integration of the shaded regions (with respect to time).

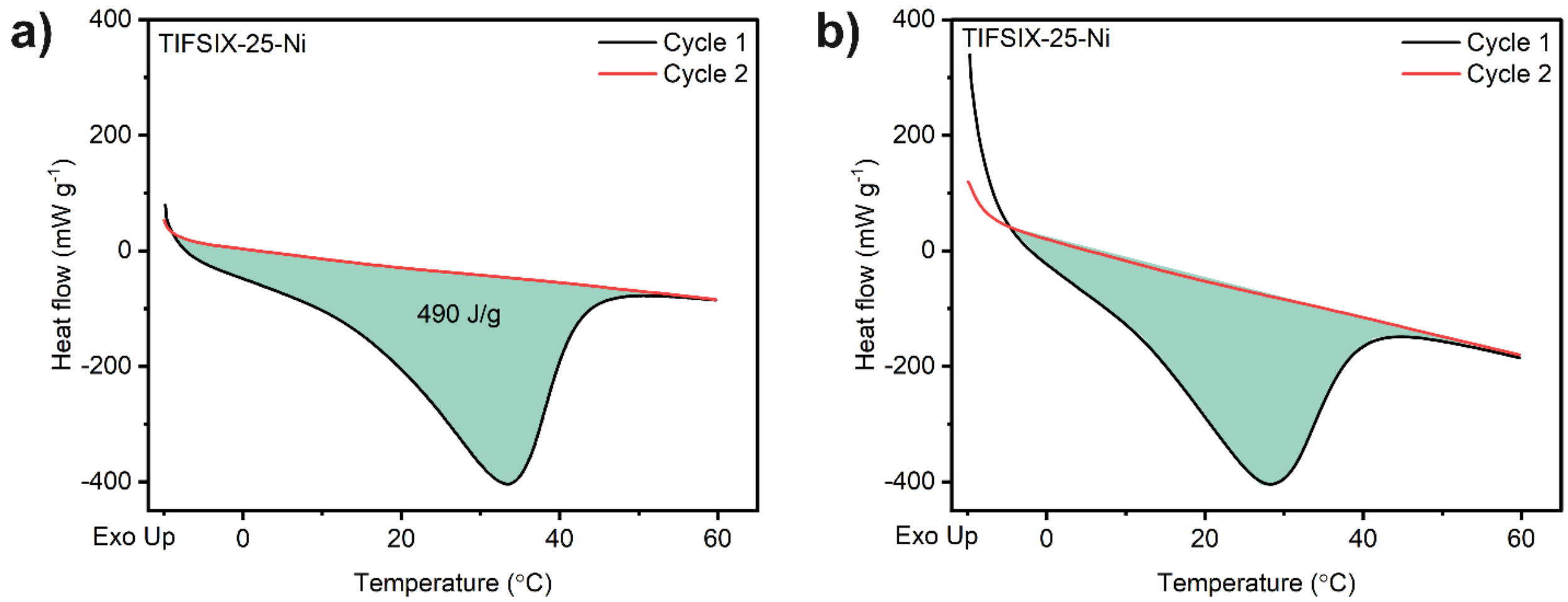

Figure S158 DSC thermograms of the heating segments for **TIFSIX-25-Ni** over two cycles (Cycle 1, black line; Cycle 2, red line). The values of Δ*h* was determined by integration of the shaded regions (with respect to time).

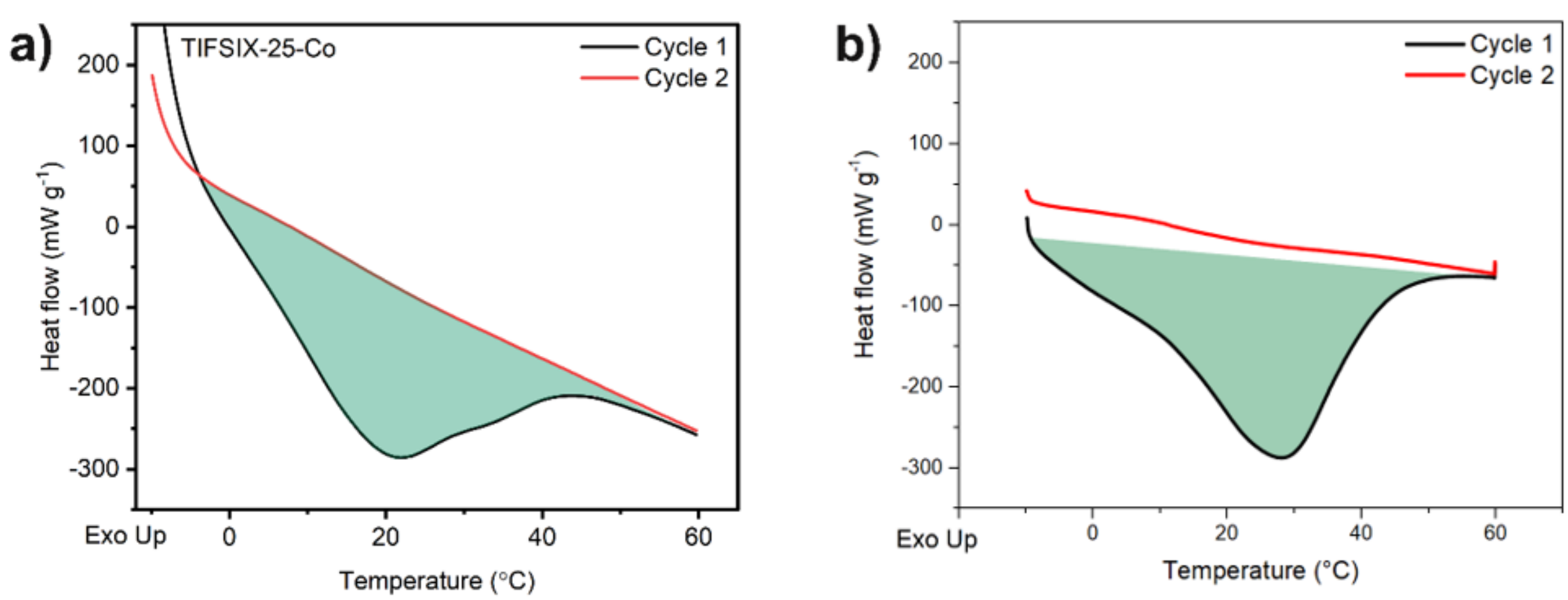

Figure S159 DSC thermograms of the heating segments for **TIFSIX-25-Co** over two cycles (Cycle 1, black line; Cycle 2, red line). The values of Δ*h* was determined by integration of the shaded regions (with respect to time).

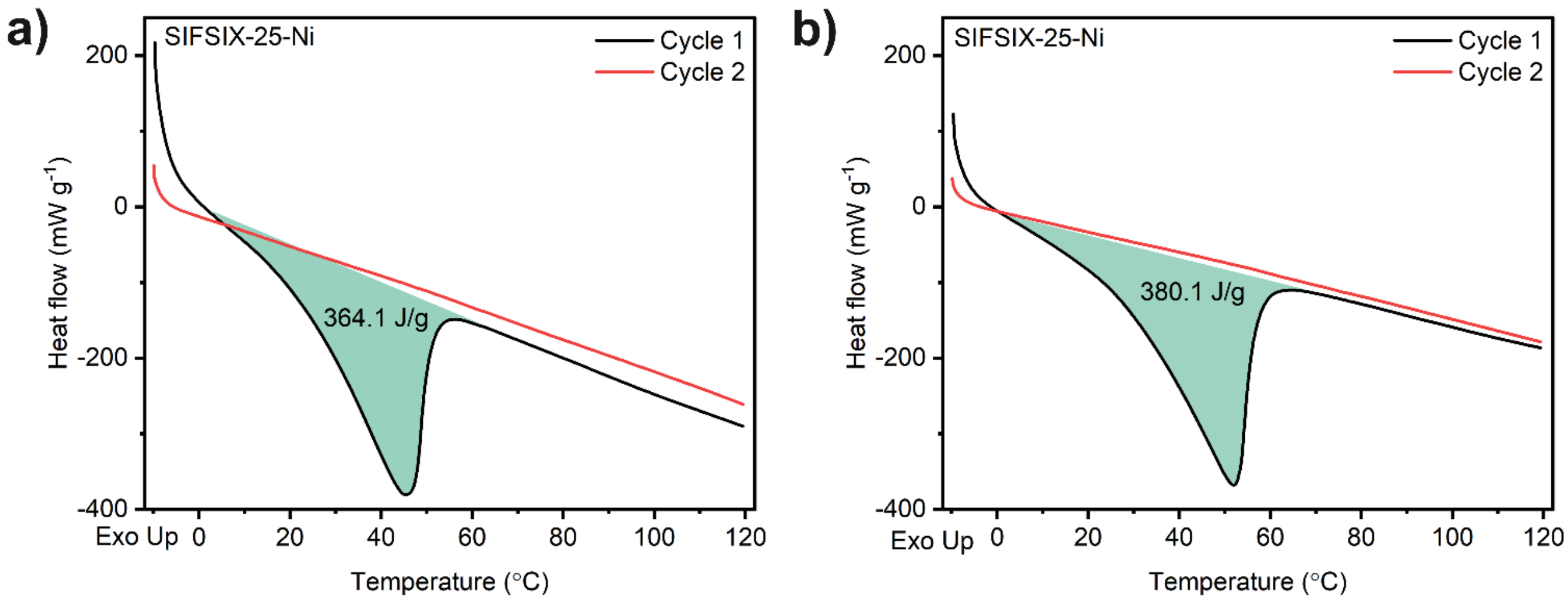

Figure S160 DSC thermograms of the heating segments for **SIFSIX-25-Ni** over two cycles (Cycle 1, black line; Cycle 2, red line): Figure a) and b) are repetition of same sample. The values of Δ*h* was determined by integration of the shaded regions (with respect to time).

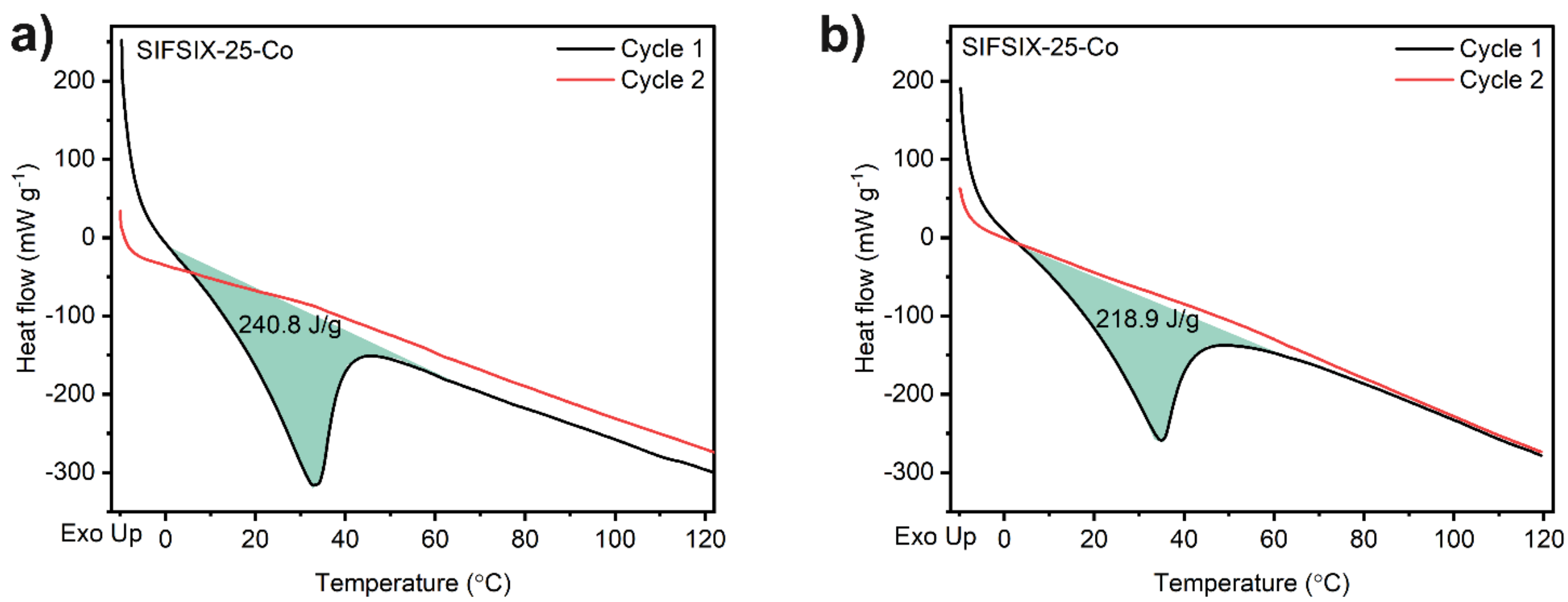

Figure S161 DSC thermograms of the heating segments for **SIFSIX-25-Co** over two cycles (Cycle 1, black line; Cycle 2, red line): Figure a) and b) are repetition of same sample. The values of Δ*h* was determined by integration of the shaded regions (with respect to time).

Table S9 Enthalpies of unloading (kJ mol^{-1}) determined from DSC analysis. The mean enthalpy was determined from two separate measurements.

Sample	Enthalpy 1	Enthalpy 2	Mean (μ)	SD (σ)	SEM
**SIFSIX-3-Ni**	42	44	43	1.4	1.0
**SIFSIX-3-Co**	51	50	51	0.7	0.5
**SIFSIX-3-Zn**	40	40	40	0.1	0.1
**TIFSIX-3-Ni**	25	27	26	1.6	1.1
**TIFSIX-3-Co**	53	51	52	1.4	1.0
**GEFSIX-3-Ni**	40	44	42	3.0	2.1
**GEFSIX-3-Co**	44	44	44	0.3	0.2
**SIFSIX-25-Ni**	37	39	38	1.2	0.8
**SIFSIX-25-Co**	24	22	23	1.5	1.1
**TIFSIX-25-Ni**	49	52	51	1.9	1.3
**TIFSIX-25-Co**	43	41	42	1.4	1.0

SEM = standard error of the mean

16 Humidity-swing kinetics

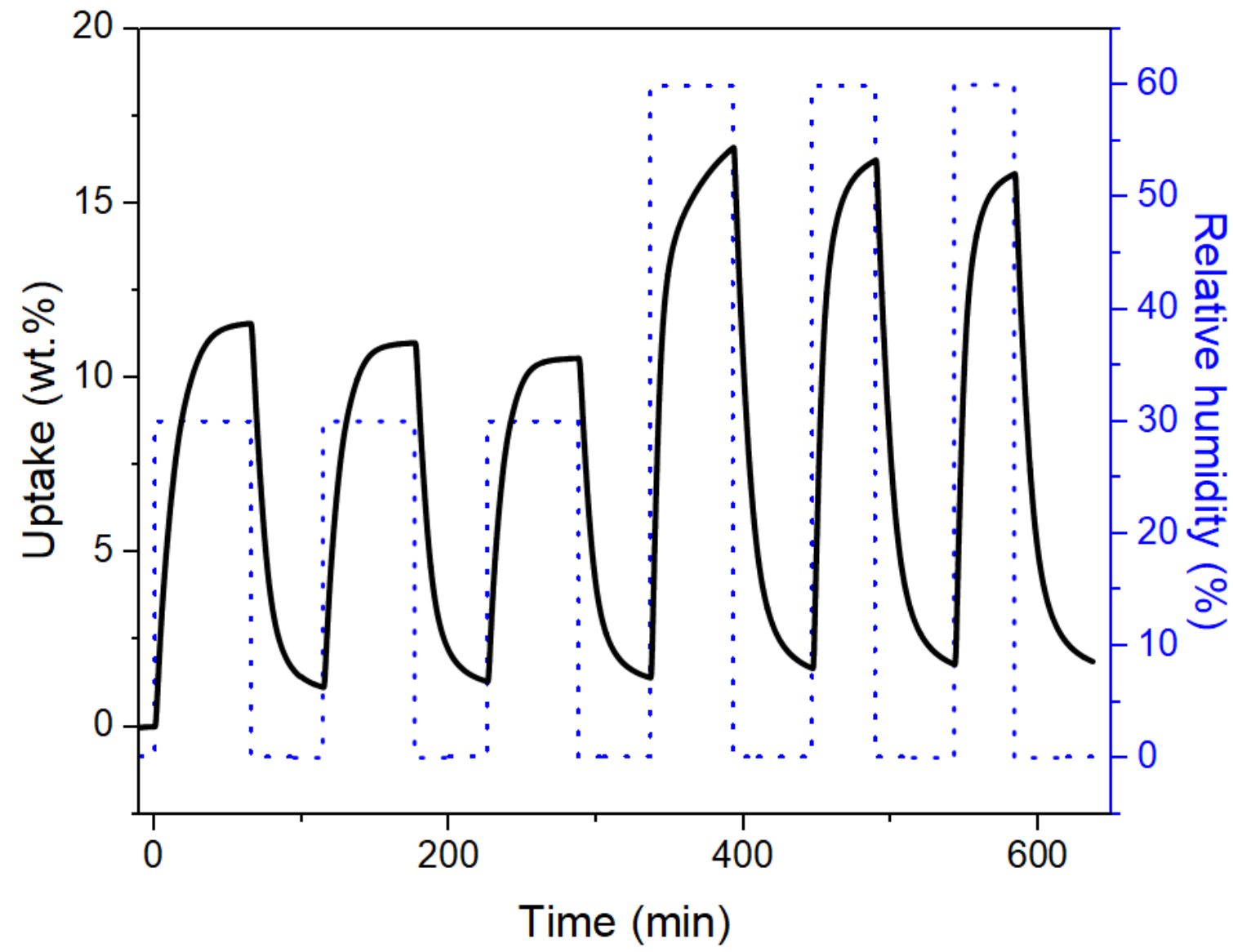

Figure S162 Six water vapor sorption cycles carried out on **TIFSIX-25-Co** using an Adventure DVS instrument at 25 °C: 3 consecutive 0–30% RH swing experiments followed by 3 consecutive 0–60% RH swing experiments.

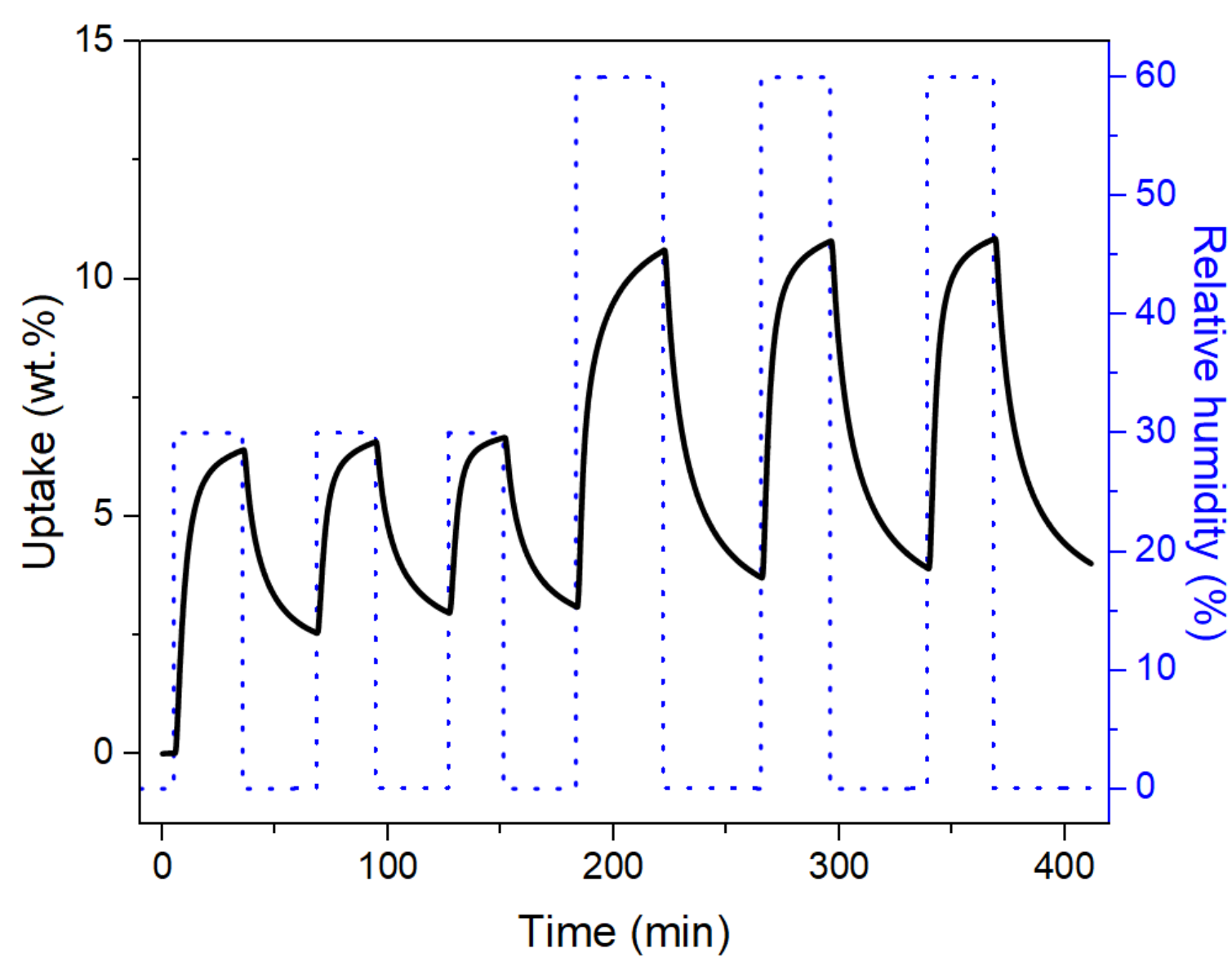

Figure S163 Six water vapor sorption cycles carried out on **SIFSIX-25-Ni** using an Adventure DVS instrument at 25 °C: 3 consecutive 0–30% RH swing experiments followed by 3 consecutive 0–60% RH swing experiments.

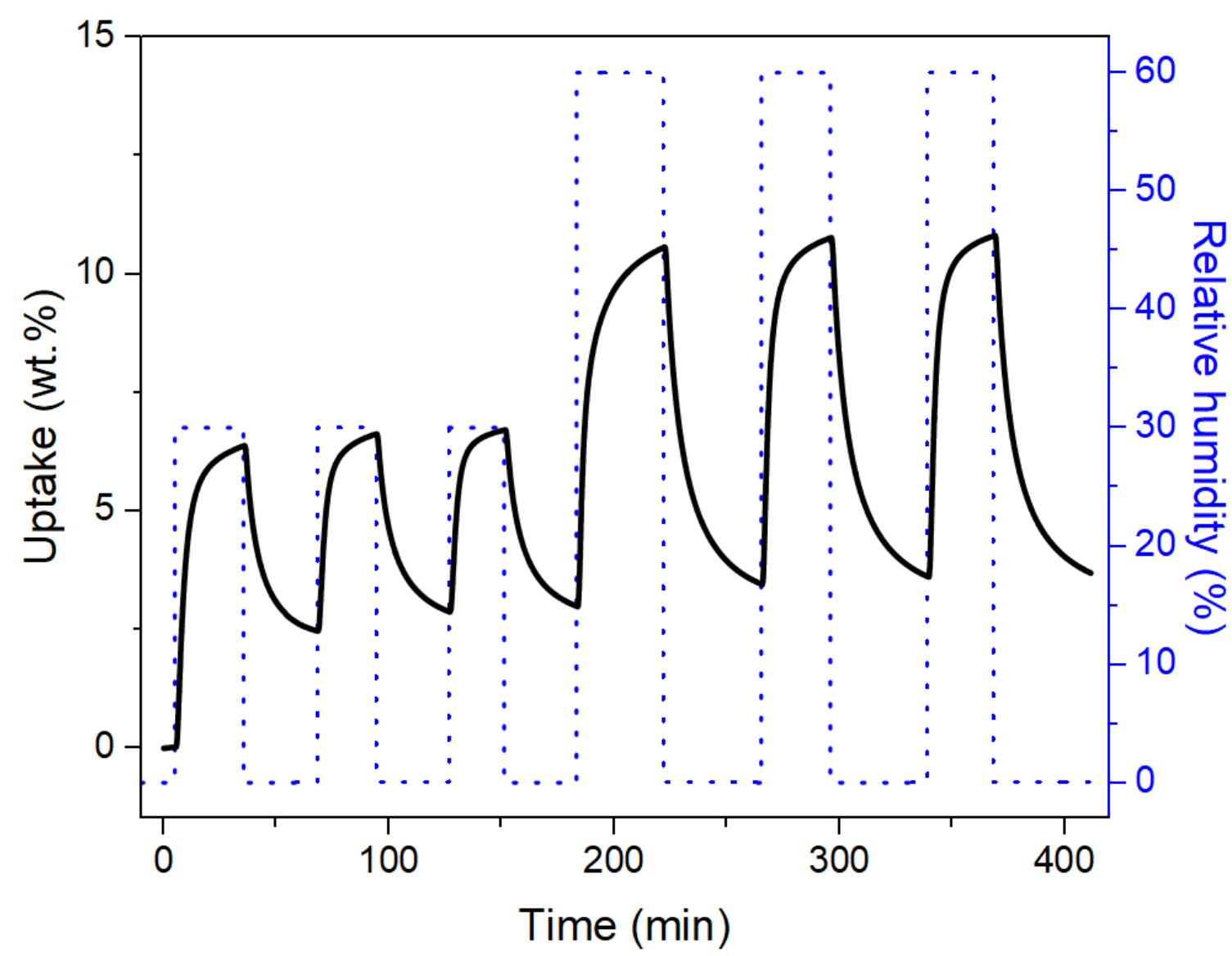

Figure S164 Five water vapor sorption cycles carried out on **SIFSIX-25-Co** using an Adventure DVS instrument at 25 °C: 2 consecutive 0–30% RH swing experiments followed by 3 consecutive 0–60% RH swing experiments.

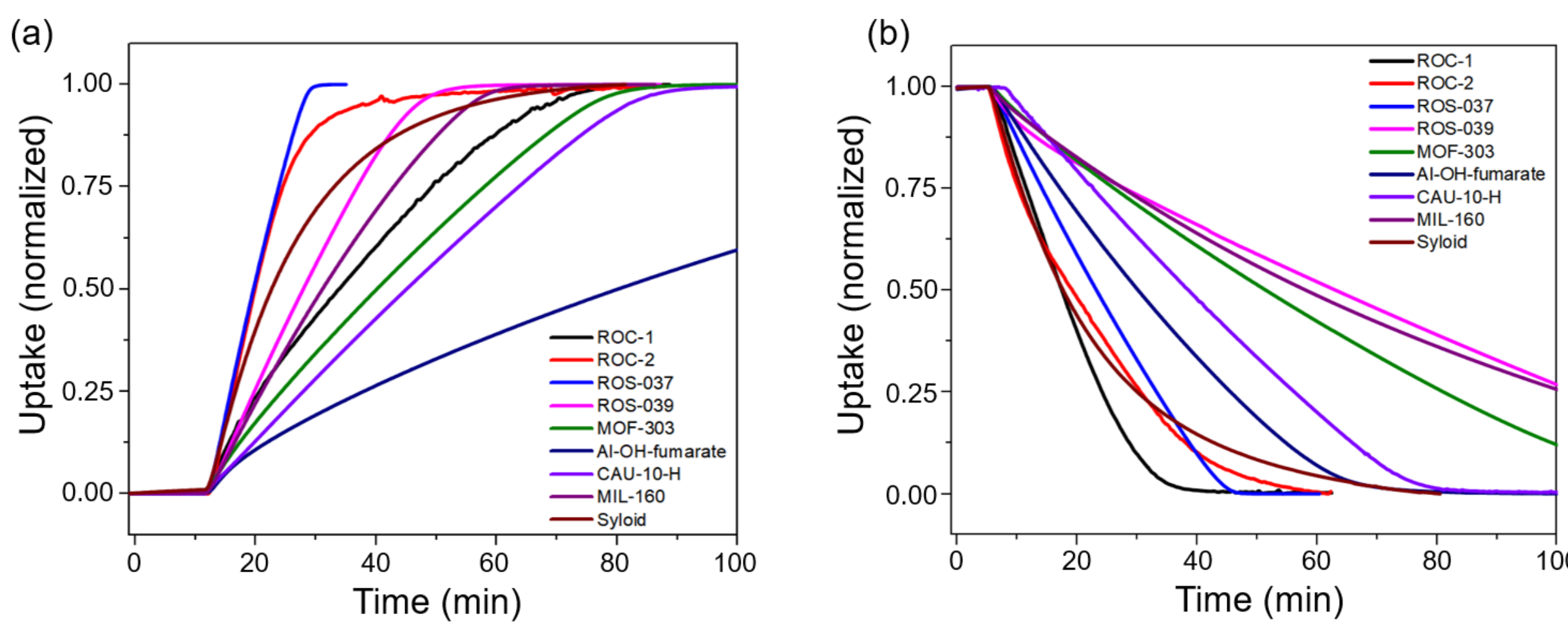

Figure S165 Water vapor RH-swing (0–30% RH) (a) adsorption and (b) desorption, and T-swing (0–30% RH) (c) adsorption and (d) desorption kinetic plots measured for **TIFSIX-25-Ni (ROC-1)**, **TIFSIX-25-Co (ROC-2)** and benchmark sorbents[27] using a DVS Adventure instrument at RT.

17 Temperature-humidity-swing kinetics

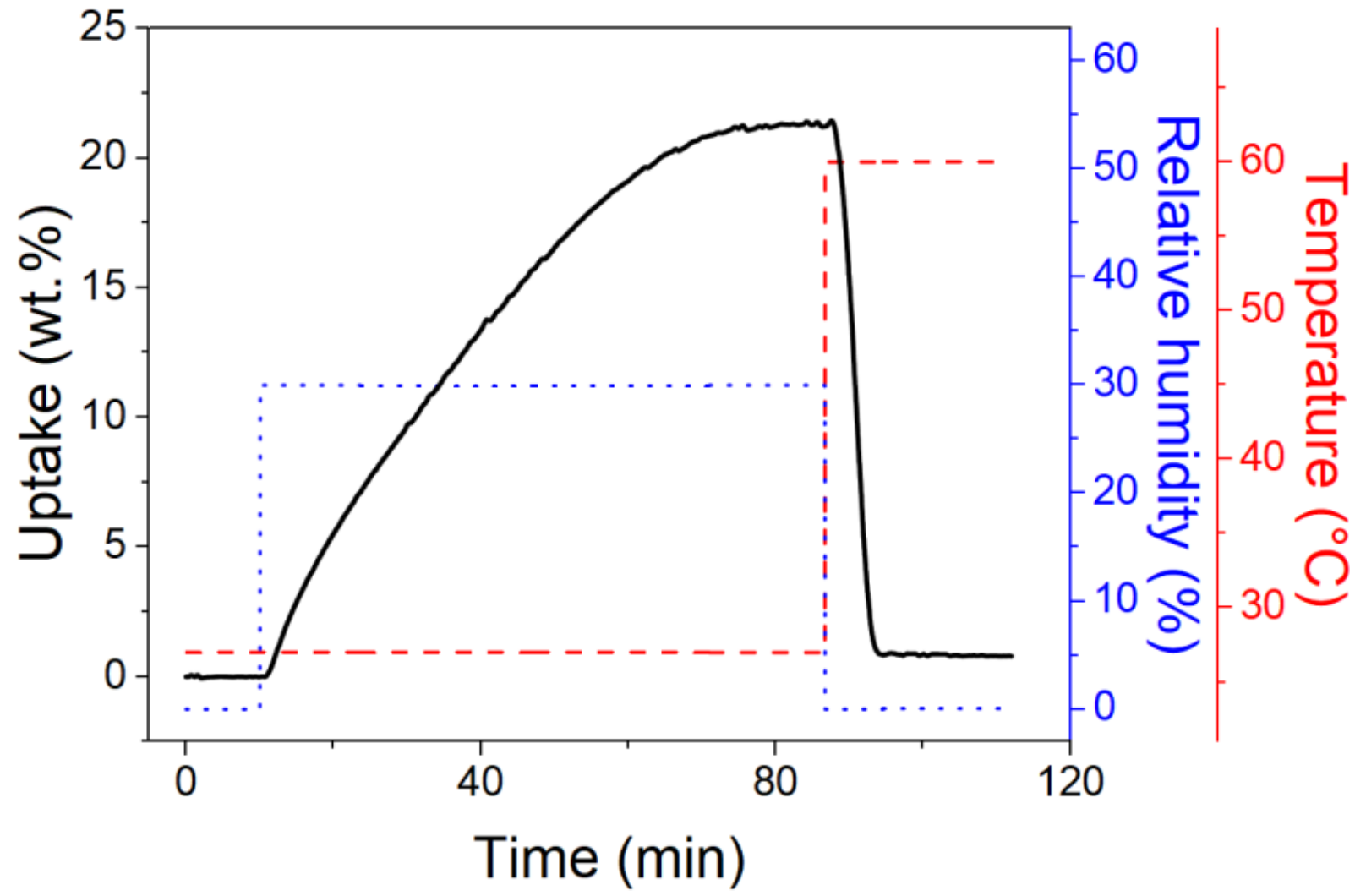

Figure S166 Water vapor sorption T-RH-swing kinetics measurements carried out on **TIFSIX-25-Ni**. Adsorption conditions: 30% RH, 27 °C; desorption conditions: 0% RH, 60 °C.

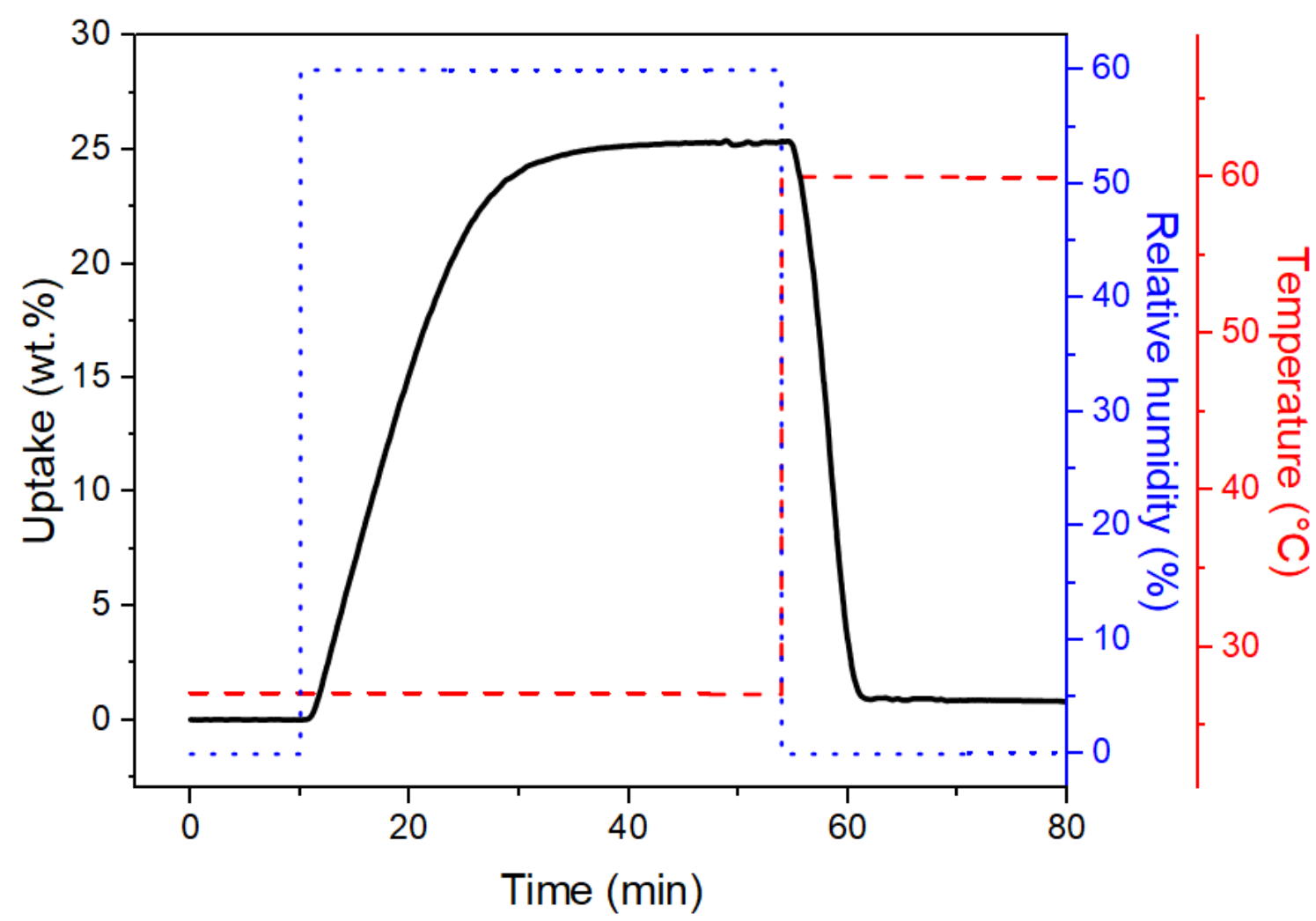

Figure S167 Water vapor sorption T-RH-swing kinetics measurements carried out on **TIFSIX-25-Ni**. Adsorption conditions: 60% RH, 27 °C; desorption conditions: 0% RH, 60 °C.

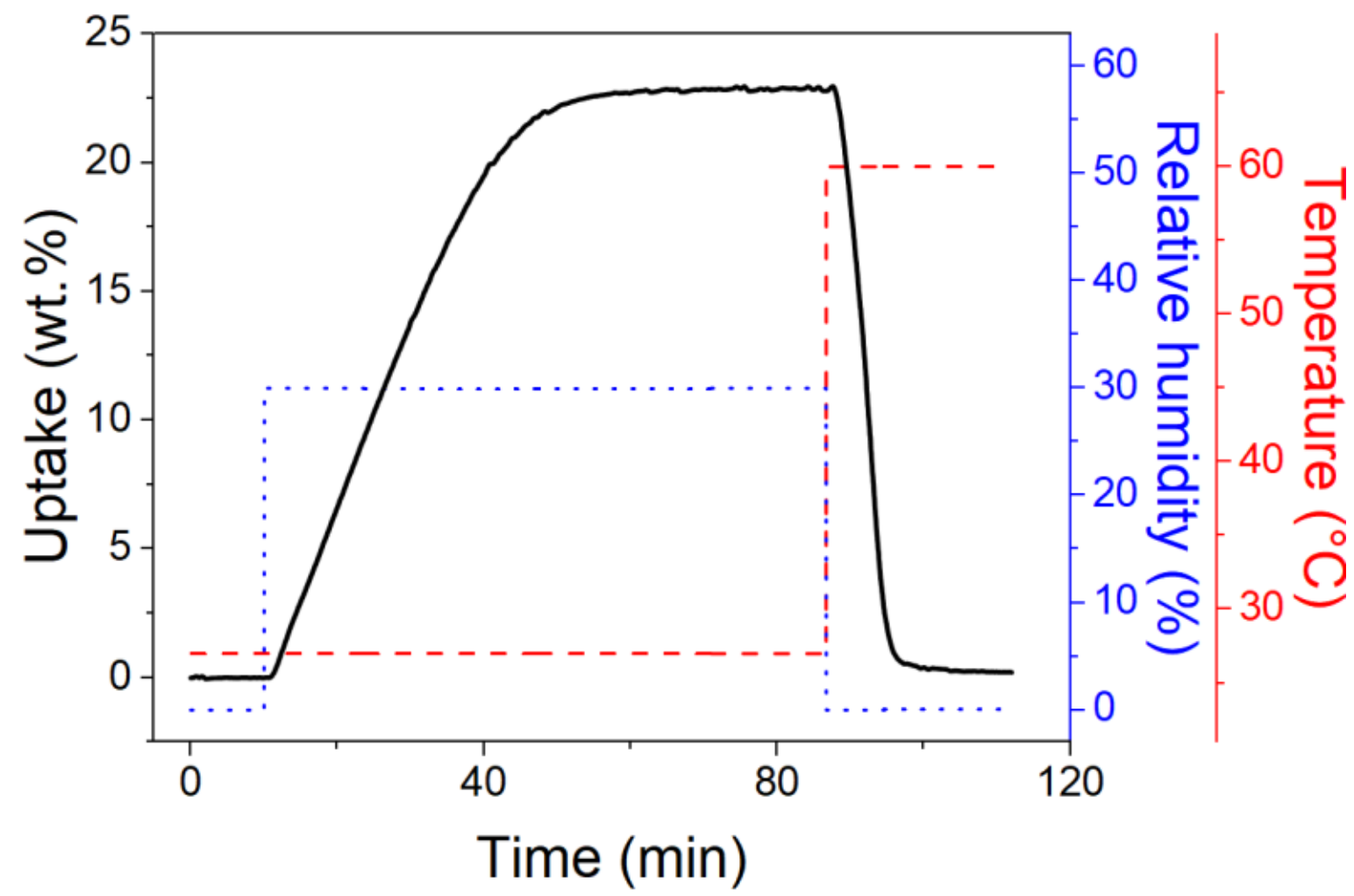

Figure S168 Water vapor sorption T-RH-swing kinetics measurements carried out on **TIFSIX-25-Co**. Adsorption conditions: 30% RH, 27 °C; desorption conditions: 0% RH, 60 °C.

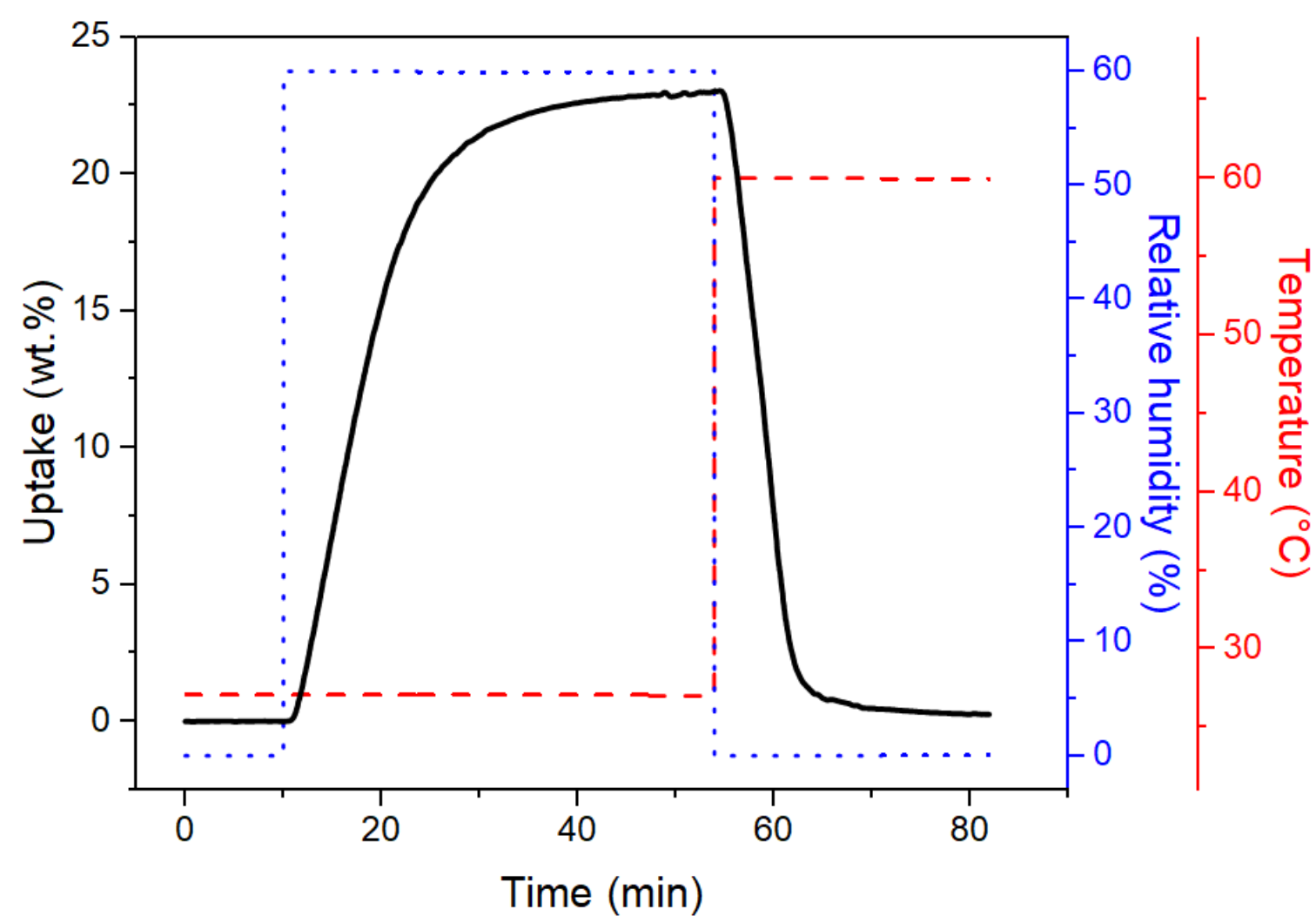

Figure S169 Water vapor sorption T-RH-swing kinetics measurements carried out on **TIFSIX-25-Co**. Adsorption conditions: 60% RH, 27 °C; desorption conditions: 0% RH, 60 °C.

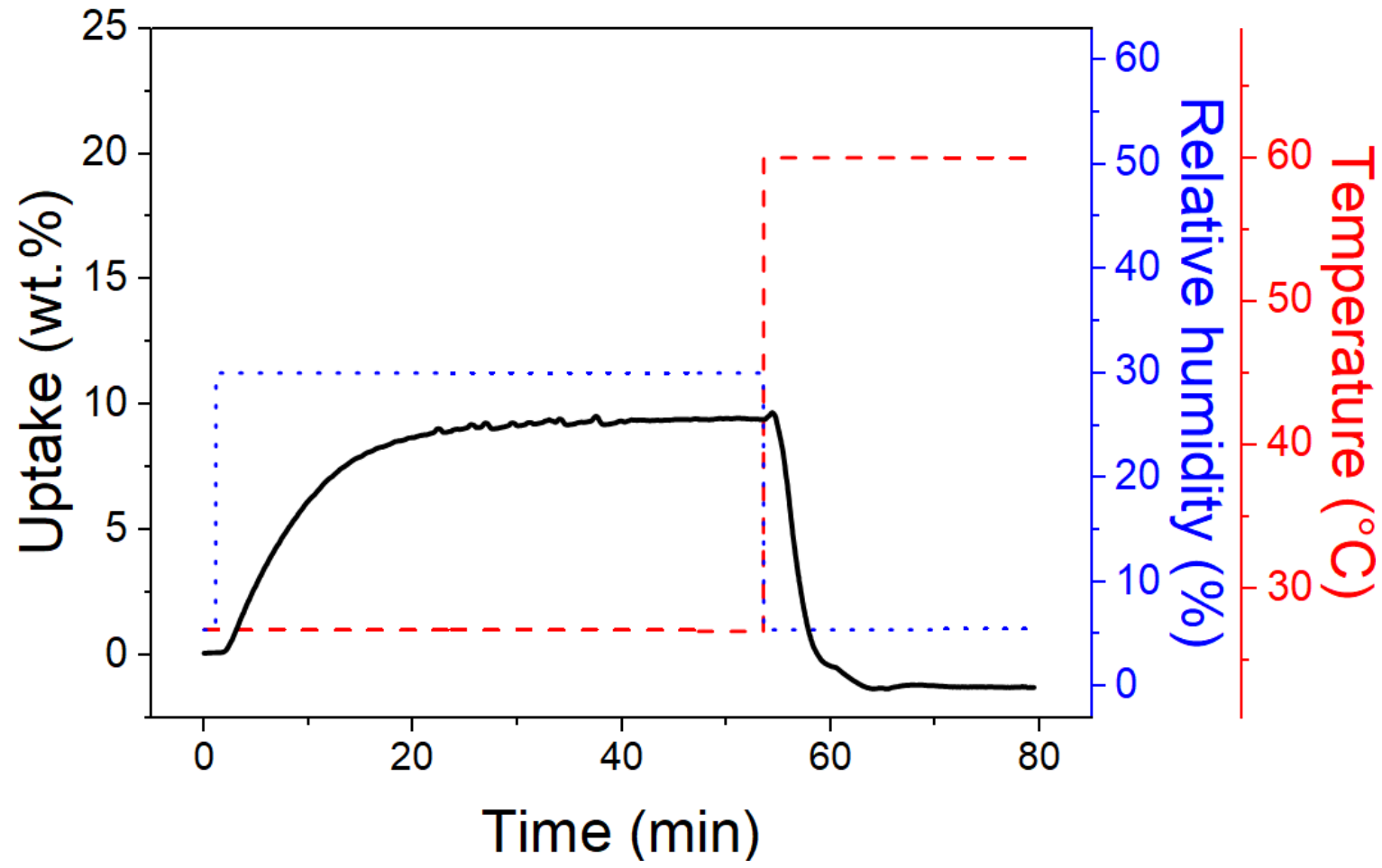

Figure S170 Water vapor sorption T-swing kinetics measurements carried out on **TIFSIX-25-Co**. Adsorption conditions: 30% RH, 27 °C; desorption conditions: 5.4% RH, 60 °C.

18 Temperature-swing kinetics

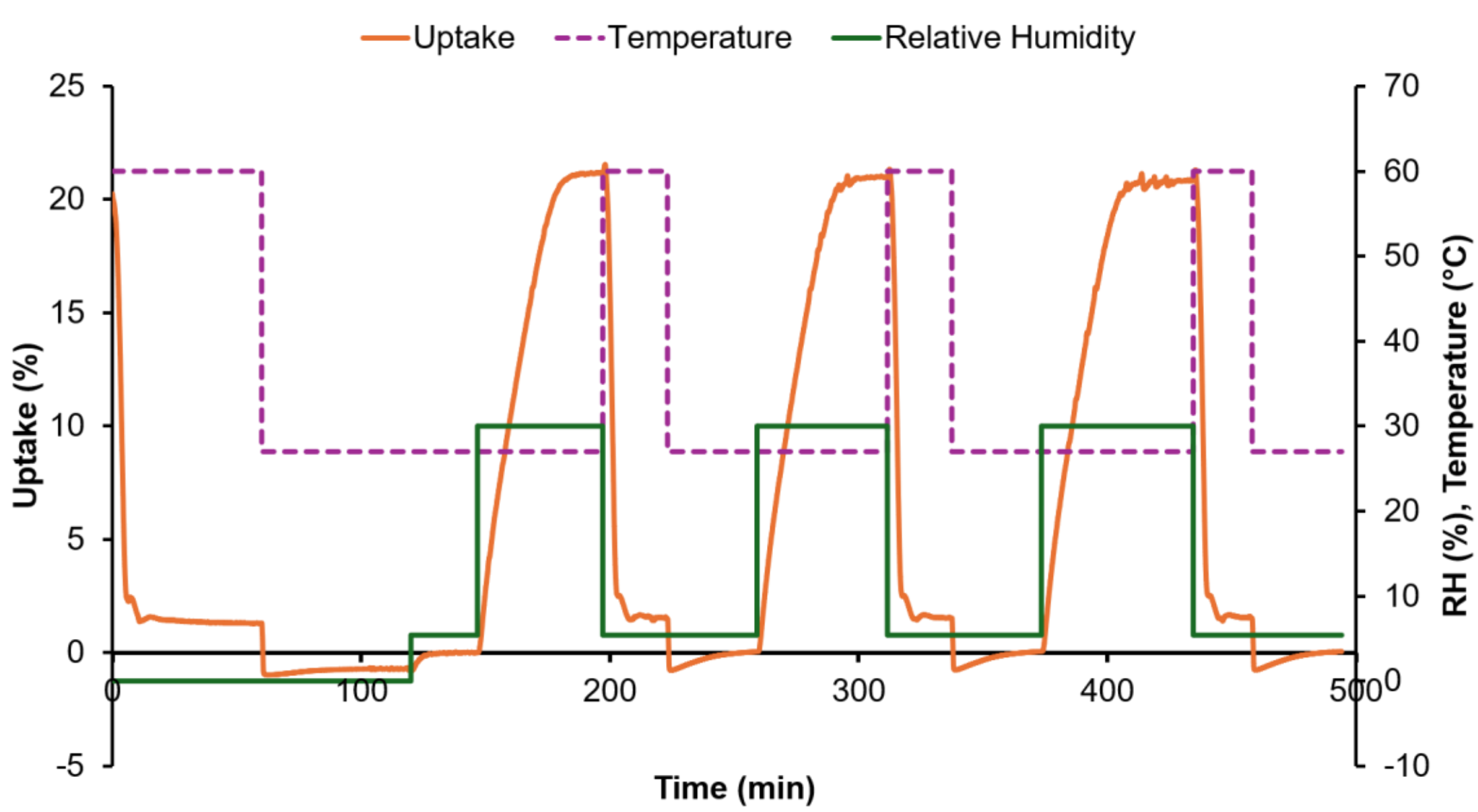

Figure S171 Water vapor sorption temperature-swing kinetics measurements carried out on **TIFSIX-25-Ni**. Adsorption conditions: 30% RH, 27 °C; desorption conditions: 5.4% RH, 60 °C. A sample mass (initial) of 4.05 mg was used.

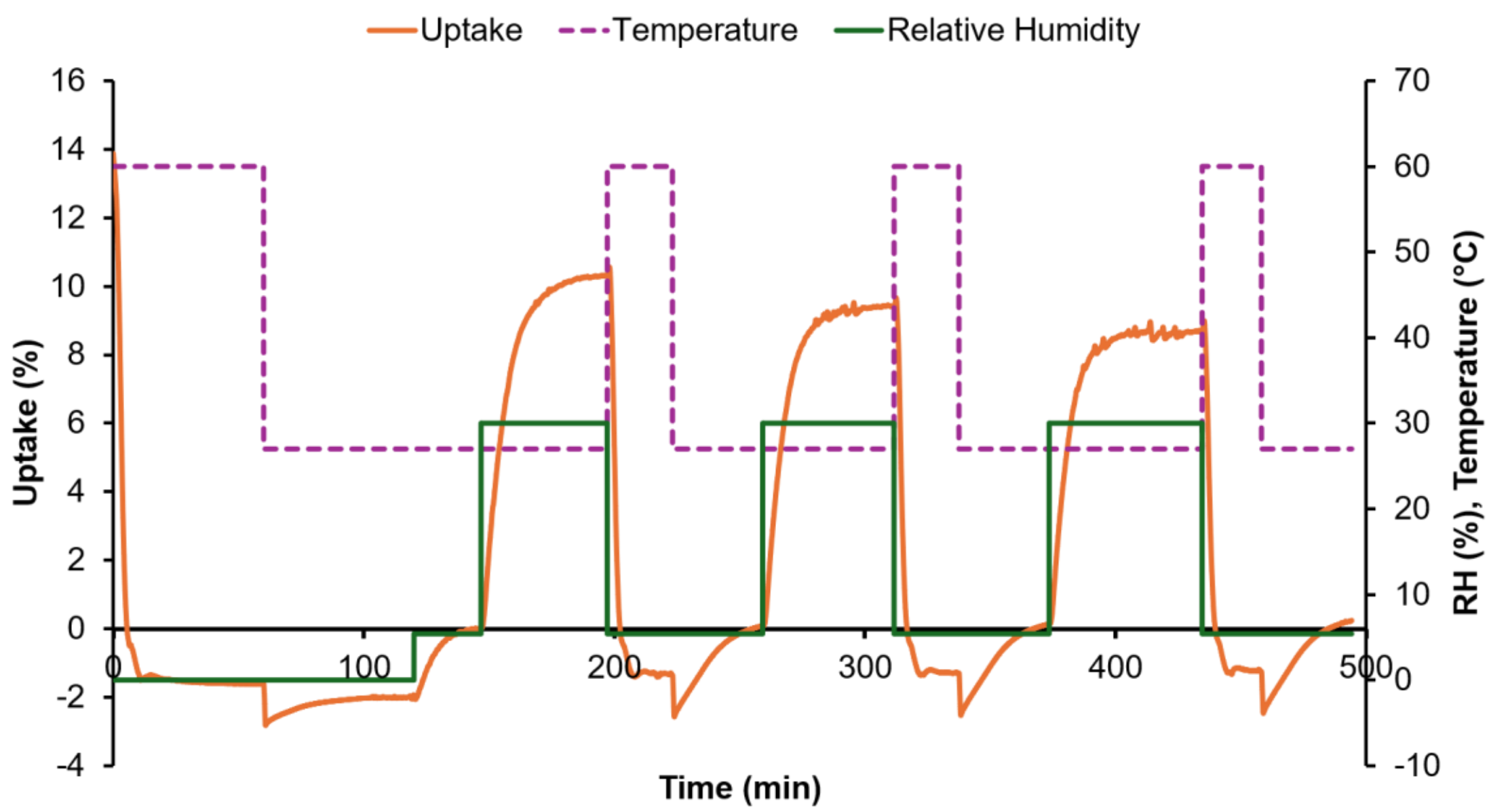

Figure S172 Water vapor sorption temperature-swing kinetics measurements carried out on **TIFSIX-25-Co**. Adsorption conditions: 30% RH, 27 °C; desorption conditions: 5.4% RH, 60 °C. A sample mass (initial) of 5.13 mg was used.

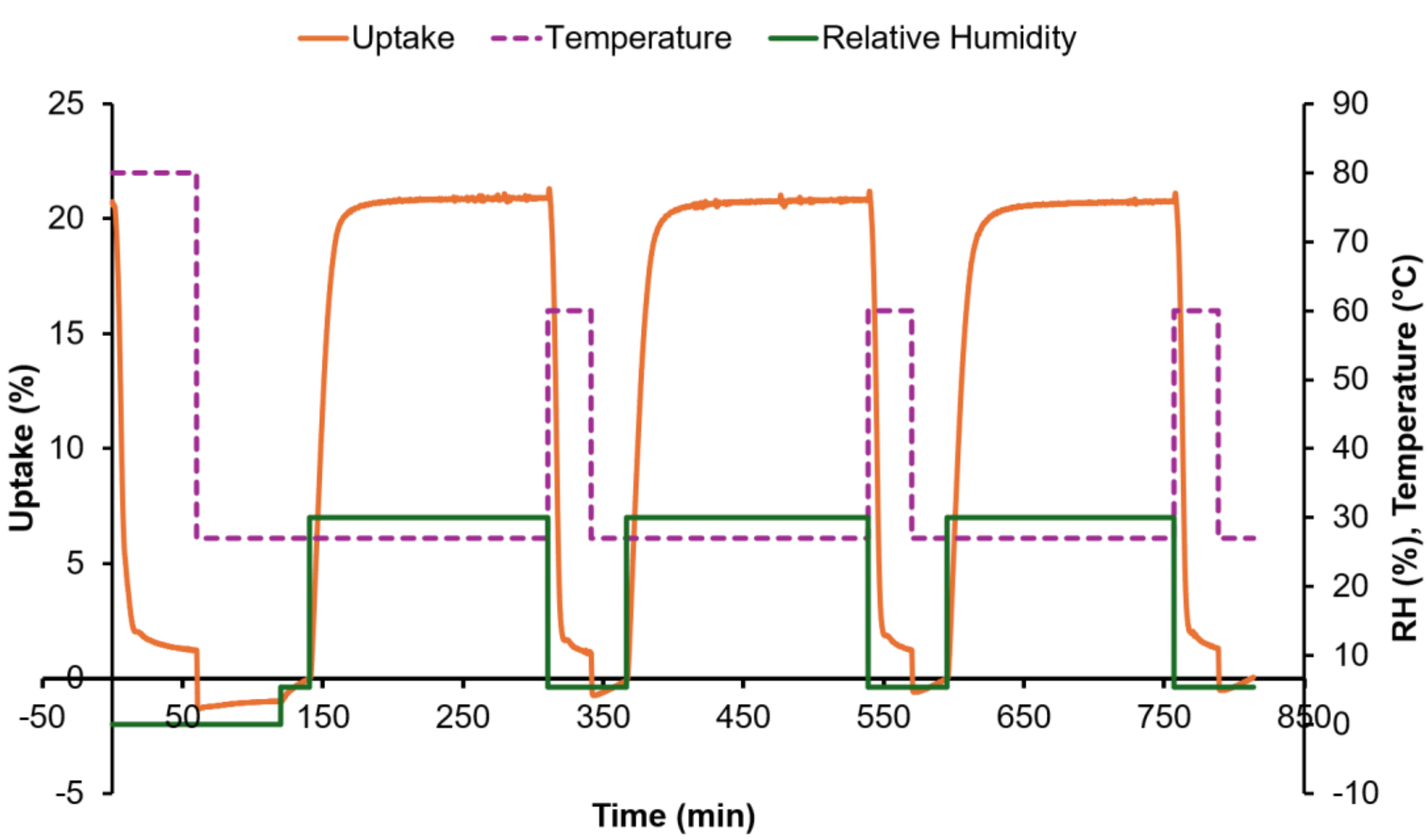

Figure S173 Water vapor sorption temperature-swing kinetics measurements carried out on **ROS-039**. Adsorption conditions: 30% RH, 27 °C; desorption conditions: 5.4% RH, 60 °C. A sample mass (initial) of 5.20 mg was used.

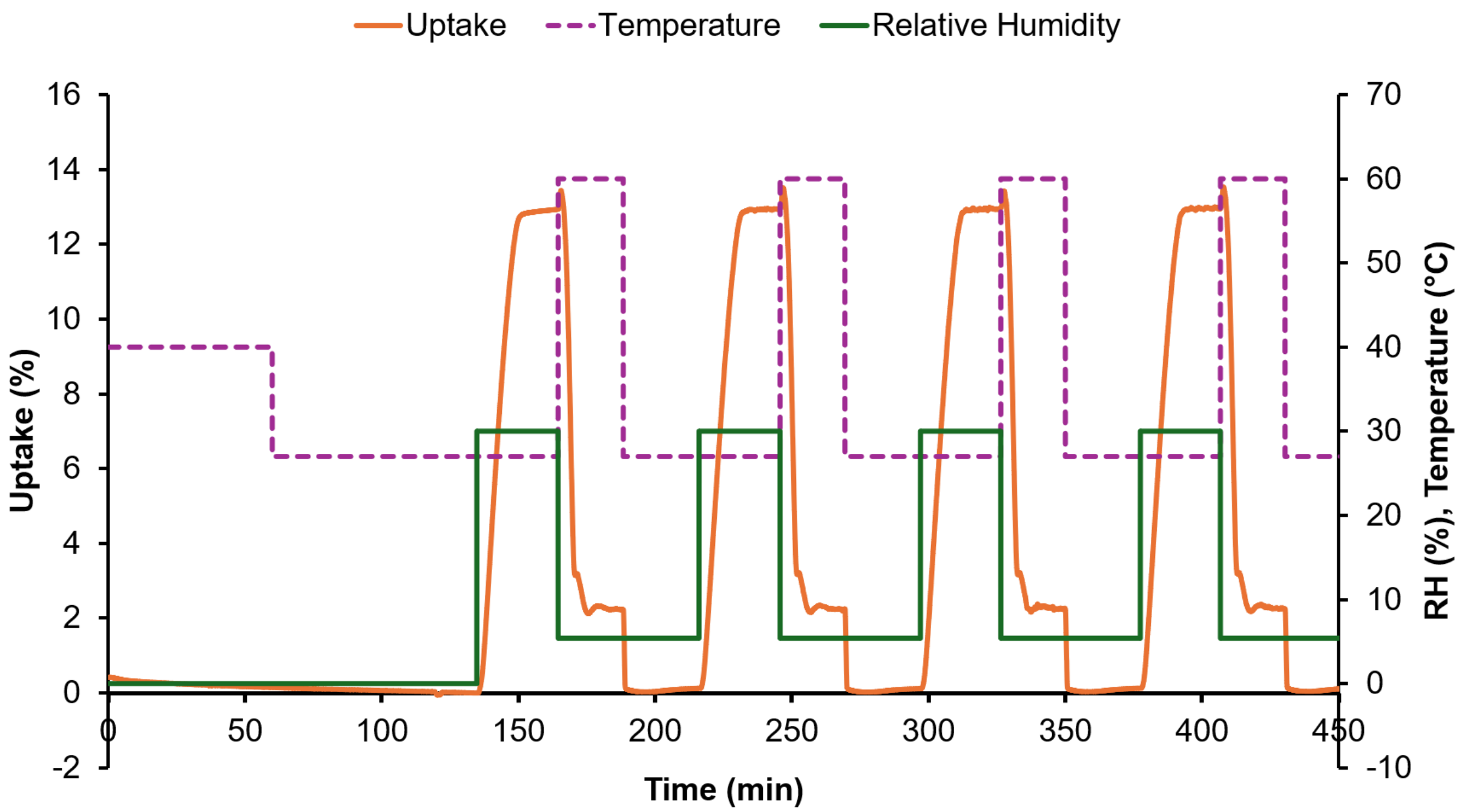

Figure S174 Water vapor sorption temperature-swing kinetics measurements carried out on **dia-SNIFFOH-1-Cu**. Adsorption conditions: 30% RH, 27 °C; desorption conditions: 5.4% RH, 60 °C. A sample mass (initial) of 5.0 mg was used.

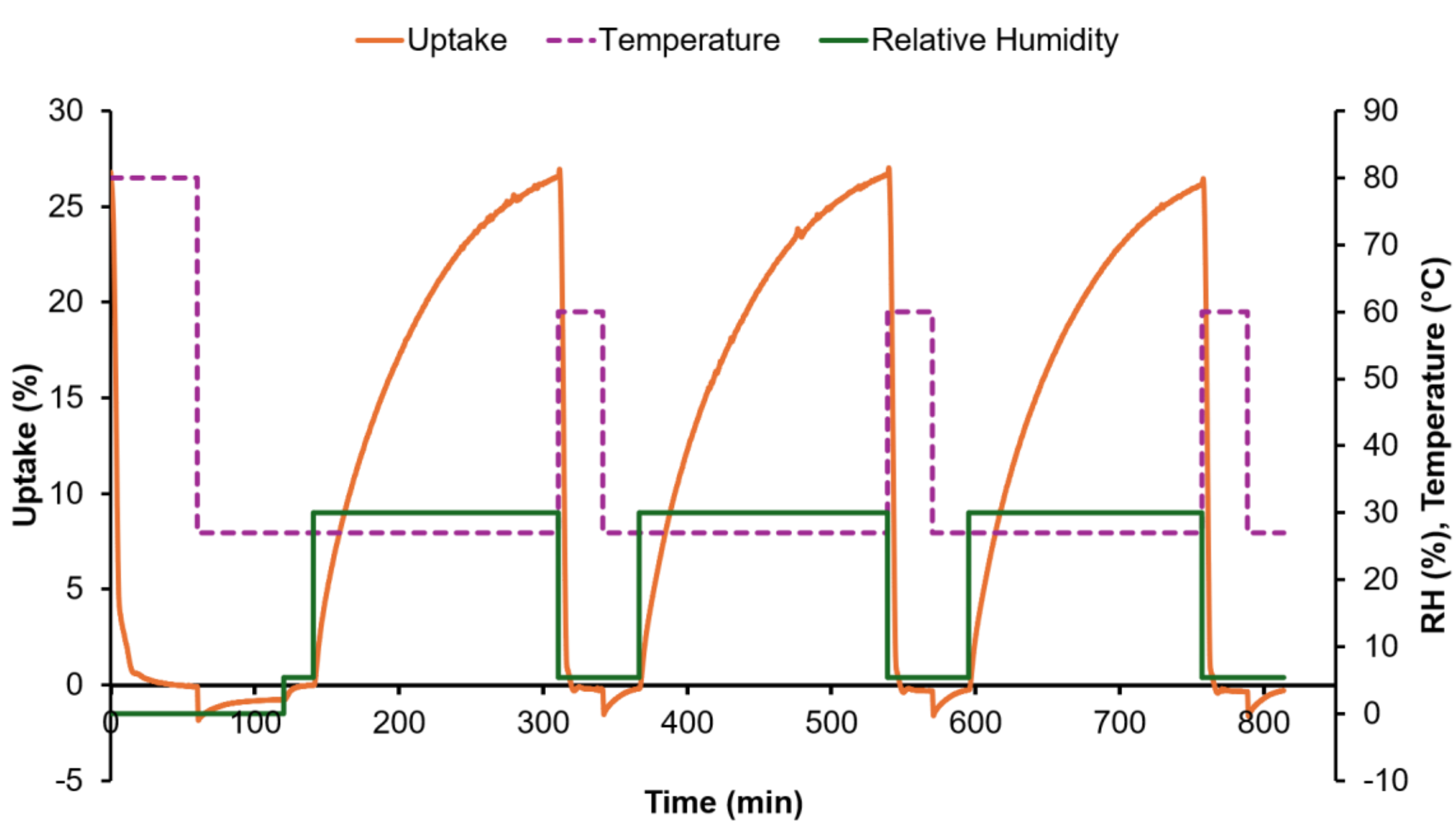

Figure S175 Water vapor sorption temperature-swing kinetics measurements carried out on **Al-OH-fumarate**. Adsorption conditions: 30% RH, 27 °C; desorption conditions: 5.4% RH, 60 °C. A sample mass (initial) of 4.97 mg was used.

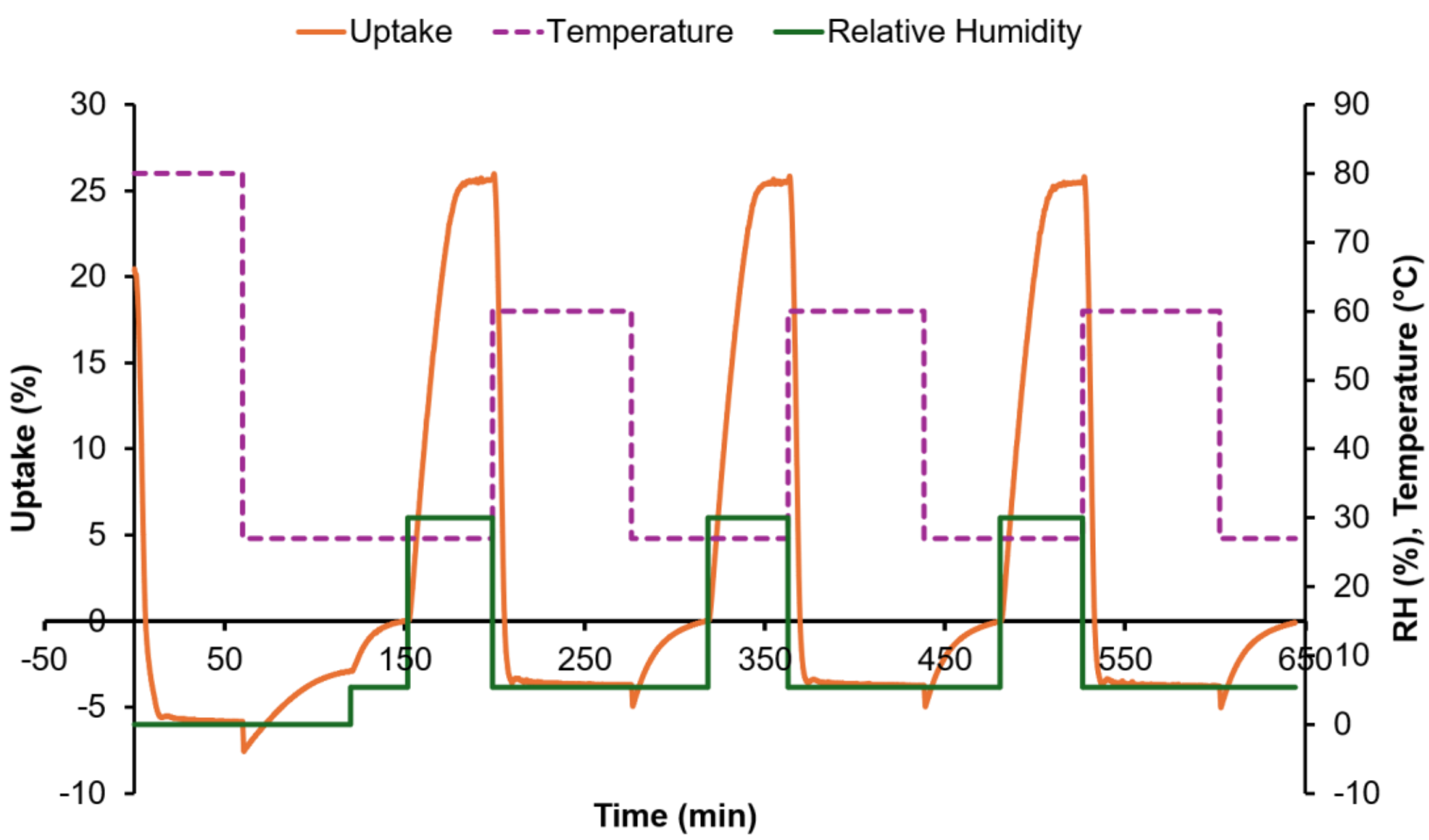

Figure S176 Water vapor sorption temperature-swing kinetics measurements carried out on **MOF-303**. Adsorption conditions: 30% RH, 27 °C; desorption conditions: 5.4% RH, 60 °C. A sample mass (initial) of 4.55 mg was used.

19 Estimation of the thermal conductivities of ROC-1 and ROC-2 by modulated DSC analysis

The thermal conductivities (k) of **ROC-1** and **ROC-2** were estimated using the modulated differential scanning calorimetry (MDSC) method developed by Marcus and Blaine,[28-29] and subsequently adopted by the American Society for Testing and Materials (ASTM International) as E1952-01.[30] This method has been applied to a wide range of materials with thermal conductivities in the range 0.1 – 2 W m^{-1} °C^{-1}. The pertinent equations are outlined below; full derivations are provided in the cited references.

19.1 Theory

Marcus and Blaine derived Eqn S1 to determine the observed thermal conductivity k_o, (W m^{-1} C^{-1}), of samples formed into right cylinders using MDSC analysis:[28-29]

$$k_o = \frac{8LC^2}{C_p M d^2 P} \qquad \text{[S1]}$$

where L is the sample thickness (mm), C is the apparent heat capacity (mJ °C^{-1}), C_p is the specific heat capacity (J g^{-1} °C^{-1}), M is the sample mass (mg), d is the sample diameter (mm), and P is the modulation period (s).

The accuracy of C_p depends on the experimental conditions and sample geometry. For a sufficiently thin sample subjected to temperature oscillations with a suitably long period (P), the sample is assumed to approach a uniform temperature distribution, enabling accurate determination of C_p. In contrast, a sufficiently thick sample develops a temperature gradient across the sample that depends on its thermal diffusivity. Under these conditions, the apparent heat capacity is proportional to the square root of the thermal conductivity. The thermal conductivity can therefore be estimated from the specific heat capacity determined using a thin sample and the apparent heat capacity of a thick sample of controlled geometry.

For materials with low thermal conductivity, such as coordination polymers, where k approaches that of the purge gas (N_2, k = 0.026 W m^{-1} °C^{-1}), heat losses from the thick sample become significant. A thermal conductivity calibration constant, D, is therefore applied to k_0 to account for

these losses. D is determined by measuring k_0 for thin and thick samples of a reference material with a known thermal conductivity, k_{true}:

$$D = \sqrt{k_0 \cdot k_{true}} - k_{true} \quad \text{[S2]}$$

The corrected thermal conductivity, k, of a test sample is then determined using Eqn S3:

$$k = \frac{1}{2} \cdot \left[k_0 - 2D + \sqrt{k_o^2 - 4Dk_0}\right] \quad \text{[S3]}$$

19.2 Experimental details

MDSC measurements were performed using a TA Instruments DSC Q2000 (V24.11, Build 124). Normal calibration of the DSC was performed using indium metal and sapphire standard reference materials. Iodine (I_2, 99.8%) was selected as the reference material for determination of D because of its known thermal conductivity (0.45 W m^{-1} °C^{-1} at 27 °C)[31] and its availability as a powder, providing a sample form comparable to **ROC-1** and **ROC-2**. The I_2 was purified by sublimation and subsequently ground to a fine powder. Sample masses were measured using a Mettler Toledo MS105 balance (accuracy ±0.01 mg). Sample dimensions were measured using a Hilka Vernier caliper (150 mm range; accuracy ±0.02 mm). In a typical experiment, the sample was loaded into a Tzero aluminium pan (internal diameter = 4.80 ± 0.01 mm; pan height = 2.36 ± 0.02 mm) and sealed with a lid (error represents the standard error of the mean). The mass of the pan was recorded before and after sample loading to determine the sample mass. Before analysis, bulk samples of **ROC-1** and **ROC-2** were activated under dynamic vacuum (1 mbar) for 30 min to remove adsorbed water and afford the **pcu** phase, thereby preventing dehydration from contributing to the measured signal, as solvent content is known to influence thermal conductivity of sorbents.[32-33] The pan lids were pierced with a pinhole to enable removal of any moisture adsorbed during sample handling under the N_2 purge gas. Samples were heated from 25 to 60 °C at a nominal heating rate of 1 °C min^{-1}. Analysis of the temperature profile indicated that a constant heating rate was established between 29 and 60 °C. Consequently, values at 30 °C were used for comparison. The modulation period and amplitude were 60 s and 1.0 °C, respectively.

19.3 Calibration and thermal conductivity determination

The C_p of I_2 was determined from three measurements using thin samples (0.34 ± 0.04 mm, Figure S177), yielding an average value of 0.20 ± 0.02 J g^{-1} °C^{-1} at 30 °C. This value is in good agreement with the reported reference value of 0.215 J g^{-1} °C^{-1}.[34] Apparent heat capacities (*C*) were similarly determined for three thick I_2 samples (Table S10). The resulting observed thermal conductivity, k_0, of I_2 at 30 °C was 0.26 ± 0.03 W m^{-1} °C^{-1}, compared with the reported value of 0.45 W m^{-1} °C^{-1}.[31] From these measurements, the thermal conductivity calibration constant *D* was determined to be −0.11 ± 0.02 W m^{-1} °C^{-1}. It should be noted that these measurements were performed on powdered I_2 samples, which have substantially lower apparent density (3.51 ± 0.15 g cm^{-3}) than the crystallographic density of iodine (4.93 g cm^{-3}). The lower experimental thermal conductivity relative to the reported value is therefore unsurprising, as interparticle voids within the powder reduce effective heat transfer.[35] Given that **ROC-1** and **ROC-2** were also measured as microcrystalline powders, the powdered I_2 samples provide a representative reference.

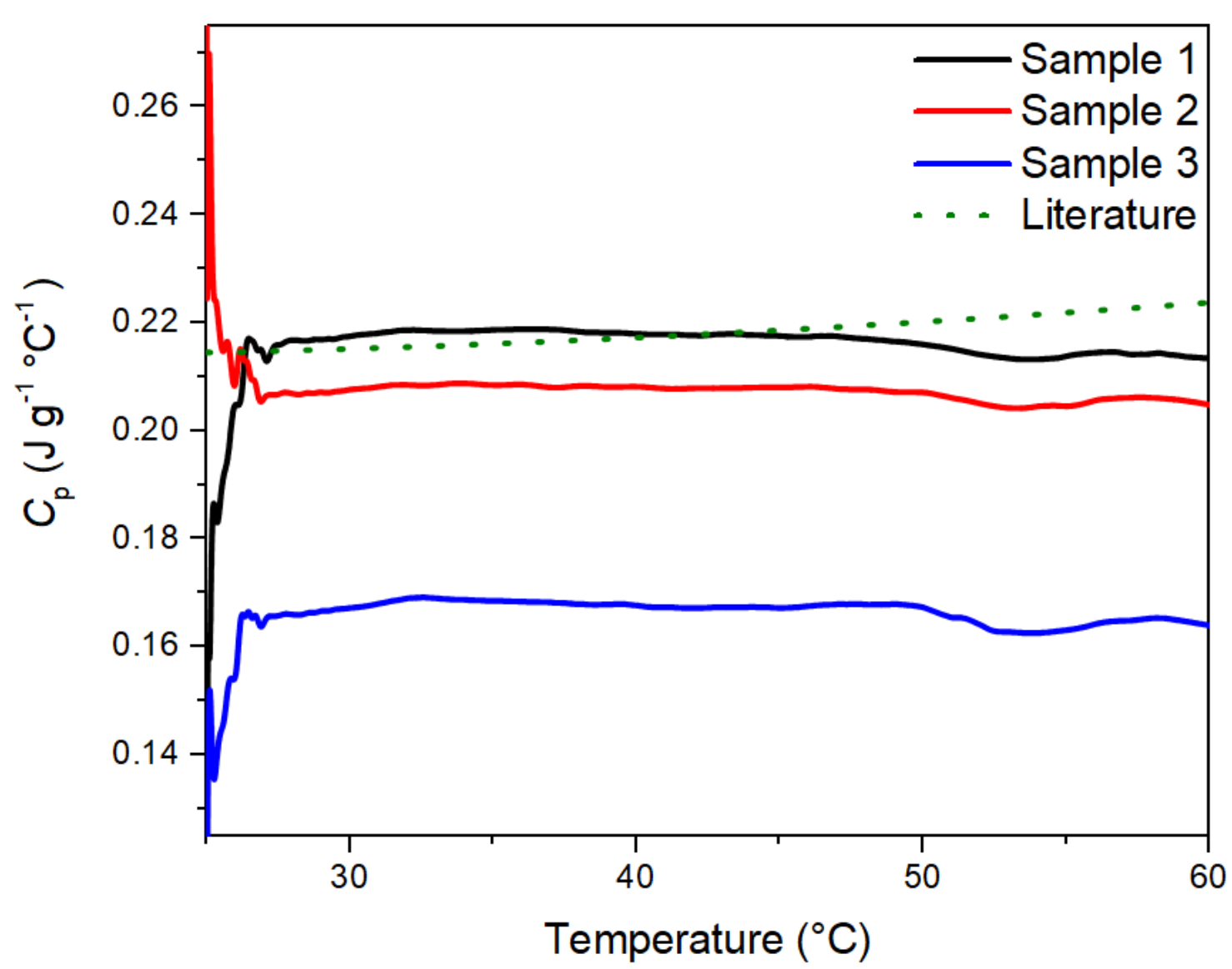

Figure S177 Specific heat capacity (C_p) values determined for three I_2 samples by MDSC during heating from 25 to 60 °C at 1 °C min^{-1}. Literature values shown as a green dotted line.

Table S10 Experimental parameters and corresponding k_0 and D values determined at 30 °C for three thick I_2 samples.

Parameter	Sample 1	Sample 2	Sample 3
M (mg)	155.2	156.4	133.0
L (mm)	2.3	2.4	2.4
d (mm)	4.8	4.8	4.7
Apparent density (g cm^{-3})	3.7	3.6	3.2
C (mJ °C^{-1})	24.1	22.4	24.1
k_o (W m^{-1} °C^{-1})	0.25	0.22	0.31
D (W m^{-1} °C^{-1})	-0.11	-0.13	-0.08

The C_p and thermal conductivity values of **ROC-1** and **ROC-2** were determined using the same procedure (Figure S178; Table S11). At 30 °C, C_p values of 0.87 ± 0.15 and 0.81 ± 0.08 J g^{-1} °C^{-1} were obtained for **ROC-1** and **ROC-2**, respectively. Following application of the calibration correction, thermal conductivities of 0.25 ± 0.02 W m^{-1} °C^{-1} for ROC-1 and 0.38 ± 0.04 W m^{-1} °C^{-1} for **ROC-2** were obtained. These values fall within the range typically reported for coordination polymers.[36]

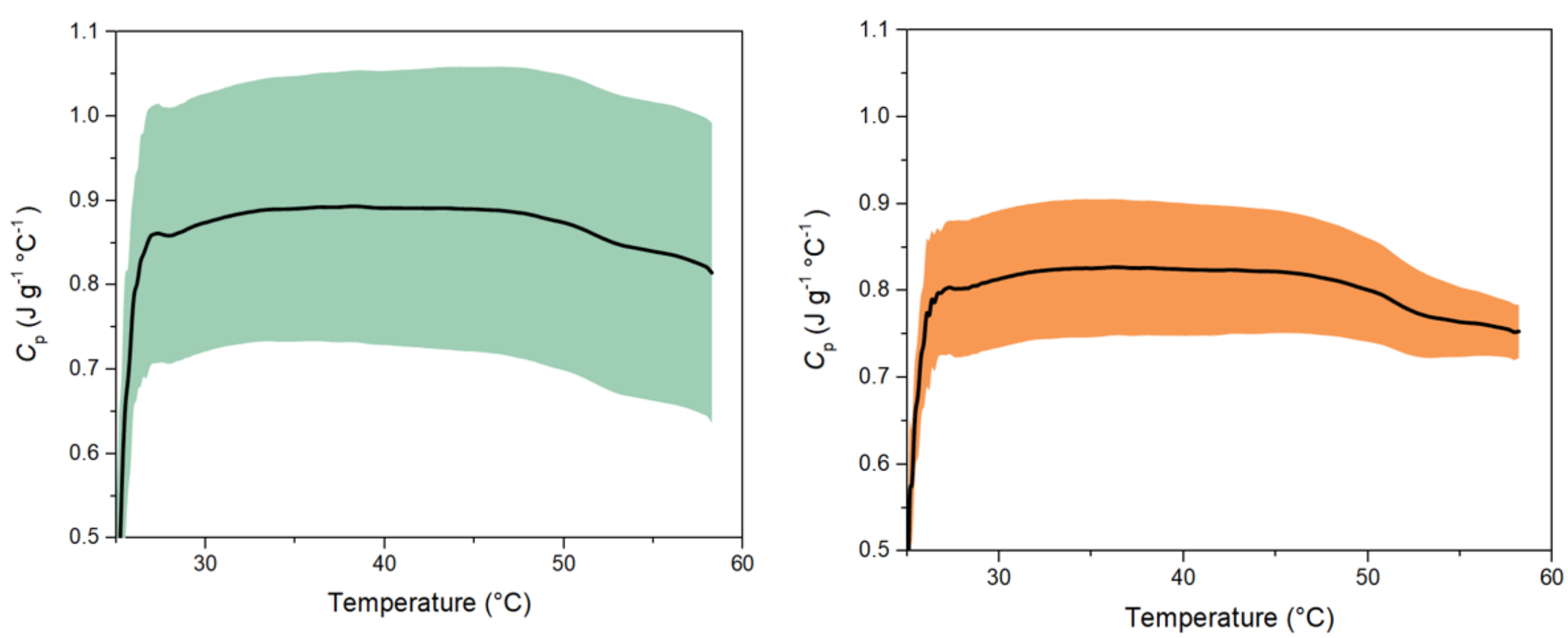

Figure S178 Average specific heat capacity (C_p) values determined for three **ROC-1** (left) and **ROC-2** (right) samples by MDSC during heating from 25 to 60 °C at 1 °C min^{-1}. Shaded regions are standard error.

Table S11 Experimental parameters and corresponding k_0 and k values determined at 30 °C for three thick **ROC-1** and **ROC-2** samples.

Parameter	**ROC-1** samples			**ROC-2** samples		
	1	2	3	1	2	3
M (mg)	15.4	19.1	13.4	18.6	20.3	21.8
L (mm)	1.1	1.3	1.0	1.6	1.9	1.9
d (mm)	4.8	4.8	4.8	4.8	4.8	4.8
Apparent density (g cm^{-3})	0.8	0.8	0.7	0.6	0.6	0.6
C (mJ $°C^{-1}$)	15.3	13.6	10.3	14.5	19.0	18.6
k_o (W m^{-1} $°C^{-1}$)	0.11	0.08	0.05	0.13	0.25	0.21
k (W m^{-1} $°C^{-1}$)	0.28	0.25	0.21	0.31	0.44	0.40

20 Water harvesting productivity heatmaps

Water sorption performance heatmap of **TIFSIX-25-Ni** under temperature swing conditions ([30% RH, 27°C] – [5.4% RH, 60°C]) was calculated using method from Bezrukov et al.[27]

$$\frac{dw}{dt} = k \cdot (RH_{flow} - RH_{bed}),$$
$$k = k' \cdot m \qquad [S4]$$

where w is uptake (wt.%), k is sorption coefficient, m = sample mass, RH_{flow} is relative humidity in the flow and RH_{bed} is relative humidity in sample bed.

The method uses Eqn. S4 to describe water vapor sorption kinetics, with physical meaning of sorption kinetics limited by extrinsic diffusion to sorbent bed surface. RH_{bed} is a function of uptake and is determined from the corresponding isotherms.

Temperature swing heatmap ([30% RH, 27°C] – [5.4% RH, 60°C]) was calculated using 3 step protocol:

Step 1a: The experimental adsorption kinetics of **TIFSIX-25-Ni** for at 27°C at 4 different sample masses was fitted using Eqn. S4 to obtain k (Figures S179-182), adsorption branch of the isotherm at 27°C was used to fit kinetics. Two parameters were fitted: k and t0, where t0 was fitted in 0-3 minutes range.

Step 2a: Adsorption kinetics coefficient k' at 27°C was determined from the slope of the {*k – 1/m*} plot (Figure S183).

Step 1b: The experimental desorption kinetics of **TIFSIX-25-Ni** at 60°C at 4 different sample masses was fitted using Eqn. S4 to obtain k (Figures S185-S188), desorption branch of the isotherm at 60°C was used to fit kinetics. Two parameters were fitted: k and t0, where t0 was fitted in 0-3 minutes range.

Step 2b: Desorption kinetics coefficient k' at 60°C was determined from the slope of the {*k – 1/m*} plot (Figure S189).

Step 3: Adsorption-desorption cycling kinetics was modelled for adsorption at [30% RH, 27°C] and desorption at [5.4% RH, 60°C]. For both 27°C and 60°C, k was determined from corresponding k' using m = 10 mg. Adsorption and desorption branches of the isotherm at corresponding temperatures were used to model adsorption and desorption kinetics

respectively. Adsorption-desorption cycling was calculated for times in a range 1−50 min with 1 min step size.

20.1 Sorption kinetics analysis

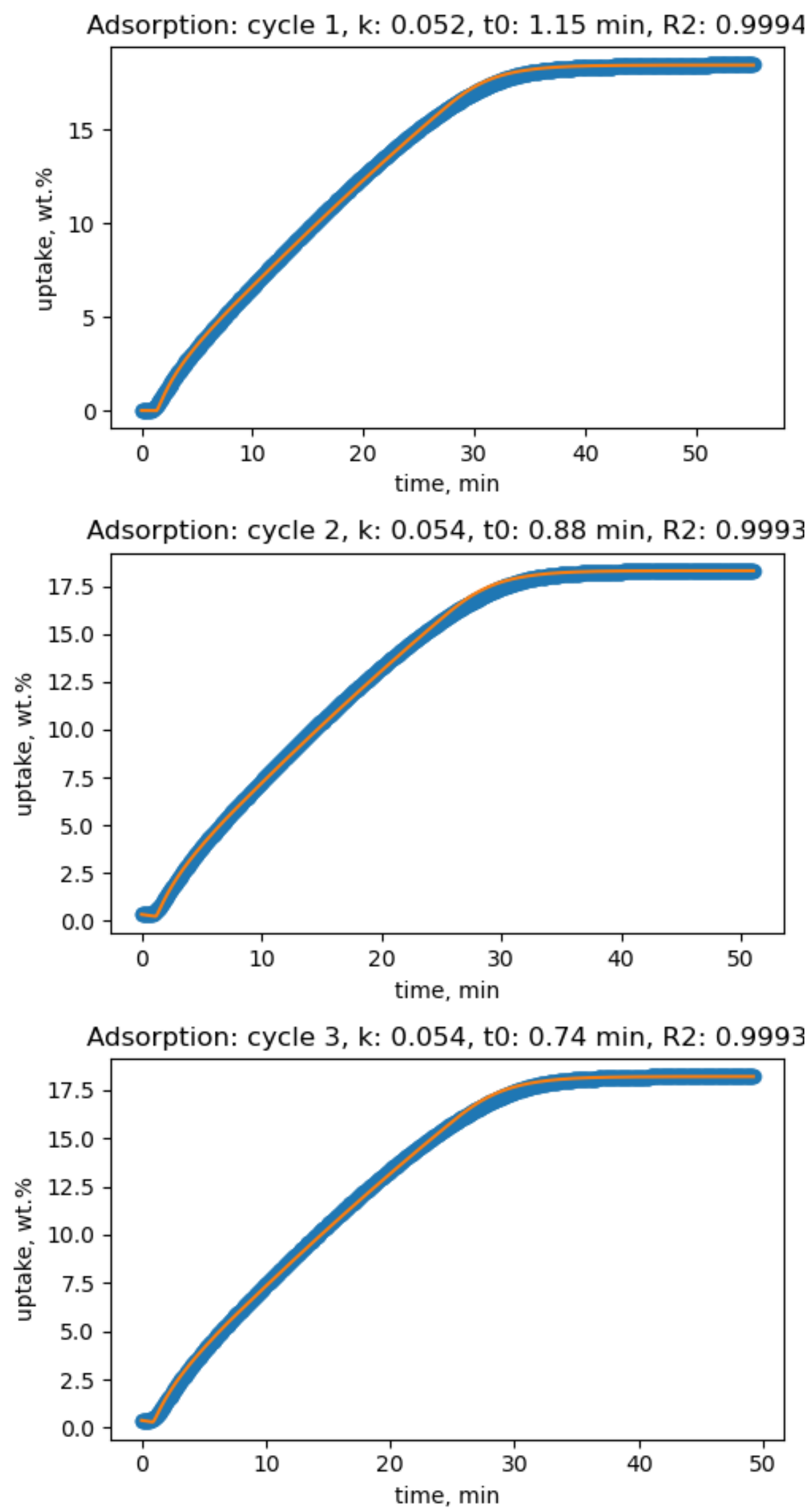

Figure S179 Fit of 0 – 30 %RH humidity swing adsorption kinetics data (blue) at 27 °C on **TIFSIX-25-Ni** sample (5.0 mg) using extrinsic diffusion model (orange).

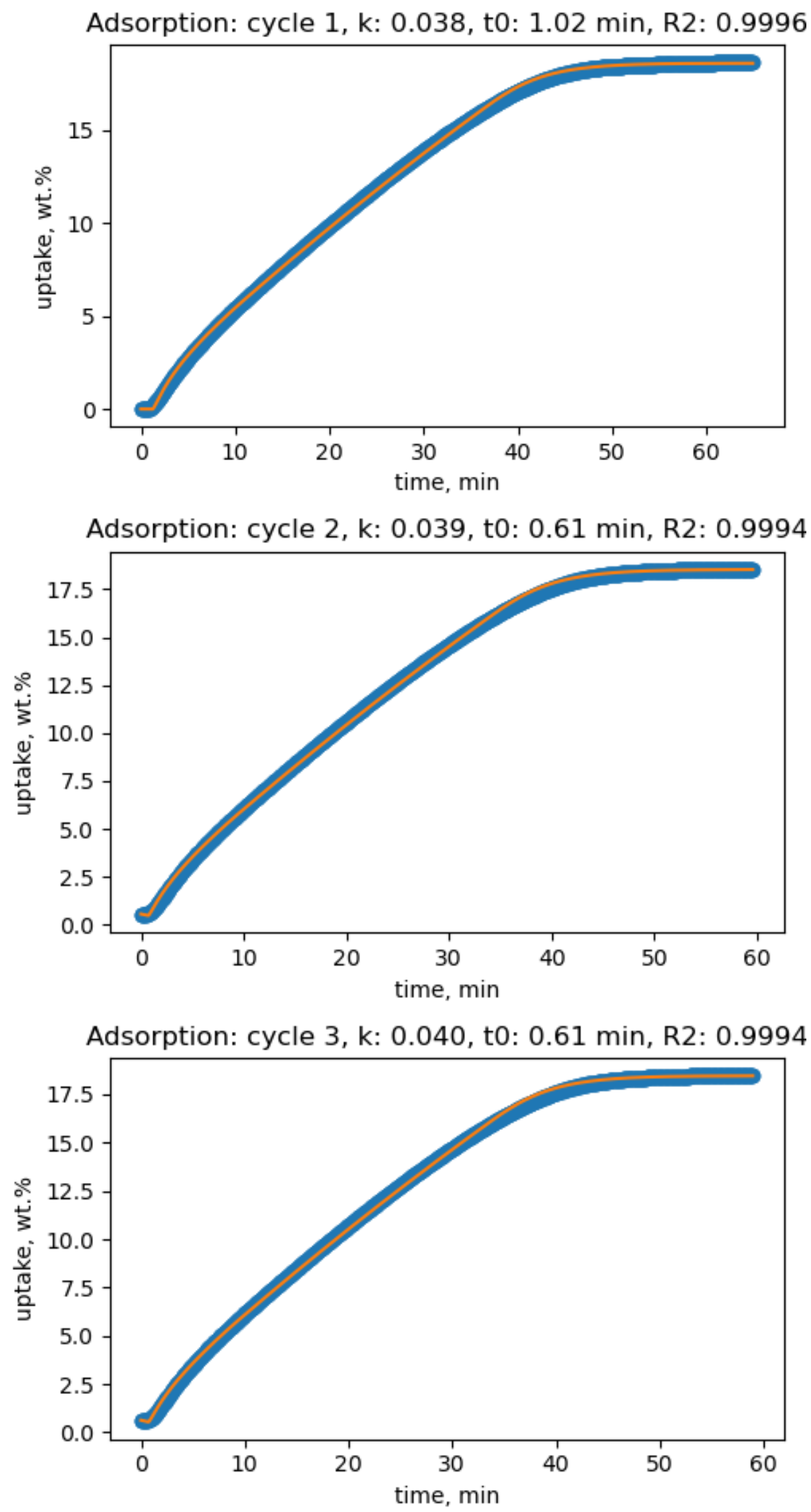

Figure S180 Fit of 0 – 30 %RH humidity swing adsorption kinetics data (blue) at 27 °C on **TIFSIX-25-Ni** sample (7.4 mg) using extrinsic diffusion model (orange).

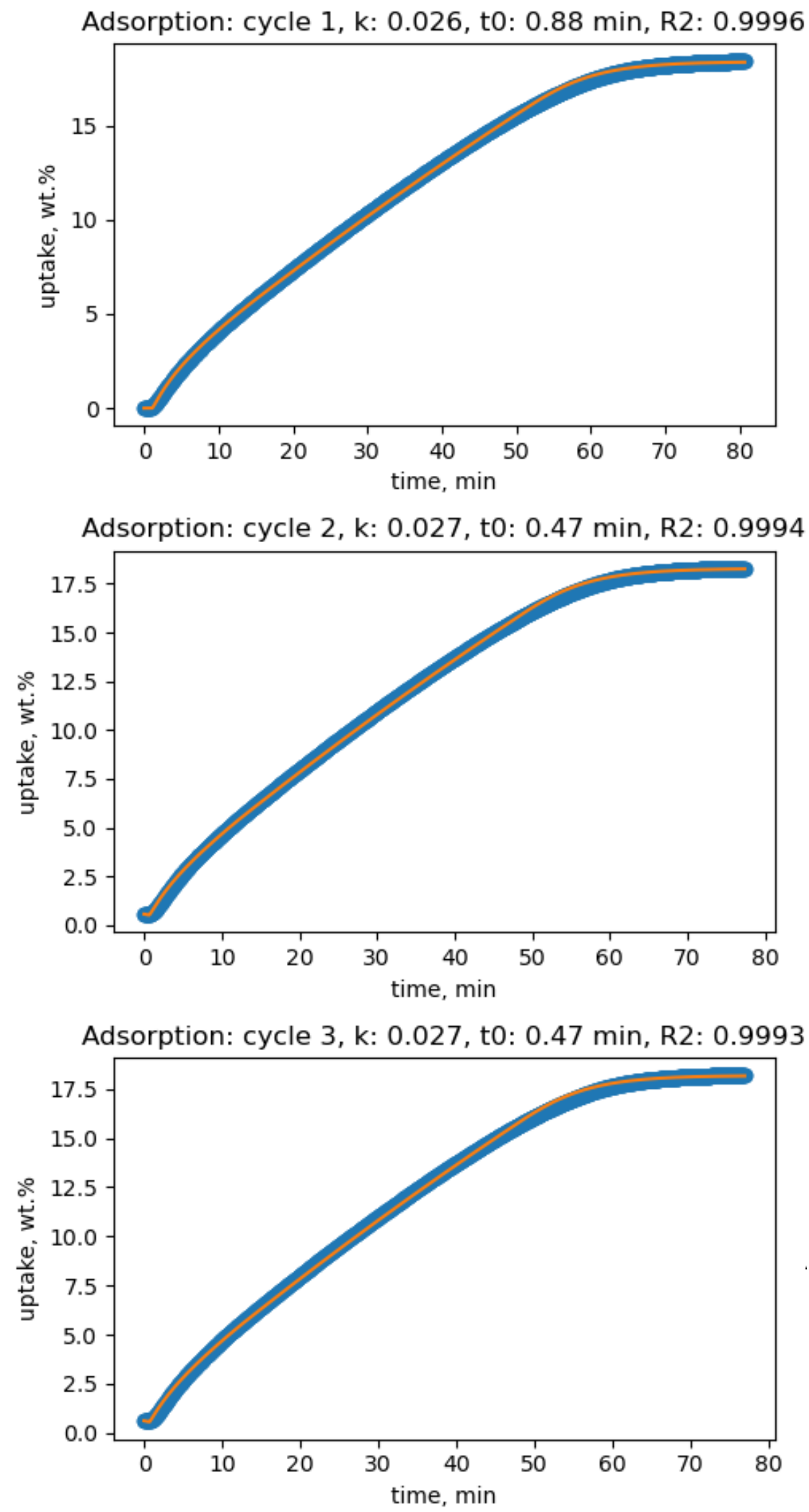

Figure S181 Fit of 0 – 30 %RH humidity swing adsorption kinetics data (blue) at 27 °C on **TIFSIX-25-Ni** sample (11.6 mg) using extrinsic diffusion model (orange).

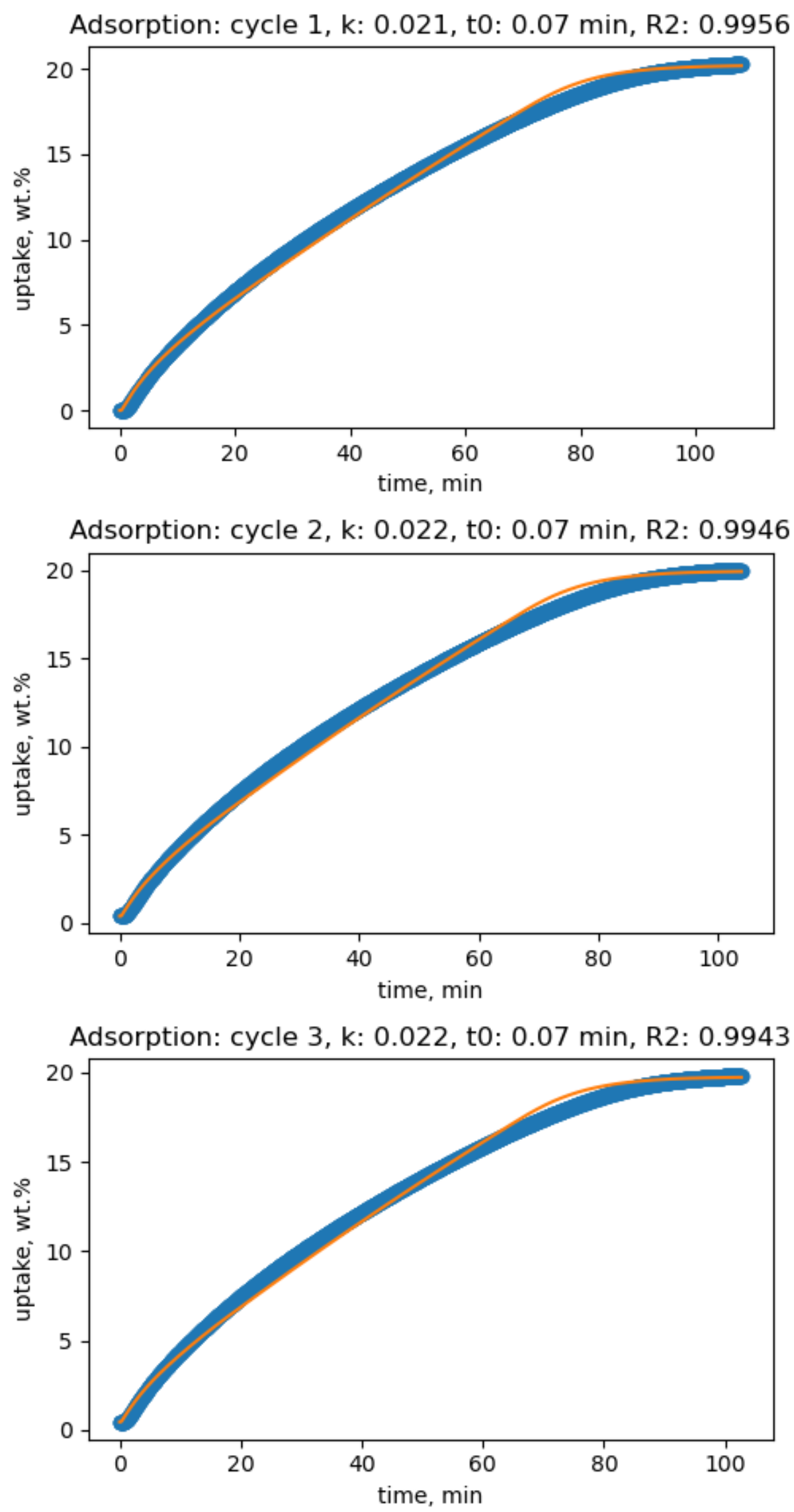

Figure S182 Fit of 0 – 30 %RH humidity swing adsorption kinetics data (blue) at 27 °C on **TIFSIX-25-Ni** sample (17.9 mg) using extrinsic diffusion model (orange).

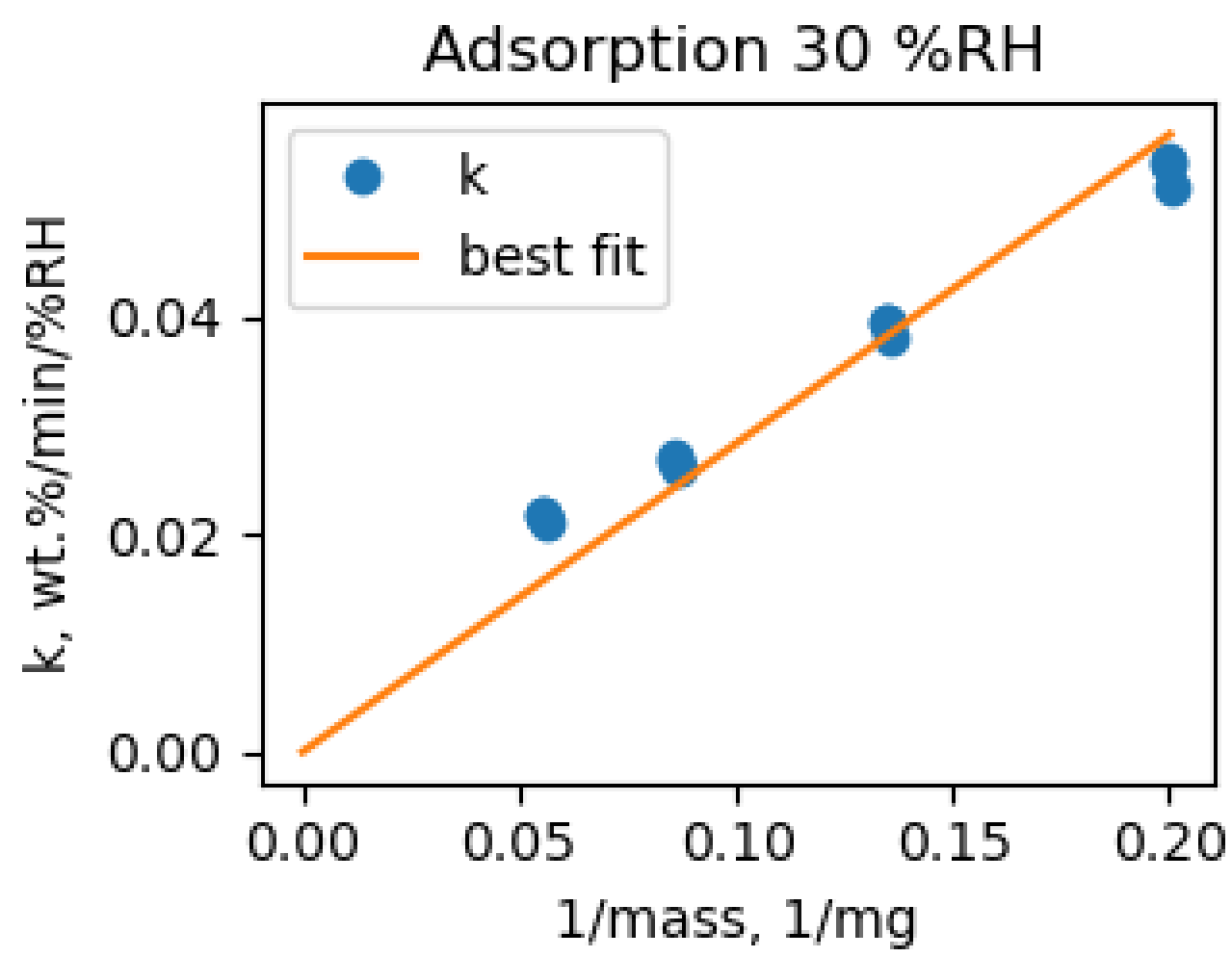

Figure S183 Kinetics coefficient *k* as function of mass of the sample in 0-30 %R humidity swing experiments on **TIFSIX-25-Ni** at 27 °C.

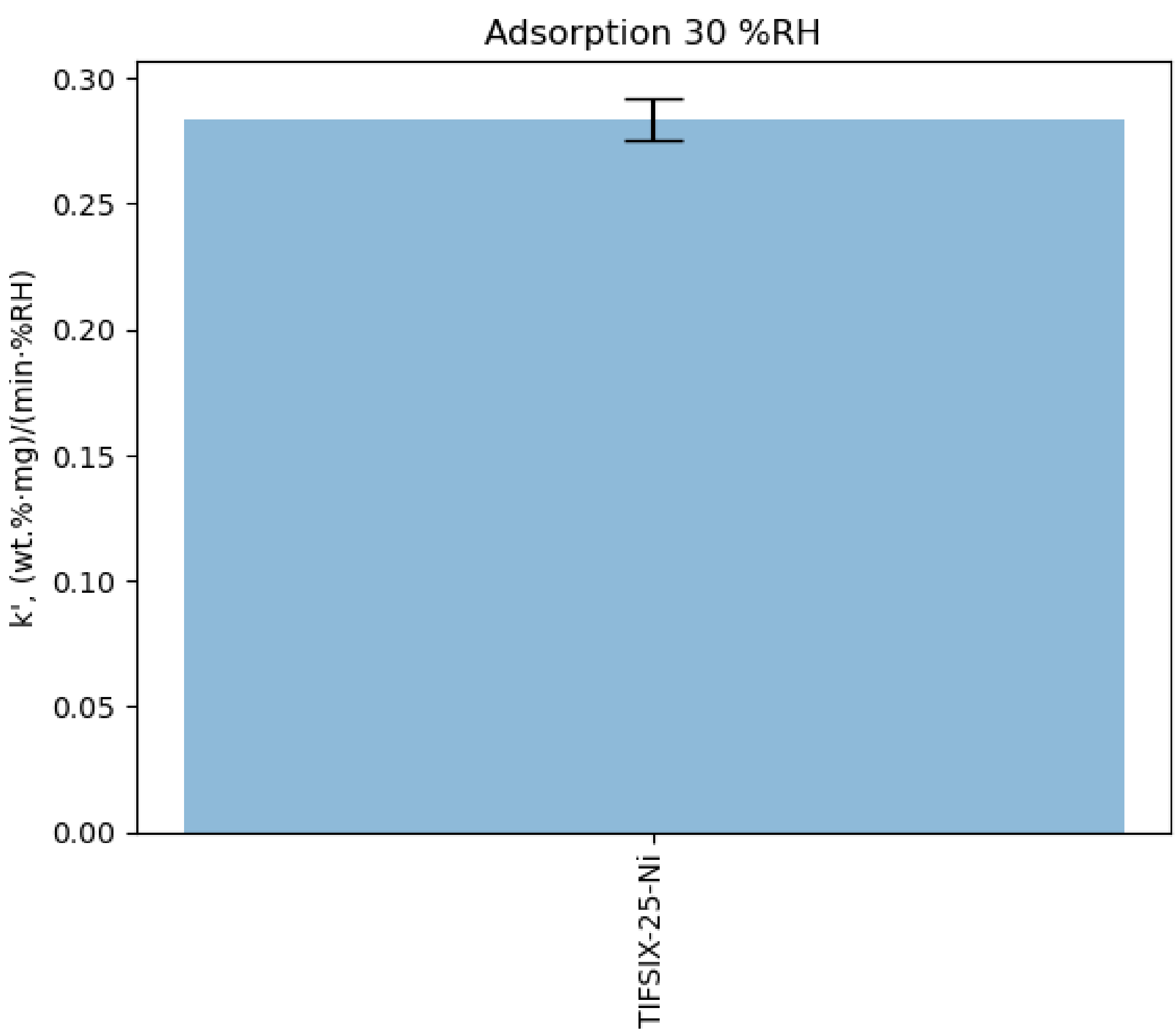

Figure S184 Kinetics coefficient k' in 0-30 %R humidity swing experiments on **TIFSIX-25-Ni** at 27 °C.

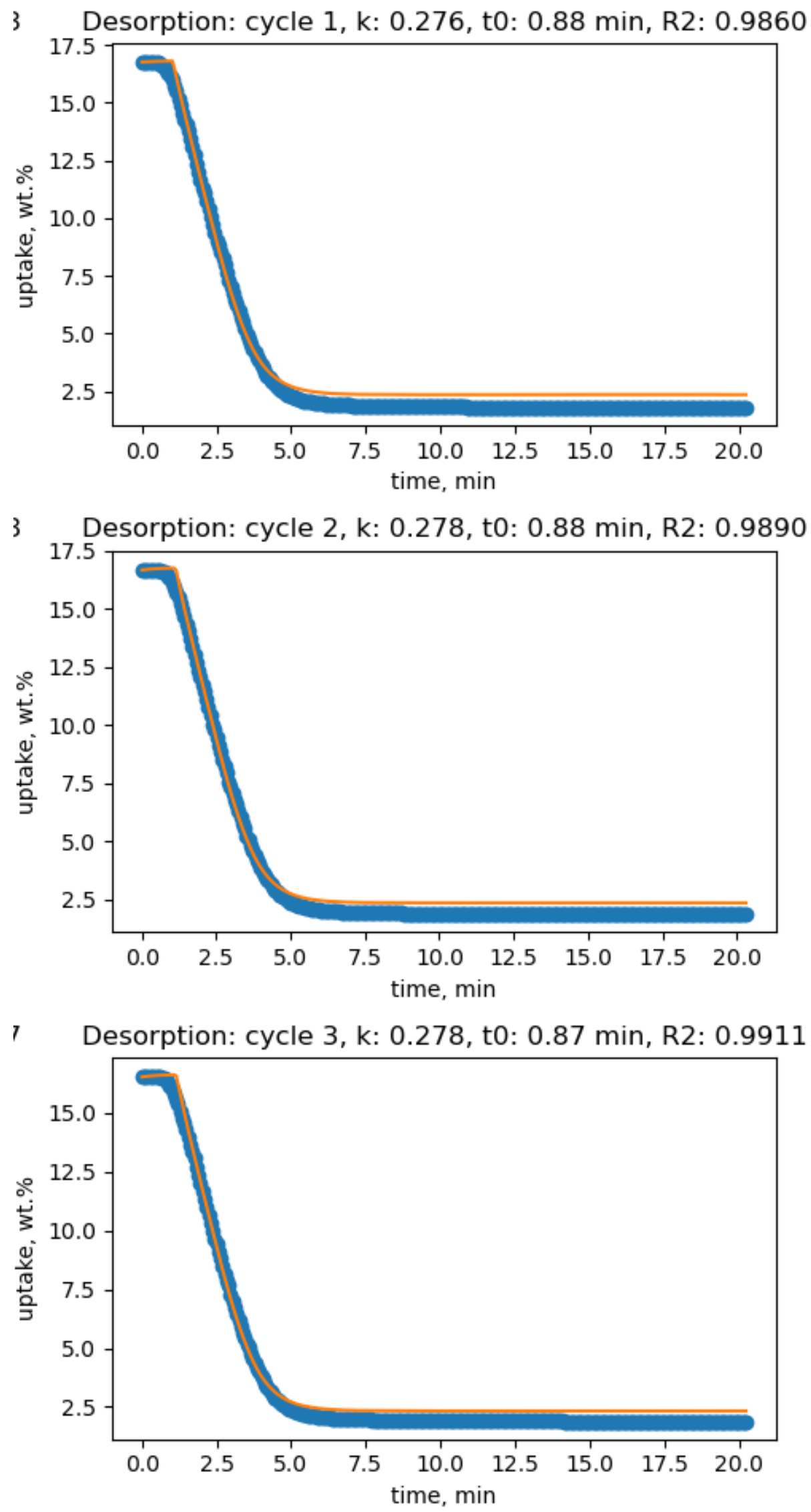

Figure S185 Fit of 30-5.4 %RH humidity swing at 60 °C desorption kinetics data (blue) **TIFSIX-25-Ni** sample (4.8 mg) using extrinsic diffusion model (orange).

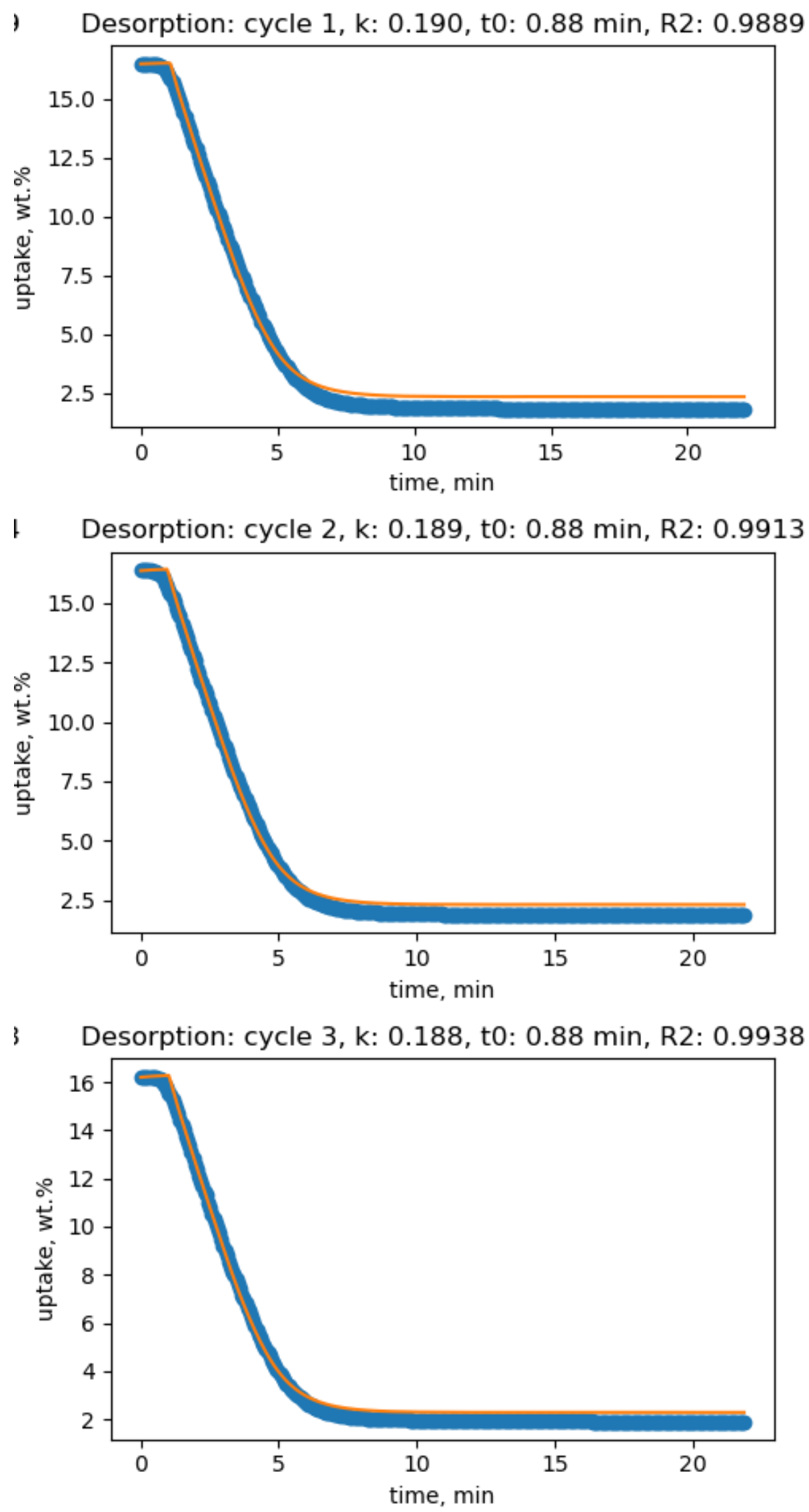

Figure S186 Fit of 30-5.4 %RH humidity swing at 60 °C desorption kinetics data (blue) **TIFSIX-25-Ni** sample (7.3 mg) using extrinsic diffusion model (orange).

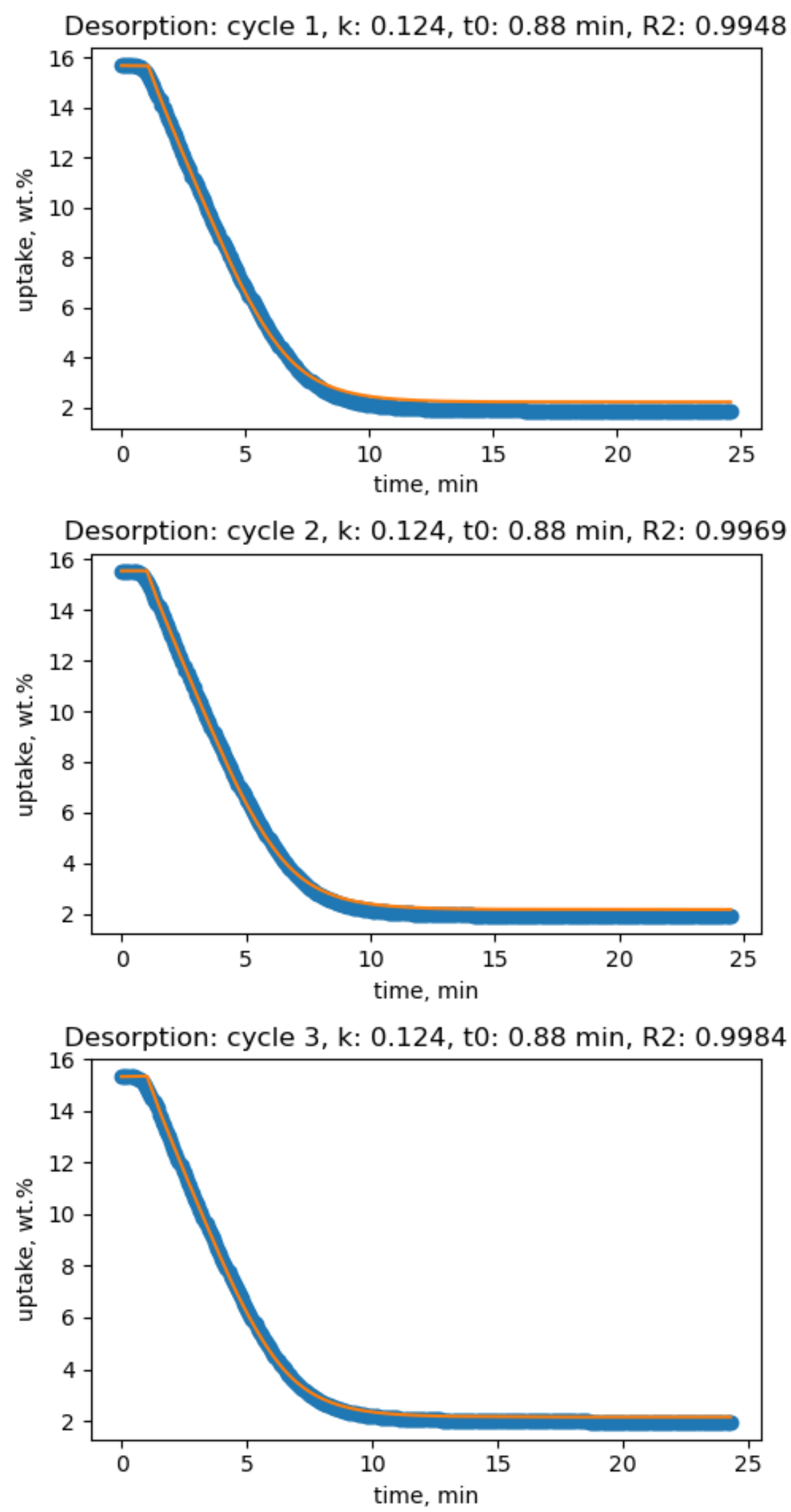

Figure S187 Fit of 30-5.4 %RH humidity swing at 60 °C desorption kinetics data (blue) **TIFSIX-25-Ni** sample (11.5 mg) using extrinsic diffusion model (orange).

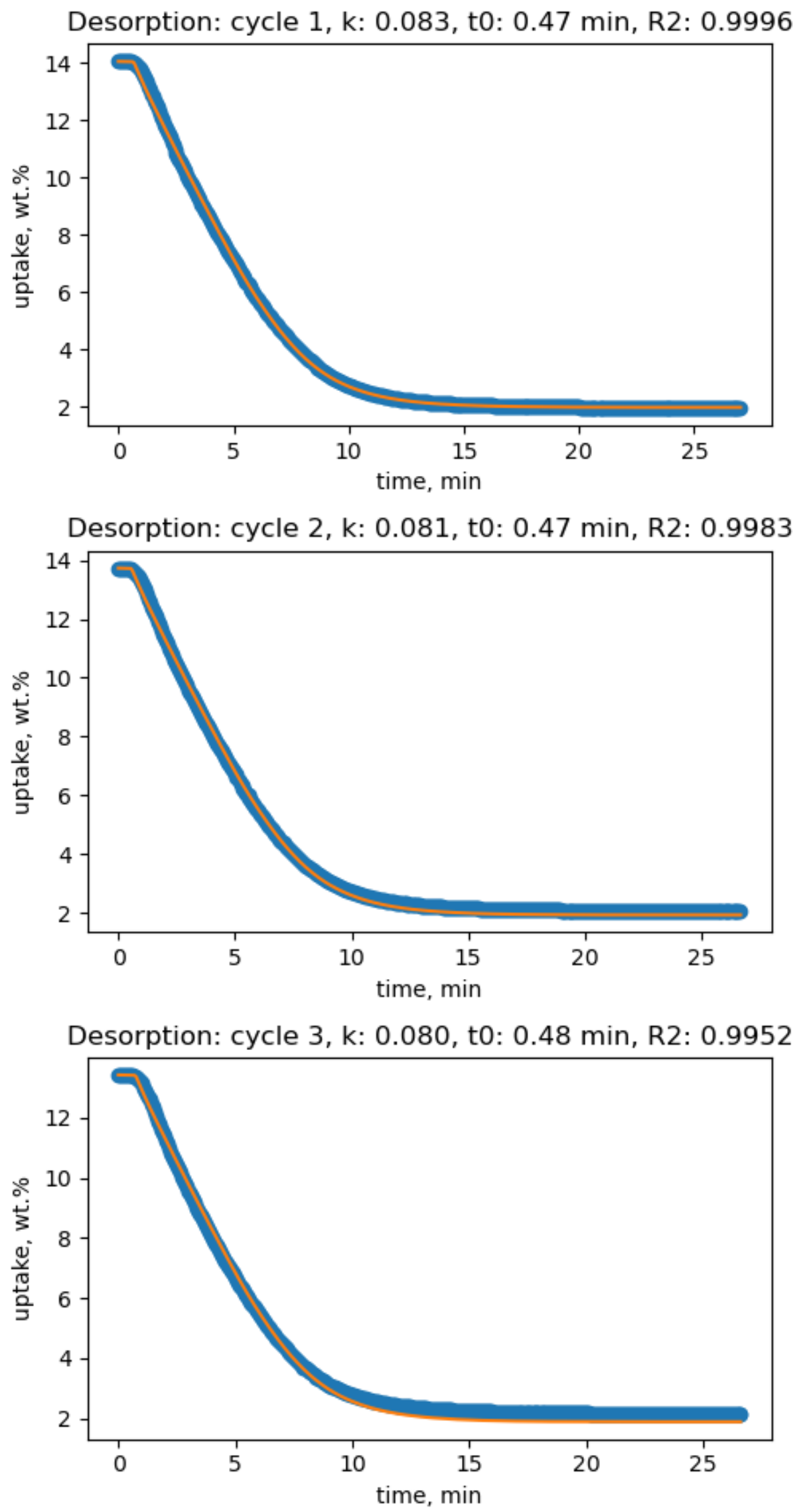

Figure S188 Fit of 30-5.4 %RH humidity swing at 60 °C desorption kinetics data (blue) **TIFSIX-25-Ni** sample (17.9 mg) using extrinsic diffusion model (orange).

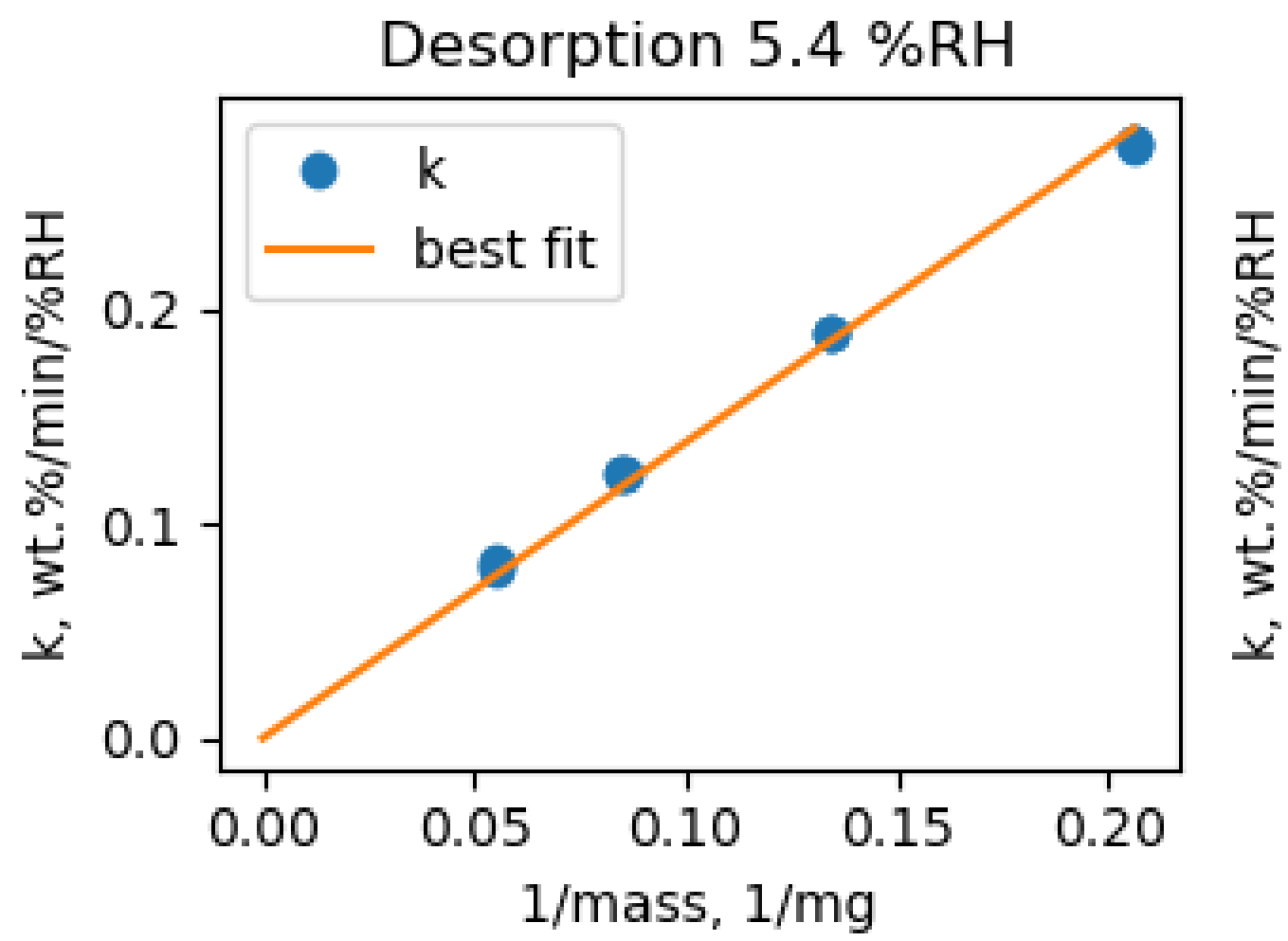

Figure S189 Kinetics coefficient *k* as function of mass of the sample in 30-5.4%RH humidity swing experiments on **TIFSIX-25-Ni** at 60 °C.

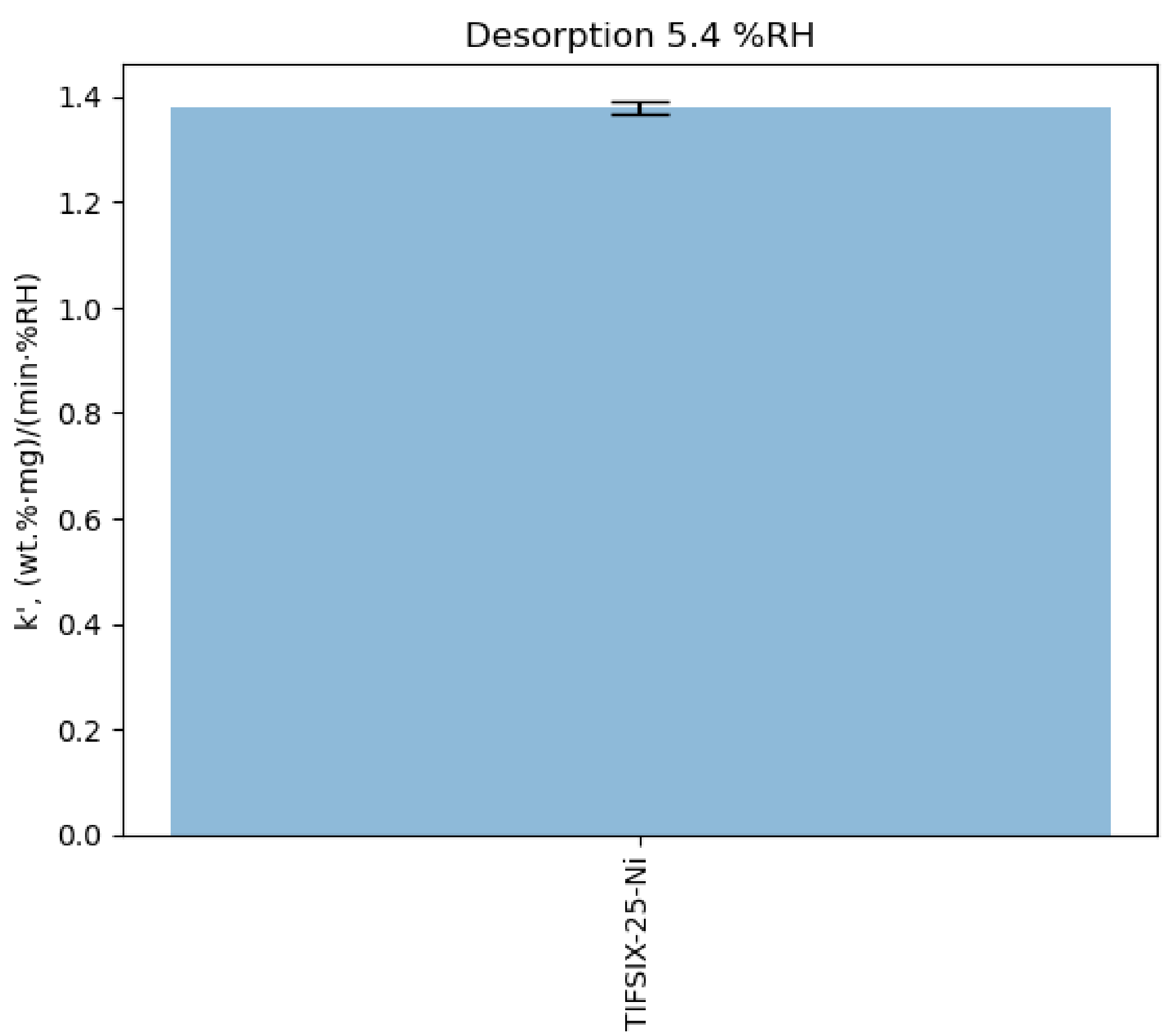

Figure S190 Kinetics coefficient k' in 5.4-30%RH humidity swing experiments on **TIFSIX-25-Ni** at 60 °C.

Table S12 Kinetics coefficients determined from experimental sorption kinetics.

Material	k’	
	Adsorption, 27 C	Desorption, 60 C
TIFSIX-25-Ni	0.284±0.009	1.379±0.013

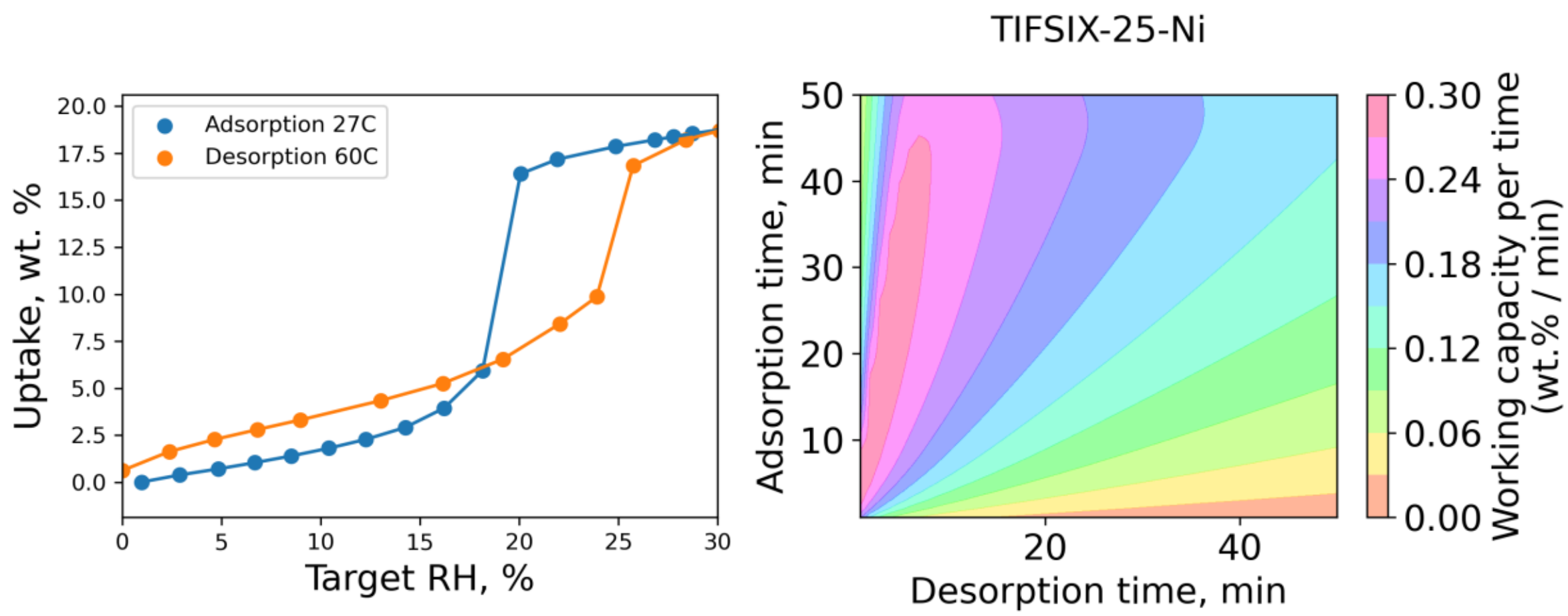

Figure S191 Heatmaps modeled using diffusion to sorbent bed surface limitation model (k experimental, 10 mg sample) predicting sorption productivity for [30% RH, 27°C] – [5.4% RH, 60°C] temperature swing cycling. Performance is calculated per dry mass of the desiccant. Heatmap of **TIFSIX-25-Ni** is reported in current work, heatmaps of other compounds reused from Bezrukov et al.[27]

21 Humidity-swing cycling

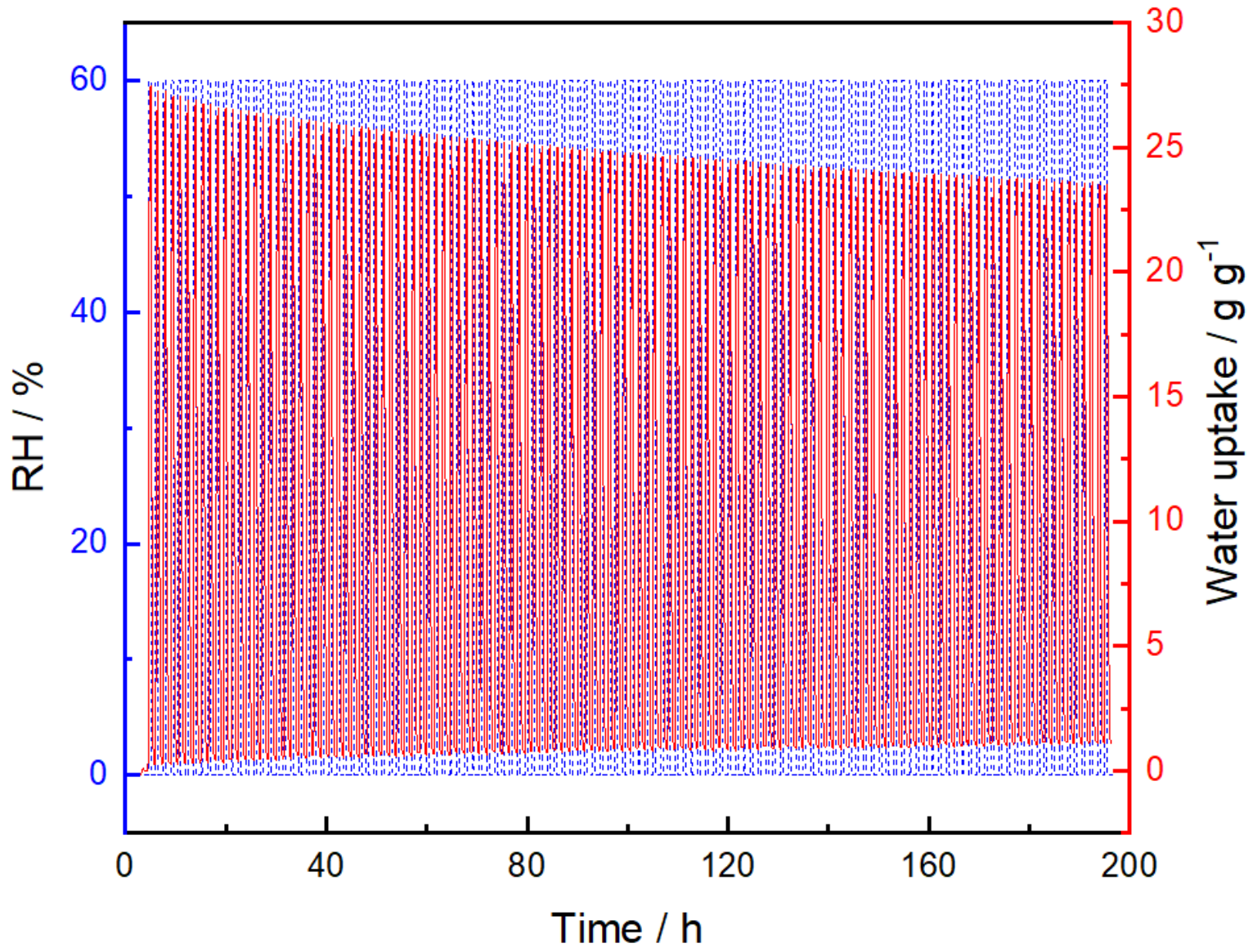

Figure S191 100 consecutive 0–60% RH swing experiments carried out on **TIFSIX-25-Ni** DVS Intrinsic instrument at 25 °C.

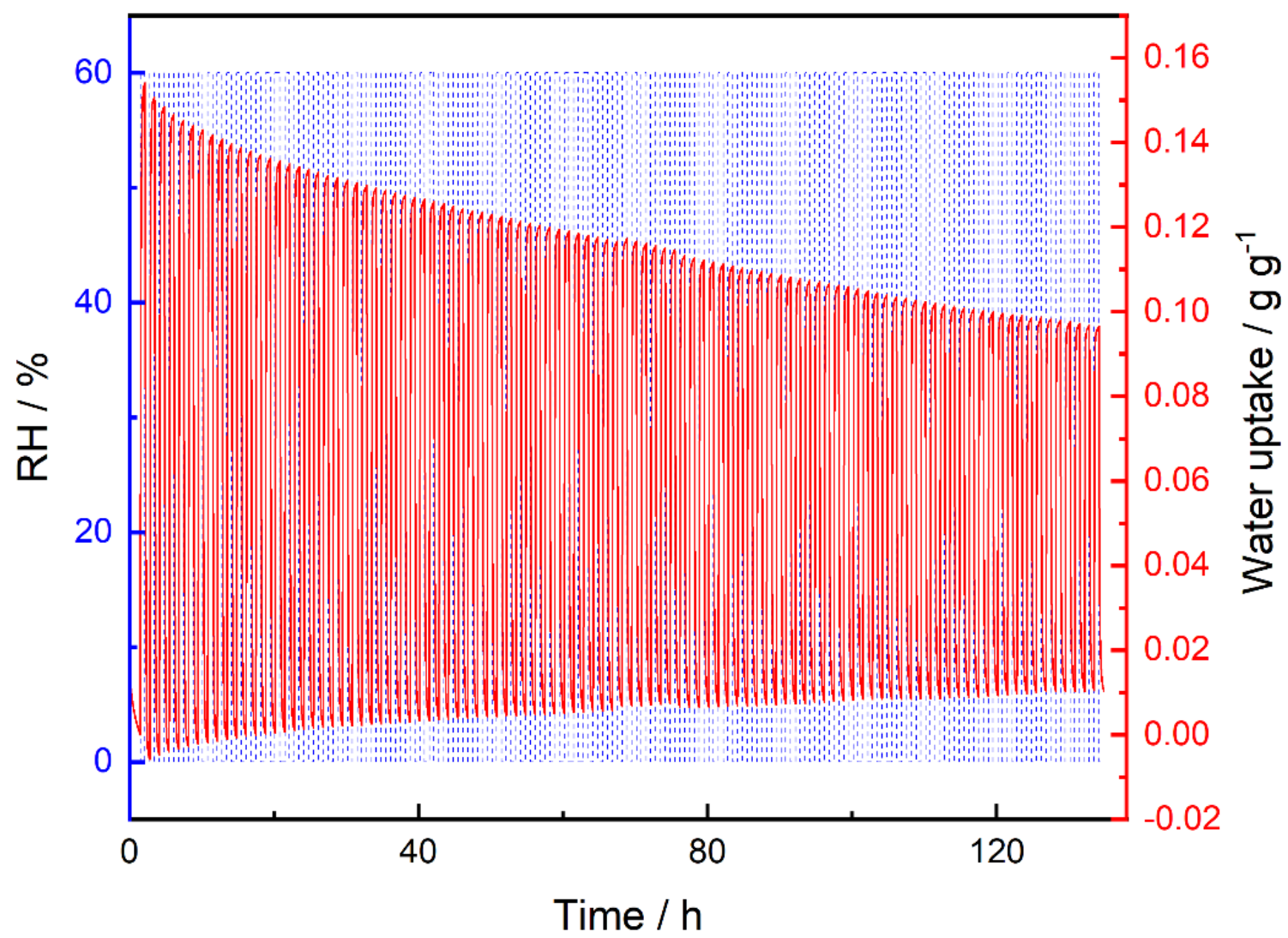

Figure S192 100 consecutive 0–60% RH swing experiments carried out on **TIFSIX-25-Co** DVS Intrinsic instrument at 25 °C.

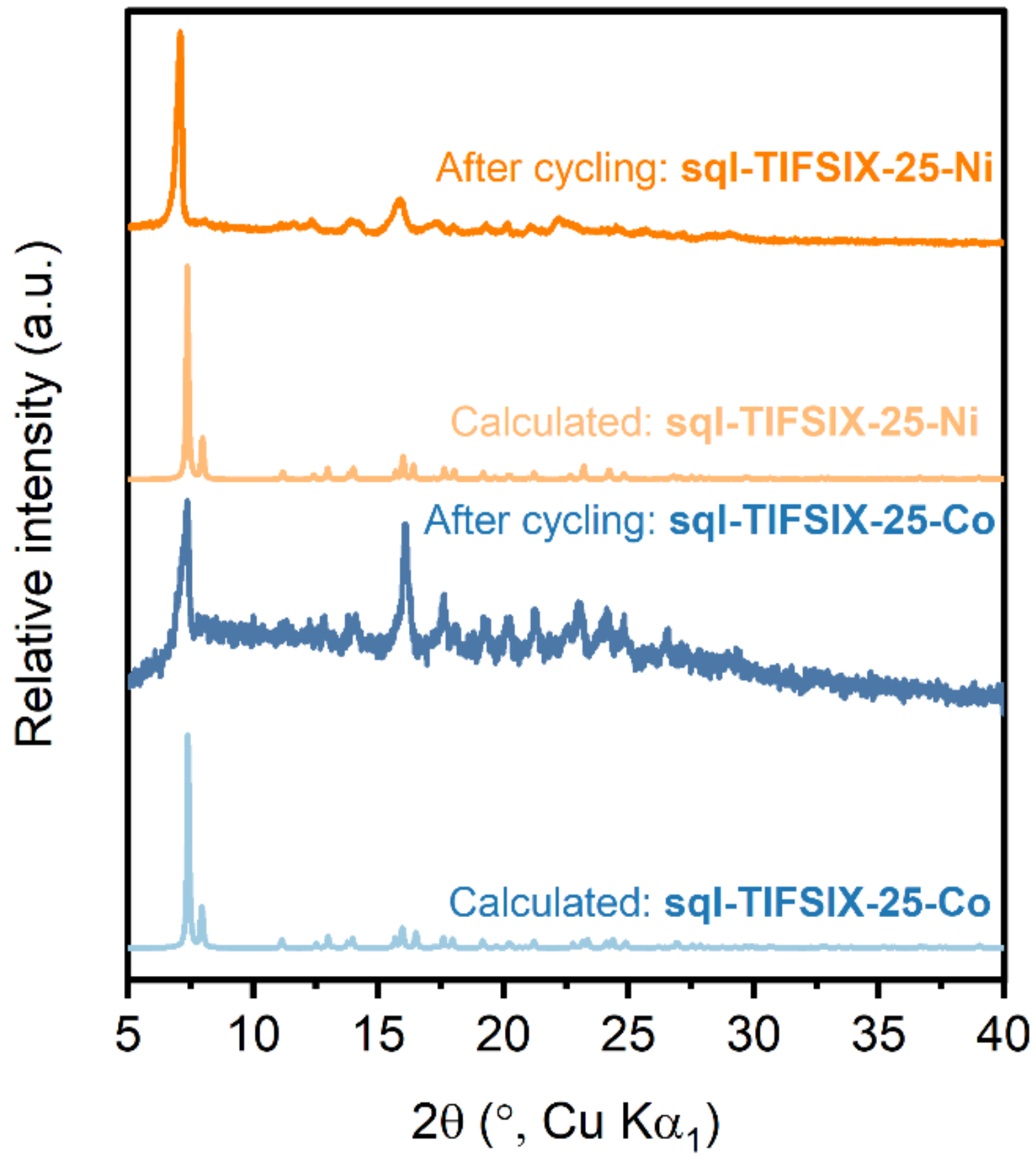

Figure S193 PXRD after RH-swing cycling (0–60% RH, 27 °C).

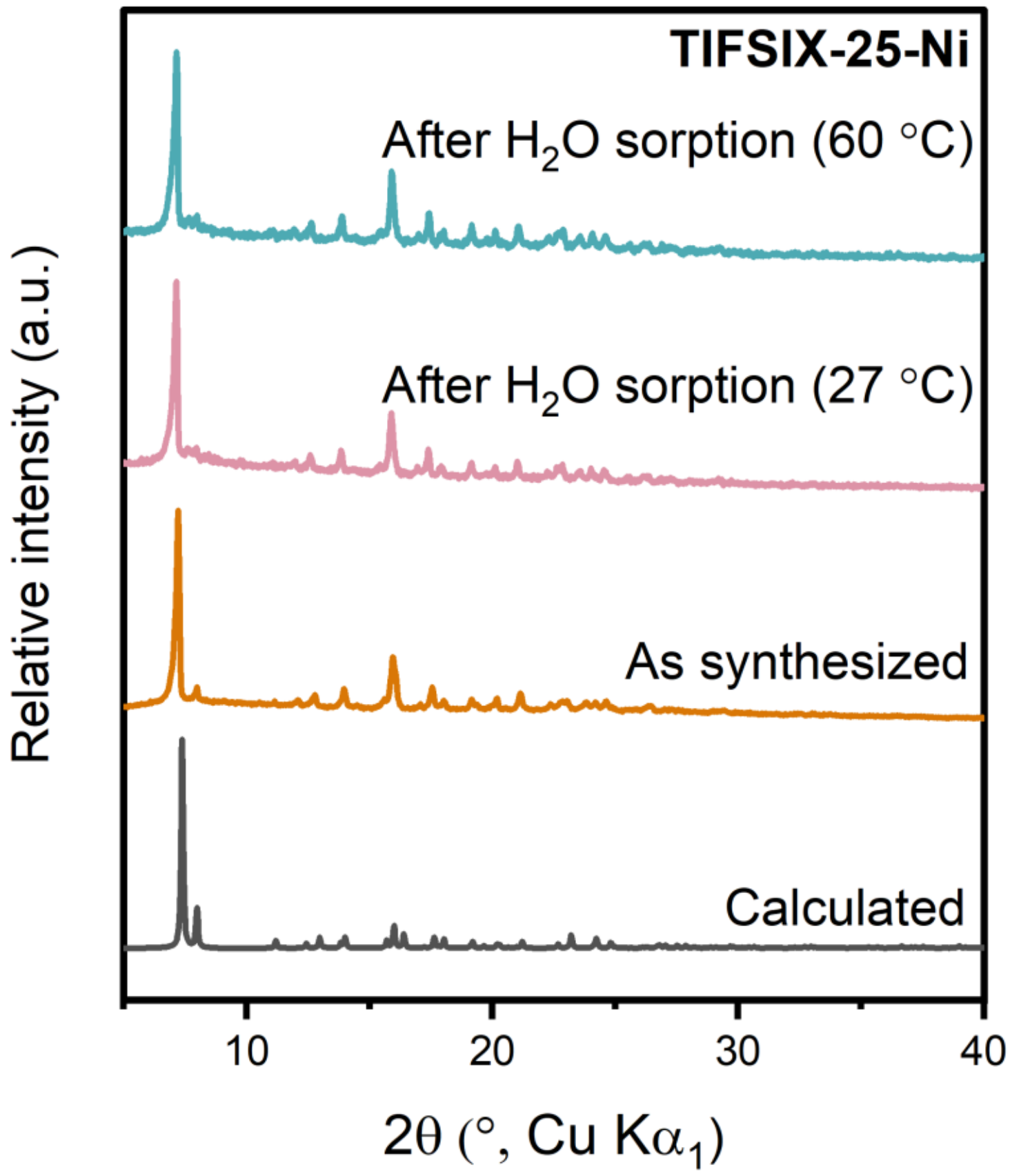

Figure S194 PXRD of **TIFSIX-25-Ni** after H_2O isotherm at different temperatures.

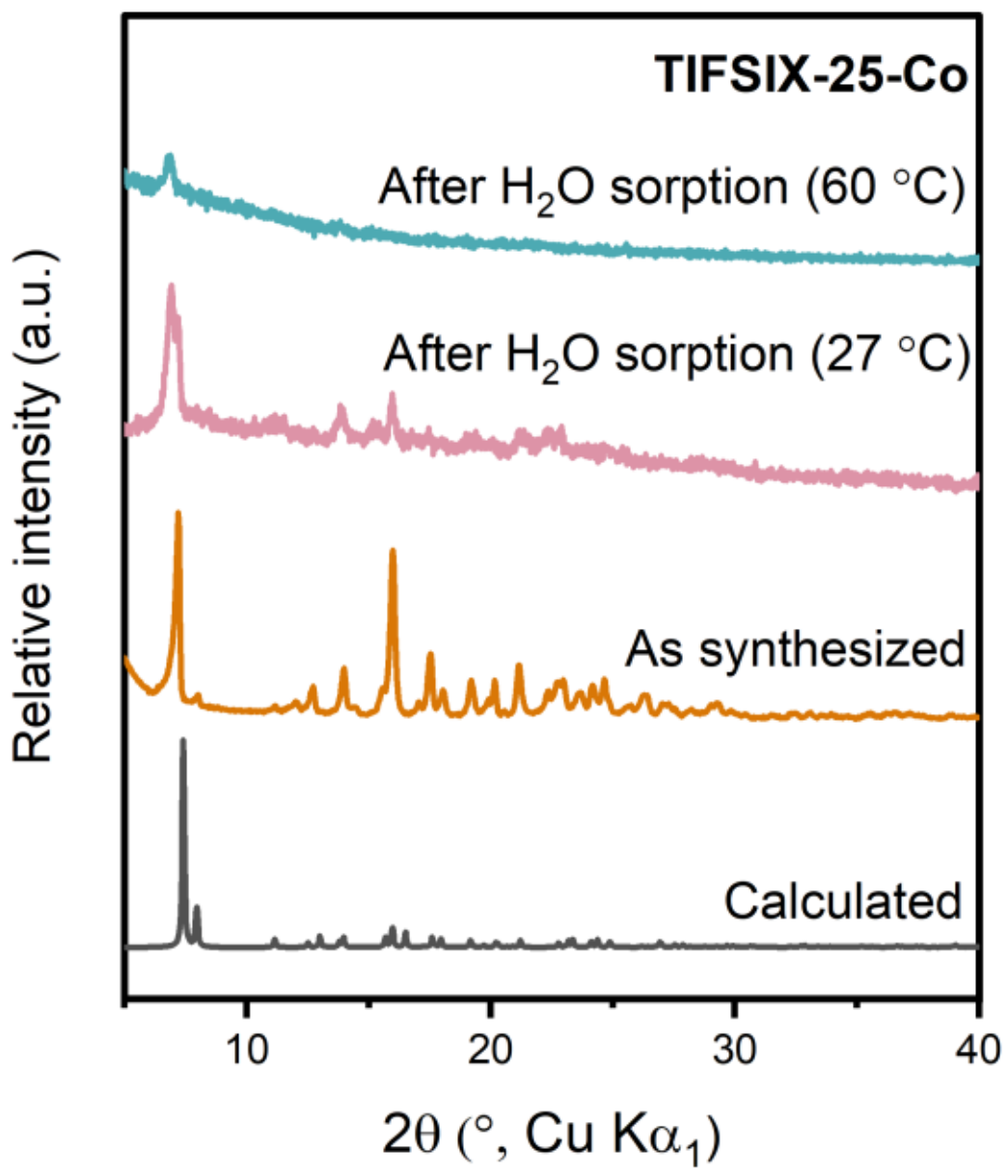

Figure S195 PXRD of **TIFSIX-25-Co** after H_2O isotherm at different temperatures.

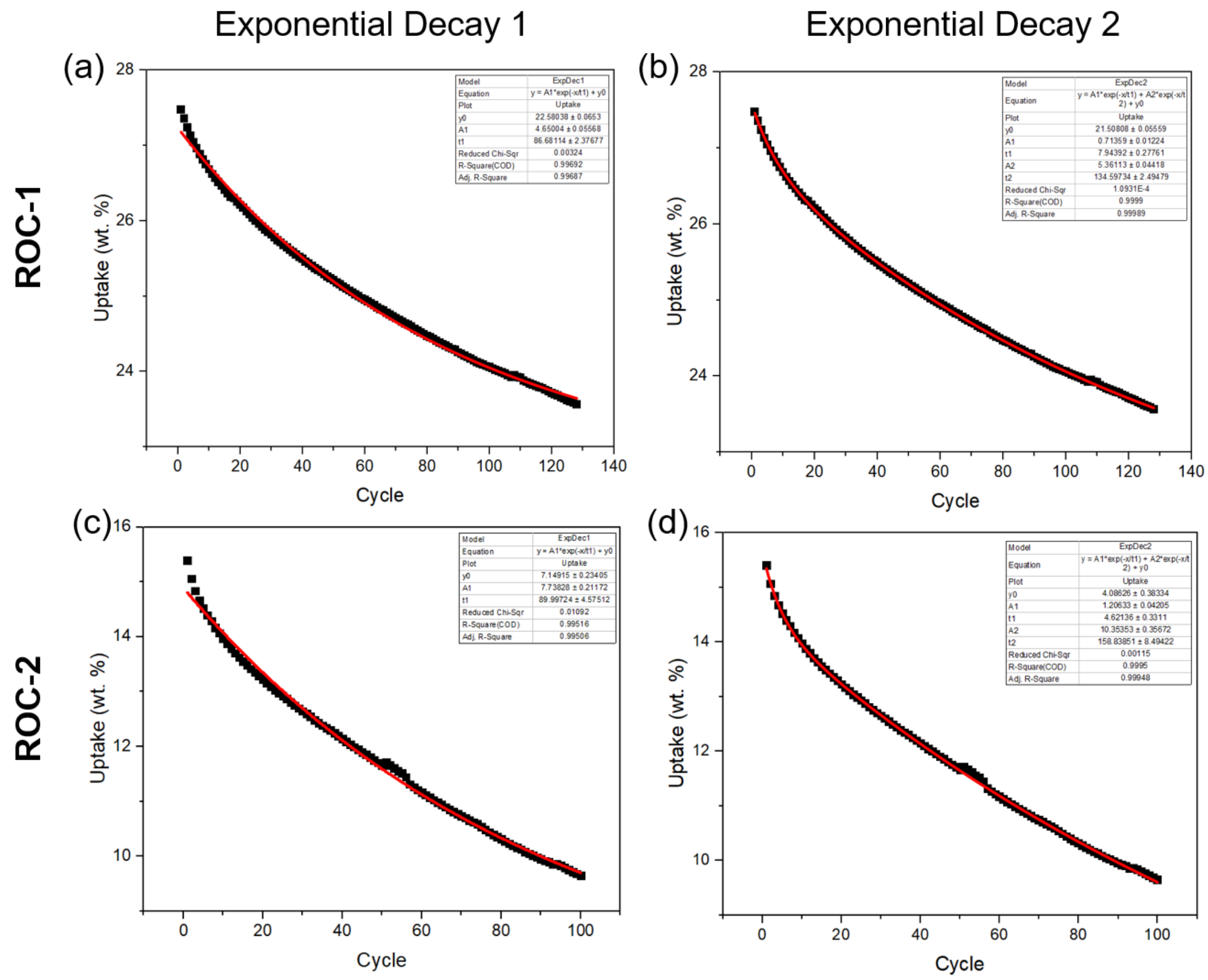

Figure S196 RH-swing cycling data for **ROC-1** (a,b) and **ROC-2** (c,d) fitted with (a,c) single-exponential and (b, d) double-exponential functions.

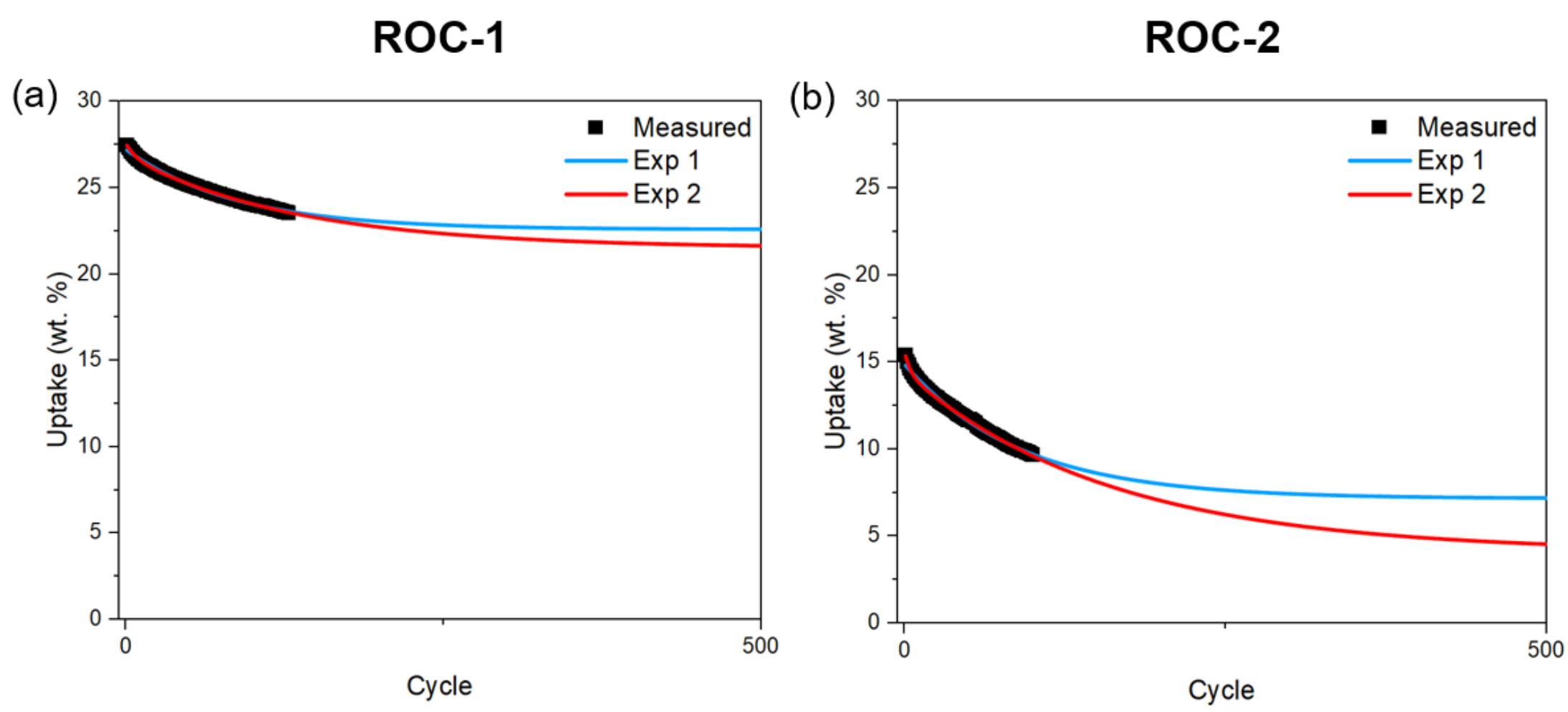

Figure S197 RH-swing cycling data for (a) **ROC-1** and (b) **ROC-2** with sorption performance extrapolated using single-exponential (Exp 1, blue) and double-exponential (Exp 2, red) models.

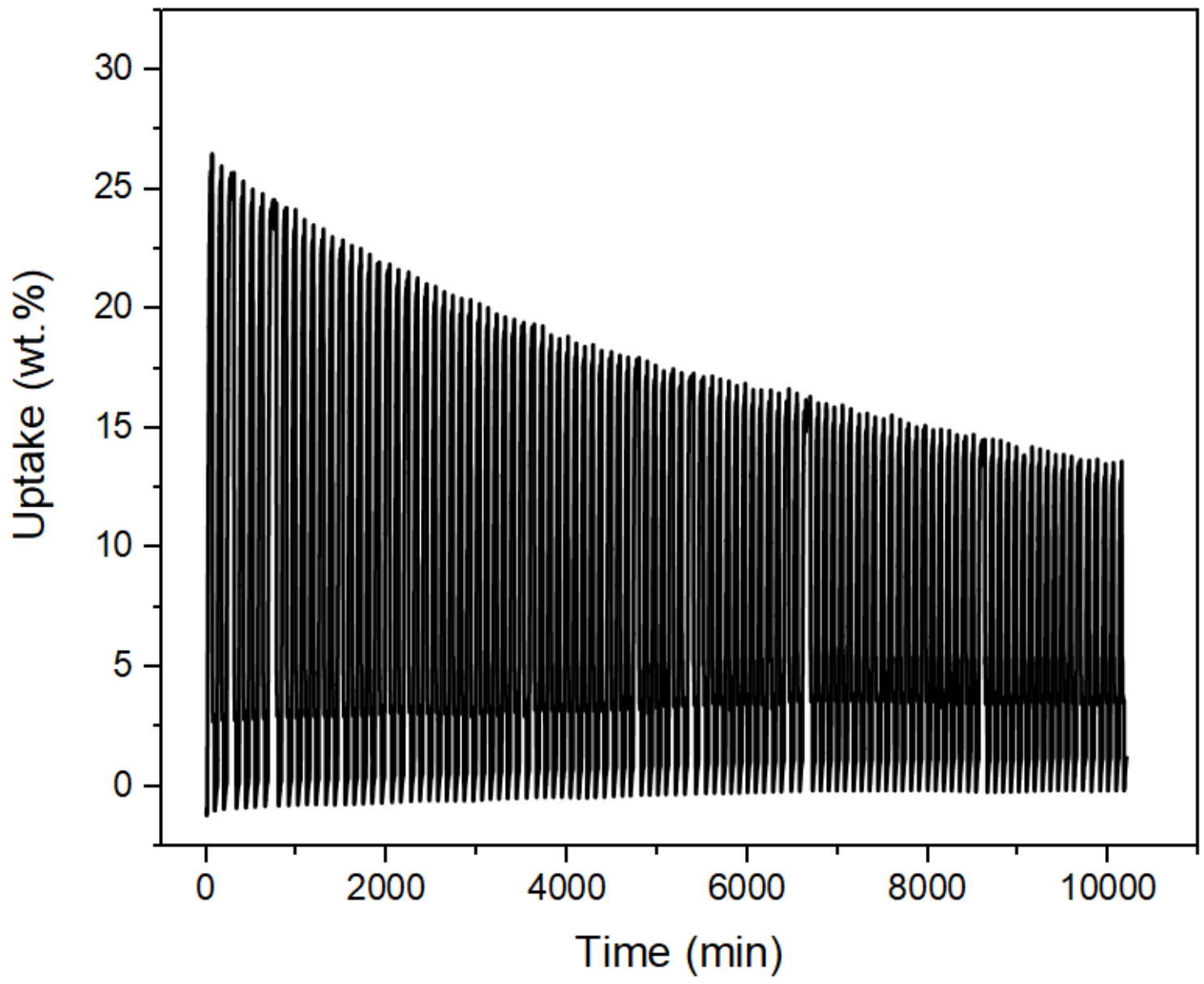

Figure S198 100 consecutive T-swing (loading [30% RH, 27 °C] – unloading [5.4% RH, 60 °C]), experiments carried out on **TIFSIX-25-Ni**.

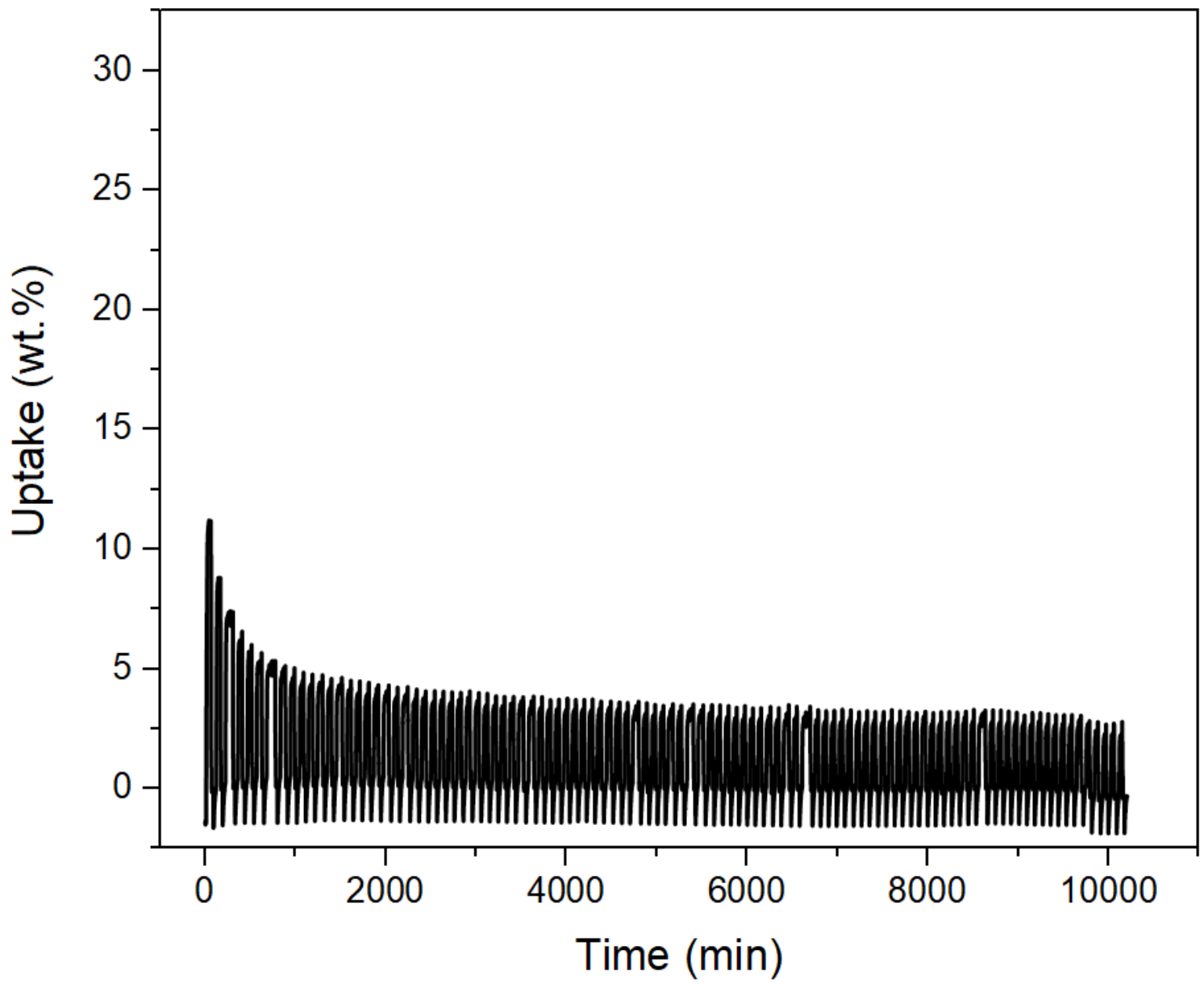

Figure S199 100 consecutive T-swing (loading [30% RH, 27 °C] – unloading [5.4% RH, 60 °C]), experiments carried out on **TIFSIX-25-Co**.

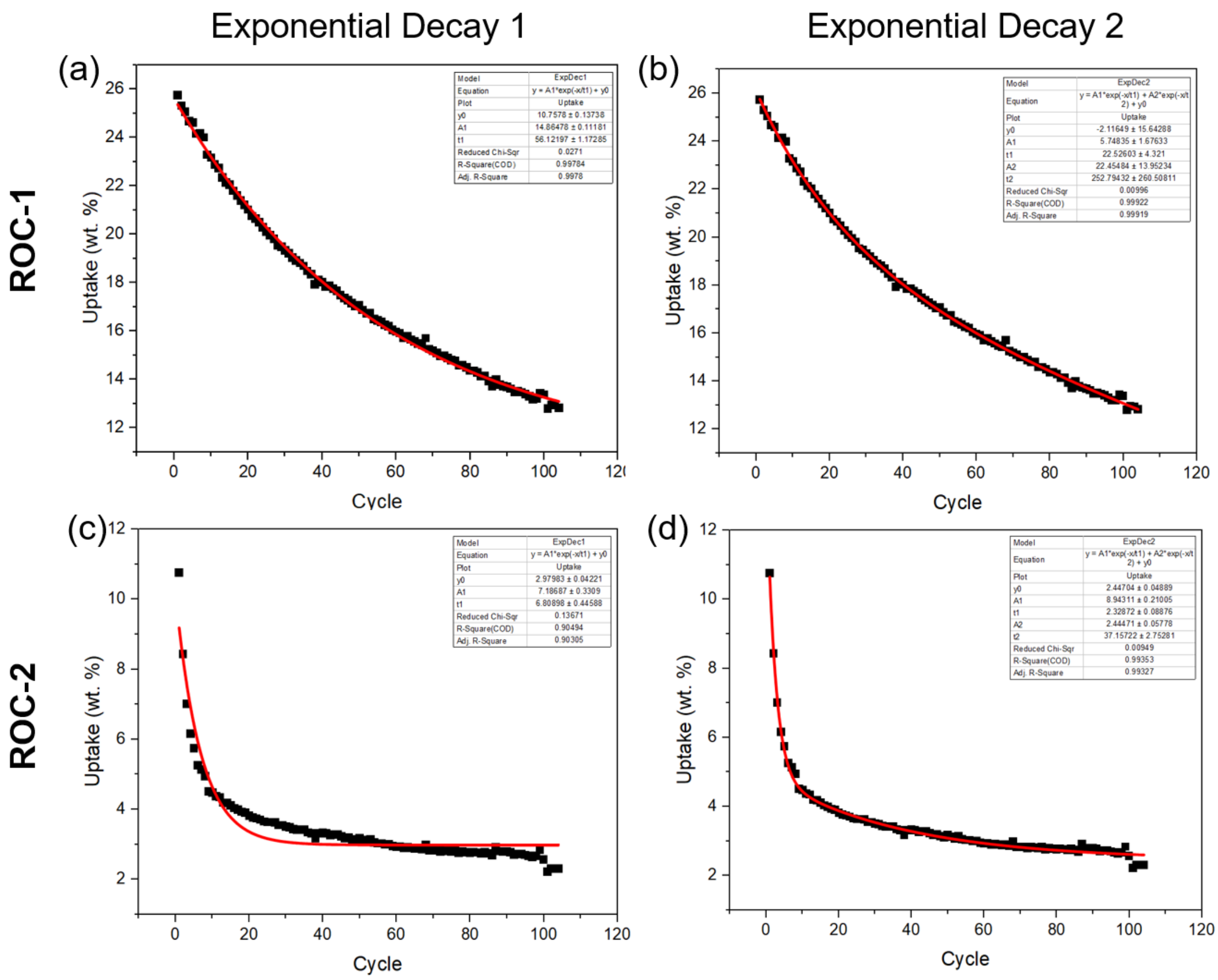

Figure S200 T-swing cycling data (loading [30% RH, 27 °C] – unloading [5.4% RH, 60 °C]) for **ROC-1** (a,b) and **ROC-2** (c,d) fitted with (a,c) single-exponential and (b, d) double-exponential functions.

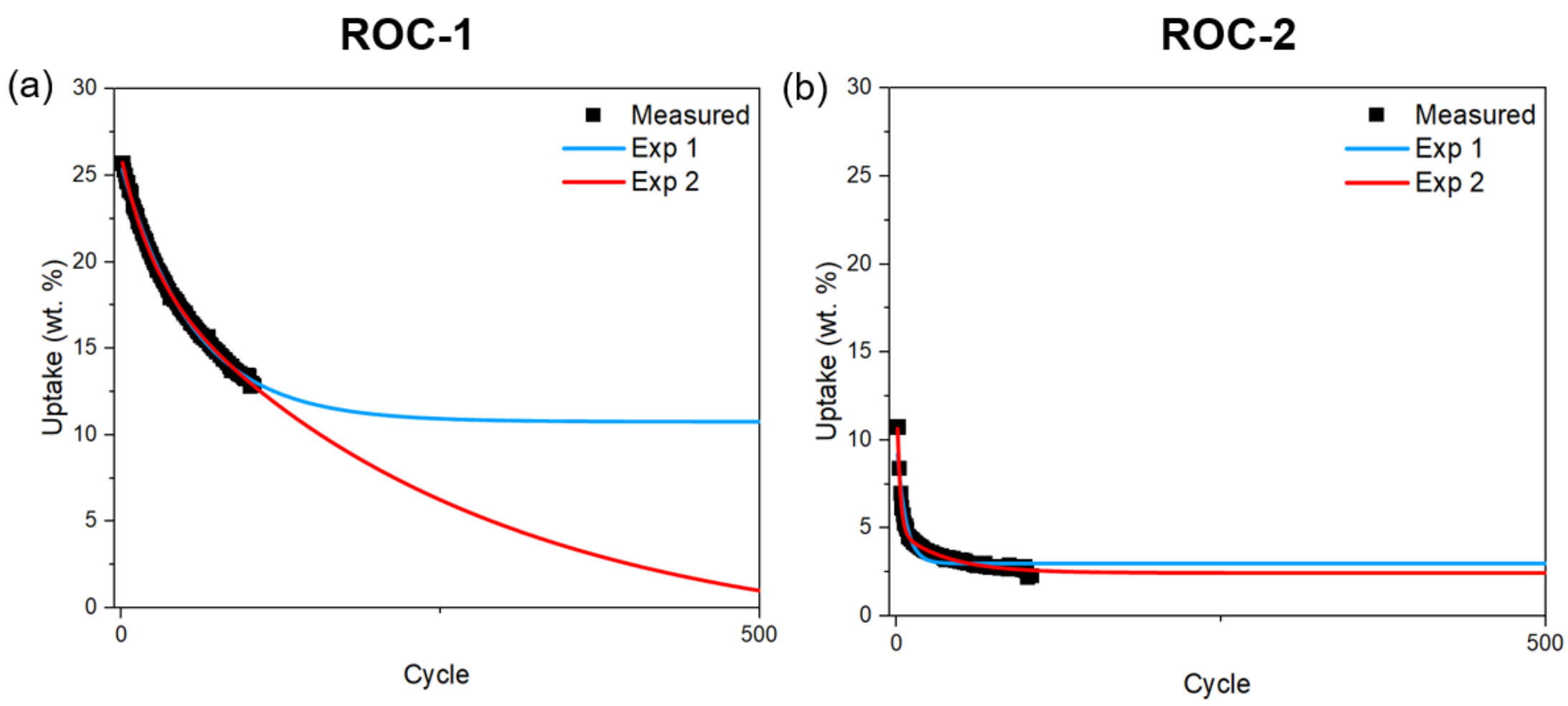

Figure S201 T-swing cycling data (loading [30% RH, 27 °C] – unloading [5.4% RH, 60 °C]) for (a) **ROC-1** and (b) **ROC-2** with sorption performance extrapolated using single-exponential (Exp 1, blue) and double-exponential (Exp 2, red) models.

22 Particle size and morphology

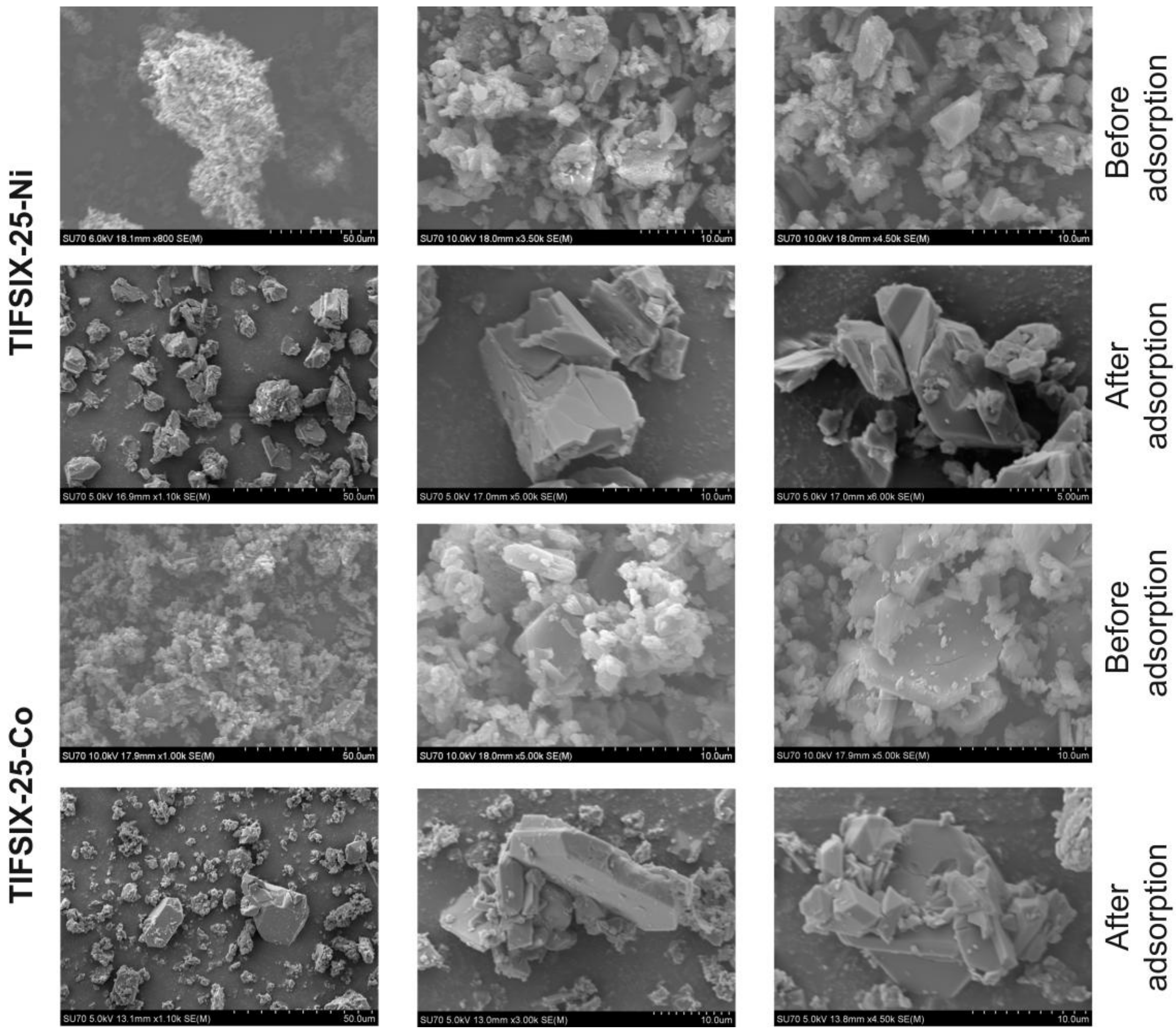

Figure S202 SEM images of **TIFSIX-25-Ni** and **TIFSIX-25-Co** before and after water adsorption measurements. Images acquired at different magnifications reveal that both materials retain their crystalline morphology after water adsorption, while exhibiting a noticeable reduction in particle agglomeration, resulting in more discrete crystallites after the adsorption–desorption cycle.

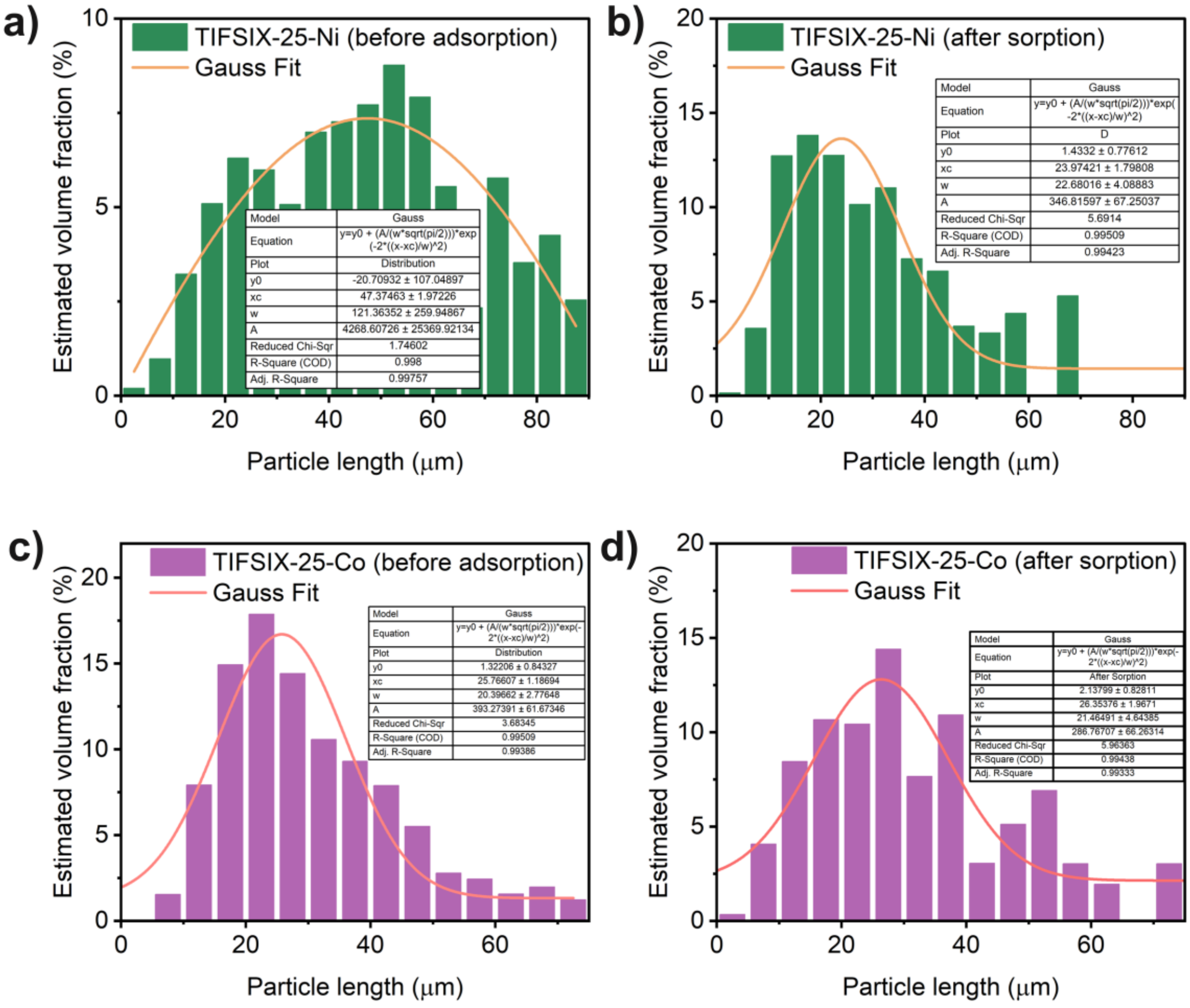

Figure S203 Particle length distributions of (a) **TIFSIX-25-Ni** before water adsorption, (b) **TIFSIX-25-Ni** after water adsorption, (c) **TIFSIX-25-Co** before water adsorption, and (d) **TIFSIX-25-Co** after water adsorption, obtained from automated image analysis of 5000 particles. Histograms show the estimated volume fraction as a function of particle length, and the solid curves represent Gaussian fits to the experimental distributions. The shift toward smaller particle lengths after water adsorption is consistent with the reduced agglomeration observed in the SEM images.

23 Computational modelling

23.1 Unit Cell Preparation

Thirteen unit cells are directly from crystal structures by SCXRD: **M'FSIX-25-Co** (Table S2), **M'FSIX-25-Ni** (Table S3), **pcu-M’FSIX-25-Cu** (Table S4), **pcu-SIFSIX-3-Ni** (CSD Refcode DUMFEY) and **HKUST-1** (CSD Refcodes: hydrated, FIQCEN; dehydrated, FUNGED). Three additional unit cells, **sql-SIFSIX-25-Cu, sql-TIFSIX-25-Cu** and **sql-SIFSIX-3-Ni**, were constructed for DFT. For **sql-M’FSIX-25-Cu**, unit cells were constructed from the **sql-M’FSIX-25-Co** structures, where Co was replaced with Cu. The **sql-SIFSIX-3-Ni** structure was constructed from [Cu(HF_2)(pyz)2]TaF_6 (CSD Refcode NATYAK[37]) by removing HF_2, and substituting Cu for Ni and Ta for Si.

23.2 Periodic Density Functional Theory (DFT) Optimizations

All calculations were carried out using the CP2K[38] software package, employing mixed Gaussian and plane wave basis sets[39]. Core electrons were represented with norm-conserving Goedecker-Teter-Hutter pseudopotentials[40], and the valence electron wavefunction was expanded in a triple-zeta basis set with polarization functions, TZVP-MOLOPT-SR-GTH for metals and TZVP-MOLOPT-GTH for non-metals[41]. Auxiliary plane wave basis sets were employed with an energy cutoff of 950 Ry and a relative cutoff of 70 Ry. All systems were described using the generalized gradient approximation exchange-correlation functional of Perdew, Burke, and Ernzerhof (PBE), with dispersion interactions accounted for using the DFT-D3 scheme[42-43]. Geometry and cell optimizations were performed using the BFGS algorithm with SCF convergence criteria of 1.0×10^{-6} a.u. All twelve members of the **M'FSIX-25-M** family underwent cell optimizations with periodic boundary conditions.

23.3 Pore Analyzer for Measuring PLD

Pore limiting diameter (PLD) of each structure was measured using the Pore Analyser feature of Mercury,[8] Version 2024.2.0, 2.58 Å helium probe sigma, 298 K temperature, 3.314 Å of nitrogen probe sigma, 500 samples per atom for the surface area calculation, 10.22 K helium probe epsilon, 12.8 Å cutoff distance, and 0.2 Å cubelet size.

23.4 Design of Computational Procedures

Hereafter, the term "hydrate," symbolized as α, will refer to **sql-M'FSIX-L-M** systems and the hydrous phase of **HKUST-1**, the latter of which serves as a benchmark for subsequent calculations. To distinguish hydrated phases from one another, we use W to denote the number of water molecules coordinated to each metal center. For example, in the hydrate of **HKUST-1**, one water molecule is bound to each copper center, giving W = 1. For **sql-M'FSIX-L-M**, two water molecules are bound to each metal center, giving W = 2. The anhydrous phase of all systems is designated as β (W = 0), which includes **pcu-M'FSIX-L-M**. This is further illustrated in Figure S204.

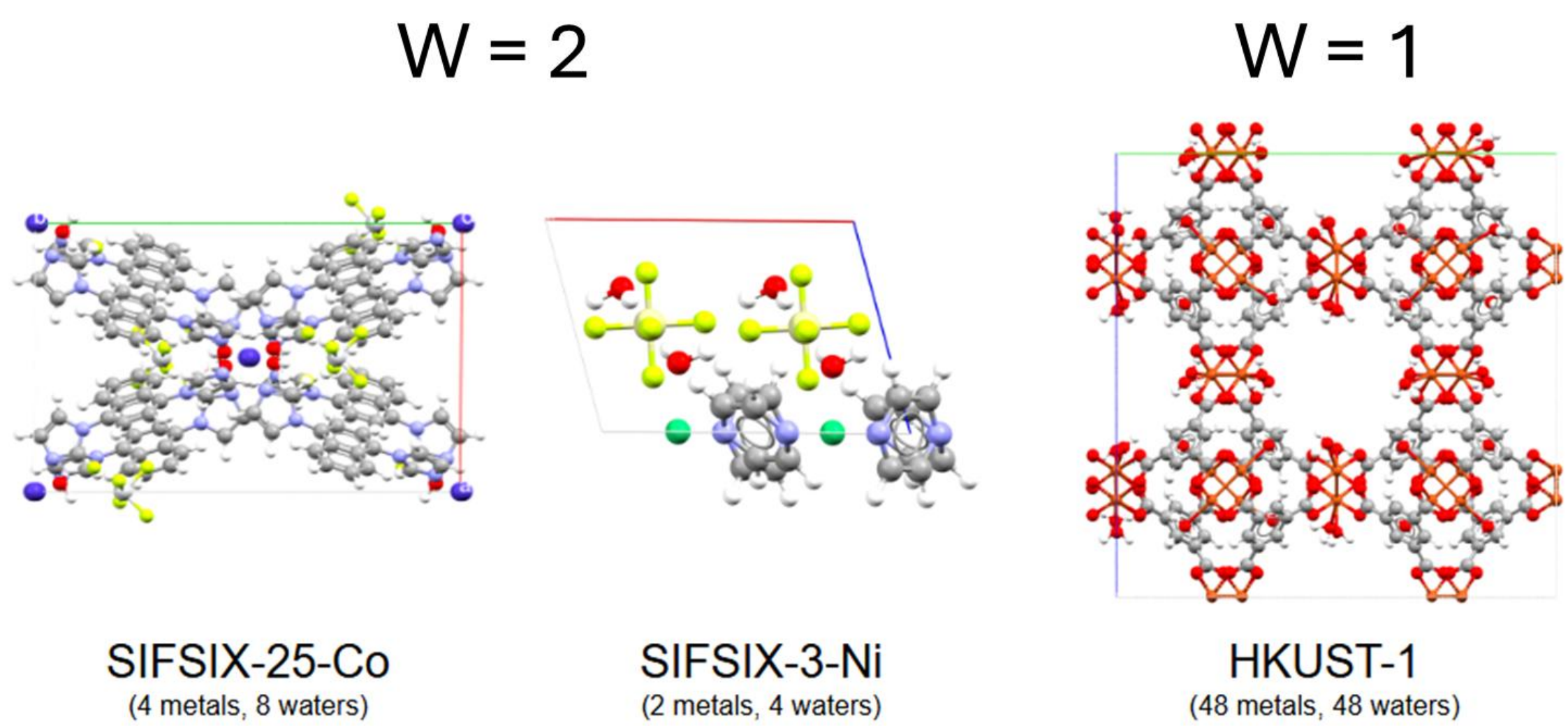

Figure S204 An illustration of the *W* as classification of the hydrous (α) phases, where *W* is the number of waters coordinated with each metal in the material. The unit cells labeled as **SIFSIX-25-Co** and **SIFSIX-3-Ni** are **sql** (W = 2). The unit cell labeled as **HKUST-1** is the hydrated MOF with a single water molecule coordinated to each metal (*W* = 1).

23.4.1 Phase Transition Energy Calculations

The **M'FSIX-25-M** family undergoes a series of steps throughout its phase transition. To understand the relative stability of the hydrated and anhydrous phases, the energetic process was broken down into steps as shown in Figure S205. Most materials in this study can reversibly perform phase transitions in both directions (α → β and β → α), and the following decomposition

was adapted from a previously reported approach[44] to capture these essential chemical processes.

In the α → β phase transition, α first undergoes breaking of the metal-water and anion-water bonds, releasing water vapor (Figure S205a). A hypothetical intermediate, α_I, is then created by deleting the water molecules on either side of the metal center in α. To capture the energetic contribution of forming the metal-anion bonds (Figure S205b), the energy of the cell-optimized β is compared to that of α_I. Note that α_I has an identical chemical composition to β but is structurally distinct. The α_I phase is purely theoretical, serving only as an energetic reference to isolate the individual contributions of metal-water, anion-water, and metal-anion bonding to the overall phase transition energy.

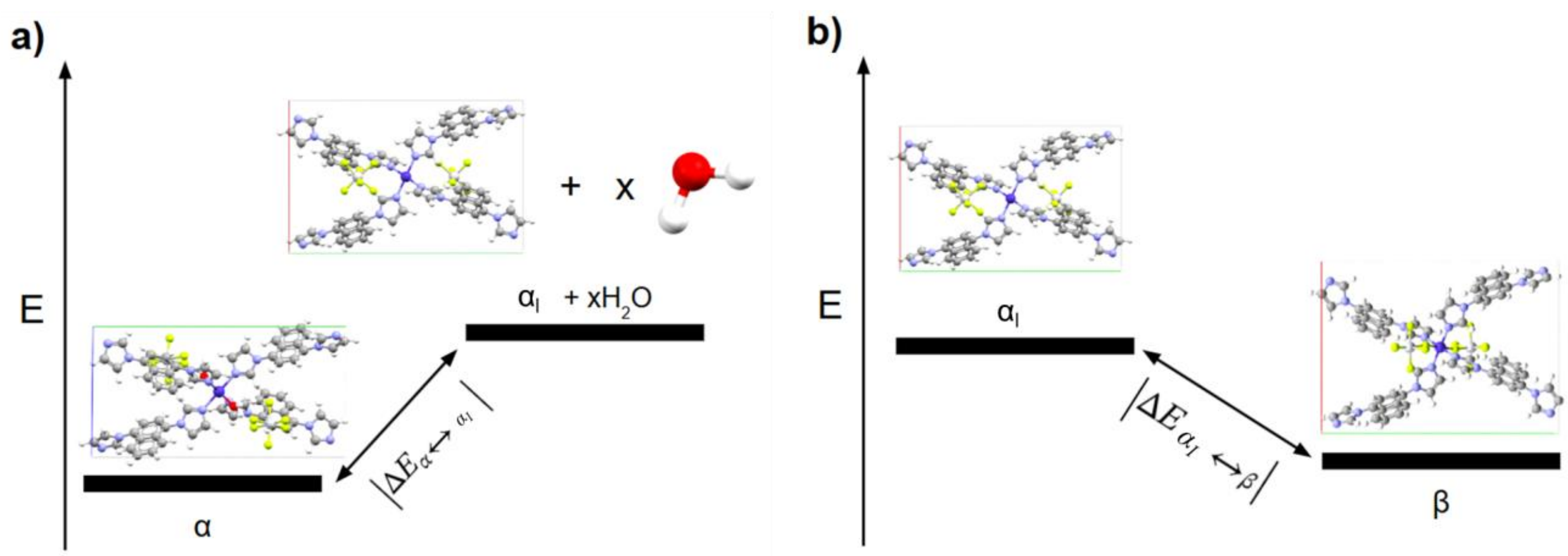

Figure S205 An illustration of the energetic decomposition scheme for the $\alpha \leftrightarrow \beta$ phase transition using the **SIFSIX-25-Co** structure as an example. Panel (a) shows the energy difference $|\Delta E_{\alpha\leftrightarrow\alpha_I}|$ between α and α_I, where the vaporization or re-absorption of x water molecules from the gas phase is accounted for. Panel (b) shows the energy difference $|\Delta E_{\alpha_I\leftrightarrow\beta}|$ between α_I and β. Atom colors: white: hydrogen, gray: carbon, blue: nitrogen, red: oxygen, yellow: fluorine, beige: silicon, purple: cobalt.

Four configurations are calculated to construct the phase transition coordinate: the α phase, the hypothetical α_I intermediate, the H_2O vapor phase, and the β phase.

The α phase energy is obtained by relaxing the experimental single-crystal structure via DFT cell optimization as described above. The α_I intermediate is derived from the resulting α structure by deleting all water molecules and recalculating the energy using a single-point energy calculation

(ENERGY job type), without allowing the structure to relax. The H_2O vapor energy is obtained by inserting a single water molecule into an empty unit cell of the same dimensions as the α cell, followed by geometry optimization. The β phase energy is obtained analogously to α via DFT cell optimization of the experimental structure. However, because the β unit cell differs in size from α, a second single-point energy calculation is performed on the cell-optimized β structure with the CELL_REF option set to the unit cell parameters of α, enabling direct energetic comparison between phases.

These four energetic points are combined in Eqn S5 to give the total phase transition energy:

$$\left|\Delta E_{\alpha\leftrightarrow\beta}\right| = \left|\frac{\left(E_{\beta} + xE_{H_2O}\right) - E_{\alpha}}{M}\right| \qquad S5$$

where x is the number of water molecules per unit cell, E_{H_2O} is the energy of a single water molecule in the vapor phase, and M is the number of metal centers per unit cell. Normalization by M gives the energy contribution per metal center. Note that $W = x/M$.

Eqn S4 can be decomposed into two opposing contributions, as shown in Eqn S6 and S7. The first (Eqn S6) captures the energy cost of breaking metal-water and anion-water bonds and releasing water vapor. The second (Eqn S7) captures the energy gained from structural relaxation of α_I into β. Together, Eqn S6-S8 follow a Hess's law-like path independence.

$$\left|\Delta E_{\alpha\leftrightarrow\alpha_I}\right| = \left|\frac{\left(E_{\alpha_I} + xE_{H_2O}\right) - E_{\alpha}}{M}\right| \qquad S6$$

$$\left|\Delta E_{\alpha_I\leftrightarrow\beta}\right| = \left|\frac{E_{\beta} - E_{\alpha_I}}{M}\right| \qquad S7$$

To validate this methodology, **HKUST-1** was used as a benchmark. It is a rigid system with coordinated waters at open metal sites (W=1), analogous to the **M'FSIX-L-M** family, but lacks the anion-water interactions present in the latter. TG/DSC measurements by Henninger et al.[45] give a coordinated water-metal bond energy of 50.7 ± 2.9 kJ mol⁻¹ for **HKUST-1**. Applying Eqn S6

yields a metal-water bond energy of 53.76 kJ mol^{-1}, while Eqn S7 gives a structural relaxation energy of just 0.05 kJ mol^{-1}, consistent with the rigidity of **HKUST-1**. The resulting total phase transition energy from Eqn S8 is 53.71 kJ mol^{-1}, a 5.77% deviation from the experimental value, validating the approach.

23.4.2 TGA and Pore Limiting Diameter for Kinetics

Molecular dynamics (MD) simulations were initially explored for modelling the kinetics of these systems. However, temperature ramps spanning 0-5000K did not yield mechanistic insights, likely due to the size and complexity of the systems involved. Alternative methods were therefore investigated that could simultaneously provide kinetic information and structure-property relationships. One key factor that may kinetically hinder water evacuation at the designated activation temperatures is the pore limiting diameter (PLD), a measure of the narrowest pore aperture in the system commonly used to estimate the diffusion accessibility of a sorbate. The change in PLD between phases (ΔPLD) is proposed as a structural proxy for the energy input required for the phase transition. ΔPLD is calculated according to Eqn S8.

$$\Delta PLD = PLD_{\beta} - PLD_{\alpha} \qquad S8$$

23.4.2.1 Extractions from TGA Data

Completion temperatures were extracted from experimental TGA data in Figures S206 and S207 and are reported in Table S12. Unlike the onset temperature, which corresponds to the temperature at which mass loss begins, the completion temperature corresponds to the temperature at which mass loss is complete, and water has been fully removed from the structure.

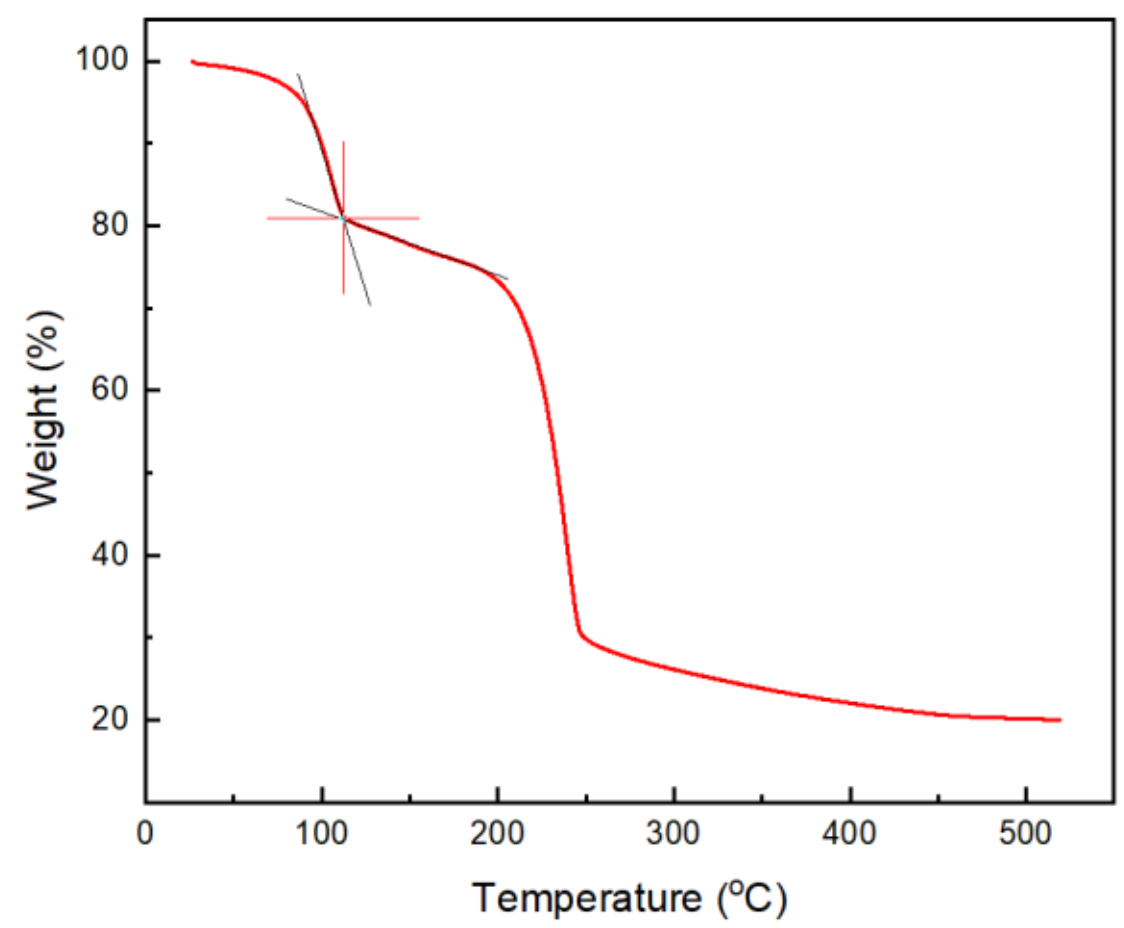

Figure S206 TGA of **SIFSIX-3-Ni** at a 2 °C/min ramp rate. The crosshairs mark the completion temperature.

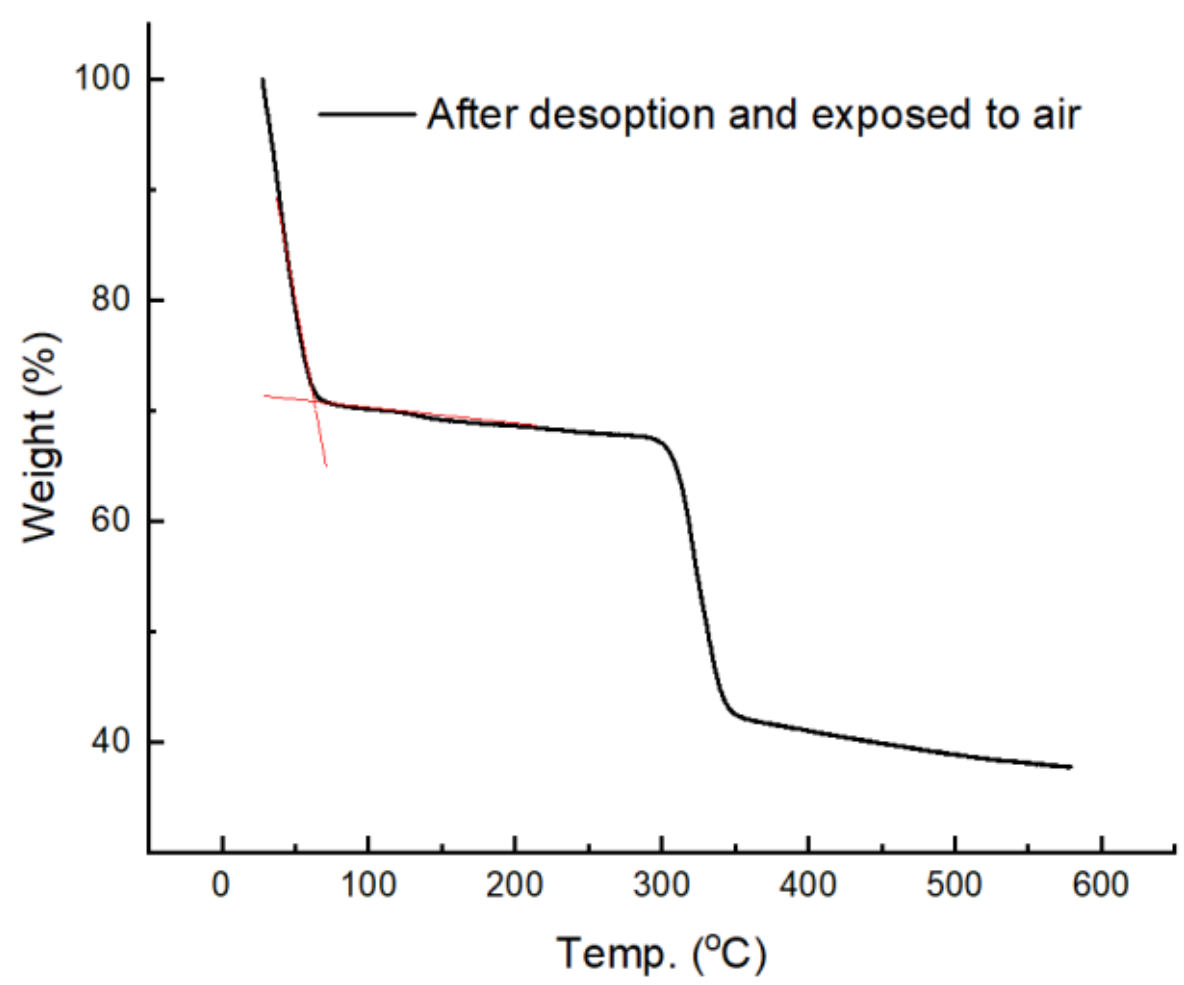

Figure S207 TGA of **HKUST-1** at a 2 °C/min ramp rate. The crosshairs mark the completion temperature.

Table S12 Materials of this study and the experimental thermal stability of water in each system as measured by the completion temperature.

Material	Completion Temperature [°C]
SIFSIX-3-Ni	112.0
TIFSIX-25-Ni	51.4
SIFSIX-25-Ni	61.7
TIFSIX-25-Co	47.2
SIFSIX-25-Co	53.5
SIFSIX-25-Cu	52.3
TIFSIX-25-Cu	52.2
HKUST-1	100.2

23.5 Validation from Experiment

Reported in Table S13 are the percent differences in unit cell lengths, angles, and metal-O_{H2O} bond lengths relative to the experiment. These serve as indicators of how well the chosen functional describes the interatomic interactions within each system. Most deviations are well below 2%, with the largest being the cell angle in **SIFSIX-3-Ni** at 10-11% (not unexpected given the nature of its structural reconstruction) and the hydrated metal-O_{H2O} bond length of **HKUST-1** at 6.19%. Both remain well within the deviations expected for DFT calculations against experiment, confirming that the methodology is suitable for energetic analysis of these systems.

23.6 Results: Thermodynamic Handles

Table S14 summarizes, for each material, the completion temperature for water degassing, $|\Delta E_{\alpha\leftrightarrow\alpha_I}|$, $|\Delta E_{\alpha_I\leftrightarrow\beta}|$, and the overall $|\Delta E_{\alpha\leftrightarrow\beta}|$, each of which are normalized by *W*, alongside a summary of experimental observations. Energetics for hypothetical phases are presented separately in Table S15.

23.7 Results: Kinetic Handles

The ΔPLD, density, and changes in density between phase transitions are summarized for both experimentally observed and theoretical structures below in Table S16, serving as kinetic handles to complement the thermodynamic analysis below.

Table S13 The corresponding number of metals (M) in each unit cell, imposed multiplicity (S), number of waters per metal (W), and DFT results compared to experimental results with percent difference. NEO stands for "Not Experimentally Observed." RFAS stands for "Refined from Alternative Structure," distinguishing structures that have no single crystal structure data but are supported by other experimental methods described in the discussion of this study.

Material	Phase	M	S	W	% Diff. DFT from Exp. Lattice Lengths (a, b, c)	% Diff. DFT from Exp. Lattice Angles (α, β, γ)	M-O(H_2O) Bond [Å] (% Diff. DFT vs. Exp.)
SIFSIX-25-Ni	α	4	9	2	-1.16%, -1.09%, -4.84%	-0.14%, -3.39%, +0.01%	2.104 (+0.57%)
	β	4	9	0	-0.06%, +0.55%, +1.16%	+0.03%, +0.42%, 0.00%	---
TIFSIX-25-Ni	α	4	9	2	+1.00%, -1.97%, -2.85%	0.00%, -3.08%, 0.00%	2.130 (+0.91%)
	β	4	9	0	+0.23%, +0.44%, +0.92%	0.00%, +0.31%, 0.00%	---
SIFSIX-25-Co	α	4	5	2	+1.04%, -3.00%, -0.19%	+0.03%, -1.21%, -0.02%	2.243 (+5.04%)
	β	4	5	0	-0.83%, -1.15%, +2.63%	0.00%, +2.78%, 0.00%	---
TIFSIX-25-Co	α	4	5	2	-1.46%, -1.92%, -3.07%	+0.74%, -1.00%, -0.42%	2.240 (+4.70%)
	β	4	5	0	+4.08%, -3.91%, +2.05%	0.00%, -0.36%, 0.00%	---
SIFSIX-25-Cu	α	4	5	2	NEO	NEO	2.308 (NEO)
	β	4	5	0	+0.07%, +0.99%, +0.94%	0.00%, +0.24%, 0.00%	---
TIFSIX-25-Cu	α	4	5	2	NEO	NEO	2.343 (NEO)
	β	4	5	0	-0.06%, +0.55%, +1.16%	+0.03%, +0.42%, 0.00%	---
SIFSIX-3-Ni	α	2	5	2	RFAS	RFAS	2.153 (RFAS)
	β	1	3	0	-0.06%, +0.55%, +1.16%	+0.03%, +0.42%, 0.00%	---
HKUST-1	α	48	49	1	+1.25%, +0.92%, +1.49%	-0.03%,+0.12%,-0.08%	2.303 (+6.19%)
	β	48	49	0	+1.05%, +1.05%, +1.05%	0.00%, 0.00%, 0.00%	---

Table S14 Material properties, experimental completion temperature, their corresponding computational energies of their phase transitions between α and β normalized by the number of aqua ligands bound to the metal, W, and notes from experimental observations on the nature of their phase transition capabilities.

**Material**	**W**	**Compl. Temp. [°C]**	$\Delta E_{\alpha\leftrightarrow\alpha_I}$ / W [kJ mol^{-1}]	$\Delta E_{\alpha_I\leftrightarrow\beta}$ / W [kJ mol^{-1}]	$\Delta E_{\alpha\leftrightarrow\beta}$ / W [kJ mol^{-1}]	**Phase Transition Capability**
SIFSIX-3-Ni	2	112.0	154.84	81.61	76.83	Reversible (high temp) α ↔ β
TIFSIX-25-Ni	2	51.4	158.08	103.73	53.96	Reversible α ↔ β
SIFSIX-25-Ni	2	61.7	152.62	93.81	58.10	Reversible α ↔ β
TIFSIX-25-Co	2	47.2	127.43	88.59	38.84	Reversible (high RH) α ↔ β
SIFSIX-25-Co	2	53.5	115.67	73.15	42.55	Only α → β
HKUST-1	1	100.2	53.76	0.05	53.71	None (Rigid)

Table S15 Energetics of hypothetical phases are presented. *W* is the number of waters per metal, the energetic magnitude for $\alpha \leftrightarrow \alpha_I$, the energetic magnitude for $\alpha_I \leftrightarrow \beta$, the overall energetic magnitude for $\alpha \leftrightarrow \beta$, each of which are normalized by *W*.

**Material**	***W***	$\Delta E_{\alpha\leftrightarrow\alpha_I}$ / W [kJ mol^{-1}]	$\Delta E_{\alpha_I\leftrightarrow\beta}$ / W [kJ mol^{-1}]	$\Delta E_{\alpha\leftrightarrow\beta}$ / W [kJ mol^{-1}]
SIFSIX-25-Cu	2	106.25	65.48	44.77
TIFSIX-25-Cu	2	112.73	66.24	46.50

Table S16 Table of density and Pore Limiting Diameter (PLD) between the α phase and β phase.

**Material**	***W***	**α Density [g cm^{-3}]**	**β Density [g cm^{-3}]**	**α PLD [Å]**	**β PLD [Å]**	**ΔPLD [Å]**	**Δ Density [g cm^{-3}]**
SIFSIX-3-Ni	2	2.07	1.511	0.79	3.85	3.06	-0.56
HKUST-1	1	0.92	0.86	5.06	6.39	1.33	-0.06
SIFSIX-25-Ni	2	1.23	1.20	4.00	4.00	0.00	-0.03
TIFSIX-25-Ni	2	1.23	1.22	4.27	4.14	-0.13	-0.01
TIFSIX-25-Co	2	1.26	1.24	4.26	4.16	-0.1	-0.02
SIFSIX-25-Co	2	1.25	1.23	4.24	3.91	-0.33	-0.02
TIFSIX-25-Cu	2	1.25	1.22	4.31	4.26	-0.05	-0.03
SIFSIX-25-Cu	2	1.26	1.20	4.27	4.11	-0.16	-0.06

23.8 Discussion of Computational Results

23.8.1 The Transformation Mechanism is Concerted

The $\left|\Delta E_{\alpha\leftrightarrow\alpha_I}\right|$ is the total energy cost for the system to remove or adsorb all waters from either side of the metal. For **HKUST-1** in the $\alpha \rightarrow \alpha_I$ direction, this simply measures the energy to break the water-metal bond (+53.76 kJ/mol), which is the lowest compared to all other materials in this study. These values in the **M'FSIX-L-M** family are significantly higher (**TIFSIX-25-Co**: +127.43 kJ mol^{-1}, **TIFSIX-25-Ni**: +158.08 kJ mol^{-1}, **SIFSIX-25-Co**: +115.67 kJ mol^{-1}, **SIFSIX-25-Ni**: +152.62 kJ mol^{-1}), because this measure is not only breaking water-metal bonds, but also breaking water-anion bonds, i.e. a metal-water-anion bond network which is highlighted in Figure S208a.

$\left|\Delta E_{\alpha_I\leftrightarrow\beta}\right|$ is effectively a measure of the process to structurally transform between phases, which is negligible for a rigid system like HKUST-1 (0.05 kJ/mol). In the $\alpha_I \rightarrow \beta$ direction, this process is highly stabilizing for the **M'FSIX-L-M** family largely due to the formation of metal-anion bond network highlighted in Figure S208b. Note that the $[TiF_6]^{2-}$ anion consistently offers a higher reward for collapsing the pore and forming the anion-metal pairs than $[SiF_6]^{2-}$ ($\alpha_I \rightarrow \beta$; **TIFSIX-25-Co**: -177.18 kJ mol^{-1}, **TIFSIX-25-Ni**: -207.44 kJ mol^{-1}, **SIFSIX-25-Co**: -146.23 kJ mol^{-1}, **SIFSIX-25-Ni**: -187.61 kJ mol^{-1}).

The exact reaction coordinate between $\alpha \rightarrow \beta$ in **M'FSIX-L-M** is not obvious, given how the energy required to break the metal-water-anion bond network is 115.67-158.08 kJ mol^{-1}, and yet in the experiment, it still breaks with relatively little heating (50 - 60 °C). This supports that the mechanism by which this family of materials undergo is a concerted as opposed to step-wise. In other words, the metal forms the bond with the incoming anion at the same time it breaks the bond with the coordinated water, and the massive structural relaxation energy is used in bond breaking.

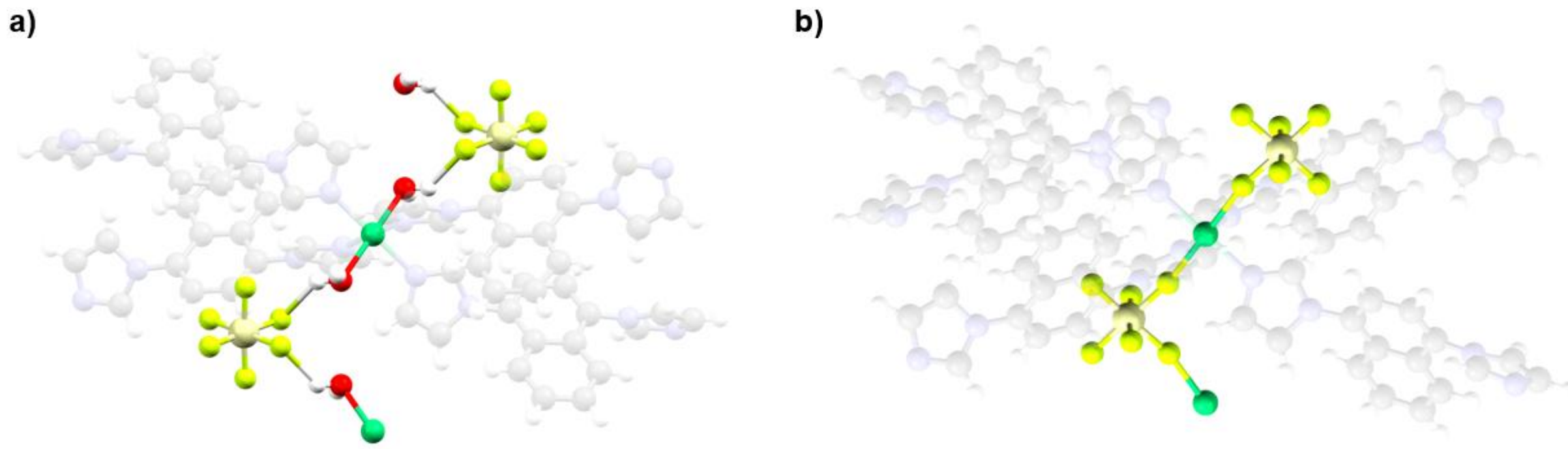

Figure S208 The repeating units of (a) the metal-water-anion network present in the *α* structure and (b) the metal-anion network present in the *β* structure. The **25** linker is included and faded for clarity of the structure. Green is nickel, red is oxygen, white is hydrogen, beige is silicon, and yellow is fluorine.

23.8.2 Theoretical sql-M'FSIX-25-Cu Structures

There are the theoretical **sql-M'FSIX-25-Cu** structures which lie out at a higher magnitude of $\left|\Delta E_{\alpha\leftrightarrow\alpha_I}\right|$. These were the target phases for M'FSIX-25-Cu but formed with poor crystallinity. Cu^{2+} has the smallest charge density (116 C mm^{-3}) compared to Co^{2+} (134 C mm^{-3}) and low-spin Co^{2+} (155 C mm^{-3})[46] which does not lend itself to comparably strong electrostatic interactions with incoming aqua ligands. Since the inorganic node is an octahedral Cu center, it is also the most prone toward Jahn-Teller effects,[47] distorting the axial positions, i.e. the aqua ligands, unfavorably farther away relative to Ni^{2+} and Co^{2+}. This is reflected in the high $\left|\Delta E_{\alpha\leftrightarrow\alpha_I}\right|$ but low $\left|\Delta E_{\alpha_I\leftrightarrow\beta}\right|$ relative to the **sql-M'FSIX-25-Ni** and **sql-M'FSIX-25-Co**.

23.8.3 Energetic Trade and Kinetically-Favorable **25** Linker Allow **TIFSIX-25-Ni** to Reversibly Transform at Low Temperatures

TIFSIX-25-Ni shows the most promise as a practical water-sorbing material such as having the steepest water isotherm slope and being the most reversible, i.e. the most stable in both adsorption and desorption with cycling. **TIFSIX-25-Ni** shares a similar Completion Temperature compared to the other **M'FSIX-L-M** members at 51.4 °C, however it is unclear why.

From a thermodynamic perspective, **TIFSIX-25-Ni** is the most energy intensive system of any material in this study. It requires 316.88 kJ mol^{-1} per metal center for the $\alpha \rightarrow \beta$ transformation and 207.44 kJ mol^{-1} per metal center for $\beta \rightarrow \alpha$. **TIFSIX-25-Ni** requires the most amount of energy to

break the metal-water-anion bond network in the α phase but is the most stabilized by forming the metal-anion bonds in the β phase. While it is costly to break bonds, there is a sufficient energetic trade to achieve reversibility.

The energetic trade-off explains why reversibility is achieved but does not explain why M'FSIX-L-M maintains lower Completion Temperatures (**TIFSIX-25-Co**: 47.2 °C, **TIFSIX-25-Ni**: 51.4 °C, **SIFSIX-25-Co**: 53.5 °C, **SIFSIX-25-Ni**: 61.7 °C) than **HKUST-1** (100.2 °C). The net energetic cost, $\Delta E_{\alpha\rightarrow\beta}$, of **M'FSIX-25-M** systems (**TIFSIX-25-Co**: +38.84 kJ mol^{-1}, **TIFSIX-25-Ni**: +53.96 kJ mol^{-1}, **SIFSIX-25-Co**: +42.55 kJ mol^{-1}, **SIFSIX-25-Ni**: +58.10 kJ mol^{-1}) is only just below or still on par with that of **HKUST-1** (+53.71 kJ/mol) despite having lower Completion Temperatures than HKUST-1. For comparison, **SIFSIX-3-Ni** has a higher Completion Temperature (112.0 °C) than HKUST-1 and commensurately has a higher $\Delta E_{\alpha\rightarrow\beta}$ (+76.83 kJ mol^{-1}). This is where kinetic factors need to be considered.

The kinetic component is exemplified by **SIFSIX-3-Ni** and **SIFSIX-25-Ni** in Figure S209 where both must undergo a dramatic physical change between phases. The smallest pores in **SIFSIX-3-Ni** expand from 0.79 Å in diameter for α to 3.85 Å for the β. In contrast, **SIFSIX-25-Ni** only must contract its smallest pores from a diameter of 4.24 Å to 3.91 Å. Note that the PLD of α will be inflated relative to the β due to coordinated water. For example, the rigid HKUST-1 expands from 5.06 Å in α to 6.39 Å in β. The structure adjusts very minimally upon relaxation, so this expansion of 1.33 Å is solely due to water loss and not structural reorganization. Large deviations from 1.33 Å indicate physical transformations beyond the loss of water. Hence, ΔPLD serves as a measure of how much a system had to physically expand or contract upon the phase transition, including the adjustment for loss of coordinated water.

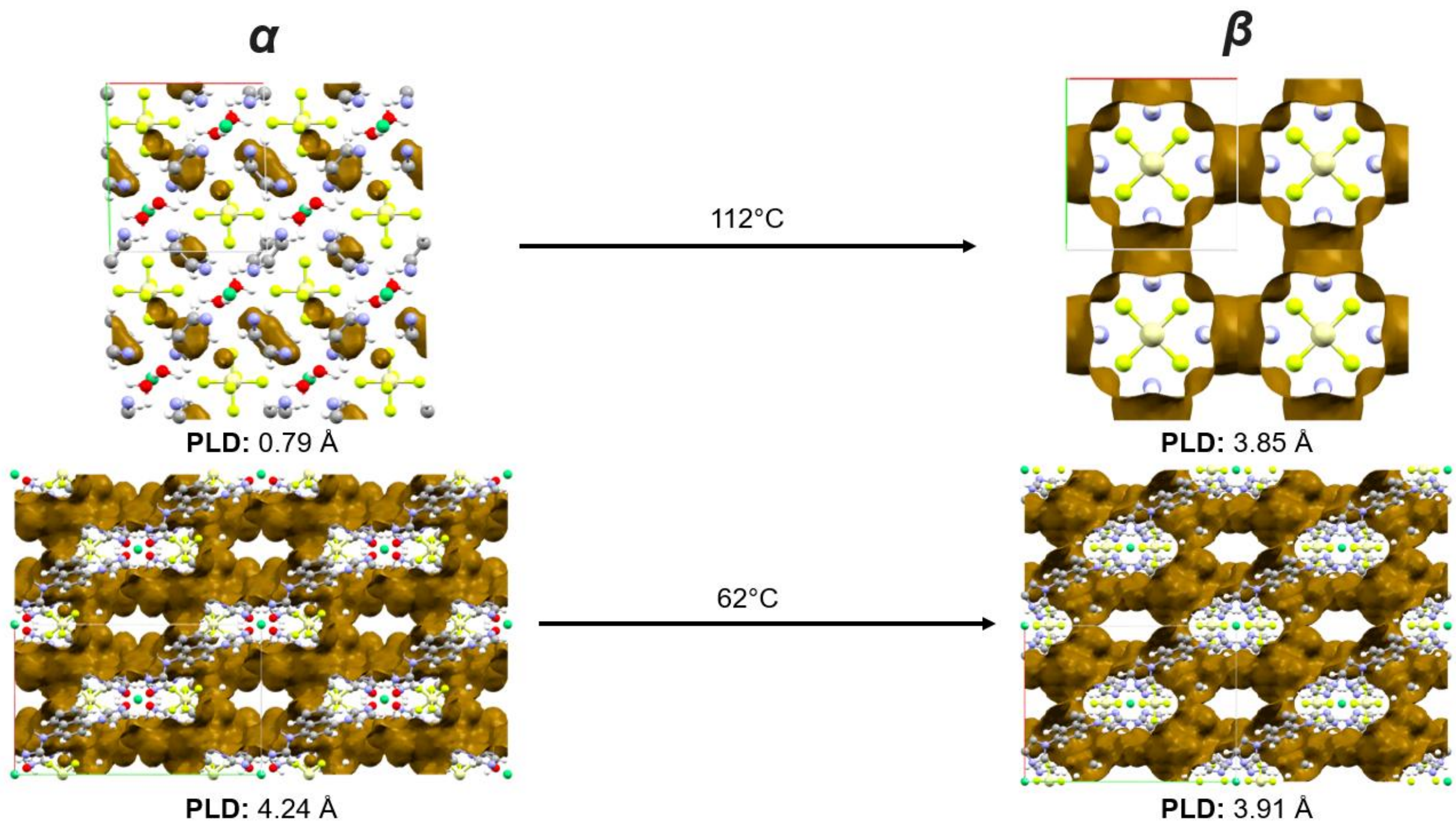

Figure S209 Contrasting the differences between the α-phases (left) and β-phases (right) of SIFSIX-3-Ni (top) and SIFSIX-25-Ni (bottom). The edges of the pores are marked with brown surfaces.

Highlighted in Figure S210 are the correlated magnitudes of these differences in pore limiting diameter (ΔPLD) and density α. Larger physical changes in the structure require more kinetic energy to transition between those phases. The **3** linker is small and compact, so this allows the **SIFSIX-3-Ni** material to form dense phases that then need to expand dramatically (ΔPLD = 3.06 Å). The **25** linker on the other hand is large and bulky, and while they do flex to achieve the phase transition, the PLD remains the same, with ΔPLD around 0 Å for W = 2.

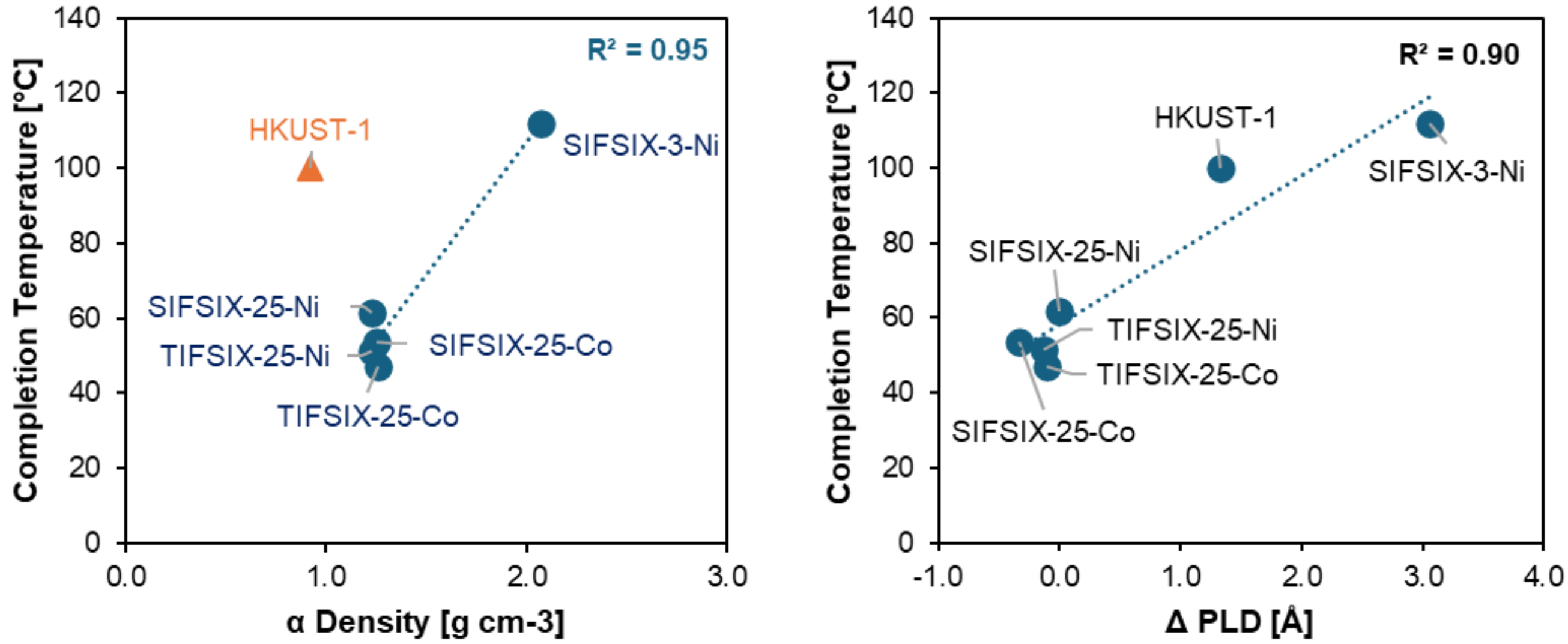

Figure S210 (a) Completion temperature vs. the density of the hydrated phase (W=2 for M'FSIX-L-M, W=1 for HKUST-1). The **M'FSIX-L-M** family is marked in purple circles, and HKUST-1 is a green triangle. Linear regression of **M'FSIX-L-M** series gives an R^2 of 0.95. (b) Comparison between completion temperature and ΔPLD, for which linear regression gives an R^2 of 0.90.

This structural scaffolding provided by the **25** linker between phases is essential to the low completion temperature of these materials. Figure S209 illustrates how maintaining an open pore allows most of the phase-driving dynamic motions of the metal-water-anion network to move freely in a spacious area rather than having to push through a dense phase in the case of the **3** linker. The **M'FSIX-L-M** family has a unique structure where the anion can behave as a 'potential energy slingshot,' where coordinated water is the "pin" holding the phase transition at bay. When that pin is broken, i.e. the water breaks free of the metal-water-anion network, the phase transition triggers, and the anion simultaneously forms the metal-anion bond with a stabilizing force proportional to the measured $|\Delta E_{\alpha_I \leftrightarrow \beta}|$. The **25** linker maintains the integrity of the pore in both phases, which allows coordinated water higher degrees of freedom. On the other hand, the **3** linker creates a much denser α phase, restricting coordinated waters' degrees of freedom to leave, thus requiring much more kinetic energy to proceed through the phase transition relative to the **M'FSIX-25-M** materials.

In conclusion, having a sufficient energetic trade-off to maintain reversibility, choosing ligands essential to the phase transition that are simultaneously non-essential to keeping the pores open (i.e. aqua ligands and anions), and selecting linkers that allow said ligands sufficient degrees of freedom during transformation (e.g. **25** linker) permits the low-temperature activation and stable water adsorption in the **M'FSIX-L-M** family.

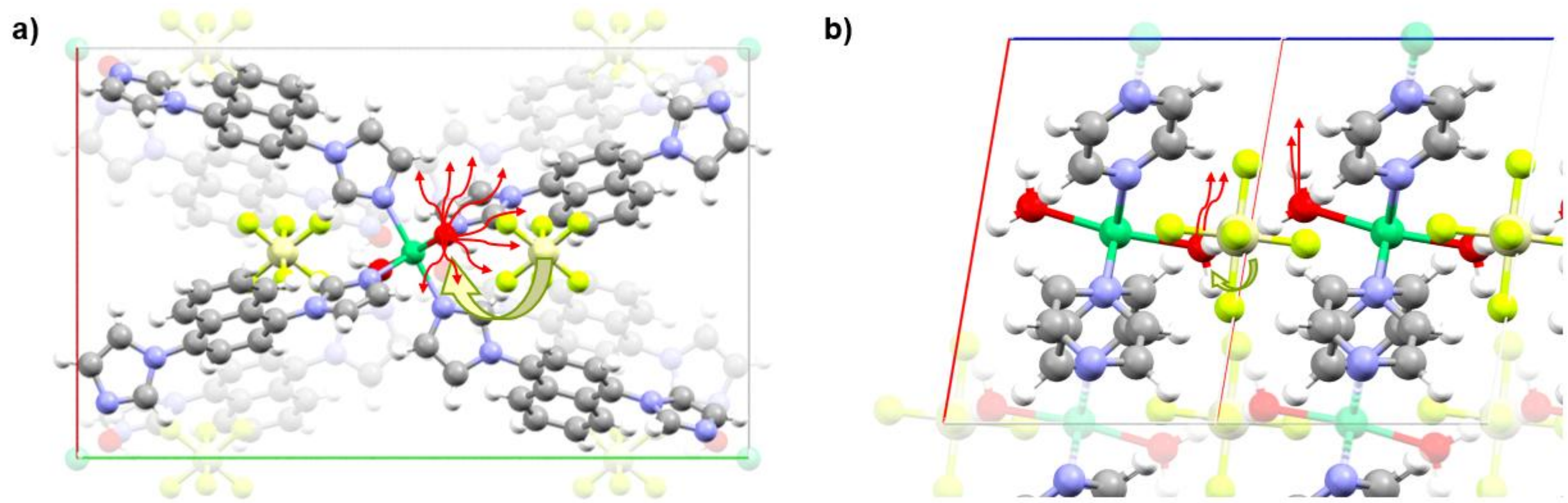

Figure S211 Increasing number of arrows demonstrate how many more degrees of freedom coordinated water has in the (a) **SIFSIX-25-Ni** α structure compared to the denser (b) **SIFSIX-3-Ni** α structure. Increasing numbers of red arrows are illustrating increased motion in the water molecule, and the increasing yellow arrow corresponds to more feasible motion in the $[SiF_6]^{2-}$ anion.

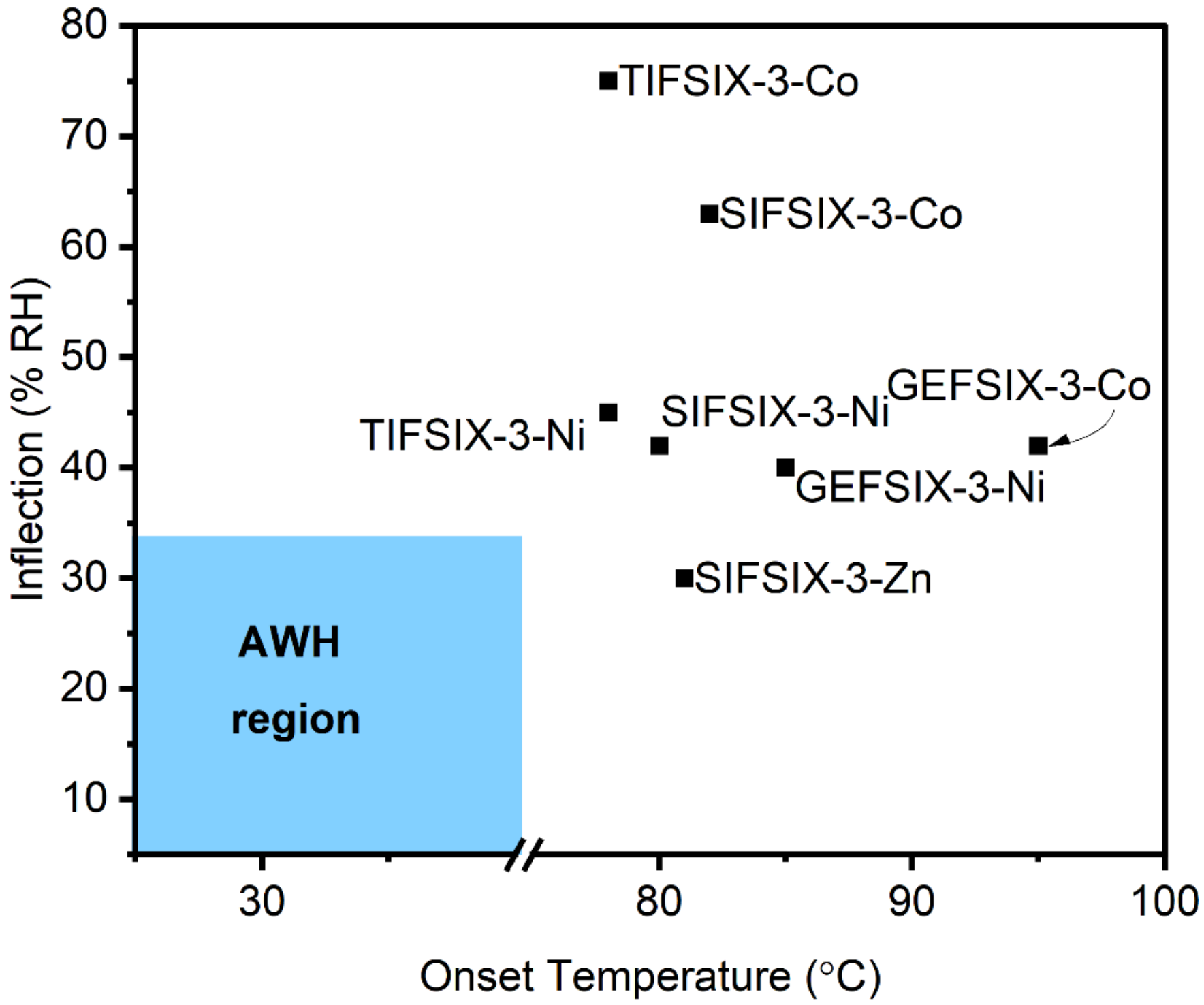

Figure 212 Scatter plot of inflection point RH, determined from dynamic vapor sorption analysis (room temperature), as a function of regeneration temperature of **M'FSIX-3-M** compounds.

24 References

[1] G. M. Sheldrick, *Acta Crystallogr A* **2008**, *64*, 112-122.
[2] A. L. Spek, *Acta Crystallogr. D: Struct. Biol.* **2009**, *65*, 148-155.
[3] H. L. Wang, L. B. Feng, Z. D. Zhang, G. I. Webb, D. H. Lin, J. N. Song, *Sci. Rep.* **2016**, *6*.
[4] G. M. Sheldrick, *Acta Crystallogr A* **2015**, *71*, 3-8.
[5] O. V. Dolomanov, L. J. Bourhis, R. J. Gildea, J. A. K. Howard, H. Puschmann, *J. Appl. Crystallogr.* **2009**, *42*, 339-341.
[6] C. R. Groom, I. J. Bruno, M. P. Lightfoot, S. C. Ward, *Acta Crystallogr. B: Struct. Sci. Cryst. Eng. Mater.* **2016**, *72*, 171-179.
[7] I. J. Bruno, J. C. Cole, P. R. Edgington, M. Kessler, C. F. Macrae, P. McCabe, J. Pearson, R. Taylor, *Acta Crystallogr. B: Struct. Sci. Cryst. Eng. Mater.* **2002**, *58*, 389-397.
[8] C. F. Macrae, P. R. Edgington, P. McCabe, E. Pidcock, G. P. Shields, R. Taylor, M. Towler, J. van De Streek, *J. Appl. Crystallogr.* **2006**, *39*, 453-457.
[9] G. S. Zhu, D. Wang, Z. H. Xu, L. Li, C. L. Song, W. T. Zhao, H. Q. Zhao, B. Y. Yu, Y. Jia, *J. Mol. Struct.* **2024**, *1301*.
[10] Z. P. Wang, Y. Wang, X. Y. Li, L. F. Jia, A. Z. Yang, W. T. Zhao, Y. Jia, B. Y. Yu, H. Q. Zhao, *J. Mol. Struct.* **2024**, *1305*, 137699.
[11] B. Y. Yu, Z. P. Wang, H. Y. Ding, F. F. Miao, A. Z. Yang, W. T. Zhao, Y. Jia, H. Q. Zhao, *J. Mol. Struct.* **2023**, *1291*, 135891.
[12] Y. H. Andaloussi, D. Sensharma, A. A. Bezrukov, D. C. Castell, T. He, S. Darwish, M. J. Zaworotko, *Cryst. Growth Des.* **2024**, *24*, 2573-2579.
[13] L. Yang, X. Li, C. Y. Sun, H. Wu, C. G. Wang, Z. M. Su, *New J. Chem.* **2017**, *41*, 3661-3666.
[14] M. H. Mohamed, S. K. Elsaidi, L. Wojtas, T. Pham, K. A. Forrest, B. Tudor, B. Space, M. J. Zaworotko, *J. Am. Chem. Soc.* **2012**, *134*, 19556-19559.
[15] P. Nugent, Y. Belmabkhout, S. D. Burd, A. J. Cairns, R. Luebke, K. Forrest, T. Pham, S. Q. Ma, B. Space, L. Wojtas, M. Eddaoudi, M. J. Zaworotko, *Nature* **2013**, *495*, 80-84.
[16] X. F. Li, H. Bian, W. Q. Huang, B. Y. Yan, X. Y. Wang, B. Zhu, *Coord. Chem. Rev.* **2022**, *470*, 214714.
[17] B. Q. Song, Q. Y. Yang, S. Q. Wang, M. Vandichel, A. Kumar, C. Crowley, N. Kumar, C. H. Deng, V. GasconPerez, M. Lusi, H. Wu, W. Zhou, M. J. Zaworotko, *J. Am. Chem. Soc.* **2020**, *142*, 6896-6901.
[18] D. J. O'Hearn, D. Sensharma, A. Raza, A. A. Bezrukov, M. Vandichel, S. Mukherjee, M. J. Zaworotko, *Chem. Sci* **2024**, *15*, 17937-17943.
[19] A. Kumar, C. Hua, D. G. Madden, D. O'Nolan, K. J. Chen, L. A. J. Keane, J. J. Perry, M. J. Zaworotko, *Chem. Commun.* **2017**, *53*, 5946-5949.
[20] D. G. Madden, D. O'Nolan, K. J. Chen, C. Hua, A. Kumar, T. Pham, K. A. Forrest, B. Space, J. J. Perry, M. Khraisheh, M. J. Zaworotko, *Chem. Commun.* **2019**, *55*, 3219-3222.
[21] Z. Q. Zhang, Q. Ding, S. B. Peh, D. Zhao, J. Y. Cui, X. L. Cui, H. B. Xing, *Chem. Commun.* **2020**, *56*, 7726-7729.
[22] S. K. Elsaidi, M. H. Mohamed, H. T. Schaef, A. Kumar, M. Lusi, T. Pham, K. A. Forrest, B. Space, W. Q. Xu, G. J. Halder, J. Liu, M. J. Zaworotko, P. K. Thallapally, *Chem. Commun.* **2015**, *51*, 15530-15533.
[23] Z. Q. Zhang, Q. Ding, J. Y. Cui, X. L. Cui, H. B. Xing, *Sci China Mater* **2021**, *64*, 691-697.
[24] M. L. Barsoum, J. Hofmann, H. M. Xie, Z. H. Y. Chen, S. M. Vornholt, R. dos Reis, N. Burns, S. Kycia, K. W. Chapman, V. P. Dravid, O. K. Farha, *J. Am. Chem. Soc.* **2024**, *146*, 6557-6565.
[25] S. K. Elsaidi, M. H. Mohamed, A. S. Helal, M. Galanek, T. Pham, S. Suepaul, B. Space, D. Hopkinson, P. K. Thallapally, J. Li, *Nat. Commun.* **2020**, *11*, 3103.

[26] C. T. Campbell, J. R. V. Sellers, *Chem. Rev.* **2013**, *113*, 4106-4135.
[27] A. A. Bezrukov, D. J. O'Hearn, V. Gascon-Perez, S. Darwish, A. Kumar, S. Sanda, N. Kumar, K. Francis, M. J. Zaworotko, *Cell Rep. Phys. Sci.* **2023**, *4*, 101252.
[28] S. M. Marcus, R. L. Blaine, *Thermochim. Acta* **1994**, *243*, 231-239.
[29] R. L. Blaine, S. M. Marcus, *J. Therm. Anal. Calorim.* **1998**, *54*, 467-476.
[30] ASTM International, *Standard Test Method for Thermal Conductivity and Thermal Diffusivity by Modulated Temperature Differential Scanning Calorimetry (E1952-01)* in *Annual Book of ASTM Standards, Vol. 14.02*, West Conshohocken, **2001**, pp. 1-6.
[31] W. M. Haynes, *Properties of solids* in *CRC Handbook of Chemistry and Physics*, 97 ed., CRC Press New York, **2016**, p. 223.
[32] H. Babaei, M. E. DeCoster, M. Jeong , Z. M. Hassan, T. Islamoglu, H. Baumgart, A. J. H. McGaughey, E. Redel, O. K. Farha, P. E. Hopkins, J. A. Malen, C. E. Wilmer, *Nat. Commun.* **2020**, *11*, 4010.
[33] J. Huang, X. X. Xia, X. J. Hu, S. Li, K. Liu, *Int. J. Heat Mass Transfer* **2019**, *138*, 11-16.
[34] D. A. Shirley, W. F. Giauque, *J. Am. Chem. Soc.* **1959**, *81*, 4778-4779.
[35] Y. N. Lin, Z. Q. Shi, P. L. D. Wildfong, *J. Pharm. Biomed. Anal.* **2010**, *51*, 979-984.
[36] C. C. Nguyen, N. M. Q. Tran, H. T. Lai, T. L. Doan, G. J. Snyder, T. B. Phan, *Chem. Phys. Rev.* **2025**, *6*, 041305.
[37] D. O'Nolan, A. Kumar, M. J. Zaworotko, *J. Am. Chem. Soc.* **2017**, *139*, 8508-8513.
[38] T. D. Kühne, M. Iannuzzi, M. Del Ben, V. V. Rybkin, P. Seewald, F. Stein, T. Laino, R. Z. Khaliullin, O. Schütt, F. Schiffmann, D. Golze, J. Wilhelm, S. Chulkov, M. H. Bani-Hashemian, V. Weber, U. Borstnik, M. Taillefumier, A. S. Jakobovits, A. Lazzaro, H. Pabst, T. Müller, R. Schade, M. Guidon, S. Andermatt, N. Holmberg, G. K. Schenter, A. Hehn, A. Bussy, F. Belleflamme, G. Tabacchi, A. Glöss, M. Lass, I. Bethune, C. J. Mundy, C. Plessl, M. Watkins, J. VandeVondele, M. Krack, J. Hutter, *J. Chem. Phys.* **2020**, *152*, 194103
[39] J. VandeVondele, M. Krack, F. Mohamed, M. Parrinello, T. Chassaing, J. Hutter, *Comput. Phys. Commun.* **2005**, *167*, 103-128.
[40] S. Goedecker, M. Teter, J. Hutter, *Phys Rev B* **1996**, *54*, 1703-1710.
[41] M. Krack, *Theor. Chem. Acc.* **2005**, *114*, 145-152.
[42] J. P. Perdew, K. Burke, M. Ernzerhof, *Phys. Rev. Lett.* **1996**, *77*, 3865-3868.
[43] J. P. Perdew, K. Burke, M. Ernzerhof, *Phys. Rev. Lett.* **1997**, *78*, 1396-1396.
[44] W. Q. You, Y. Liu, J. D. Howe, D. S. Sholl, *J. Phys. Chem. C* **2018**, *122*, 8960-8966.
[45] S. K. Henninger, F. P. Schmidt, H. M. Henning, *Appl. Therm. Eng.* **2010**, *30*, 1692-1702.
[46] G. Rayner-Canham, T. Overton, *Descriptive Inorganic Chemistry*, 5 ed., W. H. Freeman and Company, New York, **2009**.
[47] M. Gerloch, *Inorg. Chem.* **1981**, *20*, 638-640.
